# Novel pyrazole–oxadiazole–chalcone/oxime hybrids as dual EGFR/VEGFR-2 inhibitors with promising anticancer potential: a comprehensive cytotoxicity evaluation, mechanistic insights and SAR analysis

**DOI:** 10.1007/s11030-025-11411-3

**Published:** 2026-01-21

**Authors:** Omar Alshazly, Mohamed Abdel-Aziz, Gamal El-Din A. Abuo-Rahma, Mamdouh F. A. Mohamed

**Affiliations:** 1 Department of Pharmaceutical Chemistry, Faculty of Pharmacy, Sohag University, Sohag, 82524 Egypt; 2 Department of Medicinal Chemistry, Faculty of Pharmacy, Minia University, Minia, 61519 Egypt; 3 Department of Pharmaceutical Chemistry, Faculty of Pharmacy, Deraya University, New-Minia, Egypt; 4 Department of Pharmaceutical Chemistry, Faculty of Pharmacy, New Valley University, New Valley, 72511 Egypt

**Keywords:** Pyrazole, 1,3,4-oxadiazole, Chalcone, anticancer, EGFR inhibitors, VEGFR-2 inhibitors, Nitric oxide.

## Abstract

A novel series of pyrazole–1,3,4-oxadiazole hybrids incorporating chalcone/oxime scaffolds (**10a**–10**c**, **11a**–11**c**, **12a**–12**i**, **13a**–13**i**, and **14a**–**14i**) was synthesized and subjected to an detailed cytotoxicity profile against the NCI-60 human cancer cell line panel. Several derivatives demonstrated broad-spectrum growth inhibition, with compounds **10b**, **11a**, and **11b** emerging as the most active candidates, exhibiting mean GI₅₀ values in the low micromolar range (4.36–16.4 µM). A deeper biological assessment revealed that these hybrids act as dual inhibitors of EGFR and VEGFR-2, with compound **11b** showing the highest potency (IC₅₀ = 26.38 nM and 114.17 nM, respectively). Mechanistic studies further confirmed that **11b** induced G2/M phase arrest and triggered apoptotic pathways in MCF-7 cells. The oxime-containing analogs exhibited enhanced nitric oxide (NO) release, a property associated with modulation of angiogenesis and increased susceptibility of cancer cells to apoptosis, contributing to their observed anticancer activity. Molecular docking and structure–activity relationship analysis clarified the binding interactions and substitution patterns governing activity, and both cytotoxicity and mechanistic analyses converged on compound **11b** as the most promising lead of the series.

**Supplementary Information:**

The online version contains supplementary material available at 10.1007/s11030-025-11411-3.

## Introduction

 With an estimated 15 million deaths per year by 2030, cancer has become a significant global health crisis, posing a serious challenge for healthcare systems worldwide [1, 2]. Traditional anticancer drugs often suffer from limitations such as drug resistance, severe side effects, and lack of selectivity, which further complicate treatment outcomes [3]. As a result, innovative solutions have been explored to address this global health issue As a result, innovative solutions have been explored to address this global health issue [4]. One promising approach in this regard is the development of multitarget or smart hybrid molecules that combine two or more pharmacophores to target cancer [5, 6]. These rationalized hybrid compounds have attracted considerable interest in cancer treatment, as they have the potential to simultaneously inhibit multiple cancer pathways or targets [7–9].

Two promising protein kinases that could be of particular interest for further work are EGFR (Epidermal Growth Factor Receptor) and VEGF (vascular endothelial growth factor) [10, 11]. EGFR is a receptor tyrosine kinase that plays a crucial role in cell proliferation and survival, and its overexpression or mutations have been observed in various cancers, making it an attractive target for cancer therapy [12–14]. Furthermore, the EGFR mediated signaling pathway stimulates the vascular endothelial growth factor (VEGF) which is the main factor inducing tumor angiogenesis. Hence, after binding of VEGF to VEGFR-2 (vascular endothelial growth factor receptor-2) which is abundantly expressed in the endothelial cells of cancer [15], a series of signaling pathways responsible for tumor angiogenesis begins and results in promoting survival, proliferation, migration, and vascular permeability for cancer tissues [16]. From the foregoing, we can infer that the EGFR and VEGFR-2 pathways are closely related; VEGF expression is reduced when EGFR is blocked. Nevertheless, VEGFR-2 inhibition also increases the anticancer effect of EGFR blocking medications [17]. Therefore, targeting EGFR and VEGFR-2 through a dual inhibition is a promising protocol for cancer treatment.

**Fig. 1:**
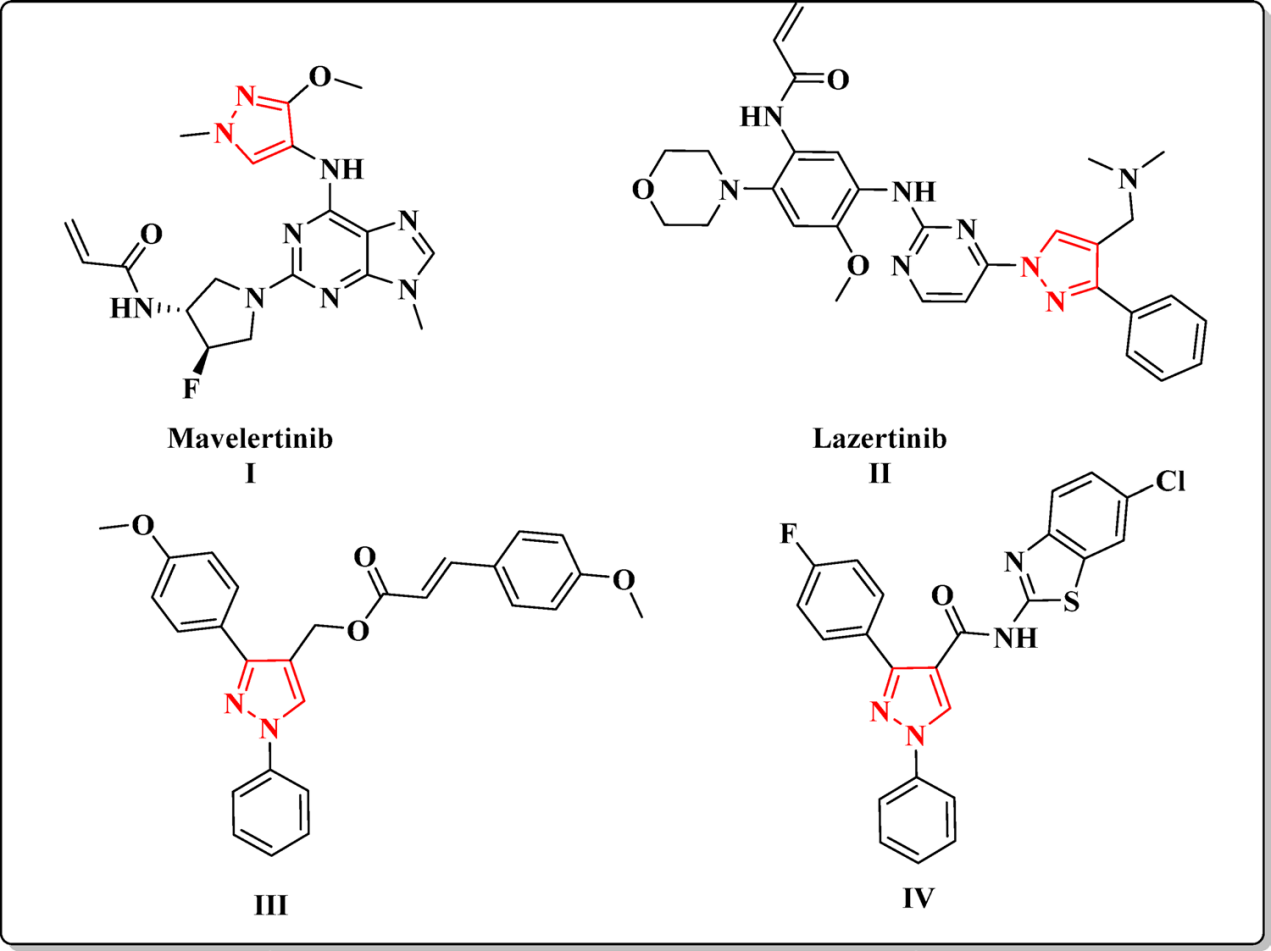
Reported pyrazole derivatives, as EGFR and VEGFR-2 inhibitors as a template

Pyrazole is among the most important heterocyclic compounds incorporated in several drugs and utilized for innovation and discovery of new biologically active candidates that has been reported to exhibit a wide range of pharmacological activities, including anti-inflammatory, analgesic, antipyretic, anticonvulsant, antidepressant, antimicrobial, and antifungal properties [18–21]. pyrazole has gained attention in cancer research due to its potential as an anticancer agent [22, 23]. One of the mechanisms by which pyrazole exhibits its anticancer activity is through the inhibition of kinases, particularly EGFR and VEGFR-2 [24–26]. Pyrazole derivatives have demonstrated inhibitory effects on EGFR, preventing its activation and downstream signaling pathways involved in cancer cell growth and metastasis, and for instance, mavelertinib **I** is a potent EGFR inhibitor and represent promising candidate currently in clinical trials for treating EGFR-mutated cancers [27–30], and lazertinib **II** is an EGFR inhibitor that approved for treatment of non-small cell lung cancer [31]. Similarly, compound **III** showed potent antiproliferative activity against MCF-7 and B16-F10 cell lines with IC_50_ values of 0.30 ± 0.04 µM and 0.44 ± 0.05 µM, respectively with potent inhibitory activity against EGFR with IC_50_ = 0.21 ± 0.05 µM [32]. Moreover, pyrazole-based compounds have shown promising inhibitory effects on VEGFR-2, leading to the suppression of cancer cell proliferation and tumor growth [15, 33]. Moreover, compound **IV** (Fig. 1) showed potent cytotoxicity against all of the tested cancer cell lines, with IC_50_ values ranging from 3.17 to 6.77 µM. Moreover, compound **IV** demonstrated c.

onsiderable in vitro inhibitory effects on VEGFR-2 with IC_50_ value of 97 nM and displayed antiangiogenic properties in an in vivo model using transgenic zebrafish (Tg(flila: EGFP)) [34].

**Fig. 2:**
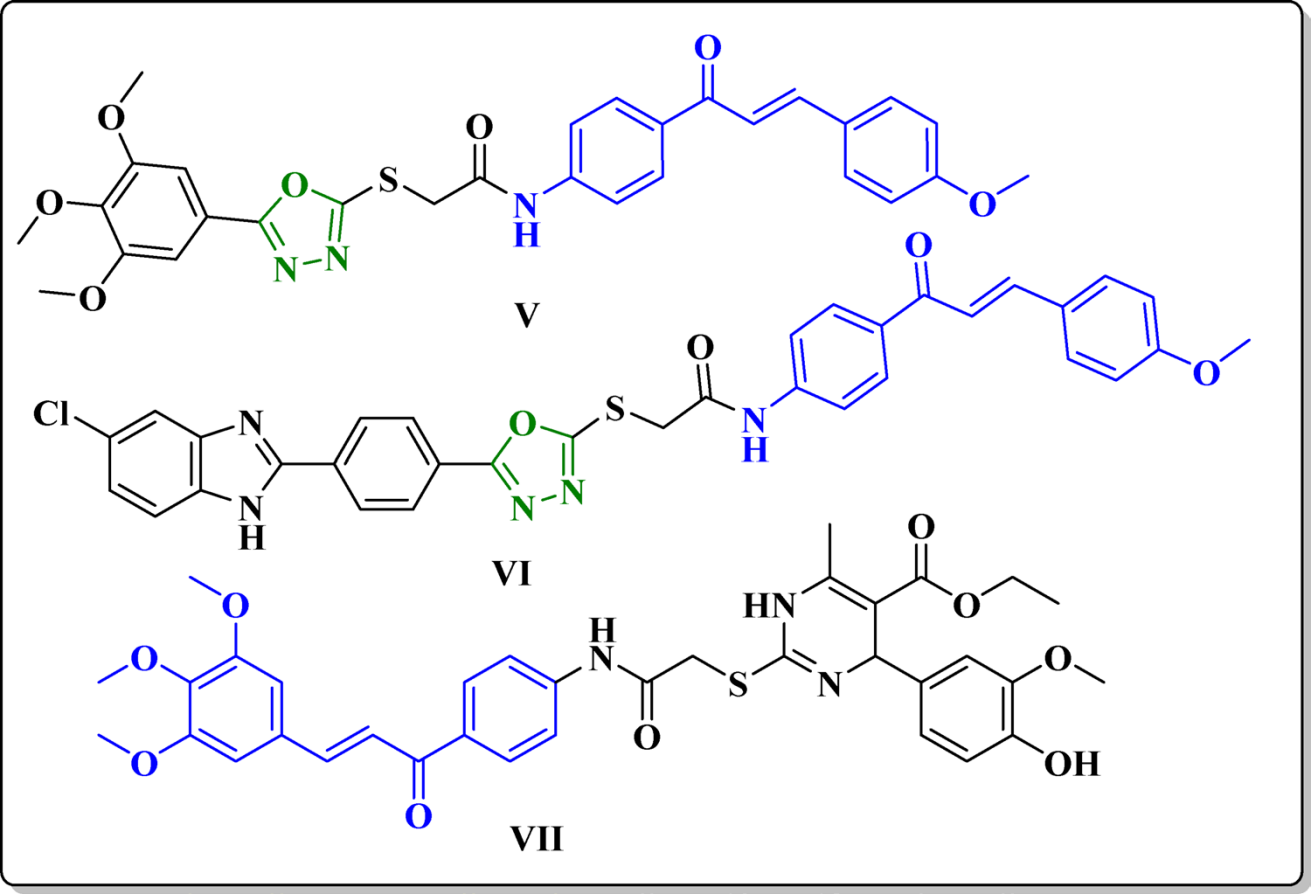
Reported EGFR and VEGFR-2 inhibitors containing chalcone and 1,3,4-oxadiazole moieties as a template

Furthermore, 1,3,4-oxadiazole moiety has displayed remarkable anticancer activity and significant activity against wide range of Kinases specially EGFR inhibition activity [35–39]. Compound **V** (Fig. 2) showed cytotoxic activity with IC_50_ of 1.95 µM, 2.36 µM and 3.45 µM against K-562, Jurkat and KG-1a leukemia cell lines, respectively. Simultaneously inhibiting EGFR with IC_50_ of 0.24 µM [35]. Likewise compound **VI** ( Fig. 2) demonstrated antiproliferation activity against four human cancer cell lines (A-549, MCF‐7, Panc‐1, and HT‐29) with IC_50_ of 1.30, 0.80, 1.20, and 1.13 µM, respectively, and a noteworthy EGFR inhibition with IC_50_ of 1.80 µM [37].

Moreover, chalcones are naturally occurring compounds present in various plant sources and have demonstrated potential as anticancer agents [40–45]. Studies indicates that chalcones act as a Michael acceptor and can inhibit kinases such as EGFR and VEGFR-2, which contributes to their anticancer properties [42, 46]. By targeting these kinases, chalcones can disrupt key signaling pathways involved in cancer cell growth and survival [47, 48]. Compounds **VII** (Fig. 2) was assayed for their antiproliferation activity against NCI cell line panel and showed remarkable activities, and were found to have a potency towards VEGFR-2 kinase comparable to that of sorafenib with a an IC_50_ value of 0.11 µM [49].

**Fig. 3:**
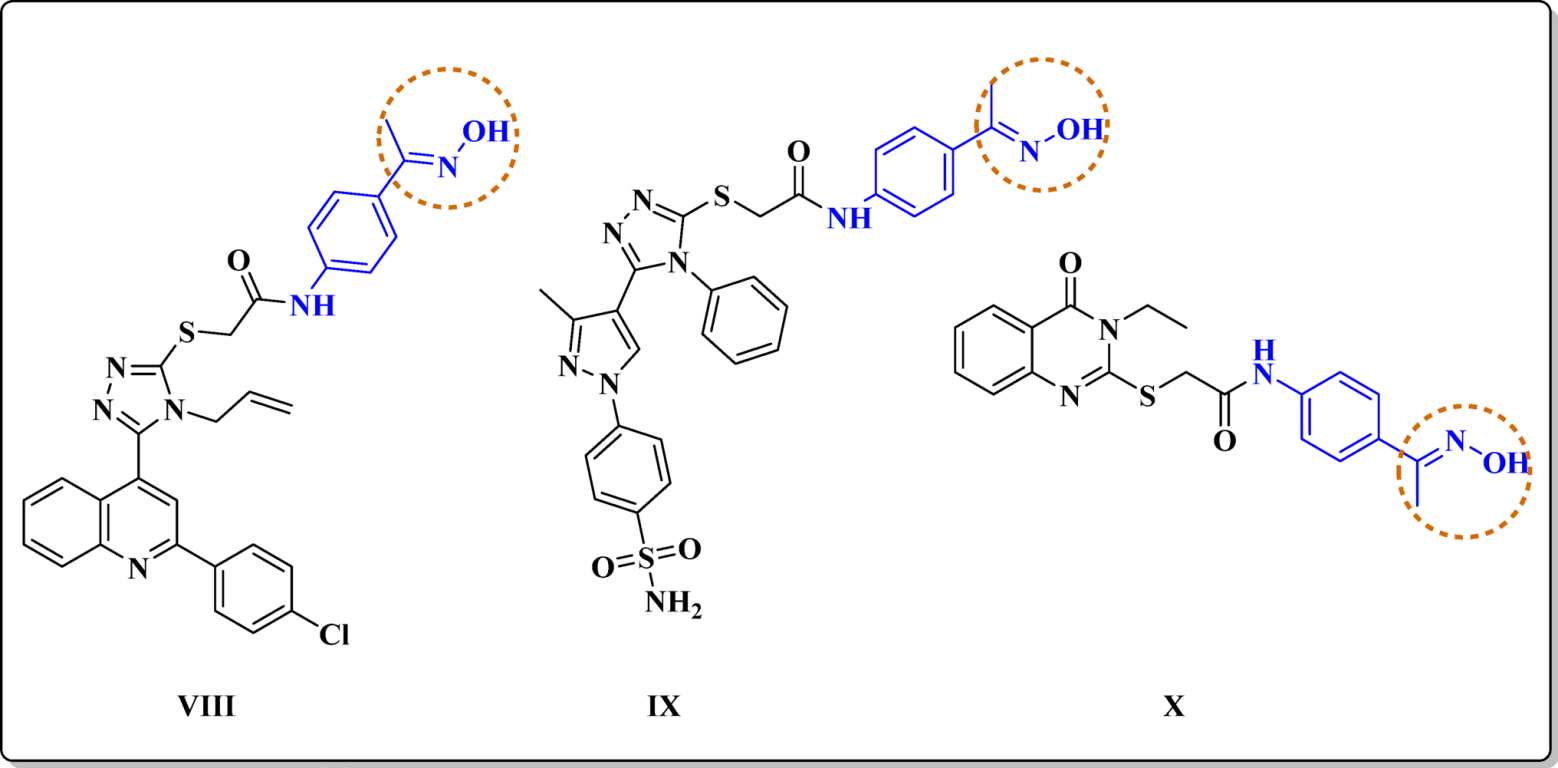
Reported compounds with antiproliferative activity bearing oxime moiety as a template

Furthermore, oximes have emerged as promising nitric oxide (NO) donors with applications in the field of anticancer therapy due to their unique ability to release NO in a controlled manner [50–52]. This release mechanism can induce various cellular responses, including apoptosis in cancer cells, inhibition of tumor growth, and enhancement of the immune response against tumors [53]. By targeting specific pathways, such as those involved in angiogenesis and metastasis, oxime derivatives can disrupt the cancerous environment, making them effective adjuncts to traditional therapies [51]. Moreover, the ability of oximes to selectively deliver NO to tumor tissues while minimizing systemic toxicity further enhances their therapeutic potential [54, 55]. A series of nitric oxide (NO)-releasing quinolone-1,2,4-triazole/oxime hybrids were developed to target STAT3 in melanoma [53]. The most potent compound, **VIII** and **(**Fig. 3**)**, bound to the STAT3-SH domain with IC_50_ value 0.25 µM. They effectively inhibited STAT3 phosphorylation in BRAFV600E melanoma cells and prevented STAT3 nuclear translocation and DNA-binding activity. Furthermore, Fadaly, W. A., et al. [51] synthesized a series of pyrazole/triazole/oxime hybrids, which showed significant antiproliferative activity, particularly the sulphamoyl derivative **IX (**Fig. 3**)**. This compound exhibited IC_50_ value of 0.33 µM against the PC-3 cancer cell line, with selectivity ratios of 39.4-fold over F180 fibroblasts. Moreover, it also effectively inhibited p38MAPK with IC_50_ of 0.58 µM, and VEGFR-2 with IC_50_ of 0.54 µM. Another series of quinazoline-based compounds were designed and synthesized as VEGFR-2 inhibitors [52]. Among these, compound **X (**Fig. 3**)** demonstrated remarkable anti-cancer activity across three human cancer cell lines (HepG-2, MCF-7, and HCT-116). In addition, compound **X** exhibited strong VEGFR-2 inhibitory activity with an IC_50_ of 2.5 µM.

**Fig. 4:**
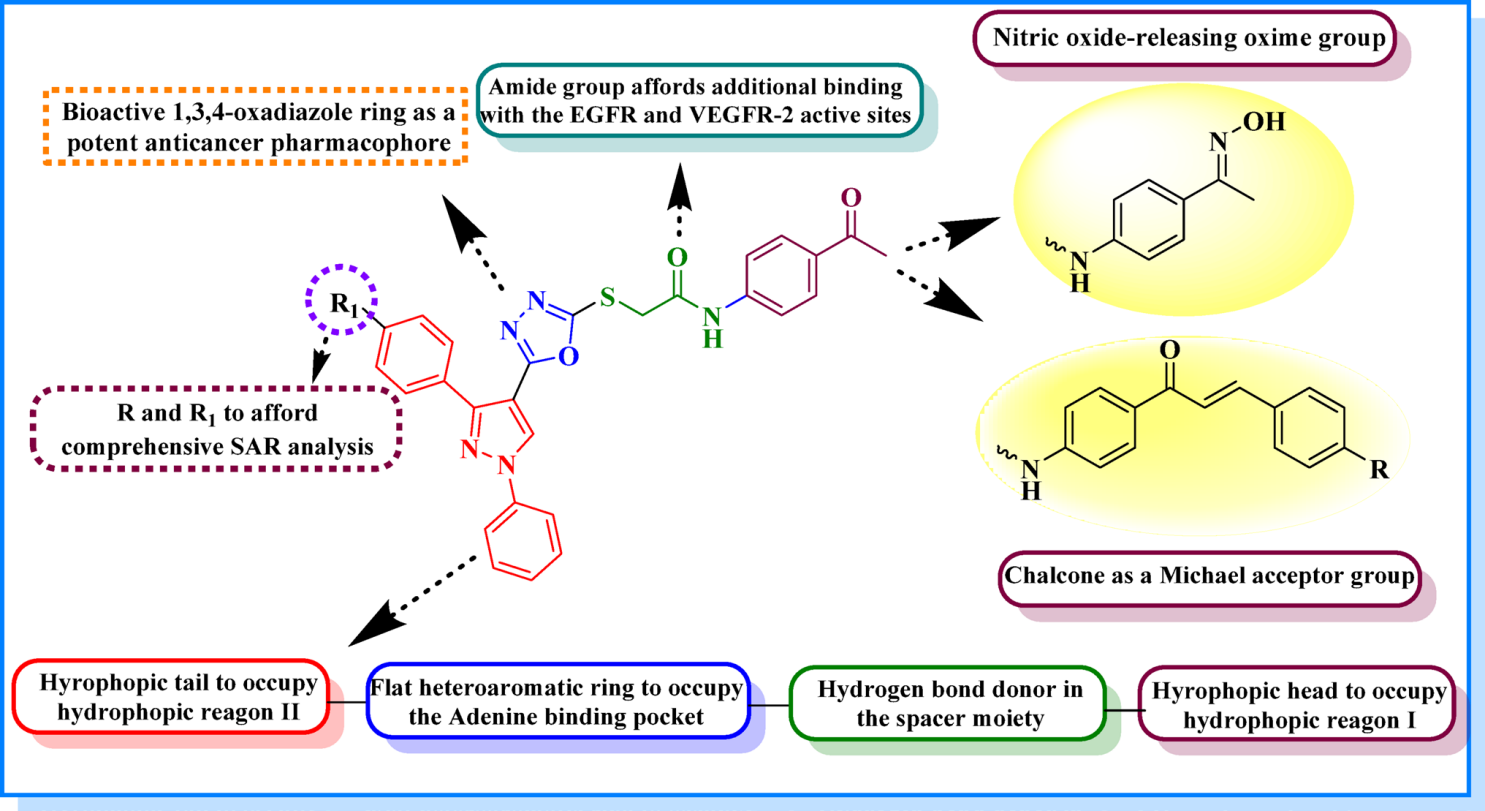
Design of novel pyrazole-1,3,4-oxadiazole hybrids as potential EGFR inhibitors

In this study, the objective was to gather the benefits of combining pyrazole, with 1,3,4-oxadiazole scaffolds into hybrid molecules. The introduction of a chalcone moiety as a Michael acceptor group and incorporating of nitric oxide-releasing oxime group is expected to result in more potent and targeted anticancer candidates, with enhanced binding ability to the target sites (Fig. 4). Aiming that this strategy offers potential advantages such as reduced toxicity, decreased resistance, and enhanced efficacy compared to single scaffold compounds.

## Results and discussion

### Chemistry

**Scheme 1:**
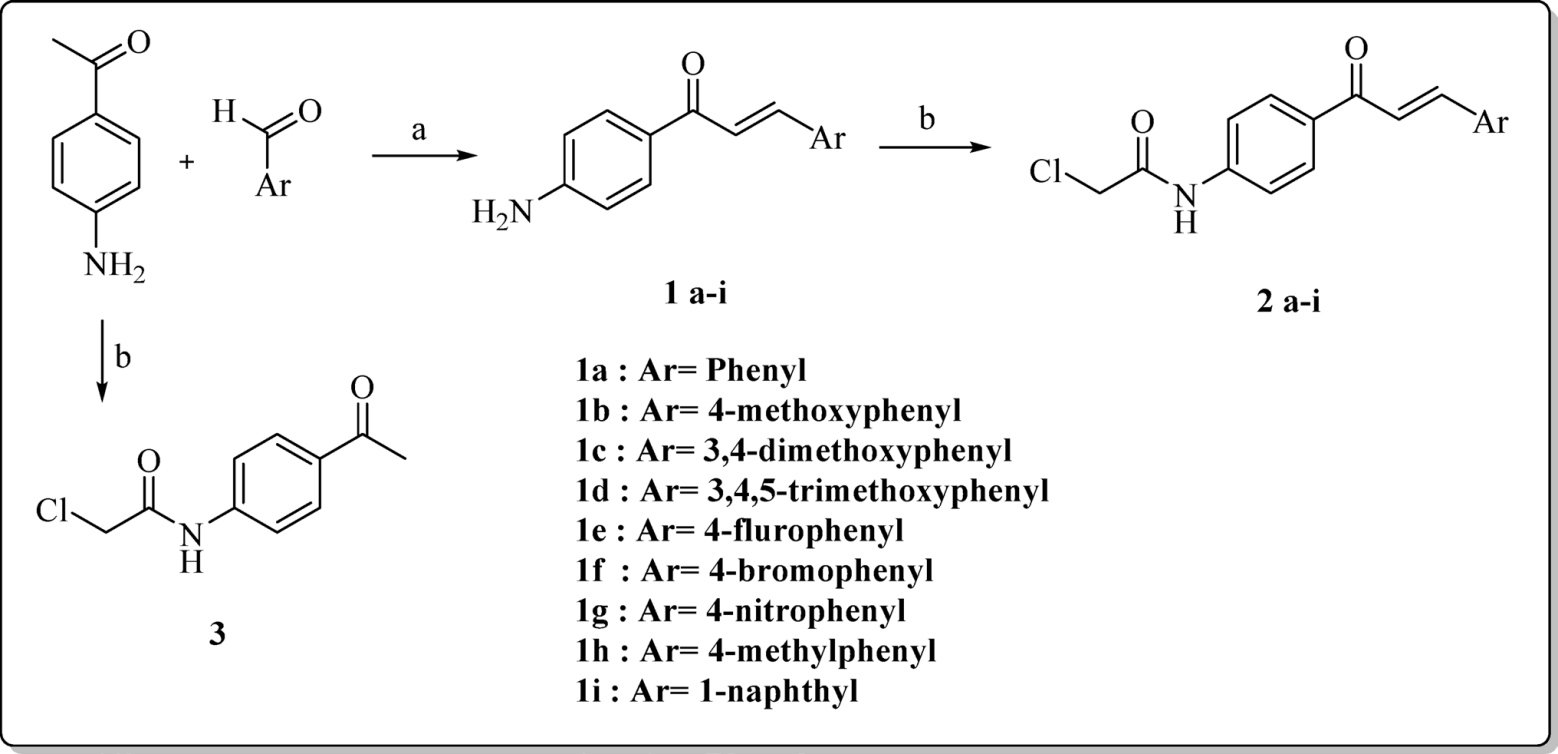
Synthesis of the intermediates **1–3**

**Scheme 2:**
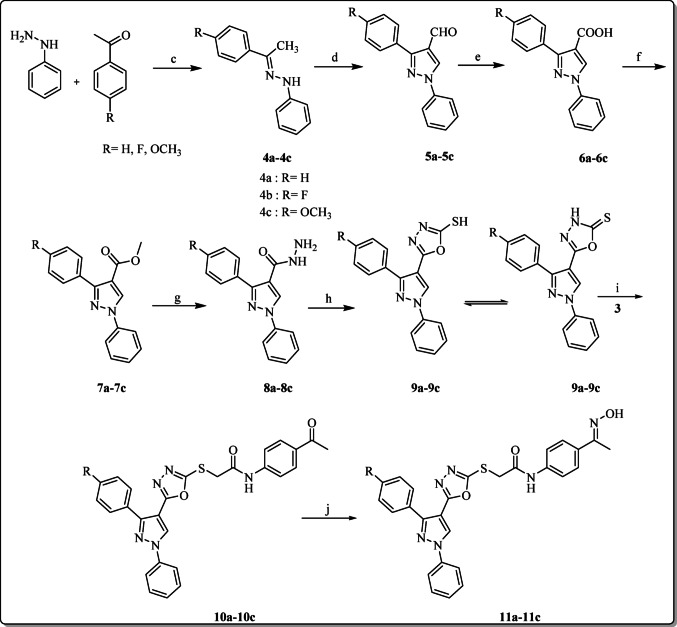
Synthesis of the target pyrazole-1,3,4-oxadiazole hybrids **10a-10c and 11a-11c**

**Scheme 3:**
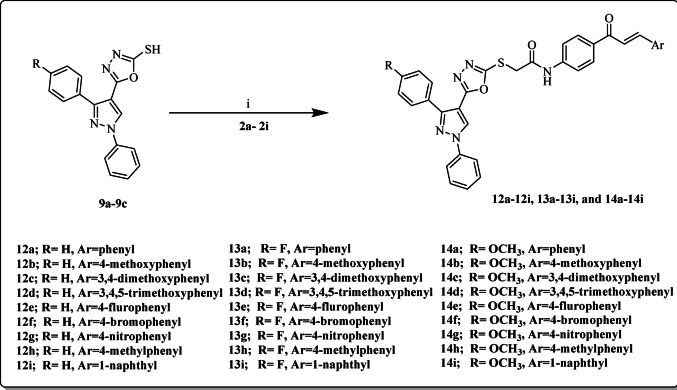
Synthesis of the target pyrazole-oxadiazole-chalcones hybrids **12a-12i**, **13a-13i**, and **14a-14i**

The designed compounds **10a–10c**, **11a-11c**, **12a-12i**, **13a-13i**, and **14a-14i** were prepared as outlined in Schemes 1, 2 and 3. Firstly, chalcones (**1a-1i**) were prepared *via* Claisen-Schmidt condensation of 4-aminoacetophenone and aromatic aldehydes [14], followed by acylation with chloroacetyl chloride to afford acylated chalcones (**2a-2i**) and similarly treating 4-aminoacetophenone to afford acylated 4-aminoacetophenone (**3**) [56, 57] as depicted in Scheme 1.

**Reagents and conditions**: **(a)** EtOH, 60% NaOH, Ice bath, stirring **(b)** DCM, TEA, ClCOCH_2_Cl, stirring 4 h.

Next, hydrazone (**4a-4c**) was prepared by refluxing substituted acetophenone derivatives with phenyl hydrazine in ethanol and drops of acetic acid as a catalyst [58]. Furthermore, pyrazole-4-carbaldehyde (**5a-5c**) was then obtained through Vilsmeier-Haack reaction by reaction of POCl_3_ with DMF, followed by addition of hydrazone (**4a-4c**) and heating [59]. Afterwards, oxidation of compound (**5a-5c**) using KMnO_4_ and KOH in pyridine/water afforded pyrazole carboxylic acid (**6a-6c**) [60], which was then esterified with methanol and sulfuric acid to give ester (**7a-7c**) [61], which upon refluxing with hydrazine hydrate in ethanol produced pyrazole hydrazide (**8a-8c**) [62]. Furthermore, pyrazole hydrazide (**8a-8c**) was subsequently cyclized with CS_2_ and KOH to form pyrazole-1,3,4-oxadiazole hybrids (**9a-8c**). Moreover, the target hybrids **10a**–10**c** were obtained by the reaction of compounds (**9a-9c**) and (**3**) in the presence of triethylamine. Additionally, target hybrids **11a-11c** were obtained by the reaction of compounds (**10a-10c**) with hydroxylammonium hydrochloride in the presence of pyridine as illustrated in Scheme 2.

**Reagents and conditions**: **(c)** AcOH, EtOH, reflux, 2 h; **(d)** DMF, POCl_3_ **(e)** Pyridine, KMnO_4_, stirring 3 h; **(f)** MeOH, H_2_SO_4_, reflux,8 h; **(g)** EtOH, Hydrazine hydrate 85% reflux, 8 h; **(h)** EtOH, NaOH, CS_2_ reflux,6 h; **(i)** CH_3_CN, TEA, stirring 4 h; **(j)** EtOH, NH_2_OH.HCl, pyridine reflux, 4 h.

Finally, the target hybrids **12a-12i**, **13a-13i**, and **14a-14i** were afforded by the reaction of compounds (**9a-9c**) and (**2a-2i**) in the presence of triethylamine as shown in **Scheme 3**. Structures of the synthesized compounds **9a-c**, **10a-10c**, **11a-11c**, **12a-12i**, **13a-13i**, and **14a-14i** were elucidated by FT-IR, ^1^H NMR, ^13^C NMR, and Elemental analysis. ^1^H NMR spectra of all compounds displayed their intended protons in the expected chemical shifts. Similarly, ^13^C NMR spectra of compounds showed the presence of the exact number of carbon atoms at their corresponding ppm. In addition, Elemental analysis showed matching of the calculated values and the found values.

**Reagents and conditions**: **(i)** CH_3_CN, TEA, stirring 4 h.

### Biological evaluation

#### In vitro anticancer activity

**Thirty-three** newly synthesized target compounds were selected by the National Cancer Institute (NCI); potential pyrazole-oxadiazole hybrids **10a-10c**,** 11a-11c**,** 12a-12i**, **13a-13i**, and **14a-14i** for their in vitro anticancer screening against full NCI’s disease-oriented 60 human cell lines. The tested cell lines are derived from nine tumor subpanels, including leukemia, lung, colon, CNS, melanoma, ovarian, renal, prostate, and breast cancer cell lines according to the reported protocol of the Drug Evaluation Branch, NCI, Bethesda (http://www.dtp.nci.nih.gov*).*

#### In vitro single-dose assay on full NCI − 60 cell lines

The selected hybrids **10a-10c**,** 11a-11c**,** 12a-12i**, **13a-13i**, and **14a-14i** were tested at single concentration of 10 µM. Screening results were recorded as growth percent of the cells treated with the tested compound as compared to the untreated reference cells. Mean graph midpoint (MG-MID) (differential activity patterns, bar scale) was constructed for each cell line to facilitate visual scanning of data for potential NCI patterns of selectivity, with bars depicting the deviation of the individual tumor cell lines from the overall mean value of all growth percentages recorded for all cell lines tested. Bars pointed to the left (positive values) denote resistance where the growth is greater than the average, while bars pointed to the right (negative values) denote sensitivity where the growth is less than the average. Delta means the logarithm of the difference between the (MG-MID). The screening results were redisplayed as percentage growth inhibition (GI %), (Supporting information Tables 1–5).

**Fig. 5:**
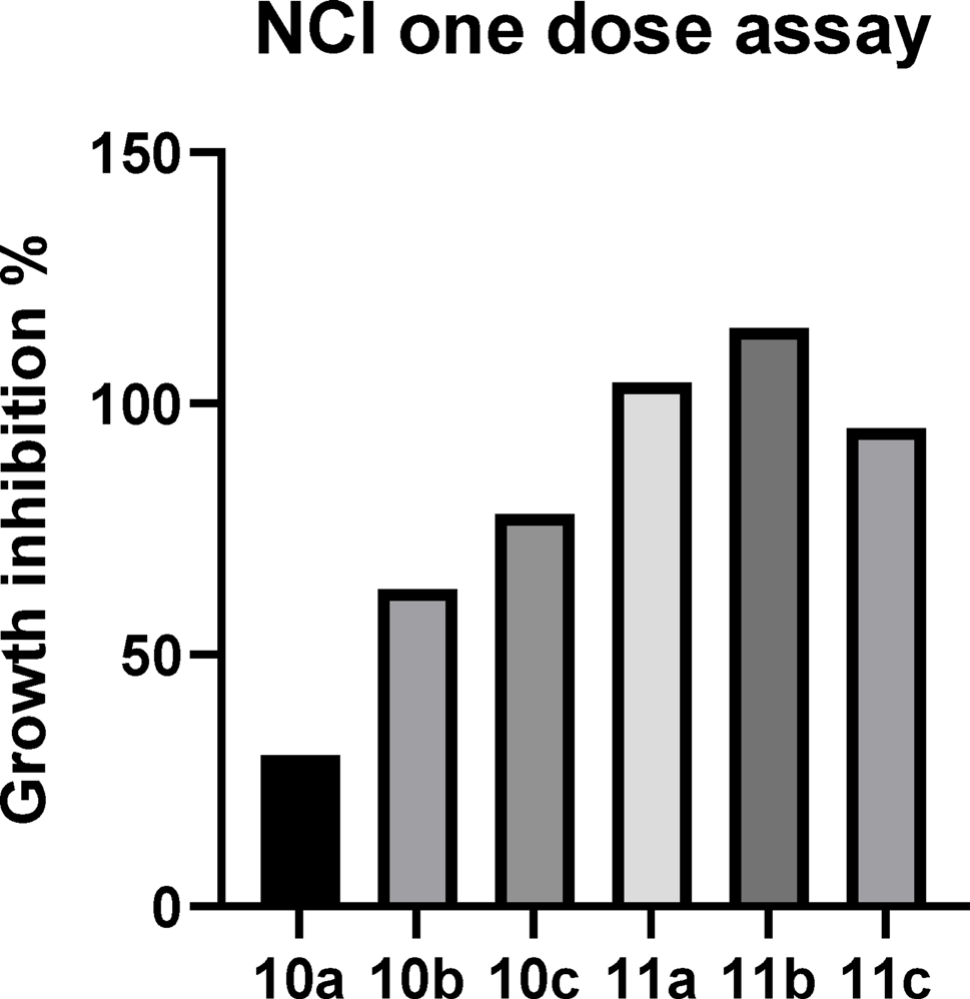
Mean GI % for in vitro tumor cell lines at 10 µM concentration for the most potent hybrids **10a-10c** and **11a-11c**

Pyrazole-oxadiazole hybrids **10a-10c** with 4-acetylphenyl group showed significant cytotoxicity with varied broadness in the spectrum of activity. The results of the ketone derivatives showed that compounds **10b** and **10c** displayed strong anticancer activity (growth inhibition % of 60.78% to 174.47%) against a number of 31, and 33 cancer cell lines, respectively. Moreover, compounds **10b** and **10c** showed mean growth inhibition percentages of 63.11, and 78.02%, respectively, and showed a moderate anticancer activity against the remaining cancer cell lines, as shown in (**Supporting information Table 1**). Furthermore, compound **10a** showed moderate activity against most cancer cell lines except non-small cell lung cancer NCI-H522 cell line, showed significant growth inhibition percent of 122.26% and a mean growth inhibition percent of 30% (Fig. 5).

Changing the acetyl group into oxime in hybrids **11a**,** 11b**, and **11c** resulted in increased activity and broadness with growth inhibition ≥ 60.78% to 197.63% against almost all cancer cell lines, with mean growth inhibition percentage of 104.23%, 115.10% and 95.10%, respectively (Fig. 26). The antiproliferative activity of these oxime derivatives showed that compound **11b** is the most active hybrid displaying very strong antiproliferative activity against almost all cancer cell lines, as shown in (**Supporting information Table 1)**. This activity could be attributed to both oxime group introduction and the presence of fluorine substituent on the pyrazole phenyl ring.

On the other hand, pyrazole-oxadiazole-chalcone hybrids **12a-12i**, **13a-13i**, and **14a-i** resulted in significant decrease in both activity and broadness compared with ketone, and oxime hybrids **10a-10c**, and **11a-11c**. The obtained results of hybrids **12a-12i**, with unsubstituted phenyl ring on C_3_ of pyrazole ring, revealed that most of the tested compounds showed potent inhibition on four cancer cell lines (Leukemia RPMI-8226, Colon Cancer HCT-116, Melanoma LOX IMVI, and Breast Cancer MCF7 cell lines). Compounds **12a** and **12e** were found to be the most active in these derivatives and they displayed a potent anticancer activity (growth inhibition ≥ 61.29% and up to 112.16%) against the four cancer cell lines that mentioned above. compound **12a** exhibited inhibitory activity on leukemia (RPMI-8226; GI% = 94.94%), colon cancer (HCT-116; GI% = 65.39%), breast cancer (MCF7; GI% = 61.29%). compound **12e** exhibited inhibitory to cytotoxic (complete cell death) activity on leukemia (RPMI-8226; GI% = 76.4%), Colon Cancer (HCT-116; GI% = 112.16%), melanoma (LOX IMVI; GI% = 63.42%), breast cancer (MCF7; GI% = 93.56%). Compound **12d** showed significant selectivity against leukemia cell lines, while compound **12f** showed selectivity against all CNS cancer cell lines except SF-268 cell line with GI% ranging from 34.96 up to 84.72, with remarkable cytotoxic activity on renal cancer cell line RXF 393 with GI % of 104.52. And finally compound **12i** showed significant cytotoxic activity on melanoma cancer cell line LOX IMVI with GI % of 169.52. The remaining derivatives showed a moderate to weak anticancer activity against most of the used cancer cell lines (Supporting information Tables 2–3). The investigation of the effect of introducing a 4-fluoro substitution on the pyrazole phenyl ring in the activity of hybrids **13a-13i** showed that the presence of the fluorine atom resulted in almost equal activity with the unsubstituted series with enhanced potency against few cell lines and these results can be attributed for the fact of the fluorine atom is a bio-isostere for the hydrogen and can play crucial role for additional binding with the target enzyme. Like the previous series most of the compounds showed potent inhibition on the same four cancer cell lines (RPMI-8226, HCT-116, LOX IMVI, and MCF7). Among this group of compounds, derivatives **13a** and **13 g** demonstrated the highest level of activity. compound **13a** inhibited the growth of cancer cells by 70.03% and 72.66% against RPMI-8226 and HCT-116 respectively and compound **13 g** inhibited the growth of cancer cells by 63.66% against RPMI-8226, 70.11% against HCT-116, and 65.36% against MCF7. Compound **12d** showed significant selectivity against leukemia cell lines, while compound **12f** displayed remarkable inhibition of the Leukemia cell line RPMI-8226, with a growth inhibition percentage (GI %) of 80.11. Additionally, compound **13i** exhibited a significant cytotoxic activity against the melanoma cancer cell line LOX IMVI, with a GI % of 107.63. The remaining derivatives displayed moderate to weak anticancer activity against most of the tested cancer cell lines (Supporting information Tables 2–3).

Substitution of the pyrazole phenyl with 4-methoxy group generally resulted in decreased activity against most cell lines and even greater reduction in the activity where the chalcone contained an electron donating group. Among this series few compounds showed noteworthy activity as compound **14a** which inhibited the growth of cancer cells by 57.03% and 71.48% against RPMI-8226 and HCT-116, respectively, while compounds **14e** and **14f** displayed remarkable inhibition of the Leukemia cell line RPMI-8226, with a growth inhibition percentage (GI %) of 80.37 and 75.09, respectively. The remaining derivatives displayed moderate to weak anticancer activity against most of the tested cancer cell lines (Table 4 and Supporting information Table 5).

#### In vitro five-dose assay on full NCI − 60 cell panel

The pyrazole-oxadiazole hybrids **10b** and **11a-11c** were further selected by NCI upon their 1st screening results for advanced NCI full panel five dose assays at 10-fold dilutions of five different concentrations (0.01, 0.1, 1, 10 & 100 µM). The resulting data of the evaluated compounds was displayed with calculated three response parameters for each cell line **GI**_**50**_ (growth inhibitory activity) value: compound concentration that causes 50% decrease in the net growth of cells, **TGI** (cytostatic activity) value: compound concentration that results in total growth inhibitions, and **LC**_**50**_ (cytotoxic activity) value: compound concentration that causes net 50% loss of initial cells at the end of the incubation period of 48 h as illustrated in Supporting information Tables 6–9. Moreover, mean graph midpoint (**MG-MID**) values have been calculated giving an averaged activity parameter over subpanel and full panel cell lines for each tested compound [**MID**_**a**_: the average sensitivity of all cell lines for the tested compound while **MID**_**b**_: the average sensitivity of cell lines of a specific subpanel for the tested compound].

**Fig. 6:**
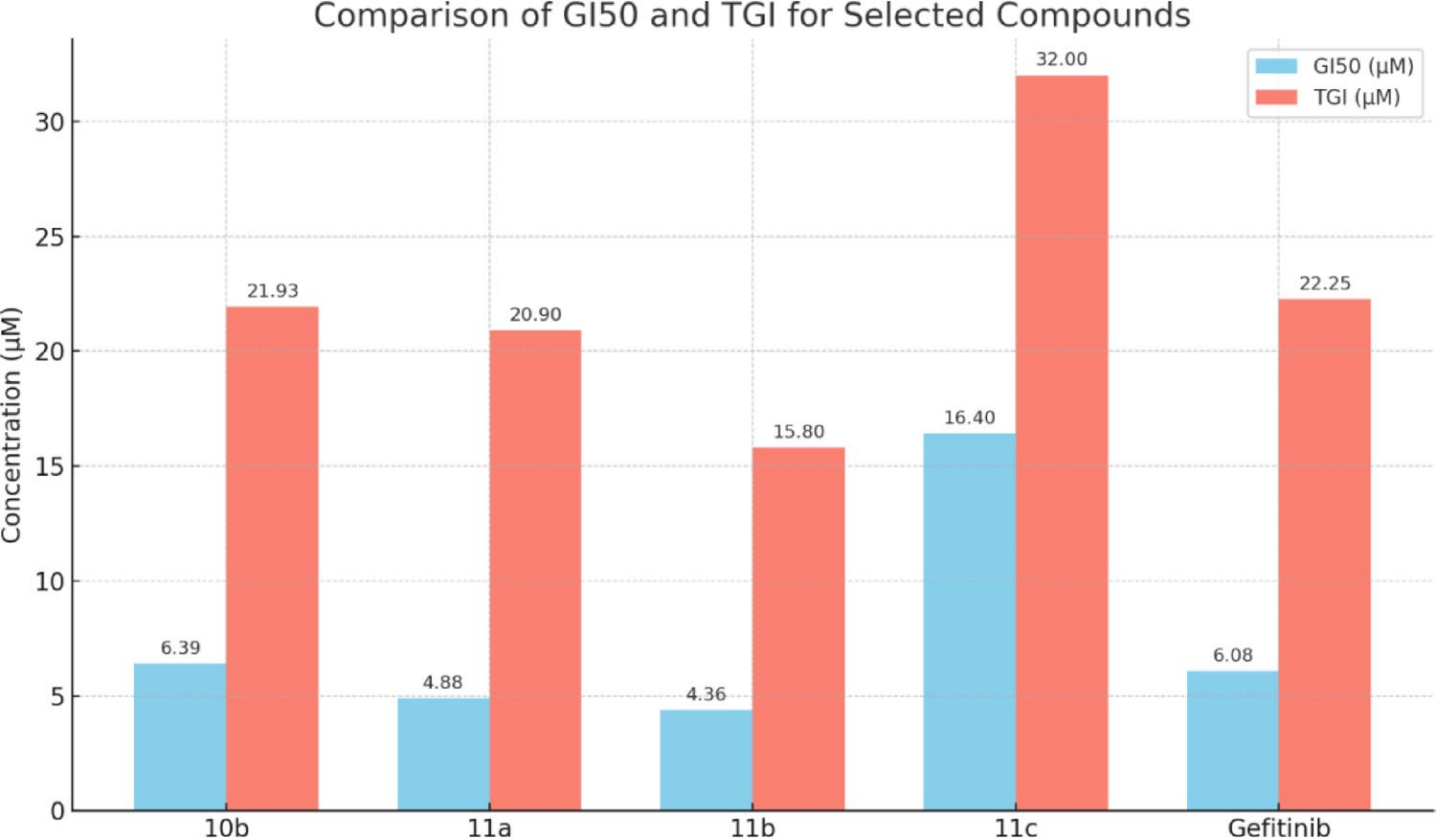
Mean (MID_a_) GI_50_ and TGI of compounds **10a**, and **11a-11c** compared with Gefitinib

As displayed in Supporting information Table 6, The ketone hybrid **10b**, exhibited very strong antiproliferative activity with GI_50_ ≤ 10 µM against 49 cancer cell lines (GI_50_ range from 1.71 to 9.52 µM) with outstanding activity against 26 cancer cell lines with GI_50_ ≤ 5 µM and remarkable activity against 23 cancer cell lines with GI_50_ ranging between 5.11 and 9.52 µM. Furthermore, hybrid **10b** showed strong activity against 11 cancer line with GI_50_ range from 10.4 to 15.8 µM. Additionally, hybrid **10b** showed an average GI_50_ (MID_a_) of 6.39 µM, and TGI of 21.93 µM which is comparable with the reference drug gefitinib average GI_50_ of 6.08 µM, and TGI of 22.25 µM. Moreover, hybrid **10b** showed more potent LC_50_ value of 47.55 µM compared with gefitinib LC_50_ value of 52.59 µM indicating potent cytotoxic activity (Fig. 6).

Regarding the sensitivity of various cell lines for hybrid **10b**, it showed distinct pattern of homogenous anticancer activity on the individual cell lines. It exhibited remarkable growth inhibition GI_50_ on entire panel of tumor cell lines with average sensitivity values (MID_b_) ranged from 3.35 to 8.91 µM. The most sensitive subpanel for **10b** was breast cancer with GI_50_ (MID_b_) = 3.35 µM, (Supporting information Table 7). For selectivity of **10b** at GI_50_ level for anti-proliferative activity and according to selectivity index (SI), hybrid **10b** showed non-selective anti-proliferative activity as the anti-proliferative activity selectivity index ranged from 0.83 to 1.91, and its selectivity at TGI level for cytostatic activity, it has non-selective potency with selectivity index ranged from 0.65 to 1.30 (Supporting information Table 7).

Once again, as in one dose assay converting ketone function group to its corresponding oxime functionality increase both activity and broadness. Hybrids **11a**, and **11b** with oxime function group, were the most active compounds among all of the synthesized derivatives. Compound **11a** exhibited very strong anti-proliferative activity with GI_50_ ≤ 10 µM against 56 cancer cell lines (GI_50_ range from 0.31 to 10.0 µM) with outstanding activity against 33 cancer cell lines with GI_50_ ≤ 5 µM and remarkable activity against 23 cancer cell lines with GI_50_ ranging between 5.15 and 10.0 µM. (Supporting information Table 8). Moreover, **11a** showed strong antitumor activity against 3 cancer cell lines with GI_50_ ≤ 20 µM (GI_50_ range from 11.9 to 17.0 µM). Regarding the sensitivity of various cell lines for hybrid **11a**, it showed distinct pattern of homogenous anticancer activity on the individual cell lines. It exhibited remarkable growth inhibition GI_50_ on entire panel of tumor cell lines with average sensitivity (MID_a_) of 4.88 µM which is more potent than the reference drug gefitinib (GI_50_ = 6.08 µM) as illustrated in Fig. 6, and on subpanels of tumor cell with average sensitivity values (MID_b_) ranged from 1.45 to 6.99 µM. The most sensitive subpanel for **11a** was leukemia with GI_50_ (MID_b_) = 1.45 µM, (Supporting information Table 9). For selectivity of **11a** at GI_50_ level for anti-proliferative activity and according to selectivity index, hybrid **11a** showed moderate selective anti-proliferative activity toward leukemia cell lines with selectivity index of 3.37, and the remaining subpanels selectivity index ranged from 0.70 to 1.29. compound **11a** selectivity at TGI level for cytostatic activity, it showed moderate selective potency toward leukemia subpanel with selectivity index of 5.39, and the remaining subpanels selectivity index ranged from 0.60 to 1.36 (Supporting information Table 9).

Moreover, hybrid **11b**, exhibited very strong antiproliferative activity against 57 cancer cell lines with GI_50_ ≤ 10 µM (GI_50_ range from 0.21 to 9.68 µM) with outstanding activity against 44 cancer cell lines with GI_50_ ≤ 5 µM and remarkable activity against 13 cancer cell lines with GI_50_ ranging between 5.24 and 9.68 µM. Moreover, **11a** showed strong antiproliferative activity against 2 cancer cell lines (Non-Small Cell Lung Cancer NCI-H322M, and Ovarian cancer OVCAR-5 cell lines) with GI_50_ of 13.4 and 15.9 µM respectively (Supporting information Table 8). Regarding the sensitivity of various cell lines for hybrid **11b**, it exhibited remarkable growth inhibition GI_50_ on entire panel of tumor cell lines with average sensitivity (MID_a_) of 4.36 µM as illustrated in Fig. 6. Moreover, on subpanels of tumor cell it showed average sensitivity values (MID_b_) ranged from 1.40 to 6.00 µM. The most sensitive subpanel for **11b** were leukemia with GI_50_ (MID_b_) = 1.40 µM. Hybrid **11b** showed moderate selectivity toward leukemia subpanel with selectivity index of 3.11, while the remaining subpanels selectivity index ranged from 0.73 to 1.35, and its selectivity at TGI level for cytostatic activity, exhibited moderate selectivity towards leukemia subpanel with selectivity index of 4.88 over the other subpanels with SI ranged from 0.71 to 1.33 (Supporting information Table 9). Notably, hybrids **11a** and **11b** showed potent cytotoxic activity towards the major of leukemia cells with LC_50_ values ranged between 0.89 and 34.3 µM.

Finally, hybrid **11c**, exhibited very strong anticancer activity with GI_50_ ≤ 10 µM against 30 cancer cell lines (GI_50_ range from 0.88 to 9.58 µM) and strong anticancer activity against 10 cancer line with GI_50_ ≤ 20 µM (GI_50_ range from 11.7 to 18.1 µM). Moreover, hybrid **11**c displayed moderate to weak activity against the remaining cancer cell lines with (GI_50_ values ranging from 20.7 to 97.9 µM) as presented in Supporting information Table 8. Ongoing through the details of the antiproliferative assay results hybrid **11c** displayed outstanding activity against 16 cancer cell lines with GI_50_ ≤ 5 µM and remarkable activity against 14 cancer cell lines with GI_50_ ranging between 5.01 and 9.58 µM. Regarding the sensitivity of various cell lines for hybrid **11c**, it showed moderate growth inhibition GI_50_ on entire panel of tumor cell lines with average sensitivity (MID_a_) of 16.4 µM (Fig. 6). Furthermore, it exhibited remarkable growth inhibition GI_50_ on leukemia subpanel with average sensitivity (MID_b_) of 2.77 µM, while showing strong to moderate sensitivity on the remaining subpanels of tumor cell with average sensitivity values (MID_b_) ranged from 6.00 to 28.77 µM (Supporting information Table 9). For selectivity of **11c** at GI_50_ level for anti-proliferative activity and according to selectivity index, hybrid **11c** showed moderate-selective anti-proliferative activity toward leukemia subpanel with SI of 5.92, over the other subpanels with SI ranged from 0.57 to 2.73, while its selectivity at TGI level for cytostatic activity, it has highly-selective potency toward leukemia cell lines with SI of 8.79 over the other subpanels with SI ranged from 0.55 to 1.32. Summing up, the results of the five-dose investigation for the oxime hybrids **11a**, **11b** and **11c**, indicating a positive impact of the oxime moiety on the activity.

Comparing the resulting antitumor activity of the most potent hybrids **11a**, and **11b** with the reference drug gefitinib, it is obvious that hybrids **11a**, and **11c** for the unsubstituted and the fluorine substituted pyrazole-C_3_ phenyl ring, exhibited average anti-proliferative activity (GI_50_) more potent than gefitinib with GI_50_ (MID_a_) of 4.88 and 4.36 µM, respectively compared with gefitinib GI_50_ (MID_a_) of 6.08 µM and showed anti-proliferative activity (GI_50_) more potent than gefitinib on 39, and 44 cancer cell lines, respectively, and the both hybrids were shared in displaying highly potent antitumor activity against leukemia (SR) cell line, Moreover, hybrids **11a** and **11b** showed cytostatic activity (TGI) more potent than gefitinib on 38, and 43 cancer cell lines, respectively, with (MID_a_) for cytostatic activity on all cancer cell lines (TGI of 20.90, 15.8, and 22.25 µM for **11a**,** 11b**, and gefitinib, respectively). Additionally, both hybrids **11a** and **11b** have potent cytotoxicity (LC_50_) more than gefitinib on 29 cancer cell lines, and LC_50_: (MID_a_) on all cancer cell lines for hybrid **11a** and **11b** (57.70 and 42.29 µM, respectively) in comparison with gefitinib (LC_50_; MID_a_ = 52.59 µM). Furthermore, hybrid **11b** exhibited sub-micromolar cytotoxicity (cytotoxic effects) on leukemia (SR) cell line with (LC_50_ = 0.89 µM) more potent than gefitinib (LC_50_ = 18.58 µM) (Supporting information Tables 8 and 9).

#### In-vitro cytotoxic effects against normal cells WI-38

The cytotoxicity of hybrid **11b** was evaluated on normal human WI-38 cells, which are derived from fibroblasts obtained from the lung tissue of a three-month pregnant aborted female embryo. The results indicated that compound **11b** exhibited low cell toxicity, with an IC_50_ of 145.5 µM, in contrast to the control drug gefitinib, which had an IC_50_ of 43.2 µM. This suggests that hybrid **11b** is comparatively safer than gefitinib against WI-38 normal human cell lines (Table 1).

**Table 1:** Cytotoxic IC_50_ of compounds **11b**, and gefitinib on human normal cells WI-38 (*n* = 3)

Compound	*Cytotoxicity IC50 / µM*
	WI-38
11b	145.5 ± 4.7
Gefitinib	43.2 ± 1.8

### Enzymatic inhibitory assay

#### Assay of EGFR and VEGFR-2

The most active newly synthesized hybrids (**10b**, **11a**, and **11b**) were selected for further evaluation of their inhibitory potential against both EGFR and VEGFR-2 enzymes (Table 2). These compounds demonstrated promising activity compared to the reference drugs, Gefitinib (for EGFR) and Sorafenib (for VEGFR-2), suggesting their potential as dual EGFR/VEGFR-2 inhibitors. Compound **11b** exhibited the most potent inhibitory activity against both targets, with IC_50_ values of 26.38 µM against EGFR and 114.17 µM against VEGFR-2. This compound showed superior EGFR inhibition compared to Gefitinib (IC_50_ = 39.56 µM) and also a comparable VEGFR-2 IC_50_ than Sorafenib (IC_50_ = 108.74 µM), indicating a favorable profile. While compounds **10b** (IC_50_ = 51.63 µM for EGFR and 137.76 µM for VEGFR-2) and **11a** (IC_50_ = 32.47 µM for EGFR and 146.09 µM for VEGFR-2) also showed inhibitory activity against both EGFR and VEGFR-2, their potency was less pronounced than that of **11b**. These results suggest that compound **11b**, in particular, warrants further investigation as a potential dual EGFR/VEGFR-2 inhibitor.

**Table 2:** In vitro EGFR, and VEGFR-2 inhibitory activity (IC_50_ nM) of the most potent compounds **10b**, **11a**, and **11b** (*n* = 3)

Compounds	EGFR	VEGFR-2
**10b**	50.97 ± 2.05	136.82 ± 6.92
**11a**	31.46 ± 1.30	149.03 ± 3.55
**11b**	26.94 ± 0.33	114.10 ± 2.12
Gefitinib	39.57 ± 3.52	–
Sorafenib	–	110.86 ± 5.06

#### Cell cycle analysis

**Fig. 7:**
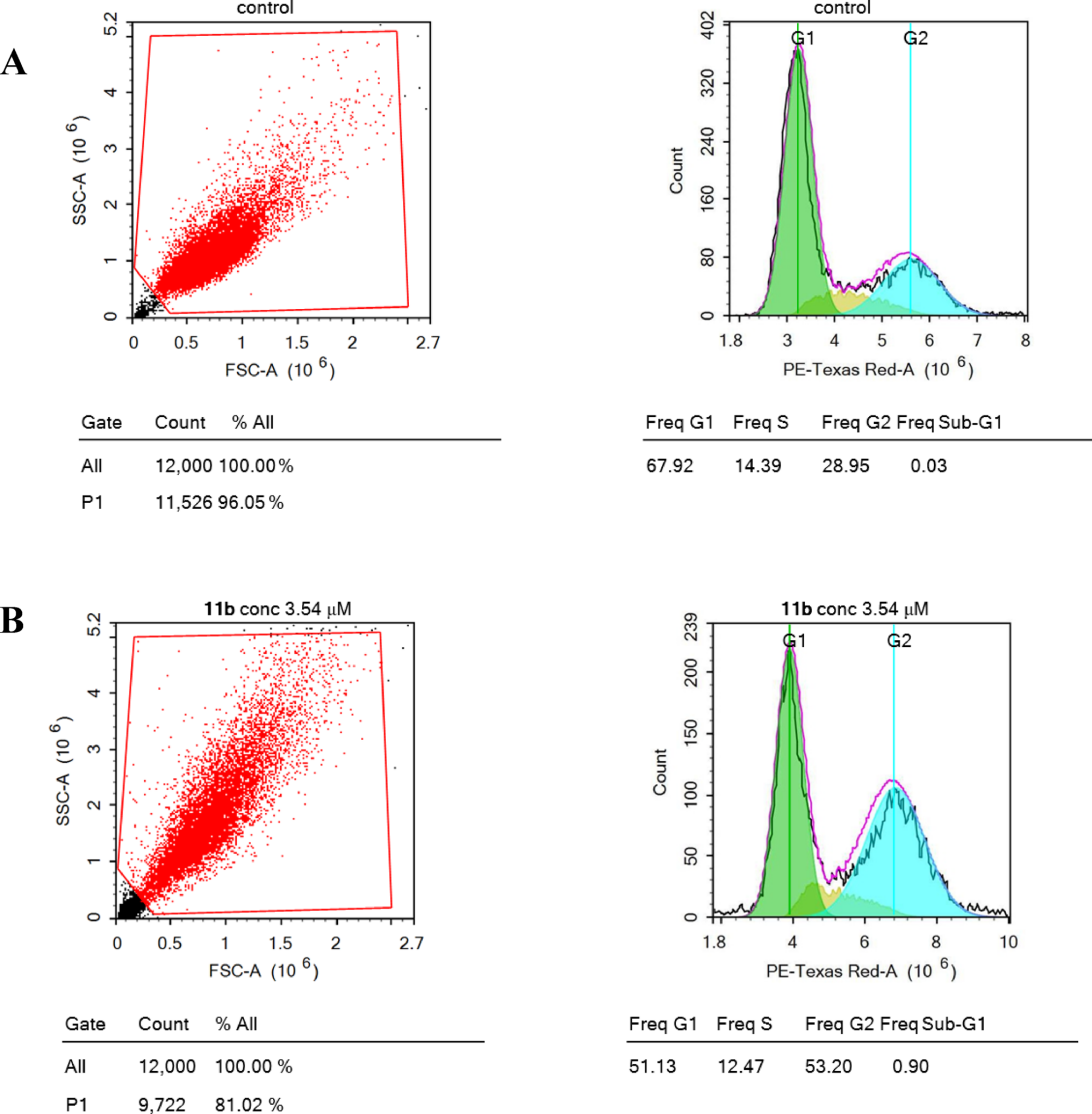
Cell cycle analysis of control (**a**) and after treatment with hybrid **11b** (**b**)

CDKs inhibition is strongly associated with the induction of cell cycle arrest at the G2/M phase [63]. Therefore, to gain more insights into the potent cytotoxic activity of hybrid **11b**, cell cycle analysis of MCF7 cells was performed *via* flow cytometry assay (Fig. 7), which clearly showed that hybrid **11b** caused a significant G2/M phase arrest. After treating MCF7 cells with IC_50_ of hybrid **11b** (3.54 µM) for 48 h, there was an increase in the number of cells in the G2/M phase (53.20%) compared to the control (28.95%). These changes were concomitant with a noteworthy decrease in the G0/G1 (51.13%) compared to the control (67.92%), similarly S phases decreased (12.47%) compared with the control (14.39%), indicating that these S phase cells can’t progress to the next cell cycle stage.

#### Apoptosis assay

**Fig. 8:**
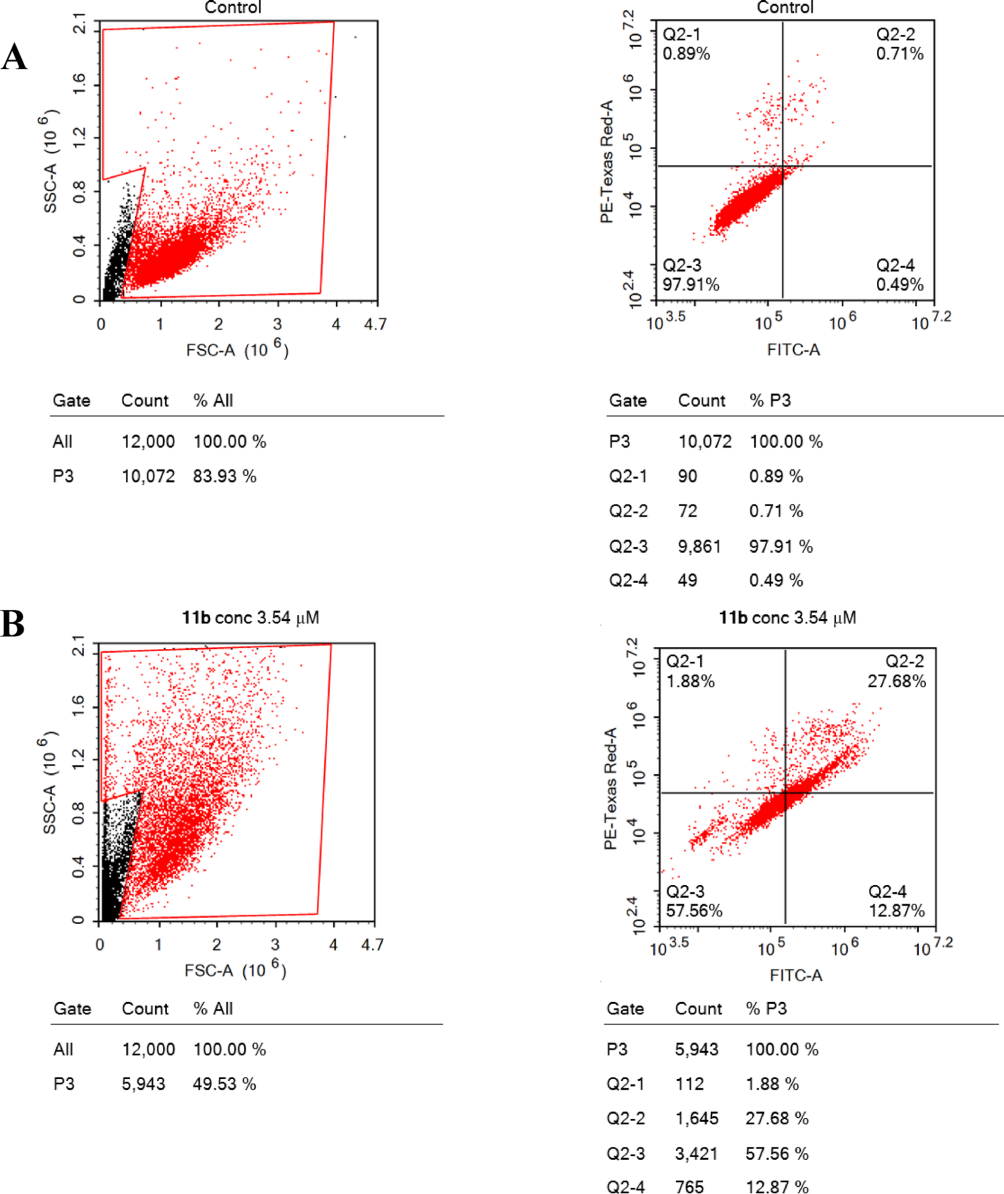
Percentage of apoptosis and necrosis of control cells (**a**), treated with hybrid **11b** (**b**)

To understand the mechanism of cell death, whether apoptosis or necrosis, induced by hybrid **11b**. MCF7 cells were assessed using Annexin-V/FITC staining coupled with flow cytometry (Fig. 8). Hybrid **11b** induced late apoptosis (programmed cell death) in MCF7 with an apoptosis percentage of 27.68% compared to untreated control cells (0.71%), suggesting hybrid **11b** as an apoptotic inducer. In addition, necrotic cell death (non-programmed) (12.86%) was also observed after treatment with hybrid **11b**, but less than apoptosis, indicating that apoptosis is the predominant cell death mechanism of hybrid **11b**. Regarding cell cycle arrest and apoptosis findings, hybrid **11b** exhibited excellent anticancer activity causing programmed cell death apoptosis induction and cell cycle arrest at the G2/M phase.

#### Evaluation of nitric oxide release

**Fig. 9:**
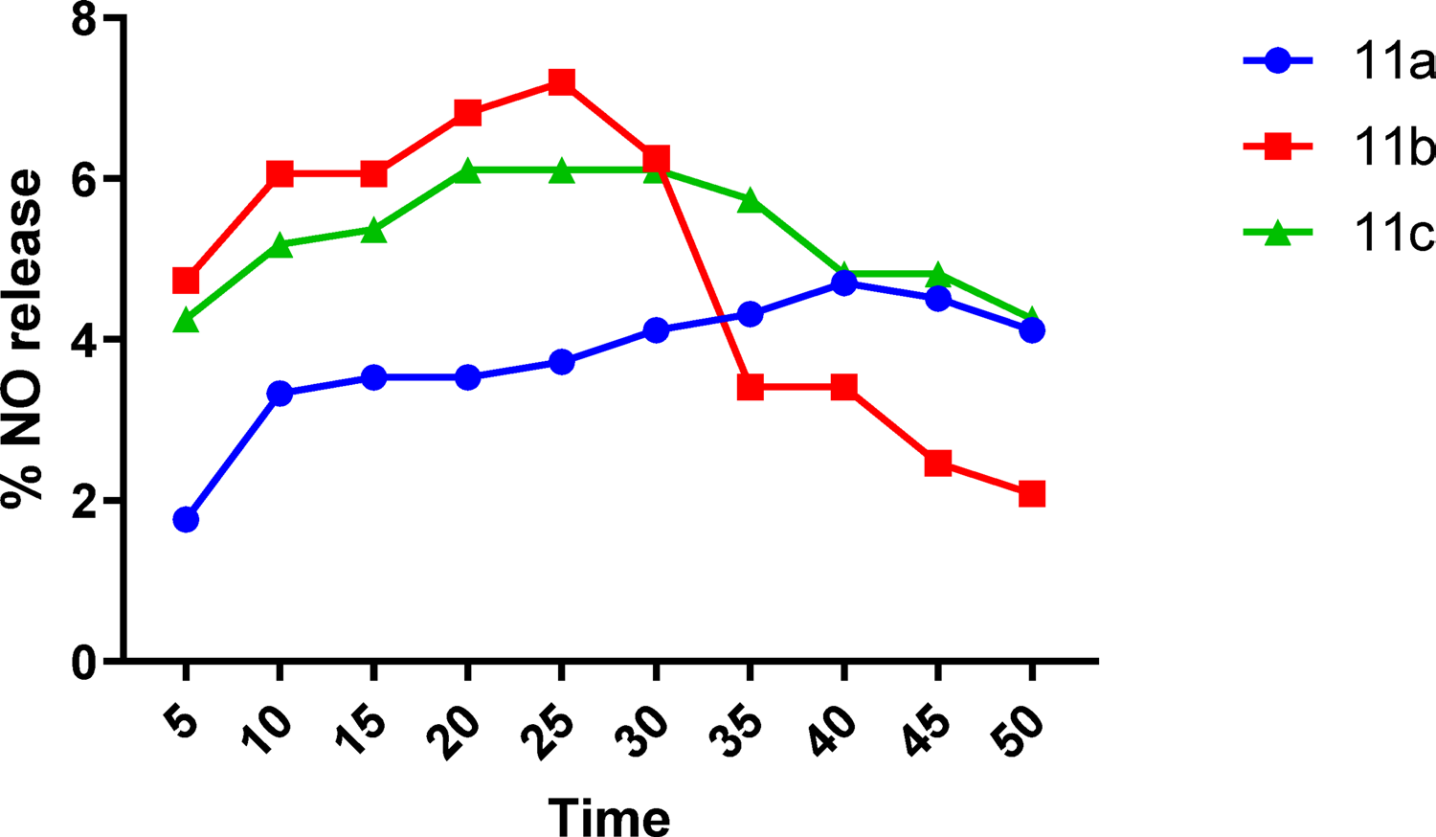
Percentage of NO release of compounds (**11a-11c**)

Due to the significant biological importance of nitric oxide (NO), quantifying NO released is an important criterion in nitric oxide donor research [1]. The synthesized compounds **11a-11c** are considered NO donors, as they can release NO under certain physiological conditions. The spontaneous oxidation of NO under physiological conditions into nitrites was the basis for a colorimetric method for measuring NO in biological systems using the Griess diazotization reaction. The Griess reaction method is a simple and sensitive technique that was used to measure the nitric oxide release from the synthesized compounds **11a-11c**. It was carried out in phosphate buffer, pH 7.4, in the presence of *L*-cysteine, using sodium nitrite solution (1–50 nmol/mL) as a standard to establish the standard sodium nitrite curve. The role of *L*-cysteine is to accelerate the NO release from the organic nitrate [64, 65]. The formed intense purple color was measured at ƛ-max 540 nm after 5 min intervals. The percentage of NO release is calculated from the following equation:

Results revealed that all the tested compounds (**11a-11c**) released NO with relatively similar %, and compound **11b** exhibited the highest % of release (7.197%) (Table 3). Correlation between time and the % of NO released indicated a gradual increase in the % of NO till reach plateau (after about 25–35 min) followed by decreasing in it (Fig. 9).

**Table 3:** Percentage of NO release of compounds (**11a-11c**)

Comp.	Percentage of NO release									
	5	10	15	20	25	30	35	40	45	50
**11a**	1.765	3.333	3.529	3.529	3.725	4.118	4.314	4.706	4.510	4.118
**11b**	4.735	6.061	6.061	6.818	7.197	6.250	3.409	3.409	2.462	2.083
**11c**	4.259	5.185	5.370	6.111	6.111	6.111	5.741	4.815	4.815	4.259

#### Docking study

Auto Dock vina was used as molecular-docking tool in order to carry out the docking simulations. The conformations with the most favorable (least) free binding energy were selected for analyzing the interactions between the target receptor and ligands by Discovery Studio Visualizer and PyMOL. The ligands are represented in different color; H-bonds and the interacting residues are represented in ball and stick model representation. Reference drugs dinaciclib, gefitinib, hybrids **10b**,** 11a**, and **11b**, was built using ChemBioDraw Ultra 12.0, and finally the crystal structures of VEGFR-2 (PDB ID: 2OH4) and EGFR (PDB ID: 1M17) enzymes were downloaded from protein data bank.

#### Molecular Docking of VEGFR-2

**Fig. 10:**
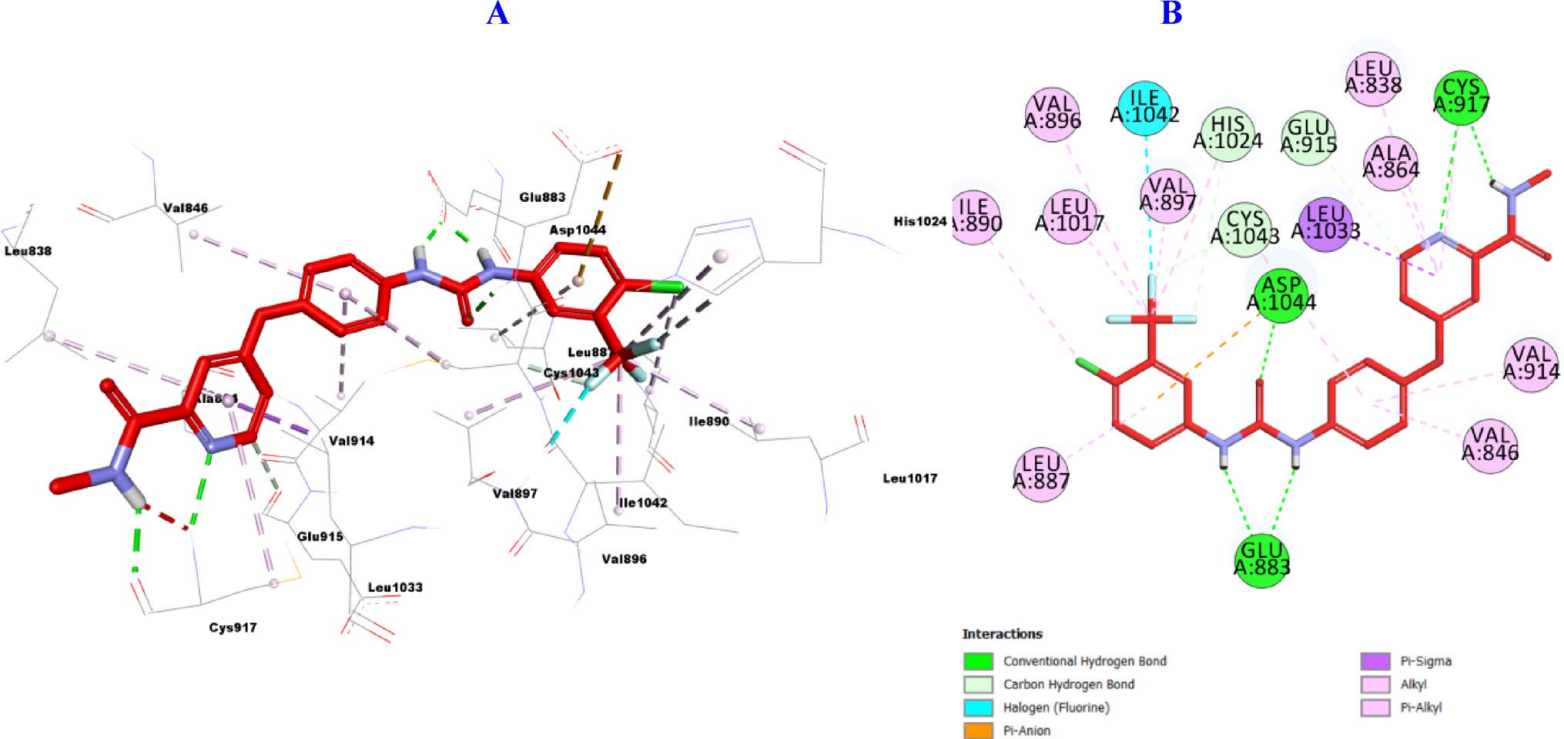
3D (**A**)/2D (**B**) Binding modes/interactions of sorafenib into the active site of VEGFR-2 (PDB ID: 2OH4)

Analysis of the docking results revealed that the docking studies were consistent with the VEGFR-2 assay. From the inspection of the docking results, the reference drug sorafenib against VEGFR-2 (PDB ID: 2OH4), as presented in (Fig. 10**)** was incorporated in the formation of five hydrogen bonds with Glu883 (2), Cys917 (2), and Asp1044 and three carbon hydrogen bonds with Glu915, His1024, and Cys1043. Moreover, it formed one halogen bond with Ile1042, also, it was engaged in the formation of many hydrophobic interactions such as: Pi Sigma with Leu1033 as well as one Pi-Anion interaction with Asp1044 and ten Pi alkyl interactions with Leu838, Val846, Ala864, Leu887, Ile890, Val896, Val897, Val914, Leu1017, and His1024.

**Fig. 11:**
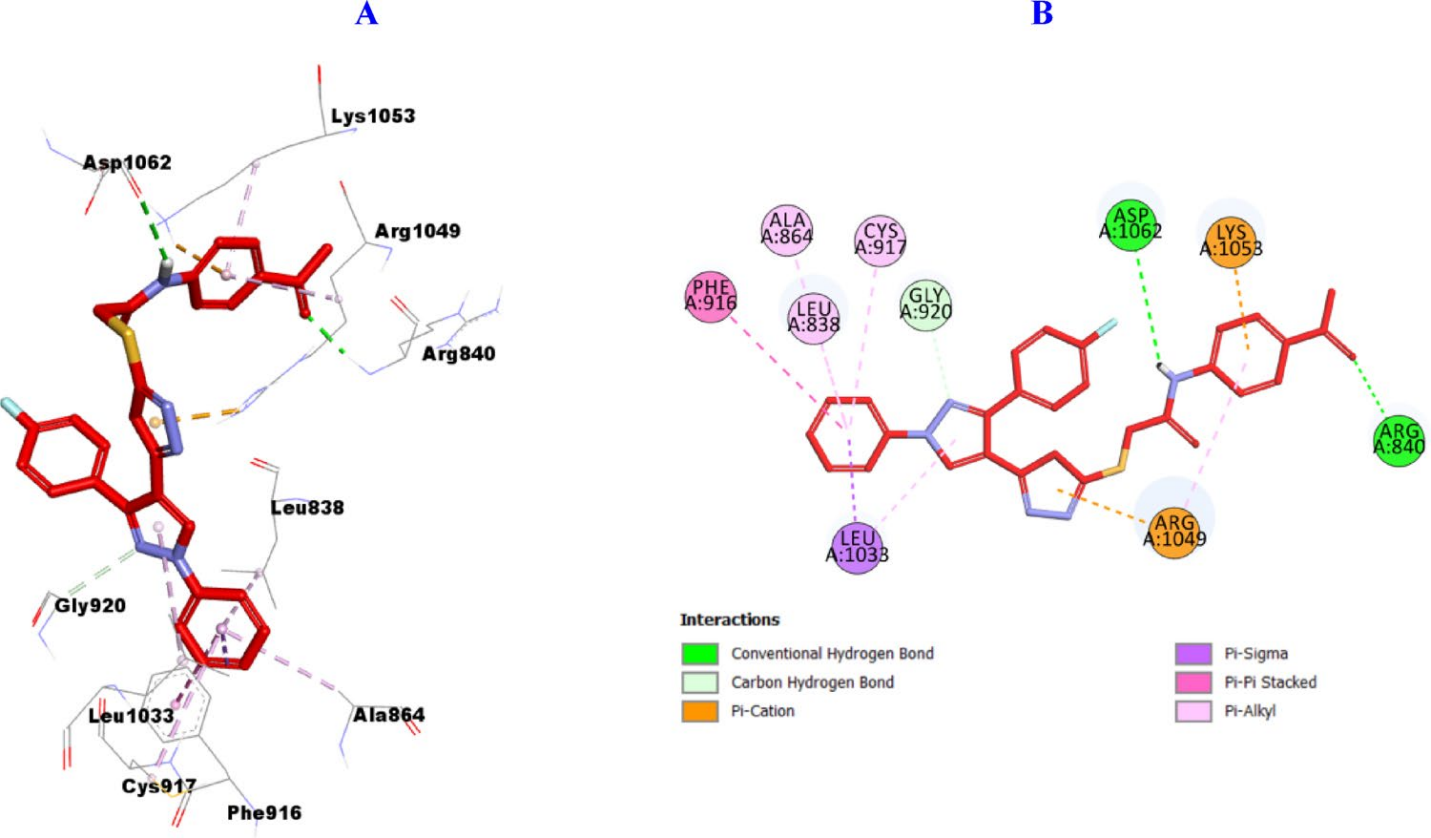
3D (**A**)/2D (**B**) Binding modes/interactions of **10b** into the active site of VEGFR-2 (PDB ID: 2OH4)

Furthermore, it was found that hybrid **10b** against VEGFR-2 (PDB ID: 2OH4), as presented in (Fig. 11**)** was incorporated in the formation of two hydrogen bonds with Arg840 and Asp1062, one carbon hydrogen bond with Gly920, also, it was engaged in the formation of many hydrophobic interactions such as: three Pi-Cation with Arg1049 (2), and Lys1053, as well as one Pi-Sigma interactions with Leu1033 and one Pi-Pi stacked with Phe916 and five Pi-alkyl with Leu838, Ala864, Cys917, Leu1033 and Arg1049.

**Fig. 12:**
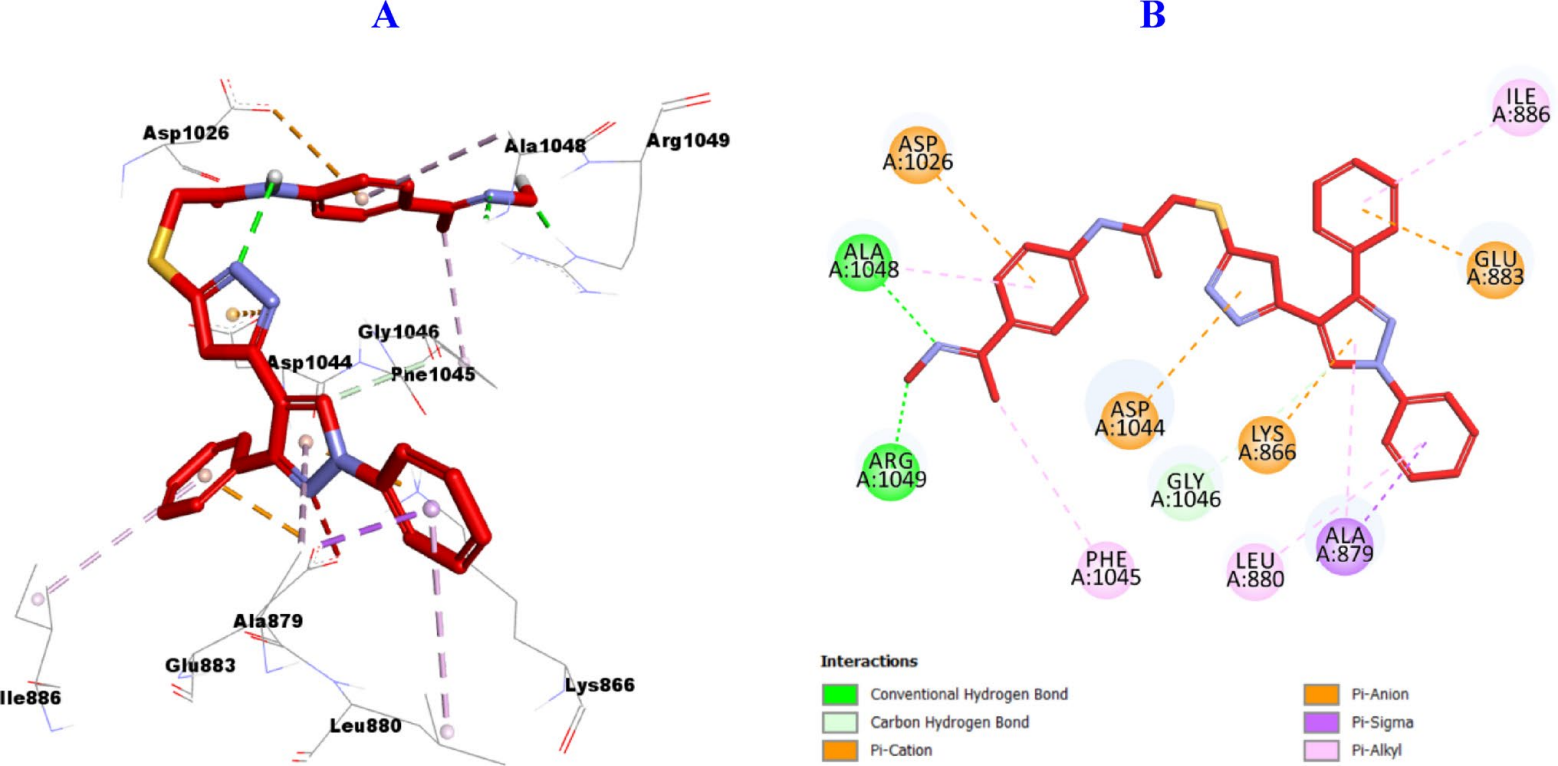
3D (**A**)/2D (**B**) Binding modes/interactions of hybrid **11a** into the active site of VEGFR-2 (PDB ID: 2OH4)

Furthermore, it was found that hybrid **11a** with oxime function group resulted in enhanced binding to the active site of VEGFR-2 (PDB ID: 2OH4), as presented in (Fig. 12**)** it was incorporated in the formation of two hydrogen bonds with Ala1048, and Arg1049, one carbon hydrogen bond with Gly1046 amino acid residue. Also, it was engaged in the formation of many hydrophobic interactions such as: four Pi-Cation with Lys866, Glu883, Asp1026, and Asp1044, and one Pi-Sigma interaction with Ala879 as well as five Pi-alkyl with Ala879, Leu880, Ile886, Phe1045, and Ala1048 amino acid residues.

**Fig. 13:**
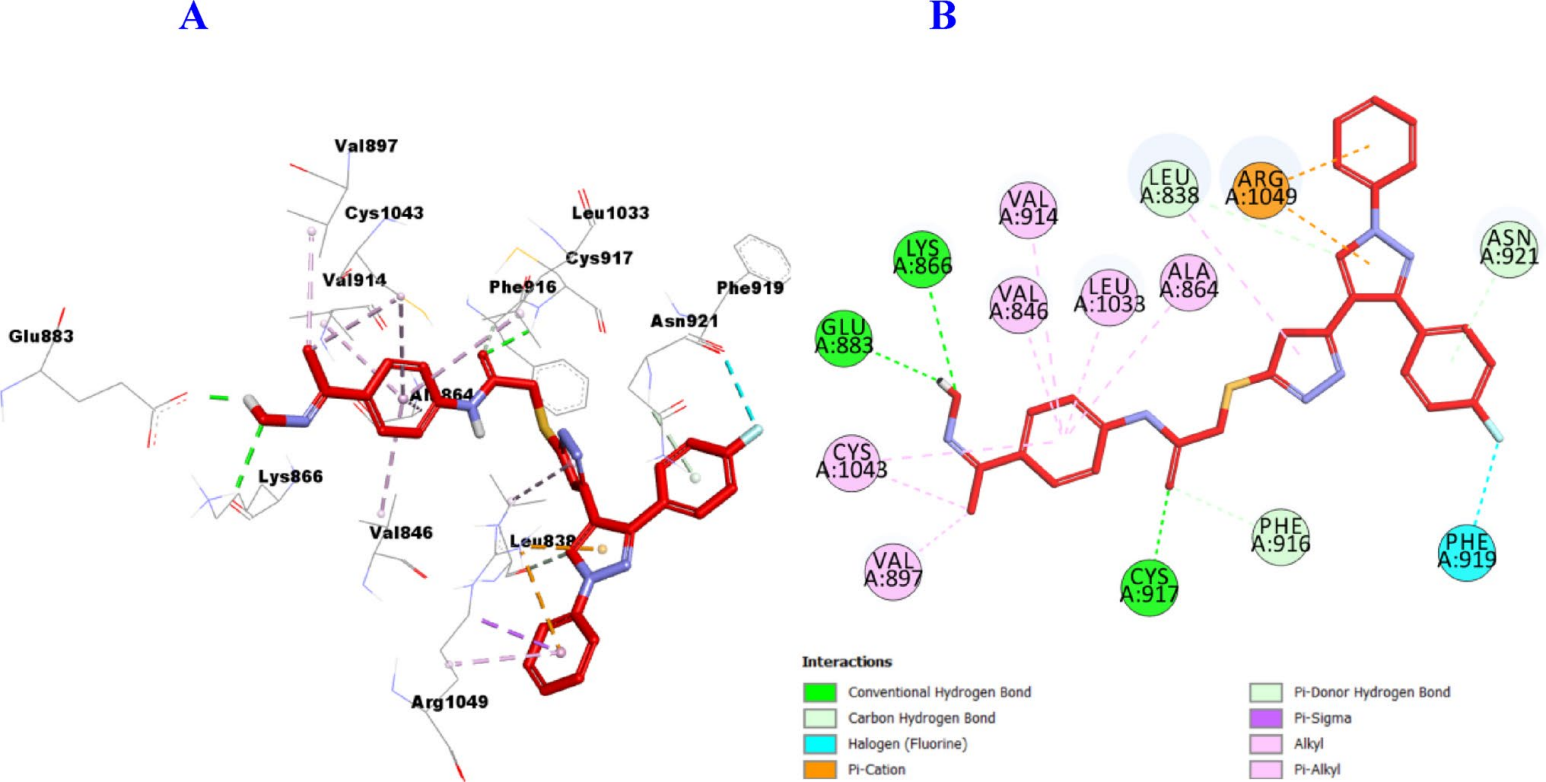
3D (**A**)/2D (**B**) Binding modes/interactions of hybrid **11b** into the active site of VEGFR-2 (PDB ID: 2OH4)

Similarly, it is clear that hybrid **11b** can fit perfectly into the catalytic binding pocket of VEGFR-2 (PDB ID: 2OH4), demonstrating good uniformity between the in vitro VEGFR-2 screening and the in-silico prediction, forming three hydrogen bonds with Lys866, Glu883, and Cys917 amino acid residues, as well as three carbon hydrogen bonds with Leu838, Phe916, and Asn921, and one halogen bond with Phe919. Also, it was engaged in the formation of many hydrophobic interactions such as: two Pi-Cation interations with Arg1049 (2), eight Pi-alkyl with Leu838, Val846, Ala864, Val897, Val914, Leu1033, and Cys1043 (2) amino acid residues (Fig. 13**)**.

#### Molecular Docking of EGFR

**Fig. 14:**
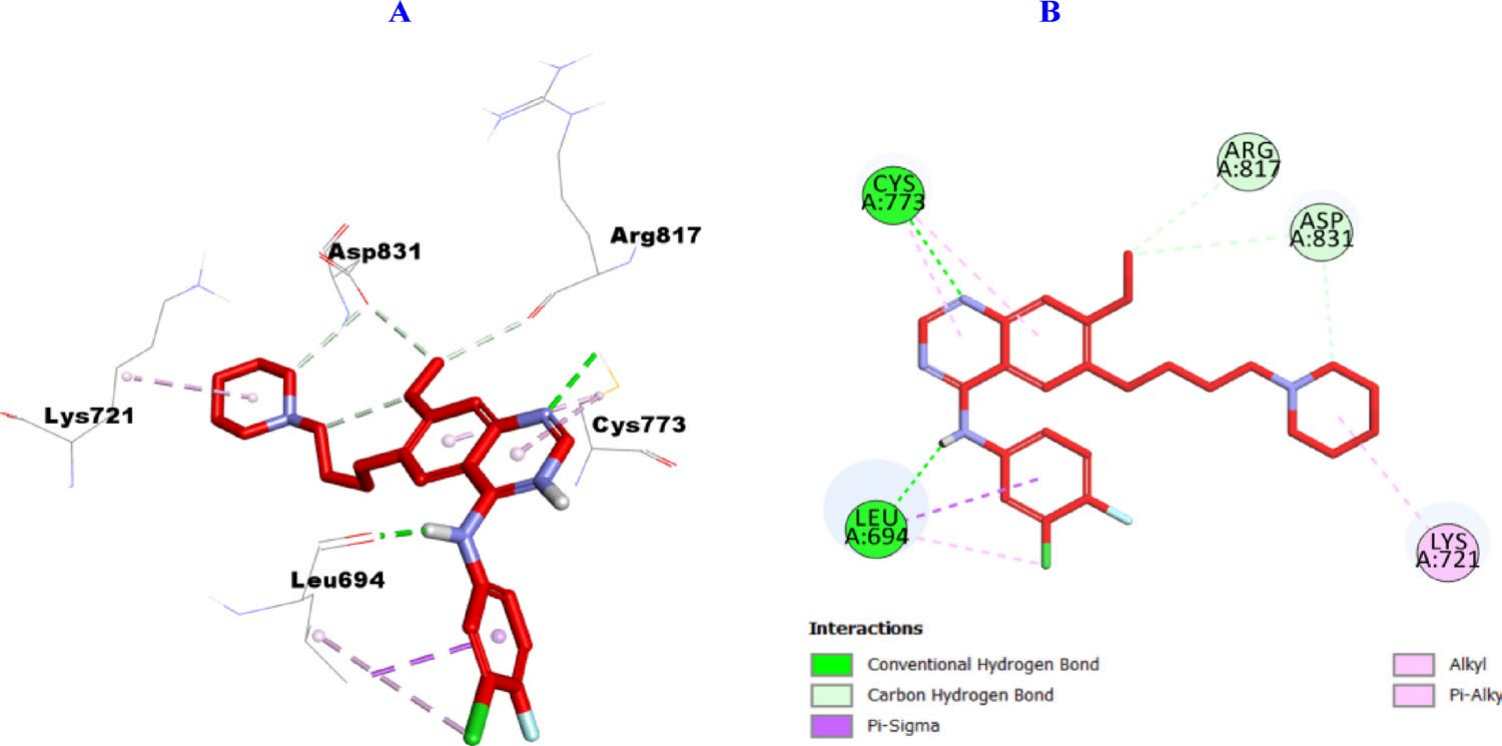
3D (**A**)/2D (**B**) Binding modes/interactions of gefitinib into the active site of EGFR (PDB ID: 1M17)

**Fig. 15:**
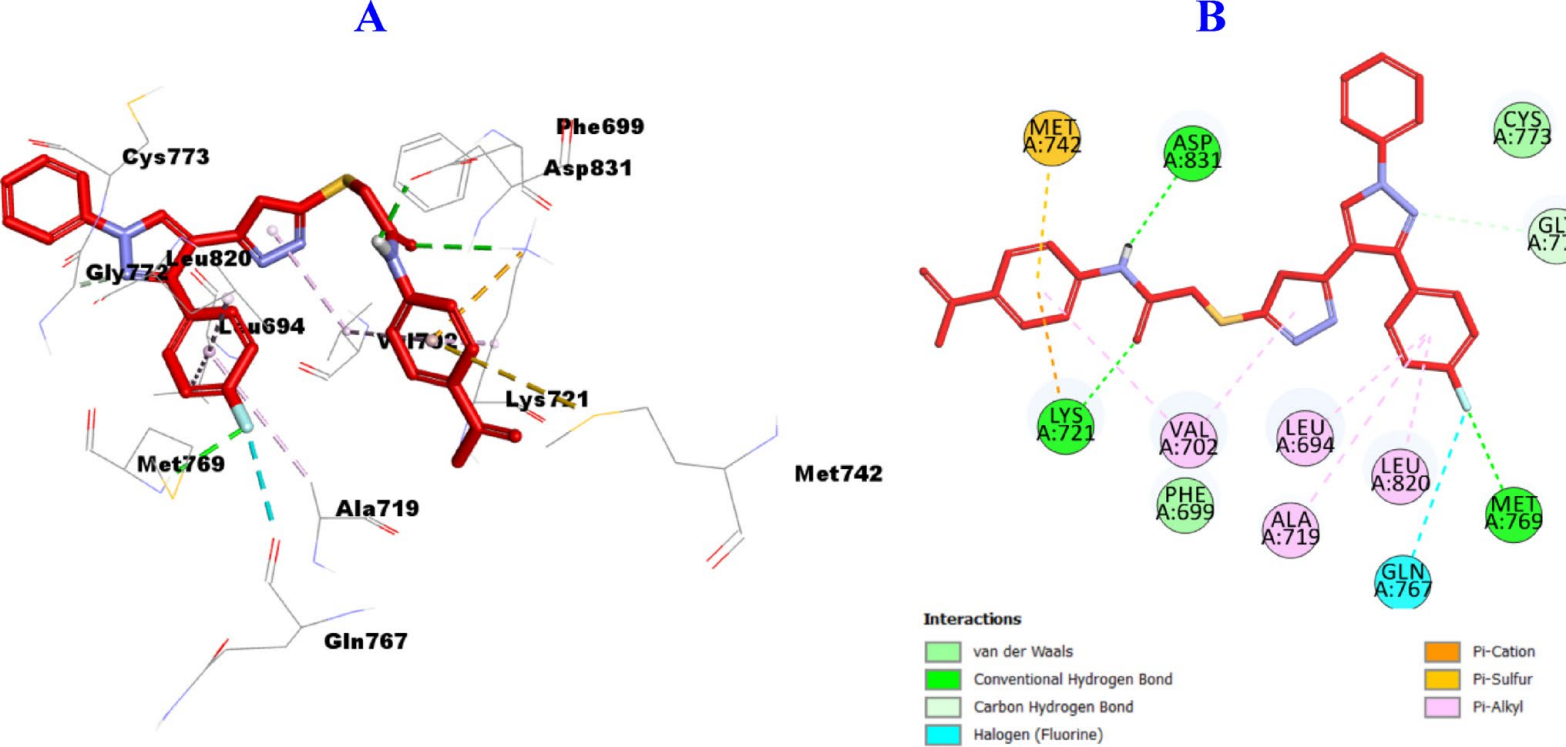
3D (**A**)/2D (**B**) Binding modes/interactions of hybrid **10b** into the active site of EGFR (PDB ID: 1M17)

**Fig. 16:**
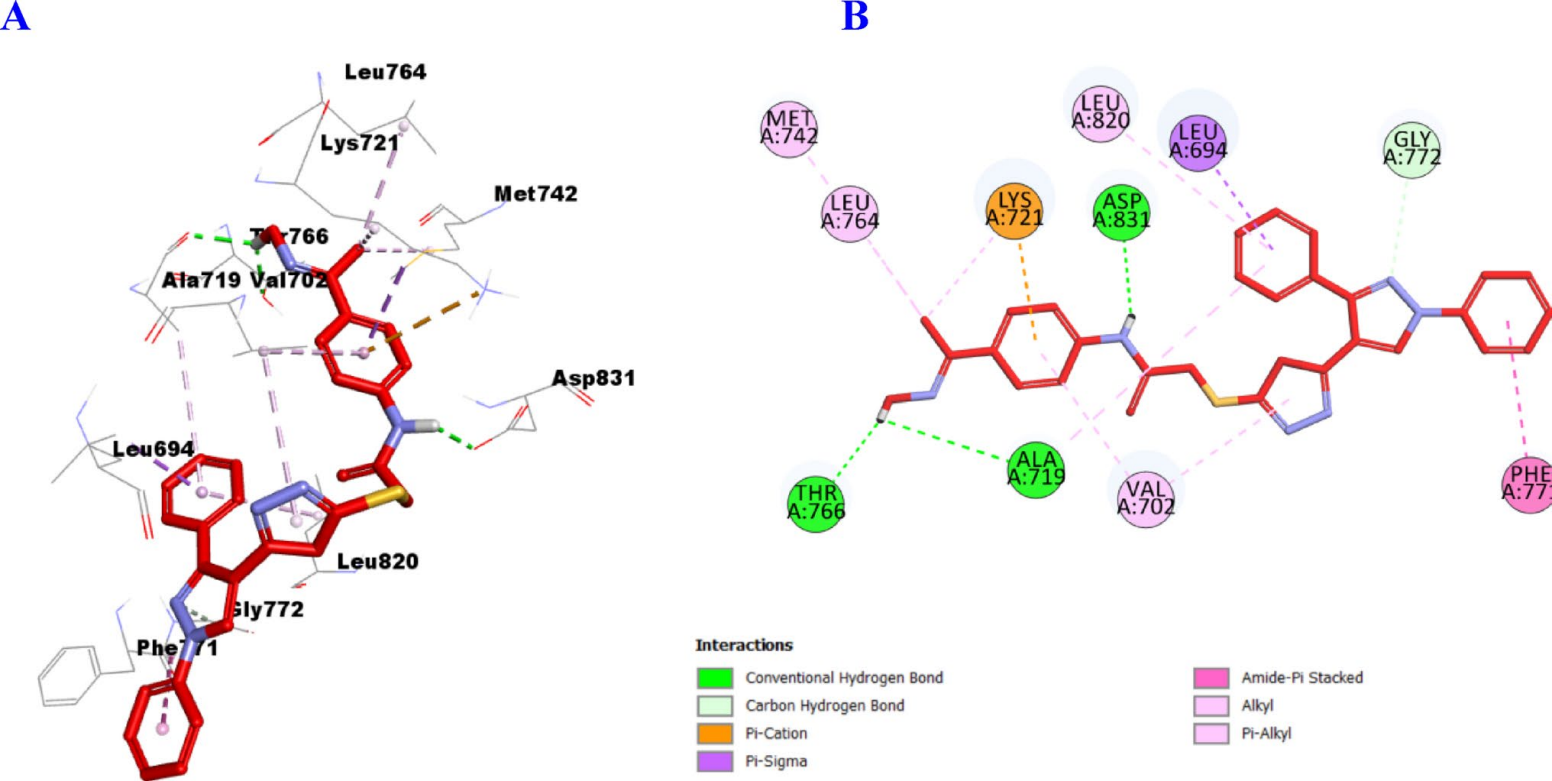
3D (**A**)/2D (**B**) Binding modes/interactions of hybrid **11a** into the active site of EGFR (PDB ID: 1M17)

**Fig. 17:**
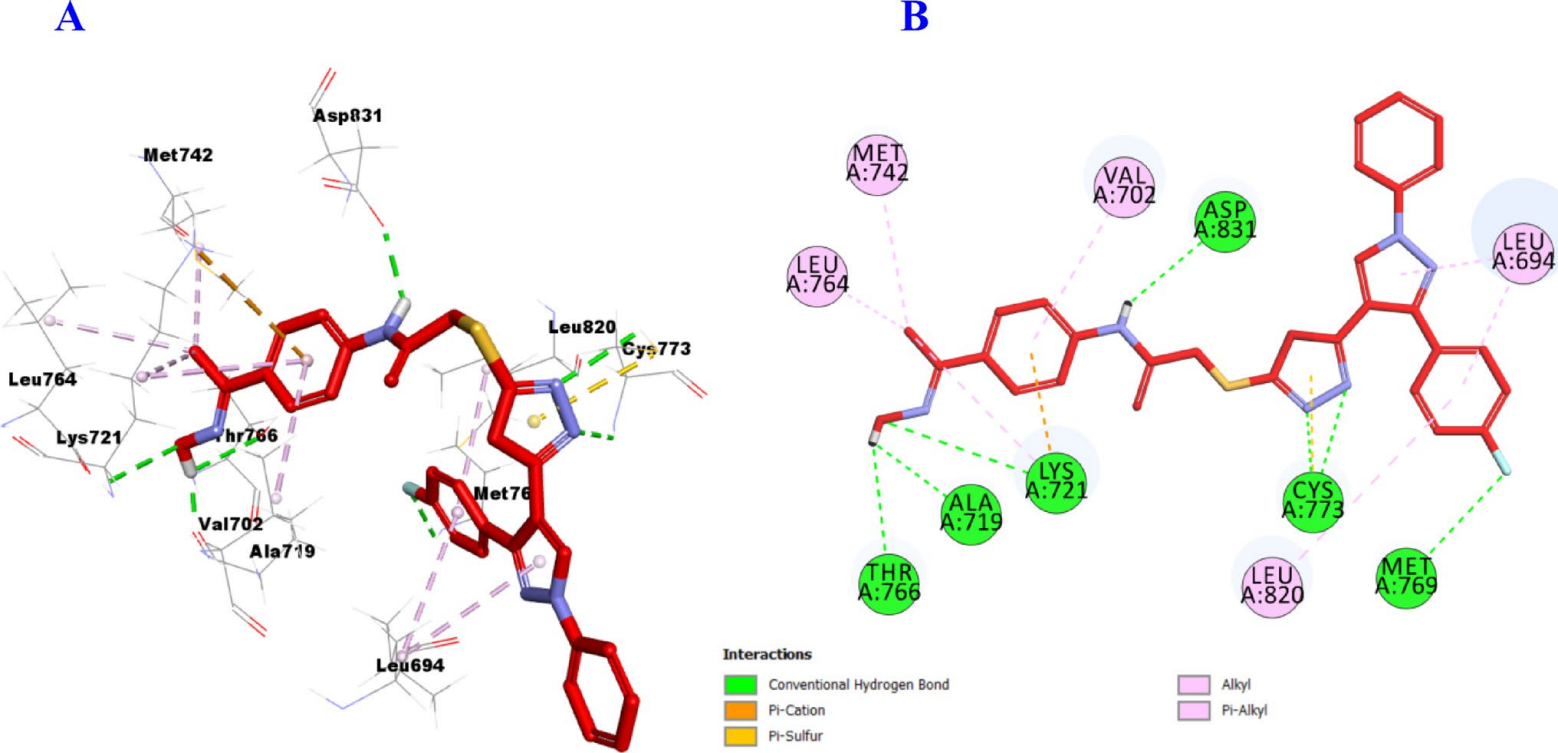
3D (**A**)/2D (**B**) Binding modes/interactions of hybrid **11b** into the active site of EGFR (PDB ID:1M17)

Docking results against EGFR (PDB ID: 1M17), showed consistency with the in vitro enzymatic assay results with the results being illustrated in Figs. 14, 15, 16 and 17. From the inspection of the docking results, the reference drug gefitinib as presented in Fig. 14 was incorporated in the formation of two hydrogen bonds with Leu694, and Cys773 amino acid residues, three carbon hydrogen bonds with Arg817 and Asp831 (2), as well as several hydrophobic interactions including one Pi-Sigma with Leu694, and four Pi-Alkyl with Leu694, Lys721, Cys773 (2) amino acid residues.

Furthermore, in agreement with the in-vitro enzymatic assay, hybrid **10b** showed enhanced binding affinity toward the binding site of EGFR (PDB ID: 1M17), and formed three hydrogen bonds with Lys721, Met769, Asp831 amino acid residues, one carbon hydrogen bond with Gly772, one halogen bond with Gln767 as well as several hydrophobic interactions including one Pi-sulfur with Met742, one Pi-Cation with Lys721, and five Pi-Alkyl with Leu694, Val702 (2), Ala719, Leu820 amino acid residues (Fig. 15).

Similarly, hybrids **11a** and **11b** showed consistency with the in vitro enzymatic assay results as well, with significant increase in binding affinity with the active site of EGFR enzyme. For instance, compound **11a** (Fig. 16) formed three hydrogen bonds with Ala719, Thr766, and Asp831 amino acid residues, and carbon hydrogen bond with Gly772 as well as several hydrophobic interactions including one Pi-Cation with Lys721, one Pi-Sigma with Leu694. Furthermore, it showed one Amide-Pi Stacked interaction with Phe771, one Alkyl with Leu820, and four Pi-Alkyl with Val702 (2), Met742, and Leu764 amino acid residues.

Finally, compound **11b** showed outstanding affinity toward the active site of EGFR and formed seven hydrogen bonds with Ala719, Lys721, Thr766, Met769, Cys773 (2), and Asp831 amino acid residues, as well as several hydrophobic interactions including two Pi-Cation with Lys721, and one Pi-Sulfur with Cys773. Additionally, it showed six Pi-Alkyl with Leu694 (2), Val702, Met742, Leu764, and Leu820 amino acid residues (Fig. 17).

Finally, from the previously mentioned data the binding mode of the compounds with the active sites of the enzymes reveals a complex network of interactions. The two nitrogens of the oxadiazole ring engaged in multiple hydrogen bonds with EGFR, enhancing the compound’s affinity for this target. Additionally, the acetamide and oxime groups are involved in the formation of hydrogen bonds with all studied enzymes, contributing to their inhibitory activity. The 1,3,4-trisubstituted pyrazole moiety participated in numerous hydrophobic interactions with all molecular targets, stabilizing the compound-enzyme complexes. Notably, the fluorine atom forms both halogen and hydrogen bonds with EGFR and VEGFR-2, further strengthening the binding interactions.

#### Physicochemical, ADME, pharmacokinetic characteristics and drug-likeness forecast

The Swiss Institute of Bioinformatics (SIB) provides the purchase free simply entered Swiss ADME website, which is one of the greatest useful computational systems for providing a worldwide evaluation of the pharmacokinetics characterization and prospects of the drug-likeness feature of tiny scaffolds. To save time, money, and enhance the likelihood of success, this was done to make sure the newly synthesized scaffolds had promising physiological potency and pharmacokinetic features. The most effective antiproliferative hybrids (**10b**,** 11a**, and **11b**) had strong hydrophilicity, and good BBB penetration, with projected log P_o/w_ values of 4.49, 4.18, and 4.50, respectively.

**Fig. 18:**
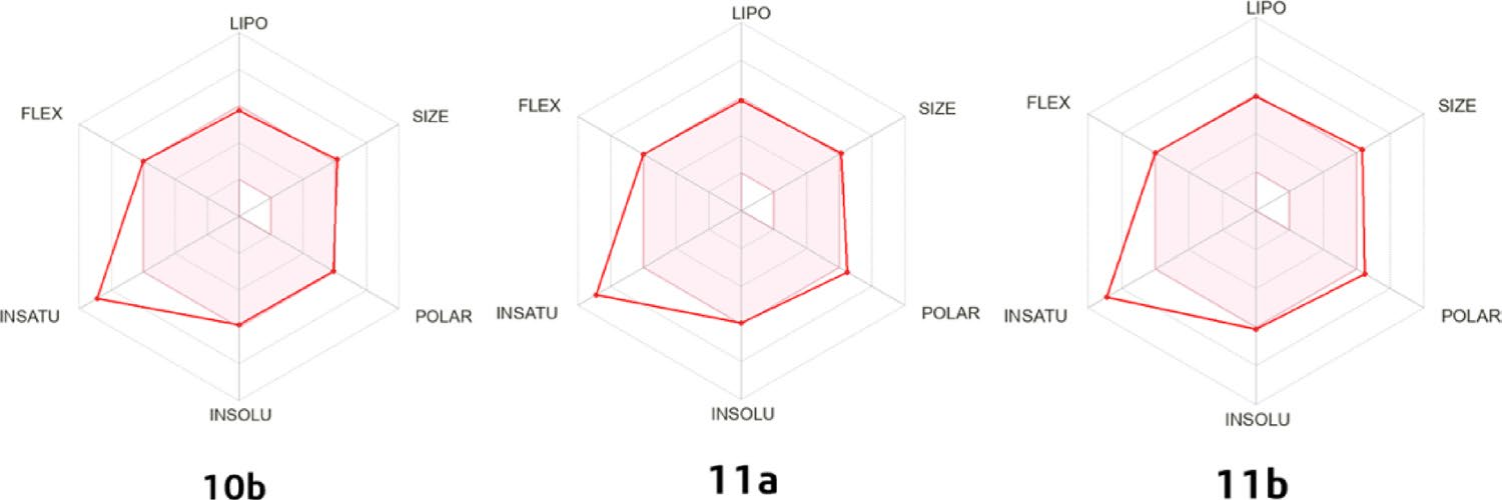
The bioavailability radar chart for hybrids **10b**, **11a**, and **11b**. The pink area represents the range of ideal property values. The red lines characterize hybrids **10b**, **11a**, and **11b**, whose estimated properties are almost entirely comprised in the pink area, indicating their good oral bioavailability

Based on drug-likeness criteria of the Lipinski and Veber rules, the oral bioavailability of the target compounds **10b**,** 11a**, and **11b** was assessed (Table 4). All compounds have an unsaturation property that is outside the colored zone (Fig. 18) with Fraction Csp3 value of 0.07 which indicate less hydrophobicity. Moreover, the solubility of the compounds in water is poorly soluble. Furthermore, the compound with the best profile, according to the toxicity parameters, is **11b**, followed by **11a**, then **10b**. All the compounds belong to the category of non-hERG I inhibitors, non-AMES toxicity, and non-skin sensitizers. Furthermore, the results showed that compound **11b** is not an inhibitor of CYP1A2 and CYP2D6, but it exhibited inhibitory activity against CYP2C19, CYP2C9, and CYP3A4.

**Table 4:** Physicochemical characteristics, lipophilicity, water solubility, pharmacokinetics, drug likeness, medicinal chemistry, and toxicity parameters of compounds **10b**, **11a**, and **11b** that we acquired from Swiss ADME and PkCSM server

Physicochemical properties		10b			11a			11b		
Formula		C_27_H_20_FN_5_O_3_S			C_27_H_22_N_6_O_3_S			C_27_H_21_FN_6_O_3_S		
Molecular weight		513.54 g/mol			510.57 g/mol			528.56 g/mol		
Num. heavy atoms		37			37			38		
Num. arom. heavy atoms		28			28			28		
Fraction Csp3		0.07			0.07			0.07		
Num. rotatable bonds		9			9			9		
Num. H‐bond acceptors		7			7			8		
Num. H‐bond donors		1			2			2		
Molar refractivity		138.00			142.03			141.99		
TPSA (topological polar surface area)		128.21 Å^2^			143.73 Å^2^			143.73 Å^2^		
Log *P*_o/w_		4.49			4.18			4.50		
*Water solubility*										
Log *S* (ESOL)		-5.88			-5.95			-6.11		
Solubility		6.84e-04 mg/ml; 1.33e-06 mol/l			5.71e-04 mg/ml; 1.12e-06 mol/l			4.09e-04 mg/ml ; 7.74e-07 mol/l		
Class		Moderately soluble			Moderately soluble			Poorly soluble		
*Pharmacokinetics*										
GI absorption		Low			Low			Low		
BBB permeant		No			No			No		
P‐gp substrate		No			No			No		
Log *K*_p_ (skin permeation)		-6.18 cm/s			-6.06 cm/s			-6.09 cm/s		
*Physicochemical properties*										
Druglikeness										
Lipinski		Yes;	1	Violation	Yes;	1	Violation	Yes;	1	Violation
Veber		Yes			No	1	Violation	No	1	Violation
Muegge		Yes			Yes			Yes		
Bioavailability	Score	0.55			0.55			0.55		
*Toxicity*										
AMES toxicity (Yes/No)		No			No			No		
Max. tolerated dose (human) (log mg/ kg/day)		0.757			0.742			0.775		
hERG I inhibitor (YES/NO)		No			No			No		
hERG II inhibitor (Yes/No)		Yes			Yes			Yes		
Oral rat acute toxicity (LD50) (mol/kg)		3.104			2.934			2.927		
Oral rat chronic toxicity (LOAEL) (log mg/Kg bw/day)		-0.481			0.854			0.834		
Hepatotoxicity *(Yes/No)*		Yes			Yes			Yes		
Skin sensitization* (Yes/No)*		No			No			No		
*T. pyriformis* toxicity (log μg/L)		0.285			0.285			0.285		
Minnow toxicity (log mM)		-0.073			0.25			0.375		

#### Structure activity relationship (SAR)

**Fig. 19:**
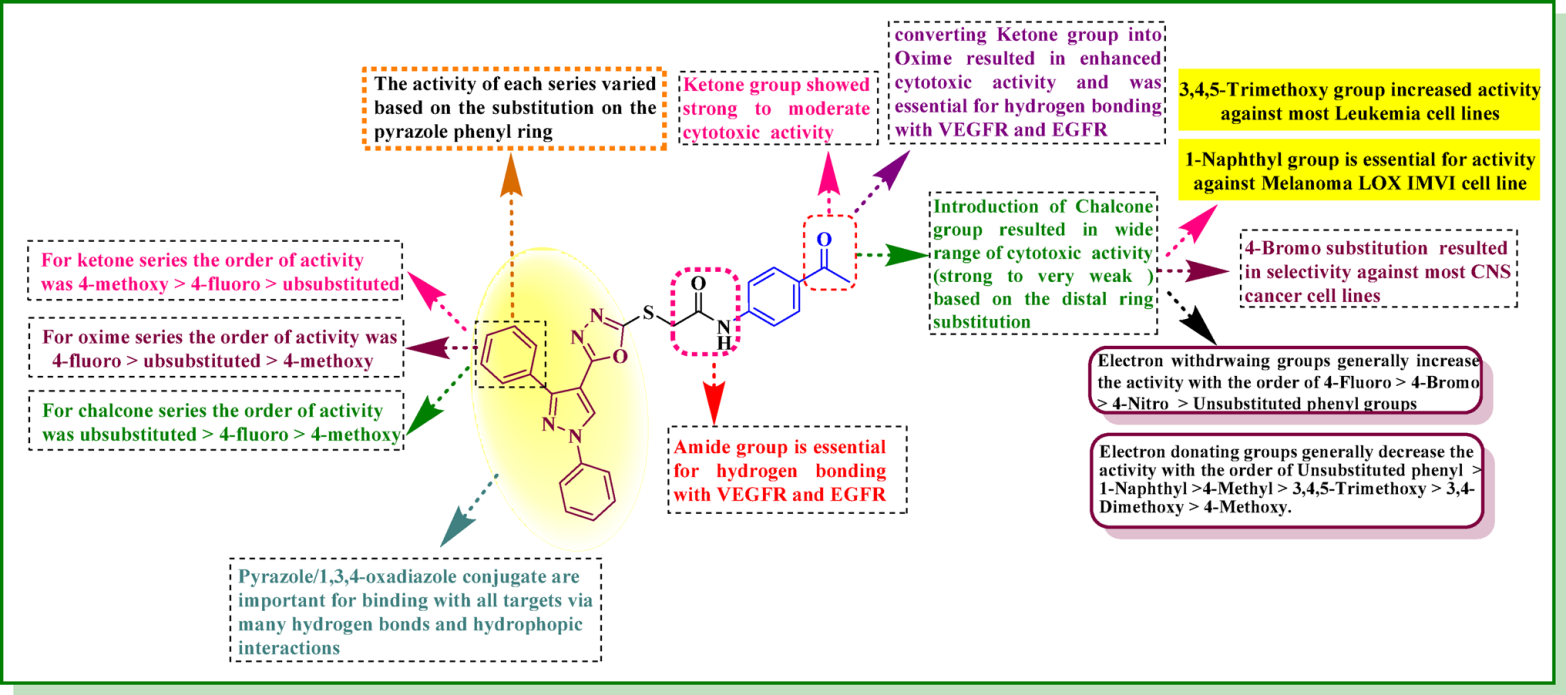
SAR of the pyrazole-oxadiazole derivatives

The SAR analysis revealed that the nature of the substituent on the phenyl ring at position 3 of the pyrazole and the functional group attached to the phenylacetamide moiety significantly influenced anticancer activity. The ketone series (**10a-10c**) with acetyl group attached to the phenylacetamide moiety exhibited strong to moderate anticancer activity. Among these, the compound with a 4-methoxyphenyl substitution at position 3 of the pyrazole **10c** demonstrated the highest activity. The order of activity was determined to be 4-methoxypheny > 4-fluorophenyl > unsubstituted phenyl. Moreover, conversion of the acetyl group to an oxime functionality in series **11a-11c** resulted in a significant increase in activity. Furthermore, compound **11b** with 4-fluorophenyl group was the most active, followed by the unsubstituted phenyl **11a** and the 4-methoxyphenyl **11c**. Lastly, the introduction of a chalcone group in hybrids **12a-12i**, **13a-13i**, and **14a-14i** generally led to a decrease in activity compared to the ketone and oxime derivatives. However, the chalcones activity varied significantly based on the substitutions on both pyrazole C3 phenyl ring and chalcone distal phenyl ring. The pyrazole C3 phenyl ring substitutions followed the order: unsubstituted phenyl > 4-fluorophenyl > 4-methoxyphenyl. For the chalcone distal phenyl ring, electron-withdrawing groups increased activity compared to unsubstituted derivatives, while electron-donating groups decreased activity. The overall order of activity was: 4-fluoro < 4-bromo < 4-nitro < unsubstituted < 1-naphthyl < 4-methyl < 3,4,5-trimethoxy < 3,4-dimethoxy < 4-methoxy. The 3,4,5-trimethoxy group showed increased selectivity towards the leukemia subpanel, and the 1-naphthyl group was selective towards the melanoma LOX IMVI cell line as illustrated in SAR (Fig. 19).

## Conclusion

In conclusion, this work focused on designing, synthesizing, and evaluating a novel series of pyrazole-1,3,4-oxadiazole hybrid compounds linked to chalcone as potential dual inhibitors of EGFR and VEGFR-2 enzymes for anticancer therapy. In-vitro screening revealed that compounds **10b**, **11a**, **11b**, and **11c** exhibited significant cytotoxic activity. Compound **10b** showed a mean GI_50_ of 6.39 µM, while **11a** and **11b** had mean GI_50_ values of 4.88 µM and 4.36 µM, respectively. Compound **11c** was effective with a mean GI_50_ of 16.4 µM. Enzymatic assays confirmed the dual inhibitory activity, with compounds **10b**, **11a**, and **11b** showing potent inhibition of EGFR and VEGFR-2. Notably, **11b** induced G2/M phase arrest and apoptosis in MCF7 cells. Molecular modeling and NO release studies supported these findings, highlighting the therapeutic potential of these hybrids. Overall, compounds **10b**, **11a**, **11b**, and **11c** are promising anticancer leads for further development.

## Experimental

### Chemistry

#### Materials and methods

All chemicals used in the preparation of the intermediate and target compounds are of commercial grade, purchased from Merck, Fluka, and El-Nasr pharmaceutical chemicals companies. All chemicals are used without further purification. Solvents used are of commercial grade and purchased from Merck, and El-Nasr pharmaceutical chemicals companies. DMF was dried with anhydrous sodium sulphate and purified by distillation on rotary evaporator.

Melting points were determined on Stuart electro-thermal melting point apparatus and were uncorrected. NMR spectra were carried out using a Bruker Advance 400 MHz NMR spectrometer at Bani-Swef University-Faculty of pharmacy, using TMS as internal reference. Chemical shifts (δ) values are given in parts per million (ppm) relative to DMSO-*d*_6_ (2.50 for proton and 39.50 for carbon) and coupling constants (*J*) in Hertz. Splitting patterns are designated as follows: s, singlet; d, doublet; t, triplet; q, quartet; dd, doublet of doublet; m, multiplet. The progress of reactions for preparation of compounds was monitored by thin-layer chromatography (TLC) using Merck 9385 pre-coated aluminum plate silica gel (Kieselgel 60) 5 × 20 cm plates with a layer thickness of 0.2 mm. The spots were detected by exposure to UV-lamp at λ = 254 nm. Elemental analyses were performed on Vario El Elementar CHN Elemental analyzer; organic microanalysis section, The Regional center for Mycology and Biotechnology, Naser City, Cairo, Egypt and the results were within ± 0.4% of the theoretical values.

#### General procedure for the synthesis of 5-(1-phenyl-3-substitutedphenyl-1 H-pyrazol-4-yl)-1,3,4-oxadiazole-2-thiol (9a-9c)

In an ice bath, an equimolar mixture of potassium hydroxide (0.561 g, 10 mmol) in 20 mL of ethanol was combined with the appropriate hydrazide (**8a-8c**) (10 mmol) and carbon disulfide (0.761 g, 10 mmol). The mixture was stirred for 10 min and then heated under reflux for 6 h, or until the evolution of hydrogen sulfide had nearly ceased. The reaction progress was monitored by TLC using a methylene chloride and methanol elution system (9:1 v/v). Once the reaction was deemed complete, ethanol was removed under vacuum. The resulting solid residue was dissolved in cold water, filtered, and acidified with diluted hydrochloric acid to yield the desired oxadiazoles (**9a-9c**). The product was then filtered, washed with water, dried, and crystallized from 90% aqueous ethanol.

##### 5-(1,3-Diphenyl-1*H*-pyrazol-4-yl)-1,3,4-oxadiazole-2-thiol (9a)

White crystals (yield, 2.8 g 88%); m.p. 225–227 °C; FT-IR; ν_max_ 2758 (SH); ^1^H NMR (400 MHz, DMSO-*d*_*6*_) δ (ppm): 14.6 (s, 1H, NH, D_2_O exchangeable), 9.3 (s, 1H, pyrazole C5–H), 8.03 (d, 2 H, *J* = 8.00 Hz Ar–H), 7.88–7.86 (m, 2 H, Ar-H), 7.59–7.48 (m, 2 H, Ar-H), 7.44–7.42 (m, 3 H, Ar-H), 7.41–7.37 (m, 1H, Ar-H); ^13^C-NMR (101 MHz, DMSO-*d*_*6*_) δ (ppm): 177.19 (oxadiazole C_2_), 156.68 (oxadiazole C_5_), 150.92 (pyrazole C_3_), 139.10 (pyrazole C_5_), 131.81, 131.67, 130.15, 129.48, 129.17, 128.69, 127.99, 119.52, 105.49 (pyrazole C_4_); Elemental analysis for C_17_H_12_N_4_OS (320.37) (Calcd./Found): C, 63.73/63.91; H, 3.78/3.89; N, 17.49/17.65.

##### 5-(3-(4-Fluorophenyl)-1-phenyl-1*H*-pyrazol-4-yl)-1,3,4-oxadiazole-2-thiol (9b)

Light brown crystals (yield, 2.73 g 81%); m.p. 229–230 °C, MW. 338.36; FT-IR; ν_max_ 2759 (SH); ^1^H NMR (400 MHz, DMSO-*d*_*6*_) δ (ppm): ), 14.56 (s, 1H, NH, D_2_O exchangeable), 9.31 (s, 1H, pyrazole C_5_–H), 8.02 (d, 2 H, *J* = 8.00 Hz Ar–H), 7.93 (d, 2 H, *J* = 8.00 Hz Ar–H), 7.56–7.54 (m, 2 H, Ar-H), 7.43–7.40 (m, 1H, Ar-H), 7. 35-7.32 (**m**, 2 H,, Ar–H); ^13^C-NMR (101 MHz, DMSO-*d*_*6*_) δ (ppm): 177.19 (oxadiazole C_2_), 164.27, 161.82, 156.61 (oxadiazole C_5_), 149.91 (pyrazole C_3_), 139.00 (pyrazole C_5_), 131.71, 131.49, 131.40, 130.14, 128.12, 128.01, 119.48, 115.74, 115.53, 105.48 (pyrazole C_4_); Elemental analysis for C_17_H_11_FN_4_OS (338.36) (Calcd./Found): C, 60.35/60.37; H, 3.28/3.30; N, 16.56/16.59.

##### 5-(3-(4-Methoxyphenyl)-1-phenyl-1*H*-pyrazol-4-yl)-1,3,4-oxadiazole-2-thiol (9c)

White crystals (yield, 2.55 g 73%); m.p. 209–210 °C; FT-IR; ν_max_ 2760 (SH); ^1^H NMR (400 MHz, DMSO-*d*_*6*_) δ (ppm): 9.26 (s, 1H, pyrazole C_5_–H), 8.01 (d, 2 H, *J* = 8.00 Hz Ar–H), 7.82 (d, 2 H, *J* = 8.00 Hz Ar–H), 7.60–7.5 (m, 2 H, Ar-H), 7.45–7.35 (m, 1H, Ar-H), 7. 04 (d, 2 H, *J* = 8.00 Hz, Ar–H), 3.83 (s, 3 H, OCH_3_ ); ^13^C-NMR (101 MHz, DMSO-*d*_*6*_) δ (ppm): 177.22 (oxadiazole C_2_), 160.35, 156.82 (oxadiazole C_5_), 150.71 (pyrazole C_3_), 139.08 (pyrazole C_5_), 131.57, 130.52, 130.14, 127.87, 123.97, 119.41, 114.12, 105.22 (pyrazole C_4_), 55.70 (OCH_3_) ; C_18_H_14_N_4_O_2_S (350.40) (Calcd./Found): C, 61.70/61.74; H, 4.03/4.05; N, 15.99/16.02.

#### General procedure for the synthesis of N-(4-acetylphenyl)-2-((5-(1-phenyl-3-substitutedphenyl-1 H-pyrazol-4-yl)-1,3,4-oxadiazol-2-yl)thio)acetamide (10a-10c)

A mixture of the appropriate oxadiazole (**9a-9c**) (1 mmol), triethylamine (3 mmol), and compound **3** (0.211 g, 1 mmol) was stirred at room temperature in 20 ml of acetonitrile for 4 h. TLC was used to follow the reaction progression. The obtained product was filtered off and washed with water, dried, crystallized from methanol to afford compounds (**10a-10c**) in a good yeild.

##### *N*-(4-Acetylphenyl)-2-((5-(1,3-diphenyl-1*H*-pyrazol-4-yl)-1,3,4-oxadiazol-2-yl)thio)acetamide (10a)

White crystals (0.193 g, 78% yield); m.p. 169–172 °C; FT-IR; ν_max_ 3350 (NH), 1675 (Ketone C = O), 1598 (amide C = O); ^1^H NMR (400 MHz, DMSO-*d*_*6*_) δ (ppm): 10.85 (s, 1H, CO-N**H**), 9.28 (s, 1H, pyrazole C_5_–H), 7.99 (d, 2 H, *J* = 8.00 Hz, Ar–H), 7.95–7.88 (m, 4 H, Ar-H), 7.75–7.70 (m, 3 H, Ar-H), 7.62–7.55 (m, 2 H, Ar-H), 7.49–7.41 (m, 3 H, Ar-H), 4.36 (s, 2 H, C**H**_**2**_) 2.52 (s, 3 H, C**H**_**3**_) under DMSO peak; ^13^C-NMR (100 MHz, DMSO-*d*_*6*_) δ (ppm): 196.95 (ketone C = O), 165.99 (amide C = O), 162.82 (oxadiazole C_2_), 161.03 (oxadiazole C_5_), 150.92 (pyrazole C_3_), 143.41 (C- NH), 139.14 (pyrazole C_5_), 132.60, 131.79, 131.68, 130.16, 129.97, 129.42, 129.05, 128.69, 127.97, 119.49, 118.99, 105.80 (pyrazole C_4_), 37.32 (CH_2_), 26.86 (CH_3_); Elemental analysis for C_27_H_21_N_6_O_3_S (495.56) (Calcd./Found): C, 65.44/65.46; H, 4.27/4.28; N, 14.13/14.15; HPLC analysis: Mobile phase (dipotassium hydrogen phosphate buffer [pH 3.2]: ACN, 30:70 respectively) FR 1mL /min, retention time = 4.7 min; peak area, 94.6% at λ_max_ 254 nm.

##### *N*-(4-acetylphenyl)-2-((5-(3-(4-fluorophenyl)-1-phenyl-1*H*-pyrazol-4-yl)-1,3,4-oxadiazol-2-yl)thio)acetamide (10b)

White crystals (0.156 g, 61% yield); m.p. 179–181 °C; FT-IR; ν_max_ 3346 (NH), 1679 (Ketone C = O), 1602 (amide C = O); ^1^H NMR (400 MHz, DMSO-*d*_*6*_) δ (ppm): 10.92 (s, 1H, CO-N**H**), 9.28 (s, 1H, pyrazole C_5_–H), 8.02-7. 98 (m, 4 H, Ar–H), 7.93 (d, 2 H, *J* = 8.00 Hz, Ar-H), 7.76 (d, 2 H, *J* = 8.00 Hz, Ar-H), 7.58–7.54 (m, 2 H, Ar-H), 7.45–7.40 (m, 1H, Ar-H), 7.31 (m, 2 H, Ar-H), 4.36 (s, 2 H, C**H**_**2**_) 2.52 (s, 3 H, C**H**_**3**_) under DMSO peak; ^13^C-NMR (100 MHz, DMSO-*d*_*6*_) δ (ppm): 196.95 (ketone C = O), 166.02 (amide C = O), 164.24, 162.80 (oxadiazole C_2_), 161.80 (oxadiazole C_5_), 160.98, 149.89 (pyrazole C_3_), 143.40 (C- NH), 139.06 (pyrazole C_5_), 132.60, 131.66, 131.35, 131.27, 130.13, 129.95, 128.25, 127.98, 119.46, 118.99, 115.72, 115.50, 105.77 (pyrazole C_4_), 37.31(CH_2_), 26.83 (CH_3_); Elemental analysis for C_27_H_20_FN_5_O_3_S (513.55) (Calcd./Found): C, 63.15/63.18; H, 3.93/3.93; N, 13.64/13.66; HPLC analysis: Mobile phase (dipotassium hydrogen phosphate buffer [pH 3.2]: ACN, 30:70 respectively) FR 1mL /min, retention time = 3.7 min; peak area, 99.1% at λ_max_ 254 nm.

##### *N*-(4-acetylphenyl)-2-((5-(3-(4-methoxyphenyl)-1-phenyl-1*H*-pyrazol-4-yl)-1,3,4-oxadiazol-2-yl)thio)acetamide (10c)

White crystals (0.176 g, 67% yield); m.p. 165–166 °C; FT-IR; ν_max_ 3308 (NH), 1676 (Ketone C = O), 1603 (amide C = O); ^1^H NMR (400 MHz, DMSO-*d*_*6*_) δ (ppm): 10.86 (s, 1H, CO-N**H**), 9.23 (s, 1H, pyrazole C_5_–H), 7.99–7.88 (m, 6 H, Ar–H), 7.75 (d, 2 H, *J* = 8.00 Hz, Ar-H), 7.58–7.55 (m, 2 H, Ar-H), 7.44–7.40 (m, 1H, Ar-H), 7.03 (d, 2 H, *J* = 8.00 Hz, Ar-H), 4.37 (s, 2 H, C**H**_**2**_), 3.81 (s, 3 H, OC**H**_**3**_) 2.52 (s, 3 H, C**H**_**3**_) under DMSO peak; ^13^C-NMR (100 MHz, DMSO-*d*_*6*_) δ (ppm): 196.94 (ketone C = O), 166.02 (amide C = O), 162.71 (oxadiazole C_2_), 161.20 (oxadiazole C_5_), 160.35, 150.72 (pyrazole C_3_), 143.39 (C- NH), 139.15 (pyrazole C_5_), 132.61, 131.51, 130.41, 130.11, 129.97, 127.82, 124.15, 119.38, 118.99, 114.13, 105.46 (pyrazole C_4_), 55.67 (OCH_3_), 37.34 (CH_2_), 26.84 (CH_3_); Elemental analysis for C_28_H_23_N_5_O_4_S (525.58) (Calcd./Found): C, 63.99/64.01; H, 4.41/4.42; N, 13.33/13.35; HPLC analysis: Mobile phase (dipotassium hydrogen phosphate buffer [pH 3.2]: ACN, 30:70 respectively) FR 1mL /min, retention time = 3.7 min; peak area, 99.1% at λ_max_ 254 nm.

##### General procedure for the synthesis of (*E*)-2-((5-(1-phenyl-3-substitutedphenyl-1 H-pyrazol-4-yl)-1,3,4-oxadiazol-2-yl)thio)-N-(4-(1-(hydroxyimino)ethyl)phenyl)acetamide (11a-11c)

To a mixture of the desired ketones **10a-10c** (0.3 mmol) and pyridine (0.079 g, 1.0 mmol) in absolute ethanol (30 ml) hydroxylamine hydrochloride (0.041 g, 0.6 mmol) added and refluxed for 4 h, and then left to cool. The formed precipitate was filtered off, washed with distilled water, dried, and crystallized from absolute ethanol affording the pure products **11a-11c** in a good yield.

##### (*E*)-2-((5-(1,3-Diphenyl-1*H*-pyrazol-4-yl)-1,3,4-oxadiazol-2-yl)thio)-*N*-(4-(1-(hydroxyimino)ethyl)phenyl)acetamide (11a)

White crystals (0.84 g, 55% yield); m.p. 191–193 °C; FT-IR; ν_max_ 3292 (NH), 1597 (amide C = O), 1533 (C = N); ^1^H NMR (400 MHz, DMSO-*d*_*6*_) δ (ppm): 11.05 (s, 1H, C = N-O**H**), 10.68 (s, 1H, CO-N**H**), 9.30 (s, 1H, pyrazole C_5_–H), 8.00 (d, 2 H, *J* = 8.00 Hz, Ar–H), 7.92 (d, 2 H, *J* = 8.00 Hz, Ar-H), 7.62 -7.60-7.55(m, 6 H, Ar-H), 7.49–7.42 (m, 4 H, Ar-H), 4.33 (s, 2 H, C**H**_**2**_), 2.12 (s, 3 H, C**H**_**3**_); ^13^C-NMR (100 MHz, DMSO-*d*_*6*_) δ (ppm): 165.46 (amide C = O), 162.89 (oxadiazole C_2_), 160.97(oxadiazole C_5_), 152.84 (oxime C = N), 150.68 (pyrazole C_3_), 139.50 (pyrazole C_5_), 139.09, 132.65, 131.75, 131.68, 130.19, 129.45, 129.08, 128.71, 127.99, 126.56, 119.43, 119.32, 105.81 (pyrazole C_4_), 37.21(CH_2_), 11.83 (CH_3_); Elemental analysis for C_27_H_22_N_6_O_3_S (510.57) (Calcd./Found): C, 63.52/63.55; H, 4.34/4.35; N, 16.46/16.48; HPLC analysis: Mobile phase (dipotassium hydrogen phosphate buffer [pH 3.2]: ACN, 30:70 respectively) FR 1mL /min, retention time = 3.3 min; peak area, 98.3% at λ_max_ 254 nm.

##### (*E*)-2-((5-(3-(4-Fluorophenyl)-1-phenyl-1*H*-pyrazol-4-yl)-1,3,4-oxadiazol-2-yl)thio)-*N*-(4-(1-(hydroxyimino)ethyl)phenyl)acetamide (11b)

White crystals (0.97 g, 62% yield); m.p. 208–210 °C; FT-IR; ν_max_ 3311 (NH), 1599 (amide CO), 1516 (C = N); ^1^H NMR (400 MHz, DMSO-*d*_*6*_) δ (ppm): 11.06 (s, 1H, C = N-O**H**), 10.96 (s, 1H, CO-N**H**), 9.28 (s, 1H, pyrazole C_5_–H), 8.01–7.93 (m, 4 H, Ar–H), 7.67-7.57- (m, 6 H, Ar–H), 7.43–7.42 (m, 1H, Ar–H), 7.33–7.30 (m, 2 H, Ar–H), 4.39 (s, 2 H, C**H**_**2**_), 2.12 (s, 3 H, C**H**_**3**_); ^13^C-NMR (100 MHz, DMSO-*d*_*6*_) δ (ppm): 165.57 (amide C = O), 164.22, 162.84 (oxadiazole C_2_), 161.78 (oxadiazole C_5_), 160.91, 152.88 (oxime C = N), 149.85 (pyrazole C_3_), 139.51(pyrazole C_5_), 138.99, 132.63, 131.67, 131.39, 131.31, 130.17, 128.19, 128.01, 126.52, 119.40, 119.33, 115.74, 115.33, 105.76 (pyrazole C_4_), 37.11 (CH_2_), 11.81(CH_3_); Elemental analysis for C_27_H_21_FN_6_O_3_S (528.56) (Calcd./Found): C, 61.35/61.37; H, 4.00/4.02; N, 15.90/15.91; HPLC analysis: Mobile phase (dipotassium hydrogen phosphate buffer [pH 3.2]: ACN, 30:70 respectively) FR 1mL /min, retention time = 3.26 min; peak area, 98.9% at λ_max_ 254 nm.

##### (*E*)-*N*-(4-(1-(hydroxyimino)ethyl)phenyl)-2-((5-(3-(4-methoxyphenyl)-1-phenyl-1*H*-pyrazol-4-yl)-1,3,4-oxadiazol-2-yl)thio)acetamide (11c).

White crystals (0.115 g, 71% yield); m.p. 186–187 °C; FT-IR; ν_max_ 3227 (NH), 1599 (amide CO), 1516 (C = N); ^1^H NMR (400 MHz, DMSO-*d*_*6*_) δ (ppm): 11.06 (s, 1H, C = N-O**H**), 10.54 (s, 1H, CO-N**H**), 9.21 (s, 1H, pyrazole C_5_–H), 7.96–7.88 (m, 4 H, Ar–H), 7.61–7.41 (m, 7 H, Ar-H), 7.12–6.99 (m, 2 H, Ar-H), 4.32 (s, 2 H, C**H**_**2**_), 3.81 (s, 3 H, OC**H**_**3**_), 2.13 (s, 3 H, C**H**_**3**_); ^13^C-NMR (100 MHz, DMSO-*d*_*6*_) δ (ppm): 165.43 (amide C = O), 162.79 (oxadiazole C_2_), 161.13 (oxadiazole C_5_), 160.31, 152.80 (oxime C = N), 150.69 (pyrazole C_3_), 139.45 (pyrazole C_5_), 139.12, 132.68, 131.50, 130.43, 130.14, 127.83, 126.58, 124.09, 119.32, 114.11, 105.47 (pyrazole C_4_), 55.66 (OCH_3_), 37.28 (CH_2_), 11.82 (CH_3_); Elemental analysis for C_28_H_24_N_6_O_4_S (540.60) (Calcd./Found): C, 62.21/62.22; H, 4.48/4.50; N, 15.55/15.55; HPLC analysis: Mobile phase (dipotassium hydrogen phosphate buffer [pH 3.2]: ACN, 30:70 respectively) FR 1mL /min, retention time = 3.5 min; peak area, 97.2% at λ_max_ 254 nm.

#### General procedure for the synthesis of N-(4-cinnamoylphenyl)-2-((5-(1-phenyl-3-substitutedphenyl-1 H-pyrazol-4-yl)-1,3,4-oxadiazol-2-yl)thio)acetamide (12a-12i, 13a-13i and 14a-14i)

A mixture of the desired oxadiazoles (**9a-9c**) (1 mmol), triethylamine (303 mg, 3 mmol) and the desired chalcone derivatives (**2a-2i**) (1 mmol) was stirred at room temperature in 20 ml of acetonitrile for 4 h. TLC was used to follow the reaction progression. The formed precipitate was filtered off, washed with water, dried, and crystallized from acetonitrile to afford compounds (**12a-12i**, **13a-13i**, and **14a-14i**).

##### (*E*) *N*-(4-Cinnamoylphenyl)-2-[(5-(1,3-diphenyl-1*H*-pyrazol-4-yl)-1,3,4-oxadiazol-2-yl)thio)acetamide (12a).

White crystals (0.206 g, 71% yield); m.p. 230–232 °C; FT-IR; ν_max_ 3315 (NH), 1662 (Ketone CO), 1603 (amide CO); ^1^H NMR (400 MHz, DMSO-*d*_*6*_) δ (ppm): 10.82 (s, 1H, CO-NH), 9.22 (s, 1H, pyrazole C_5_–H), 8.16 (d, 2 H, *J* = 8.00 Hz, Ar–H), 7.98–7.53 (m, 2 H, Ar-H), 7.88–7.86 (m, 5 H, Ar-H), 7.78 (d, 2 H, *J* = 8.00 Hz, Ar–H),7.72 (d, 1H, C**H** = CH *J* = 16.00 Hz, Ar-H), 7.58–7.54 (m, 2 H, Ar-H), 7.48–7.45 (m, 6 H, Ar-H), 7.41 (t, 1H, *J* = 8.00 Hz Ar-H), 4.34 (s, 2 H, CH_2_); ^13^C-NMR (100 MHz, DMSO-*d*_*6*_) δ (ppm): δ 188.17 (ketone C = O), 166.05 (amide C = O), 162.86 (oxadiazole C_2_), 161.02 (oxadiazole C_5_), 150.96 (pyrazole C_3_), 144.05 (Ar-*C* = C), 143.43, 139.05 (pyrazole C_5_), 135.14, 133.18, 131.71, 131.53, 131.05, 130.45, 130.19, 129.47, 129.41, 129.26, 129.05, 128.71, 128.02, 122.36, 119.45 (CO-*C* = C), 119.15, 105.77 (pyrazole C_4_), 37.27 (CH_2_); Elemental analysis for C_34_H_25_N_5_O_3_S (583.67) (Calcd./Found): C, 69.97/69.85; H, 4.32/4.15; N, 12.00/12.23; HPLC analysis: Mobile phase (dipotassium hydrogen phosphate buffer [pH 3.2]: ACN, 30:70 respectively) FR 1mL /min, retention time = 6.48 min; peak area, 97.8% at λ_max_ 254 nm.

##### (*E*)-2-((5-(1,3-Diphenyl-1*H*-pyrazol-4-yl)-1,3,4-oxadiazol-2-yl)thio)-N-(4-(3-(4-methoxyphenyl)acryloyl)phenyl)acetamide (12b)

Yellow crystals (0.206 g, 69% yield); m.p. 230–232 °C; FT-IR; ν_max_ 3312 (NH), 1647 (Ketone CO), 1597 (amide CO); ^1^H NMR (400 MHz, DMSO-*d*_*6*_) δ (ppm): 10.77 (s, 1H, CO-NH), 9.22 (s, 1H, pyrazole C_5_–H), 8.14 (d, 2 H, *J* = 8.00 Hz, Ar–H), 7.96 (d, 2 H,, *J* = 8.00 Hz, Ar-H), 7.91–7.89 (m, 2 H, Ar-H), 7.81 (d, 2 H, *J* = 8.00 Hz Ar-H), 7.79–7.75 (m, 3 H, Ar-H), 7.68 (d, 1H, C**H** = CH *J* = 16.00 Hz), 7.56 (m, 2 H, Ar-H), 7.49–7.43 (m, 3 H, Ar-H), 7.40 (m, 1H, Ar-H), 7.01 (d, 2 H, *J* = 8.00 Hz, Ar–H), 4.34 (s, 2 H, CH_2_), 3.81 (s, 3 H, OCH_3_); ^13^C-NMR (100 MHz, DMSO-*d*_*6*_) δ (ppm): 187.97 (ketone C = O), 165.98 (amide C = O), 162.86 (oxadiazole C_2_), 161.78, 161.03 (oxadiazole C_5_), 150.94 (pyrazole C_3_), 143.98 (Ar-*C* = C), 143.25, 139.09 (pyrazole C_5_), 133.47, 131.75, 131.59, 131.17, 130.30, 130.18, 129.45, 129.06, 128.70, 127.99, 127.84, 119.87 (CO-*C* = C), 119.45, 119.09, 114.89, 105.79 (pyrazole C_4_), 55.85 (OCH_3_), 37.34 (CH_2_); Elemental analysis for C_35_H_27_N_5_O_5_S (613.69) (Calcd./Found): C, 68.50/68.61; H, 4.43/4.68; N, 11.41/11.68; HPLC analysis: Mobile phase (dipotassium hydrogen phosphate buffer [pH 3.2]: ACN, 30:70 respectively) FR 1mL /min, retention time = 6.24 min; peak area, 96.8% at λ_max_ 254 nm.

##### (*E*)-N-(4-(3-(3,4-Dimethoxyphenyl)acryloyl)phenyl)-2-((5-(1,3-diphenyl-1*H*-pyrazol-4-yl)-1,3,4-oxadiazol-2-yl)thio)acetamide (12c)

Yellow crystals (0.196 g, 61% yield); m.p. 185–187 °C; FT-IR; ν_max_ 3319 (NH), 1656 (Ketone CO), 1597 (amide CO); ^1^H NMR (400 MHz, DMSO-*d*_*6*_) δ (ppm): 10.73 (s, 1H, CO-NH), 9.22 (s, 1H, pyrazole C_5_–H), 8.19–8.17 (m, 3 H, Ar–H), 8.00-7.91 (m, 2 H, Ar-H), 7.85–7.76 (m, 6 H, Ar-H), 7.60–7.37 (m, 7 H, Ar-H), 7.04–7.02 (m, 1H, Ar-H), 4.34 (s, 2 H, CH_2_), 3.87 (s, 3 H, OCH_3_), 3.83 (s, 3 H, OCH_3_); ^13^C-NMR (100 MHz, DMSO-*d*_*6*_) δ (ppm): 187.97 (ketone C = O), 166.26, 165.94 (amide C = O), 165.34, 162.85 (oxadiazole C_2_), 161.04 (oxadiazole C_5_), 151.75, 150.95 (pyrazole C_3_), 149.56, 144.44 (Ar-*C* = C), 143.26, 139.13 (pyrazole C_5_), 133.52, 131.62, 130.29, 130.15, 129.42, 129.05, 128.69, 128.11, 127.97, 124.25, 120.05, 119.49 (CO-*C* = C), 119.08, 112.16, 111.38 (pyrazole C_4_), 56.28 (OCH_3_), 56.12 (OCH_3_), 37.40 (CH_2_); Elemental analysis for C_36_H_29_N_5_O_5_S (643.72) (Calcd./Found): C, 67.17/67.61; H, 4.54/4.62; N, 10.88/11.07; HPLC analysis: Mobile phase (dipotassium hydrogen phosphate buffer [pH 3.2]: ACN, 30:70 respectively) FR 1mL /min, retention time = 4.9 min; peak area, 96.9% at λ_max_ 254 nm.

##### (*E*)-2-((5-(1,3-Diphenyl-1*H*-pyrazol-4-yl)-1,3,4-oxadiazol-2-yl)thio)-*N*-(4-(3-(3,4,5-trimethoxyphenyl)acryloyl)phenyl)acetamide (12d).

Pale yellow crystals (0.206 g, 70% yield); m.p. 110–112 °C; FT-IR; ν_max_ 3317 (NH), 1661 (Ketone CO), 1597 (amide CO); ^1^H NMR (400 MHz, DMSO-*d*_*6*_) δ (ppm): 10.82 (s, 1H, CO-NH), 9.26 (s, 1H, pyrazole C_5_–H), 8.18 (d, 2 H, *J* = 8.00 Hz, Ar–H), 7.98 (d, 2 H, *J* = 8.00 Hz, Ar-H), 7.91–7.89 (m, 2 H, Ar-H), 7.86 (s, 1H, Ar-H), 7.79 (d, 2 H, *J* = 8.00 Hz, Ar-H), 7.68 (d, 1H, CH = C, *J* = 16.00 Hz), 7.59–7.54 (m, 2 H, Ar-H), 7.50–7.45 (m, 3 H, Ar-H), 7.42 (m, 1H, Ar–H), 7.22 (s, 2 H, Ar-H), 4.35 (s, 2 H, CH_2_), 3.87 (s, 6 H, OCH_3_), 3.72 (s, 3 H, OCH_3_); ^13^C-NMR (100 MHz, DMSO-*d*_*6*_) δ (ppm): 188.02 (ketone C = O), 166.01 (amide C = O), 162.87 (oxadiazole C_2_), 161.03 (oxadiazole C_5_), 153.58, 150.94 (pyrazole C_3_), 144.50 (Ar-*C* = C), 143.41, 140.18, 139.09 (pyrazole C_5_), 133.28, 131.75, 131.60, 130.76, 130.45, 130.18, 129.46, 129.06, 128.71, 128.00, 121.57, 119.46 (CO-*C* = C), 119.07, 106.96, 105.79 (pyrazole C_4_), 60.62 (OCH_3_), 56.62 (OCH_3_), 37.34 (CH_2_); Elemental analysis for C_37_H_31_N_5_O_6_S (673.74) (Calcd./Found): C, 65.96/65.82; H, 4.64/4.68; N, 10.39/10.61; HPLC analysis: Mobile phase (dipotassium hydrogen phosphate buffer [pH 3.2]: ACN, 30:70 respectively) FR 1mL /min, retention time = 4.9 min; peak area, 96.9% at λ_max_ 254 nm.

##### (*E*)-2-((5-(1,3-Diphenyl-1*H*-pyrazol-4-yl)-1,3,4-oxadiazol-2-yl)thio)-*N*-(4-(3-(4-fluorophenyl)acryloyl)phenyl)acetamide (12e)

Light grey crystals (0.159 g, 53% yield); m.p. 207–209 °C; FT-IR; ν_max_ **33**41 (NH), 1651 (Ketone CO), 1606 (amide CO); ^1^H NMR (400 MHz, DMSO-*d*_*6*_) δ (ppm): 10.82 (s, 1H, CO-NH), 9.28 (s, 1H, pyrazole C_5_–H), 8.19–8.17 (m, 2 H, Ar–H), 8.00-7.89 (m, 7 H, Ar-H), 7.84–7.72 (m, 4 H, Ar-H), 7.59–7.56 (m, 2 H, Ar-H), 7.49–7.42 (m, 3 H, Ar-H), 7.34–7.29 (m, 2 H, Ar–H), 4.37 (s, 2 H, CH_2_); ^13^C-NMR (100 MHz, DMSO-*d*_*6*_) δ (ppm): 187.97 (ketone C = O), 166.00 (amide C = O), 162.86 (oxadiazole C_2_), 161.03 (oxadiazole C_5_), 150.93 (pyrazole C_3_), 143.48 (Ar-*C* = C), 142.72, 139.11 (pyrazole C_5_), 133.16, 131.94, 131.77, 131.68, 130.47, 130.17, 129.44, 129.07, 128.70, 127.99, 127.98, 122.35, 119.46 (CO-*C* = C), 119.08, 116.50, 116.29, 105.80 (pyrazole C_4_), 37.37 (CH_2_); Elemental analysis for C_34_H_24_FN_5_O_3_S (601.66) (Calcd./Found): C, 67.87/68.05; H, 4.02/4.19; N, 11.64/11.87; HPLC analysis: Mobile phase (dipotassium hydrogen phosphate buffer [pH 3.2]: ACN, 30:70 respectively) FR 1mL /min, retention time = 7.1 min; peak area, 95.3% at λ_max_ 254 nm.

##### (*E*)-*N*-(4-(3-(4-Bromophenyl)acryloyl)phenyl)-2-((5-(1,3-diphenyl-1*H*-pyrazol-4-yl)-1,3,4-oxadiazol-2-yl)thio)acetamide (12f)

White crystals (0.208 g, 68% yield); m.p. 194–196 °C; FT-IR; ν_max_ **331**4 (NH), 1652 (Ketone CO), 1601 (amide CO); ^1^H NMR (400 MHz, DMSO-*d*_*6*_) δ (ppm): 11.34 (s, 1H, CO-NH), 9.38 (s, 1H, pyrazole C_5_–H), 8.15 (d, 2 H, *J* = 8.00 Hz, Ar–H), 8.03–7.98 (d, 2 H, *J* = 8.00 Hz, Ar-H), 7.94–7.92 (m, 3 H, Ar-H), 7.87–7.83 (m, 4 H, Ar-H), 7.69–7.64 (m, 3 H, Ar-H), 7.59–7.56 (m, 2 H, Ar-H), 7.50–7.44 (m, 3 H, Ar-H), 7.44–7.39 (m, 1H, Ar-H), 4.44 (s, 2 H, CH_2_); ^13^C-NMR (100 MHz, DMSO-*d*_*6*_) δ (ppm): 187.87 (ketone C = O), 166.09 (amide C = O), 164.24, 162.84 (oxadiazole C_2_), 160.97 (oxadiazole C_5_), 149.93 (pyrazole C_3_), 148.48, 143.75 (Ar-*C* = C), 141.69, 141.11, 139.01 (pyrazole C_5_), 132.78, 131.57, 131.38, 131.30, 130.68, 130.24, 130.18, 128.04, 126.44, 124.40, 119.45 (CO-*C* = C), 119.14, 115.75, 115.53, 105.76 (pyrazole C_4_), 37.32 (CH_2_); Elemental analysis for C_34_H_24_BrN_5_O_3_S (662.56) (Calcd./Found): C, 61.64/61.85; H, 3.65/3.73; N, 10.57/10.84; HPLC analysis: Mobile phase (dipotassium hydrogen phosphate buffer [pH 3.2]: ACN, 30:70 respectively) FR 1mL /min, retention time = 5.3 min; peak area, 97.2% at λ_max_ 254 nm.

##### (*E*)-2-((5-(1,3-Diphenyl-1*H*-pyrazol-4-yl)-1,3,4-oxadiazol-2-yl)thio)-*N*-(4-(3-(4-nitrophenyl)acryloyl)phenyl)acetamide (12 g)

Orange crystals (0.185 g, 59% yield); m.p. 190–192 °C; FT-IR; ν_max_ 3293 (NH), 1657 (Ketone CO), 1598 (amide CO); ^1^H NMR (400 MHz, DMSO-*d*_*6*_) δ (ppm): 10.97 (s, 1H, CO-NH), 9.22 (s, 1H, pyrazole C_5_–H), 8.29 (d, 2 H, *J* = 8.00 Hz, Ar–H), 8.21–8.16 (m, 3 H, Ar-H), 8.15–8.10 (m, 2 H, Ar-H), 7.99 (d, 2 H, *J* = 8.00 Hz, Ar-H), 7.92–7.90 (m, 2 H, Ar-H), 7.83–7.78 (m, 3 H, Ar-H), 7.59–7.56 (m, 2 H, Ar-H), 7.55–7.45 (m, 3 H, Ar–H), 7.44–7.39 (m, 1H, Ar-H), 4.38 (s, 2 H, CH_2_); ^13^C-NMR (100 MHz, DMSO-*d*_*6*_) δ (ppm): 187.82 (ketone C = O), 166.11 (amide C = O), 162.83 (oxadiazole C_2_), 161.03 (oxadiazole C_5_), 150.90 (pyrazole C_3_), 148.49, 143.84 (Ar-*C* = C), 141.75, 141.07, 139.10 (pyrazole C_5_), 132.76, 131.77, 131.65, 130.68, 130.26, 130.16, 129.43, 129.07, 128.68, 127.96, 126.49, 124.39, 119.43 (CO-*C* = C), 119.11, 105.79 (pyrazole C_4_), 37.36 (CH_2_); Elemental analysis for C_34_H_24_N_6_O_5_S (628.66) (Calcd./Found): C, 64.96/65.12; H, 3.85/3.94; N, 13.37/13.60; HPLC analysis: Mobile phase (dipotassium hydrogen phosphate buffer [pH 3.2]: ACN, 30:70 respectively) FR 1mL /min, retention time = 6.6 min; peak area, 95.7% at λ_max_ 254 nm.

##### (*E*)-2-((5-(13-Diphenyl-1*H*-pyrazol-4-yl)-1,3,4-oxadiazol-2-yl)thio)-*N*-(4-(3-(p-tolyl)acryloyl)phenyl)acetamide (12 h)

Yellow crystals (0.182 g, 61% yield); m.p. 197–199 °C; FT-IR; ν_max_ 3317 (NH), 1650 (Ketone CO), 1599 (amide CO); ^1^H NMR (400 MHz, DMSO-*d*_*6*_) δ (ppm): 10.85 (s, 1H, CO-NH), 9.27 (s, 1H, pyrazole C_5_–H), 8.15 (d, 2 H, *J* = 8.00 Hz, Ar–H), 7.98 (d, 2 H, *J* = 8.00 Hz, Ar–H), 7.92–7.85 (m, 3 H, Ar-H), 7.79–7.75 (m, 4 H,, Ar-H), 7.69 (d, 1H, C**H** = CH *J* = 16.00 Hz), 7.59–7.55(m, 2 H, Ar-H), 7.50–7.45 (m, 3 H, Ar-H), 7.44–7.39 (m, 1H, Ar-H), 7.28 (d, 2 H, *J* = 8.00 Hz, Ar–H), 4.36 (s, 2 H, CH_2_), 2.35 (s, 3 H, CH_3_); ^13^C-NMR (100 MHz, DMSO-*d*_*6*_) δ (ppm): 188.08 (ketone C = O), 166.02 (amide C = O), 162.85 (oxadiazole C_2_), 161.02 (oxadiazole C_5_), 150.94 (pyrazole C_3_), 144.08, 143.38 (Ar-*C* = C), 141.11, 139.08 (pyrazole C_5_), 133.28, 132.47, 131.74, 131.60, 130.38, 130.18, 130.03, 129.46, 129.31, 129.06, 128.71, 128.00, 121.32, 119.46 (CO-*C* = C), 119.11, 105.79 (pyrazole C_4_), 37.30 (CH_2_), 21.54 (CH_3_); Elemental analysis for C_35_H_27_N_5_O_3_S (597.69) (Calcd./Found): C, 70.33/70.19; H, 4.55/4.67; N, 11.72/11.98; HPLC analysis: Mobile phase (dipotassium hydrogen phosphate buffer [pH 3.2]: ACN, 30:70 respectively) FR 1mL /min, retention time = 8.2 min; peak area, 96.8% at λ_max_ 254 nm.

##### (*E*)-2-((5-(1,3-Diphenyl-1*H*-pyrazol-4-yl)-1,3,4-oxadiazol-2-yl)thio)-*N*-(4-(3-(naphthalen-1-yl)acryloyl)phenyl)acetamide (12i)

Yellow crystals (0.260 g, 82% yield); m.p. 113–114 °C; FT-IR; ν_max_ 3313 (NH), 1651 (Ketone CO), 1604 (amide CO); ^1^H NMR (400 MHz, DMSO-*d*_*6*_) δ (ppm): 10.86 (s, 1H, CO-NH), 9.22 (s, 1H, pyrazole C_5_–H), 8.55 (d, 1H, *J* = 16.00 Hz, Ar–H), 8.28 (d, 1H, *J* = 8.00 Hz, Ar–H), 8.24–8.10 (m, 3 H, Ar-H), 7.96–8.07 (m, 6 H, Ar-H), 7.90 (d, 2 H, *J* = 8.00 Hz), 7.81 (d, 2 H, *J* = 8.00 Hz, Ar-H), 7.68–7.55 (m, 5 H, Ar-H), 7.50–7.45 (m, 2 H, Ar-H), 7.42–7.39 (m, 1H, Ar-H), 4.36 (s, 2 H, CH_2_); ^13^C-NMR (100 MHz, DMSO-*d*_*6*_) δ (ppm): 188.05 (ketone C = O), 166.06 (amide C = O), 162.85 (oxadiazole C_2_), 161.03 (oxadiazole C_5_), 150.94 (pyrazole C_3_), 143.54 (Ar-*C* = C), 139.92 (pyrazole C_5_), 139.08, 133.84, 133.14, 131.84, 131.74, 131.66, 131.59, 131.24, 130.55, 130.18, 129.46, 129.27, 129.06, 128.70, 127.99, 127.73, 126.80, 126.19, 126.10, 124.91, 123.44, 119.45 (CO-*C* = C), 119.17, 105.79 (pyrazole C_4_), 37.33 (CH_2_); Elemental analysis for C_38_H_27_N_5_O_3_S (633.73) (Calcd./Found): C, 72.02/72.25; H, 4.29/4.37; N, 11.05/11.26; HPLC analysis: Mobile phase (dipotassium hydrogen phosphate buffer [pH 3.2]: ACN, 30:70 respectively) FR 1mL /min, retention time = 11.1 min; peak area, 96.7% at λ_max_ 254.

##### *N*-(4-Cinnamoylphenyl)-2-((5-(3-(4-fluorophenyl)-1-phenyl-1*H*-pyrazol-4-yl)-1,3,4-oxadiazol-2-yl)thio)acetamide (13a)

White crystals (0.234 g, 78% yield); m.p. 240–242 °C; FT-IR; ν_max_ 3312 (NH), 1656 (Ketone CO), 1598 (amide CO); ^1^H NMR (400 MHz, DMSO-*d*_*6*_) δ (ppm): 10.82 (s, 1H, CO-N**H**), 9.22 (s, 1H, pyrazole C_5_–H), 8.16 (d, 2 H, *J* = 8.00 Hz, Ar–H), 7.99–7.93 (m, 4 H, Ar-H), 7.89–7.86 (m, 3 H, Ar-H), 7.77 (d, 2 H, *J* = 8.00 Hz, Ar–H) 7.72 (d, 1H, C**H** = CH *J* = 16.00 Hz), 7.58–7.52 (m, 2 H, Ar-H), 7.47–7.46 (m, 3 H, Ar-H), 7.42–7.38 (m, 1H, Ar-H), 7.31 (m, 2 H, Ar-H), 4.34 (s, 2 H, C**H**_**2**_); ^13^C-NMR (100 MHz, DMSO-*d*_*6*_) δ (ppm): 188.15 (ketone C = O), 166.05 (amide C = O), 162.86 (oxadiazole C_2_), 160.97 (oxadiazole C_5_), 149.96 (pyrazole C_3_), 144.04, 143.43 (Ar-*C* = C), 139.01 (pyrazole C_5_), 135.16, 133.18, 131.57, 131.39, 131.31, 131.05, 130.45, 130.20, 129.42, 129.26, 128.06, 122.38, 119.47 (CO-*C* = C), 119.14, 115.77, 115.55, 105.77 (pyrazole C_4_), 37.29 (CH_2_); Elemental analysis for C_34_H_24_FN_5_O_3_S (601.66) (Calcd./Found): C, 67.87/67.91; H, 4.02/4.05; N, 11.64/11.67; HPLC analysis: Mobile phase (dipotassium hydrogen phosphate buffer [pH 3.2]: ACN, 30:70 respectively) FR 1mL /min, retention time = 7.96 min; peak area, 96.6% at λ_max_ 254.

##### (*E*)-2-((5-(3-(4-Fluorophenyl)-1-phenyl-1*H*-pyrazol-4-yl)-1,3,4-oxadiazol-2-yl)thio)-*N*-(4-(3-(4-methoxyphenyl)acryloyl)phenyl)acetamide (13b)

white crystals (0.205 g, 65% yield); m.p. 220–222 °C; FT-IR; ν_max_ 3277 (NH), 1659 (Ketone CO), 1599 (amide CO); ^1^H NMR (400 MHz, DMSO-*d*_*6*_) δ (ppm): 10.97 (s, 1H, CO-N**H**), 9.29 (s, 1H, pyrazole C_5_–H), 8.14 (d, 2 H, *J* = 8.00 Hz, Ar–H), 8.01–7.97 (m, 4 H, Ar-H), 7.85–7.76 (m, 5 H, Ar-H), 7.69 (d, 1H, C**H** = CH *J* = 16.00 Hz), 7.77–7.55 (m, 2 H, Ar-H), 7.41 (m, 1H, Ar-H), 7.34–7.10 (m, 2 H, Ar-H), 7.02 (d, 2 H, *J* = 8.00 Hz, Ar–H), 4.38 (s, 2 H, C**H**_**2**_), 3.82 (s, 3 H, OCH_3_); ^13^C-NMR (100 MHz, DMSO-*d*_*6*_) δ (ppm): 187.95 (ketone C = O), 166 (amide C = O), 162.83 (oxadiazole C_2_), 161.77 (oxadiazole C_5_), 160.96, 149.91 (pyrazole C_3_), 143.96 (Ar-*C* = C), 143.31, 139.04 (pyrazole C_5_), 133.43, 131.67, 131.39, 131.31, 131.17, 130.27, 130.18, 128.24, 128.21, 128.02, 127.85, 119.89, 119.45 (CO-*C* = C), 119.07, 115.76, 115.54, 114.89, 105.79 (pyrazole C_4_), 55.85 (OCH_3_), 37.29 (CH_2_); Elemental analysis for C_35_H_26_FN_5_O_4_S (631.68) (Calcd./Found): C, 66.55/66.58; H, 4.15/4.18; N, 11.09/11.12; HPLC analysis: Mobile phase (dipotassium hydrogen phosphate buffer [pH 3.2]: ACN, 30:70 respectively) FR 1mL /min, retention time = 7.12 min; peak area, 96.3% at λ_max_ 254.

##### (*E*)-*N*-(4-(3-(3,4-dimethoxyphenyl)acryloyl)phenyl)-2-((5-(3-(4-fluorophenyl)-1-phenyl-1*H*-pyrazol-4-yl)-1,3,4-oxadiazol-2-yl)thio)acetamide (13c)

Pale yellow crystals (0.244 g, 74% yield); m.p. 140–142 °C; FT-IR; ν_max_ 3298 (NH), 1656 (Ketone CO), 1598 (amide CO); ^1^H NMR (400 MHz, DMSO-*d*_*6*_) δ (ppm): 10.84 (s, 1H, CO-N**H**), 9.26 (s, 1H, pyrazole C_5_–H), 8.17 (d, 2 H, *J* = 8.00 Hz, Ar–H), 8.00-7.96 (m, 4 H, Ar-H), 7.83–7.78 (m, 3 H, Ar-H), 7.69 (d, 1H, C**H** = CH *J* = 16.00 Hz), 7.59–7.53 (m, 2 H, Ar-H), 7.528–7.520 (m, 1H, Ar-H), 7.43–7.39 (m, 1H, Ar-H), 7.38–7.36 (m, 1H, Ar-H), 7.33–7.29 (m, 2 H, Ar-H), 7.02 (d, 1H, *J* = 8.00 Hz, Ar–H), 4.36 (s, 2 H, C**H**_**2**_), 3.86 (s, 3 H, OCH_3_), 3.82 (s, 3 H, OCH_3_); ^13^C-NMR (100 MHz, DMSO-*d*_*6*_) δ (ppm): 187.97 (ketone C = O), 166.00 (amide C = O), 164.24, 162.86 (oxadiazole C_2_), 161.79 (oxadiazole C_5_), 160.97, 151.69, 149.93 (pyrazole C_3_), 149.49, 144.52, 143.26 (Ar-*C* = C), 139.02 (pyrazole C_5_), 133.47, 131.59, 131.39, 131.30, 130.33, 130.17, 128.20, 128.03, 124.34, 119.91 (CO-*C* = C), 119.45, 119.06, 115.75, 115.53, 112.04, 111.16, 105.77 (pyrazole C_4_), 56.21 (OCH_3_), 56.06 (OCH_3_), 37.32 (CH_2_); Elemental analysis for C_36_H_28_FN_5_O_5_S (661.71) (Calcd./Found): C, 65.34/65.39; H, 4.27/4.30; N, 10.58/10.63; HPLC analysis: Mobile phase (dipotassium hydrogen phosphate buffer [pH 3.2]: ACN, 30:70 respectively) FR 1mL /min, retention time = 5.31 min; peak area, 97.6% at λ_max_ 254.

##### (*E*)-2-((5-(3-(4-fluorophenyl)-1-phenyl-1*H*-pyrazol-4-yl)-1,3,4-oxadiazol-2-yl)thio)-*N*-(4-(3-(3,4,5-trimethoxyphenyl)acryloyl)phenyl)acetamide (13d)

Pale yellow crystals (0.200 g, 58% yield); m.p. 145–147 °C; FT-IR; ν_max_ 3301 (NH), 1656 (Ketone CO), 1597 (amide CO); ^1^H NMR (400 MHz, DMSO-*d*_*6*_) δ (ppm): 11.46 (s, 1H, CO-N**H**), 9.40 (s, 1H, pyrazole C_5_–H), 8.16 (d, 2 H, *J* = 8.00 Hz, Ar–H), 8.03–7.99 (m, 4 H, Ar-H), 7.86–7.93 (m, 3 H, Ar-H), 7.67 (d, 1H, C**H** = CH, *J* = 16.00 Hz), 7.59–7.55 (m, 2 H, Ar-H), 7.43–7.41 (m, 1H, Ar–H), 7.34–7.29 (m, 2 H, Ar–H), 7.25–7.21 (m, 2 H, Ar-H), 4.35 (s, 2 H, C**H**_**2**_), 3.87 (s, 6 H, OCH_3_), 3.72 (s, 3 H, OCH_3_); ^13^C-NMR (100 MHz, DMSO-*d*_*6*_) δ (ppm): 188.04 (ketone C = O), 166.28 (amide C = O), 162.75 (oxadiazole C_2_), 161.78 (oxadiazole C_5_), 160.95, 153.57, 149.81 (pyrazole C_3_), 144.42, 143.67 (Ar-*C* = C), 140.15, 139.03 (pyrazole C_5_), 133.16, 131.85, 131.40, 131.31, 130.89, 130.79, 130.36, 130.15, 127.97, 121.63, 119.41 (CO-*C* = C), 119.23, 119.06, 115.74, 115.52, 107.15, 106.97, 105.77 (pyrazole C_4_), 60.62 (OCH_3_), 56.64 (OCH_3_), 37.15 (CH_2_); Elemental analysis for C_37_H_30_FN_5_O_6_S (691.73) (Calcd./Found): C, 64.25/64.28; H, 4.37/4.39; N, 10.12/10.14; HPLC analysis: Mobile phase (dipotassium hydrogen phosphate buffer [pH 3.2]: ACN, 30:70 respectively) FR 1mL /min, retention time = 6.37 min; peak area, 97.4% at λ_max_ 254.

##### (*E*)-2-((5-(3-(4-fluorophenyl)-1-phenyl-1*H*-pyrazol-4-yl)-1,3,4-oxadiazol-2-yl)thio)-*N*-(4-(3-(4-fluorophenyl)acryloyl)phenyl)acetamide (13e)

Gray crystals (0.189 g, 61% yield); m.p. 240–243 °C; FT-IR; ν_max_ 3315 (NH), 1654 (Ketone CO), 1602 (amide CO); ^1^H NMR (400 MHz, DMSO-*d*_*6*_) δ (ppm): 10.82 (s, 1H, CO-N**H**), 9.27 (s, 1H, pyrazole C_5_–H), 8.17 (d, 2 H, *J* = 8.00 Hz, Ar–H), 8.01–7.95 (m, 6 H, Ar-H), 7.89 (d, 1H, C**H** = CH, *J* = 16.00 Hz), 7.78 (d, 2 H, *J* = 8.00 Hz, Ar-H), 7.72 (d, 1H, C**H** = CH, *J* = 16.00 Hz), 7.59–7.55 (m, 2 H, Ar-H), 7.43–7.41 (m, 1H, Ar-H), 7.34–7.28 (m, 4 H, Ar-H), 4.36 (s, 2 H, C**H**_**2**_); ^13^C-NMR (100 MHz, DMSO-*d*_*6*_) δ (ppm): 187.98 (ketone C = O), 166.02 (amide C = O), 162.85 (oxadiazole C_2_), 162.59, 161.79 (oxadiazole C_5_), 160.98, 149.92 (pyrazole C_3_), 143.47 (Ar-*C* = C), 142.73, 139.04 (pyrazole C_5_), 133.16, 131.92, 131.66, 131.61, 131.58, 131.39, 131.30, 130.46, 130.17, 128.25, 128.02, 122.32, 119.45 (CO-*C* = C), 119.08, 116.50, 116.28, 115.75, 115.54, 105.78 (pyrazole C_4_), 37.36 (CH_2_); Elemental analysis for C_34_H_23_F_2_N_5_O_3_S (619.65) (Calcd./Found): C, 65.90/65.92; H, 3.74/3.77; N, 11.30/11.31; HPLC analysis: Mobile phase (dipotassium hydrogen phosphate buffer [pH 3.2]: ACN, 30:70 respectively) FR 1mL /min, retention time = 6.59 min; peak area, 98.0% at λ_max_ 254.

##### (*E*)-*N*-(4-(3-(4-bromophenyl)acryloyl)phenyl)-2-((5-(3-(4-fluorophenyl)-1-phenyl-1*H*-pyrazol-4-yl)-1,3,4-oxadiazol-2-yl)thio)acetamide (13f)

White crystals (0.241 g, 71% yield); m.p. 210–212 °C; FT-IR; ν_max_ 3316 (NH), 1661 (Ketone CO), 1596 (amide CO); ^1^H NMR (400 MHz, DMSO-*d*_*6*_) δ (ppm): 11.43 (s, 1H, CO-N**H**), 9.27 (s, 1H, pyrazole C_5_–H), 8.14 (d, 2 H, *J* = 8.00 Hz, Ar–H), 8.03–7.93 (m, 4 H, Ar-H), 7.89–7.83 (m, 4 H, Ar-H), 7.69–7.64 (m, 3 H, Ar-H), 7.57–7.54 (m, 3 H, Ar-H), 7.43–7.41 (m, 1H, Ar-H), 7.33–7.29 (m, 2 H, Ar-H), 4.47 (s, 2 H, C**H**_**2**_); ^13^C-NMR (100 MHz, DMSO-*d*_*6*_) δ (ppm): 187.99 (ketone C = O), 166.33 (amide C = O), 164.21, 162.73 (oxadiazole C_2_), 160.94 (oxadiazole C_5_), 149.81 (pyrazole C_3_), 142.53 (Ar-*C* = C), 138.99 (pyrazole C_5_), 134.47, 132.92, 132.34, 131.82, 131.39, 131.30, 131.19, 130.41, 130.17, 128.19, 128.00, 124.30, 123.22, 119.40 (CO-*C* = C), 119.10, 115.74, 115.52, 105.75 (pyrazole C_4_), 37.14 (CH_2_); Elemental analysis for C_34_H_23_BrFN_5_O_3_S (680.55) (Calcd./Found): C, 60.01/60.03; H, 3.41/3.41; N, 10.29/10.31; HPLC analysis: Mobile phase (dipotassium hydrogen phosphate buffer [pH 3.2]: ACN, 30:70 respectively) FR 1mL /min, retention time = 6.21 min; peak area, 97.7% at λ_max_ 254.

##### (*E*)-2-((5-(3-(4-Fluorophenyl)-1-phenyl-1*H*-pyrazol-4-yl)-1,3,4-oxadiazol-2-yl)thio)-*N*-(4-(3-(4-nitrophenyl)acryloyl)phenyl)acetamide (13g)

Pale yellow crystals (0.210 g, 65% yield); m.p. 210–212 °C; FT-IR; ν_max_ 3310 (NH), 1654 (Ketone CO), 1600 (amide CO); ^1^H NMR (400 MHz, DMSO-*d*_*6*_) δ (ppm): 10.85 (s, 1H, CO-N**H**), 9.26 (s, 1H, pyrazole C_5_–H), 8.28 (d, 2 H, *J* = 8.00 Hz, Ar–H), 8.20–8.14 (m, 4 H, Ar-H), 8.12 (d, 1H, C**H** = CH, *J* = 16.00 Hz), 8.00-7.96 (m, 4 H, Ar-H), 7.81–7.77 (m, 3 H, Ar-H), 7.58–7.55 (m, 2 H, Ar-H), 7.41 (m, 1H Ar-H), 7.33–7.29 (m, 2 H, Ar-H), 4.36 (s, 2 H, C**H**_**2**_); ^13^C-NMR (100 MHz, DMSO-*d*_*6*_) δ (ppm): 187.87 (ketone C = O), 166.09 (amide C = O), 164.24, 162.84 (oxadiazole C_2_), 161.79 (oxadiazole C_5_), 160.97, 149.93 (pyrazole C_3_), 148.48, 143.75 (Ar-*C* = C), 141.69, 141.11, 139.01 (pyrazole C_5_), 131.57, 131.38, 131.30, 130.68, 130.24, 130.18, 128.04, 126.44, 124.40, 119.45 (CO-*C* = C), 119.14, 115.75, 115.53, 105.76 (pyrazole C_4_), 37.32 (CH_2_); Elemental analysis for C_34_H_23_FN_6_O_5_S (680.55) (Calcd./Found): C, 63.15/63.18; H, 3.59/3.560; N, 13.00/13.02; HPLC analysis: Mobile phase (dipotassium hydrogen phosphate buffer [pH 3.2]: ACN, 30:70 respectively) FR 1mL /min, retention time = 6.47 min; peak area, 94.2% at λ_max_ 254.

##### (*E*)-2-((5-(3-(4-Fluorophenyl)-1-phenyl-1*H*-pyrazol-4-yl)-1,3,4-oxadiazol-2-yl)thio)-*N*-(4-(3-(p-tolyl)acryloyl)phenyl)acetamide (13h)

Orange crystals (0.175 g, 57% yield); m.p. 230–232 °C; FT-IR; ν_max_ 3328 (NH), 1658 (Ketone CO), 1598 (amide CO); ^1^H NMR (400 MHz, DMSO-*d*_*6*_) δ (ppm): 10.88 (s, 1H, CO-N**H**), 9.30 (s, 1H, pyrazole C_5_–H), 8.16 (d, 2 H, *J* = 8.00 Hz, Ar–H), 8.02–7.98 (m, 4 H, Ar-H), 7.88 (d, 1H, C**H** = C, *J* = 16.00 Hz), 8.00-7.96 (m, 4 H, Ar-H), 7.70 (d, 1H, C**H** = CH, *J* = 16.00 Hz), 7.59–7.55 (m, 2 H, Ar-H), 7.43–7.41 (m, 1H, Ar-H), 7.34–7.27 (m, 4 H, Ar-H), 4.38 (s, 2 H, C**H**_**2**_), 2.36 (s, 3 H, C**H**_**3**_); ^13^C-NMR (100 MHz, DMSO-*d*_*6*_) δ (ppm): δ 188.03 (ketone C = O), 166.02 (amide C = O), 162.84 (oxadiazole C_2_), 160.97 (oxadiazole C_5_), 149.92 (pyrazole C_3_), 144.04, 143.40 (Ar-*C* = C), 141.07, 139.05 (pyrazole C_5_), 133.28, 132.50, 131.65, 131.39, 131.31, 130.38, 130.17, 130.02, 129.32, 128.02, 121.34, 119.46 (CO-*C* = C), 119.08, 115.76, 115.54, 105.79 (pyrazole C_4_), 37.34 (CH_2_), 21.55 (CH_3_); Elemental analysis for C_35_H_26_FN_5_O_3_S (615.68) (Calcd./Found): C, 68.28/68.30; H, 4.26/4.29; N, 11.38/11.41; HPLC analysis: Mobile phase (dipotassium hydrogen phosphate buffer [pH 3.2]: ACN, 30:70 respectively) FR 1mL /min, retention time = 8.97 min; peak area, 95.3% at λ_max_ 254.

##### (*E*)-2-((5-(3-(4-Fluorophenyl)-1-phenyl-1*H*-pyrazol-4-yl)-1,3,4-oxadiazol-2-yl)thio)-*N*-(4-(3-(naphthalen-1-yl)acryloyl)phenyl)acetamide (13i)

Yellow crystals (0.253 g, 78% yield); m.p. 110–112 °C; FT-IR; ν_max_ 3313 (NH), 1651 (Ketone CO), 1603 (amide CO); ^1^H NMR (400 MHz, DMSO-*d*_*6*_) δ (ppm): 10.93 (s, 1H, CO-N**H**), 9.29 (s, 1H, pyrazole C_5_–H), 8.55 (d, 1H, C**H** = CH, *J* = 16.00 Hz, Ar–H), 8.29 (d, 1H, *J* = 8.00 Hz, Ar–H), 8.25–8.22 (m, 3 H, Ar-H), 8.07 (d, 1H, *J* = 8.00 Hz, Ar–H), 8.02–7.98 (m, 6 H, Ar-H), 7.81 (d, 2 H, *J* = 8.00 Hz Ar-H), 7.69–7.61 (m, 3 H, Ar-H), 7.59–7.55 (m, 2 H, Ar-H), 7.41 (m, 1H, Ar-H), 7.34–7.41 (m, 2 H, Ar-H), 4.38 (s, 2 H, C**H**_**2**_); ^13^C-NMR (100 MHz, DMSO-*d*_*6*_) δ (ppm): 188.00 (ketone C = O), 166.07 (amide C = O), 164.24, 162.84 (oxadiazole C_2_), 160.98 (oxadiazole C_5_), 149.91 (pyrazole C_3_), 143.57 (Ar-*C* = C), 139.89 (pyrazole C_5_), 139.05, 133.85, 133.13, 131.88, 131.67, 131.39, 131.31, 131.22, 130.55, 130.17, 129.27, 128.01, 127.72, 126.79, 126.18, 126.10, 124.95, 123.46, 119.46 (CO-*C* = C), 119.15, 115.76, 115.55, 105.79 (pyrazole C_4_), 37.35 (CH_2_); Elemental analysis for C_38_H_26_FN_5_O_3_S (651.72) (Calcd./Found): C, 70.03/70.04; H, 4.02/4.03; N, 10.75/10.77; HPLC analysis: Mobile phase (dipotassium hydrogen phosphate buffer [pH 3.2]: ACN, 30:70 respectively) FR 1mL /min, retention time = 8.00 min; peak area, 94.7% at λ_max_ 254.

##### *N*-(4-Cinnamoylphenyl)-2-((5-(3-(4-methoxyphenyl)-1-phenyl-1*H*-pyrazol-4-yl)-1,3,4-oxadiazol-2-yl)thio)acetamide (14a)

White crystals (0.202 g, 66% yield); m.p. 185–186 °C; FT-IR; ν_max_ 3312 (NH), 1664 (Ketone CO), 1600 (amide CO); ^1^H NMR (400 MHz, DMSO-*d*_*6*_) δ (ppm): 10.83 (s, 1H, CO-NH), 9.21 (s, 1H, pyrazole C_5_–H), 8.17 (d, 2 H, *J* = 8.00 Hz, Ar–H), 7.96–7.92 (m, 3 H, Ar-H), 7.92–7.84 (m, 4 H, Ar-H), 7.79 (d, 2 H, *J* = 8.00 Hz, Ar–H), 7.73 (d, 1H, C**H** = CH *J* = 16.00 Hz Ar-H), 7.59–7.55 (m, 2 H, Ar-H), 7.50–7.43 (m, 3 H, Ar-H), 7.41–7.39 (m, 1H, Ar-H), 7.02 (d, 2 H, *J* = 8.00 Hz, Ar–H), 4.36 (s, 2 H, CH_2_), 3.81 (s, 3 H, OCH_3_); ^13^C-NMR (100 MHz, DMSO-*d*_*6*_) δ (ppm): 188.11 (ketone C = O), 166.06 (amide C = O), 162.75 (oxadiazole C_2_), 161.19 (oxadiazole C_5_), 160.33, 150.74 (pyrazole C_3_), 144.01, 143.45 (Ar-*C* = C), 139.11 (pyrazole C_5_), 135.19, 133.19, 131.47, 131.02, 130.46, 130.44, 130.15, 129.40, 129.27, 127.87, 124.09, 122.40, 119.36 (CO-*C* = C), 119.12, 114.12, 105.45 (pyrazole C_4_), 55.66 (OCH_3_), 37.33 (CH_2_); Elemental analysis for C_35_H_27_N_5_O_4_S (613.69) (Calcd./Found): C, 68.50/68.62; H, 4.43/4.46; N, 11.41/11.43; HPLC analysis: Mobile phase (dipotassium hydrogen phosphate buffer [pH 3.2]: ACN, 30:70 respectively) FR 1mL /min, retention time = 6.22 min; peak area, 96.2% at λ_max_ 254.

##### (*E*)-2-((5-(3-(4-Methoxyphenyl)-1-phenyl-1*H*-pyrazol-4-yl)-1,3,4-oxadiazol-2-yl)thio)-*N*-(4-(3-(4-methoxyphenyl)acryloyl)phenyl)acetamide (14b)

Pale yellow crystals (0.225 g, 70% yield); m.p. 118–120 °C; FT-IR; ν_max_ 3314 (NH), 1653 (Ketone CO), 1599 (amide CO); ^1^H NMR (400 MHz, DMSO-*d*_*6*_) δ (ppm): 10.81 (s, 1H, CO-N**H**), 9.23 (s, 1H, pyrazole C_5_–H), 8.15 (d, 2 H, *J* = 8.00 Hz, Ar–H), 7.97 (d, 2 H, *J* = 8.00 Hz, Ar–H), 7.89–7.77 (m, 7 H, Ar-H), 7.70 (d, 1H, C**H** = CH *J* = 16.00 Hz Ar-H), 7.58–7.55 (m, 2 H, Ar-H), 7.41 (m, 1H, Ar-H), 7.04–7.01 (m, 4 H, Ar–H), 4.36 (s, 2 H, C**H**_**2**_), 3.83 (s, 3 H, OC**H**_**3**_), 3.81 (s, 3 H, OC**H**_**3**_); ^13^C-NMR (100 MHz, DMSO-*d*_*6*_) δ (ppm): 187.93 (ketone C = O), 166.00 (amide C = O), 162.76 (oxadiazole C_2_), 161.78 (oxadiazole C_5_), 161.19, 160.33, 150.73 (pyrazole C_3_), 143.96, 143.26 (Ar-*C* = C), 139.13 (pyrazole C_5_), 133.47, 131.50, 131.17, 130.43, 130.31, 130.15, 127.87, 127.84, 124.11, 119.90 (CO-*C* = C), 119.36, 119.06, 114.88, 114.13, 105.47 (pyrazole C_4_), 55.85 (OCH_3_), 55.66 (OCH_3_), 37.37(CH_2_); Elemental analysis for C_36_H_29_N_5_O_5_S (643.72) (Calcd./Found): C, 67.17/67.18; H, 4.54/4.55; N, 10.88/10.90; HPLC analysis: Mobile phase (dipotassium hydrogen phosphate buffer [pH 3.2]: ACN, 30:70 respectively) FR 1mL /min, retention time = 5.84 min; peak area, 97.4% at λ_max_ 254.

##### (*E*)-*N*-(4-(3-(3,4-Dimethoxyphenyl)acryloyl)phenyl)-2-((5-(3-(4-methoxyphenyl)-1-phenyl-1*H*-pyrazol-4-yl)-1,3,4-oxadiazol-2-yl)thio)acetamide )14c)

Yellow crystals (0.215 g, 64% yield); m.p. 168–170 °C; FT-IR; ν_max_ 3319 (All chemicals used in the preparation NH), 1650 (Ketone CO), 1602 (amide CO); ^1^H NMR (400 MHz, DMSO-*d*_*6*_) δ (ppm): 10.91 (s, 1H, CO-NH), 9.25 (s, 1H, pyrazole C_5_–H), 8.17 (d, 2 H, *J* = 8.00 Hz, Ar–H), 7.97 (d, 2 H, *J* = 8.00 Hz, Ar–H), 7.88 (d, 2 H, *J* = 8.00 Hz, Ar–H), 7.83–7.76 (m, 3 H, Ar-H), 7.69 (d, 1H, C**H** = CH *J* = 16.00 Hz Ar-H), 7.58–7.53 (m, 3 H, Ar-H), 7.42–7.36 (m, 2 H, Ar-H), 7.04–7.01 (m, 3 H, Ar–H), 4.38 (s, 2 H, CH_2_), 3.87 (s, 3 H, OCH_3_), 3.83 (s, 3 H, OCH_3_), 3.81 (s, 3 H, OCH_3_); ^13^C-NMR (100 MHz, DMSO-*d*_*6*_) δ (ppm): 187.93 (ketone C = O), 166.05 (amide C = O), 162.75 (oxadiazole C_2_), 161.19 (oxadiazole C_5_), 160.33, 151.70, 150.72 (pyrazole C_3_), 149.51, 144.49, 143.32 (Ar-*C* = C), 139.13 (pyrazole C_5_), 133.46, 131.52, 130.44, 130.33, 130.14, 128.06, 127.84, 124.34, 124.11, 119.94 (CO-*C* = C), 119.35, 119.04, 114.12, 112.05, 111.20, 105.47 (pyrazole C_4_), 56.23 (OCH_3_), 56.08 (OCH_3_), 55.66 (OCH_3_), 37.33 (CH_2_); Elemental analysis for C_37_H_31_N_5_O_6_S (673.74) (Calcd./Found): C, 65.96/65.97; H, 4.64/4.67; N, 10.39/10.42; HPLC analysis: Mobile phase (dipotassium hydrogen phosphate buffer [pH 3.2]: ACN, 30:70 respectively) FR 1mL /min, retention time = 4.78 min; peak area, 96.2% at λ_max_ 254.

##### (*E*)-2-((5-(3-(4-Methoxyphenyl)-1-phenyl-1*H*-pyrazol-4-yl)-1,3,4-oxadiazol-2-yl)thio)-*N*-(4-(3-(3,4,5-trimethoxyphenyl)acryloyl)phenyl)acetamide (14d)

White crystals (0.225 g, 70% yield); m.p. 130–132 °C; FT-IR; ν_max_ 3315 (NH), 1650 (Ketone CO), 1605 (amide CO); ^1^H NMR (400 MHz, DMSO-*d*_*6*_) δ (ppm): 10.83 (s, 1H, CO-NH), 9.23 (s, 1H, pyrazole C_5_–H), 8.18 (d, 2 H, *J* = 8.00 Hz, Ar–H), 7.97 (d, 2 H, *J* = 8.00 Hz, Ar–H), 7.90–7.86 (m, 3 H, Ar-H), 7.81 (d, 2 H, *J* = 8.00 Hz, Ar–H), 7.68 (d, 1H, C**H** = CH *J* = 16.00 Hz Ar-H), 7.58–7.54 (m, 2 H, Ar-H), 7.43–7.41 (m, 1H, Ar-H), 7.22 (s, 2 H, Ar-H), 7.03 (d, 2 H, *J* = 8.00 Hz, Ar–H), 4.37 (s, 2 H, CH_2_), 3.87 (s, 6 H, OCH_3_), 3.81 (s, 3 H, OCH_3_), 3.72 (s, 3 H, OCH_3_); ^13^C-NMR (100 MHz, DMSO-*d*_*6*_) δ (ppm): δ 188.04 (ketone C = O), 166.07 (amide C = O), 165.35, 162.76 (oxadiazole C_2_), 161.19 (oxadiazole C_5_), 160.33, 153.58, 150.74 (pyrazole C_3_), 144.50, 143.41 (Ar-*C* = C), 140.17, 139.10 (pyrazole C_5_), 133.28, 131.48, 130.76, 130.44, 130.16, 127.87, 124.08, 121.57, 119.36 (CO-*C* = C), 119.08, 114.12, 106.95, 105.45 (pyrazole C_4_), 60.63 (OCH_3_), 56.62 (OCH_3_), 55.66 (OCH_3_), 37.30 (CH_2_); Elemental analysis for C_38_H_33_N_5_O_7_S (703.77) (Calcd./Found): C, 64.85/64.87; H, 4.73/4.75; N, 9.95/9.96; HPLC analysis: Mobile phase (dipotassium hydrogen phosphate buffer [pH 3.2]: ACN, 30:70 respectively) FR 1mL /min, retention time = 5.16 min; peak area, 95.4% at λ_max_ 254.

##### (*E*)-*N*-(4-(3-(4-Fluorophenyl)acryloyl)phenyl)-2-((5-(3-(4-methoxyphenyl)-1-phenyl-1H-pyrazol-4-yl)-1,3,4-oxadiazol-2-yl)thio)acetamide (14e)

Gray crystals (0.233 g, 74% yield); m.p. 185–186 °C; FT-IR; ν_max_ 3315 (NH), 1657 (Ketone CO), 1598 (amide CO); ^1^H NMR (400 MHz, DMSO-*d*_*6*_) δ (ppm): 11.13 (s, 1H, CO-NH), 9.29 (s, 1H, pyrazole C_5_–H), 8.15 (d, 2 H, *J* = 8.00 Hz, Ar–H), 8.01–7.92 (m, 4 H, Ar-H), 7.92–7.79 (m, 6 H, Ar-H), 7.72 (d, 1H, C**H** = CH *J* = 16.00 Hz Ar-H), 7.58–7.54 (m, 2 H,, Ar-H), 7.43–7.41 (m, 1H, Ar-H), 7.30 (m, 2 H, Ar-H), 7.03 (d, 1H, *J* = 8.00 Hz, Ar–H), 4.40 (s, 2 H, CH_2_), 3.81 (s, 3 H, OCH_3_); ^13^C-NMR (100 MHz, DMSO-*d*_*6*_) δ (ppm): 188.01 (ketone C = O), 166.19 (amide C = O), 162.70 (oxadiazole C_2_), 161.18 (oxadiazole C_5_), 160.32, 150.69 (pyrazole C_3_), 143.59 (Ar-*C* = C), 142.71, 139.11 (pyrazole C_5_), 133.10, 131.91, 131.59, 130.43, 130.14, 127.84, 124.10, 122.34, 122.32, 119.34 (CO-*C* = C), 119.09, 116.49, 116.28, 114.12, 105.46, 105.43 (pyrazole C_4_), 55.66 (OCH_3_), 37.24 (CH_2_); Elemental analysis for C_35_H_26_FN_5_O_4_S (631.68) (Calcd./Found): C, 66.55/66.56; H, 4.15/4.18; N, 11.09/11.10; HPLC analysis: Mobile phase (dipotassium hydrogen phosphate buffer [pH 3.2]: ACN, 30:70 respectively) FR 1mL /min, retention time = 6.33 min; peak area, 97.1% at λ_max_ 254.

##### (*E*)-*N*-(4-(3-(4-Bromophenyl)acryloyl)phenyl)-2-((5-(3-(4-methoxyphenyl)-1-phenyl-1*H*-pyrazol-4-yl)-1,3,4-oxadiazol-2-yl)thio)acetamide (14f)

White crystals (0.200 g, 62% yield); m.p. 185–186 °C; FT-IR; ν_max_ 3313 (NH), 1653 (Ketone CO), 1601 (amide CO); ^1^H NMR (400 MHz, DMSO-*d*_*6*_) δ (ppm): 11.42 (s, 1H, CO-NH), 9.35 (s, 1H, pyrazole C_5_–H), 8.15 (d, 2 H, *J* = 8.00 Hz, Ar–H), 8.01–7.94 (m, 3 H, Ar-H), 7.91–7.83 (m, 7 H, Ar-H), 7.70–7.64 (m, 3 H, Ar-H), 7.58–7.54 (m, 2 H, Ar-H), 7.42–7.40 (m, 1H, Ar-H), 7.03 (d, 1H, *J* = 8.00 Hz, Ar–H), 4.46 (s, 2 H, CH_2_), 3.81 (s, 3 H, OCH_3_); ^13^C-NMR (100 MHz, DMSO-*d*_*6*_) δ (ppm): 187.91 (ketone C = O), 162.66 (amide C = O), 161.16 (oxadiazole C_2_), 160.30 (oxadiazole C_5_), 150.61 (pyrazole C_3_), 143.78 (Ar-*C* = C), 142.54, 139.10 (pyrazole C_5_), 134.53, 132.91, 132.33, 131.72, 131.69, 131.22, 130.46, 130.14, 127.81, 124.33, 124.09, 123.24, 123.19, 119.30 (CO-*C* = C), 119.05, 114.10, 105.46 (pyrazole C_4_), 55.66 (OCH_3_), 37.16 (CH_2_); Elemental analysis for C_35_H_26_BrN_5_O_4_S (692.59) (Calcd./Found): C, 60.70/60.72; H, 3.78/3.78; N, 10.11/10.12; HPLC analysis: Mobile phase (dipotassium hydrogen phosphate buffer [pH 3.2]: ACN, 30:70 respectively) FR 1mL /min, retention time = 6.46 min; peak area, 95.3% at λ_max_ 254.

##### (*E*)-2-((5-(3-(4-Methoxyphenyl)-1-phenyl-1*H*-pyrazol-4-yl)-1,3,4-oxadiazol-2-yl)thio)-*N*-(4-(3-(4-nitrophenyl)acryloyl)phenyl)acetamide (14g)

Orange crystals (0.168 g, 51% yield); m.p. 168–170 °C; FT-IR; ν_max_ 3313 (NH), 1660 (Ketone CO), 1597 (amide CO); ^1^H NMR (400 MHz, DMSO-*d*_*6*_) δ (ppm): 10.99 (s, 1H, CO-N**H**), 9.25 (s, 1H, pyrazole C_5_–H), 8.28 (d, 2 H, *J* = 8.00 Hz, Ar–H), 8.20–8.10 (m, 5 H, Ar-H), 7.98 (d, 2 H, *J* = 8.00 Hz, Ar–H), 7.88 (d, 2 H, *J* = 8.00 Hz, Ar–H), 7.83–7.77 (m, 3 H, Ar-H), 7.58–7.54 (m, 2 H, Ar-H), 7.42–7.41 (m, 1H, Ar-H), 7.03 (d, 2 H, *J* = 8.00 Hz, Ar–H), 4.39 (s, 2 H, C**H**_**2**_), 3.81 (s, 3 H, OC**H**_**3**_); ^13^C-NMR (100 MHz, DMSO-*d*_*6*_) δ (ppm): 187.84 (ketone C = O), 166.16 (amide C = O), 162.71 (oxadiazole C_2_), 161.19 (oxadiazole C_5_), 160.32, 150.70 (pyrazole C_3_), 148.49, 143.83 (Ar-*C* = C), 141.74, 141.08, 139.12 (pyrazole C_5_), 132.76, 131.53, 130.68, 130.43, 130.26, 130.14, 127.84, 126.48, 124.39, 124.10, 119.35 (CO-*C* = C), 119.11, 114.12, 105.45 (pyrazole C_4_), 55.66 (OCH_3_), 37.32 (CH_2_); Elemental analysis for C_35_H_26_N_6_O_6_S (658.69) (Calcd./Found): C, 63.82/63.84; H, 3.98/3.99; N, 12.76/12.77; HPLC analysis: Mobile phase (dipotassium hydrogen phosphate buffer [pH 3.2]: ACN, 30:70 respectively) FR 1mL /min, retention time = 5.93 min; peak area, 96.0% at λ_max_ 254.

##### (*E*)-2-((5-(3-(4-Methoxyphenyl)-1-phenyl-1H-pyrazol-4-yl)-1,3,4-oxadiazol-2-yl)thio)-*N*-(4-(3-(p-tolyl)acryloyl)phenyl)acetamide (14h)

Yellow crystals (0.247 g, 79% yield); m.p. 119–121 °C; FT-IR; ν_max_ 3313 (NH), 1651 (Ketone CO), 1604 (amide CO); ^1^H NMR (400 MHz, DMSO-*d*_*6*_) δ (ppm): 11.17 (s, 1H, CO-N**H**), 9.32 (s, 1H, pyrazole C_5_–H), 8.14 (d, 2 H, *J* = 8.00 Hz, Ar–H), 7.99 (d, 2 H, *J* = 8.00 Hz, Ar–H), 7.90 (d, 2 H, *J* = 8.00 Hz, Ar–H), 7.83 (d, 2 H, *J* = 8.00 Hz, Ar–H), 7.80–7.76 (m, 3 H, Ar-H), 7.70 (d, 1H, C**H** = CH *J* = 16.00 Hz Ar-H), 7.58–7.55 (m, 2 H, Ar-H), 7.42–7.41 (m, 1H, Ar-H), 7.27 (d, 2 H, *J* = 8.00 Hz, Ar–H), 7.03 (d, 2 H, *J* = 8.00 Hz, Ar–H), 4.42 (s, 2 H, C**H**_**2**_), 3.81 (s, 3 H, OC**H**_**3**_), 2.36 (s, 3 H, C**H**_**3**_); ^13^C-NMR (100 MHz, DMSO-*d*_*6*_) δ (ppm): 188.04 (ketone C = O), 166.16 (amide C = O), 162.70 (oxadiazole C_2_), 161.18 (oxadiazole C_5_), 160.32, 150.68 (pyrazole C_3_), 144.02, 143.51 (Ar-*C* = C), 141.06, 139.13 (pyrazole C_5_), 133.23, 132.50, 131.60, 130.44, 130.34, 130.14, 130.01, 129.32, 127.83, 124.11, 121.35, 119.34 (CO-*C* = C), 119.08, 114.12, 105.47 (pyrazole C_4_), 55.67 (OCH_3_), 37.25 (CH_2_), 21.55 (CH_3_); Elemental analysis for C_36_H_29_N_5_O_4_S (627.72) (Calcd./Found): C, 68.88/68.90; H, 4.66/4.69; N, 11.16/11.18; HPLC analysis: Mobile phase (dipotassium hydrogen phosphate buffer [pH 3.2]: ACN, 30:70 respectively) FR 1mL /min, retention time = 7.83 min; peak area, 95.3% at λ_max_ 254.

##### (*E*)-2-((5-(3-(4-Methoxyphenyl)-1-phenyl-1H-pyrazol-4-yl)-1,3,4-oxadiazol-2-yl)thio)-*N*-(4-(3-(naphthalen-1-yl)acryloyl)phenyl)acetamide (14i)

Yellow crystals (0.209 g, 63% yield); m.p. 120–122 °C; FT-IR; ν_max_ 3312 (NH), 1656 (Ketone CO), 1598 (amide CO); ^1^H NMR (400 MHz, DMSO-*d*_*6*_) δ (ppm): 10.83 (s, 1H, CO-N**H**), 9.22 (s, 1H, pyrazole C_5_–H), 8.55 (d, 1H, C**H** = CH *J* = 16.00 Hz, Ar–H), 8.29 (d, 1H, *J* = 8.00 Hz, Ar–H), 8.23 (t, 3 H, *J* = 8.00 Hz, Ar-H), 8.07 (d, 1H, *J* = 8.00 Hz, Ar–H), 8.03–8.01 (m, 2 H, Ar-H), 7.97 (d, 2 H, *J* = 8.00 Hz Ar-H), 7.88 (d, 2 H, *J* = 8.00 Hz Ar-H), 7.81 (d, 2 H, *J* = 8.00 Hz Ar-H), 7.69–7.61 (m, 3 H, Ar-H), 7.59–7.55 (m, 2 H, Ar-H), 7.42-7,41 (m, 1H Ar-H), 7.03 (d, 2 H, *J* = 8.00 Hz Ar-H), 4.37 (s, 2 H, C**H**_**2**_), 3.81 (s, 3 H, OC**H**_**3**_); ^13^C-NMR (100 MHz, DMSO-*d*_*6*_) δ (ppm): 188.02 (ketone C = O), 169.44, 166.07 (amide C = O), 160.33 (oxadiazole C_2_), 160.12 (oxadiazole C_5_), 150.74 (pyrazole C_3_), 143.55 (Ar-*C* = C), 139.91 (pyrazole C_5_), 139.12 133.85, 133.15, 131.86, 131.67, 131.50, 131.24, 130.57, 130.44, 130.16, 130.13, 129.27, 127.87, 127.73, 126.79, 126.19, 124.94, 124.31, 124.10, 123.46, 119.37 (CO-*C* = C), 119.16, 115.43, 114.13, 55.67 (OCH_3_), 37.36 (CH_3_); Elemental analysis for C_39_H_29_N_5_O_4_S (663.75) (Calcd./Found): C, 70.57/70.59; H, 4.40/4.41; N, 10.55/10.58; HPLC analysis: Mobile phase (dipotassium hydrogen phosphate buffer [pH 3.2]: ACN, 30:70 respectively) FR 1mL /min, retention time = 9.83 min; peak area, 94.8% at λ_max_ 254.

## Biology

### In vitro anticancer activity

#### In vitro one-dose assay on NCI 60 cancer cell lines

The thirty-three selected compounds (**10a-10c**, **11a-11c**, **12a-12i**, **13a-13i** and **14a-14i**) were primary evaluated for their in vitro anticancer activity at single dose concentration on a panel of about 60 cancer cell lines derived from nine different cancer types: lung, leukemia, colon, ovarian, melanoma, renal, prostate, CNS and breast cancers. The tested compounds seeded to the culture at a single at single concentration 10^− 5^ M, then incubated for 48 h. the end determinations were made with a protein binding dye, Sulforhodamine B (SRB). The percent growth was evaluated spectrophotometrically versus control (untreated with test agents). Compounds which exhibit significant growth inhibition (**10b**,** 11a**,** 11b** and **11c)** are again evaluated against the 60 cell lines panel at five concentration levels, 10-fold dilutions each with the top dose being 10^− 4^ molar (100 μm) *via* solubilizing drug in dimethylsulphoxide. After drug addition, cells were incubated at at 37 °C, 5% CO_2_, 95% air, and 100% relative humidity for 48 h then staining by SRB and the absorbance was evaluated spectrophotometrically by using an automated plate. The percent growth was calculated at different drug concentration levels and at different time. These methods of assay were performed according to the protocol of the Drug Evaluation Branch, National Cancer Institute (NCI), Bethesda, MA, USA. The methodology details of the assay is described on the web site of NCI [66] (https://dtp.cancer.gov/discovery_development/nci-60/methodology.htm).

### Cytotoxicity IC_50_ on normal human cells WI-38

Cell lines were obtained from American Type Culture Collection; cells were cultivated using Dulbecco’s modified Eagle’s medium (DMEM(Invitrogen/Life Technologies)), and the cytotoxicity of the tested compounds was determined using MTT (3-[4,5- dimethylthiazole-2yl]-2,5-diphenyltetrazolium bromide) assay [67]. Cell viability is determined spectrophotometrically by counting the number of living cells after treating the cells with tested compounds compared to untreated control cell lines.

### Enzymatic assay

#### VEGFR-2 Inhibition assay

Enzyme reactions were performed in 50 mM Tris-HCl pH 7.5, 5 mM MnCl2, 5 mM MgCl2, 0.01% Tween-20 and 2 mM dithiothreitol, containing 10 µM ATP, 0.1 µg/ml biotinylated poly-GluTyr (4:1) and 0.1 nM of VEGFR-2 (Millipore, UK). Before the catalytic initiation with ATP, the tested compounds at final concentrations ranging from 0 to 300 µg/ml and enzyme were incubated for 5 min at room temperature. The reactions were quenched by the addition of 25 µl of 100 mM ethylenediaminetetraacetic acid, 10 µg/ml Alpha Screen streptavidin donor beads, and 10 µg/ml acceptor beads in 62.5 mM 4-(2-hydroxyethyl)-1-piperazineethanesulfonic acid pH 7.4, 250 mM NaCl, and 0.1% bovine serum albumin. The plate was incubated in the dark overnight and then read by ELISA Reader (PerkinElmer). Wells containing the substrate and the enzyme without compounds were used as reaction control. Wells containing biotinylated poly-GluTyr (4:1) and enzyme without ATP were used as basal control. Percent inhibition was calculated by the comparison of compounds treated to control incubations. The concentration of the test compound causing 50% inhibition (IC_50_) was calculated from the concentration–inhibition response curve (triplicate determinations) and the data were compared with sorafenib (Sigma-Aldrich) as standard VEGFR-2 inhibitor.

#### EGFR inhibitory assay

A cell-free assay was used to investigate the mechanism of inhibition of EGFR kinase. Kit used for immune-assay was cloud clone SEA757Hu 96 Tests. 200 lM (EGFR) was used. From the following equation: E (%) = E max/(1 + [I]/ID50), where E (%) is the fraction of the enzyme activity measured in the presence of the inhibitor, E max is the activity in the absence of the inhibitor, [I] is the inhibitor concentration and ID_50_ is the inhibitor concentration at which E (%) = 0.5 E max, a dose–response curve was generated. Mean values of two independent replicates for each experiment were used for the interpolation.

#### Cell cycle analysis

After treatment with test compounds for the specified duration, cells (105 cells) are collected by trypsinization and washed twice with ice-cold PBS (pH 7.4). Cells are resuspended in two milliliters of 60% ice-cold ethanol and incubated at 4 °C for 1 h for fixation. Fixed cells are washed twice again with PBS (pH 7.4) and re-suspended in 1 mL of PBS containing 50 µg/mL RNAase A and 10 µg/mL propidium iodide (PI). After 20 min of incubation in dark at 37 C, cells are analyzed for DNA contents using flow cytometry analysis using FL2 (λex/em 535/617 nm) signal detector (ACEA Novocyte™ flowcytometer, ACEA Biosciences Inc., San Diego, CA, USA). For each sample, 12,000 events are acquired. Cell cycle distribution is calculated using ACEA NovoExpress™ software (ACEA Biosciences Inc., San Diego, CA, USA) [68].

#### Apoptosis assay

Apoptosis and necrosis cell populations are determined using Annexin V-FITC apoptosis detection kit (Abcam Inc., Cambridge Science Park, Cambridge, UK) coupled with 2fluorescent channels flowcytometry. After treatment with test compounds for the specifiedduration, cells (105 cells) are collected by trypsinization and washed twice with ice-coldPBS (pH 7.4). Then, cells are incubated in dark with 0.5 ml of Annexin V-FITC/PI solutionfor 30 min in dark at room temperature according to manufacturer protocol. After staining, cells are injected via ACEA Novocyte™ flowcytometer (ACEA Biosciences Inc., SanDiego, CA, USA) and analyzed for FITC and PI fluorescent signals using FL1 and FL2signal detector, respectively (λex/em 488/530 nm for FITC and λex/em 535/617 nm for PI).For each sample, 12,000 events are acquired and positive FITC and/or PI cells are quantifiedby quadrant analysis and calculated using ACEA NovoExpress™ software (ACEABiosciences Inc., San Diego, CA, USA) [69, 70].

#### Evaluation of nitric oxide release

A solution of the appropriate compound (20 µL of 10^− 4^ M in 100 mL DMSO) was added to 2 ml of 1:1 V/V mixture of 50 mM phosphate buffer (pH 7.4) and methanol containing (5 × 10-4 M) L-cysteine. After 1 h at 37Cº, 1 ml of the reaction mixture was treated with 250 µL of Griess reagent. After 5, 10, 15, 20, 25, 30, 35, 40, 45, 50 min., the absorbance was measured at 540 nm. Sodium nitrite standard solutions (10–50 nmol/ml) were used to construct the calibration curve [65]. Griess reagent was prepred as follows: [sulfanilamide (4 g), N-naphthylethylenediamine dihydrochloride (0.2 g), 85% phosphoric acid (10 ml) in distilled water (final volume: 100 ml)].

#### Docking methodology

AutoDock Vina v.1.2.0 was used for carrying out the molecular docking and Discovery Studio Visualizer was used for analyzing the interactions between the target receptor and ligands. For more details see supporting information.

## Supplementary Information

Below is the link to the electronic supplementary material.

Supplementary Material 1

## Data Availability

Data is provided within the manuscript or supplementary information files.
