# Supplementary material for: Novel pyrazole–oxadiazole–chalcone/oxime hybrids as dual EGFR/VEGFR-2 inhibitors with promising anticancer potential: a comprehensive cytotoxicity evaluation, mechanistic insights and SAR analysis
Source: Mol Divers. 2026 Jan 21;30(3):4667–99. doi: 10.1007/s11030-025-11411-3 (PMC13198519; doi:10.1007/s11030-025-11411-3)
Supplement: Supplementary file 1 — Supplementary Material 1 [file 11030_2025_11411_MOESM1_ESM.pdf]

Novel pyrazole–oxadiazole–chalcone/oxime hybrids as dual EGFR/VEGFR-2 inhibitors with promising anticancer potential: a comprehensive cytotoxicity evaluation, mechanistic insights and SAR analysis

Omar Alshazly[1,2], Mohamed Abdel-Aziz[2], Gamal El-Din A. Abuo-Rahma[2,3*], Mamdouh F. A. Mohamed[1,4]

[1]*Department of Pharmaceutical Chemistry, Faculty of Pharmacy, Sohag University, 82524 Sohag, Egypt.*

[2]*Department of Medicinal Chemistry, Faculty of Pharmacy Minia University, 61519 Minia, Egypt.*

[3]*Department of Pharmaceutical Chemistry, Faculty of Pharmacy, Deraya University, New-Minia, Egypt.*

[4]*Department of Pharmaceutical Chemistry, Faculty of Pharmacy, New Valley University, New Valley, 72511, Egypt.*

***To whom correspondence should be addressed:**

* Gamal El-Din. A. Abuo -Rahma

[2]*Department of Medicinal Chemistry, Faculty of Pharmacy Minia University, 61519 Minia, Egypt.*

[3]*Department of Pharmaceutical Chemistry, Faculty of Pharmacy, Deraya University, New-Minia, Egypt.*

E-mail: gamal.aborahama@mu.edu.eg

Tel.:(002)-201003069431

In vitro assay on full NCI -60 cell lines:

Table 1: GI % for *in vitro* subpanel tumor cell lines at 10 μM concentration for compounds **10a-c** and **11a-c**

Subpanel	Compounds.					
	**10a**	**10b**	**10c**	**11a**	**11b**	**11c**
**Leukemia**						
**CCRF-CEM**	37.13	74.24	11.45	194.34	191.7	134.26
**HL-60(TB)**	48.62	12.46	20.44	193.79	193.71	157.64
**K-562**	31.85	52.54	33.95	141.6	148.14	82.49
**MOLT-4**	28.62	48.19	NA	193.56	197.01	183.5
**RPMI-8226**	22.91	70.59	32.25	171.64	180.78	139.49
**SR**	74.01	97.54	68.86	196.58	197.63	192.53
**Non-Small Cell Lung Cancer**						
**A549/ATCC**	23.49	39.46	40.38	61.94	69.87	74.22
**EKVX**	69.67	96.2	150.99	115.98	124.75	102.4
**HOP-62**	ND	ND	ND	ND	ND	ND
**HOP-92**	37.59	102.65	145.73	66.79	86.42	68.44
**NCI-H226**	31.98	86.31	147.15	103.14	110.08	106.99
**NCI-H23**	36.94	76.11	94.43	94.15	103.5	74.59
**NCI-H322M**	NA	18.01	41.92	47.78	59.74	37.27
**NCI-H460**	28.82	59.79	69.96	98.89	129.57	86.97
**NCI-H522**	122.26	72.78	158.06	169.53	170.78	168.8
**Colon Cancer**						
**COLO 205**	NA	12.26	NA	75.75	70.1	NA
**HCC-2998**	NA	NA	20.09	71.49	81.65	32.74
**HCT-116**	17.29	41.14	70.66	79.33	89.07	65.75
**HCT-15**	NA	33.2	19.19	77.17	79.71	60.24
**HT29**	NA	36.79	27.06	101.05	93.72	63.75
**KM12**	26.11	46.96	31.5	99.23	132.88	80.89
**SW-620**	NA	42.38	28.1	83.94	91.49	18.45
**CNS Cancer**						
**SF-268**	31.11	69.57	100.75	81.95	106.88	51.18
**SF-295**	ND	ND	ND	ND	ND	ND
**SF-539**	55.67	111.58	168	137.05	145.43	134.21
**SNB-19**	21.66	49.65	72.96	73.57	78.16	47.23
**SNB-75**	16.49	117.84	119.61	108.99	117.51	121.25
**U251**	ND	ND	ND	ND	ND	ND
**Melanoma**						
**LOX IMVI**	32.34	93.19	97.18	85.62	98.81	181.82
**MALME-3M**	NA	11.16	83.31	91.54	91.63	79.18
**M14**	24.46	48.37	35.93	105.11	119.61	75.43
**MDA-MB-435**	NA	NA	NA	133.36	153.14	40.16
**SK-MEL-2**	63.99	136.8	147.98	147.49	174.84	186.72
**SK-MEL-28**	44.09	60.78	74.74	82.79	97.35	54
**SK-MEL-5**	40.44	89.09	78.2	146.76	161.03	77.92
**UACC-257**	75.85	71.08	113.44	115.55	114.58	148.33
**UACC-62**	56.03	91.69	114.09	97.2	125.23	73.51
**Ovarian Cancer**						
**IGROV1**	13.08	38.82	83.78	76.28	83.85	57.24

**OVCAR-3**	NA	20.72	59.09	142.18	138.48	83.32
**OVCAR-4**	28.54	79.11	77.72	64.08	84.78	97.96
**OVCAR-5**	NA	17.4	33.91	37.04	48.4	26.88
**OVCAR-8**	29.4	66.02	85.43	75.89	90.84	159.26
**NCI/ADR-RES**	14.25	67.53	79.26	157.2	171.88	162.29
**SK-OV-3**	47.55	99.75	156.54	102.84	111.51	110.87
**Renal Cancer**						
**786-0**	32.16	67.96	95.02	95.86	113.38	125.63
**A498**	37.62	66.09	95.11	82.21	91.91	121.22
**ACHN**	26.75	64.79	82.02	56.4	75.27	80.54
**CAKI-1**	44.71	77.4	174.47	74.55	89.56	128.29
**RXF 393**	ND	ND	ND	ND	ND	ND
**SN12C**	19.72	39.11	70.76	58.96	72.02	67.84
**TK-10**	45.53	97.58	116.1	73.19	96.89	81.48
**UO-31**	ND	ND	ND	ND	ND	ND
**Prostate Cancer**						
**PC-3**	NA	68.34	36.07	87.79	97.99	95.79
**DU-145**	NA	81.29	42.38	74.84	78.21	52.76
**Breast Cancer**						
**MCF7**	NA	78.2	94.19	97.69	113.78	84.28
**HS578T**	NA	58.77	80.75	56.33	76.44	45
**BT-549**	NA	110.67	125.79	161.57	163.23	148.78
**T-47D**	NA	63.33	102.69	104.71	115.38	101.87
**MDA-MB-468**	ND	ND	ND	ND	ND	ND

NA: not applicable where GI% =<10%, ND: not determined

Table 2: GI % for *in vitro* subpanel tumor cell lines at 10 μM concentration for compounds **12a-g**.

**Subpanel**	**Compounds.**						
	**12a**	**12b**	**12c**	**12d**	**12e**	**12f**	**12g**
**Leukemia**							
**CCRF-CEM**	18.16	NA	NA	55.25	16.62	NA	NA
**HL-60(TB)**	23.49	NA	11.61	34.96	11.4	16.23	NA
**K-562**	19.76	17.6	21.17	36.56	48.85	15.14	NA
**MOLT-4**	32.37	24.46	21.38	68.25	28.34	33.76	NA
**RPMI-8226**	94.94	24.72	24.39	45.98	76.4	76.38	NA
**SR**	29.82	20.87	ND	54.42	45.72	ND	15.29
**Non-Small Cell Lung Cancer**							
**A549/ATCC**	NA	18.62	NA	24.62	38.4	NA	NA
**EKVX**	NA	NA	12.05	NA	10.7	30.46	NA
**HOP-62**	NA	NA	NA	NA	NA	10.26	NA
**HOP-92**	NA	NA	NA	12.79	NA	24.15	NA
**NCI-H226**	NA	NA	30.3	NA	13.54	47.05	NA
**NCI-H23**	NA	11.6	NA	NA	NA	44.86	10.82
**NCI-H322M**	NA	NA	NA	NA	NA	NA	NA
**NCI-H460**	NA	NA	NA	NA	NA	NA	NA
**NCI-H522**	11.92	16.87	12.26	38.74	21.25	25.44	35.06
**Colon Cancer**							
**COLO 205**	NA	NA	NA	NA	NA	NA	NA
**HCC-2998**	NA	NA	NA	NA	NA	NA	NA

**HCT-116**	65.39	25.73	19.04	45.1	112.16	62.74	13.33
**HCT-15**	21.31	NA	29.47	26.79	32.18	36.17	NA
**HT29**	NA	NA	NA	30.83	34.11	NA	12.39
**KM12**	16.62	NA	NA	NA	41.71	NA	NA
**SW-620**	NA	NA	NA	NA	62.3	NA	NA
**CNS Cancer**							
**SF-268**	14.8	11.7	NA	NA	24.78	10.28	NA
**SF-295**	NA	NA	18.03	NA	NA	84.72	NA
**SF-539**	NA	NA	12.17	NA	15.28	55.09	NA
**SNB-19**	12.52	NA	12.76	12.43	19.82	59.89	17.91
**SNB-75**	14.54	21.32	NA	NA	31.54	57.41	NA
**U251**	NA	13.97	NA	20.87	47.72	34.96	14.17
**Melanoma**							
**LOX IMVI**	12.65	36.65	29.97	29.43	63.42	43.93	15.03
**MALME-3M**	NA	NA	NA	NA	NA	14.08	NA
**M14**	NA	NA	NA	NA	NA	NA	NA
**MDA-MB-435**	14.73	NA	NA	7.64	33.61	12.93	NA
**SK-MEL-2**	NA	NA	NA	23.63	NA	NA	17.85
**SK-MEL-28**	NA	NA	NA	NA	NA	NA	NA
**SK-MEL-5**	10.38	NA	NA	NA	NA	10.89	NA
**UACC-257**	NA	NA	NA	11.34	NA	NA	NA
**UACC-62**	30.57	ND	26.55	ND	ND	54.61	ND
**Ovarian Cancer**							
**IGROV1**	NA	NA	NA	NA	NA	NA	NA
**OVCAR-3**	12.62	NA	NA	13.26	29.23	NA	NA
**OVCAR-4**	NA	NA	NA	NA	NA	NA	NA
**OVCAR-5**	NA	NA	NA	NA	NA	NA	NA
**OVCAR-8**	10.13	10.19	NA	36.87	19.17	18.98	NA
**NCI/ADR-RES**	15.92	NA	NA	12.17	17.15	19.68	NA
**SK-OV-3**	NA	NA	NA	NA	NA	15.15	13.48
**Renal Cancer**							
**786-0**	NA	NA	NA	NA	10.55	37.32	NA
**A498**	NA	NA	NA	NA	NA	NA	NA
**ACHN**	NA	NA	NA	NA	NA	NA	NA
**CAKI-1**	20.21	11.47	NA	NA	22.69	14.18	NA
**RXF 393**	NA	NA	NA	NA	NA	104.52	NA
**SN12C**	10.76	NA	NA	14.74	36.31	23.27	10.99
**TK-10**	NA	NA	NA	NA	NA	NA	NA
**UO-31**	39.58	29.67	31.21	22.37	32.5	38.09	13.91
**Prostate Cancer**							
**PC-3**	21.97	NA	21.22	23.32	30.68	15.09	NA
**DU-145**	13.73	NA	NA	NA	20.8	NA	NA
**Breast Cancer**							
**MCF7**	61.29	29.58	50.63	43.8	93.56	76.93	NA
**HS578T**	NA	18.48	NA	NA	19.48	52.27	14.7
**BT-549**	NA	NA	NA	NA	12.78	22.25	16.98
**T-47D**	NA	NA	NA	NA	NA	24.99	NA
**MDA-MB-468**	NA	12.34	21.25	26.54	21.02	22	NA

NA: not applicable where GI% =<10%, ND: not determined

Table 3: GI % for *in vitro* subpanel tumor cell lines at 10 μM concentration for compounds **12h-i,** and **13a-e**.

Subpanel	Compounds.						
	**12h**	**12i**	**13a**	**13b**	**13c**	**13d**	**13e**
**Leukemia**							
**CCRF-CEM**	30.32	78.17	NA	15.09	26.2	40.57	NA
**HL-60(TB)**	23.15	NA	NA	14.19	10.34	37.93	NA
**K-562**	25.23	36.59	NA	16.75	15.36	28.26	NA
**MOLT-4**	29.99	67.15	14.53	33.96	26.59	45.17	NA
**RPMI-8226**	52.29	38.18	70.03	20.45	25.14	59.52	35.57
**SR**	27.6	73.81	26.85	12.81	27.03	49.53	15.79
**Non-Small Cell Lung Cancer**							
**A549/ATCC**	12.36	NA	27.2	NA	NA	NA	26.23
**EKVX**	NA	NA	NA	NA	NA	NA	NA
**HOP-62**	NA	NA	NA	NA	NA	NA	NA
**HOP-92**	NA	NA	16.37	13.15	13.72	20.22	NA
**NCI-H226**	11.32	NA	NA	NA	NA	NA	NA
**NCI-H23**	NA	NA	18.23	NA	NA	NA	20.93
**NCI-H322M**	NA	NA	NA	NA	NA	NA	NA
**NCI-H460**	1.95	NA	NA	NA	NA	NA	NA
**NCI-H522**	12.09	18.74	36.36	16.71	NA	11.07	30.06
**Colon Cancer**							
**COLO 205**	NA	NA	NA	NA	NA	NA	NA
**HCC-2998**	NA	NA	NA	NA	NA	NA	NA
**HCT-116**	37.03	23.31	72.66	28.79	11.88	30.63	46.82
**HCT-15**	19.51	68.52	12.11	18.99	28.16	32.8	NA
**HT29**	NA	NA	10.25	NA	NA	NA	10.28
**KM12**	12.82	NA	NA	NA	NA	NA	NA
**SW-620**	NA	23.23	NA	NA	NA	NA	NA
**CNS Cancer**							
**SF-268**	17.17	NA	16.02	11.62	18.36	16.12	NA
**SF-295**	NA	NA	NA	15.8	NA	NA	NA
**SF-539**	NA	NA	17.74	NA	NA	NA	NA
**SNB-19**	NA	NA	19.44	NA	10.2	12.93	NA
**SNB-75**	11.27	NA	16.5	30.91	NA	16.09	NA
**U251**	14.19	NA	32.68	NA	NA	10.24	14.98
**Melanoma**							
**LOX IMVI**	16.48	169.52	48.81	NA	NA	10.43	32.94
**MALME-3M**	NA	NA	NA	NA	NA	NA	NA
**M14**	NA	22.84	NA	14.41	NA	NA	NA
**MDA-MB-435**	NA	10.6	NA	13.33	NA	10.33	NA
**SK-MEL-2**	NA	NA	14.95	10.33	NA	NA	NA
**SK-MEL-28**	NA	NA	NA	NA	NA	NA	NA
**SK-MEL-5**	NA	NA	NA	14.11	21.37	NA	NA
**UACC-257**	NA	NA	NA	4.01	NA	NA	6.94
**UACC-62**	34.64	27.17	ND	33.73	32.76	23.42	ND
**Ovarian Cancer**							
**IGROV1**	NA	35.08	NA	NA	NA	NA	NA
**OVCAR-3**	NA	NA	NA	NA	NA	NA	NA

OVCAR-4	NA	NA	NA	NA	NA	NA	NA
OVCAR-5	NA	NA	NA	NA	NA	NA	NA
OVCAR-8	14.55	NA	NA	NA	NA	39.2	11.87
NCI/ADR-RES	11.88	NA	15.76	16.54	16.92	11.49	10.09
SK-OV-3	NA	NA	NA	NA	NA	NA	NA
**Renal Cancer**							
786-0	NA	NA	NA	NA	11.66	NA	NA
A498	NA	NA	NA	NA	NA	NA	NA
ACHN	NA	NA	NA	NA	NA	10.75	NA
CAKI-1	15.04	NA	NA	26.09	14.27	NA	NA
RXF 393	NA	NA	NA	19.36	10.22	12.3	NA
SN12C	11.82	10.92	20.71	NA	NA	NA	15.23
TK-10	NA	NA	NA	NA	NA	NA	NA
UO-31	39.46	23.7	15.28	35.63	24.43	25.71	19.52
**Prostate Cancer**							
PC-3	18.83	15.57	19.89	25.45	20.58	14.25	14.23
DU-145	NA	NA	NA	NA	NA	11.09	NA
**Breast Cancer**							
MCF7	51.24	41.43	54.3	55.3	27.89	38.19	61.37
HS578T	16.27	NA	29.15	15.9	11.51	19.29	15.54
BT-549	NA	NA	26.18	NA	NA	NA	NA
T-47D	NA	14.51	NA	22.67	19.88	NA	NA
MDA-MB-468	NA	12.63	NA	20.4	32.54	28.43	NA

NA: not applicable where GI% =<10%, ND: not determined

Table 4: GI % for *in vitro* subpanel tumor cell lines at 10 μM concentration for compounds **13f-i** and **14a-c**.

Subpanel	Compounds.						
	**13f**	**13g**	**13h**	**13i**	**14a**	**14b**	**14c**
**Leukemia**							
CCRF-CEM	20.01	15.71	22.96	22.38	18.02	NA	10.36
HL-60(TB)	NA	10.05	18.68	NA	NA	NA	NA
K-562	13.63	16.51	25.37	NA	10.84	NA	NA
MOLT-4	32.65	27.53	34.37	28	34.25	NA	24.51
RPMI-8226	80.11	63.66	46.58	16.3	57.97	NA	NA
SR	ND	ND	35.55	33.28	31.27	17.39	38.22
**Non-Small Cell Lung Cancer**							
A549/ATCC	NA	NA	25.89	15.48	24.91	NA	20.61
EKVX	NA	28.86	10.57	NA	NA	NA	NA
NCI-H226	23.95	36.57	NA	NA	NA	NA	NA
NCI-H23	NA	19.18	21.58	NA	NA	NA	NA
NCI-H522	15.73	16.18	13.39	20.57	25.93	NA	34.21
**Colon Cancer**							
HCT-116	35.42	70.11	44.81	NA	71.48	NA	NA
HCT-15	15.5	26.48	21.16	15.4	NA	NA	NA
HT29	NA	NA	NA	NA	11.22	NA	NA
KM12	12.82	NA	11.53	NA	NA	NA	NA
**CNS Cancer**							

**SF-268**	12.49	NA	NA	NA	NA	NA	NA
**SF-295**	NA	25.19	NA	NA	NA	NA	NA
**SF-539**	11.08	11.79	NA	NA	NA	NA	NA
**SNB-19**	15.13	16.43	11.23	NA	NA	NA	NA
**SNB-75**	21.49	18.21	NA	NA	NA	NA	NA
**U251**	14.87	16.16	20.9	14.12	11.18	NA	NA
**Melanoma**							
**LOX IMVI**	29.41	46.09	22.45	107.63	NA	NA	NA
**SK-MEL-2**	NA	NA	10.35	NA	12.69	NA	19.09
**UACC-62**	35.98	41.45	33.16	ND	ND	24.74	ND
**Ovarian Cancer**							
**IGROV1**	12.74	19.2	NA	37.84	NA	NA	NA
**OVCAR-5**	NA	NA	NA	NA	NA	-16.12	NA
**OVCAR-8**	NA	NA	28.97	NA	NA	NA	NA
**NCI/ADR-RES**	18.48	NA	NA	NA	NA	NA	NA
**Renal Cancer**							
**CAKI-1**	NA	14.33	16.73	NA	NA	NA	NA
**RXF 393**	NA	32.68	NA	NA	NA	NA	NA
**SN12C**	NA	16.33	NA	NA	12.8	NA	NA
**UO-31**	46.53	39.66	48.37	20.48	20.47	31.37	14.29
**Prostate Cancer**							
**PC-3**	20.91	21.56	21.75	NA	12.3	NA	NA
**Breast Cancer**							
**MCF7**	48.32	65.37	63.56	23.78	36.41	24.48	11.53
**HS578T**	13.54	27.74	15.11	NA	NA	NA	NA
**T-47D**	NA	NA	16.5	NA	NA	NA	NA
**MDA-MB-468**	20.85	23.83	12.49	10.08	NA	18.15	NA

NA: not applicable where GI% =<10%, ND: not determined

Table 5: GI % for *in vitro* subpanel tumor cell lines at 10 μM concentration for compounds **14d-i**.

**Subpanel**	**Compounds.**					
	**14d**	**14e**	**14f**	**14g**	**14h**	**14i**
**Leukemia**						
**CCRF-CEM**	29.03	22.45	NA	20.93	11.58	NA
**HL-60(TB)**	24.86	NA	NA	NA	NA	NA
**K-562**	17.53	21.04	NA	NA	NA	NA
**MOLT-4**	44.85	29.34	13.61	NA	19.01	18.5
**RPMI-8226**	52.98	81.37	75.09	40.44	36.98	NA
**SR**	ND	32.7	ND	ND	24.21	23.43
**Non-Small Cell Lung Cancer**						
**A549/ATCC**	NA	NA	NA	NA	NA	NA
**EKVX**	16.44	16.74	NA	NA	NA	NA
**NCI-H226**	NA	NA	NA	13.37	NA	NA
**NCI-H522**	13.95	17.68	12.41	NA	NA	15.75
**Colon Cancer**						
**HCT-116**	31.78	36.83	19.53	10.8	21.46	16.68
**HCT-15**	28.51	15.29	NA	NA	12.07	NA
**KM12**	NA	NA	NA	NA	6.76	NA

**CNS Cancer**						
**SF-295**	12.11	NA	18.69	NA	NA	NA
**SF-539**	NA	13.04	NA	NA	NA	NA
**SNB-19**	NA	13.09	11.91	NA	NA	NA
**SNB-75**	11.08	NA	20.56	NA	NA	NA
**U251**	NA	14.24	NA	NA	NA	NA
**Melanoma**						
**LOX IMVI**	11.68	17.4	NA	NA	NA	NA
**MDA-MB-435**	NA	10.25	NA	NA	11.74	NA
**SK-MEL-2**	NA	NA	NA	NA	NA	14.65
**SK-MEL-5**	13.83	14.77	NA	NA	NA	NA
**UACC-62**	25.81	32.35	27.8	17.73	23.89	ND
**Ovarian Cancer**						
**OVCAR-4**	NA	10.53	NA	NA	NA	NA
**Renal Cancer**						
**786-0**	NA	11.7	NA	NA	NA	NA
**CAKI-1**	NA	12.2	NA	NA	NA	NA
**RXF 393**	28	NA	NA	NA	NA	NA
**UO-31**	17.39	30.19	35.16	30.07	30.86	12.15
**Prostate Cancer**						
**PC-3**	13.79	18.08	16.6	10.14	NA	10.93
**Breast Cancer**						
**MCF7**	47.75	49.99	37.48	10.1	36.81	17.98
**HS578T**	NA	22.3	NA	NA	NA	NA
**T-47D**	NA	11.85	NA	NA	NA	NA
**MDA-MB-468**	31.27	32.49	19.05	NA	NA	NA

NA: not applicable where GI% =<10%, ND: not determined

Table 6: National Cancer Institute *in vitro* five dose evaluation data (µM) of compound **10b** compared to reference drug Gefitinib.

**Subpanel**	**10b**			**Gefitinib (NSC: 715055)**		
	**GI_{50}**	**TGI**	**LC_{50}**	**GI_{50}**	**TGI**	**LC_{50}**
**Leukemia**						
CCRF-CEM	12.4	>100.0	>100.0	**4.56**	19.86	57.94
HL-60(TB)	7.62	26.8	84.2	5.55	16.87	44.87
K-562	7.47	>100.0	>100.0	**2.28**	7.18	22.70
MOLT-4	10.6	30.4	87	**3.92**	16.83	41.02
RPMI-8226	**2.0**	8.5	32	**1.73**	10.23	38.73
SR	13.4	40.9	100	**3.22**	6.44	18.58
**Non-Small Cell Lung Cancer**						
A549/ATCC	5.34	21.6	67.4	7.60	21.78	46.67
EKVX	**2.42**	14.8	51.2	**0.04**	17.30	51.76
HOP-62	**3.78**	12.6	40.7	10.21	35.89	73.28
HOP-92	**1.71**	5.03	19.1	7.46	23.33	49.09
NCI-H226	5.34	19.8	49.6	15.49	33.04	70.31
NCI-H23	**3.36**	15.5	52.4	16.83	46.45	95.06
NCI-H322M	14.6	53.9	>100.0	**0.09**	17.06	59.16
NCI-H460	**2.66**	12.6	39.2	6.65	19.86	45.92

NCI-H522	5.49	18.4	44.6	6.78	38.73	87.10
**Colon Cancer**						
COLO 205	10.4	35.4	>100.0	6.43	20.94	52.84
HCC-2998	**3.67**	>100.0	>100.0	10.84	53.58	73.11
HCT-116	6.24	32.3	>100.0	7.19	23.01	68.71
HCT-15	7.84	>100.0	>100.0	5.22	18.75	49.20
HT29	9.07	33.8	>100.0	4.04	41.59	64.71
KM12	6.84	29.6	>100.0	8.09	32.73	81.10
SW-620	5.52	37.2	>100.0	8.00	29.51	73.11
**CNS Cancer**						
SF-268	8.24	21.8	49.9	7.18	33.04	75.34
SF-295	8.78	22.1	51.3	**2.02**	5.15	16.03
SF-539	6.99	22.5	59.8	10.91	22.86	47.97
SNB-19	12.6	27.3	59	13.12	25.82	50.82
SNB-75	**1.82**	6.07	25.4	5.68	20.99	50.58
U251	**3.7**	13.5	37.1	10.96	23.71	48.64
**Melanoma**						
LOX IMVI	**3.21**	13.4	38.5	8.87	24.55	59.57
MALME-3M	**2.15**	4.34	8.76	**3.08**	8.57	19.23
M14	11.3	40.3	>100.0	5.56	13.71	39.54
MDA-MB-435	12.0	72.1	>100.0	**3.17**	11.67	34.91
SK-MEL-2	**2.63**	6.31	21.2	13.15	27.93	59.29
SK-MEL-28	7.68	21.7	53.8	**0.30**	3.05	7.69
SK-MEL-5	**3.04**	12.7	45	**3.66**	14.76	38.37
UACC-257	**2.88**	8.96	48.5	6.90	15.28	41.02
UACC-62	**4.72**	17	43.9	5.58	10.67	20.42
**Ovarian Cancer**						
IGROV1	7.62	24.5	66.8	**0.19**	9.48	49.20
OVCAR-3	9.25	25.6	67.1	**4.82**	23.77	76.74
OVCAR-4	**1.92**	5.9	24	8.79	26.61	68.87
OVCAR-5	15.8	60.3	>100.0	11.14	25.94	60.39
OVCAR-8	**4.52**	17.3	46.3	10.84	36.90	98.40
NCI/ADR-RES	**3.67**	17.6	62.3	13.30	65.16	>100
SK-OV-3	8.13	20.9	47.3	**0.61**	10.96	35.89
**Renal Cancer**						
786-0	9.52	21.9	48.9	8.61	23.77	55.34
A498	5.95	17.5	42.5	**0.39**	9.40	31.99
ACHN	12.4	28.2	64.1	**0.22**	12.16	34.83
CAKI-1	**3.82**	14.1	38	**0.18**	9.27	33.19
RXF 393	**2.32**	5.89	20.4	**4.76**	21.83	60.67
SN12C	5.95	22.3	68.5	6.17	22.18	55.08
TK-10	8.94	21.5	47.7	**0.11**	14.83	43.85
UO-31	13.0	29.1	65.1	**1.26**	15.67	41.69
**Prostate Cancer**						
PC-3	**3.58**	14.3	39.9	**0.85**	20.18	51.05
DU-145	7.62	21	49.6	**2.57**	25.18	92.68

**Breast Cancer**						
MCF7	**4.43**	34.8	>100.0	10.67	23.12	50.00
MDA-MB-231/ATCC	5.11	19.3	52.8	13.24	26.49	52.84
HS 578T	**2.76**	11.5	39.3	9.31	34.20	78.70
BT-549	**2.59**	12.3	46.2	8.18	21.28	47.75
T-47D	**2.90**	14.6	>100.0	**6.00**	21.93	56.36
MDA-MB-468	**2.33**	8.61	46	**0.01**	10.96	>100
**MIDa**	**6.39**	**21.93**	**47.55**	**6.08**	**22.25**	**52.59**

ND: not determined.
Bold figures indicate superior potency.

Table 7: *In vitro* (GI_{50} and TGI, μM) and selectivity index of the compounds **10b** on nine subpanel tumor cell lines compared to Gefitinib

Subpanel	10b				Gefitinib (NSC: 715055)			
	GI_{50}		TGI		GI_{50}		TGI	
	MID_b	SI	MID_b	SI	MID_b	SI	MID_b	SI
**I**	8.91	0.72	26.65	0.82	3.54	1.72	12.9	1.72
**II**	4.96	1.29	19.35	1.13	7.9	0.77	28.16	0.79
**III**	7.08	0.90	33.66	0.65	7.11	0.86	31.44	0.71
**IV**	7.02	0.91	18.86	1.16	8.3	0.73	21.92	1.02
**V**	5.51	1.16	21.86	1.00	5.58	1.09	14.46	1.54
**VI**	7.26	0.88	24.50	0.90	7.09	0.86	28.46	0.78
**VII**	7.73	0.83	20.06	1.09	2.71	2.24	16.13	1.38
**VIII**	5.60	1.14	17.65	1.24	1.71	**3.56**	22.68	0.98
**IX**	3.35	1.91	16.85	1.30	7.9	0.77	22.99	0.97
**MIDa**	**6.39**		**21.93**		**6.08**		**22.25**	

I: Leukemia, II: Non-Small Cell Lung Cancer, III: Colon Cancer, IV: CNS Cancer, V: Melanoma, VI: Ovarian Cancer, VII: Renal Cancer, VIII: Prostate Cancer, IX Breast Cancer,
MIDa: the average sensitivity of all cell lines for the tested compound, **MID_b**: the average sensitivity of cell lines of a specific subpanel for the tested compound. **SI**: selectivity index

Table 8: National Cancer Institute *in vitro* five dose evaluation data (μM) of compounds **11a, 11b, 11c** compared to Gefitinib.

Subpanel	11a			11b			11c			Gefitinib (NSC: 715055)		
	GI_{50}	TGI	LC_{50}	GI_{50}	TGI	LC_{50}	GI_{50}	TGI	LC_{50}	GI_{50}	TGI	LC_{50}
**Leukemia**												
**CCRF-CEM**	**1.70**	**3.27**	6.28	**1.70**	**3.18**	5.95	**4.27**	>100	>100	**4.56**	19.86	57.94
**HL-60 (TB)**	**1.67**	**3.10**	5.76	**1.84**	**3.27**	5.79	**2.39**	4.51	8.53	5.55	16.87	44.87
**K-562**	**1.69**	9.83	33.4	**1.87**	7.09	34.4	**4.70**	>100	>100	**2.28**	7.18	22.70
**MOLT-4**	**1.41**	**2.79**	5.5	**1.43**	**2.78**	5.4	**2.25**	4.22	7.92	**3.92**	16.83	41.02
**RPMI-8226**	**1.92**	**3.49**	6.34	**1.36**	**2.72**	5.41	**2.15**	3.7	6.35	**1.73**	10.23	38.73
**SR**	**0.31**	**0.807**	**2.88**	**0.21**	**0.441**	**0.895**	**0.88**	**2.13**	**4.75**	**3.22**	6.44	18.58

**Non-Small Cell Lung Cancer**												
**A549/ATCC**	10.0	89.5	>100	6.83	31	>100	7.59	>100	>100	7.60	21.78	46.67
**EKVX**	7.14	44.4	>100	6.96	28.8	>100	20.7	>100	>100	**0.04**	17.30	51.76
**HOP-62**	5.68	21.4	71.6	**4.59**	18.3	51.1	13.0	>100	>100	10.21	35.89	73.28
**HOP-92**	5.31	14	75.9	**3.68**	8.3	37	**4.16**	10.4	>100	7.46	23.33	49.09
**NCI-H226**	8.39	24.6	67.4	5.67	19.8	57.7	6.76	32.9	>100	15.49	33.04	70.31
**NCI-H23**	**4.88**	20.3	69.3	**4.19**	19.1	64.7	17.7	>100	>100	16.83	46.45	95.06
**NCI-H322M**	17.0	83.0	>100	13.4	40.7	>100	89.9	>100	>100	**0.09**	17.06	59.16
**NCI-H460**	**3.25**	10.9	35.5	**3.01**	11.4	34.5	**4.55**	>100	>100	6.65	19.86	45.92
**NCI-H522**	**1.32**	**3.25**	7.99	**1.30**	**3.04**	7.1	**4.02**	29.7	>100	6.78	38.73	87.10
**Colon Cancer**												
**COLO 205**	**4.08**	12.0	68.3	**3.6**	14.3	>100	62	>100	>100	6.43	20.94	52.84
**HCC-2998**	5.58	20.2	70.4	**3.64**	13.3	42.7	6.86	>100	>100	10.84	53.58	73.11
**HCT-116**	**4.66**	16.2	46.1	**4.33**	15.8	43.2	8.77	>100	>100	7.19	23.01	68.71
**HCT-15**	**2.82**	19.6	>100	**4.68**	23.6	90.9	14.0	>100	>100	5.22	18.75	49.20
**HT29**	**3.62**	12.6	>100	**3.75**	13.1	94.8	34.2	>100	>100	4.04	41.59	64.71
**KM12**	**2.90**	12.2	40.7	**3.23**	12.7	42.6	7.41	>100	>100	8.09	32.73	81.10
**SW-620**	5.51	20.4	63.7	**4.99**	19.8	71.4	>100	>100	>100	8.00	29.51	73.11
**CNS Cancer**												
**SF-268**	5.75	21	63.3	**4.5**	16.8	49.7	25.0	>100	>100	7.18	33.04	75.34
**SF-295**	**3.34**	9.52	>100	**2.89**	6.93	50.6	30.2	>100	>100	**2.02**	5.15	16.03
**SF-539**	**3.32**	9.51	33.6	**3.00**	7.30	25.6	**3.11**	8.65	>100	10.91	22.86	47.97
**SNB-19**	8.76	48.7	>100	7.7	31.6	>100	97.9	>100	>100	13.12	25.82	50.82
**SNB-75**	6.26	33	>100	5.59	21.7	60.8	**4.08**	>100	>100	5.68	20.99	50.58
**U251**	**4.99**	18	62	**4.33**	14.9	49	11.7	>100	>100	10.96	23.71	48.64
**Melanoma**												
**LOX IMVI**	5.4	17.1	42.5	**4.39**	15.4	40	9.58	>100	>100	8.87	24.55	59.57
**MALME-3M**	**3.5**	10.4	37.1	**3.08**	10.2	46.5	6.71	56	>100	**3.08**	8.57	19.23
**M14**	**3.59**	13.2	55.4	**2.96**	11.3	>100	12.0	>100	>100	5.56	13.71	39.54
**MDA-MB-435**	**1.46**	**3.65**	9.17	**1.40**	3.61	9.30	41.5	>100	>100	**3.17**	11.67	34.91
**SK-MEL-2**	**1.93**	4.55	12.2	**1.78**	3.94	8.7	**1.82**	**4.52**	>100	13.15	27.93	59.29
**SK-MEL-28**	7.23	51.2	>100	7.06	30.4	>100	38.9	>100	>100	**0.30**	3.05	7.69
**SK-MEL-5**	**2.37**	9.3	31.9	**2.25**	8.25	29.5	15.5	97.1	>100	**3.66**	14.76	38.37
**UACC-257**	5.89	18.5	47.4	**3.95**	15	49.7	6.88	>100	>100	6.90	15.28	41.02
**UACC-62**	**2.59**	10.8	33.4	**2.11**	8.58	33.8	18.1	>100	>100	5.58	10.67	20.42
**Ovarian Cancer**												
**IGROV1**	6.82	27.0	98.9	6.13	24.6	81.0	35.6	>100	>100	**0.19**	9.48	49.20
**OVCAR-3**	**2.56**	6.16	24.5	**2.37**	7.4	36.3	6.37	>100	>100	**4.82**	23.77	76.74
**OVCAR-4**	5.29	21.8	96.6	**4.41**	15.9	79.3	5.01	28.1	>100	8.79	26.61	68.87
**OVCAR-5**	15.7	77.3	>100	15.9	54.0	>100	>100	>100	>100	11.14	25.94	60.39
**OVCAR-8**	5.73	18.8	48.4	5.24	19.3	52.5	**3.63**	70.9	>100	10.84	36.90	98.40
**NCI/ADR-RES**	**2.12**	**4.05**	7.76	**1.79**	**3.55**	7.08	5.9	>100	>100	13.30	65.16	>100
**SK-OV-3**	**3.18**	14.0	66.3	**3.84**	16.4	53.3	21.2	>100	>100	**0.61**	10.96	35.89
**Renal Cancer**												
**786-0**	**3.65**	16.4	>100	**4.22**	17	58.1	7.39	>100	>100	8.61	23.77	55.34
**A498**	5.15	23.0	92.0	**4.28**	16.4	44.1	**2.75**	>100	>100	**0.39**	9.40	31.99
**ACHN**	ND	ND	ND	ND	ND	ND	ND	ND	ND	**0.22**	12.16	34.83
**CAKI-1**	5.3	19.9	57.8	**4.81**	17.7	53.6	5.93	18.1	43.7	**0.18**	9.27	33.19
**RXF 393**	**2.58**	6.22	28.8	**2.64**	6.36	25.7	**2.09**	**4.05**	7.86	**4.76**	21.83	60.67
**SN12C**	7.75	24.0	66.6	6.94	21.9	60.8	6.62	>100	>100	6.17	22.18	55.08

**TK-10**	11.9	42.4	>100	9.49	30.6	96.5	20.8	>100	>100	**0.11**	14.83	43.85
**UO-31**	7.82	48.9	>100	9.68	45.3	>100	42.0	>100	>100	**1.26**	15.67	41.69
**Prostate Cancer**												
**PC-3**	5.23	18.0	52.7	**4.04**	16.5	59.8	6.50	>100	>100	**0.85**	20.18	51.05
**DU-145**	**4.60**	20.1	97.4	**4.26**	17.2	54.1	5.51	>100	>100	**2.57**	25.18	92.68
**Breast Cancer**												
**MCF7**	**3.11**	13.9	48.2	**3.54**	14.8	55.6	23.1	>100	>100	10.67	23.12	50.00
**MDA-MB-231/ATCC**	8.40	60.8	>100	8.65	32.5	>100	48.9	>100	>100	13.24	26.49	52.84
**HS 578T**	**2.48**	5.65	25.2	**2.18**	5.18	19.0	14.8	82	>100	9.31	34.20	78.70
**BT-549**	**3.23**	13.1	36.8	**2.69**	8.15	30.1	13.3	36.3	99.1	8.18	21.28	47.75
**T-47D**	6.63	19.8	47.0	5.29	22.6	80.1	12.0	91.4	>100	**6.00**	21.93	56.36
**MDA-MB-468**	**1.95**	3.73	7.11	**1.52**	**3.36**	7.46	**4.44**	23.5	>100	**0.01**	10.96	>100
**MIDa**	**4.88**	**20.90**	**57.70**	**4.36**	**15.8**	**42.29**	**16.4**	**32.0**	NA	**6.08**	**22.25**	**52.59**

ND: not determined

Bold figures indicate superior potency.

Table 9: *In vitro* (GI_{50} and TGI, µM) and selectivity index of the compounds **11a, 11b,** and **11c** on nine subpanel tumor cell lines compared to Gefitinib.

**Sub-panels**	**11a**				**11b**				**11c**				**Gefitinib (NSC: 715055)**			
	**GI_{50}**		**TGI**		**GI_{50}**		**TGI**		**GI_{50}**		**TGI**		**GI_{50}**		**TGI**	
	**MID_b**	**SI**	**MID_b**	**SI**	**MID_b**	**SI**	**MID_b**	**SI**	**MID_b**	**SI**	**MID_b**	**SI**	**MID_b**	**SI**	**MID_b**	**SI**
**I**	**1.45**	**3.37**	**3.88**	**5.39**	**1.4**	**3.11**	**3.24**	**4.88**	**2.77**	**5.92**	**3.64**	**8.79**	3.54	1.72	12.9	1.72
**II**	6.99	0.70	34.59	0.60	5.51	0.79	20.04	0.79	18.7	0.88	24.33	1.32	7.9	0.77	28.16	0.79
**III**	4.16	1.17	16.17	1.29	4.03	1.08	16.08	0.98	22.2	0.74	NA	NA	7.11	0.86	31.44	0.71
**IV**	5.4	0.90	23.28	0.90	4.66	0.94	16.53	0.96	28.66	0.57	NA	NA	8.3	0.73	21.92	1.02
**V**	3.77	1.29	15.41	1.36	3.22	1.35	11.85	1.33	28.77	0.57	52.54	0.61	5.58	1.09	14.46	1.54
**VI**	5.91	0.83	24.15	0.87	5.66	0.77	20.16	0.78	12.95	1.27	49.5	0.65	7.09	0.86	28.46	0.78
**VII**	6.3	0.77	25.83	0.81	6	0.73	22.18	0.71	12.51	1.31	NA	NA	2.71	2.24	16.13	1.38
**VIII**	4.91	0.99	19.05	1.10	4.15	1.05	16.85	0.94	6.00	2.73	NA	NA	1.71	**3.56**	22.68	0.98
**IX**	4.3	1.13	19.49	1.07	3.97	1.10	14.43	1.09	19.42	0.84	58.3	0.55	7.9	0.77	22.99	0.97
**MIDa**	**4.88**		**20.90**		**4.36**		**15.8**		**16.4**		**32.0**		**6.08**		**22.25**	

I: Leukemia, II: Non-Small Cell Lung Cancer, III: Colon Cancer, IV: CNS Cancer, V: Melanoma,
VI: Ovarian Cancer, VII: Renal Cancer, VIII: Prostate Cancer IX Breast Cancer,
MID_a: the average sensitivity of all cell lines for the tested compound,
MID_b: the average sensitivity of cell lines of a specific subpanel for the tested compound. SI: selectivity index

Developmental Therapeutics Program

One Dose Mean Graph

**NSC:** D-852386 / 1	**Conc:** 1.000E-5 M
**Test Date:** Apr 22, 2024	**Experiment ID:** 2404HT36
**Report Date:** May 30, 2024	

Panel/Cell Line	Growth Percent
Leukemia	
CCRF-CEM	62.87
HL-60(TB)	51.38
K-562	68.15
MOLT-4	71.38
RPMI-8226	77.09
SR	25.99
Non-Small Cell Lung Cancer	
A549/ATCC	76.51
EKVX	30.33
HOP-92	62.41
NCI-H226	68.02
NCI-H23	63.06
NCI-H322M	107.90
NCI-H460	71.18
NCI-H522	-22.26
Colon Cancer	
COLO 205	120.56
HCC-2998	109.37
HCT-116	82.71
HCT-15	90.01
HT29	101.05
KM12	73.89
SW-620	99.49
CNS Cancer	
SF-268	68.89
SF-539	44.33
SNB-19	78.34
SNB-75	83.51
Melanoma	
LOX IMVI	67.66
MALME-3M	90.36
M14	75.54
MDA-MB-435	106.15
SK-MEL-2	36.01
SK-MEL-28	55.91
SK-MEL-5	59.56
UACC-257	24.15
UACC-62	43.97
Ovarian Cancer	
IGROV1	86.92
OVCAR-3	101.84
OVCAR-4	71.46
OVCAR-5	94.05
OVCAR-8	70.60
NCI/ADR-RES	85.75
SK-OV-3	52.45
Renal Cancer	
786-0	67.84
A498	62.38
ACHN	73.25
CAKI-1	55.29
SN12C	80.28
TK-10	54.47
Prostate Cancer	
PC-3	94.86
DU-145	104.82
Breast Cancer	
MCF7	64.99
MDA-MB-231/ATCC	86.52
HS 578T	30.88
T-47D	45.66
Mean	70.00
Delta	92.26
Range	142.82

Mean Growth Percent - Growth Percent

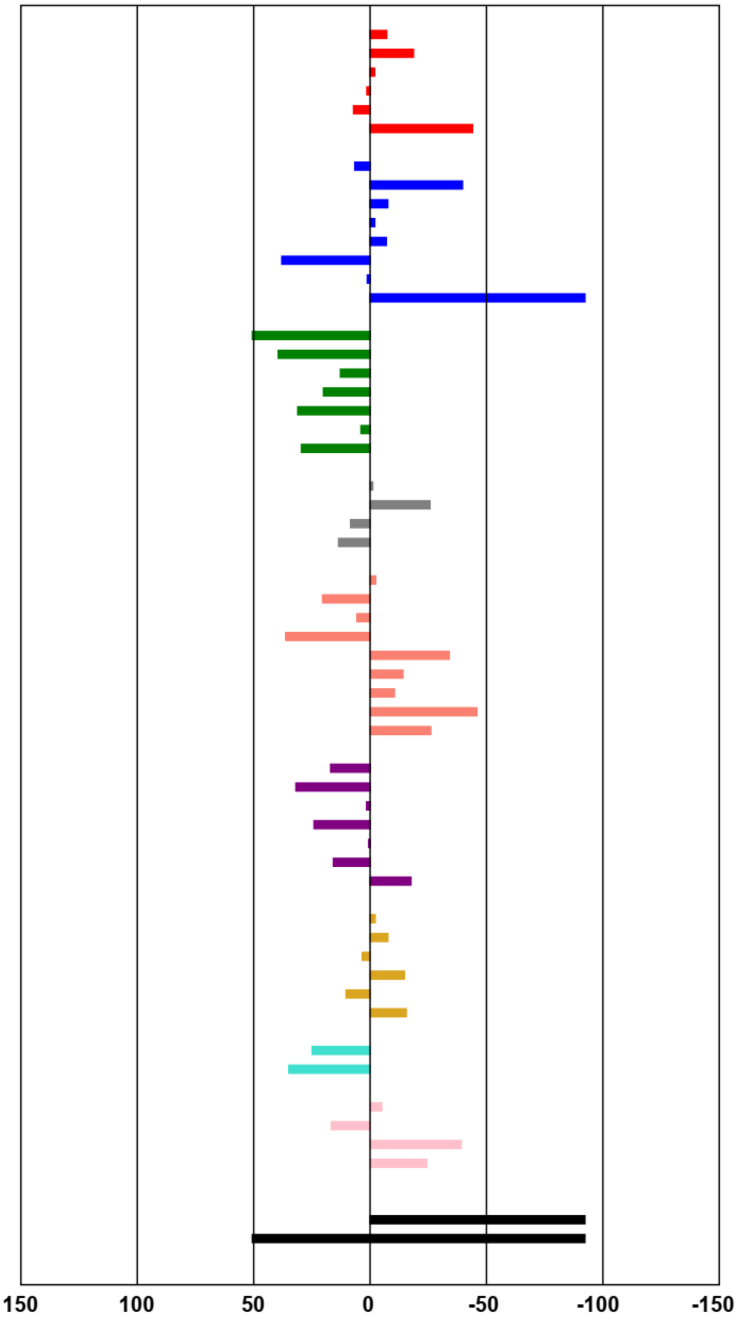

NCI screening results of compound **10a**

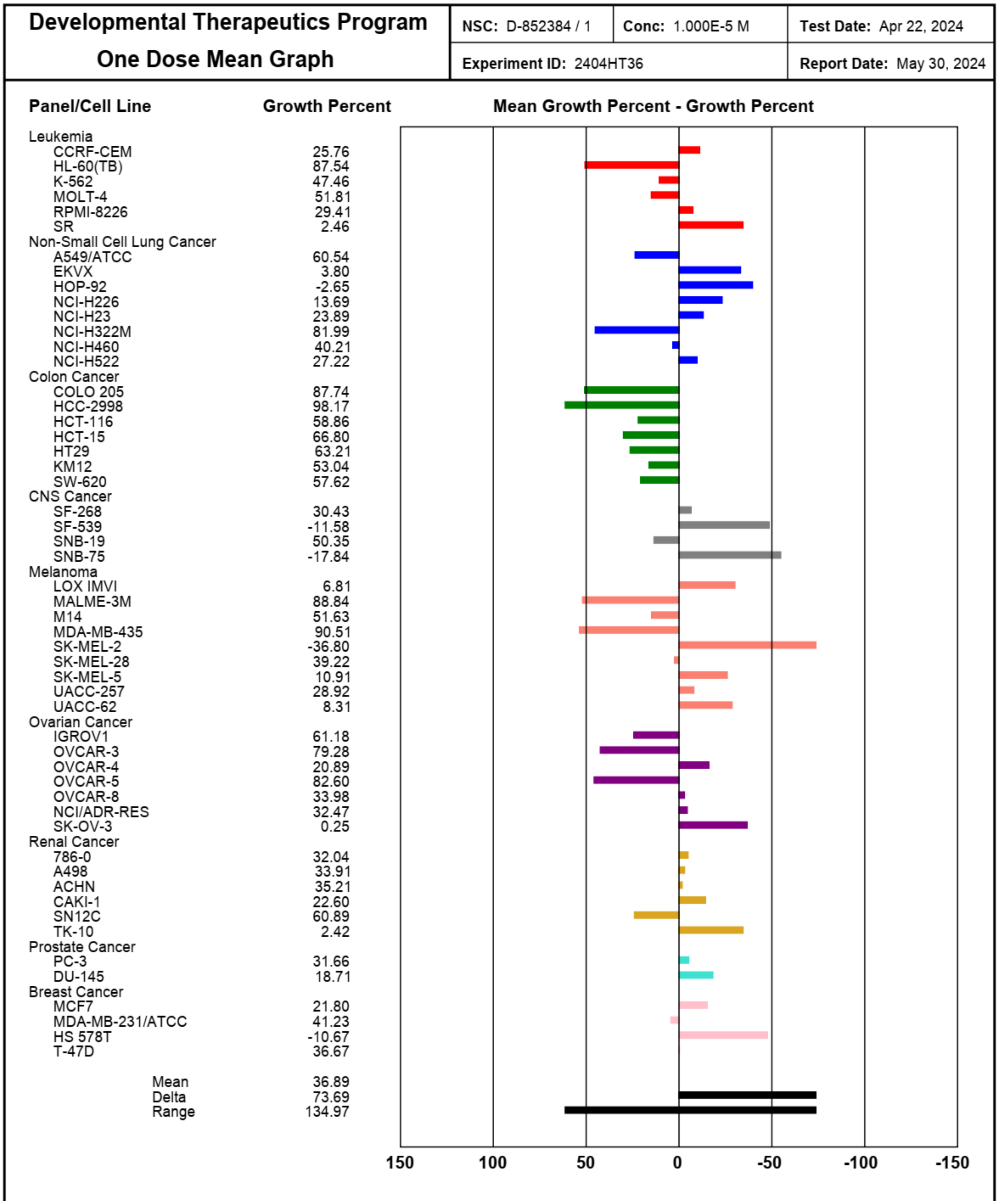

NCI screening results of compound **10b**

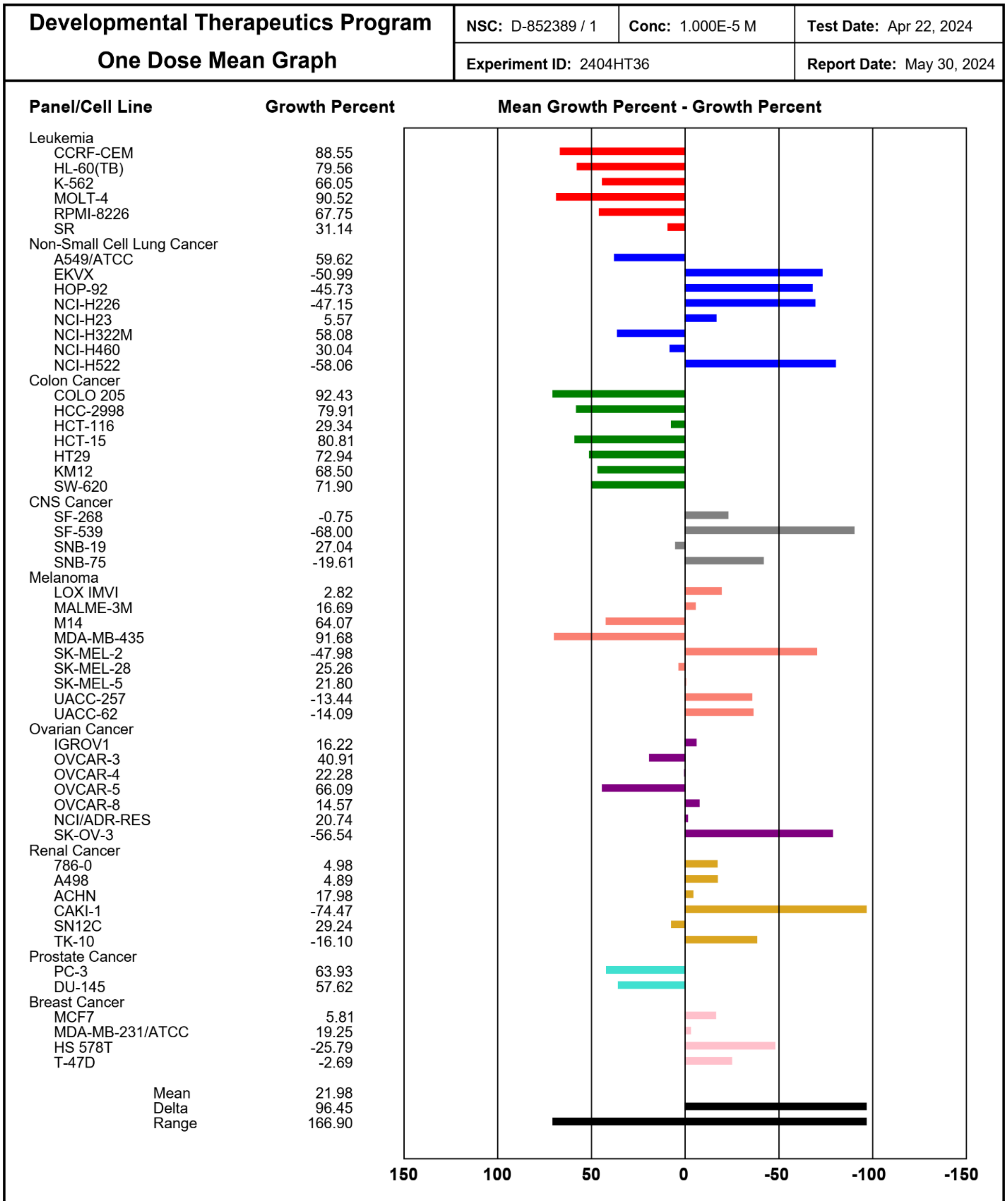

NCI screening results of compound **10c**

Developmental Therapeutics Program<br>One Dose Mean Graph	NSC: D-852387 / 1	Conc: 1.000E-5 M	Test Date: Apr 22, 2024
	Experiment ID: 2404HT36		Report Date: May 30, 2024

Panel/Cell Line	Growth Percent
Leukemia	
CCRF-CEM	-94.34
HL-60(TB)	-93.79
K-562	-41.60
MOLT-4	-93.56
RPMI-8226	-71.64
SR	-96.58
Non-Small Cell Lung Cancer	
A549/ATCC	38.06
EKVX	-15.98
HOP-92	33.21
NCI-H226	-3.14
NCI-H23	5.85
NCI-H322M	52.22
NCI-H460	1.11
NCI-H522	-69.53
Colon Cancer	
COLO 205	24.25
HCC-2998	28.51
HCT-116	20.67
HCT-15	22.83
HT29	-1.05
KM12	0.77
SW-620	16.06
CNS Cancer	
SF-268	18.05
SF-539	-37.05
SNB-19	26.43
SNB-75	-8.99
Melanoma	
LOX IMVI	14.38
MALME-3M	8.46
M14	-5.11
MDA-MB-435	-33.36
SK-MEL-2	-47.49
SK-MEL-28	17.21
SK-MEL-5	-46.76
UACC-257	-15.55
UACC-62	2.80
Ovarian Cancer	
IGROV1	23.72
OVCAR-3	-42.18
OVCAR-4	35.92
OVCAR-5	62.96
OVCAR-8	24.11
NCI/ADR-RES	-57.20
SK-OV-3	-2.84
Renal Cancer	
786-0	4.14
A498	17.79
ACHN	43.60
CAKI-1	25.45
SN12C	41.04
TK-10	26.81
Prostate Cancer	
PC-3	12.21
DU-145	25.16
Breast Cancer	
MCF7	2.31
MDA-MB-231/ATCC	43.67
HS 578T	-61.57
T-47D	-4.71
Mean	-4.23
Delta	92.35
Range	159.54

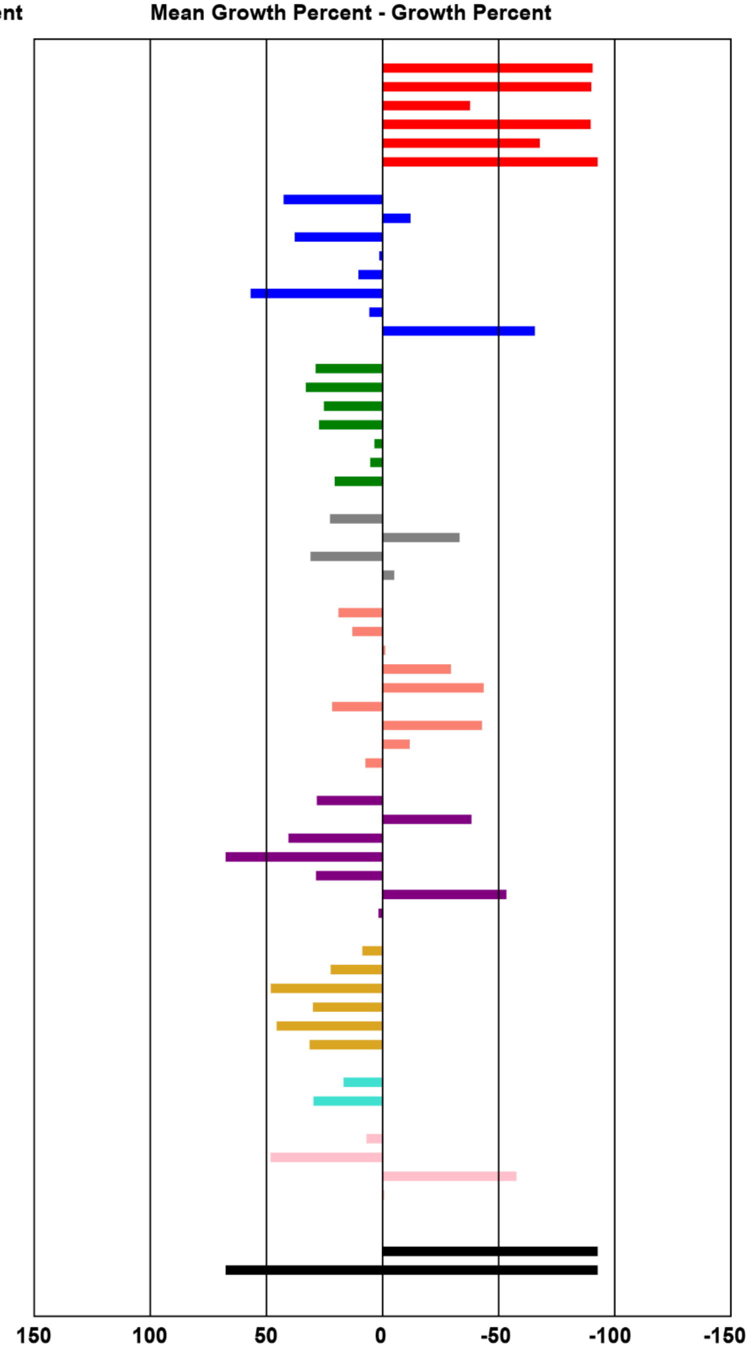

NCI screening results of compound **11a**

Developmental Therapeutics Program One Dose Mean Graph	NSC: D-852385 / 1	Conc: 1.000E-5 M	Test Date: Apr 22, 2024
	Experiment ID: 2404HT36		Report Date: May 30, 2024

Panel/Cell Line	Growth Percent
Leukemia	
CCRF-CEM	-91.70
HL-60(TB)	-93.71
K-562	-48.14
MOLT-4	-97.01
RPMI-8226	-80.78
SR	-97.63
Non-Small Cell Lung Cancer	
A549/ATCC	30.13
EKVX	-24.75
HOP-92	13.58
NCI-H226	-10.08
NCI-H23	-3.50
NCI-H322M	40.26
NCI-H460	-29.57
NCI-H522	-70.78
Colon Cancer	
COLO 205	29.90
HCC-2998	18.35
HCT-116	10.93
HCT-15	20.29
HT29	6.28
KM12	-32.88
SW-620	8.51
CNS Cancer	
SF-268	-6.88
SF-539	-45.43
SNB-19	21.84
SNB-75	-17.51
Melanoma	
LOX IMVI	1.19
MALME-3M	8.37
M14	-19.61
MDA-MB-435	-53.14
SK-MEL-2	-74.84
SK-MEL-28	2.65
SK-MEL-5	-61.03
UACC-257	-14.58
UACC-62	-25.23
Ovarian Cancer	
IGROV1	16.15
OVCAR-3	-38.48
OVCAR-4	15.22
OVCAR-5	51.60
OVCAR-8	9.16
NCI/ADR-RES	-71.88
SK-OV-3	-11.51
Renal Cancer	
786-0	-13.38
A498	8.09
ACHN	24.73
CAKI-1	10.44
SN12C	27.98
TK-10	3.11
Prostate Cancer	
PC-3	2.01
DU-145	21.79
Breast Cancer	
MCF7	-13.78
MDA-MB-231/ATCC	23.56
HS 578T	-63.23
T-47D	-15.38
Mean	-15.10
Delta	82.53
Range	149.23

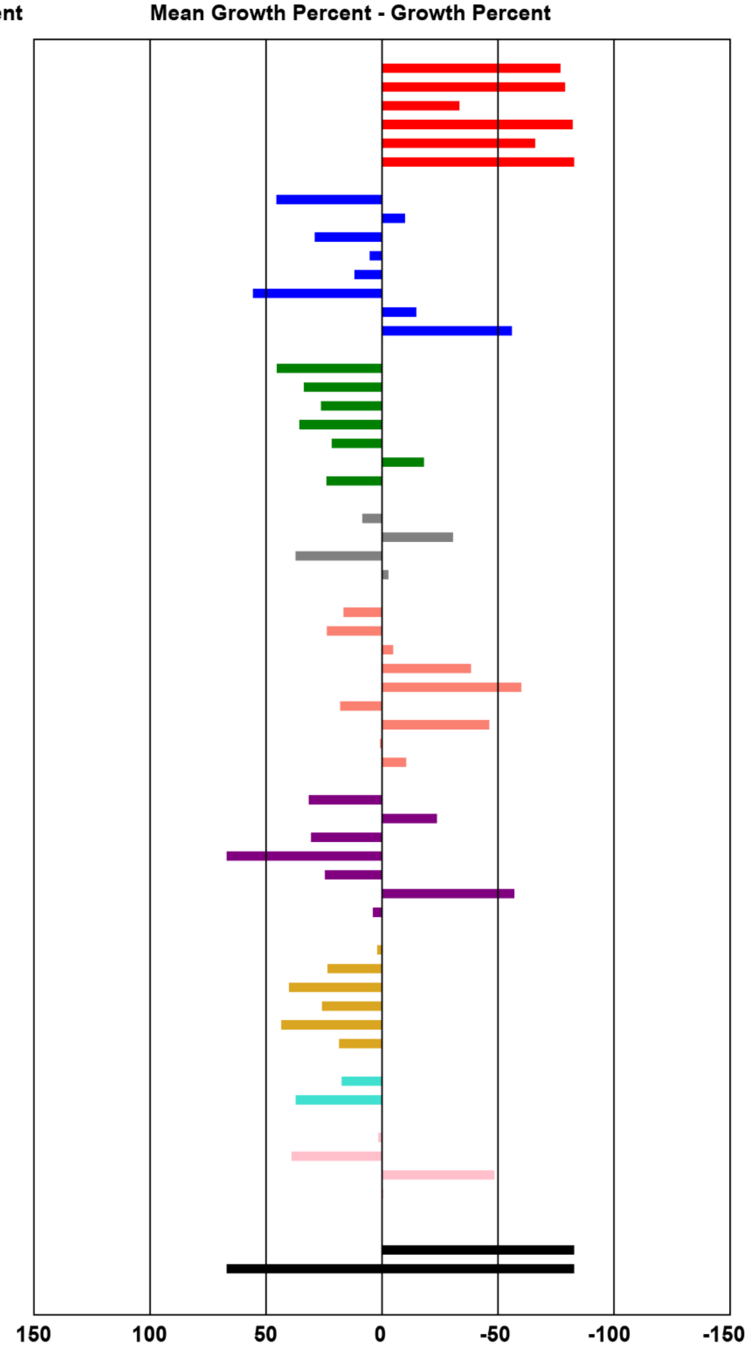

NCI screening results of compound **11b**

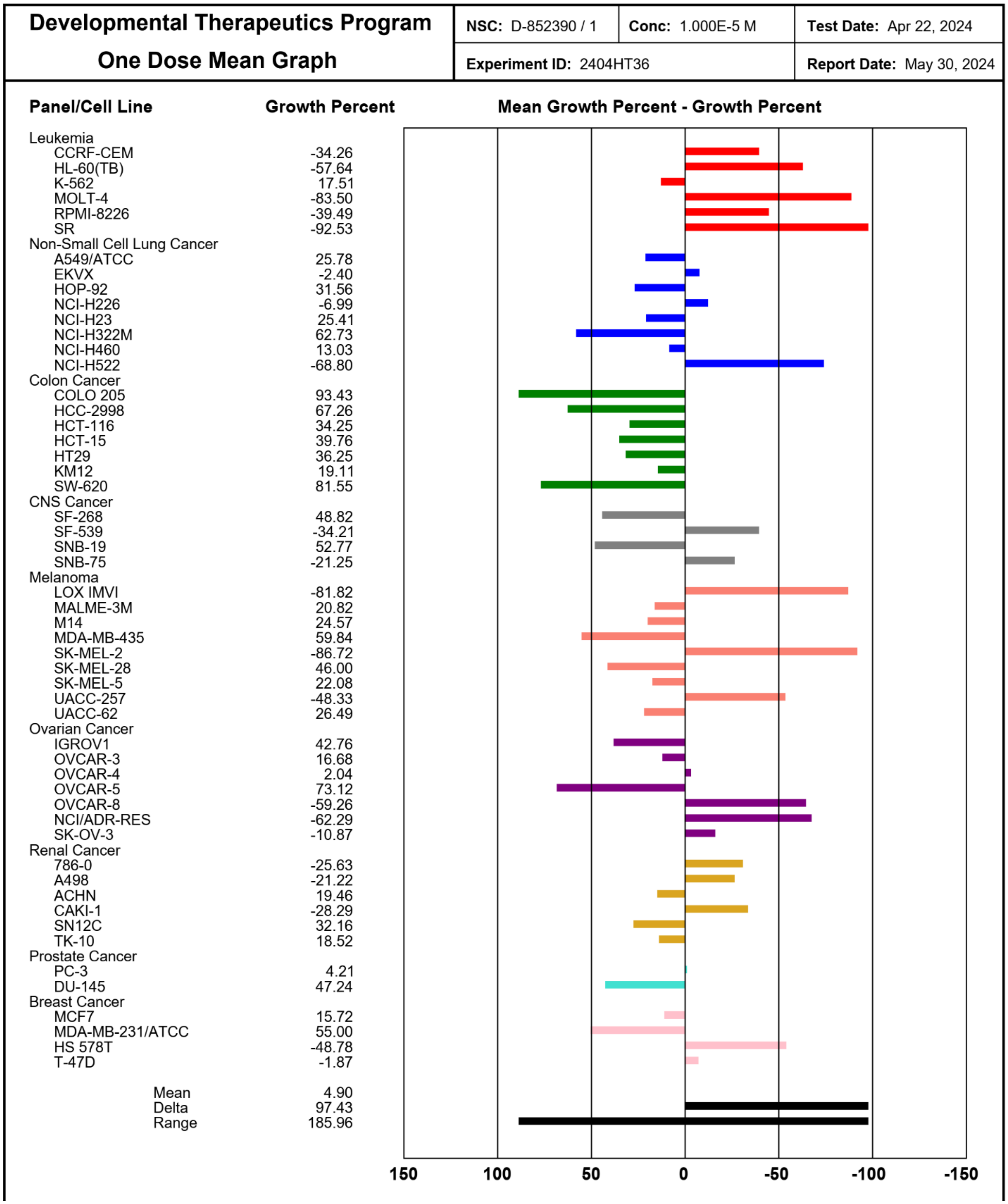

NCI screening results of compound **11c**

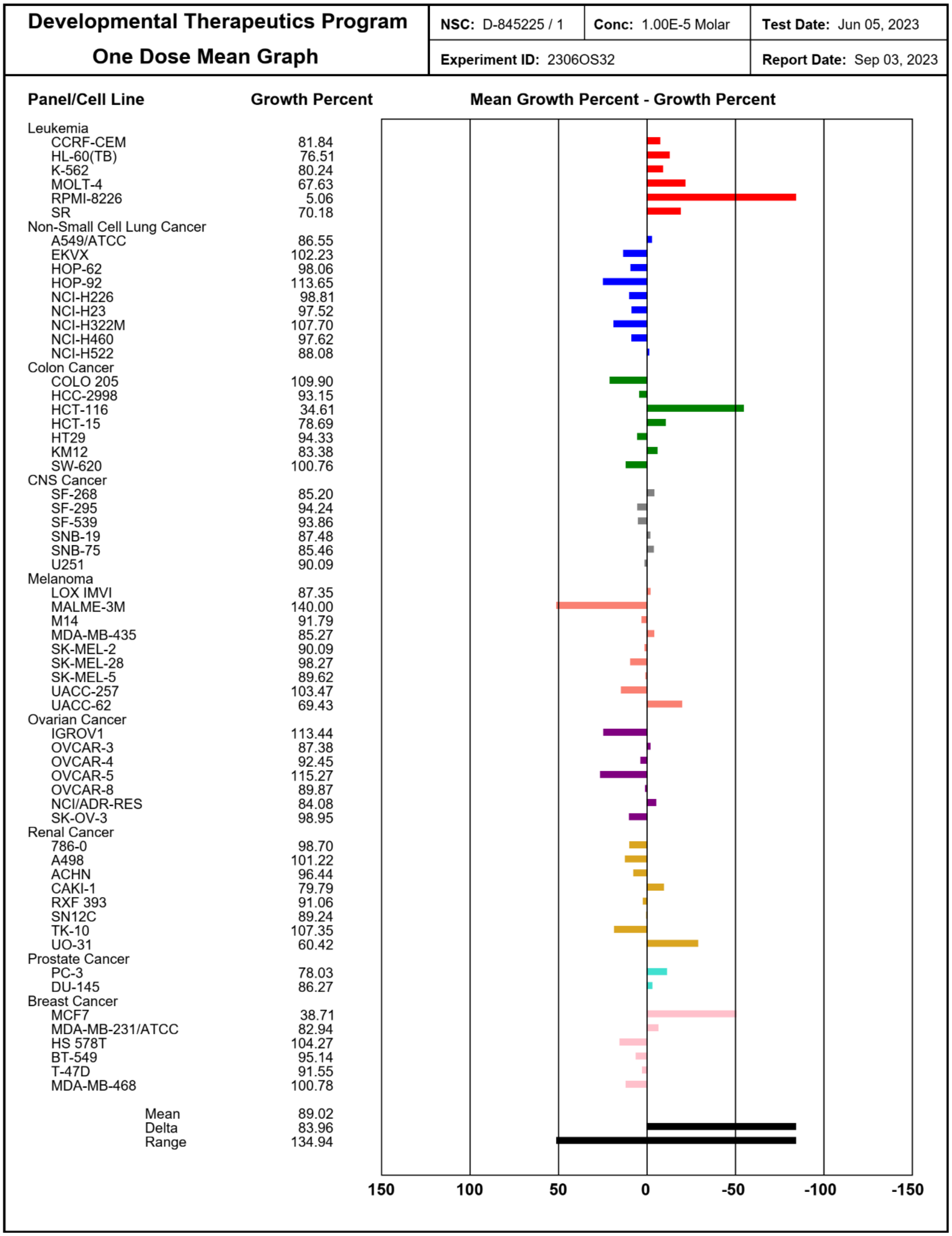

NCI screening results of compound **12a**

Developmental Therapeutics Program One Dose Mean Graph	NSC: D-846928 / 1	Conc: 1.00E-5 Molar	Test Date: Jul 24, 2023
	Experiment ID: 2307OS49		Report Date: Sep 03, 2023

Panel/Cell Line	Growth Percent
**Leukemia**	
CCRF-CEM	92.33
HL-60(TB)	91.11
K-562	82.40
MOLT-4	75.54
RPMI-8226	75.28
SR	79.13
**Non-Small Cell Lung Cancer**	
A549/ATCC	81.38
EKVX	96.61
HOP-62	98.49
HOP-92	103.00
NCI-H226	93.70
NCI-H23	88.40
NCI-H322M	108.98
NCI-H460	101.72
NCI-H522	83.13
**Colon Cancer**	
COLO 205	108.74
HCC-2998	98.96
HCT-116	74.27
HCT-15	92.58
HT29	107.02
KM12	94.32
SW-620	98.38
**CNS Cancer**	
SF-268	88.30
SF-295	100.24
SF-539	92.74
SNB-19	93.66
SNB-75	78.68
U251	86.03
**Melanoma**	
LOX IMVI	63.35
MALME-3M	104.98
M14	96.56
MDA-MB-435	93.62
SK-MEL-2	99.13
SK-MEL-28	99.61
SK-MEL-5	91.60
UACC-257	91.05
**Ovarian Cancer**	
IGROV1	100.02
OVCAR-3	95.09
OVCAR-4	96.96
OVCAR-5	102.02
OVCAR-8	89.81
NCI/ADR-RES	92.07
SK-OV-3	102.29
**Renal Cancer**	
786-0	110.47
A498	113.60
ACHN	96.04
CAKI-1	88.53
RXF 393	113.33
SN12C	90.74
TK-10	134.83
UO-31	70.33
**Prostate Cancer**	
PC-3	92.90
DU-145	92.71
**Breast Cancer**	
MCF7	70.42
MDA-MB-231/ATCC	81.52
HS 578T	92.30
BT-549	102.03
T-47D	87.66
MDA-MB-468	104.81
Mean	93.65
Delta	30.30
Range	71.48

Mean Growth Percent - Growth Percent

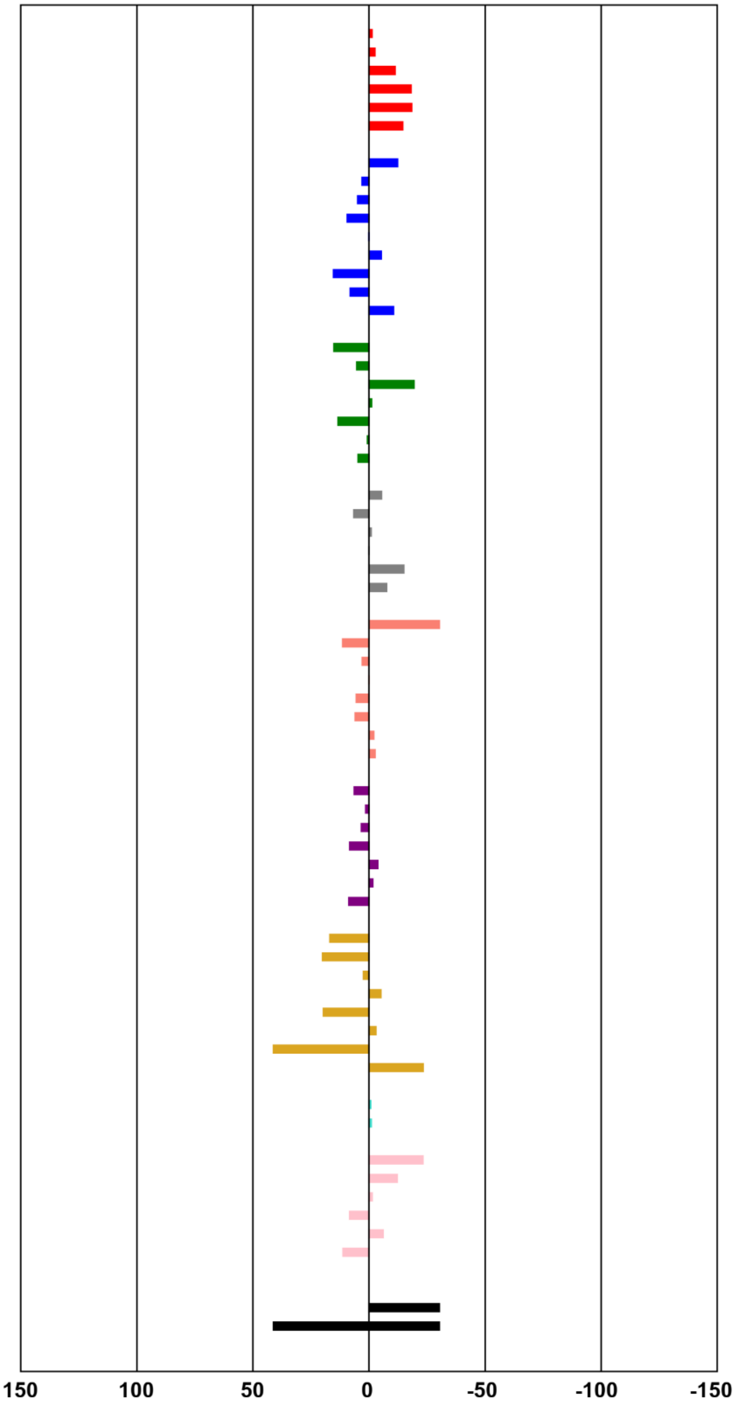

NCI screening results of compound **12b**

Developmental Therapeutics Program One Dose Mean Graph	NSC: D-849626 / 1	Conc: 1.00E-5 Molar	Test Date: Oct 23, 2023
	Experiment ID: 2310OS97		Report Date: Dec 29, 2023

Panel/Cell Line	Growth Percent
**Leukemia**	
CCRF-CEM	91.08
HL-60(TB)	88.39
K-562	78.83
MOLT-4	78.62
RPMI-8226	75.61
**Non-Small Cell Lung Cancer**	
A549/ATCC	101.71
EKVX	87.95
HOP-62	108.88
HOP-92	115.53
NCI-H226	69.70
NCI-H23	95.13
NCI-H322M	100.30
NCI-H460	104.82
NCI-H522	87.74
**Colon Cancer**	
COLO 205	117.25
HCC-2998	106.85
HCT-116	80.96
HCT-15	70.53
HT29	114.61
KM12	93.74
SW-620	110.37
**CNS Cancer**	
SF-268	104.00
SF-295	81.97
SF-539	87.83
SNB-19	87.24
SNB-75	97.66
U251	92.37
**Melanoma**	
LOX IMVI	70.03
MALME-3M	111.84
M14	105.35
MDA-MB-435	105.60
SK-MEL-2	101.61
SK-MEL-28	114.29
SK-MEL-5	94.22
UACC-257	106.58
UACC-62	73.45
**Ovarian Cancer**	
IGROV1	107.99
OVCAR-3	112.87
OVCAR-4	101.21
OVCAR-5	136.80
OVCAR-8	97.27
NCI/ADR-RES	97.93
SK-OV-3	107.49
**Renal Cancer**	
786-0	109.31
A498	117.62
ACHN	101.30
CAKI-1	96.91
RXF 393	113.13
SN12C	91.83
TK-10	127.35
UO-31	68.79
**Prostate Cancer**	
PC-3	78.78
DU-145	104.60
**Breast Cancer**	
MCF7	49.37
MDA-MB-231/ATCC	95.17
HS 578T	109.11
BT-549	96.30
T-47D	78.75
MDA-MB-468	90.48
Mean	96.66
Delta	47.29
Range	87.43

Mean Growth Percent - Growth Percent

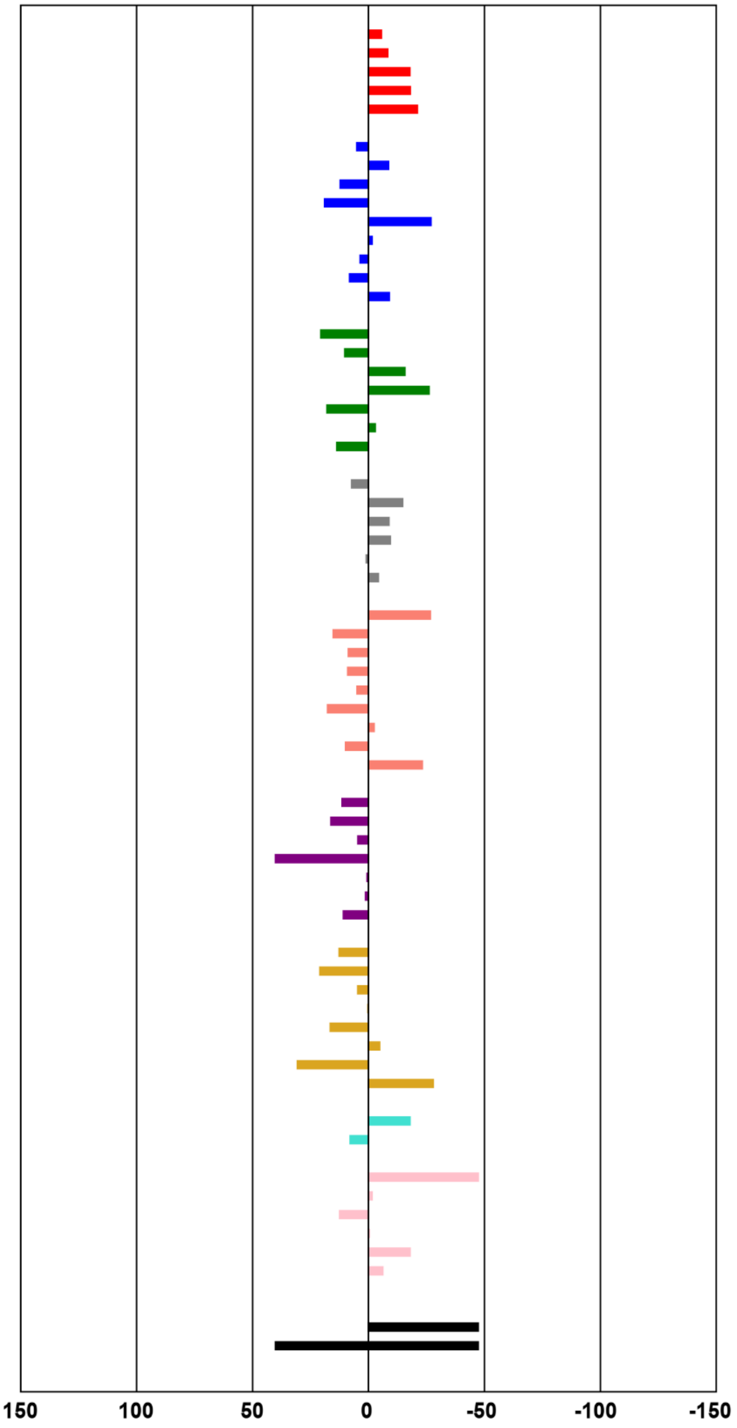

NCI screening results of compound **12c**

Developmental Therapeutics Program<br>One Dose Mean Graph	NSC: D-846930 / 1	Conc: 1.00E-5 Molar	Test Date: Jul 24, 2023
	Experiment ID: 2307OS49		Report Date: Sep 03, 2023

Panel/Cell Line	Growth Percent
**Leukemia**	
CCRF-CEM	44.75
HL-60(TB)	65.04
K-562	63.44
MOLT-4	31.75
RPMI-8226	54.02
SR	45.58
**Non-Small Cell Lung Cancer**	
A549/ATCC	75.38
EKVX	99.27
HOP-62	130.11
HOP-92	87.21
NCI-H226	95.98
NCI-H23	92.28
NCI-H322M	96.48
NCI-H460	105.56
NCI-H522	61.26
**Colon Cancer**	
COLO 205	111.91
HCC-2998	101.54
HCT-116	54.90
HCT-15	73.21
HT29	69.17
KM12	100.69
SW-620	104.75
**CNS Cancer**	
SF-268	122.62
SF-295	101.34
SF-539	93.67
SNB-19	87.57
SNB-75	113.94
U251	79.13
**Melanoma**	
LOX IMVI	70.57
MALME-3M	138.40
M14	105.63
MDA-MB-435	92.36
SK-MEL-2	76.37
SK-MEL-28	101.55
SK-MEL-5	94.29
UACC-257	88.66
**Ovarian Cancer**	
IGROV1	124.61
OVCAR-3	86.74
OVCAR-4	99.92
OVCAR-5	104.44
OVCAR-8	63.13
NCI/ADR-RES	87.83
SK-OV-3	139.84
**Renal Cancer**	
786-0	111.57
A498	109.69
ACHN	100.21
CAKI-1	90.74
RXF 393	117.96
SN12C	85.26
TK-10	111.53
UO-31	77.63
**Prostate Cancer**	
PC-3	76.68
DU-145	105.88
**Breast Cancer**	
MCF7	56.20
MDA-MB-231/ATCC	90.14
HS 578T	104.43
BT-549	92.34
T-47D	73.46
MDA-MB-468	112.94
Mean	90.74
Delta	58.99
Range	108.09

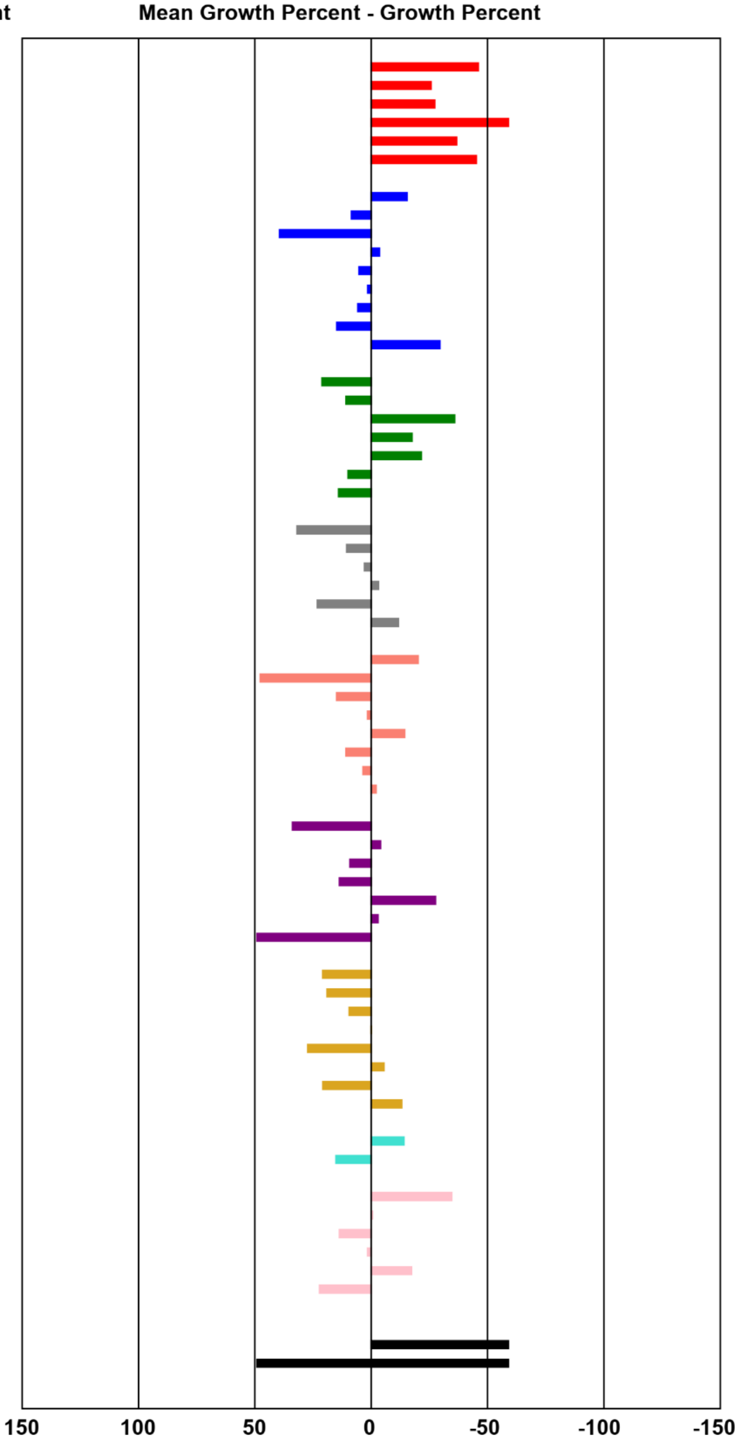

NCI screening results of compound **12d**

Developmental Therapeutics Program
One Dose Mean Graph

NSC: D-846927 / 1	Conc: 1.00E-5 Molar	Test Date: Jul 24, 2023
Experiment ID: 2307OS49		Report Date: Sep 03, 2023

Panel/Cell Line	Growth Percent
**Leukemia**	
CCRF-CEM	83.38
HL-60(TB)	88.60
K-562	51.15
MOLT-4	71.66
RPMI-8226	23.60
SR	54.28
**Non-Small Cell Lung Cancer**	
A549/ATCC	61.60
EKVX	89.30
HOP-62	98.93
HOP-92	92.50
NCI-H226	86.46
NCI-H23	91.01
NCI-H322M	109.73
NCI-H460	101.88
NCI-H522	78.75
**Colon Cancer**	
COLO 205	115.29
HCC-2998	95.48
HCT-116	-12.16
HCT-15	67.82
HT29	65.89
KM12	58.29
SW-620	37.70
**CNS Cancer**	
SF-268	75.22
SF-295	95.21
SF-539	84.72
SNB-19	80.18
SNB-75	68.46
U251	52.28
**Melanoma**	
LOX IMVI	36.58
MALME-3M	127.53
M14	92.61
MDA-MB-435	66.39
SK-MEL-2	105.89
SK-MEL-28	102.41
SK-MEL-5	91.36
UACC-257	98.23
**Ovarian Cancer**	
IGROV1	111.25
OVCAR-3	70.77
OVCAR-4	84.79
OVCAR-5	103.44
OVCAR-8	80.83
NCI/ADR-RES	82.85
SK-OV-3	102.29
**Renal Cancer**	
786-0	89.45
A498	102.04
ACHN	92.09
CAKI-1	77.31
RXF 393	92.02
SN12C	63.69
TK-10	125.98
UO-31	67.50
**Prostate Cancer**	
PC-3	69.32
DU-145	79.20
**Breast Cancer**	
MCF7	6.44
MDA-MB-231/ATCC	80.52
HS 578T	87.22
BT-549	90.66
T-47D	78.98
MDA-MB-468	98.32
Mean	80.09
Delta	92.25
Range	139.69

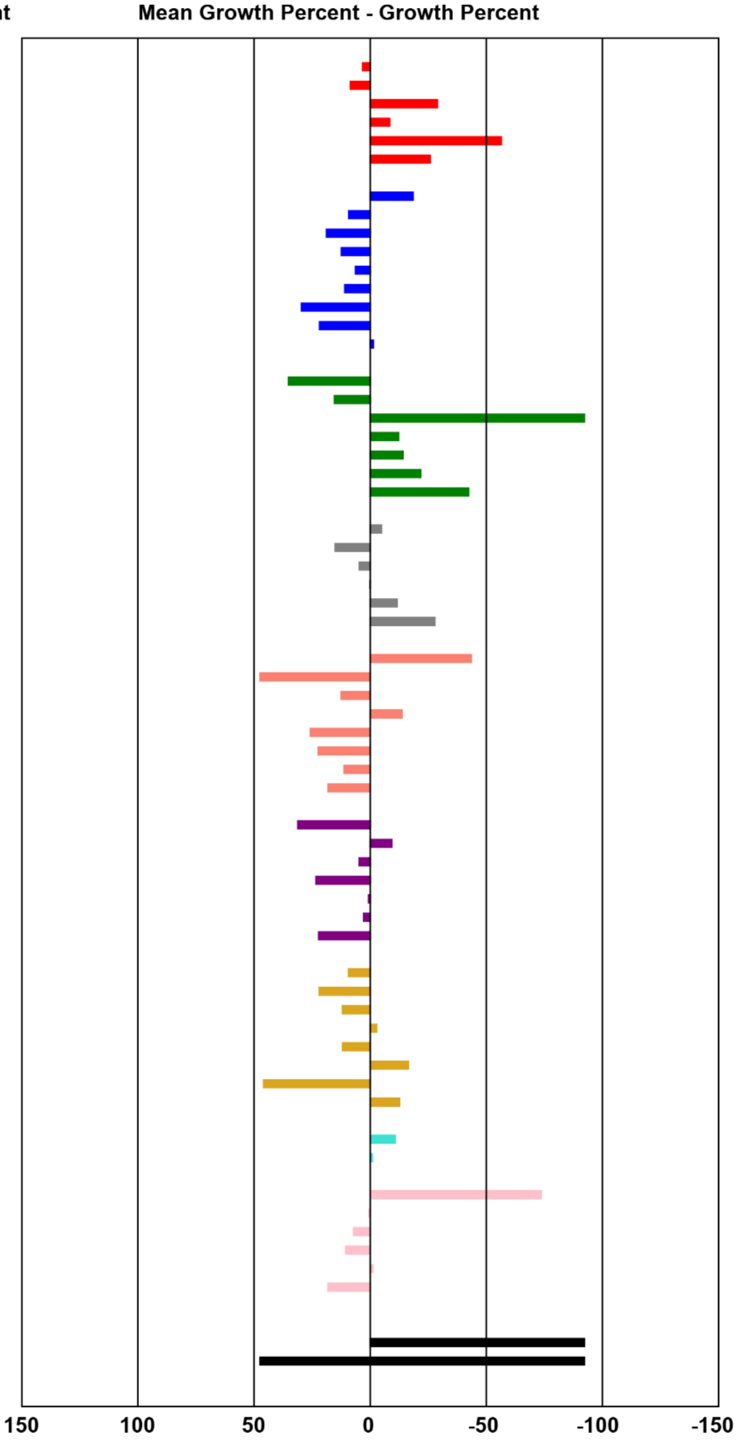

NCI screening results of compound **12e**

Developmental Therapeutics Program<br>One Dose Mean Graph	NSC: D-849625 / 1	Conc: 1.00E-5 Molar	Test Date: Oct 23, 2023
	Experiment ID: 2310OS97		Report Date: Dec 29, 2023

Panel/Cell Line	Growth Percent
Leukemia	
CCRF-CEM	90.17
HL-60(TB)	83.77
K-562	84.86
MOLT-4	66.24
RPMI-8226	23.62
Non-Small Cell Lung Cancer	
A549/ATCC	94.14
EKVX	69.54
HOP-62	89.74
HOP-92	75.85
NCI-H226	52.95
NCI-H23	55.14
NCI-H322M	97.52
NCI-H460	102.25
NCI-H522	74.56
Colon Cancer	
COLO 205	108.00
HCC-2998	94.93
HCT-116	37.26
HCT-15	63.83
HT29	102.22
KM12	96.69
SW-620	96.51
CNS Cancer	
SF-268	89.72
SF-295	15.28
SF-539	44.91
SNB-19	40.11
SNB-75	42.59
U251	65.04
Melanoma	
LOX IMVI	56.07
MALME-3M	85.92
M14	91.59
MDA-MB-435	87.07
SK-MEL-2	93.21
SK-MEL-28	104.35
SK-MEL-5	89.11
UACC-257	99.70
UACC-62	45.39
Ovarian Cancer	
IGROV1	99.53
OVCAR-3	118.89
OVCAR-4	91.07
OVCAR-5	134.85
OVCAR-8	81.02
NCI/ADR-RES	80.32
SK-OV-3	84.85
Renal Cancer	
786-0	62.68
A498	107.48
ACHN	78.79
CAKI-1	85.82
RXF 393	-4.52
SN12C	76.73
TK-10	99.45
UO-31	61.91
Prostate Cancer	
PC-3	84.91
DU-145	97.19
Breast Cancer	
MCF7	23.07
MDA-MB-231/ATCC	47.73
HS 578T	77.75
BT-549	75.01
T-47D	78.00
MDA-MB-468	40.62
Mean	76.15
Delta	80.67
Range	139.37

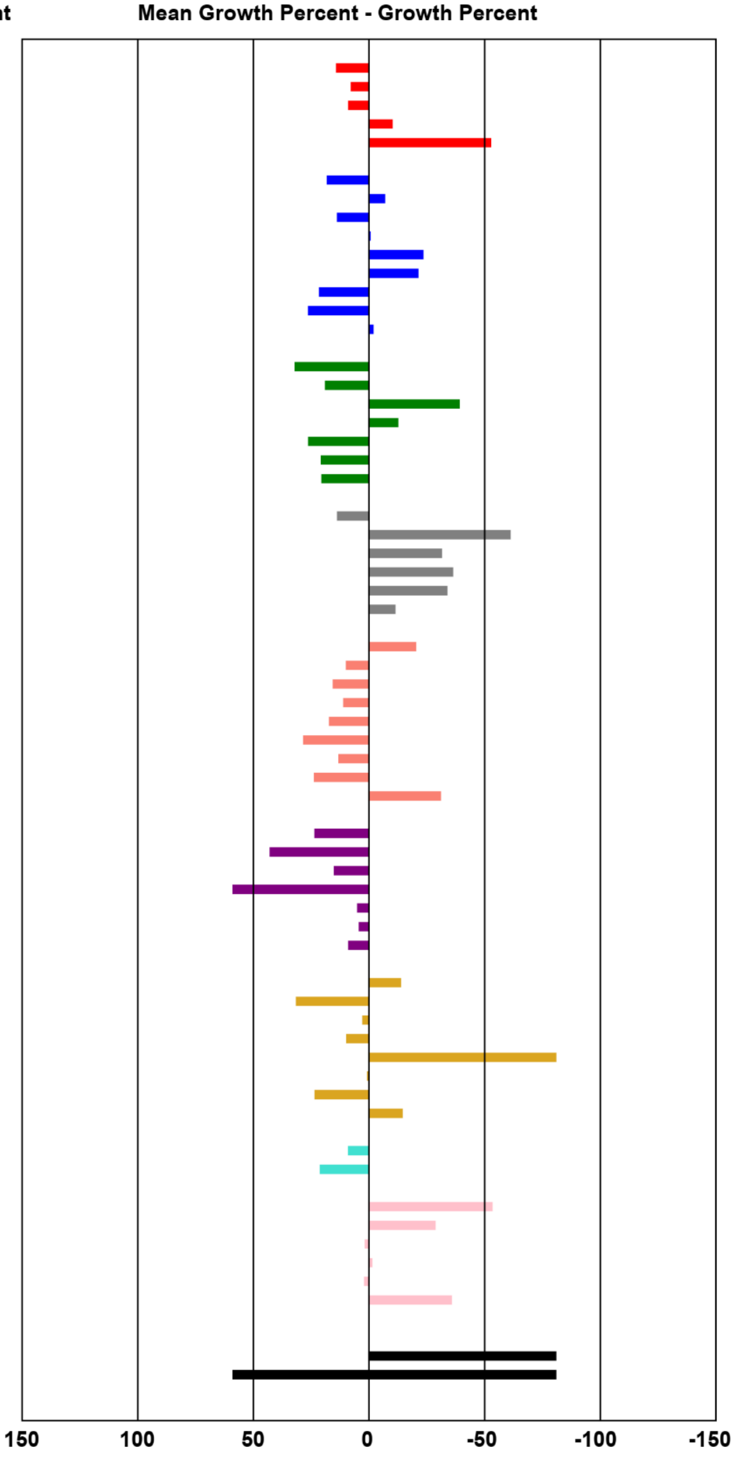

NCI screening results of compound **12f**

Developmental Therapeutics Program<br>One Dose Mean Graph	NSC: D-846929 / 1	Conc: 1.00E-5 Molar	Test Date: Jul 24, 2023
	Experiment ID: 2307OS49		Report Date: Sep 03, 2023

Panel/Cell Line	Growth Percent
**Leukemia**	
CCRF-CEM	122.33
HL-60(TB)	103.46
K-562	107.42
MOLT-4	94.25
RPMI-8226	113.02
SR	84.71
**Non-Small Cell Lung Cancer**	
A549/ATCC	95.64
EKVX	101.23
HOP-62	96.62
HOP-92	94.77
NCI-H226	97.09
NCI-H23	89.18
NCI-H322M	132.19
NCI-H460	117.31
NCI-H522	64.94
**Colon Cancer**	
COLO 205	119.54
HCC-2998	107.35
HCT-116	86.67
HCT-15	111.22
HT29	87.61
KM12	107.61
SW-620	113.82
**CNS Cancer**	
SF-268	91.14
SF-295	95.93
SF-539	92.29
SNB-19	82.09
SNB-75	121.35
U251	85.83
**Melanoma**	
LOX IMVI	84.97
MALME-3M	122.59
M14	121.23
MDA-MB-435	105.46
SK-MEL-2	82.15
SK-MEL-28	103.69
SK-MEL-5	99.20
UACC-257	96.10
**Ovarian Cancer**	
IGROV1	122.57
OVCAR-3	117.52
OVCAR-4	105.25
OVCAR-5	102.17
OVCAR-8	97.08
NCI/ADR-RES	93.77
SK-OV-3	86.52
**Renal Cancer**	
786-0	111.36
A498	114.23
ACHN	100.71
CAKI-1	104.84
RXF 393	121.96
SN12C	89.01
TK-10	117.13
UO-31	86.09
**Prostate Cancer**	
PC-3	91.36
DU-145	100.68
**Breast Cancer**	
MCF7	97.50
MDA-MB-231/ATCC	85.30
HS 578T	83.02
BT-549	96.83
T-47D	92.54
MDA-MB-468	115.93
Mean	101.11
Delta	36.17
Range	67.25

Mean Growth Percent - Growth Percent

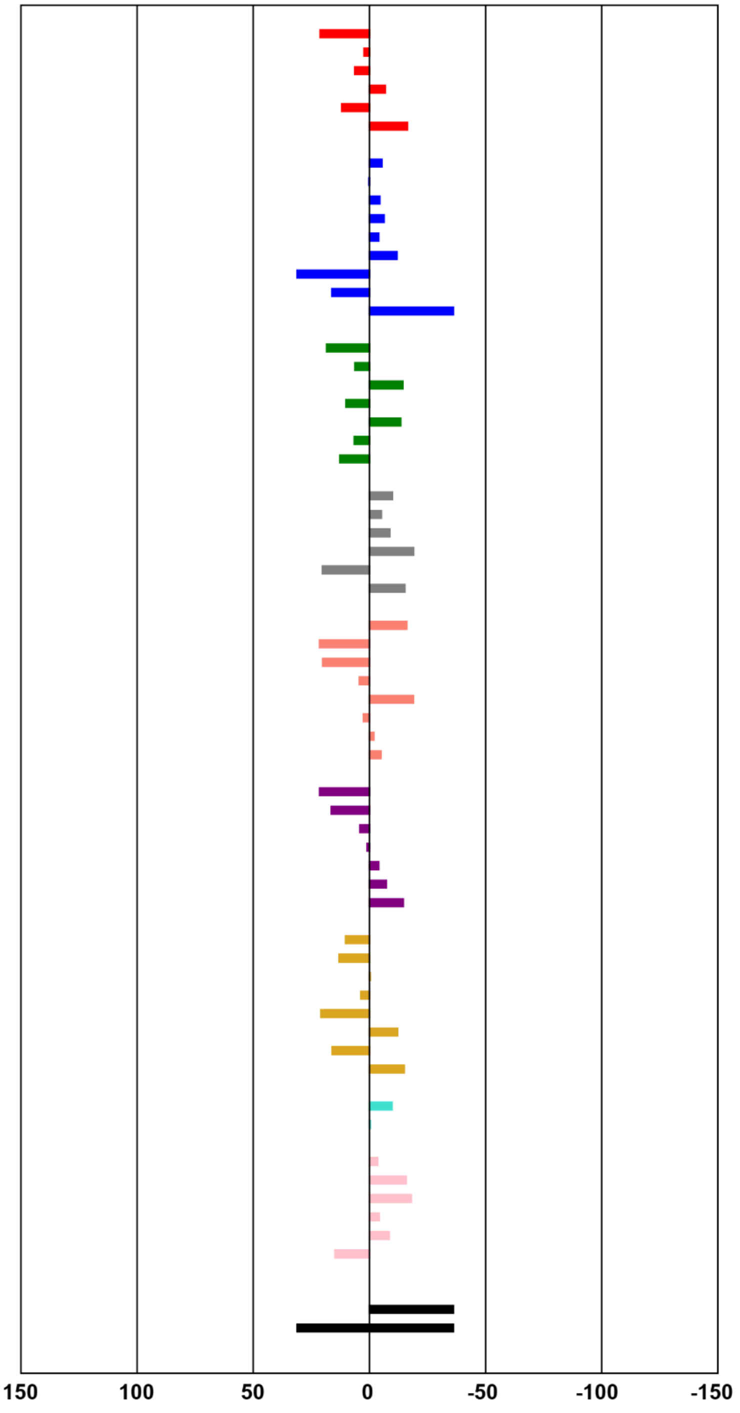

NCI screening results of compound **12g**

Developmental Therapeutics Program One Dose Mean Graph	NSC: D-845226 / 1	Conc: 1.00E-5 Molar	Test Date: Jun 05, 2023
	Experiment ID: 2306OS32		Report Date: Sep 03, 2023

Panel/Cell Line	Growth Percent
**Leukemia**	
CCRF-CEM	69.68
HL-60(TB)	76.85
K-562	74.77
MOLT-4	70.01
RPMI-8226	47.71
SR	72.40
**Non-Small Cell Lung Cancer**	
A549/ATCC	87.64
EKVX	100.51
HOP-62	105.44
HOP-92	104.36
NCI-H226	88.68
NCI-H23	94.75
NCI-H322M	112.90
NCI-H460	98.05
NCI-H522	87.91
**Colon Cancer**	
COLO 205	112.76
HCC-2998	98.86
HCT-116	62.97
HCT-15	80.49
HT29	91.82
KM12	87.18
SW-620	98.10
**CNS Cancer**	
SF-268	82.83
SF-295	99.09
SF-539	91.41
SNB-19	92.16
SNB-75	88.73
U251	85.81
**Melanoma**	
LOX IMVI	83.52
MALME-3M	121.40
M14	100.26
MDA-MB-435	90.67
SK-MEL-2	97.17
SK-MEL-28	113.16
SK-MEL-5	92.59
UACC-257	103.43
UACC-62	65.36
**Ovarian Cancer**	
IGROV1	126.34
OVCAR-3	90.60
OVCAR-4	97.58
OVCAR-5	114.30
OVCAR-8	85.45
NCI/ADR-RES	88.12
SK-OV-3	101.60
**Renal Cancer**	
786-0	99.09
A498	108.47
ACHN	91.19
CAKI-1	84.96
RXF 393	90.28
SN12C	88.18
TK-10	105.80
UO-31	60.54
**Prostate Cancer**	
PC-3	81.17
DU-145	93.42
**Breast Cancer**	
MCF7	48.76
MDA-MB-231/ATCC	83.73
HS 578T	101.64
BT-549	99.45
T-47D	97.84
MDA-MB-468	99.85
Mean	91.16
Delta	43.45
Range	78.63

Mean Growth Percent - Growth Percent

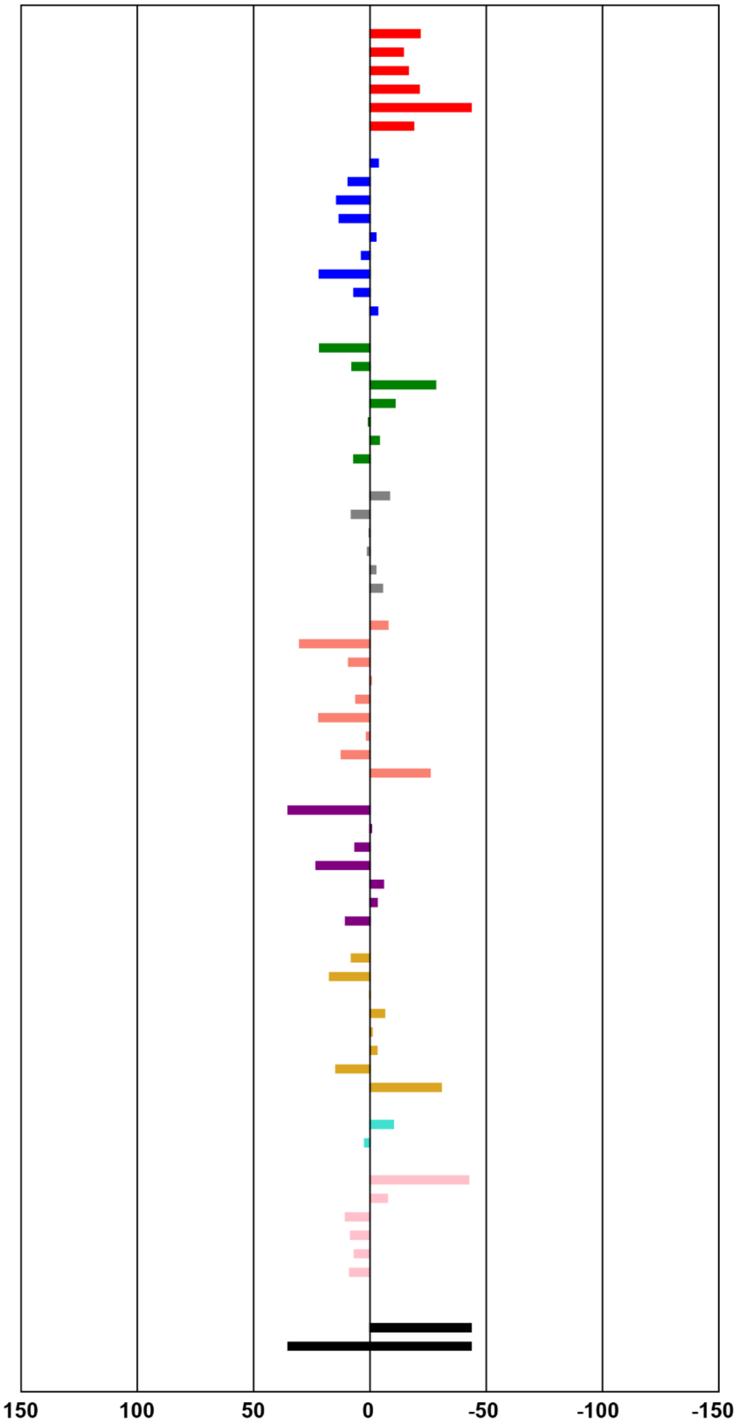

NCI screening results of compound **12h**

Developmental Therapeutics Program	NSC: D-845227 / 1	Conc: 1.00E-5 Molar	Test Date: Jun 05, 2023
One Dose Mean Graph	Experiment ID: 2306OS32		Report Date: Sep 03, 2023

Panel/Cell Line	Growth Percent
Leukemia	
CCRF-CEM	21.83
HL-60(TB)	92.05
K-562	63.41
MOLT-4	32.85
RPMI-8226	61.82
SR	26.19
Non-Small Cell Lung Cancer	
A549/ATCC	103.59
EKVX	96.60
HOP-62	105.01
HOP-92	126.59
NCI-H226	104.48
NCI-H23	90.83
NCI-H322M	118.86
NCI-H460	103.45
NCI-H522	81.26
Colon Cancer	
COLO 205	119.75
HCC-2998	106.91
HCT-116	76.69
HCT-15	31.48
HT29	95.87
KM12	95.37
SW-620	76.77
CNS Cancer	
SF-268	98.88
SF-295	100.31
SF-539	96.06
SNB-19	91.04
SNB-75	93.46
U251	95.62
Melanoma	
LOX IMVI	-69.52
MALME-3M	115.94
M14	77.16
MDA-MB-435	89.40
SK-MEL-2	107.02
SK-MEL-28	103.44
SK-MEL-5	91.12
UACC-257	120.02
UACC-62	72.83
Ovarian Cancer	
IGROV1	64.92
OVCAR-3	94.15
OVCAR-4	96.80
OVCAR-5	118.10
OVCAR-8	94.29
NCI/ADR-RES	102.13
SK-OV-3	107.05
Renal Cancer	
786-0	102.92
A498	121.67
ACHN	101.14
CAKI-1	100.13
RXF 393	100.37
SN12C	89.08
TK-10	110.36
UO-31	76.30
Prostate Cancer	
PC-3	84.43
DU-145	100.42
Breast Cancer	
MCF7	58.57
MDA-MB-231/ATCC	96.29
HS 578T	102.12
BT-549	85.49
T-47D	87.37
MDA-MB-468	117.56
Mean	88.77
Delta	158.29
Range	196.11

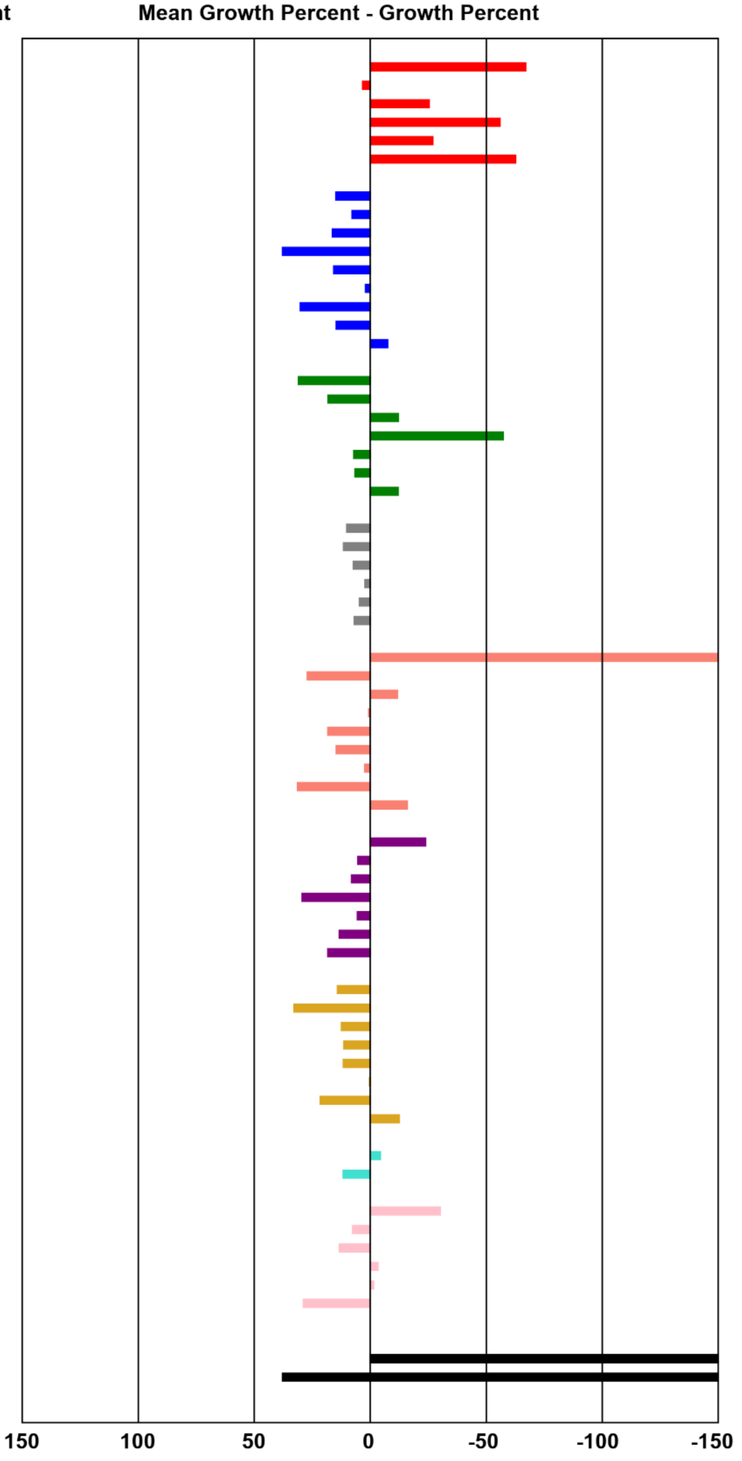

NCI screening results of compound **12i**

Developmental Therapeutics Program

One Dose Mean Graph

**NSC:** D-846925 / 1	**Conc:** 1.00E-5 Molar		**Test Date:** Jul 24, 2023
**Experiment ID:** 2307OS49			**Report Date:** Sep 03, 2023

Panel/Cell Line	Growth Percent
Leukemia	
CCRF-CEM	96.99
HL-60(TB)	105.92
K-562	94.39
MOLT-4	85.47
RPMI-8226	29.97
SR	73.15
Non-Small Cell Lung Cancer	
A549/ATCC	72.80
EKVX	96.55
HOP-62	106.61
HOP-92	83.63
NCI-H226	106.75
NCI-H23	81.77
NCI-H322M	105.94
NCI-H460	99.27
NCI-H522	63.64
Colon Cancer	
COLO 205	114.98
HCC-2998	108.34
HCT-116	27.34
HCT-15	87.89
HT29	89.75
KM12	94.10
SW-620	102.36
CNS Cancer	
SF-268	83.98
SF-295	91.84
SF-539	82.26
SNB-19	80.56
SNB-75	83.50
U251	67.32
Melanoma	
LOX IMVI	51.19
MALME-3M	119.89
M14	100.93
MDA-MB-435	100.90
SK-MEL-2	85.05
SK-MEL-28	103.48
SK-MEL-5	97.03
UACC-257	95.52
Ovarian Cancer	
IGROV1	104.98
OVCAR-3	97.06
OVCAR-4	103.29
OVCAR-5	109.78
OVCAR-8	91.97
NCI/ADR-RES	84.24
SK-OV-3	123.89
Renal Cancer	
786-0	101.66
A498	116.56
ACHN	104.19
CAKI-1	101.36
RXF 393	109.13
SN12C	79.29
TK-10	120.13
UO-31	84.72
Prostate Cancer	
PC-3	80.11
DU-145	90.65
Breast Cancer	
MCF7	45.70
MDA-MB-231/ATCC	70.85
HS 578T	73.82
BT-549	101.81
T-47D	99.17
MDA-MB-468	133.16
Mean	91.50
Delta	64.16
Range	105.82

Mean Growth Percent - Growth Percent

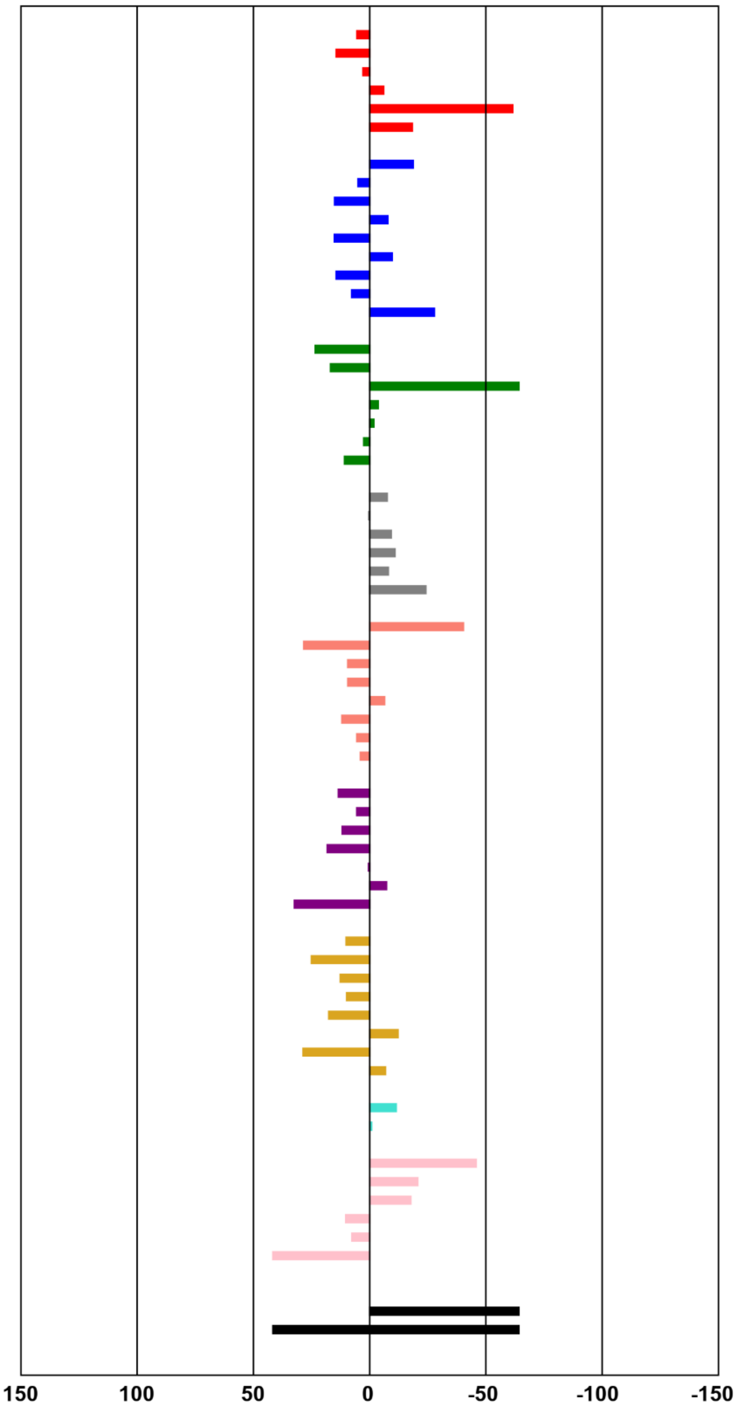

NCI screening results of compound **13a**

Developmental Therapeutics Program — One Dose Mean Graph

**NSC:** D-845222 / 1	**Conc:** 1.00E-5 Molar
**Test Date:** Jun 05, 2023	**Experiment ID:** 2306OS32
**Report Date:** Sep 03, 2023	

Panel/Cell Line	Growth Percent
**Leukemia**	
CCRF-CEM	84.91
HL-60(TB)	85.81
K-562	83.25
MOLT-4	66.04
RPMI-8226	79.55
SR	87.19
**Non-Small Cell Lung Cancer**	
A549/ATCC	90.22
EKVX	91.45
HOP-62	103.61
HOP-92	86.85
NCI-H226	103.86
NCI-H23	91.21
NCI-H322M	116.39
NCI-H460	99.23
NCI-H522	83.29
**Colon Cancer**	
COLO 205	114.70
HCC-2998	92.68
HCT-116	71.21
HCT-15	81.01
HT29	95.14
KM12	92.81
SW-620	104.57
**CNS Cancer**	
SF-268	88.38
SF-295	84.20
SF-539	92.82
SNB-19	90.13
SNB-75	69.09
U251	101.19
**Melanoma**	
LOX IMVI	93.76
MALME-3M	117.60
M14	85.59
MDA-MB-435	86.67
SK-MEL-2	89.67
SK-MEL-28	96.39
SK-MEL-5	85.89
UACC-257	95.99
UACC-62	66.27
**Ovarian Cancer**	
IGROV1	112.22
OVCAR-3	102.65
OVCAR-4	96.03
OVCAR-5	105.72
OVCAR-8	92.48
NCI/ADR-RES	83.46
SK-OV-3	114.98
**Renal Cancer**	
786-0	94.02
A498	96.14
ACHN	92.53
CAKI-1	73.91
RXF 393	80.64
SN12C	90.48
TK-10	98.53
UO-31	64.37
**Prostate Cancer**	
PC-3	74.55
DU-145	93.57
**Breast Cancer**	
MCF7	44.70
MDA-MB-231/ATCC	84.10
HS 578T	99.77
BT-549	77.33
T-47D	79.60
MDA-MB-468	94.63
Mean	89.92
Delta	45.22
Range	72.90

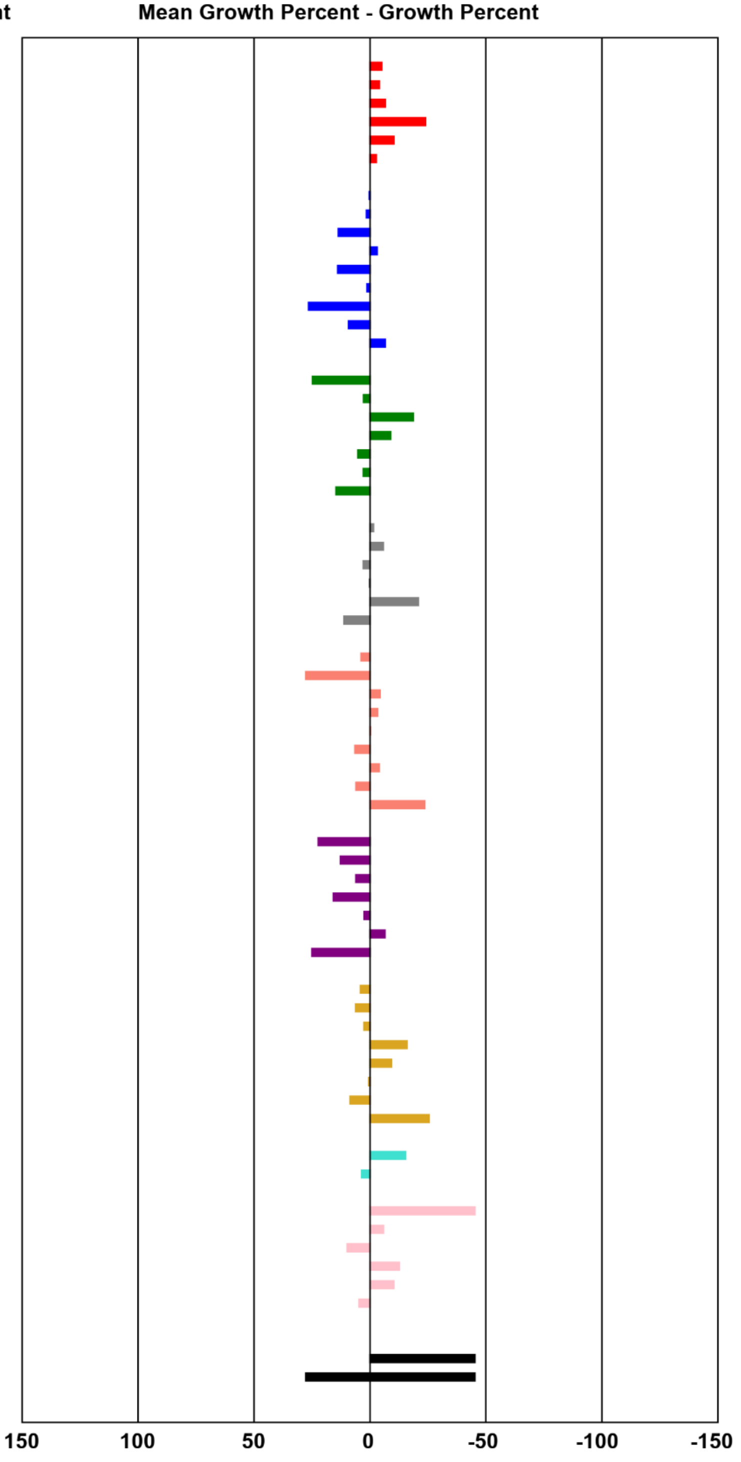

NCI screening results of compound **13b**

Developmental Therapeutics Program

One Dose Mean Graph

**NSC:** D-845221 / 1	**Conc:** 1.00E-5 Molar
**Test Date:** Jun 05, 2023	**Experiment ID:** 2306OS32
**Report Date:** Sep 03, 2023	

Panel/Cell Line	Growth Percent
Leukemia	
CCRF-CEM	73.80
HL-60(TB)	89.66
K-562	84.64
MOLT-4	73.41
RPMI-8226	74.86
SR	72.97
Non-Small Cell Lung Cancer	
A549/ATCC	91.11
EKVX	93.08
HOP-62	101.97
HOP-92	86.28
NCI-H226	96.20
NCI-H23	90.27
NCI-H322M	111.41
NCI-H460	99.02
NCI-H522	90.53
Colon Cancer	
COLO 205	108.17
HCC-2998	97.43
HCT-116	88.12
HCT-15	71.84
HT29	95.87
KM12	93.78
SW-620	100.85
CNS Cancer	
SF-268	81.64
SF-295	96.55
SF-539	93.53
SNB-19	89.80
SNB-75	91.76
U251	95.00
Melanoma	
LOX IMVI	90.72
MALME-3M	114.28
M14	94.22
MDA-MB-435	96.32
SK-MEL-2	90.57
SK-MEL-28	96.34
SK-MEL-5	78.63
UACC-257	95.04
UACC-62	67.24
Ovarian Cancer	
IGROV1	119.89
OVCAR-3	102.66
OVCAR-4	103.71
OVCAR-5	106.45
OVCAR-8	91.74
NCI/ADR-RES	83.08
SK-OV-3	101.65
Renal Cancer	
786-0	88.34
A498	94.40
ACHN	97.41
CAKI-1	85.73
RXF 393	89.78
SN12C	93.63
TK-10	104.99
UO-31	75.57
Prostate Cancer	
PC-3	79.42
DU-145	99.34
Breast Cancer	
MCF7	72.11
MDA-MB-231/ATCC	88.49
HS 578T	96.51
BT-549	80.12
T-47D	67.46
MDA-MB-468	93.97
Mean	91.22
Delta	23.98
Range	52.65

Mean Growth Percent - Growth Percent

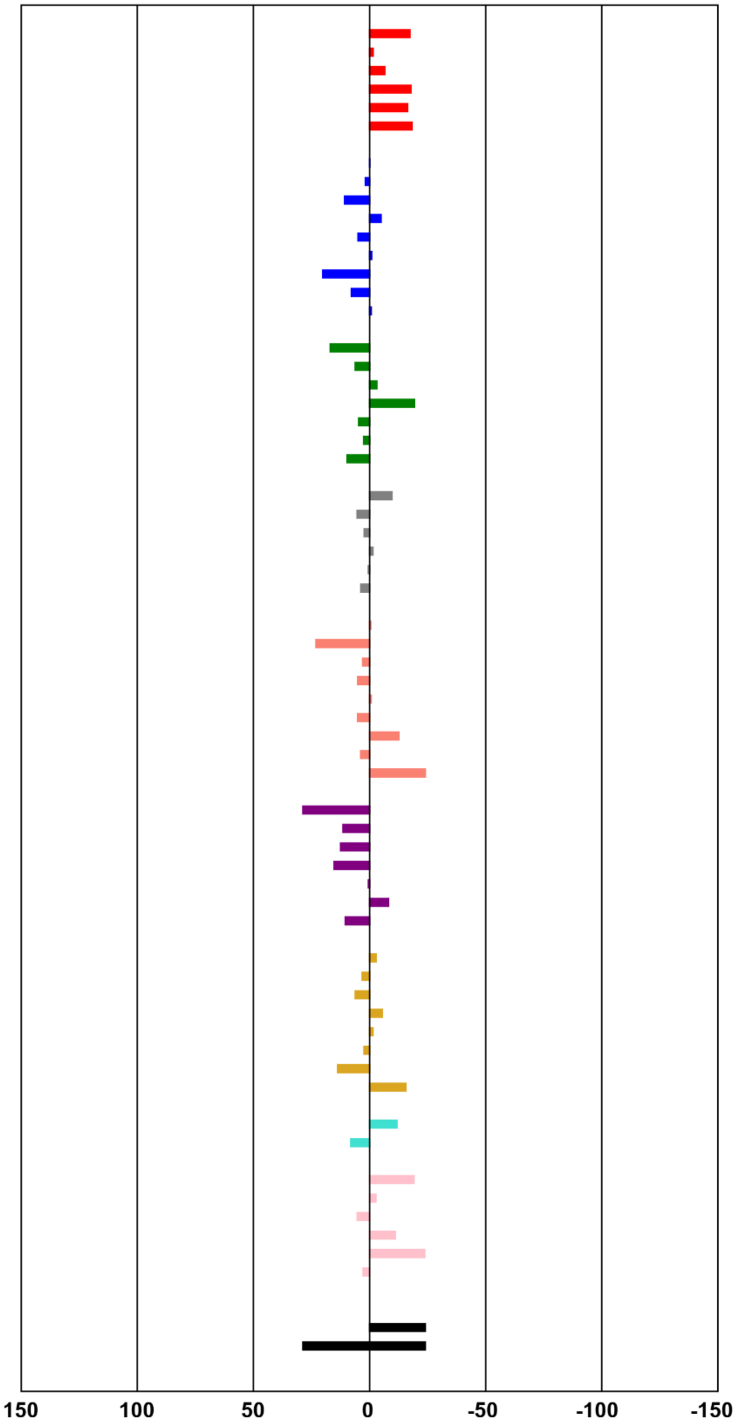

NCI screening results of compound **13c**

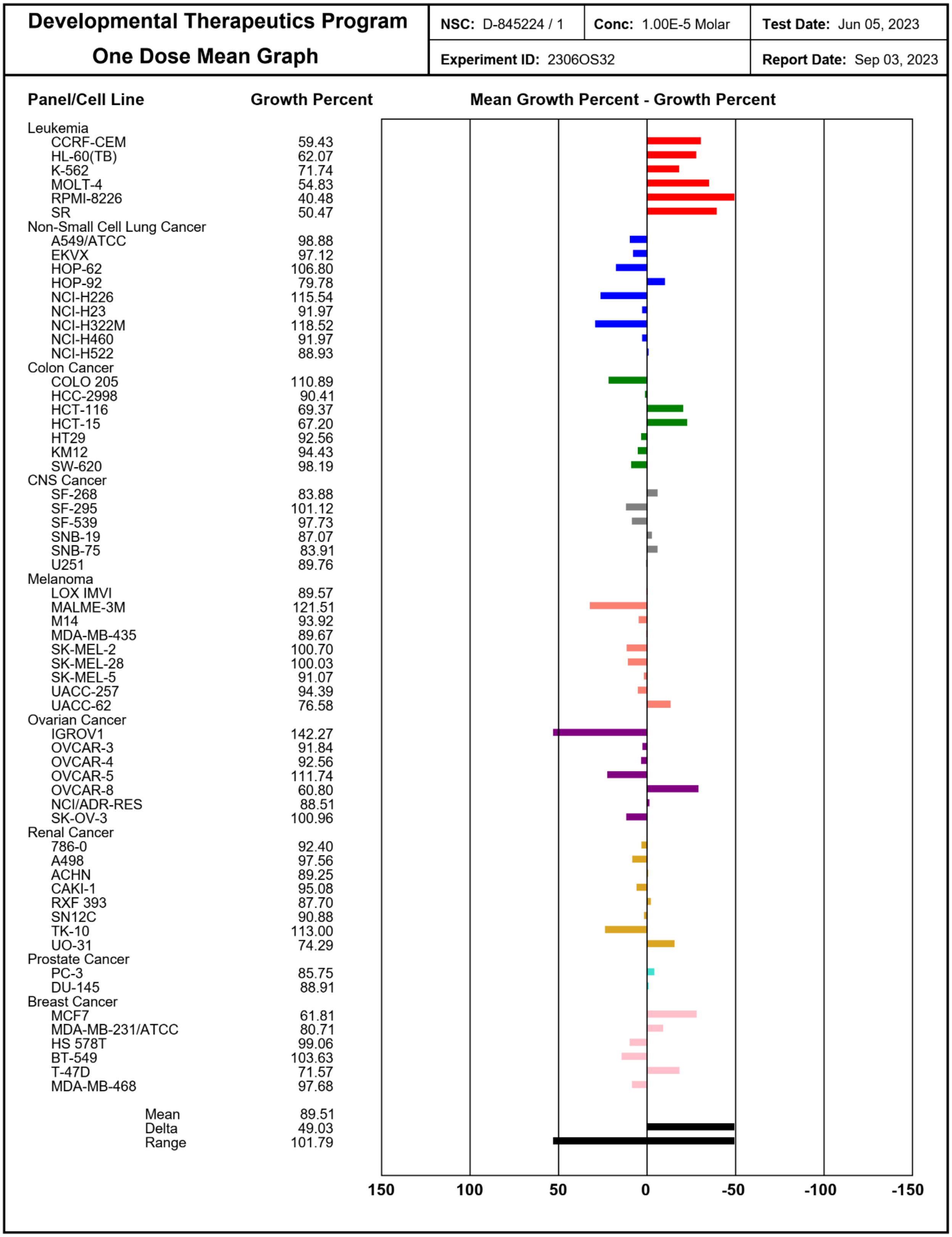

NCI screening results of compound **13d**

Developmental Therapeutics Program One Dose Mean Graph	NSC: D-846924 / 1	Conc: 1.00E-5 Molar	Test Date: Jul 24, 2023
	Experiment ID: 2307OS49		Report Date: Sep 03, 2023

Panel/Cell Line	Growth Percent
**Leukemia**	
CCRF-CEM	92.51
HL-60(TB)	107.40
K-562	95.60
MOLT-4	93.58
RPMI-8226	64.43
SR	84.21
**Non-Small Cell Lung Cancer**	
A549/ATCC	73.77
EKVX	93.94
HOP-62	114.49
HOP-92	102.96
NCI-H226	108.21
NCI-H23	79.07
NCI-H322M	95.22
NCI-H460	110.97
NCI-H522	69.94
**Colon Cancer**	
COLO 205	117.56
HCC-2998	102.77
HCT-116	53.18
HCT-15	97.15
HT29	89.72
KM12	101.38
SW-620	100.57
**CNS Cancer**	
SF-268	96.21
SF-295	98.23
SF-539	93.86
SNB-19	93.42
SNB-75	171.79
U251	85.02
**Melanoma**	
LOX IMVI	67.06
MALME-3M	136.55
M14	107.96
MDA-MB-435	97.25
SK-MEL-2	103.81
SK-MEL-28	102.50
SK-MEL-5	98.05
UACC-257	93.06
**Ovarian Cancer**	
IGROV1	106.22
OVCAR-3	105.83
OVCAR-4	108.29
OVCAR-5	113.28
OVCAR-8	88.13
NCI/ADR-RES	89.91
SK-OV-3	120.33
**Renal Cancer**	
786-0	99.34
A498	108.18
ACHN	101.38
CAKI-1	104.43
RXF 393	118.39
SN12C	84.77
TK-10	122.25
UO-31	80.48
**Prostate Cancer**	
PC-3	85.77
DU-145	99.04
**Breast Cancer**	
MCF7	38.63
MDA-MB-231/ATCC	84.46
HS 578T	107.02
BT-549	94.94
T-47D	102.45
MDA-MB-468	123.29
Mean	97.97
Delta	59.34
Range	133.16

Mean Growth Percent - Growth Percent

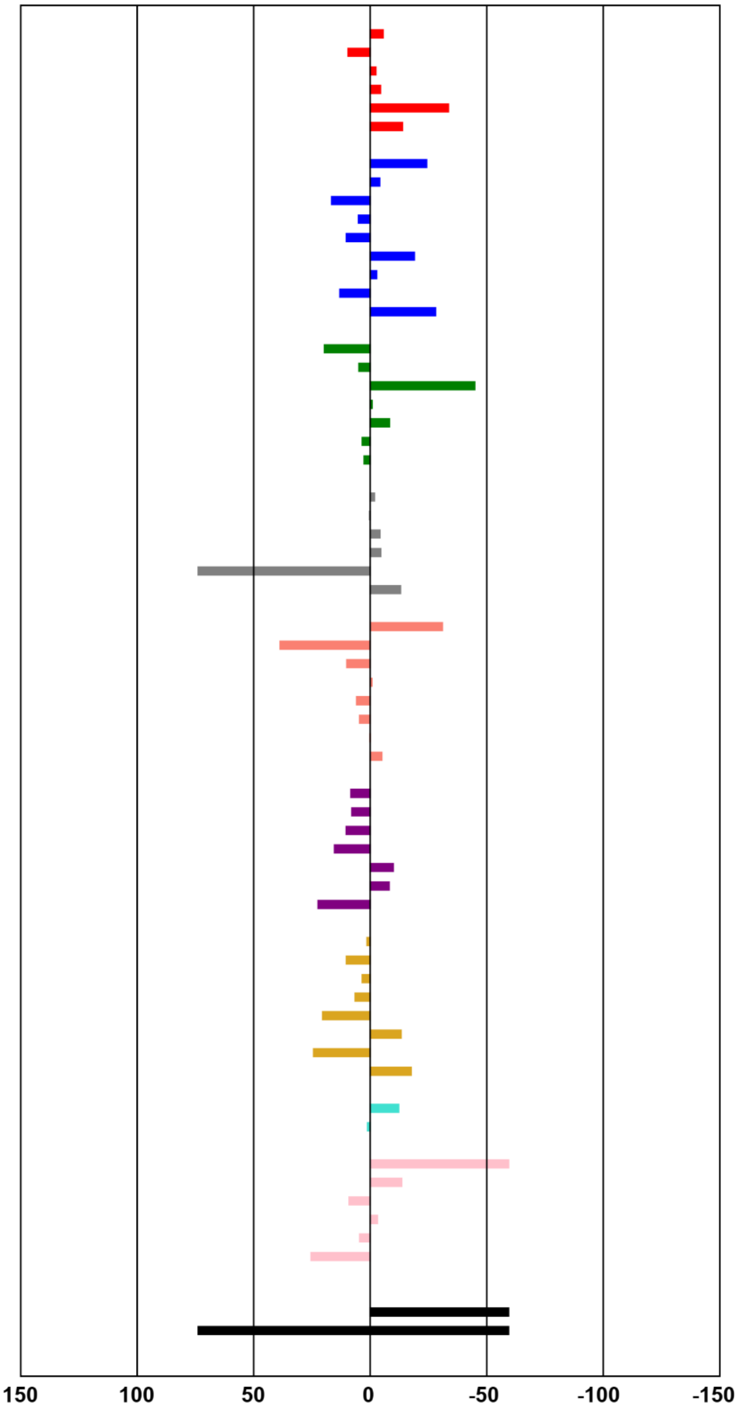

NCI screening results of compound **13e**

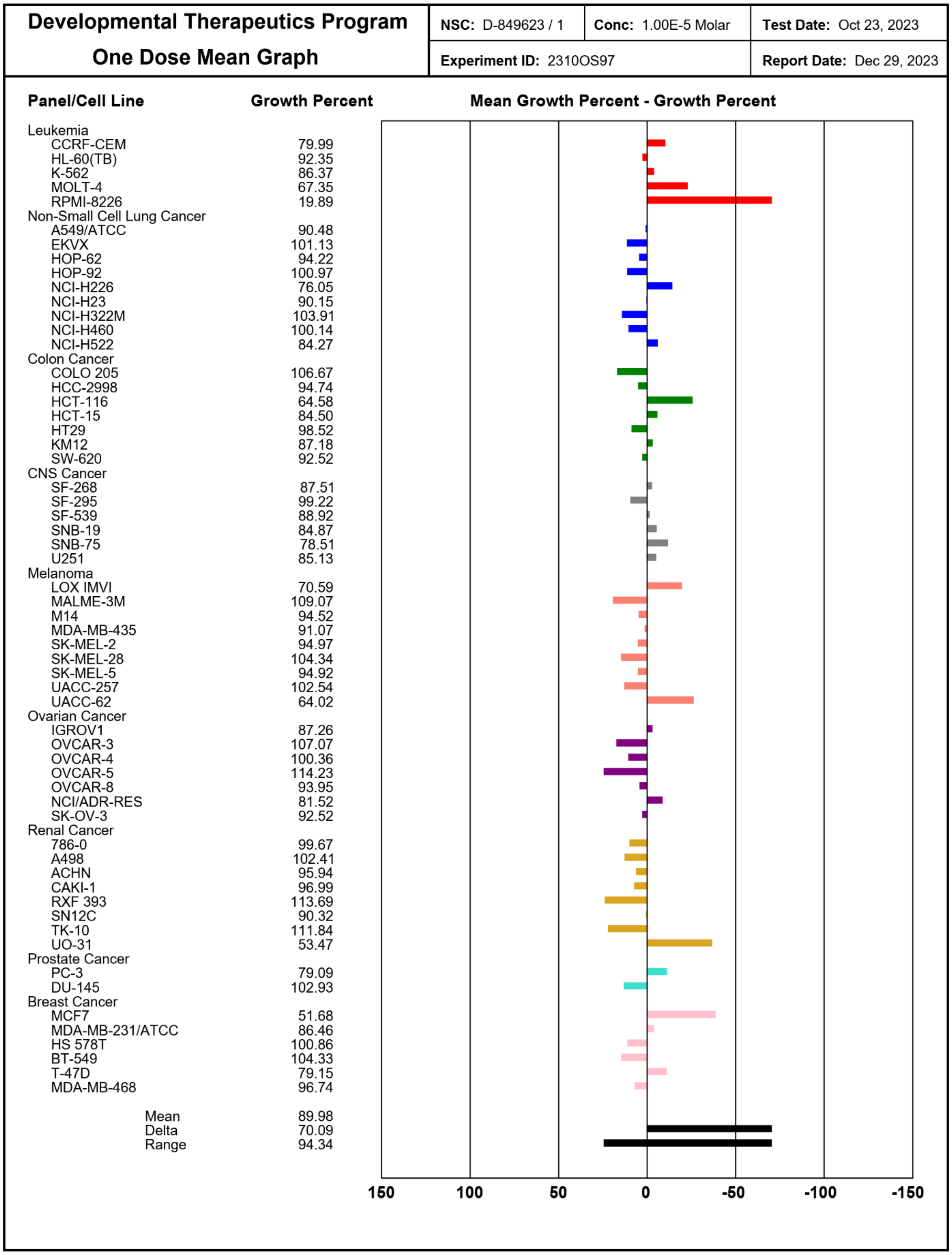

NCI screening results of compound **13f**

Developmental Therapeutics Program<br>One Dose Mean Graph	NSC: D-849624 / 1	Conc: 1.00E-5 Molar	Test Date: Oct 23, 2023
	Experiment ID: 2310OS97		Report Date: Dec 29, 2023

Panel/Cell Line	Growth Percent
**Leukemia**	
CCRF-CEM	84.29
HL-60(TB)	89.95
K-562	83.49
MOLT-4	72.47
RPMI-8226	36.34
**Non-Small Cell Lung Cancer**	
A549/ATCC	109.82
EKVX	71.14
HOP-62	99.27
HOP-92	104.04
NCI-H226	63.43
NCI-H23	80.82
NCI-H322M	107.84
NCI-H460	108.55
NCI-H522	83.82
**Colon Cancer**	
COLO 205	108.73
HCC-2998	106.02
HCT-116	29.89
HCT-15	73.52
HT29	98.39
KM12	99.37
SW-620	95.51
**CNS Cancer**	
SF-268	101.12
SF-295	74.81
SF-539	88.21
SNB-19	83.57
SNB-75	81.79
U251	83.84
**Melanoma**	
LOX IMVI	53.91
MALME-3M	103.47
M14	95.06
MDA-MB-435	97.33
SK-MEL-2	94.48
SK-MEL-28	108.32
SK-MEL-5	96.93
UACC-257	103.55
UACC-62	58.55
**Ovarian Cancer**	
IGROV1	80.80
OVCAR-3	118.35
OVCAR-4	104.56
OVCAR-5	118.95
OVCAR-8	100.70
NCI/ADR-RES	93.28
SK-OV-3	95.68
**Renal Cancer**	
786-0	102.04
A498	115.32
ACHN	84.30
CAKI-1	85.67
RXF 393	67.32
SN12C	83.67
TK-10	125.85
UO-31	60.34
**Prostate Cancer**	
PC-3	78.44
DU-145	105.99
**Breast Cancer**	
MCF7	34.63
MDA-MB-231/ATCC	72.26
HS 578T	103.59
BT-549	98.63
T-47D	76.17
MDA-MB-468	81.76
Mean	88.47
Delta	58.58
Range	95.96

Mean Growth Percent - Growth Percent

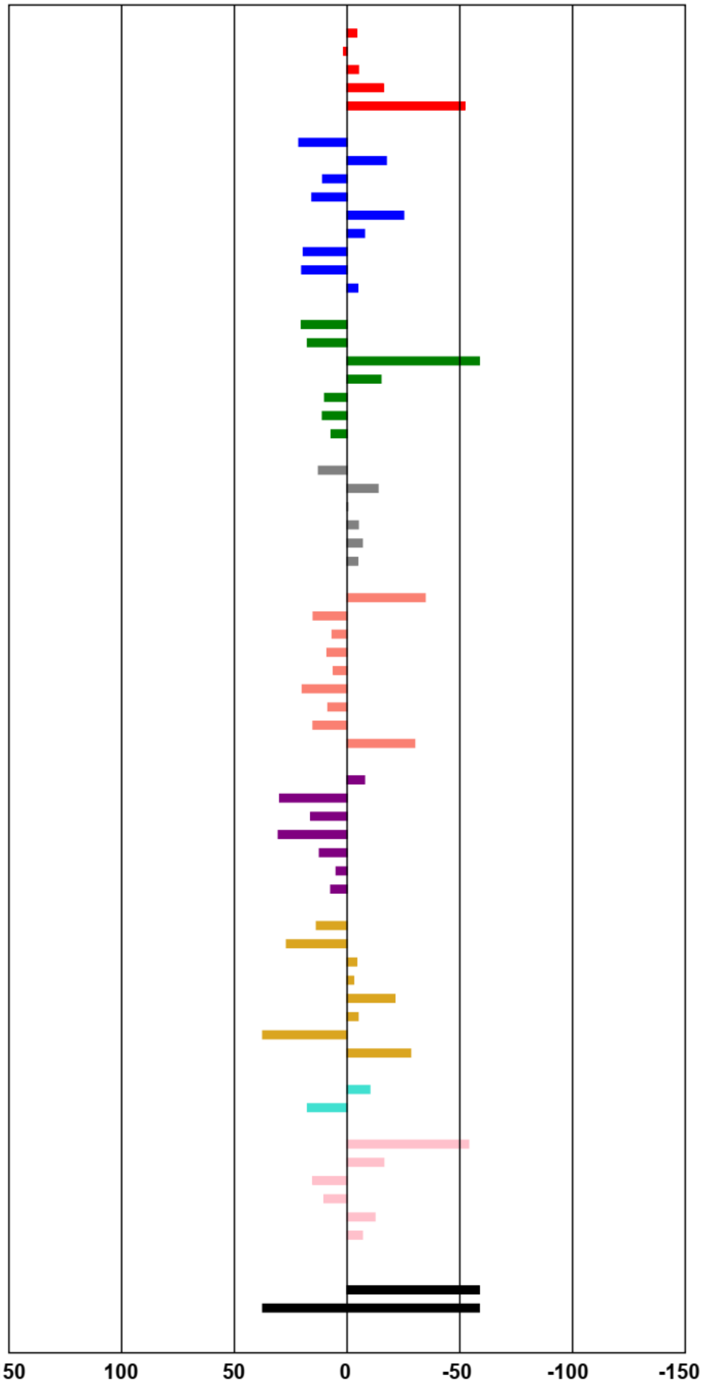

NCI screening results of compound **13g**

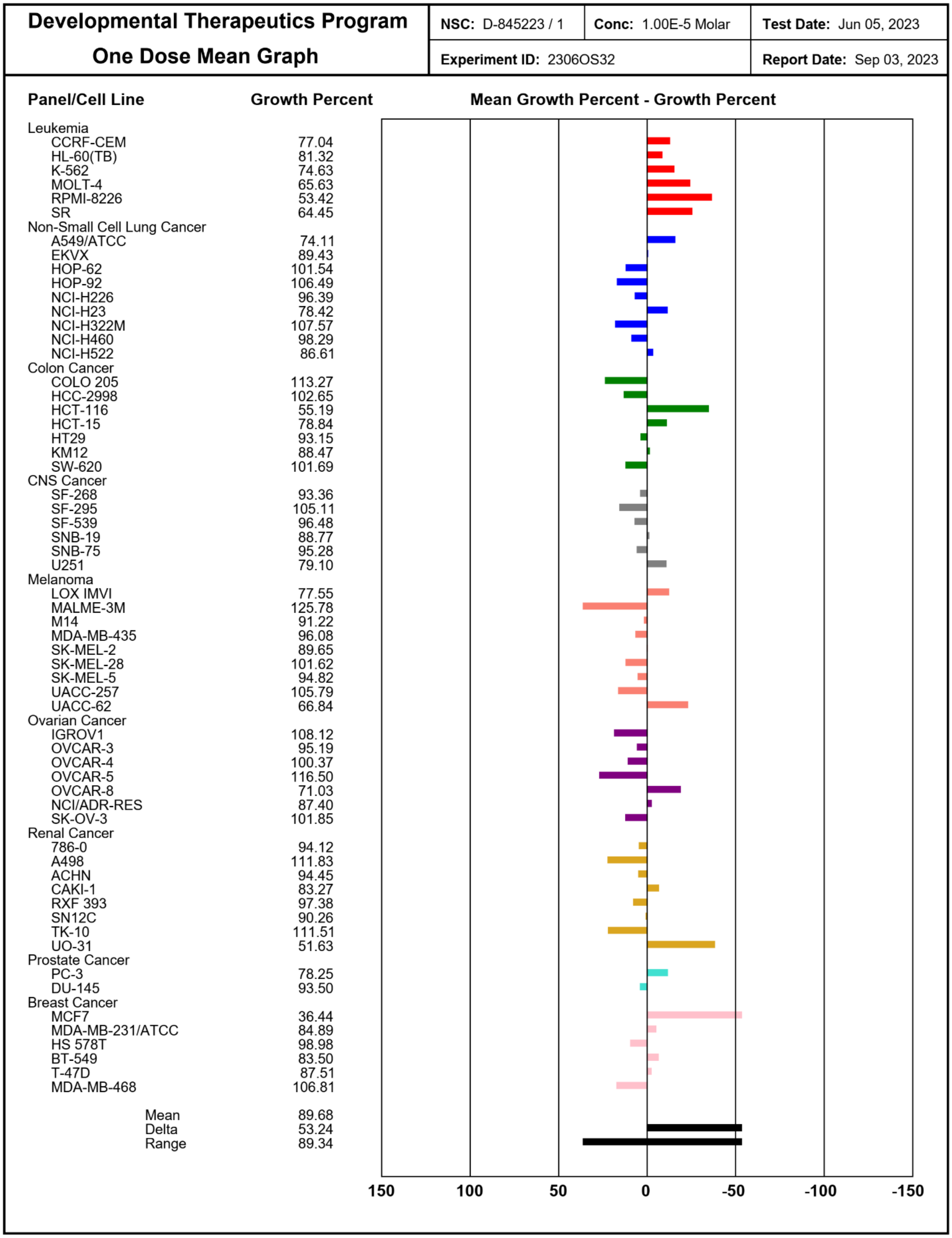

NCI screening results of compound **13h**

Developmental Therapeutics Program One Dose Mean Graph	NSC: D-846926 / 1	Conc: 1.00E-5 Molar	Test Date: Jul 24, 2023
	Experiment ID: 2307OS49		Report Date: Sep 03, 2023

Panel/Cell Line	Growth Percent
**Leukemia**	
CCRF-CEM	77.62
HL-60(TB)	106.02
K-562	92.67
MOLT-4	72.00
RPMI-8226	83.70
SR	66.72
**Non-Small Cell Lung Cancer**	
A549/ATCC	84.52
EKVX	106.24
HOP-62	121.31
HOP-92	104.58
NCI-H226	101.68
NCI-H23	98.02
NCI-H322M	97.96
NCI-H460	104.06
NCI-H522	79.43
**Colon Cancer**	
COLO 205	115.87
HCC-2998	93.32
HCT-116	90.27
HCT-15	84.60
HT29	96.55
KM12	99.67
SW-620	101.30
**CNS Cancer**	
SF-268	93.23
SF-295	103.60
SF-539	104.38
SNB-19	97.73
SNB-75	105.91
U251	85.88
**Melanoma**	
LOX IMVI	-7.63
MALME-3M	122.33
M14	100.41
MDA-MB-435	98.48
SK-MEL-2	95.97
SK-MEL-28	107.65
SK-MEL-5	97.20
UACC-257	94.41
**Ovarian Cancer**	
IGROV1	62.16
OVCAR-3	95.70
OVCAR-4	102.17
OVCAR-5	110.32
OVCAR-8	96.43
NCI/ADR-RES	97.34
SK-OV-3	122.03
**Renal Cancer**	
786-0	111.06
A498	108.48
ACHN	105.78
CAKI-1	105.43
RXF 393	123.53
SN12C	94.17
TK-10	112.41
UO-31	79.52
**Prostate Cancer**	
PC-3	96.43
DU-145	96.97
**Breast Cancer**	
MCF7	76.22
MDA-MB-231/ATCC	102.66
HS 578T	97.48
BT-549	103.38
T-47D	89.92
MDA-MB-468	117.63
Mean	96.35
Delta	103.98
Range	131.16

Mean Growth Percent - Growth Percent

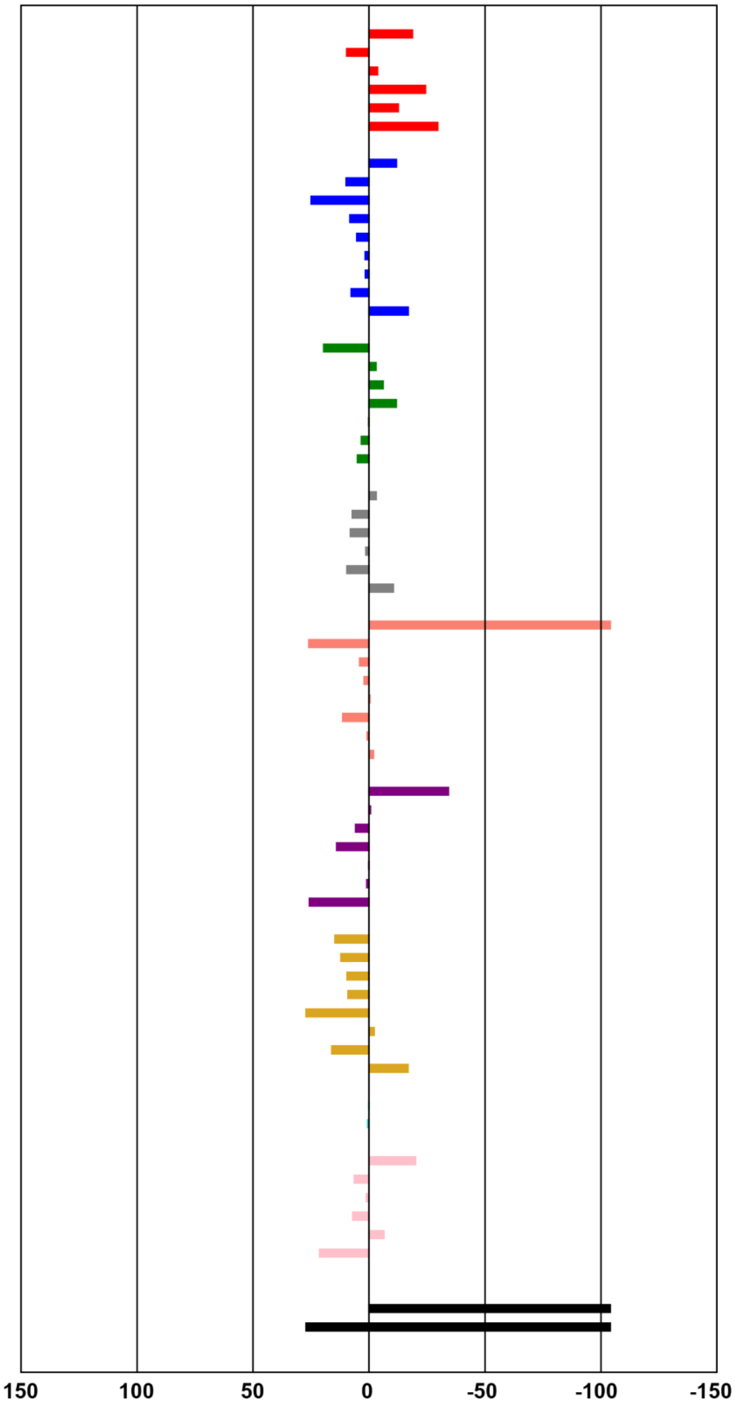

NCI screening results of compound **13i**

Developmental Therapeutics Program One Dose Mean Graph	NSC: D-846932 / 1	Conc: 1.00E-5 Molar	Test Date: Jul 24, 2023
	Experiment ID: 2307OS49		Report Date: Sep 03, 2023

Panel/Cell Line	Growth Percent
Leukemia	
CCRF-CEM	81.98
HL-60(TB)	103.18
K-562	89.16
MOLT-4	65.75
RPMI-8226	42.03
SR	68.73
Non-Small Cell Lung Cancer	
A549/ATCC	75.09
EKVX	97.36
HOP-62	137.54
HOP-92	112.93
NCI-H226	94.19
NCI-H23	100.16
NCI-H322M	111.84
NCI-H460	114.61
NCI-H522	74.07
Colon Cancer	
COLO 205	123.70
HCC-2998	121.78
HCT-116	28.52
HCT-15	107.77
HT29	88.78
KM12	103.48
SW-620	130.89
CNS Cancer	
SF-268	101.22
SF-295	96.49
SF-539	98.46
SNB-19	95.46
SNB-75	124.03
U251	88.82
Melanoma	
LOX IMVI	96.34
MALME-3M	124.53
M14	101.34
MDA-MB-435	90.60
SK-MEL-2	87.31
SK-MEL-28	105.75
SK-MEL-5	93.51
UACC-257	93.96
Ovarian Cancer	
IGROV1	141.31
OVCAR-3	101.73
OVCAR-4	91.19
OVCAR-5	114.19
OVCAR-8	98.47
NCI/ADR-RES	100.05
SK-OV-3	152.81
Renal Cancer	
786-0	112.82
A498	108.08
ACHN	111.44
CAKI-1	126.54
RXF 393	139.41
SN12C	87.20
TK-10	109.46
UO-31	79.53
Prostate Cancer	
PC-3	87.70
DU-145	108.70
Breast Cancer	
MCF7	63.59
MDA-MB-231/ATCC	102.77
HS 578T	111.13
BT-549	104.08
T-47D	97.37
MDA-MB-468	124.59
Mean	100.77
Delta	72.25
Range	124.29

Mean Growth Percent - Growth Percent

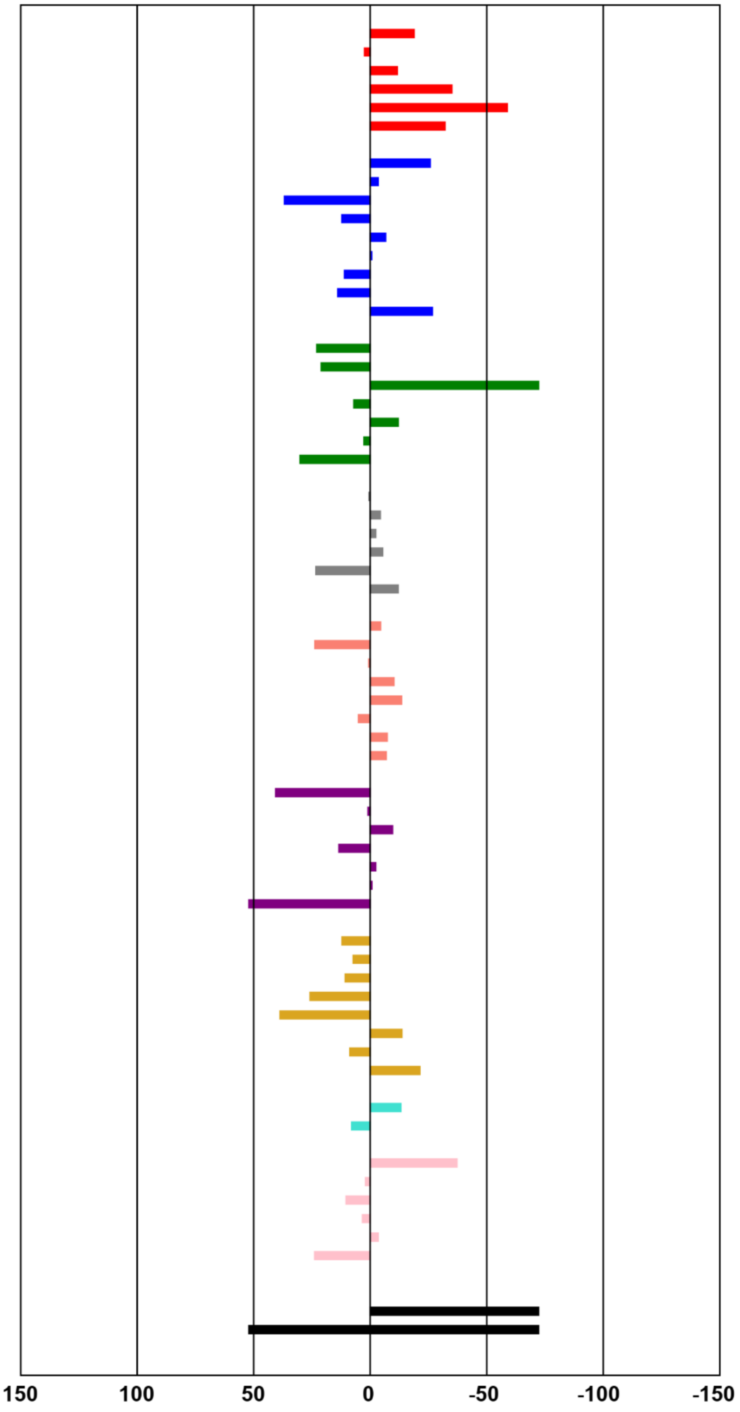

NCI screening results of compound **14a**

Developmental Therapeutics Program<br>One Dose Mean Graph	NSC: D-845229 / 1	Conc: 1.00E-5 Molar	Test Date: Jun 05, 2023
	Experiment ID: 2306OS32		Report Date: Sep 03, 2023

Panel/Cell Line	Growth Percent
**Leukemia**	
CCRF-CEM	97.34
HL-60(TB)	115.32
K-562	102.70
MOLT-4	95.51
RPMI-8226	90.93
SR	82.61
**Non-Small Cell Lung Cancer**	
A549/ATCC	103.58
EKVX	94.87
HOP-62	106.81
HOP-92	130.56
NCI-H226	114.34
NCI-H23	99.71
NCI-H322M	120.29
NCI-H460	108.61
NCI-H522	90.90
**Colon Cancer**	
COLO 205	122.13
HCC-2998	112.33
HCT-116	97.83
HCT-15	97.77
HT29	114.95
KM12	99.61
SW-620	131.77
**CNS Cancer**	
SF-268	113.83
SF-295	96.57
SF-539	100.00
SNB-19	93.78
SNB-75	99.30
U251	108.05
**Melanoma**	
LOX IMVI	95.85
MALME-3M	117.82
M14	95.87
MDA-MB-435	100.35
SK-MEL-2	102.60
SK-MEL-28	108.41
SK-MEL-5	99.86
UACC-257	114.71
UACC-62	75.26
**Ovarian Cancer**	
IGROV1	137.56
OVCAR-3	127.06
OVCAR-4	117.22
OVCAR-5	116.12
OVCAR-8	108.44
NCI/ADR-RES	101.69
SK-OV-3	98.87
**Renal Cancer**	
786-0	96.75
A498	113.53
ACHN	101.74
CAKI-1	91.57
RXF 393	116.85
SN12C	103.24
TK-10	111.01
UO-31	68.63
**Prostate Cancer**	
PC-3	94.35
DU-145	118.25
**Breast Cancer**	
MCF7	75.52
MDA-MB-231/ATCC	96.75
HS 578T	103.37
BT-549	99.57
T-47D	81.85
MDA-MB-468	115.83
Mean	104.14
Delta	35.51
Range	68.93

Mean Growth Percent - Growth Percent

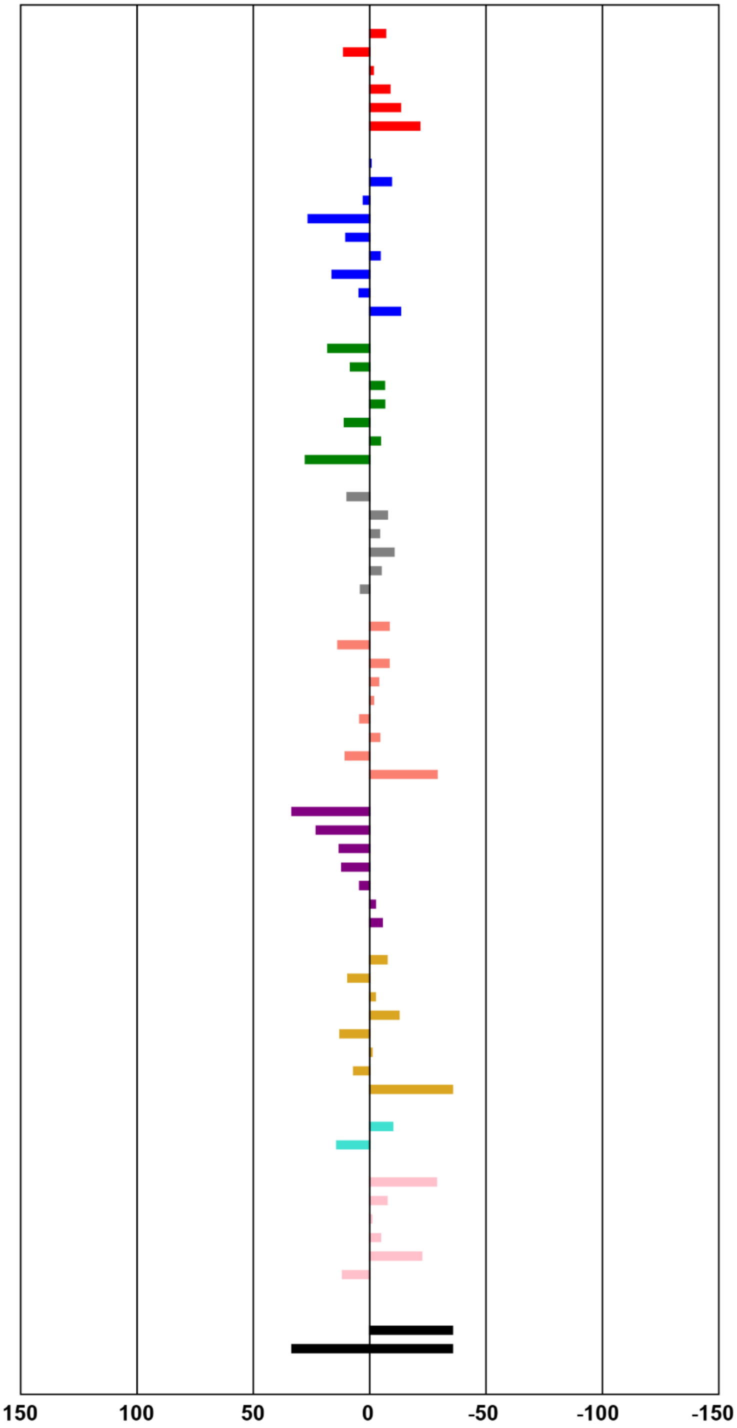

NCI screening results of compound **14b**

Developmental Therapeutics Program One Dose Mean Graph	NSC: D-846931 / 1	Conc: 1.00E-5 Molar	Test Date: Jul 24, 2023
	Experiment ID: 2307OS49		Report Date: Sep 03, 2023

Panel/Cell Line	Growth Percent
Leukemia	
CCRF-CEM	89.64
HL-60(TB)	98.51
K-562	102.05
MOLT-4	75.49
RPMI-8226	98.78
SR	61.78
Non-Small Cell Lung Cancer	
A549/ATCC	79.39
EKVX	99.75
HOP-62	139.00
HOP-92	105.75
NCI-H226	103.26
NCI-H23	98.17
NCI-H322M	127.09
NCI-H460	116.30
NCI-H522	65.79
Colon Cancer	
COLO 205	124.30
HCC-2998	120.06
HCT-116	95.08
HCT-15	97.48
HT29	97.92
KM12	107.97
SW-620	130.21
CNS Cancer	
SF-268	130.93
SF-295	102.89
SF-539	97.37
SNB-19	93.38
SNB-75	107.63
U251	96.22
Melanoma	
LOX IMVI	95.91
MALME-3M	138.25
M14	112.76
MDA-MB-435	102.09
SK-MEL-2	80.91
SK-MEL-28	108.22
SK-MEL-5	94.18
UACC-257	93.88
Ovarian Cancer	
IGROV1	141.53
OVCAR-3	106.03
OVCAR-4	110.59
OVCAR-5	112.79
OVCAR-8	100.63
NCI/ADR-RES	103.85
SK-OV-3	137.30
Renal Cancer	
786-0	112.97
A498	111.39
ACHN	103.28
CAKI-1	104.01
RXF 393	133.84
SN12C	94.04
TK-10	109.09
UO-31	85.71
Prostate Cancer	
PC-3	91.99
DU-145	120.58
Breast Cancer	
MCF7	88.47
MDA-MB-231/ATCC	100.89
HS 578T	103.81
BT-549	102.11
T-47D	92.75
MDA-MB-468	139.44
Mean	105.01
Delta	43.23
Range	79.75

Mean Growth Percent - Growth Percent

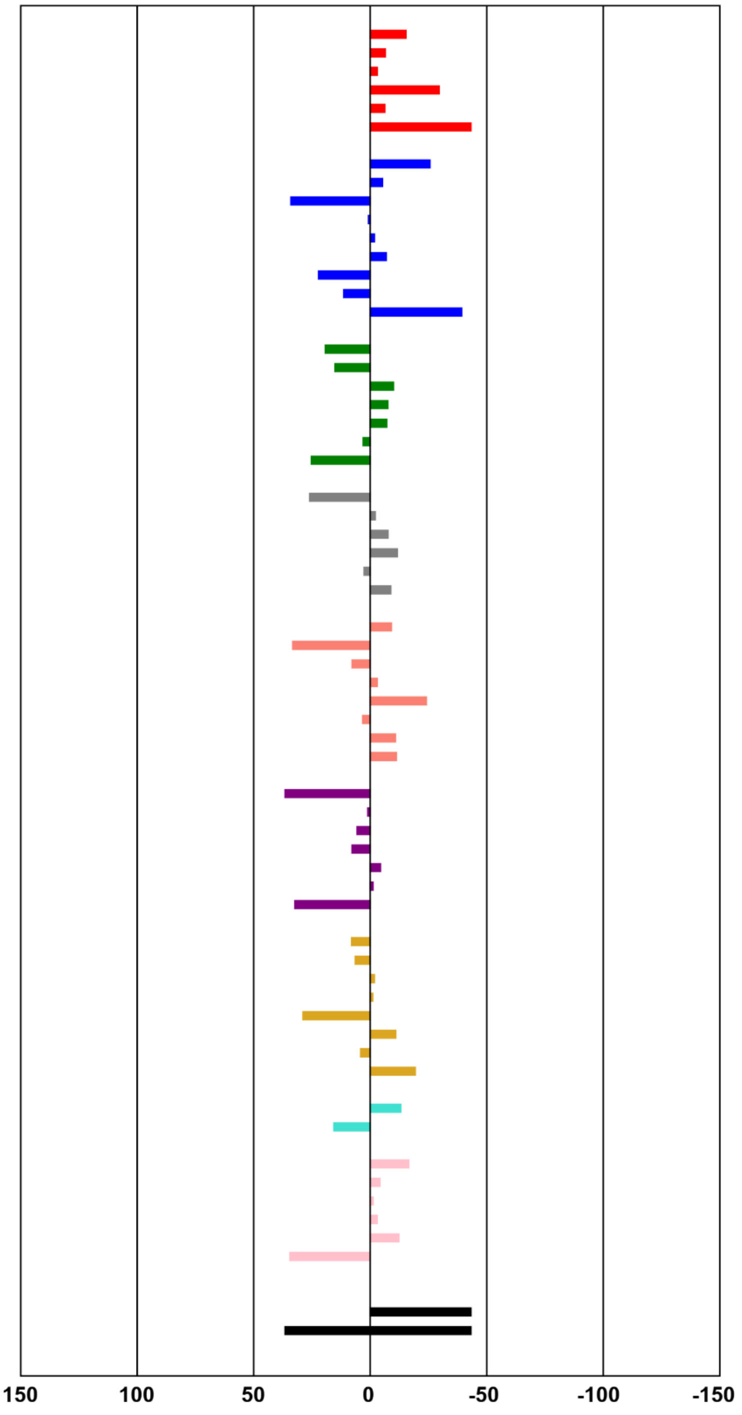

NCI screening results of compound **14c**

Developmental Therapeutics Program

One Dose Mean Graph

**NSC:** D-849632 / 1	**Conc:** 1.00E-5 Molar
**Test Date:** Oct 23, 2023	**Experiment ID:** 2310OS97
**Report Date:** Dec 29, 2023	

Panel/Cell Line	Growth Percent
Leukemia	
CCRF-CEM	70.97
HL-60(TB)	75.14
K-562	82.47
MOLT-4	55.15
RPMI-8226	47.02
Non-Small Cell Lung Cancer	
A549/ATCC	100.03
EKVX	83.56
HOP-62	106.30
HOP-92	104.04
NCI-H226	91.13
NCI-H23	98.71
NCI-H322M	117.09
NCI-H460	95.36
NCI-H522	86.05
Colon Cancer	
COLO 205	105.49
HCC-2998	126.68
HCT-116	68.22
HCT-15	71.49
HT29	106.62
KM12	98.04
SW-620	104.56
CNS Cancer	
SF-268	104.32
SF-295	87.89
SF-539	93.08
SNB-19	92.50
SNB-75	88.92
U251	105.12
Melanoma	
LOX IMVI	88.32
MALME-3M	122.46
M14	101.96
MDA-MB-435	92.03
SK-MEL-2	102.72
SK-MEL-28	105.75
SK-MEL-5	86.17
UACC-257	91.94
UACC-62	74.19
Ovarian Cancer	
IGROV1	121.54
OVCAR-3	114.99
OVCAR-4	98.07
OVCAR-5	131.41
OVCAR-8	80.53
NCI/ADR-RES	98.06
SK-OV-3	107.35
Renal Cancer	
786-0	102.75
A498	106.02
ACHN	97.58
CAKI-1	106.20
RXF 393	72.00
SN12C	92.49
TK-10	130.79
UO-31	82.61
Prostate Cancer	
PC-3	86.21
DU-145	108.31
Breast Cancer	
MCF7	52.25
MDA-MB-231/ATCC	94.01
HS 578T	98.86
BT-549	97.28
T-47D	68.73
MDA-MB-468	89.31
Mean	94.39
Delta	47.37
Range	84.39

Mean Growth Percent - Growth Percent

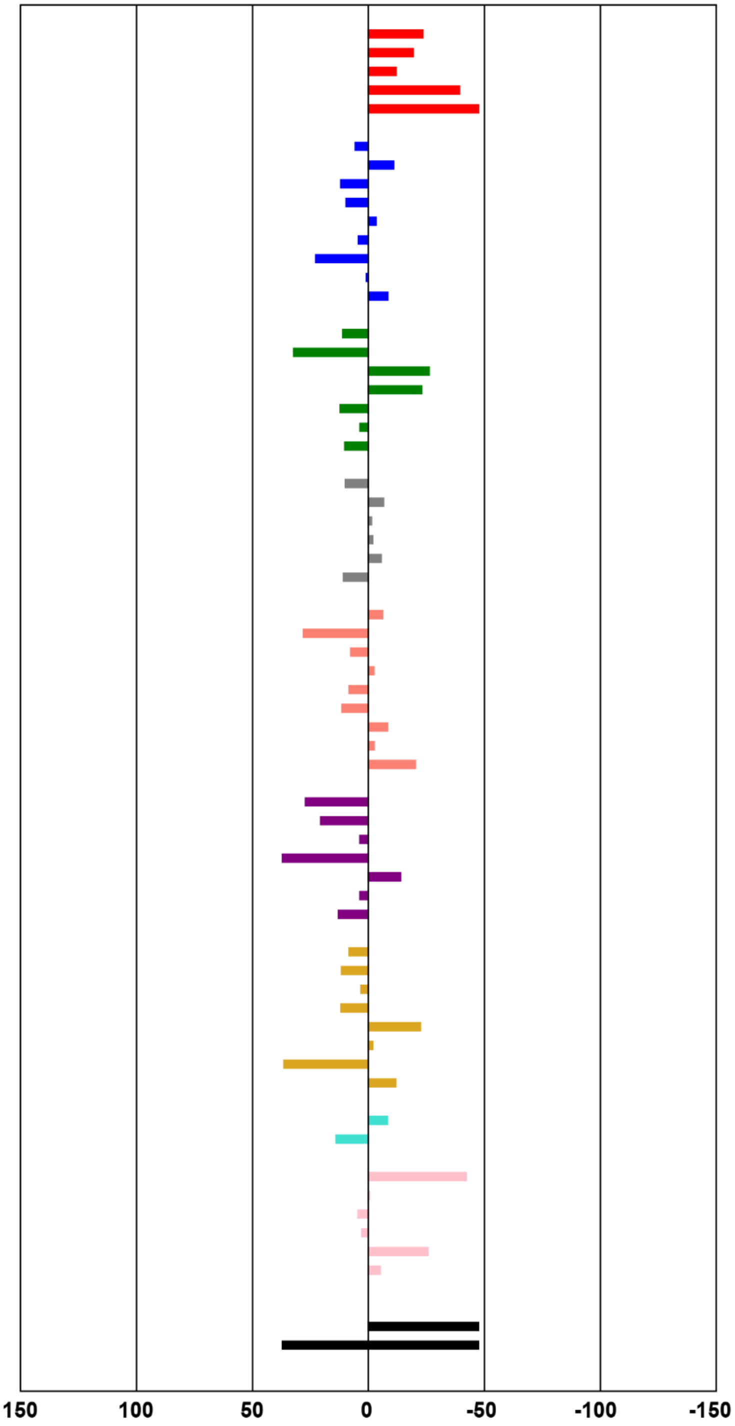

NCI screening results of compound **14d**

Developmental Therapeutics Program One Dose Mean Graph	NSC: D-845228 / 1	Conc: 1.00E-5 Molar	Test Date: Jun 05, 2023
	Experiment ID: 2306OS32		Report Date: Sep 03, 2023

Panel/Cell Line	Growth Percent
**Leukemia**	
CCRF-CEM	77.55
HL-60(TB)	90.93
K-562	78.96
MOLT-4	70.66
RPMI-8226	18.63
SR	67.30
**Non-Small Cell Lung Cancer**	
A549/ATCC	93.93
EKVX	83.26
HOP-62	104.99
HOP-92	93.16
NCI-H226	107.12
NCI-H23	97.94
NCI-H322M	120.12
NCI-H460	105.67
NCI-H522	82.32
**Colon Cancer**	
COLO 205	121.75
HCC-2998	99.12
HCT-116	63.17
HCT-15	84.71
HT29	110.71
KM12	93.13
SW-620	119.81
**CNS Cancer**	
SF-268	103.93
SF-295	95.85
SF-539	86.96
SNB-19	86.91
SNB-75	102.72
U251	85.76
**Melanoma**	
LOX IMVI	82.60
MALME-3M	111.12
M14	91.84
MDA-MB-435	89.75
SK-MEL-2	100.38
SK-MEL-28	103.08
SK-MEL-5	85.23
UACC-257	113.45
UACC-62	67.65
**Ovarian Cancer**	
IGROV1	135.61
OVCAR-3	95.27
OVCAR-4	89.47
OVCAR-5	117.63
OVCAR-8	102.14
NCI/ADR-RES	90.85
SK-OV-3	102.23
**Renal Cancer**	
786-0	88.30
A498	102.18
ACHN	98.66
CAKI-1	87.80
RXF 393	102.50
SN12C	93.13
TK-10	97.68
UO-31	69.81
**Prostate Cancer**	
PC-3	81.92
DU-145	94.97
**Breast Cancer**	
MCF7	50.01
MDA-MB-231/ATCC	77.70
HS 578T	104.32
BT-549	88.15
T-47D	67.51
MDA-MB-468	99.40
Mean	92.16
Delta	73.53
Range	116.98

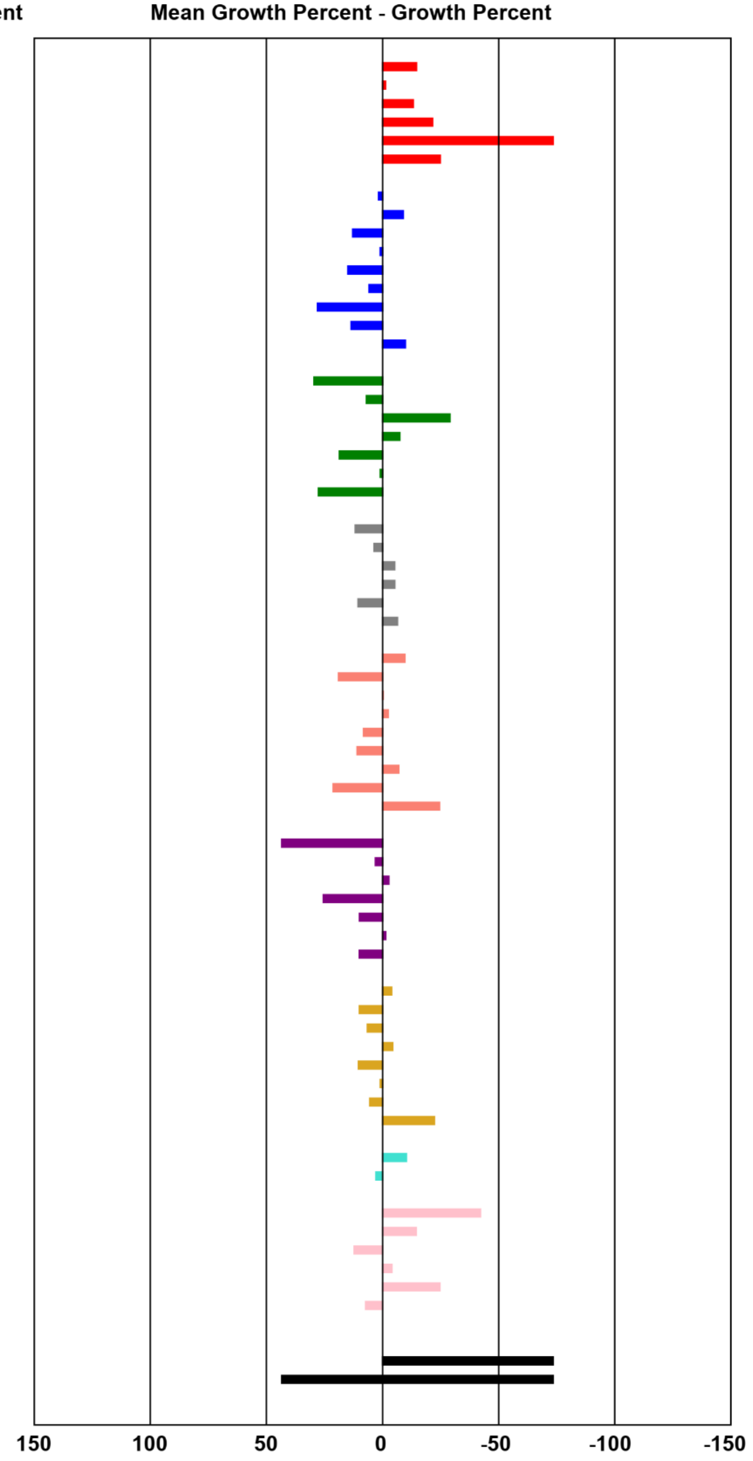

NCI screening results of compound **14e**

Developmental Therapeutics Program<br>One Dose Mean Graph	NSC: D-849629 / 1	Conc: 1.00E-5 Molar	Test Date: Oct 23, 2023
	Experiment ID: 2310OS97		Report Date: Dec 29, 2023

Panel/Cell Line	Growth Percent
**Leukemia**	
CCRF-CEM	94.64
HL-60(TB)	98.63
K-562	90.13
MOLT-4	86.39
RPMI-8226	24.91
**Non-Small Cell Lung Cancer**	
A549/ATCC	96.12
EKVX	92.72
HOP-62	95.73
HOP-92	123.24
NCI-H226	91.52
NCI-H23	100.59
NCI-H322M	101.72
NCI-H460	105.18
NCI-H522	87.59
**Colon Cancer**	
COLO 205	112.96
HCC-2998	93.01
HCT-116	80.47
HCT-15	90.74
HT29	110.34
KM12	102.39
SW-620	104.13
**CNS Cancer**	
SF-268	103.14
SF-295	81.31
SF-539	97.54
SNB-19	88.09
SNB-75	79.44
U251	94.82
**Melanoma**	
LOX IMVI	92.28
MALME-3M	102.29
M14	96.13
MDA-MB-435	96.62
SK-MEL-2	96.21
SK-MEL-28	106.23
SK-MEL-5	101.51
UACC-257	101.64
UACC-62	72.20
**Ovarian Cancer**	
IGROV1	104.97
OVCAR-3	116.32
OVCAR-4	94.53
OVCAR-5	125.10
OVCAR-8	104.21
NCI/ADR-RES	97.55
SK-OV-3	98.23
**Renal Cancer**	
786-0	106.23
A498	92.01
ACHN	103.53
CAKI-1	100.24
RXF 393	103.73
SN12C	94.18
TK-10	105.65
UO-31	64.84
**Prostate Cancer**	
PC-3	83.40
DU-145	106.99
**Breast Cancer**	
MCF7	62.52
MDA-MB-231/ATCC	96.66
HS 578T	96.97
BT-549	119.01
T-47D	80.95
MDA-MB-468	92.31
Mean	95.64
Delta	70.73
Range	100.19

Mean Growth Percent - Growth Percent

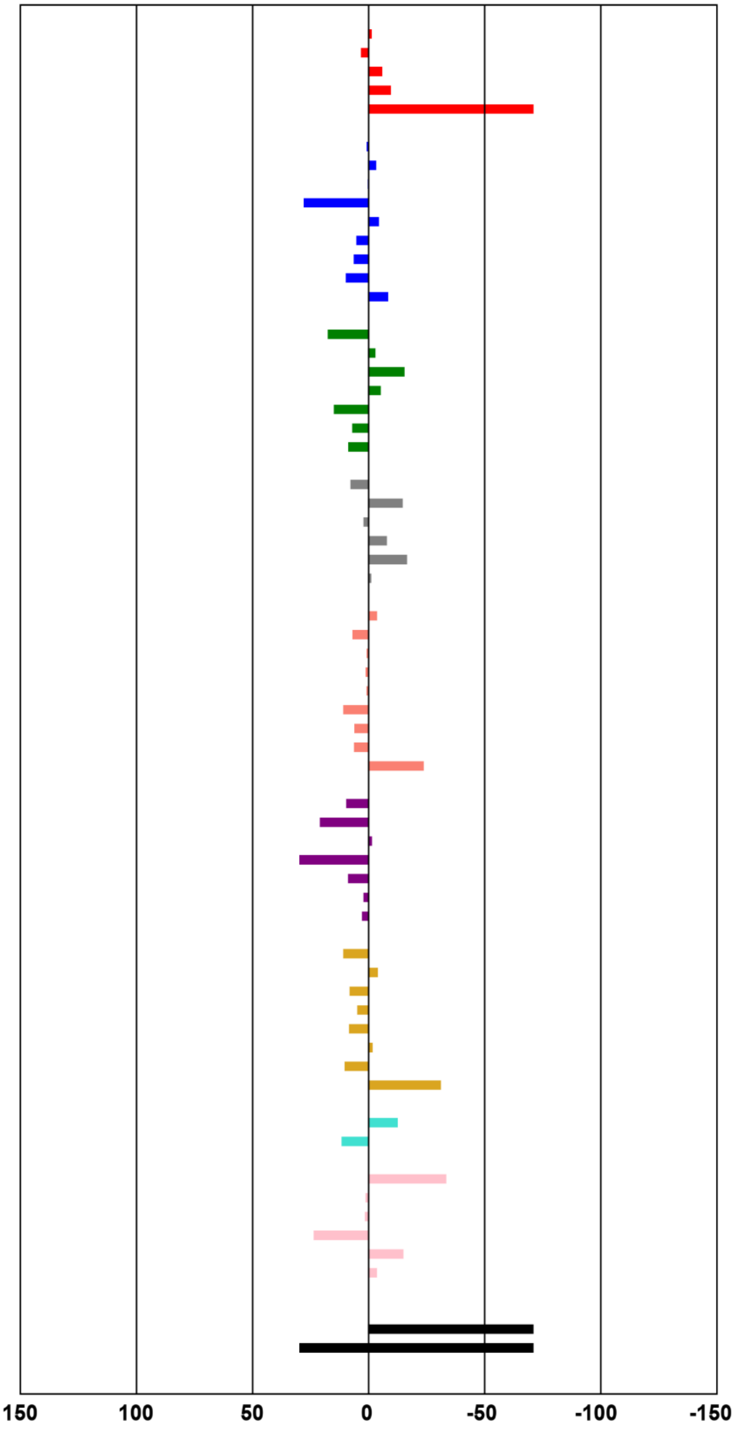

NCI screening results of compound **14f**

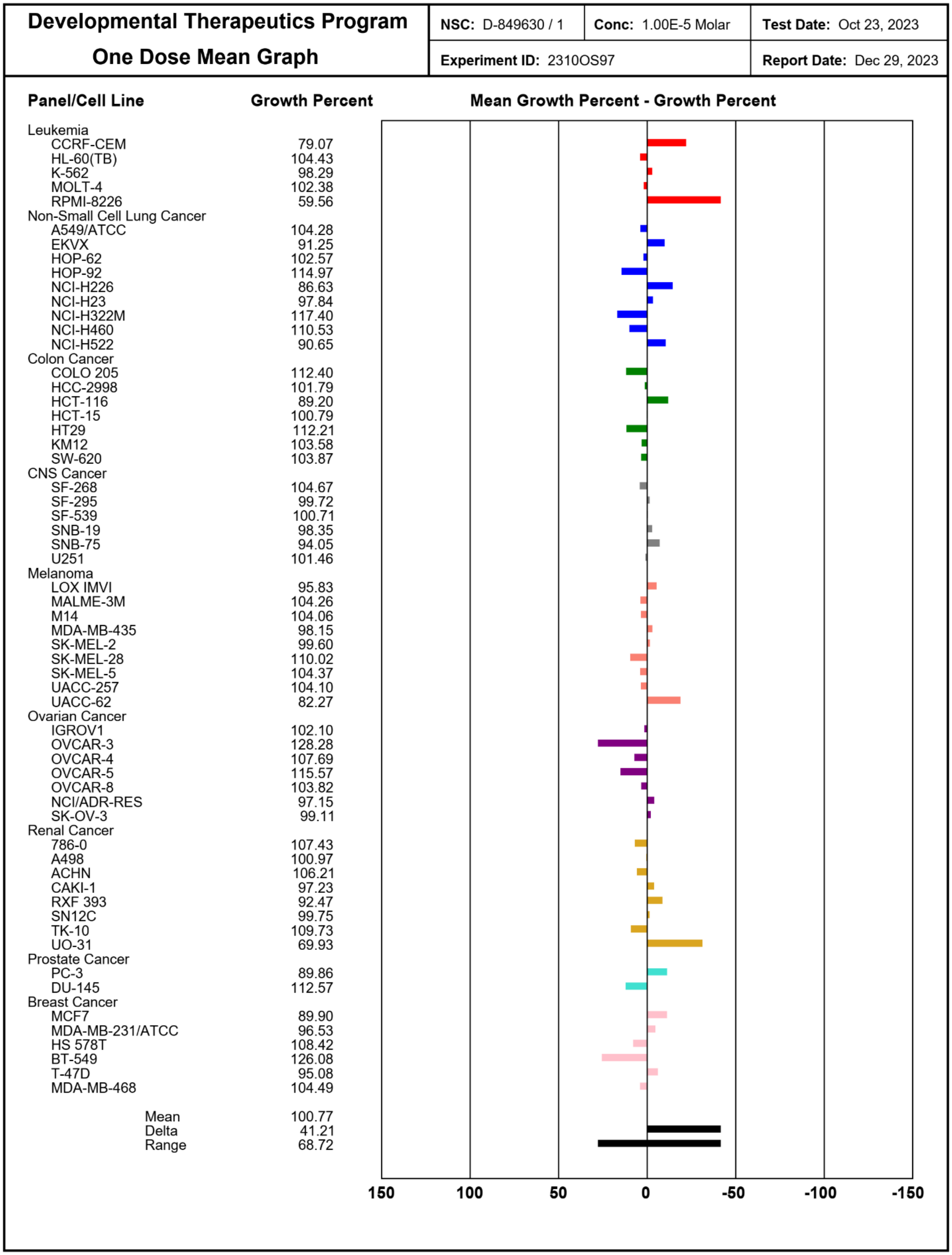

NCI screening results of compound **14g**

Developmental Therapeutics Program	NSC: D-845230 / 1	Conc: 1.00E-5 Molar	Test Date: Jun 05, 2023
One Dose Mean Graph	Experiment ID: 2306OS32		Report Date: Sep 03, 2023

Panel/Cell Line	Growth Percent
**Leukemia**	
CCRF-CEM	88.42
HL-60(TB)	99.78
K-562	94.48
MOLT-4	80.99
RPMI-8226	63.02
SR	75.79
**Non-Small Cell Lung Cancer**	
A549/ATCC	96.38
EKVX	97.03
HOP-62	119.95
HOP-92	143.49
NCI-H226	114.78
NCI-H23	101.42
NCI-H322M	123.08
NCI-H460	107.99
NCI-H522	96.38
**Colon Cancer**	
COLO 205	123.18
HCC-2998	110.80
HCT-116	78.54
HCT-15	87.93
HT29	107.59
KM12	93.24
SW-620	119.88
**CNS Cancer**	
SF-268	110.35
SF-295	103.79
SF-539	101.72
SNB-19	98.74
SNB-75	102.31
U251	103.85
**Melanoma**	
LOX IMVI	94.73
MALME-3M	105.82
M14	94.30
MDA-MB-435	88.26
SK-MEL-2	98.67
SK-MEL-28	110.45
SK-MEL-5	97.46
UACC-257	118.37
UACC-62	76.11
**Ovarian Cancer**	
IGROV1	134.55
OVCAR-3	125.73
OVCAR-4	101.65
OVCAR-5	126.80
OVCAR-8	103.63
NCI/ADR-RES	98.61
SK-OV-3	110.03
**Renal Cancer**	
786-0	100.49
A498	121.79
ACHN	101.39
CAKI-1	97.13
RXF 393	104.70
SN12C	107.35
TK-10	110.05
UO-31	69.14
**Prostate Cancer**	
PC-3	91.02
DU-145	106.01
**Breast Cancer**	
MCF7	63.19
MDA-MB-231/ATCC	99.75
HS 578T	110.27
BT-549	99.91
T-47D	91.42
MDA-MB-468	114.39
Mean	101.97
Delta	38.95
Range	80.47

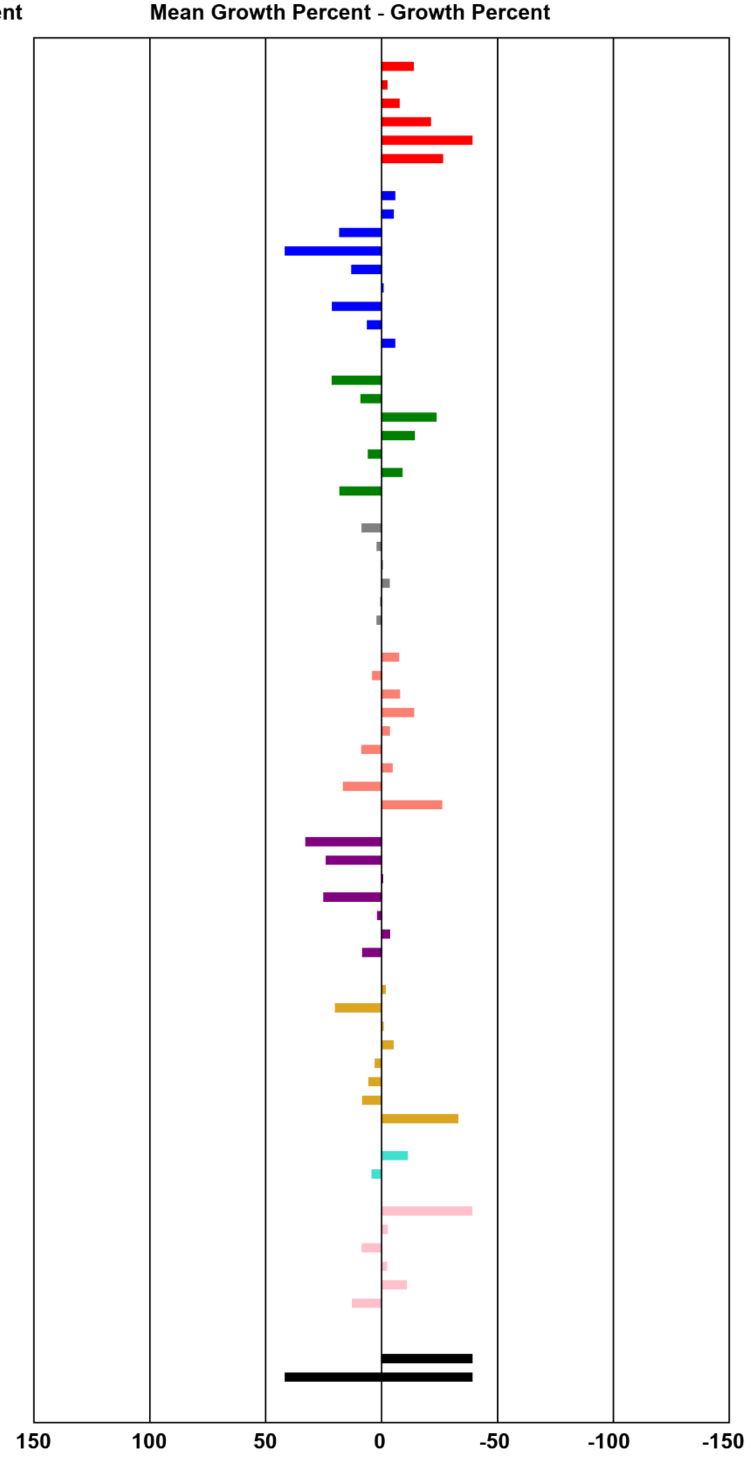

NCI screening results of compound **14h**

Developmental Therapeutics Program<br>One Dose Mean Graph	NSC: D-846933 / 1	Conc: 1.00E-5 Molar	Test Date: Jul 24, 2023
	Experiment ID: 2307OS49		Report Date: Sep 03, 2023

Panel/Cell Line	Growth Percent
Leukemia	
CCRF-CEM	93.96
HL-60(TB)	109.19
K-562	102.07
MOLT-4	81.50
RPMI-8226	91.71
SR	76.57
Non-Small Cell Lung Cancer	
A549/ATCC	90.47
EKVX	105.01
HOP-62	128.75
HOP-92	127.31
NCI-H226	104.44
NCI-H23	97.88
NCI-H322M	134.67
NCI-H460	109.28
NCI-H522	84.25
Colon Cancer	
COLO 205	118.23
HCC-2998	121.52
HCT-116	83.32
HCT-15	109.05
HT29	99.51
KM12	99.63
SW-620	118.18
CNS Cancer	
SF-268	104.44
SF-295	117.87
SF-539	107.15
SNB-19	99.36
SNB-75	98.45
U251	93.66
Melanoma	
LOX IMVI	93.90
MALME-3M	135.28
M14	100.23
MDA-MB-435	103.09
SK-MEL-2	85.35
SK-MEL-28	106.97
SK-MEL-5	102.55
UACC-257	96.48
Ovarian Cancer	
IGROV1	129.82
OVCAR-3	104.93
OVCAR-4	103.01
OVCAR-5	114.95
OVCAR-8	104.14
NCI/ADR-RES	106.74
SK-OV-3	126.79
Renal Cancer	
786-0	119.23
A498	127.22
ACHN	105.91
CAKI-1	102.16
RXF 393	151.48
SN12C	97.61
TK-10	123.35
UO-31	87.85
Prostate Cancer	
PC-3	89.07
DU-145	105.55
Breast Cancer	
MCF7	82.02
MDA-MB-231/ATCC	101.93
HS 578T	110.84
BT-549	120.60
T-47D	97.77
MDA-MB-468	126.90
Mean	106.29
Delta	29.72
Range	74.91

Mean Growth Percent - Growth Percent

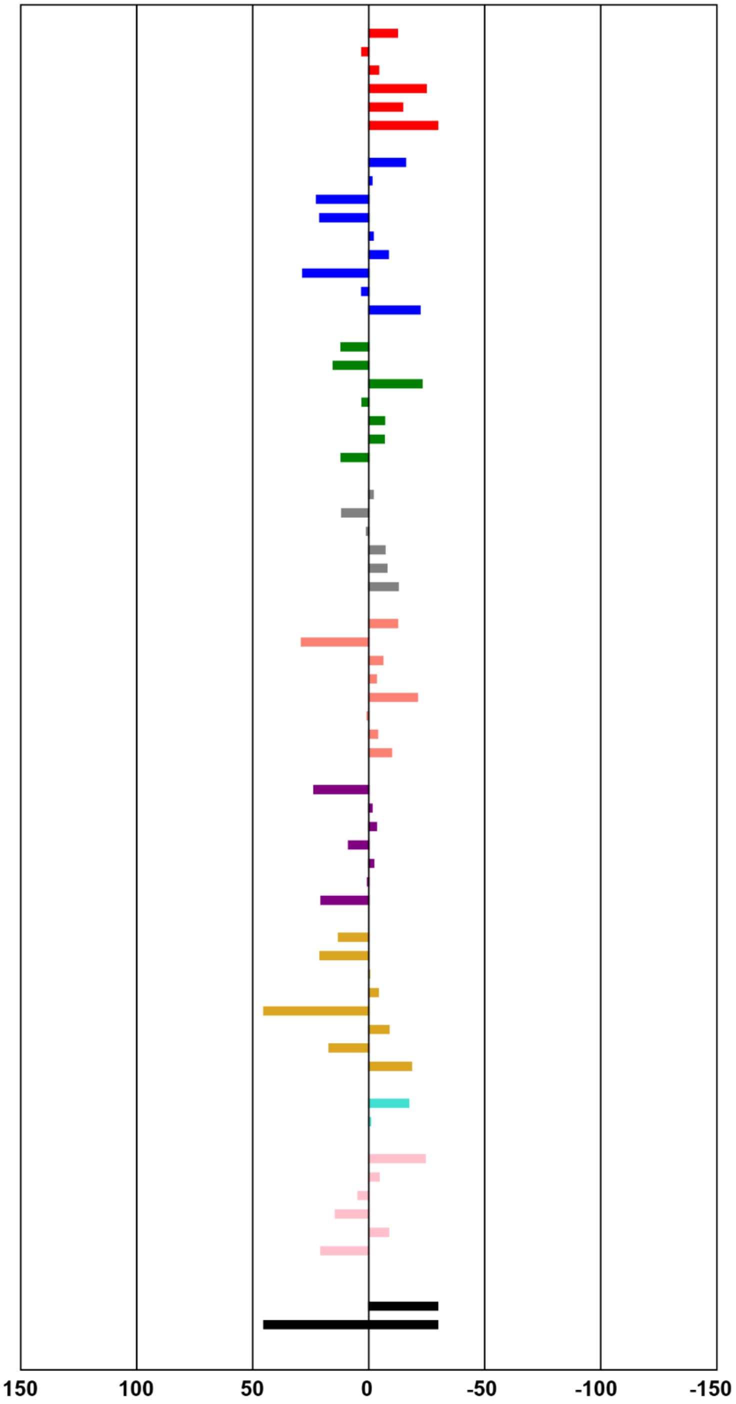

NCI screening results of compound **14i**

National Cancer Institute Developmental Therapeutics Program
In-Vitro Testing Results

NSC : D - 852384 / 1	Experiment ID : 2407HT58	Test Type : HTS	Units : Molar
Report Date : September 23, 2024	Test Date : July 22, 2024	QNS :	MC :
COMI : FA-C	Stain Reagent :	SSPL : 0XUD	

			Log10 Concentration												
	Time	Mean Optical Densities						Percent Growth							
Panel/Cell Line	Zero	Ctrl	-8.0	-7.0	-6.0	-5.0	-4.0	-8.0	-7.0	-6.0	-5.0	-4.0	GI50	TGI	LC50
Leukemia															
CCRF-CEM	0.971	4.465	4.274	4.261	4.194	2.865	1.279	95	94	92	54	9	* 1.24E-5	> 1.00E-4	> 1.00E-4
HL-60(TB)	0.502	2.520	2.499	2.462	2.547	1.371	0.213	99	97	101	43	-58	* 7.62E-6	* 2.68E-5	* 8.42E-5
K-562	0.421	7.034	6.321	6.180	6.162	3.376	0.614	89	87	87	45	3	* 7.47E-6	> 1.00E-4	> 1.00E-4
MOLT-4	0.728	3.936	4.141	3.963	3.838	2.417	0.316	106	101	97	53	-57	* 1.06E-5	* 3.04E-5	* 8.70E-5
RPMI-8226	4.586	10.291	8.372	8.950	8.811	4.353	0.297	66	76	74	-6	-94	* 2.00E-6	* 8.50E-6	* 3.20E-5
SR	0.953	5.966	6.050	6.054	5.610	4.120	0.571	102	102	93	63	-40	* 1.34E-5	* 4.09E-5	> 1.00E-4
Non-Small Cell Lung Cancer															
A549/ATCC	0.392	3.845	3.756	3.519	3.608	1.560	0.128	97	91	93	34	-67	* 5.34E-6	* 2.16E-5	* 6.74E-5
EKVX	5.120	12.533	10.621	10.969	10.413	6.295	1.180	74	79	71	16	-77	* 2.42E-6	* 1.48E-5	* 5.12E-5
HOP-62	1.512	4.417	4.119	4.136	4.560	1.797	0.175	90	90	105	10	-88	* 3.78E-6	* 1.26E-5	* 4.07E-5
HOP-92	8.037	10.073	9.780	9.759	9.562	5.475	0.284	86	85	75	-32	-96	* 1.71E-6	* 5.03E-6	* 1.91E-5
NCI-H226	6.623	16.636	15.794	15.155	15.079	10.338	0.788	92	85	84	37	-88	* 5.34E-6	* 1.98E-5	* 4.96E-5
NCI-H23	4.634	12.765	11.747	12.053	11.607	6.087	1.091	88	91	86	18	-76	* 3.36E-6	* 1.55E-5	* 5.24E-5
NCI-H322M	6.734	14.105	12.351	13.071	13.429	11.500	5.135	76	86	91	65	-24	* 1.46E-5	* 5.39E-5	> 1.00E-4
NCI-H460	0.969	14.239	12.196	13.035	11.518	2.292	0.085	85	91	80	10	-91	* 2.66E-6	* 1.26E-5	* 3.92E-5
NCI-H522	3.316	8.700	8.544	7.997	8.366	5.206	0.141	97	87	94	35	-96	* 5.49E-6	* 1.84E-5	* 4.46E-5
Colon Cancer															
COLO 205	0.835	4.088	3.584	3.807	4.034	2.514	0.480	84	91	98	52	-42	* 1.04E-5	* 3.54E-5	> 1.00E-4
HCC-2998	4.833	12.899	10.952	12.260	11.303	6.994	5.449	76	92	80	27	3	* 3.67E-6	> 1.00E-4	> 1.00E-4
HCT-116	0.373	4.413	4.559	4.268	4.264	1.914	0.236	104	96	96	38	-37	* 6.24E-6	* 3.23E-5	> 1.00E-4
HCT-15	1.263	11.600	9.159	11.292	10.623	5.946	3.878	76	97	91	45	25	* 7.84E-6	> 1.00E-4	> 1.00E-4
HT29	0.509	4.764	4.419	4.493	4.949	2.535	0.293	92	94	104	48	-42	* 9.07E-6	* 3.38E-5	> 1.00E-4
KM12	0.587	3.695	3.327	3.606	3.484	1.874	0.315	88	97	93	42	-46	* 6.84E-6	* 2.96E-5	> 1.00E-4
SW-620	0.580	4.432	4.140	4.233	4.148	1.934	0.426	92	95	93	35	-26	* 5.52E-6	* 3.72E-5	> 1.00E-4
CNS Cancer															
SF-268	1.084	3.256	2.999	3.193	2.890	2.108	0.087	88	97	83	47	-92	* 8.24E-6	* 2.18E-5	* 4.99E-5
SF-295	1.714	4.361	4.452	4.442	4.349	2.959	0.179	103	103	100	47	-90	* 8.78E-6	* 2.21E-5	* 5.13E-5
SF-539	4.105	11.961	11.011	11.781	11.697	7.360	0.974	88	98	97	41	-76	* 6.99E-6	* 2.25E-5	* 5.98E-5
SNB-19	1.783	4.846	4.765	4.815	4.943	3.782	0.280	97	99	103	65	-84	* 1.26E-5	* 2.73E-5	* 5.90E-5
SNB-75	2.056	3.026	2.675	2.752	2.785	1.630	0.142	64	72	75	-21	-93	* 1.82E-6	* 6.07E-6	* 2.54E-5
U251	0.623	3.644	3.489	3.624	3.527	1.072	0.006	95	99	96	15	-99	* 3.70E-6	* 1.35E-5	* 3.71E-5
Melanoma															
LOX IMVI	0.362	2.393	2.504	2.324	2.139	0.644	0.017	105	97	87	14	-95	* 3.21E-6	* 1.34E-5	* 3.85E-5
MALME-3M	2.638	4.186	3.905	3.679	4.254	1.071	0.081	83	67	104	-59	-97	* 2.15E-6	* 4.34E-6	* 8.76E-6
M14	4.190	12.223	11.230	11.615	12.349	8.624	2.692	88	92	102	55	-36	* 1.13E-5	* 4.03E-5	> 1.00E-4
MDA-MB-435	1.129	3.672	3.573	3.669	3.634	2.530	1.058	96	100	99	55	-9	* 1.20E-5	* 7.21E-5	> 1.00E-4
SK-MEL-2	2.588	5.897	6.097	5.969	6.072	1.912	0.024	106	102	105	-26	-99	* 2.63E-6	* 6.31E-6	* 2.12E-5
SK-MEL-28	1.628	5.162	4.859	4.958	5.372	3.140	0.257	91	94	106	43	-84	* 7.68E-6	* 2.17E-5	* 5.38E-5
SK-MEL-5	2.807	8.413	7.275	7.977	7.726	3.340	0.518	80	92	88	9	-82	* 3.04E-6	* 1.27E-5	* 4.50E-5
UACC-257	4.071	8.193	7.333	7.632	8.058	3.874	1.194	79	86	97	-5	-71	* 2.88E-6	* 8.96E-6	* 4.85E-5
UACC-62	0.765	3.471	3.179	3.236	3.356	1.523	0.051	89	91	96	28	-93	* 4.72E-6	* 1.70E-5	* 4.39E-5
Ovarian Cancer															
IGROV1	1.599	4.185	3.715	3.972	3.920	2.760	0.478	82	92	90	45	-70	* 7.62E-6	* 2.45E-5	* 6.68E-5
OVCAR-3	2.879	11.110	10.719	9.600	9.594	6.943	0.842	96	82	82	49	-71	* 9.25E-6	* 2.56E-5	* 6.71E-5
OVCAR-4	4.638	7.807	7.191	7.221	7.212	3.546	0.324	80	81	79	-24	-93	* 1.92E-6	* 5.90E-6	* 2.40E-5
OVCAR-5	5.432	11.681	11.036	11.255	11.136	9.625	4.405	90	93	91	67	-19	* 1.58E-5	* 6.03E-5	> 1.00E-4
OVCAR-8	0.988	4.547	4.195	4.252	4.252	1.986	0.106	90	92	92	28	-89	* 4.52E-6	* 1.73E-5	* 4.63E-5
NCI/ADR-RES	3.228	11.136	10.346	10.414	10.023	4.991	1.011	90	91	86	22	-69	* 3.67E-6	* 1.76E-5	* 6.23E-5
SK-OV-3	2.770	12.397	12.359	11.340	12.615	7.081	0.120	100	89	102	45	-96	* 8.13E-6	* 2.09E-5	* 4.73E-5
Renal Cancer															
786-0	1.731	5.201	5.342	5.263	5.223	3.428	0.093	104	102	101	49	-95	* 9.52E-6	* 2.19E-5	* 4.89E-5
A498	2.609	8.390	8.257	8.562	9.157	4.421	0.049	98	103	113	32	-98	* 5.95E-6	* 1.75E-5	* 4.25E-5
ACHN	1.003	4.558	4.459	4.606	4.610	3.236	0.230	97	101	101	63	-77	* 1.24E-5	* 2.82E-5	* 6.41E-5
CAKI-1	1.015	4.758	4.535	4.413	4.598	1.663	0.014	94	91	96	17	-99	* 3.82E-6	* 1.41E-5	* 3.80E-5
RXF 393	2.078	3.410	3.461	3.410	3.346	1.489	0.033	104	100	95	-28	-98	* 2.32E-6	* 5.89E-6	* 2.04E-5
SN12C	0.660	2.694	2.464	2.684	2.672	1.387	0.219	89	99	99	36	-67	* 5.95E-6	* 2.23E-5	* 6.85E-5
TK-10	7.459	17.390	14.582	15.332	15.784	12.241	0.252	72	79	84	48	-97	* 8.94E-6	* 2.15E-5	* 4.77E-5
UO-31	1.590	5.994	5.863	5.804	5.957	4.502	0.372	97	96	99	66	-77	* 1.30E-5	* 2.91E-5	* 6.51E-5
Prostate Cancer															
PC-3	4.255	11.740	10.682	10.831	11.044	5.557	0.227	86	88	91	17	-95	* 3.58E-6	* 1.43E-5	* 3.99E-5
DU-145	0.789	3.555	3.703	3.597	3.594	1.981	0.073	105	102	101	43	-91	* 7.62E-6	* 2.10E-5	* 4.96E-5
Breast Cancer															
MCF7	0.974	8.716	7.499	8.551	7.758	3.240	0.732	84	98	88	29	-25	* 4.43E-6	* 3.48E-5	> 1.00E-4
MDA-MB-231/ATCC	4.073	8.995	8.551	8.355	8.601	5.683	0.744	91	87	92	33	-82	* 5.11E-6	* 1.93E-5	* 5.28E-5
HS 578T	2.251	3.977	3.522	3.735	3.721	2.339	0.269	74	86	85	6	-88	* 2.76E-6	* 1.15E-5	* 3.93E-5
BT-549	6.928	13.238	11.774	12.469	11.965	7.400	1.446	77	88	80	8	-79	* 2.59E-6	* 1.23E-5	* 4.62E-5
T-47D	6.839	11.433	10.520	10.779	10.720	7.293	3.422	80	86	84	10	-50	* 2.90E-6	* 1.46E-5	> 1.00E-4
MDA-MB-468	5.295	9.689	9.214	9.201	8.907	4.992	1.452	89	89	82	-6	-73	* 2.33E-6	* 8.61E-6	* 4.60E-5

NCI five dose results of compound **10b**

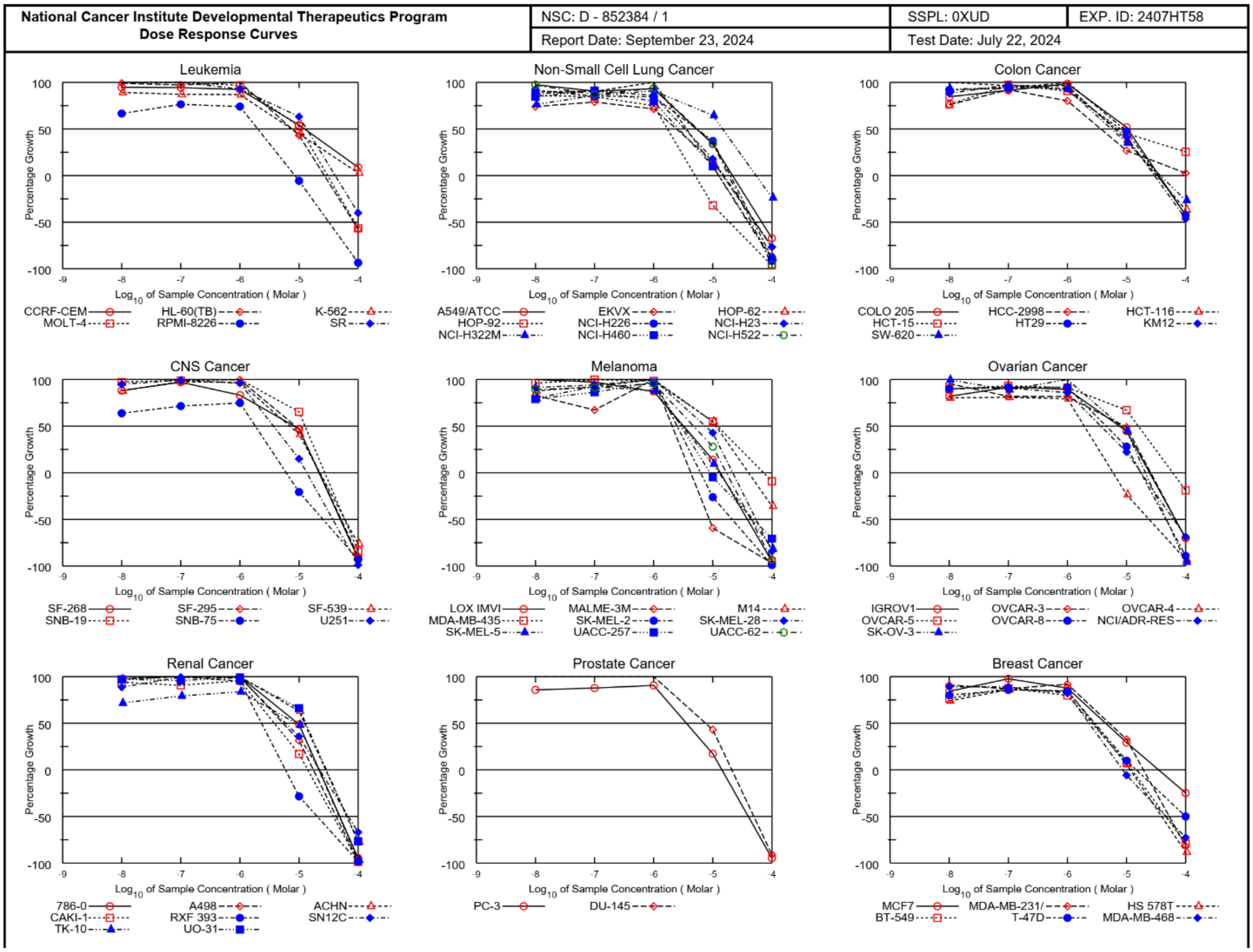

NCI five dose results of compound **10b**

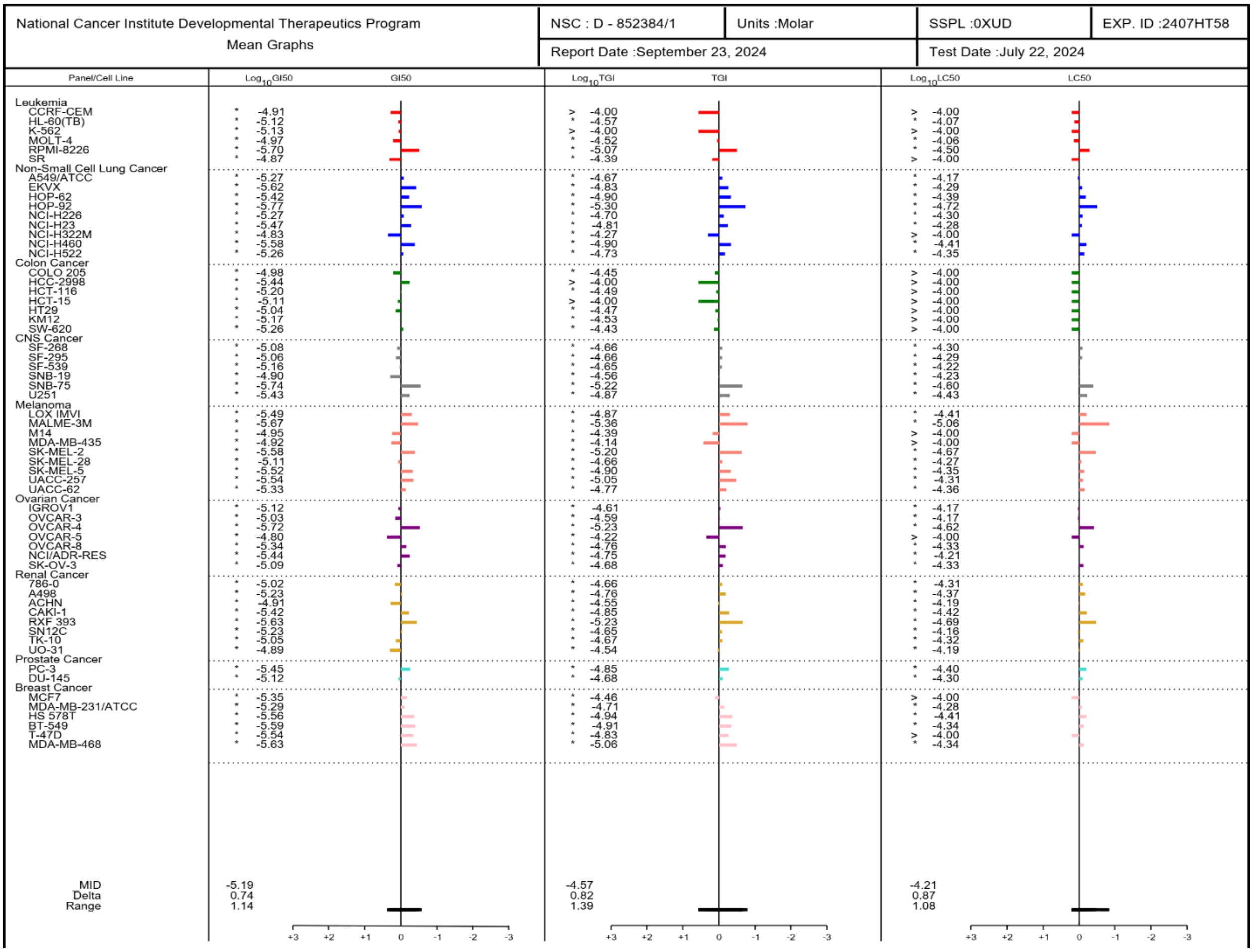

NCI five dose results of compound **10b**

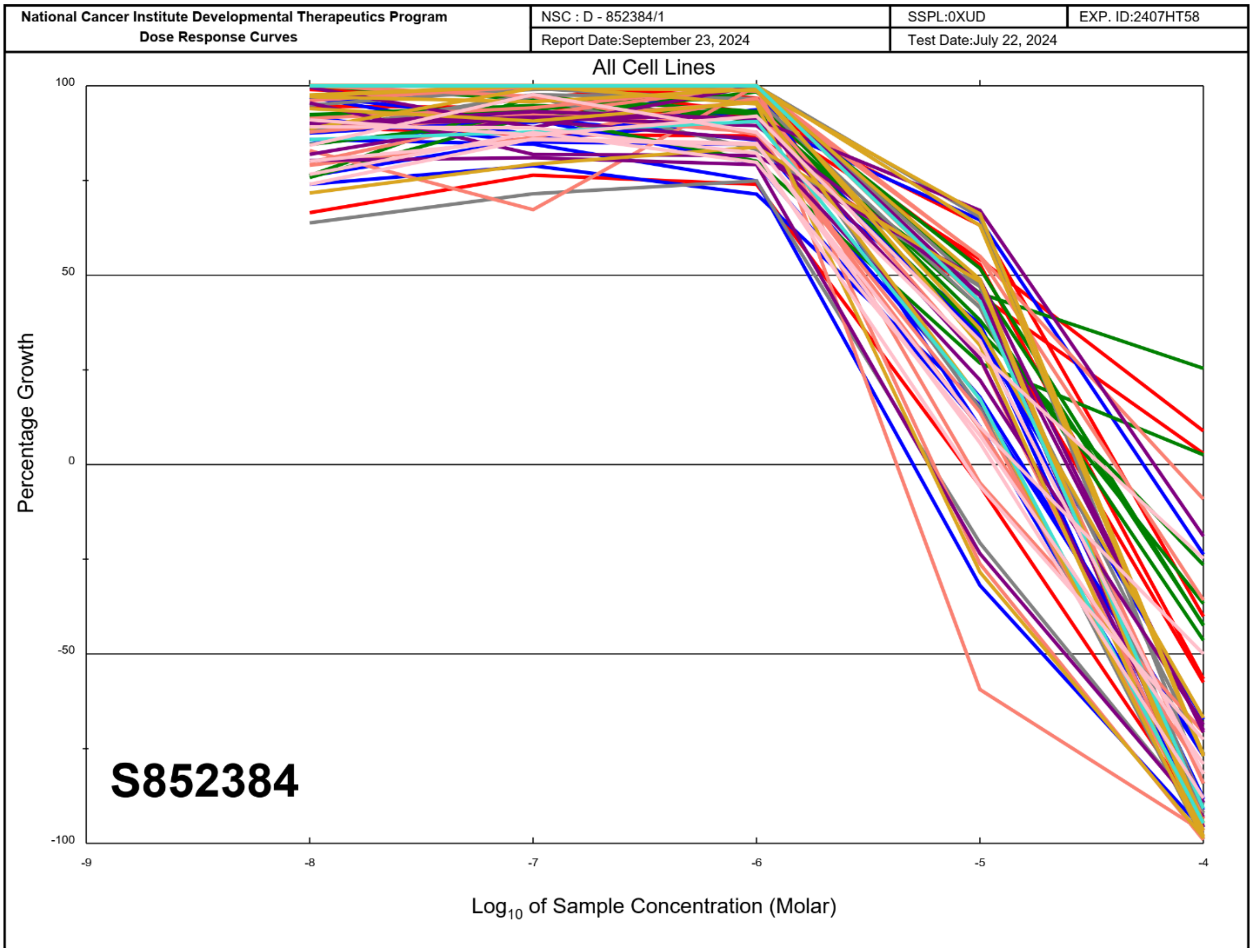

NCI five dose results of compound **10b**

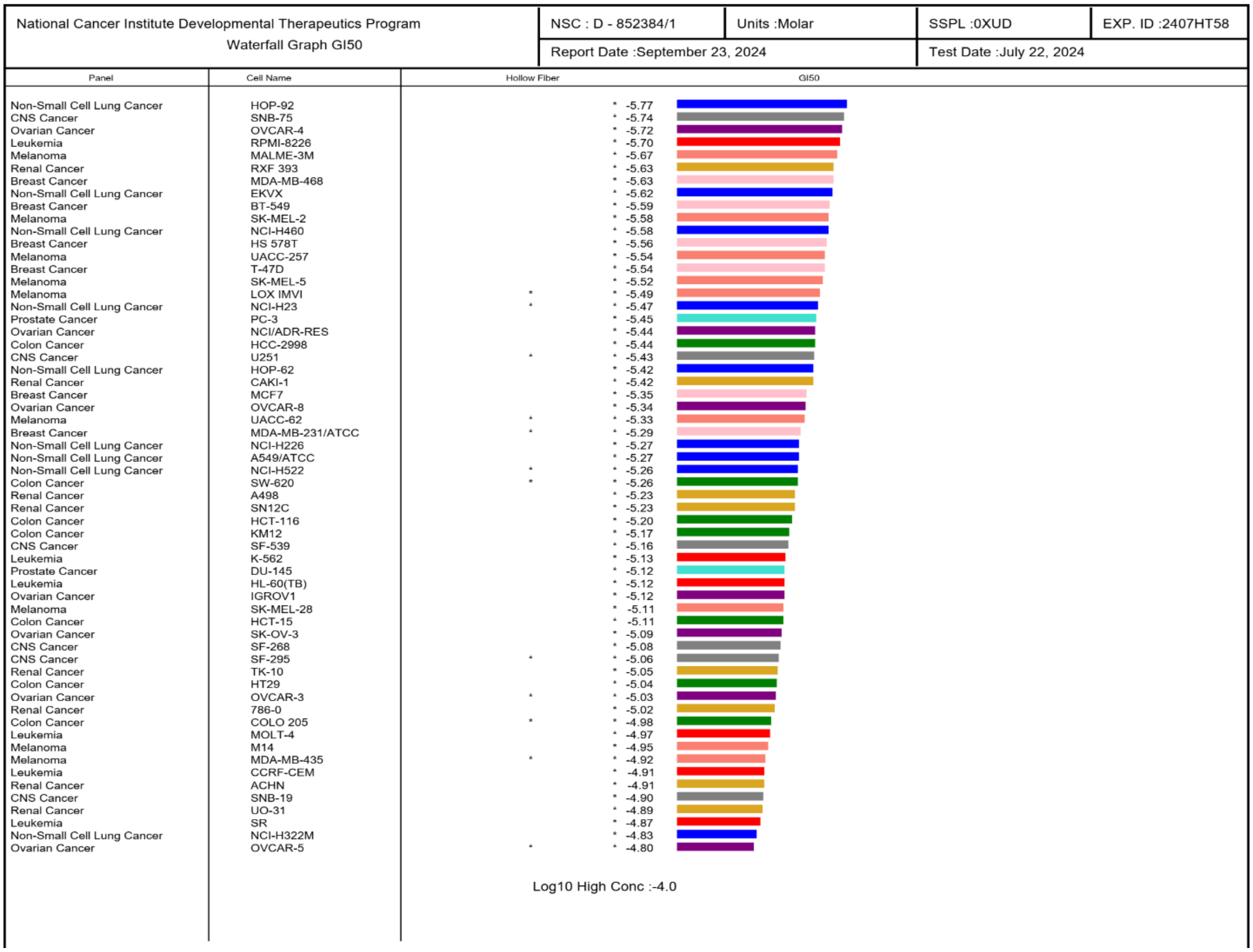

NCI five dose results of compound **10b**

National Cancer Institute Developmental Therapeutics Program<br>Waterfall Graph TGI	NSC : D - 852384/1	Units :Molar	SSPL :0XUD	EXP. ID :2407HT58
	Report Date :September 23, 2024		Test Date :July 22, 2024	

Panel	Cell Name	Hollow Fiber	TGI
Melanoma	MALME-3M		* -5.36
Non-Small Cell Lung Cancer	HOP-92		* -5.30
Renal Cancer	RXF 393		* -5.23
Ovarian Cancer	OVCAR-4		* -5.23
CNS Cancer	SNB-75		* -5.22
Melanoma	SK-MEL-2		* -5.20
Leukemia	RPMI-8226		* -5.07
Breast Cancer	MDA-MB-468		* -5.06
Melanoma	UACC-257		* -5.05
Breast Cancer	HS 578T		* -4.94
Breast Cancer	BT-549		* -4.91
Non-Small Cell Lung Cancer	NCI-H460		* -4.90
Non-Small Cell Lung Cancer	HOP-62		* -4.90
Melanoma	SK-MEL-5		* -4.90
Melanoma	LOX IMVI	*	* -4.87
CNS Cancer	U251	*	* -4.87
Renal Cancer	CAKI-1		* -4.85
Prostate Cancer	PC-3		* -4.85
Breast Cancer	T-47D		* -4.83
Non-Small Cell Lung Cancer	EKVX		* -4.83
Non-Small Cell Lung Cancer	NCI-H23	*	* -4.81
Melanoma	UACC-62	*	* -4.77
Ovarian Cancer	OVCAR-8		* -4.76
Renal Cancer	A498		* -4.76
Ovarian Cancer	NCI/ADR-RES		* -4.75
Non-Small Cell Lung Cancer	NCI-H522	*	* -4.73
Breast Cancer	MDA-MB-231/ATCC	*	* -4.71
Non-Small Cell Lung Cancer	NCI-H226		* -4.70
Ovarian Cancer	SK-OV-3		* -4.68
Prostate Cancer	DU-145		* -4.68
Renal Cancer	TK-10		* -4.67
Non-Small Cell Lung Cancer	A549/ATCC		* -4.67
Melanoma	SK-MEL-28		* -4.66
CNS Cancer	SF-268		* -4.66
Renal Cancer	786-0		* -4.66
CNS Cancer	SF-295	*	* -4.66
Renal Cancer	SN12C		* -4.65
CNS Cancer	SF-539		* -4.65
Ovarian Cancer	IGROV1		* -4.61
Ovarian Cancer	OVCAR-3	*	* -4.59
Leukemia	HL-60(TB)		* -4.57
CNS Cancer	SNB-19		* -4.56
Renal Cancer	ACHN		* -4.55
Renal Cancer	UO-31		* -4.54
Colon Cancer	KM12		* -4.53
Leukemia	MOLT-4		* -4.52
Colon Cancer	HCT-116		* -4.49
Colon Cancer	HT29		* -4.47
Breast Cancer	MCF7		* -4.46
Colon Cancer	COLO 205	*	* -4.45
Colon Cancer	SW-620	*	* -4.43
Melanoma	M14		* -4.39
Leukemia	SR		* -4.39
Non-Small Cell Lung Cancer	NCI-H322M		* -4.27
Ovarian Cancer	OVCAR-5	*	* -4.22
Melanoma	MDA-MB-435	*	* -4.14
Leukemia	CCRF-CEM		> -4.00
Leukemia	K-562		> -4.00
Colon Cancer	HCC-2998		> -4.00
Colon Cancer	HCT-15		> -4.00

Log10 High Conc : -4.0

NCI five dose results of compound **10b**

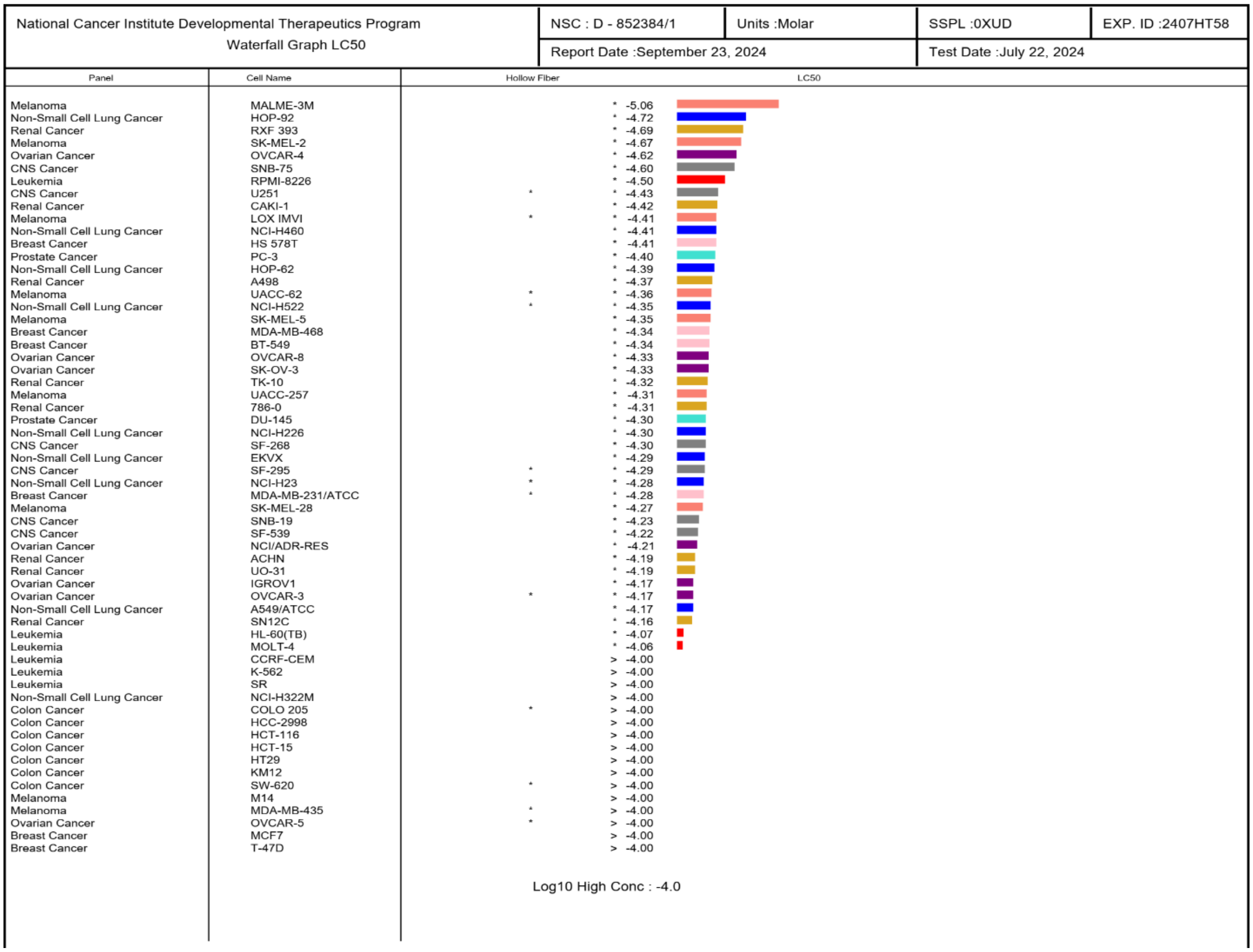

NCI five dose results of compound **10b**

National Cancer Institute Developmental Therapeutics Program
In-Vitro Testing Results

NSC : D - 852387 / 1	Experiment ID : 2407HT59	Test Type : HTS	Units : Molar
Report Date : September 23, 2024	Test Date : July 29, 2024	QNS :	MC :
COMI : HA-H	Stain Reagent :	SSPL : 0XUD	

			Log10 Concentration												
	Time		Mean Optical Densities					Percent Growth							
Panel/Cell Line	Zero	Ctrl	-8.0	-7.0	-6.0	-5.0	-4.0	-8.0	-7.0	-6.0	-5.0	-4.0	GI50	TGI	LC50
**Leukemia**															
CCRF-CEM	0.676	4.761	4.980	4.965	4.383	0.097	0.016	105	105	91	-86	-98	* 1.70E-6	* 3.27E-6	* 6.28E-6
HL-60(TB)	0.587	3.503	4.042	3.969	3.248	0.033	0.009	118	116	91	-94	-98	* 1.67E-6	* 3.10E-6	* 5.76E-6
K-562	0.506	7.319	7.000	7.688	4.938	0.565	0.025	96	105	65	-1	-95	* 1.69E-6	* 9.83E-6	* 3.34E-5
MOLT-4	0.700	3.692	4.228	4.270	2.953	0.043	0.012	118	119	75	-94	-98	* 1.41E-6	* 2.79E-6	* 5.50E-6
RPMI-8226	4.400	9.354	10.618	10.396	9.562	0.521	0.007	126	120	105	-88	-100	* 1.92E-6	* 3.49E-6	* 6.34E-6
SR	0.057	0.586	0.721	0.639	0.051	0.002	0.000	125	110	-11	-96	-100	* 3.13E-7	* 8.07E-7	* 2.88E-6
**Non-Small Cell Lung Cancer**															
A549/ATCC	0.612	4.598	4.722	4.815	4.761	2.609	0.596	103	105	104	50	-3	* 1.00E-5	* 8.95E-5	> 1.00E-4
EKVX	2.594	7.668	8.443	8.647	7.540	4.714	2.002	115	119	98	42	-23	* 7.14E-6	* 4.44E-5	> 1.00E-4
HOP-62	1.049	3.493	3.725	3.937	3.652	1.822	0.379	110	118	106	32	-64	* 5.68E-6	* 2.14E-5	* 7.16E-5
HOP-92	8.768	10.439	11.800	11.856	11.348	8.863	3.666	182	185	155	10	-58	* 5.31E-6	* 1.40E-5	* 7.59E-5
NCI-H226	6.508	13.787	15.354	15.426	14.808	9.773	1.978	121	123	114	45	-70	* 8.39E-6	* 2.46E-5	* 6.74E-5
NCI-H23	4.317	11.617	11.884	11.178	11.396	6.417	1.515	104	94	97	29	-65	* 4.88E-6	* 2.03E-5	* 6.93E-5
NCI-H322M	4.446	12.114	13.869	13.931	13.620	9.555	4.185	123	124	120	67	-6	* 1.70E-5	* 8.30E-5	> 1.00E-4
NCI-H460	1.246	19.071	20.168	19.852	18.806	1.925	0.075	106	104	98	4	-94	* 3.25E-6	* 1.09E-5	* 3.55E-5
NCI-H522	3.641	9.084	9.771	9.639	7.201	1.368	0.024	113	110	66	-62	-99	* 1.32E-6	* 3.25E-6	* 7.99E-6
**Colon Cancer**															
COLO 205	1.187	4.822	5.388	5.424	5.553	1.380	0.463	116	117	120	5	-61	* 4.08E-6	* 1.20E-5	* 6.83E-5
HCC-2998	1.982	5.532	6.460	7.681	6.053	2.978	0.712	126	161	115	28	-64	* 5.58E-6	* 2.02E-5	* 7.04E-5
HCT-116	0.434	4.693	5.188	5.154	4.884	1.414	0.056	112	111	104	23	-87	* 4.66E-6	* 1.62E-5	* 4.61E-5
HCT-15	1.389	12.446	12.122	12.618	9.949	3.213	0.834	97	102	77	16	-40	* 2.82E-6	* 1.96E-5	> 1.00E-4
HT29	0.630	5.181	5.719	5.706	5.520	0.835	0.373	112	112	107	5	-41	* 3.62E-6	* 1.26E-5	> 1.00E-4
KM12	0.537	3.078	3.578	3.456	2.720	0.749	0.068	120	115	86	8	-87	* 2.90E-6	* 1.22E-5	* 4.07E-5
SW-620	0.598	4.140	4.621	4.695	4.251	1.712	0.180	114	116	103	32	-70	* 5.51E-6	* 2.04E-5	* 6.37E-5
**CNS Cancer**															
SF-268	0.654	2.159	2.354	2.402	2.189	1.159	0.192	113	116	102	34	-71	* 5.75E-6	* 2.10E-5	* 6.33E-5
SF-295	1.921	4.457	4.618	4.601	4.649	1.879	0.965	106	106	108	-2	-50	* 3.34E-6	* 9.52E-6	> 1.00E-4
SF-539	3.656	13.363	14.313	14.141	14.051	3.709	0.260	110	108	107	-2	-93	* 3.32E-6	* 9.51E-6	* 3.36E-5
SNB-19	1.925	4.855	5.276	5.379	5.267	3.275	1.521	114	118	114	46	-21	* 8.76E-6	* 4.87E-5	> 1.00E-4
SNB-75	1.257	2.362	2.581	2.594	2.366	1.669	0.823	120	121	100	37	-34	* 6.26E-6	* 3.30E-5	> 1.00E-4
U251	0.750	3.962	4.490	4.435	4.294	1.517	0.230	117	115	110	24	-69	* 4.99E-6	* 1.80E-5	* 6.20E-5
**Melanoma**															
LOX IMVI	0.607	3.631	3.857	3.908	3.825	1.496	0.018	107	109	106	29	-97	* 5.40E-6	* 1.71E-5	* 4.25E-5
MALME-3M	6.325	8.983	9.280	9.519	9.191	6.371	0.693	111	120	108	2	-89	* 3.50E-6	* 1.04E-5	* 3.71E-5
M14	4.271	12.275	13.258	13.768	12.277	5.057	1.253	112	119	100	10	-71	* 3.59E-6	* 1.32E-5	* 5.54E-5
MDA-MB-435	1.473	4.841	5.247	5.190	3.844	0.667	0.209	112	110	70	-55	-86	* 1.46E-6	* 3.65E-6	* 9.17E-6
SK-MEL-2	2.519	5.641	5.527	5.534	5.269	1.367	0.123	96	97	88	-46	-95	* 1.93E-6	* 4.55E-6	* 1.22E-5
SK-MEL-28	1.431	3.390	3.669	3.598	3.709	2.197	1.202	114	111	116	39	-16	* 7.23E-6	* 5.12E-5	> 1.00E-4
SK-MEL-5	3.825	11.835	12.365	11.540	10.377	3.722	0.126	107	96	82	-3	-97	* 2.37E-6	* 9.30E-6	* 3.19E-5
UACC-257	3.486	7.413	8.015	8.283	7.729	4.771	0.358	115	122	108	33	-90	* 5.89E-6	* 1.85E-5	* 4.74E-5
UACC-62	0.739	3.130	3.449	3.303	2.716	0.825	0.009	113	107	83	4	-99	* 2.59E-6	* 1.08E-5	* 3.34E-5
**Ovarian Cancer**															
IGROV1	1.018	3.274	3.719	3.704	3.472	1.880	0.505	120	119	109	38	-50	* 6.82E-6	* 2.70E-5	* 9.89E-5
OVCAR-3	4.654	18.797	20.903	21.033	19.292	3.372	0.684	115	116	104	-28	-85	* 2.56E-6	* 6.16E-6	* 2.45E-5
OVCAR-4	5.328	9.515	10.894	10.879	10.026	6.426	2.602	133	133	112	26	-51	* 5.29E-6	* 2.18E-5	* 9.66E-5
OVCAR-5	6.474	14.864	15.972	16.062	15.468	11.862	5.951	113	114	107	64	-8	* 1.57E-5	* 7.73E-5	> 1.00E-4
OVCAR-8	0.673	3.741	4.224	3.984	3.788	1.701	0.078	116	108	102	34	-88	* 5.73E-6	* 1.88E-5	* 4.84E-5
NCI/ADR-RES	3.746	11.148	12.620	12.555	11.716	1.142	1.006	120	119	108	-70	-73	* 2.12E-6	* 4.05E-6	* 7.76E-6
SK-OV-3	2.611	9.944	9.752	10.985	9.153	3.398	0.960	98	114	89	11	-63	* 3.18E-6	* 1.40E-5	* 6.63E-5
**Renal Cancer**															
786-0	1.778	6.542	6.403	6.386	6.393	2.418	0.904	97	97	97	13	-49	* 3.65E-6	* 1.64E-5	> 1.00E-4
A498	1.927	7.664	8.097	8.171	7.607	3.657	0.905	108	109	99	30	-53	* 5.15E-6	* 2.30E-5	* 9.20E-5
ACHN	1.537	.	.	.	.	.	.	.	.	.	.	.	.	.	.
CAKI-1	1.045	4.222	4.474	4.524	4.105	2.074	0.253	108	110	96	32	-76	* 5.30E-6	* 1.99E-5	* 5.78E-5
RXF 393	1.802	3.132	3.165	3.102	3.188	1.314	0.416	103	98	104	-27	-77	* 2.58E-6	* 6.22E-6	* 2.88E-5
SN12C	0.623	2.414	2.484	2.567	2.536	1.392	0.188	104	109	107	43	-70	* 7.75E-6	* 2.40E-5	* 6.66E-5
TK-10	6.290	17.260	18.161	18.438	18.106	12.515	4.169	108	111	108	57	-34	* 1.19E-5	* 4.24E-5	> 1.00E-4
UO-31	1.550	5.913	6.041	5.936	5.845	3.479	1.242	103	101	98	44	-20	* 7.82E-6	* 4.89E-5	> 1.00E-4
**Prostate Cancer**															
PC-3	3.978	11.159	12.855	12.664	11.706	5.949	0.799	124	121	108	28	-80	* 5.23E-6	* 1.80E-5	* 5.27E-5
DU-145	0.636	3.766	4.127	4.060	3.911	1.328	0.313	112	109	105	22	-51	* 4.60E-6	* 2.01E-5	* 9.74E-5
**Breast Cancer**															
MCF7	2.550	16.169	13.962	13.140	14.217	4.366	0.527	84	78	86	13	-79	* 3.11E-6	* 1.39E-5	* 4.82E-5
MDA-MB-231/ATCC	5.261	12.044	12.870	12.802	12.101	8.371	4.595	112	111	101	46	-13	* 8.40E-6	* 6.08E-5	> 1.00E-4
HS 578T	1.006	2.636	2.582	2.657	2.726	0.658	0.272	97	102	105	-35	-73	* 2.48E-6	* 5.65E-6	* 2.52E-5
BT-549	3.822	8.395	9.468	9.695	7.865	4.414	0.068	123	128	88	13	-98	* 3.23E-6	* 1.31E-5	* 3.68E-5
T-47D	5.838	11.611	11.401	11.528	11.462	8.132	0.364	96	99	97	40	-94	* 6.63E-6	* 1.98E-5	* 4.70E-5
MDA-MB-468	6.310	10.765	10.756	10.586	10.830	1.490	2.304	100	96	102	-76	-64	* 1.95E-6	* 3.73E-6	* 7.11E-6

NCI five dose results of compound **11a**

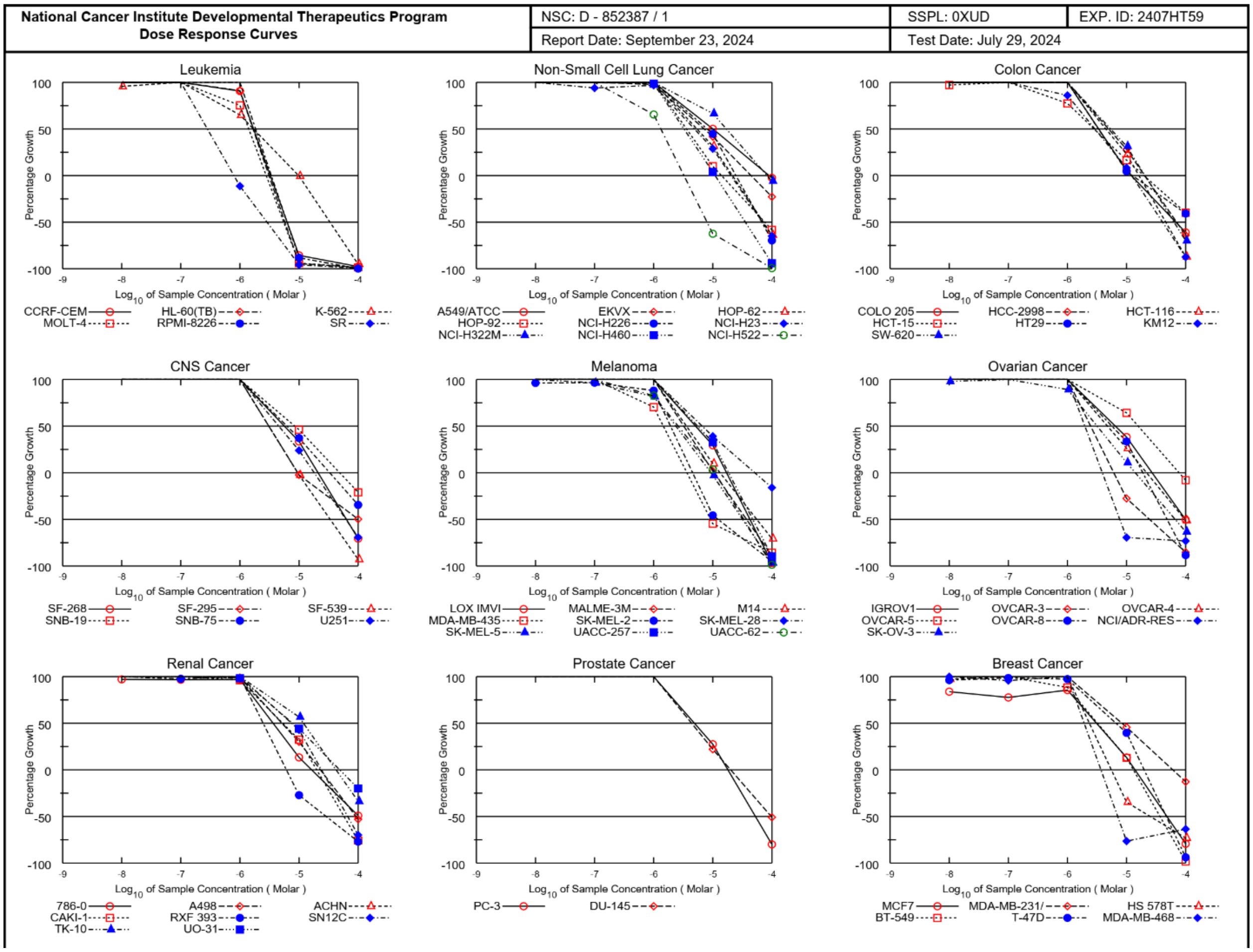

NCI five dose results of compound **11a**

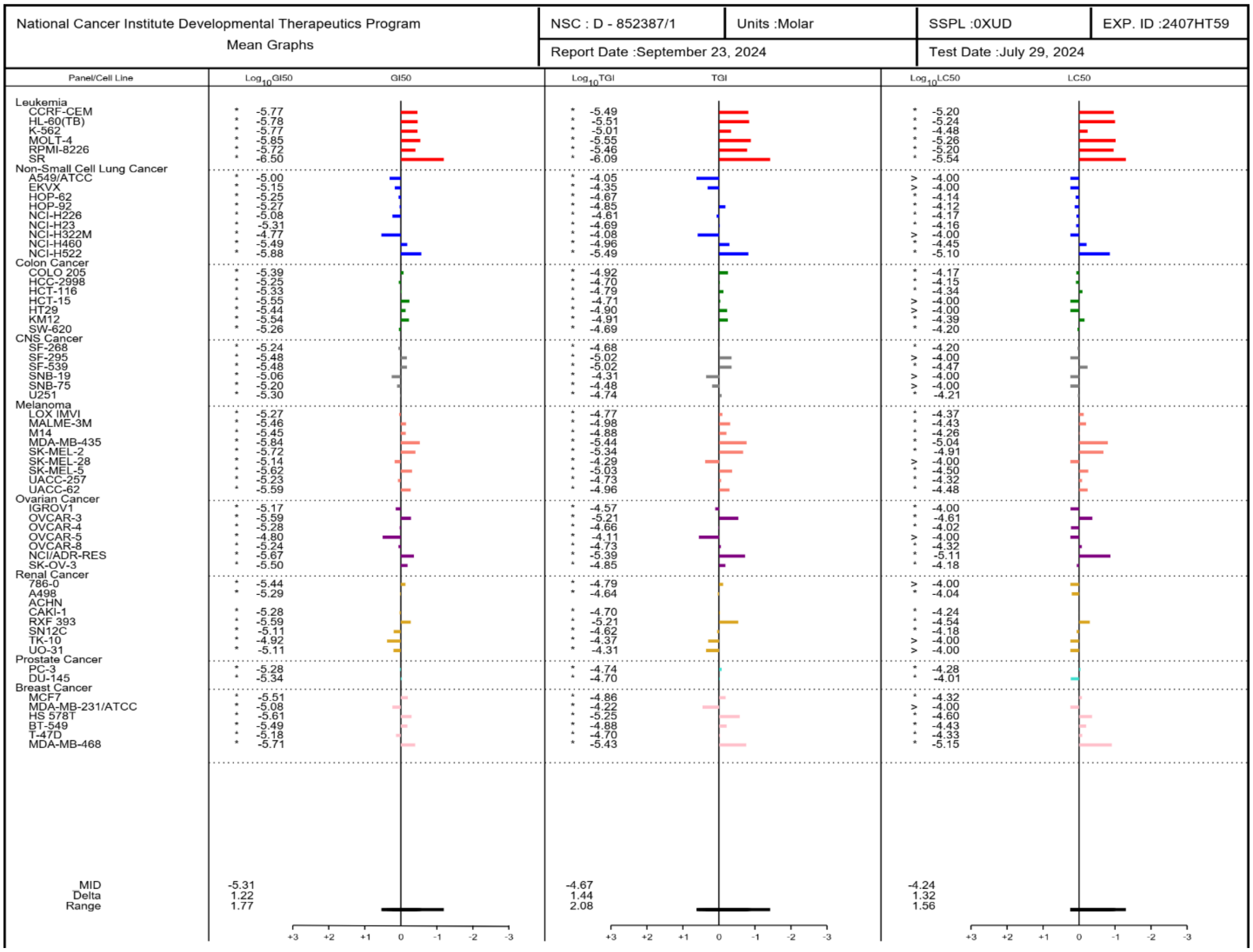

NCI five dose results of compound **11a**

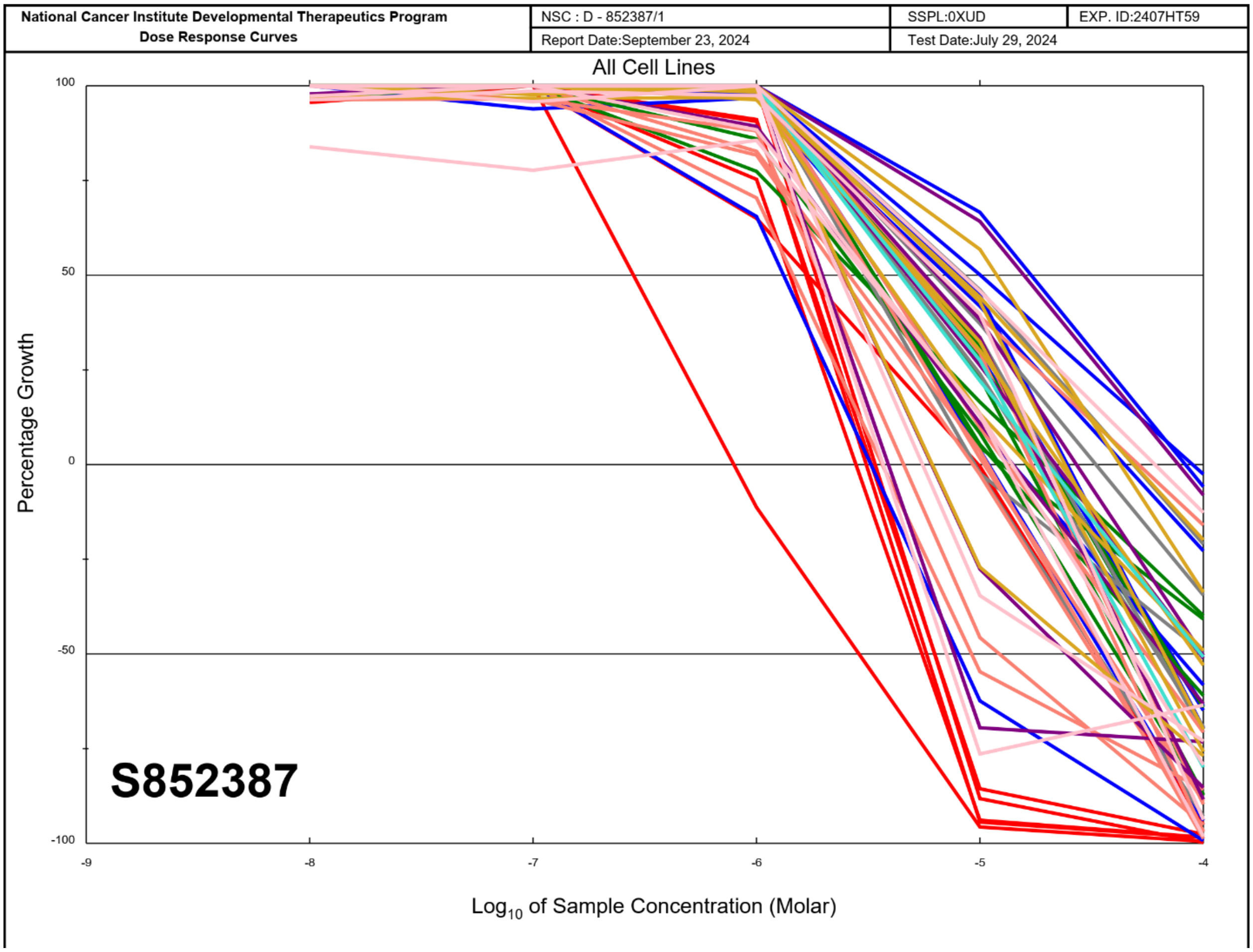

NCI five dose results of compound **11a**

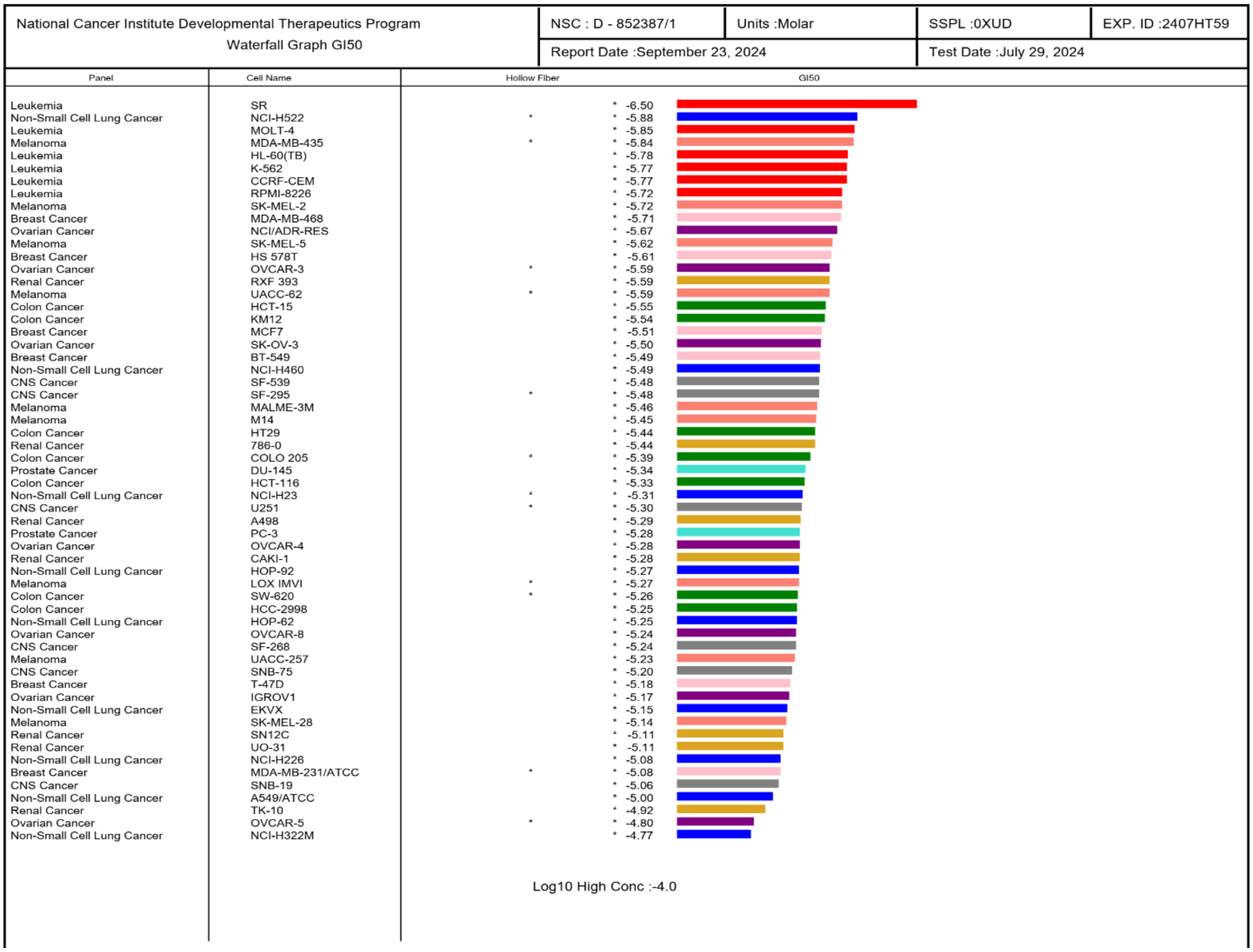

NCI five dose results of compound **11a**

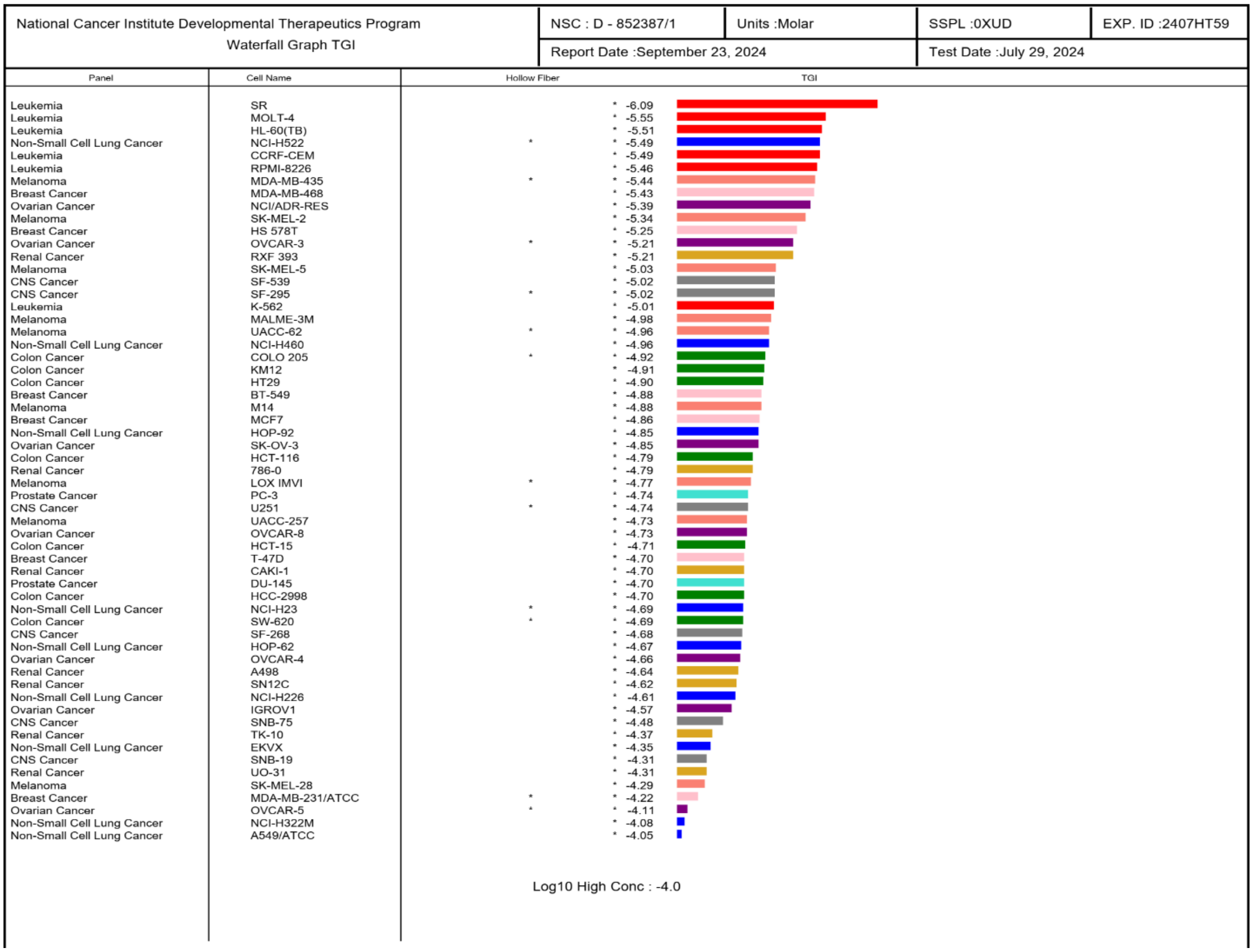

NCI five dose results of compound **11a**

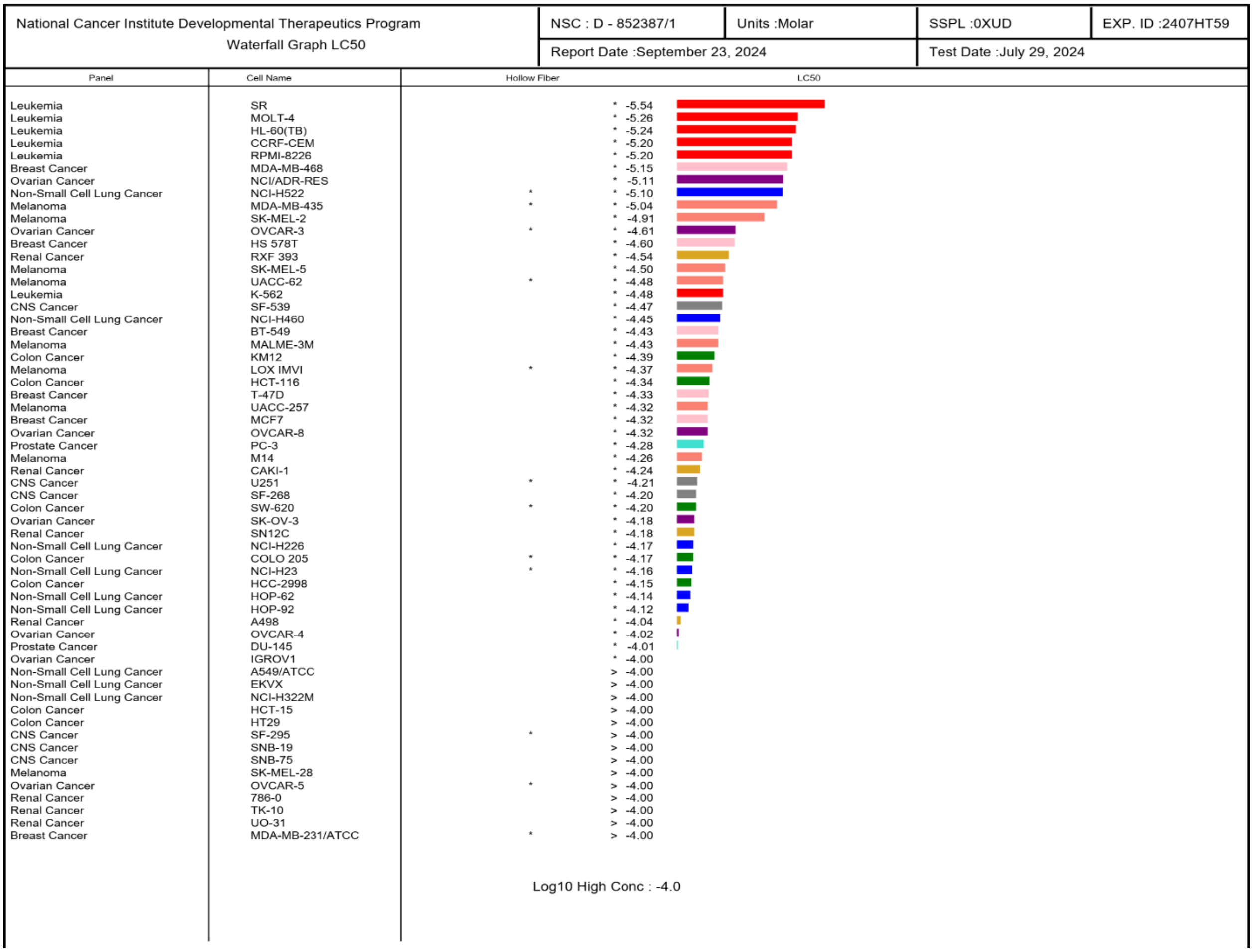

NCI five dose results of compound **11a**

National Cancer Institute Developmental Therapeutics Program
In-Vitro Testing Results

NSC : D - 852385 / 1	Experiment ID : 2407HT59	Test Type : HTS	Units : Molar
Report Date : September 23, 2024	Test Date : July 29, 2024	QNS :	MC :
COMI : FA-H	Stain Reagent :	SSPL : 0XUD	

			Log10 Concentration												
	Time		Mean Optical Densities					Percent Growth							
Panel/Cell Line	Zero	Ctrl	-8.0	-7.0	-6.0	-5.0	-4.0	-8.0	-7.0	-6.0	-5.0	-4.0	GI50	TGI	LC50
**Leukemia**															
CCRF-CEM	0.676	4.761	4.951	4.818	4.443	0.058	0.039	105	101	92	-91	-94	* 1.70E-6	* 3.18E-6	* 5.95E-6
HL-60(TB)	0.587	3.503	3.734	3.875	3.600	0.013	0.001	108	113	104	-98	-100	* 1.84E-6	* 3.27E-6	* 5.79E-6
K-562	0.506	7.319	7.101	7.091	5.500	0.441	0.091	97	97	73	-13	-82	* 1.87E-6	* 7.09E-6	* 3.44E-5
MOLT-4	0.700	3.692	4.178	4.277	2.992	0.026	0.001	116	120	77	-96	-100	* 1.43E-6	* 2.78E-6	* 5.40E-6
RPMI-8226	4.400	9.354	8.587	8.620	8.068	0.234	0.014	85	85	73	-95	-100	* 1.36E-6	* 2.72E-6	* 5.41E-6
SR	0.057	0.586	0.660	0.611	0.024	0.001	0.000	114	105	-58	-98	-100	* 2.17E-7	* 4.41E-7	* 8.95E-7
**Non-Small Cell Lung Cancer**															
A549/ATCC	0.612	4.598	4.770	4.501	4.708	2.188	0.361	104	98	103	40	-41	* 6.83E-6	* 3.10E-5	> 1.00E-4
EKVX	2.594	7.668	8.052	7.587	7.668	4.652	1.354	108	99	100	41	-48	* 6.96E-6	* 2.88E-5	> 1.00E-4
HOP-62	1.049	3.493	3.576	3.339	3.250	1.770	0.182	103	94	90	30	-83	* 4.59E-6	* 1.83E-5	* 5.11E-5
HOP-92	8.768	10.439	11.258	11.443	10.930	7.761	1.822	150	160	130	-12	-79	* 3.68E-6	* 8.30E-6	* 3.70E-5
NCI-H226	6.508	13.787	14.695	14.602	14.197	8.820	1.587	112	111	106	32	-76	* 5.67E-6	* 1.98E-5	* 5.77E-5
NCI-H23	4.317	11.617	11.057	11.822	10.787	6.255	1.389	92	103	89	26	-68	* 4.19E-6	* 1.91E-5	* 6.47E-5
NCI-H322M	4.446	12.114	13.029	12.800	13.029	9.296	2.645	112	109	112	63	-40	* 1.34E-5	* 4.07E-5	> 1.00E-4
NCI-H460	1.246	19.071	17.695	18.334	17.402	2.283	0.025	92	96	91	6	-98	* 3.01E-6	* 1.14E-5	* 3.45E-5
NCI-H522	3.641	9.084	9.621	9.672	7.198	1.085	0.468	110	111	66	-70	-87	* 1.30E-6	* 3.04E-6	* 7.10E-6
**Colon Cancer**															
COLO 205	1.187	4.822	4.656	4.832	4.945	1.460	0.704	95	100	103	8	-41	* 3.60E-6	* 1.43E-5	> 1.00E-4
HCC-2998	1.982	5.532	4.052	4.876	5.480	2.410	0.270	58	82	98	12	-86	* 3.64E-6	* 1.33E-5	* 4.27E-5
HCT-116	0.434	4.693	5.188	4.790	4.581	1.411	0.035	112	102	97	23	-92	* 4.33E-6	* 1.58E-5	* 4.32E-5
HCT-15	1.389	12.446	8.739	13.724	10.987	4.918	0.645	66	112	87	32	-54	* 4.68E-6	* 2.36E-5	* 9.09E-5
HT29	0.630	5.181	5.194	5.111	5.553	0.935	0.306	100	98	108	7	-51	* 3.75E-6	* 1.31E-5	* 9.48E-5
KM12	0.537	3.078	3.095	3.172	2.862	0.790	0.079	101	104	92	10	-85	* 3.23E-6	* 1.27E-5	* 4.26E-5
SW-620	0.598	4.140	4.364	4.498	4.280	1.543	0.221	106	110	104	27	-63	* 4.99E-6	* 1.98E-5	* 7.14E-5
**CNS Cancer**															
SF-268	0.654	2.159	2.217	2.235	2.143	1.016	0.116	104	105	99	24	-82	* 4.50E-6	* 1.68E-5	* 4.97E-5
SF-295	1.921	4.457	4.581	4.590	4.731	1.518	0.727	105	105	111	-21	-62	* 2.89E-6	* 6.93E-6	* 5.06E-5
SF-539	3.656	13.363	14.809	14.563	14.494	3.011	0.112	115	112	112	-18	-97	* 3.00E-6	* 7.30E-6	* 2.56E-5
SNB-19	1.925	4.855	5.141	5.215	5.088	3.172	1.102	110	112	108	43	-43	* 7.70E-6	* 3.16E-5	> 1.00E-4
SNB-75	1.257	2.362	2.356	2.319	2.216	1.674	0.325	100	96	87	38	-74	* 5.59E-6	* 2.17E-5	* 6.08E-5
U251	0.750	3.962	4.189	4.351	4.220	1.295	0.150	107	112	108	17	-80	* 4.33E-6	* 1.49E-5	* 4.90E-5
**Melanoma**															
LOX IMVI	0.607	3.631	3.974	4.002	3.601	1.293	0.012	111	112	99	23	-98	* 4.39E-6	* 1.54E-5	* 4.00E-5
MALME-3M	6.325	8.983	8.839	9.021	8.908	6.334	1.568	95	101	97	1	-75	* 3.08E-6	* 1.02E-5	* 4.65E-5
M14	4.271	12.275	12.176	12.446	11.637	4.497	2.136	99	102	92	3	-50	* 2.96E-6	* 1.13E-5	> 1.00E-4
MDA-MB-435	1.473	4.841	4.480	4.587	3.761	0.679	0.189	89	92	68	-54	-87	* 1.40E-6	* 3.61E-6	* 9.30E-6
SK-MEL-2	2.519	5.641	5.656	5.722	5.217	1.038	0.101	100	103	86	-59	-96	* 1.78E-6	* 3.94E-6	* 8.70E-6
SK-MEL-28	1.431	3.390	3.615	3.669	3.647	2.190	0.837	111	114	113	39	-42	* 7.06E-6	* 3.04E-5	> 1.00E-4
SK-MEL-5	3.825	11.835	10.726	10.337	10.330	3.542	0.069	86	81	81	-7	-98	* 2.25E-6	* 8.25E-6	* 2.95E-5
UACC-257	3.486	7.413	7.841	7.918	7.372	4.149	0.728	111	113	99	17	-79	* 3.95E-6	* 1.50E-5	* 4.97E-5
UACC-62	0.739	3.130	3.068	2.966	2.571	0.699	0.075	97	93	77	-5	-90	* 2.11E-6	* 8.58E-6	* 3.38E-5
**Ovarian Cancer**															
IGROV1	1.018	3.274	3.599	3.376	3.173	1.868	0.419	114	105	96	38	-59	* 6.13E-6	* 2.46E-5	* 8.10E-5
OVCAR-3	4.654	18.797	19.037	18.362	17.077	4.038	0.983	102	97	88	-13	-79	* 2.37E-6	* 7.40E-6	* 3.63E-5
OVCAR-4	5.328	9.515	10.482	10.493	10.133	5.928	2.281	123	123	115	14	-57	* 4.41E-6	* 1.59E-5	* 7.93E-5
OVCAR-5	6.474	14.864	14.489	15.307	15.164	12.252	4.845	96	105	104	69	-25	* 1.59E-5	* 5.40E-5	> 1.00E-4
OVCAR-8	0.673	3.741	3.592	3.864	3.542	1.686	0.119	95	104	94	33	-82	* 5.24E-6	* 1.93E-5	* 5.25E-5
NCI/ADR-RES	3.746	11.148	11.677	11.977	10.568	0.932	0.816	107	111	92	-75	-78	* 1.79E-6	* 3.55E-6	* 7.08E-6
SK-OV-3	2.611	9.944	10.846	11.977	9.241	4.131	0.611	112	128	91	21	-77	* 3.84E-6	* 1.64E-5	* 5.33E-5
**Renal Cancer**															
786-0	1.778	6.542	6.272	6.629	6.409	2.814	0.495	94	102	97	22	-72	* 4.22E-6	* 1.70E-5	* 5.81E-5
A498	1.927	7.664	8.125	8.609	7.274	3.347	0.168	108	117	93	25	-91	* 4.28E-6	* 1.64E-5	* 4.41E-5
ACHN	1.537	.	.	.	.	.	.	.	.	.	.	.	.	.	.
CAKI-1	1.045	4.222	4.434	4.410	4.278	1.867	0.228	107	106	102	26	-78	* 4.81E-6	* 1.77E-5	* 5.36E-5
RXF 393	1.802	3.132	3.345	3.306	3.204	1.337	0.273	116	113	105	-26	-85	* 2.64E-6	* 6.36E-6	* 2.57E-5
SN12C	0.623	2.414	2.649	2.684	2.643	1.307	0.160	113	115	113	38	-74	* 6.94E-6	* 2.19E-5	* 6.08E-5
TK-10	6.290	17.260	19.164	18.230	18.597	11.616	3.049	117	109	112	48	-52	* 9.49E-6	* 3.06E-5	* 9.65E-5
UO-31	1.550	5.913	6.090	6.117	6.130	3.698	1.150	104	105	105	49	-26	* 9.68E-6	* 4.53E-5	> 1.00E-4
**Prostate Cancer**															
PC-3	3.978	11.159	12.116	12.508	10.944	5.378	1.197	113	119	97	19	-70	* 4.04E-6	* 1.65E-5	* 5.98E-5
DU-145	0.636	3.766	3.635	3.905	3.598	1.377	0.148	96	104	95	24	-77	* 4.26E-6	* 1.72E-5	* 5.41E-5
**Breast Cancer**															
MCF7	2.550	16.169	16.709	17.124	15.222	4.551	0.711	104	107	93	15	-72	* 3.54E-6	* 1.48E-5	* 5.56E-5
MDA-MB-231/ATCC	5.261	12.044	12.330	11.947	12.026	8.423	2.927	104	99	100	47	-44	* 8.65E-6	* 3.25E-5	> 1.00E-4
HS 578T	1.006	2.636	2.714	2.871	2.581	0.625	0.188	106	115	95	-38	-81	* 2.18E-6	* 5.18E-6	* 1.90E-5
BT-549	3.822	8.395	8.987	9.451	8.146	3.471	0.208	113	123	94	-9	-94	* 2.69E-6	* 8.15E-6	* 3.01E-5
T-47D	5.838	11.611	11.192	11.714	11.416	7.693	2.409	93	102	97	32	-59	* 5.29E-6	* 2.26E-5	* 8.01E-5
MDA-MB-468	6.310	10.765	11.004	10.826	9.686	1.997	1.289	106	102	76	-68	-80	* 1.52E-6	* 3.36E-6	* 7.46E-6

NCI five dose results of compound **11b**

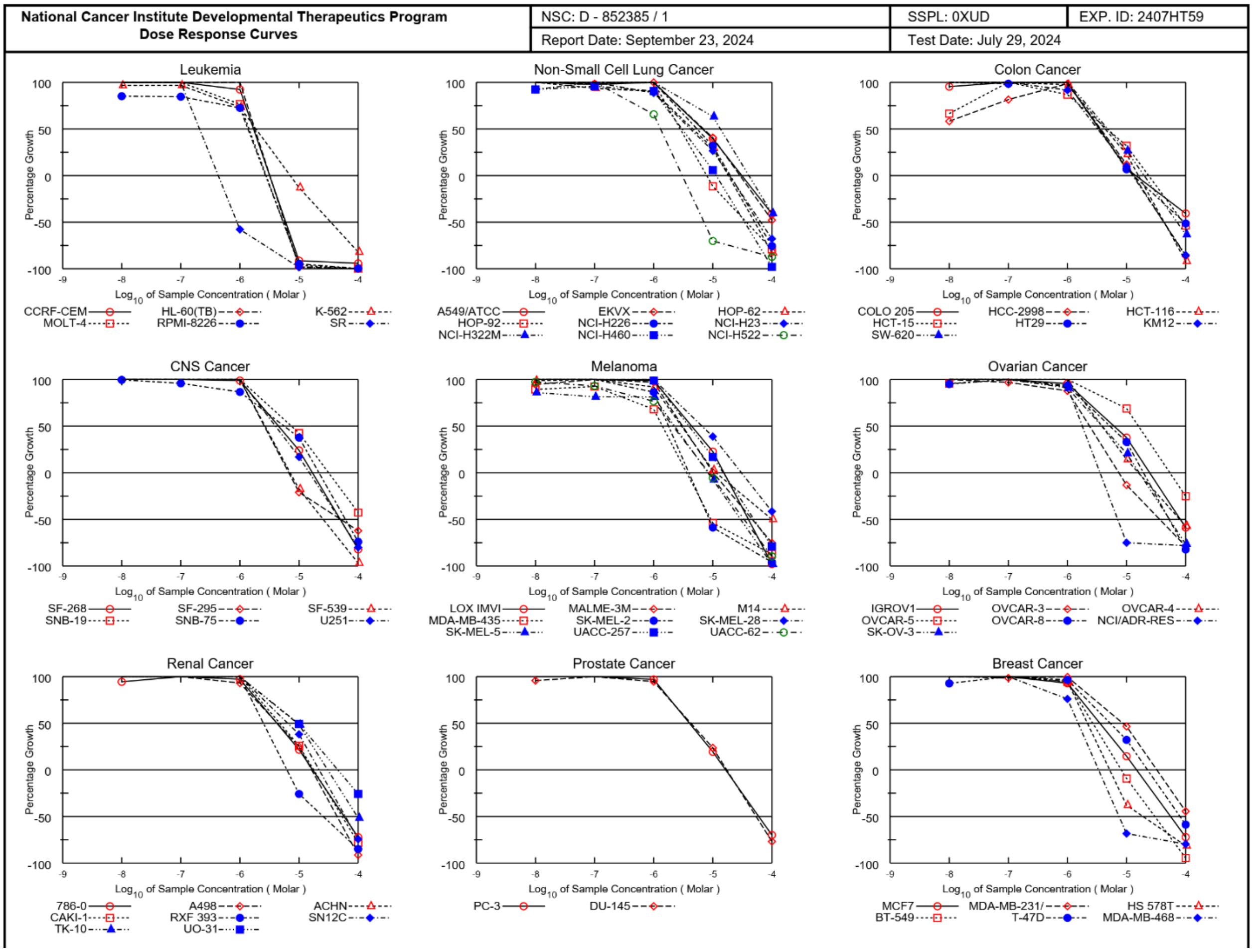

NCI five dose results of compound **11b**

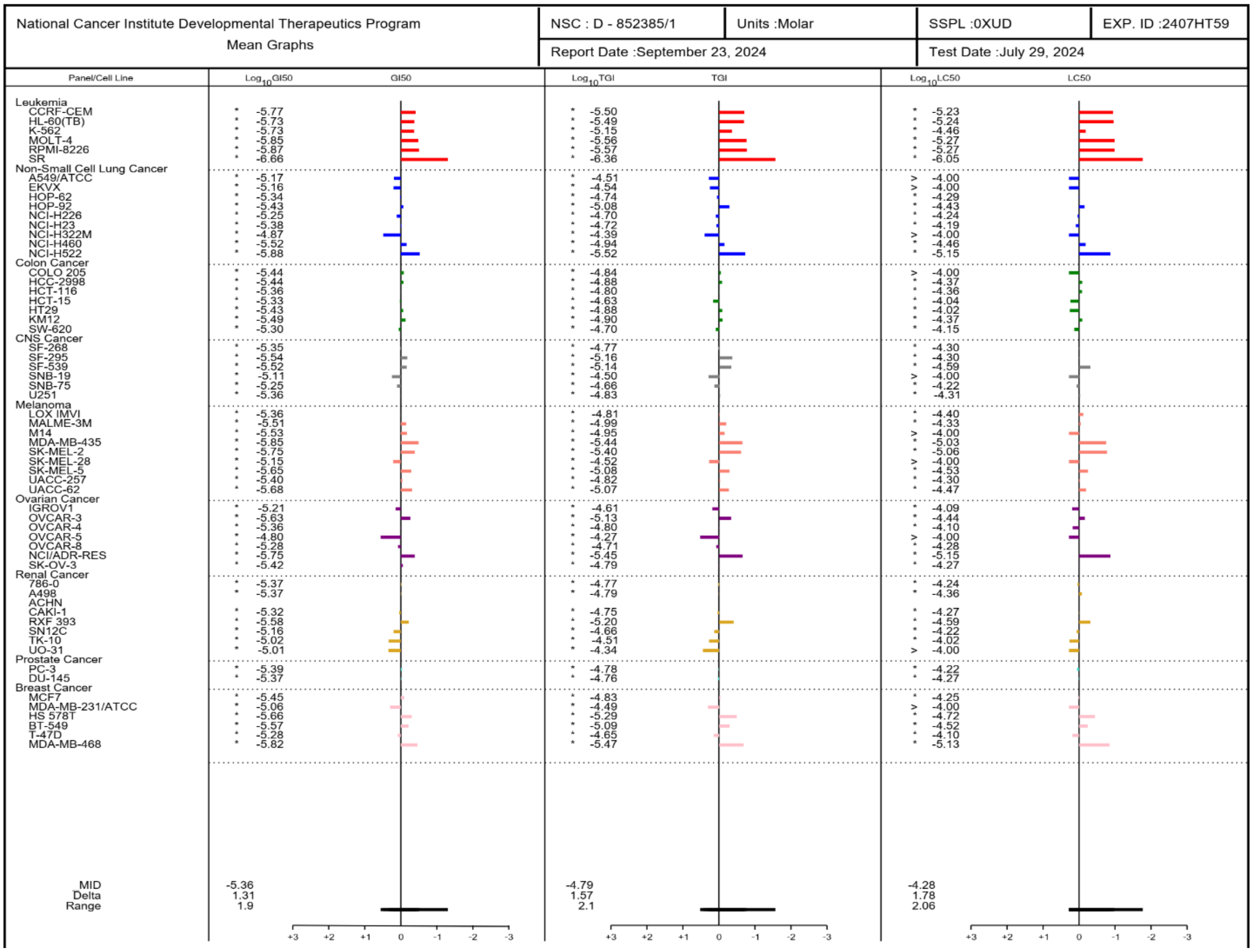

NCI five dose results of compound **11b**

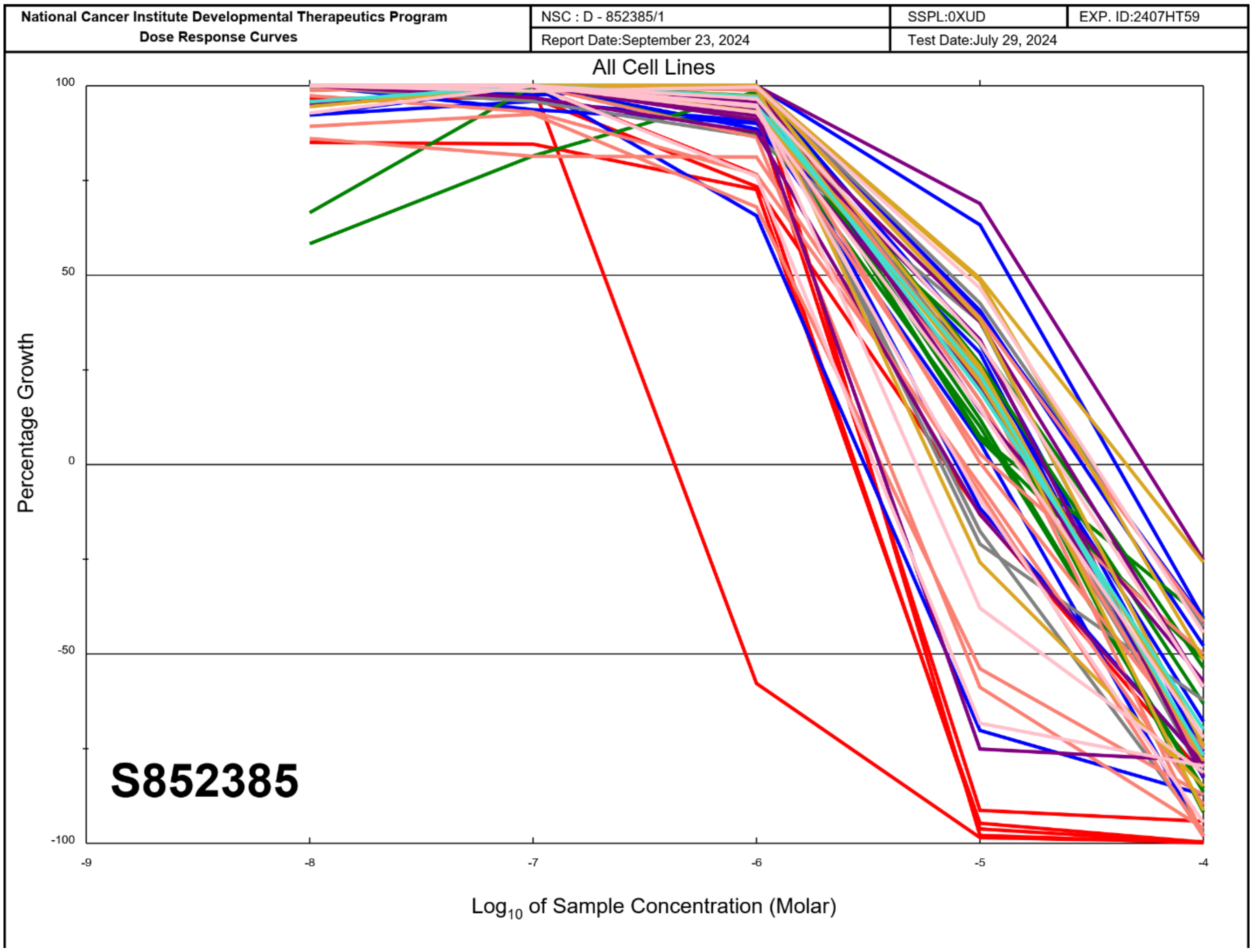

NCI five dose results of compound **11b**

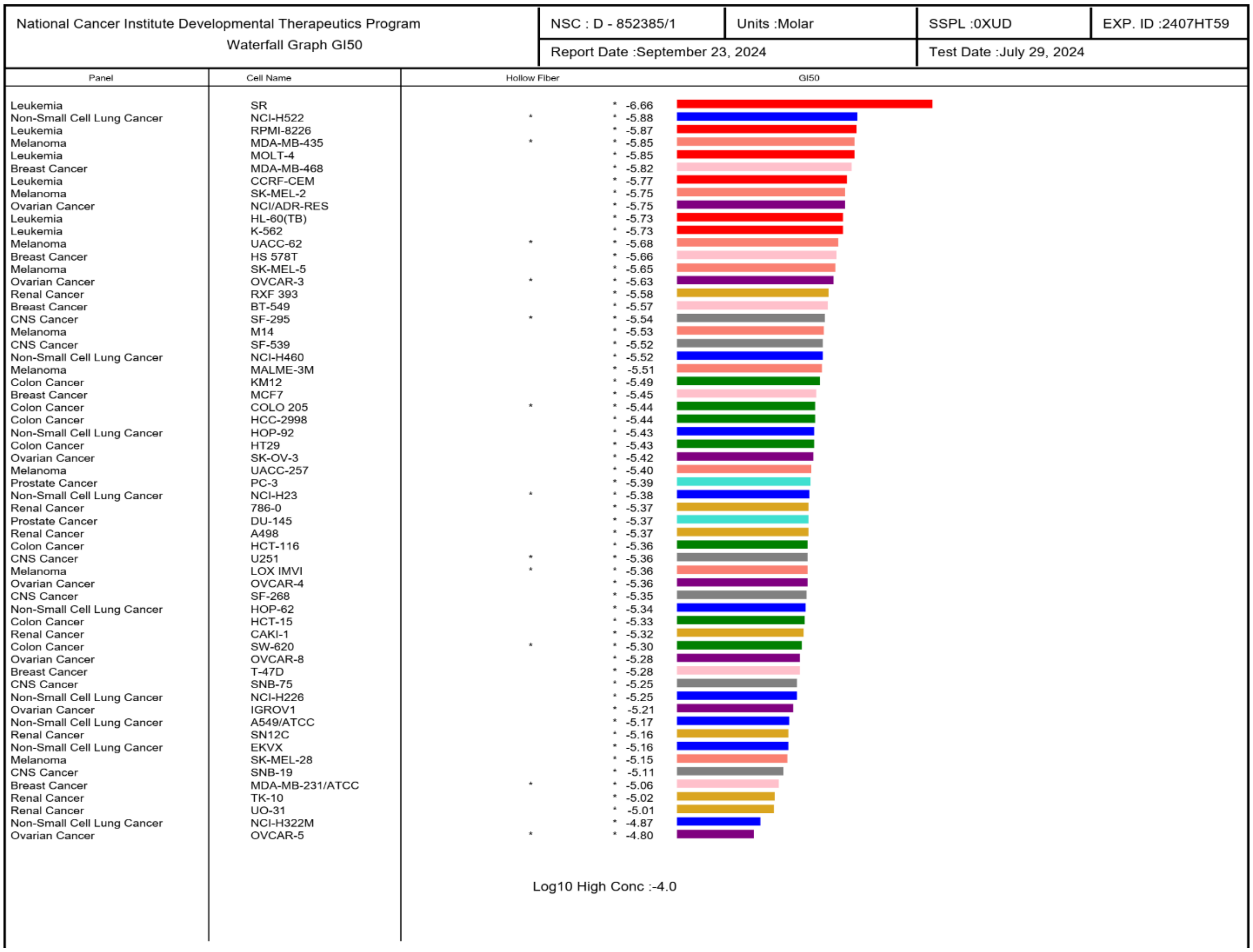

NCI five dose results of compound **11b**

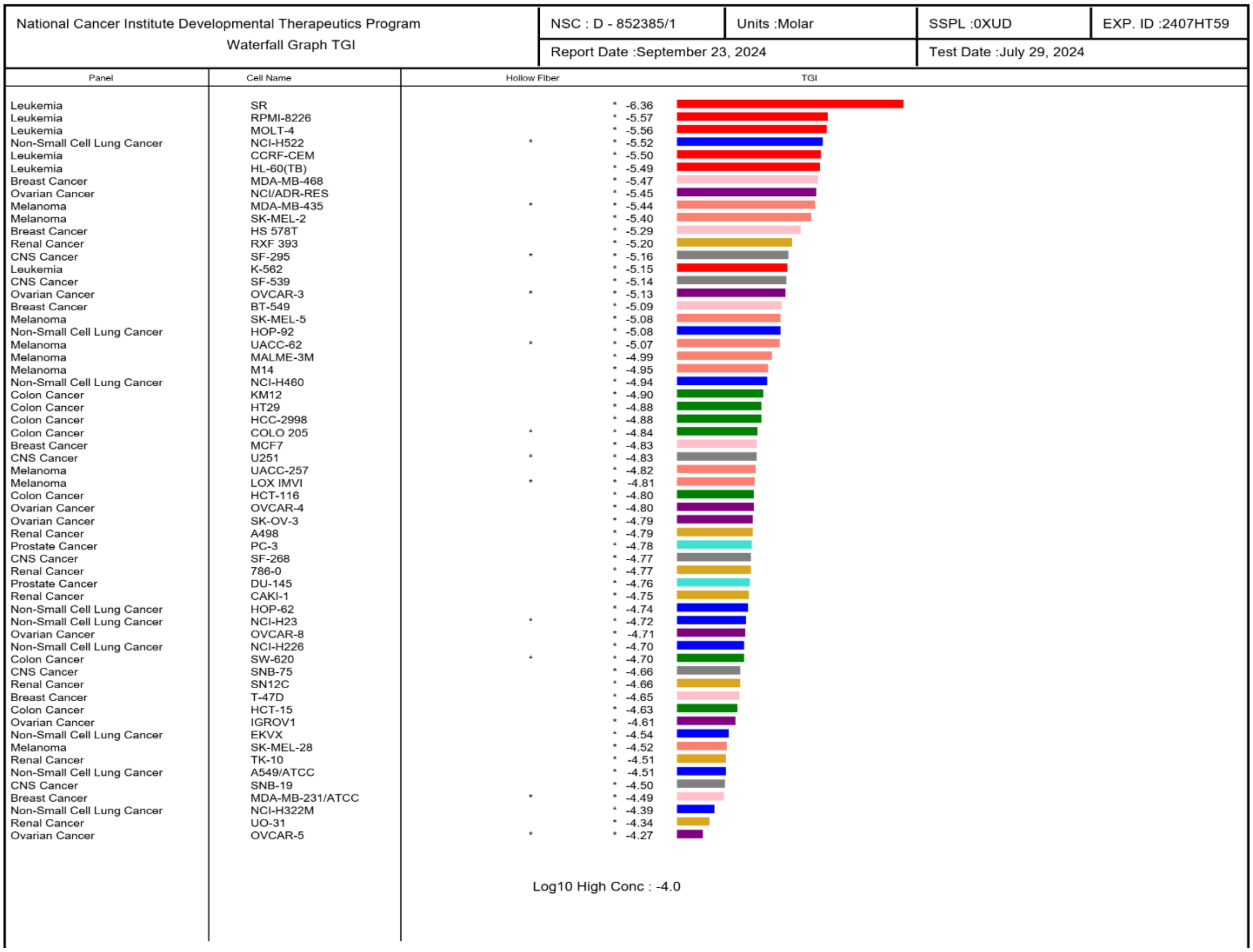

NCI five dose results of compound **11b**

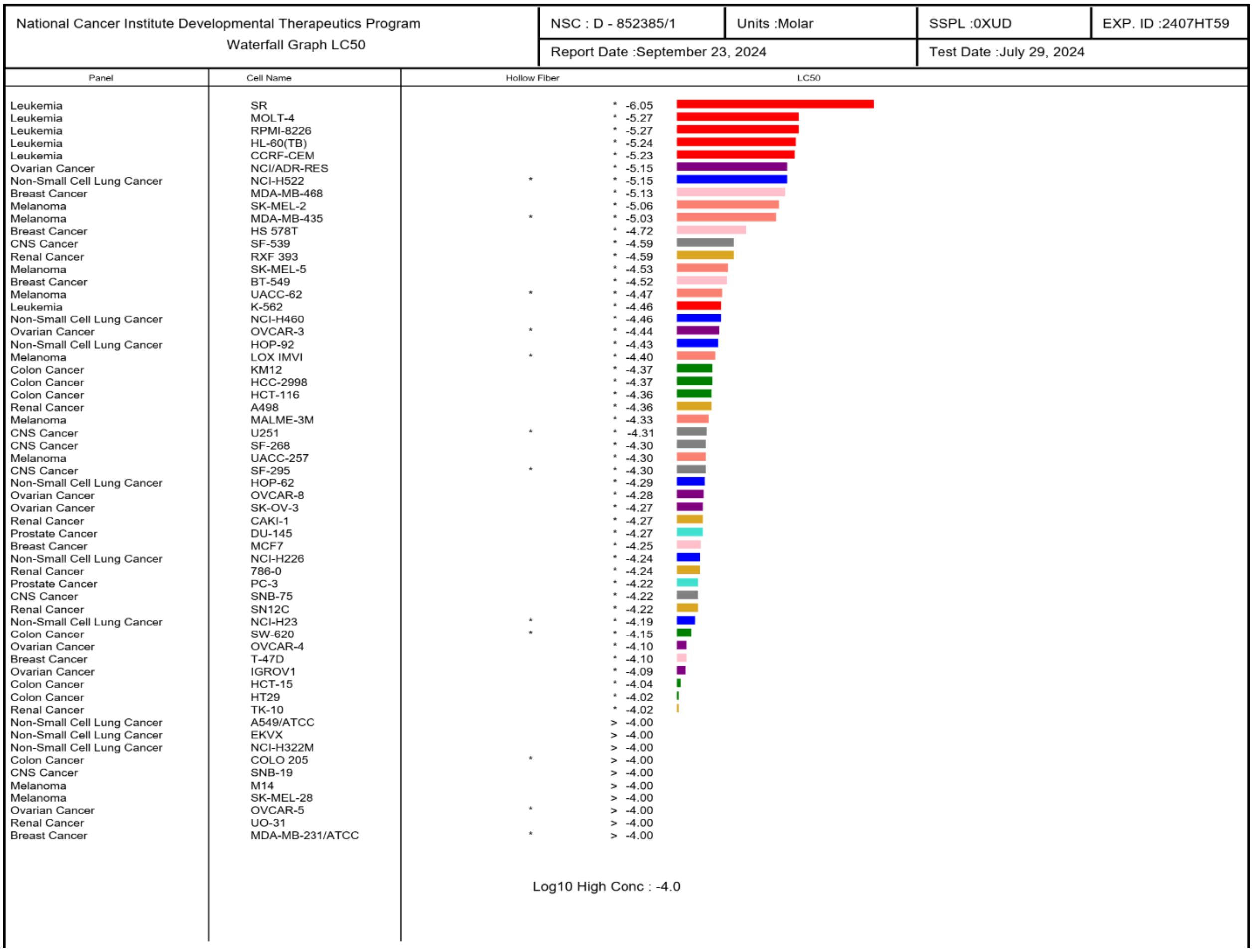

NCI five dose results of compound **11b**

National Cancer Institute Developmental Therapeutics Program
In-Vitro Testing Results

NSC : D - 852390 / 1	Experiment ID : 2407HT59	Test Type : HTS	Units : Molar
Report Date : September 23, 2024	Test Date : July 29, 2024	QNS :	MC :
COMI : MA-H	Stain Reagent :	SSPL : 0XUD	

Panel/Cell Line	Time Zero	Ctrl	Mean Optical Densities (Log10 Concentration) -8.0	-7.0	-6.0	-5.0	-4.0	Percent Growth (Log10 Concentration) -8.0	-7.0	-6.0	-5.0	-4.0	GI50	TGI	LC50
**Leukemia**															
CCRF-CEM	0.676	4.761	5.017	4.828	5.035	1.361	0.752	106	101	107	17	2	* 4.27E-6	> 1.00E-4	> 1.00E-4
HL-60(TB)	0.587	3.503	3.635	3.950	4.043	0.220	0.008	104	115	118	-62	-99	* 2.39E-6	* 4.51E-6	* 8.53E-6
K-562	0.506	7.319	7.399	7.137	7.275	2.263	1.625	101	97	99	26	16	* 4.70E-6	> 1.00E-4	> 1.00E-4
MOLT-4	0.700	3.692	4.136	4.310	4.127	0.220	0.018	115	121	115	-69	-97	* 2.25E-6	* 4.22E-6	* 7.92E-6
RPMI-8226	4.400	9.354	10.540	10.298	10.391	0.350	0.054	125	120	121	-92	-99	* 2.15E-6	* 3.70E-6	* 6.35E-6
SR	0.057	0.586	0.676	0.602	0.307	0.002	0.002	117	103	47	-96	-97	* 8.89E-7	* 2.13E-6	* 4.75E-6
**Non-Small Cell Lung Cancer**															
A549/ATCC	0.612	4.598	4.806	4.770	4.803	2.306	1.154	105	104	105	42	14	* 7.59E-6	> 1.00E-4	> 1.00E-4
EKVX	2.594	7.668	8.798	8.736	8.352	5.532	4.251	122	121	114	58	33	* 2.07E-5	> 1.00E-4	> 1.00E-4
HOP-62	1.049	3.493	3.956	3.614	3.864	2.407	1.199	119	105	115	56	6	* 1.30E-5	> 1.00E-4	> 1.00E-4
HOP-92	8.768	10.439	11.441	11.292	10.940	8.721	4.797	160	152	130	1	-45	* 4.16E-6	* 1.04E-5	> 1.00E-4
NCI-H226	6.508	13.787	15.651	15.198	14.887	9.170	4.280	126	119	115	37	-34	* 6.76E-6	* 3.29E-5	> 1.00E-4
NCI-H23	4.317	11.617	12.062	11.976	11.669	9.114	4.502	106	105	101	66	3	* 1.77E-5	> 1.00E-4	> 1.00E-4
NCI-H322M	4.446	12.114	13.450	14.005	13.926	9.998	8.198	117	125	124	72	49	* 8.99E-5	> 1.00E-4	> 1.00E-4
NCI-H460	1.246	19.071	20.185	19.800	20.617	4.716	2.502	106	104	109	20	7	* 4.55E-6	> 1.00E-4	> 1.00E-4
NCI-H522	3.641	9.084	9.167	9.355	8.986	4.636	2.890	101	105	98	18	-21	* 4.02E-6	* 2.97E-5	> 1.00E-4
**Colon Cancer**															
COLO 205	1.187	4.822	5.637	5.701	5.784	5.698	2.298	122	124	126	124	30	* 6.20E-5	> 1.00E-4	> 1.00E-4
HCC-2998	1.982	5.532	6.625	6.483	5.181	3.479	3.617	131	127	90	42	46	* 6.86E-6	> 1.00E-4	> 1.00E-4
HCT-116	0.434	4.693	5.020	5.078	5.156	2.407	0.865	108	109	111	46	10	* 8.77E-6	> 1.00E-4	> 1.00E-4
HCT-15	1.389	12.446	11.533	11.317	10.503	7.354	4.400	92	90	82	54	27	* 1.40E-5	> 1.00E-4	> 1.00E-4
HT29	0.630	5.181	5.557	5.493	5.705	4.098	1.862	108	107	112	76	27	* 3.42E-5	> 1.00E-4	> 1.00E-4
KM12	0.537	3.078	3.630	3.640	3.580	1.545	0.603	122	122	120	40	3	* 7.41E-6	> 1.00E-4	> 1.00E-4
SW-620	0.598	4.140	4.836	4.808	4.812	4.071	2.943	120	119	119	98	66	> 1.00E-4	> 1.00E-4	> 1.00E-4
**CNS Cancer**															
SF-268	0.654	2.159	2.238	2.299	2.212	1.538	1.207	105	109	104	59	37	* 2.50E-5	> 1.00E-4	> 1.00E-4
SF-295	1.921	4.457	4.435	4.460	4.450	4.032	2.276	99	100	100	83	14	* 3.02E-5	> 1.00E-4	> 1.00E-4
SF-539	3.656	13.363	13.691	13.913	13.904	3.556	3.502	103	106	106	-7	-4	* 3.11E-6	* 8.65E-6	> 1.00E-4
SNB-19	1.925	4.855	5.194	5.236	5.182	4.188	3.382	112	113	111	77	50	* 9.79E-5	> 1.00E-4	> 1.00E-4
SNB-75	1.257	2.362	2.555	2.566	2.561	1.334	1.308	117	118	118	7	4	* 4.08E-6	> 1.00E-4	> 1.00E-4
U251	0.750	3.962	4.424	4.564	4.391	2.421	1.483	114	119	113	52	23	* 1.17E-5	> 1.00E-4	> 1.00E-4
**Melanoma**															
LOX IMVI	0.607	3.631	4.020	3.830	3.946	2.084	0.975	113	107	110	49	12	* 9.58E-6	> 1.00E-4	> 1.00E-4
MALME-3M	6.325	8.983	9.529	9.473	9.450	7.279	5.562	121	118	118	36	-12	* 6.71E-6	* 5.60E-5	> 1.00E-4
M14	4.271	12.275	13.299	13.910	13.832	8.471	5.954	113	120	119	52	21	* 1.20E-5	> 1.00E-4	> 1.00E-4
MDA-MB-435	1.473	4.841	5.515	5.448	5.638	3.514	2.935	120	118	124	61	43	* 4.15E-5	> 1.00E-4	> 1.00E-4
SK-MEL-2	2.519	5.641	5.519	5.582	5.107	1.420	1.776	96	98	83	-44	-30	* 1.82E-6	* 4.52E-6	> 1.00E-4
SK-MEL-28	1.431	3.390	3.687	3.658	3.732	2.993	2.005	115	114	117	80	29	* 3.89E-5	> 1.00E-4	> 1.00E-4
SK-MEL-5	3.825	11.835	10.519	12.375	12.843	8.770	3.924	84	107	112	62	-1	* 1.55E-5	* 9.71E-5	> 1.00E-4
UACC-257	3.486	7.413	8.137	8.373	8.582	4.839	4.588	118	124	130	34	28	* 6.88E-6	> 1.00E-4	> 1.00E-4
UACC-62	0.739	3.130	3.359	3.198	3.504	2.275	0.957	110	103	116	64	9	* 1.81E-5	> 1.00E-4	> 1.00E-4
**Ovarian Cancer**															
IGROV1	1.018	3.274	3.768	3.704	3.642	2.985	1.464	122	119	116	87	20	* 3.56E-5	> 1.00E-4	> 1.00E-4
OVCAR-3	4.654	18.797	18.940	19.826	19.603	9.814	7.540	101	107	106	36	20	* 6.37E-6	> 1.00E-4	> 1.00E-4
OVCAR-4	5.328	9.515	10.809	11.067	10.571	6.071	4.171	131	137	125	18	-22	* 5.01E-6	* 2.81E-5	> 1.00E-4
OVCAR-5	6.474	14.864	15.228	15.699	15.444	14.810	11.054	104	110	107	99	55	> 1.00E-4	> 1.00E-4	> 1.00E-4
OVCAR-8	0.673	3.741	4.212	4.058	3.816	0.941	0.687	115	110	102	9	-2	* 3.63E-6	* 7.09E-5	> 1.00E-4
NCI/ADR-RES	3.746	11.148	12.494	12.638	12.579	5.924	4.517	118	120	119	29	10	* 5.90E-6	> 1.00E-4	> 1.00E-4
SK-OV-3	2.611	9.944	9.467	10.790	10.710	7.391	3.954	94	112	111	65	18	* 2.12E-5	> 1.00E-4	> 1.00E-4
**Renal Cancer**															
786-0	1.778	6.542	6.526	6.590	6.504	3.806	2.438	100	101	99	42	14	* 7.39E-6	> 1.00E-4	> 1.00E-4
A498	1.927	7.664	7.997	8.363	7.879	1.566	2.485	106	112	104	-19	10	* 2.75E-6	.	> 1.00E-4
ACHN	1.537	.	.	.	.	.	.	.	.	.	.	.	.	.	.
CAKI-1	1.045	4.222	4.486	4.479	4.398	2.115	0.031	108	108	106	34	-97	* 5.93E-6	* 1.81E-5	* 4.37E-5
RXF 393	1.802	3.132	3.086	3.232	3.203	0.574	0.632	96	107	105	-68	-65	* 2.09E-6	* 4.05E-6	* 7.86E-6
SN12C	0.623	2.414	2.531	2.633	2.559	1.292	1.093	106	112	108	37	26	* 6.62E-6	> 1.00E-4	> 1.00E-4
TK-10	6.290	17.260	17.690	18.138	17.852	13.699	7.650	104	108	105	68	12	* 2.08E-5	> 1.00E-4	> 1.00E-4
UO-31	1.550	5.913	5.918	5.826	5.880	4.779	3.099	100	98	99	74	36	* 4.20E-5	> 1.00E-4	> 1.00E-4
**Prostate Cancer**															
PC-3	3.978	11.159	13.142	12.583	13.390	6.230	4.209	128	120	131	31	3	* 6.50E-6	> 1.00E-4	> 1.00E-4
DU-145	0.636	3.766	4.200	4.060	4.023	1.565	1.374	114	109	108	30	24	* 5.51E-6	> 1.00E-4	> 1.00E-4
**Breast Cancer**															
MCF7	2.550	16.169	13.679	13.856	15.192	11.394	5.812	82	83	93	65	24	* 2.31E-5	> 1.00E-4	> 1.00E-4
MDA-MB-231/ATCC	5.261	12.044	13.172	12.393	12.573	11.335	7.443	117	105	108	90	32	* 4.89E-5	> 1.00E-4	> 1.00E-4
HS 578T	1.006	2.636	2.643	3.100	2.836	1.978	0.956	102	129	114	61	-6	* 1.48E-5	* 8.20E-5	> 1.00E-4
BT-549	3.822	8.395	9.521	9.838	9.485	6.761	1.893	125	132	124	64	-50	* 1.33E-5	* 3.63E-5	* 9.91E-5
T-47D	5.838	11.611	11.242	11.667	11.328	8.986	5.709	94	101	95	54	-2	* 1.20E-5	* 9.14E-5	> 1.00E-4
MDA-MB-468	6.310	10.765	10.853	11.073	11.005	7.199	4.198	102	107	105	20	-34	* 4.44E-6	* 2.35E-5	> 1.00E-4

NCI five dose results of compound **11c**

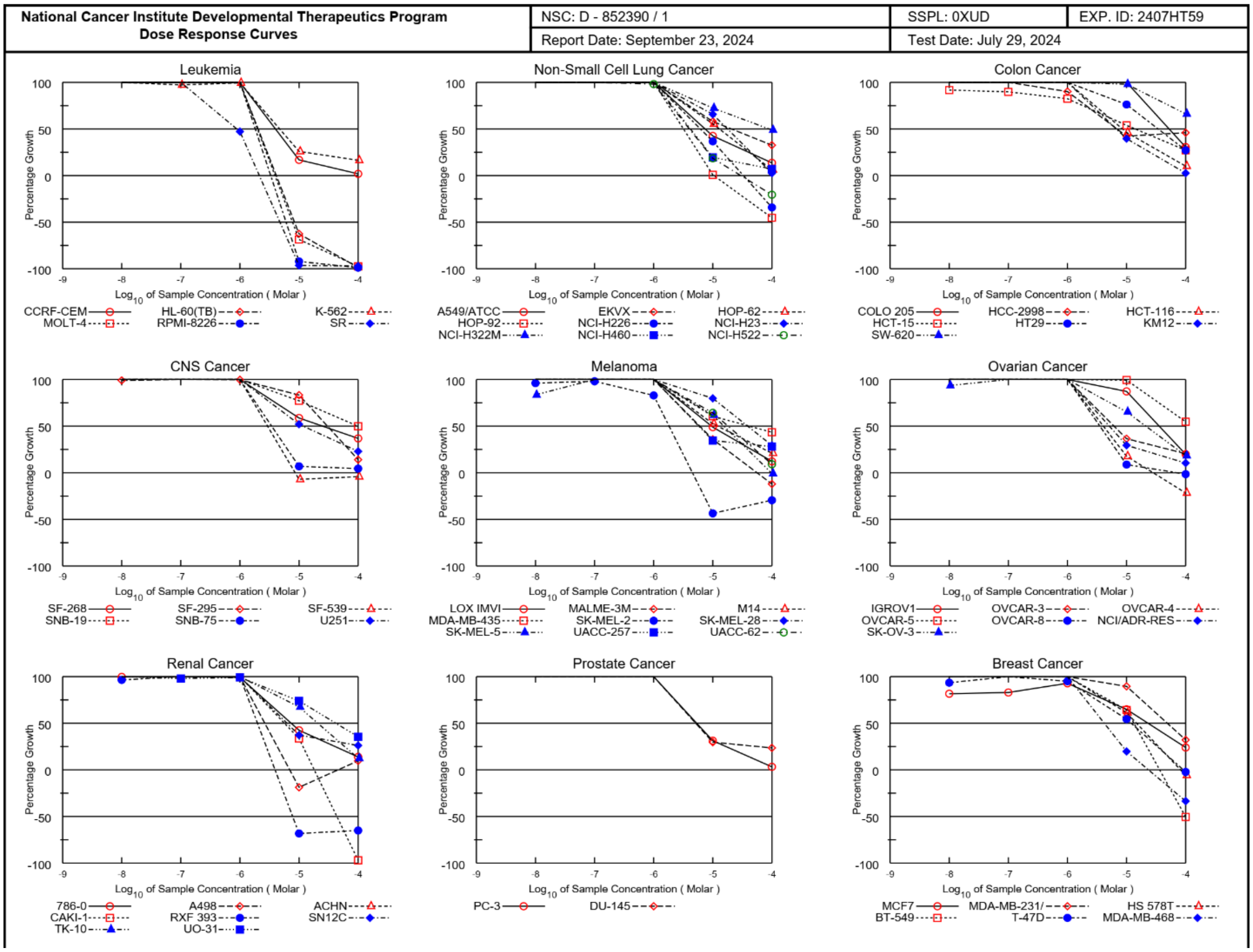

NCI five dose results of compound **11c**

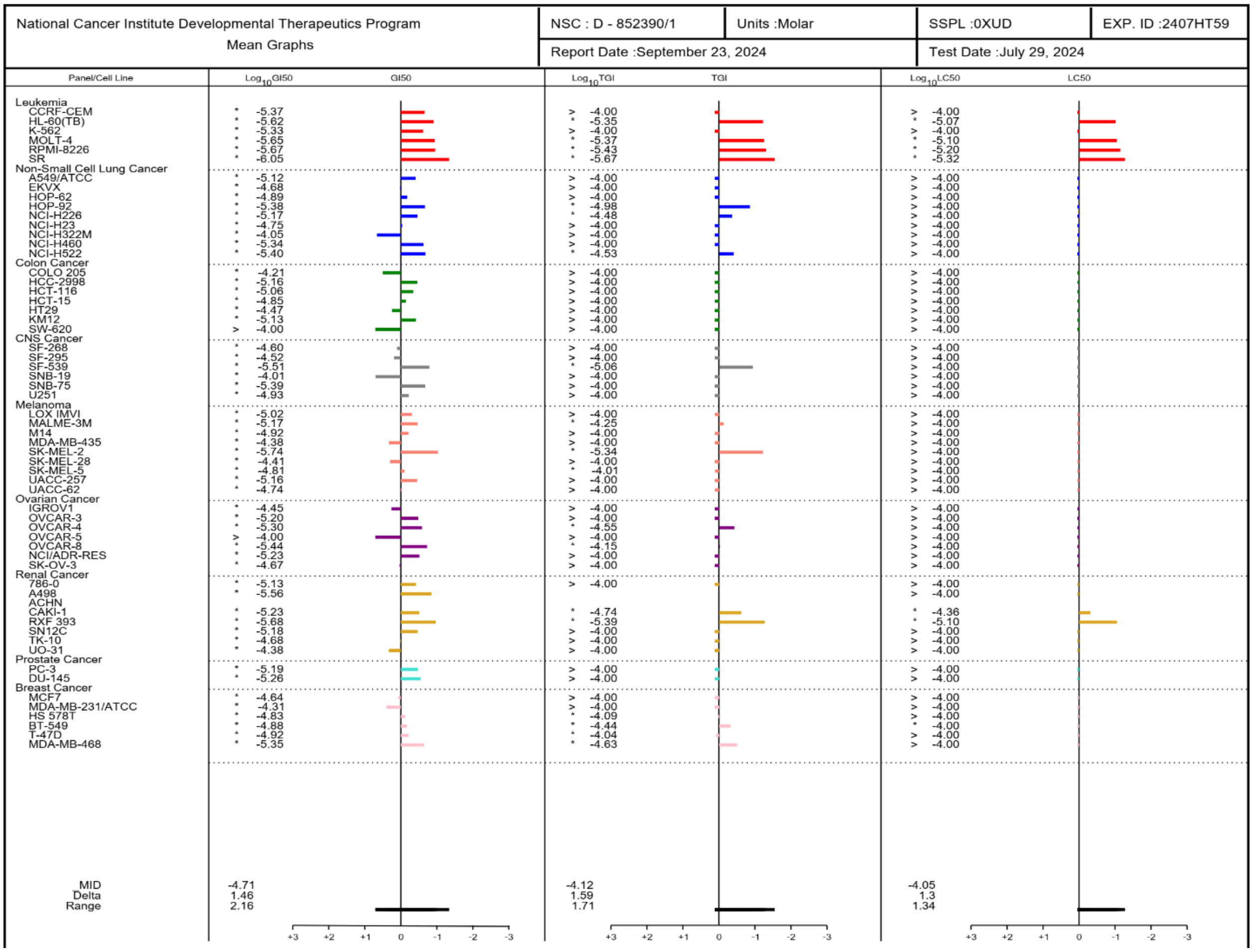

NCI five dose results of compound **11c**

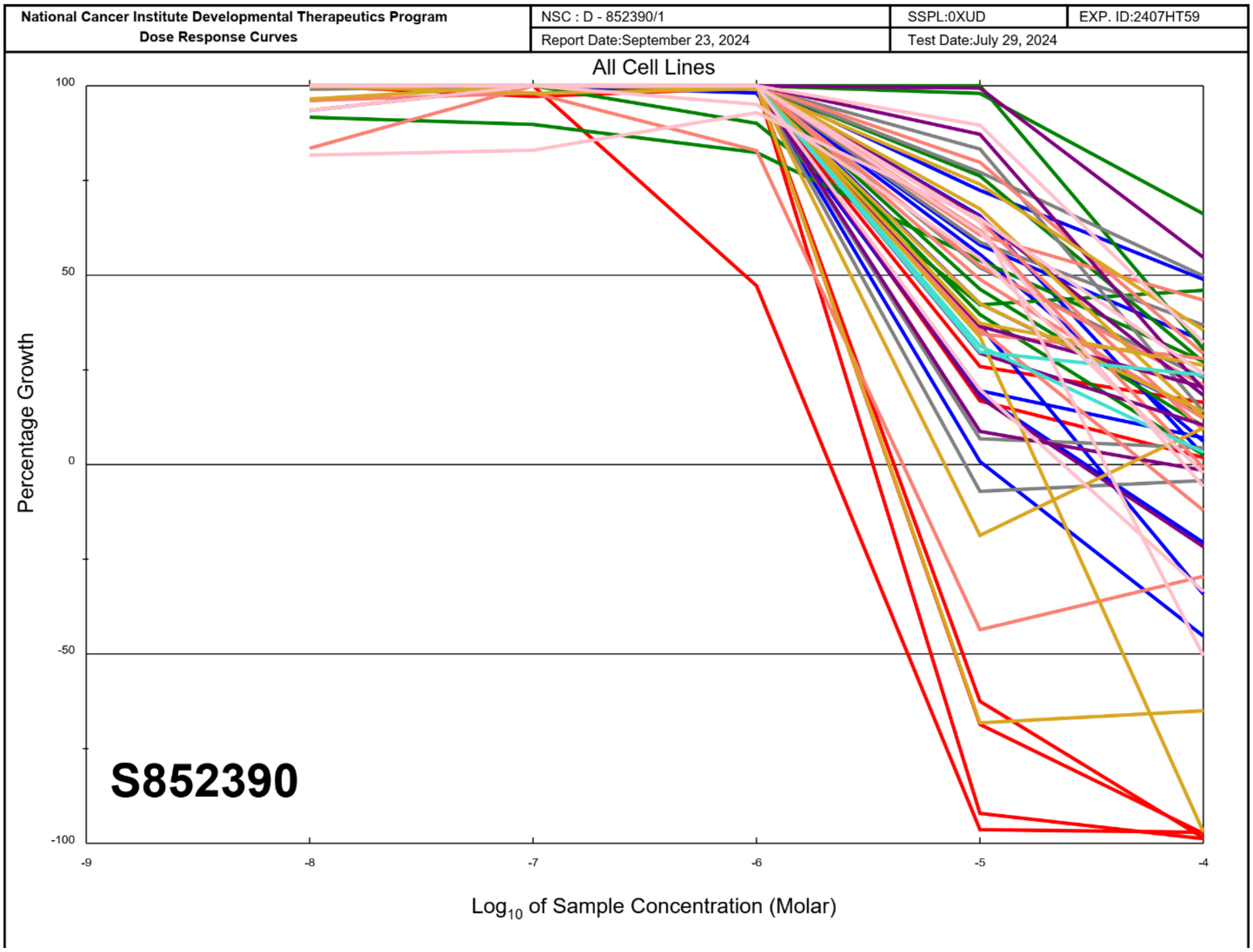

NCI five dose results of compound **11c**

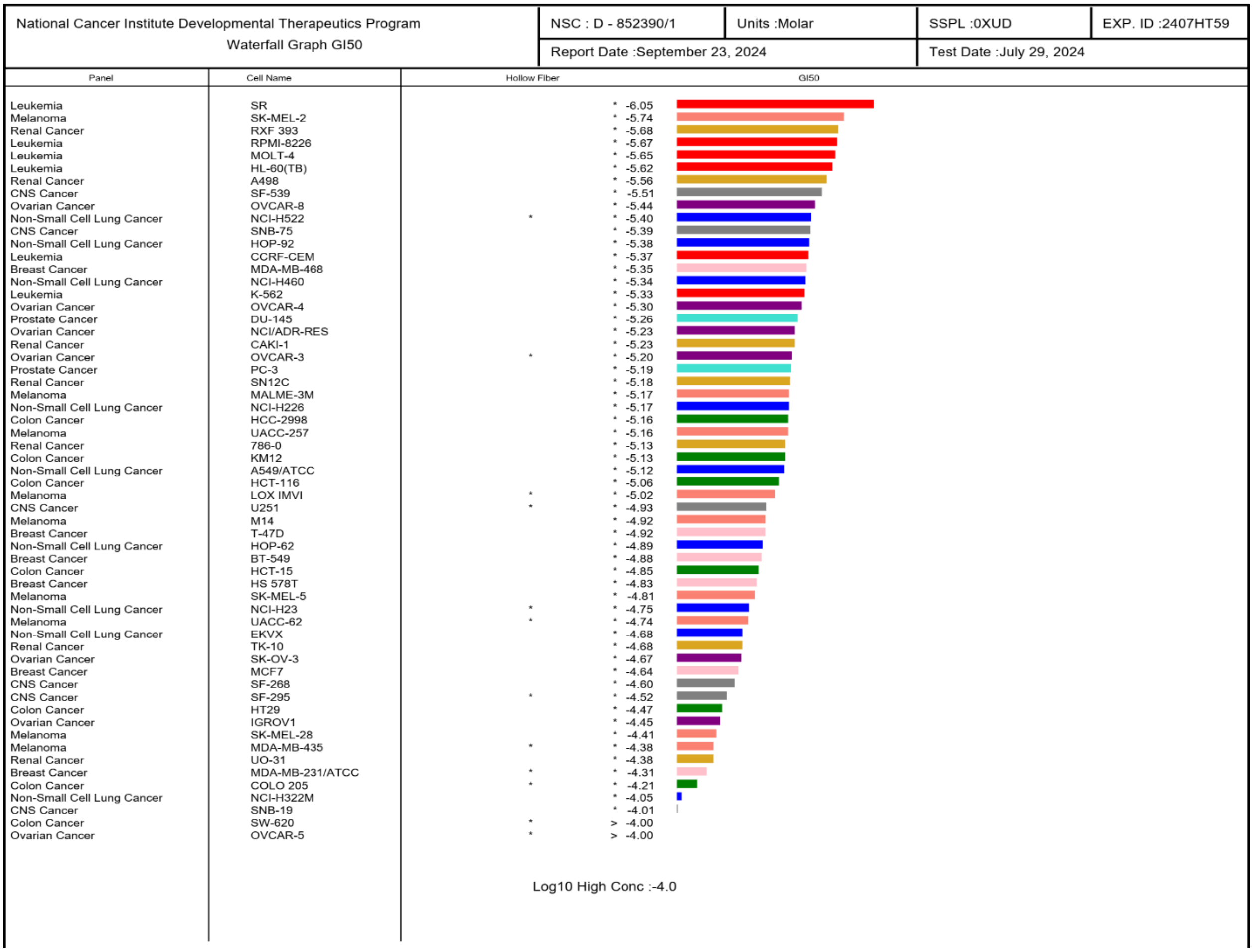

NCI five dose results of compound **11c**

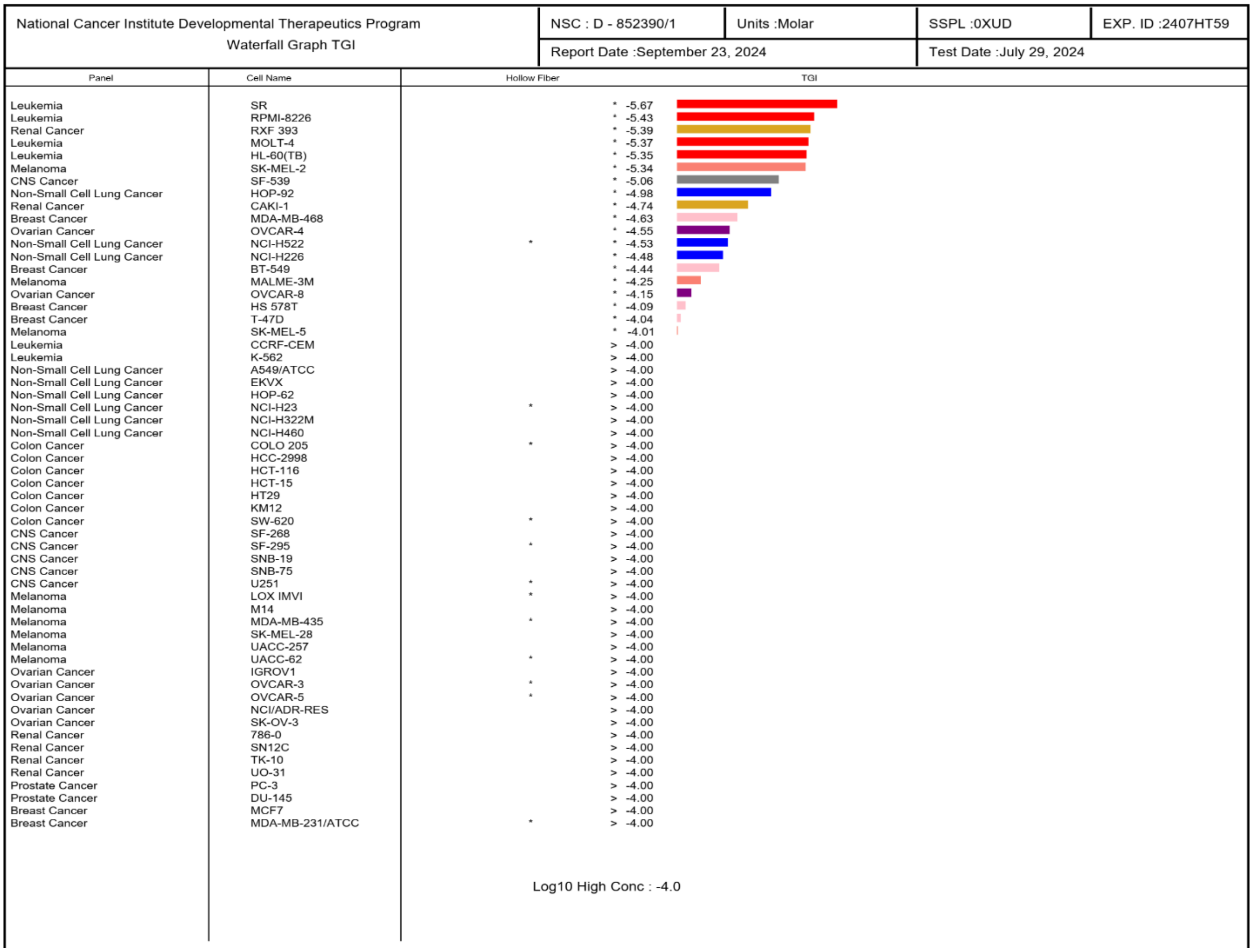

NCI five dose results of compound **11c**

National Cancer Institute Developmental Therapeutics Program<br>Waterfall Graph LC50	NSC : D - 852390/1	Units :Molar	SSPL :0XUD	EXP. ID :2407HT59
	Report Date :September 23, 2024		Test Date :July 29, 2024	

Panel	Cell Name	Hollow Fiber	LC50
Leukemia	SR		* -5.32
Leukemia	RPMI-8226		* -5.20
Renal Cancer	RXF 393		* -5.10
Leukemia	MOLT-4		* -5.10
Leukemia	HL-60(TB)		* -5.07
Renal Cancer	CAKI-1		* -4.36
Breast Cancer	BT-549		* -4.00
Leukemia	CCRF-CEM		> -4.00
Leukemia	K-562		> -4.00
Non-Small Cell Lung Cancer	A549/ATCC		> -4.00
Non-Small Cell Lung Cancer	EKVX		> -4.00
Non-Small Cell Lung Cancer	HOP-62		> -4.00
Non-Small Cell Lung Cancer	HOP-92		> -4.00
Non-Small Cell Lung Cancer	NCI-H226		> -4.00
Non-Small Cell Lung Cancer	NCI-H23	*	> -4.00
Non-Small Cell Lung Cancer	NCI-H322M		> -4.00
Non-Small Cell Lung Cancer	NCI-H460		> -4.00
Non-Small Cell Lung Cancer	NCI-H522	*	> -4.00
Colon Cancer	COLO 205	*	> -4.00
Colon Cancer	HCC-2998		> -4.00
Colon Cancer	HCT-116		> -4.00
Colon Cancer	HCT-15		> -4.00
Colon Cancer	HT29		> -4.00
Colon Cancer	KM12		> -4.00
Colon Cancer	SW-620	*	> -4.00
CNS Cancer	SF-268		> -4.00
CNS Cancer	SF-295	*	> -4.00
CNS Cancer	SF-539		> -4.00
CNS Cancer	SNB-19		> -4.00
CNS Cancer	SNB-75		> -4.00
CNS Cancer	U251	*	> -4.00
Melanoma	LOX IMVI	*	> -4.00
Melanoma	MALME-3M		> -4.00
Melanoma	M14		> -4.00
Melanoma	MDA-MB-435	*	> -4.00
Melanoma	SK-MEL-2		> -4.00
Melanoma	SK-MEL-28		> -4.00
Melanoma	SK-MEL-5		> -4.00
Melanoma	UACC-257		> -4.00
Melanoma	UACC-62	*	> -4.00
Ovarian Cancer	IGROV1		> -4.00
Ovarian Cancer	OVCAR-3	*	> -4.00
Ovarian Cancer	OVCAR-4		> -4.00
Ovarian Cancer	OVCAR-5	*	> -4.00
Ovarian Cancer	OVCAR-8		> -4.00
Ovarian Cancer	NCI/ADR-RES		> -4.00
Ovarian Cancer	SK-OV-3		> -4.00
Renal Cancer	786-0		> -4.00
Renal Cancer	A498		> -4.00
Renal Cancer	SN12C		> -4.00
Renal Cancer	TK-10		> -4.00
Renal Cancer	UO-31		> -4.00
Prostate Cancer	PC-3		> -4.00
Prostate Cancer	DU-145		> -4.00
Breast Cancer	MCF7		> -4.00
Breast Cancer	MDA-MB-231/ATCC	*	> -4.00
Breast Cancer	HS 578T		> -4.00
Breast Cancer	T-47D		> -4.00
Breast Cancer	MDA-MB-468		> -4.00

Log10 High Conc : -4.0

NCI five dose results of compound **11c**

in-vitro cytotoxic effects against normal cells WI-38

Detailed results:

researcher	assay	Date	cells
Dr.Omar alshazly	MTT	25-Sep	WI38

	Blank	CC	Sample No.	11b/WI38				Sample No.	Gef/WI38			
	1	2	3	4	5	6	7	8	9	10	11	12
A	B	C	100ug	25ug	6.25ug	1.6ug	0.4ug	100ug	25ug	6.25ug	1.6ug	0.4ug
B	B	C	100ug	25ug	6.25ug	1.6ug	0.4ug	100ug	25ug	6.25ug	1.6ug	0.4ug
C	B	C	100ug	25ug	6.25ug	1.6ug	0.4ug	100ug	25ug	6.25ug	1.6ug	0.4ug

ROBONIK P2000 eia reader

Wave length: 450 nm

Reference: 630 nm

	1	2	3	4	5	6	7	8	9	10	11	12
A	0.001	0.479	0.226	0.286	0.325	0.367	0.439	0.161	0.238	0.292	0.372	0.418
B	0.001	0.513	0.241	0.292	0.346	0.392	0.451	0.174	0.244	0.307	0.363	0.425
C	0.001	0.492	0.239	0.303	0.357	0.411	0.461	0.165	0.229	0.318	0.359	0.419
mean	0.00038	0.4947	0.2353	0.29367	0.3427	0.39	0.4503	0.1667	0.237	0.3057	0.3647	0.421
%		`	47.574	59.3666	69.272	78.841	91.038	33.693	47.911	61.792	73.72	85.04

11b/WI38

log conc.	% viability
2	47.5741
1.3979	59.3666
0.7959	69.2722
0.1931	78.841
-0.409	91.0377

IC50=

[redacted] [redacted] [redacted]

Gef/WI38

log conc.	% viability
2	33.693
1.3979	47.911
0.7959	61.792
0.1931	73.72
-0.409	85.04

IC50=

[redacted] [redacted]

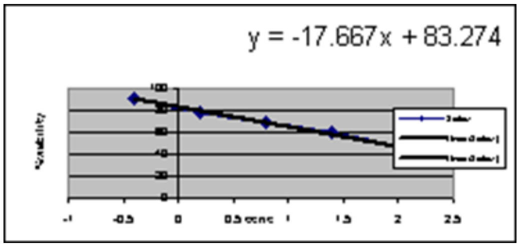

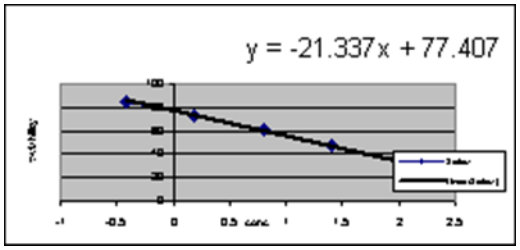

Enzymatic Assay

Detailed results

VEGFR-2

code	Conc µM	log	%inhibition	T2	T1	ΔT
**10b**	10	1	84.53	30	0	30
	1	0	65.77	30	0	30
	0.1	-1	49.68	30	0	30
	0.01	-2	28.79	30	0	30
	0.001	-3	7.10	30	0	30

EC

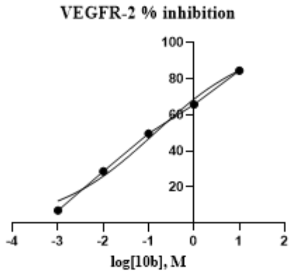

code	conc	log	%inhibition	T2	T1	ΔT
**11a**	10	1	81.98	30	0	30
	1	0	65.61	30	0	30
	0.1	-1	45.83	30	0	30
	0.01	-2	32.56	30	0	30
	0.001	-3	9.71	30	0	30

EC

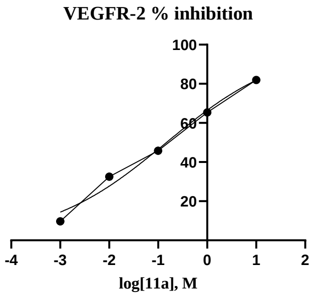

code	conc	log	%inhibition	T2	T1	ΔT
**11b**	10	1	89.08	30	0	30
	1	0	67.82	30	0	30
	0.1	-1	45.51	30	0	30
	0.01	-2	30.86	30	0	30
	0.001	-3	14.66	30	0	30

EC

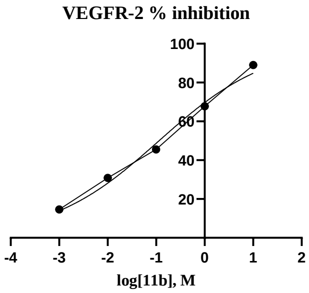

code	conc	log	%inhibition	T2	T1	ΔT
**Sorafenib**	10	1	88.08	30	0	30
	1	0	64.24	30	0	30
	0.1	-1	49.51	30	0	30
	0.01	-2	33.53	30	0	30
	0.001	-3	12.47	30	0	30

EC

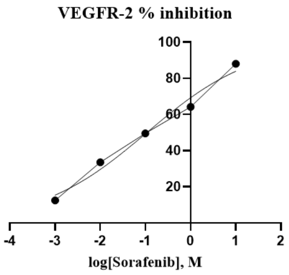

EGFR

code	Conc µM	log	%inhibition	T2	T1	ΔT
**10b**	10	1	85.32	30	0	30
	1	0	70.34	30	0	30
	0.1	-1	58.77	30	0	30
	0.01	-2	35.91	30	0	30

	0.001	-3	20.25	30	0	30

EC

code	conc	log	%inhibition	T2	T1	ΔT
**11a**	10	1	88.09	30	0	30
	1	0	75.28	30	0	30
	0.1	-1	64.19	30	0	30
	0.01	-2	36.48	30	0	30
	0.001	-3	23.14	30	0	30

EC

code	conc	log	%inhibition	T2	T1	ΔT
**11b**	10	1	86.97	30	0	30
	1	0	76.14	30	0	30
	0.1	-1	67.39	30	0	30
	0.01	-2	40.79	30	0	30
	0.001	-3	20.75	30	0	30

EC

code	conc	log	%inhibition	T2	T1	ΔT
**Gefitinib**	10	2	87.17	30	0	30
	1	1	72.46	30	0	30
	0.1	0	62.99	30	0	30
	0.01	-1	38.15	30	0	30
	0.001	-2	18.16	30	0	30

EC

IR Results

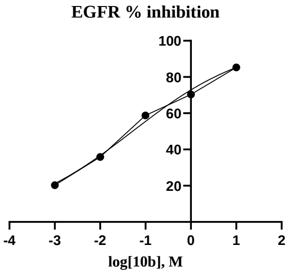

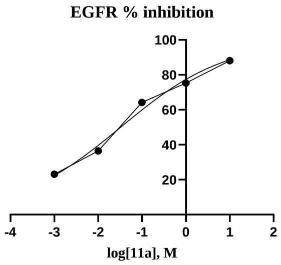

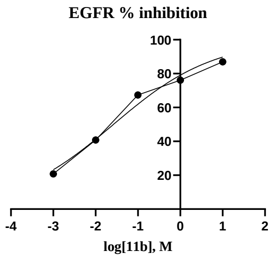

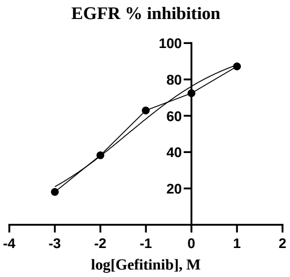

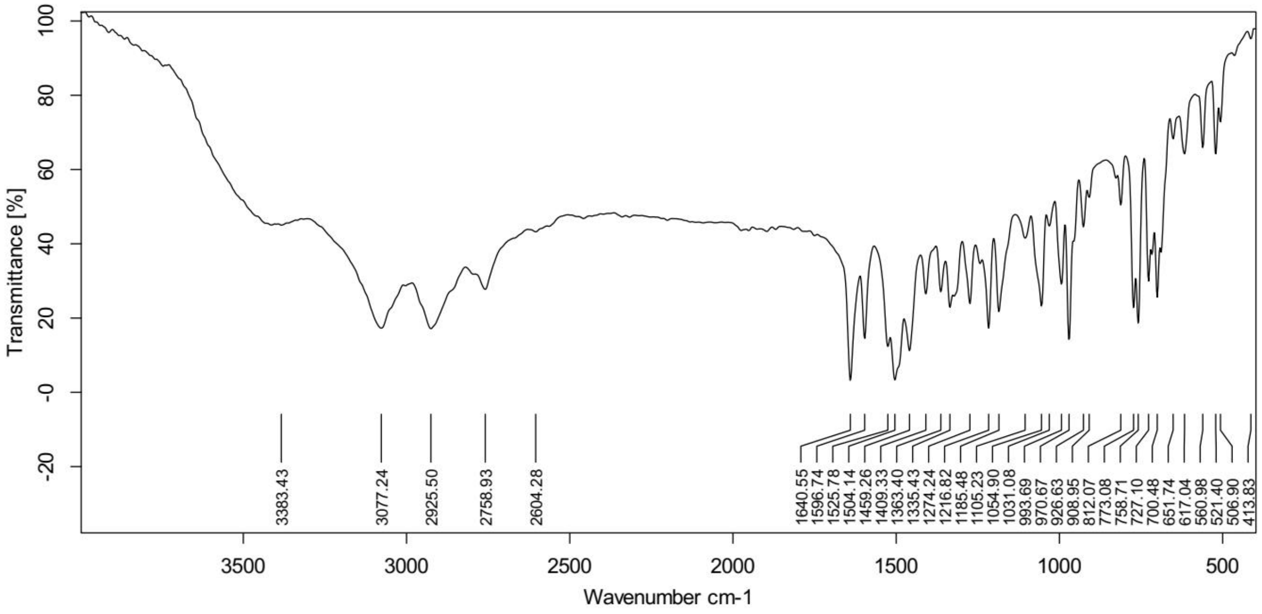

1_TRANS.46 **10:29:38 Õ** **14/05/2024**

IR results of compound 9a

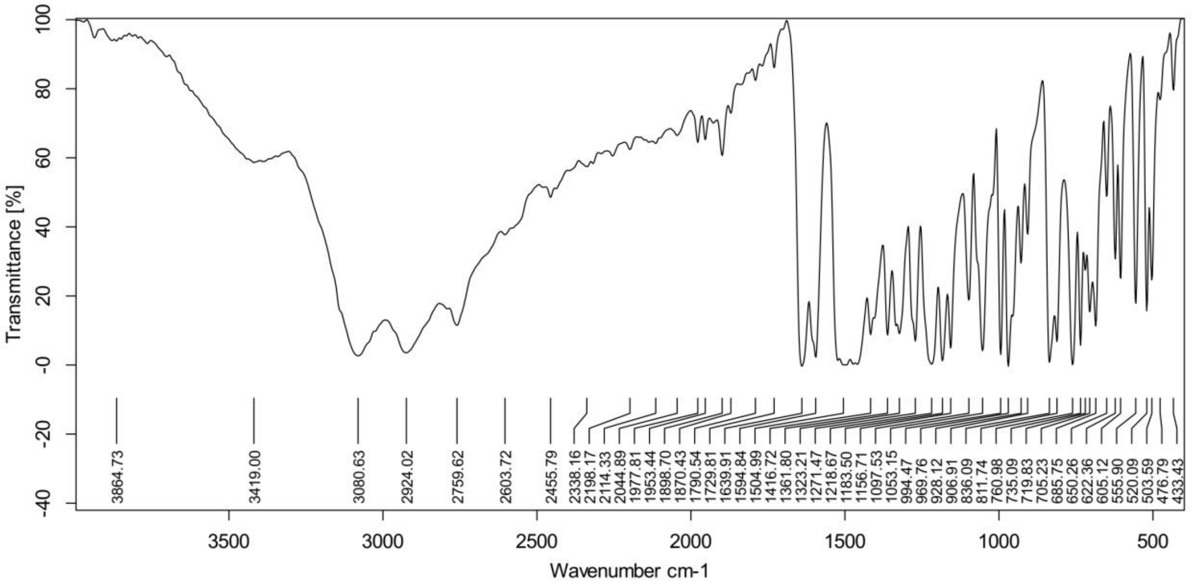

2_TRANS.42 **10:32:34 Õ** **14/05/2024**

IR results of compound 9b

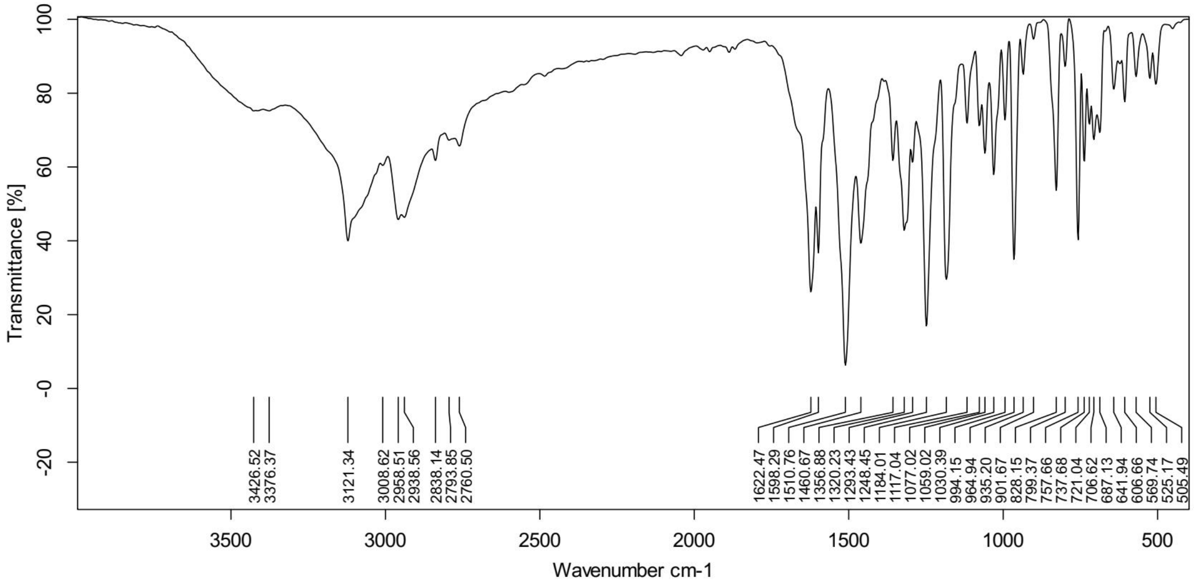

3_TRANS.30 **10:37:03 Õ** **14/05/2024**

IR results of compound 9c

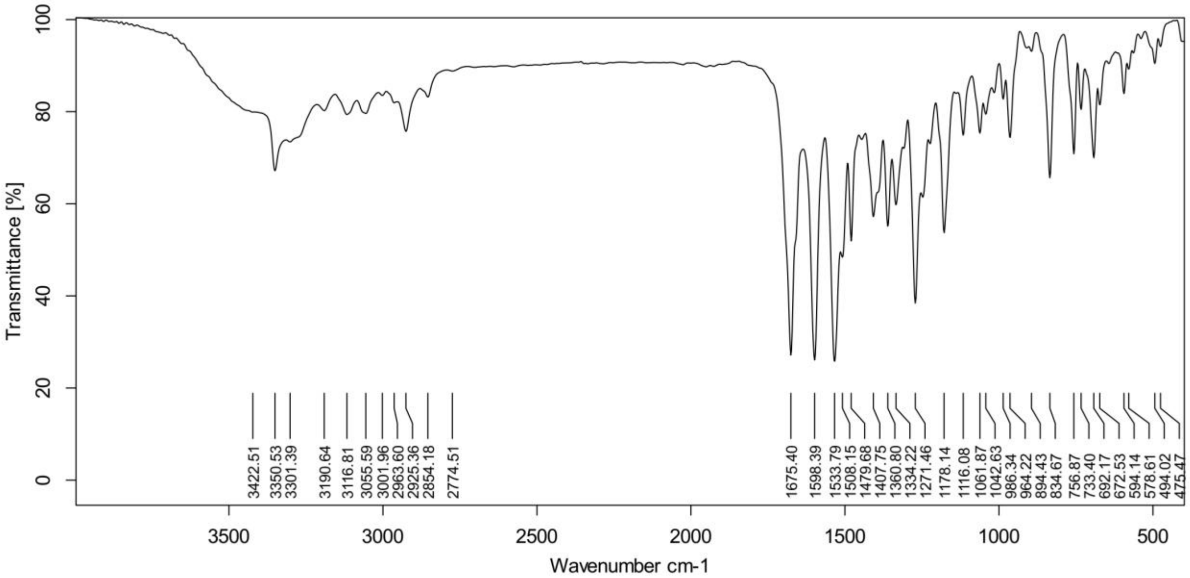

31_TRANS.1 01:44:16 ã 14/05/2024

IR results of compound 10a

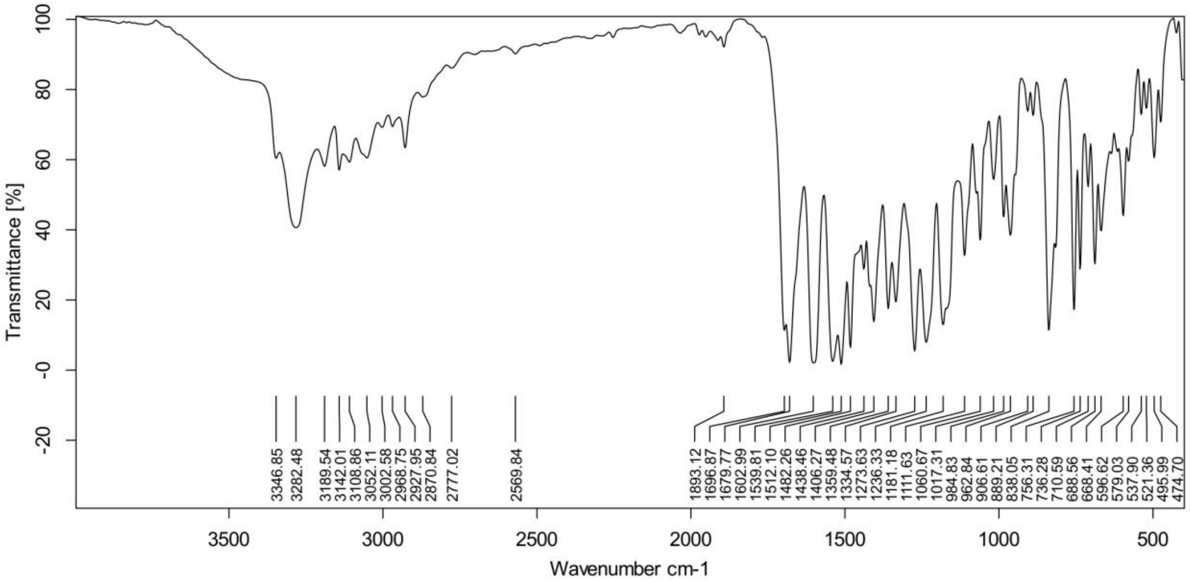

32_TRANS.1 **01:47:20 ã** **14/05/2024**

IR results of compound 10b

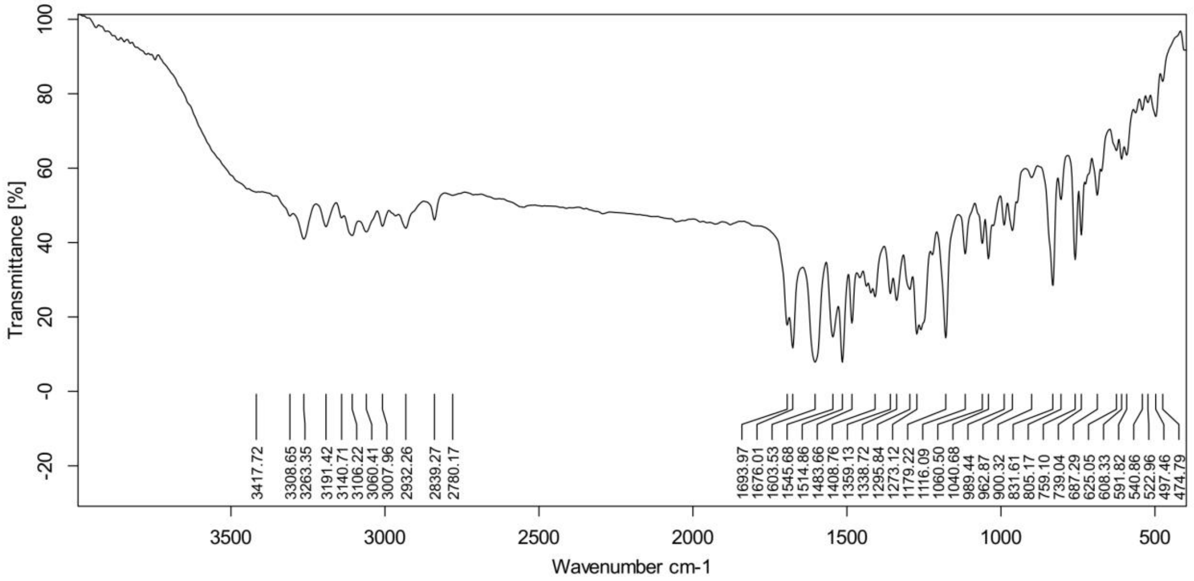

33_TRANS.1 **01:53:22 ã** **14/05/2024**

IR results of compound 10c

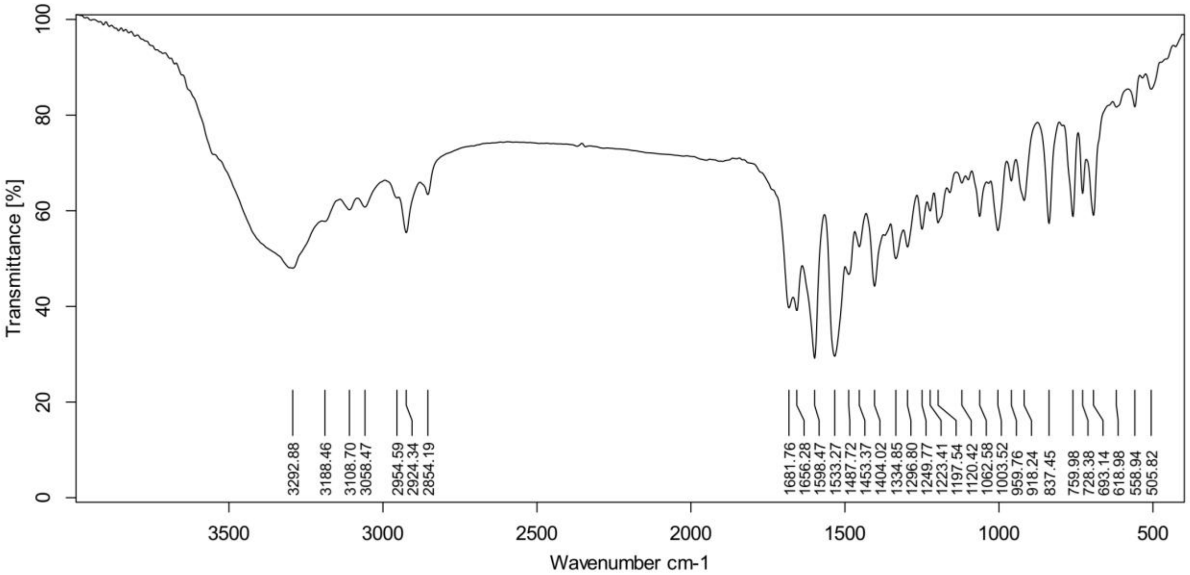

34_TRANS.1 **02:01:31 ã** **14/05/2024**

IR results of compound 11a

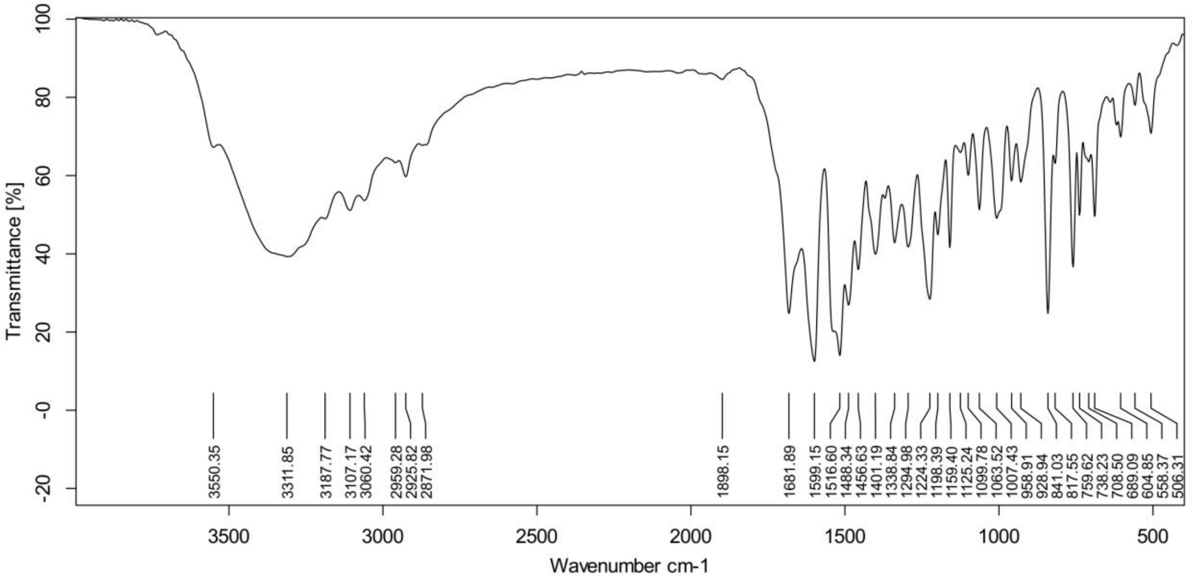

35_TRANS.1 **02:05:12 ã** **14/05/2024**

IR results of compound 11b

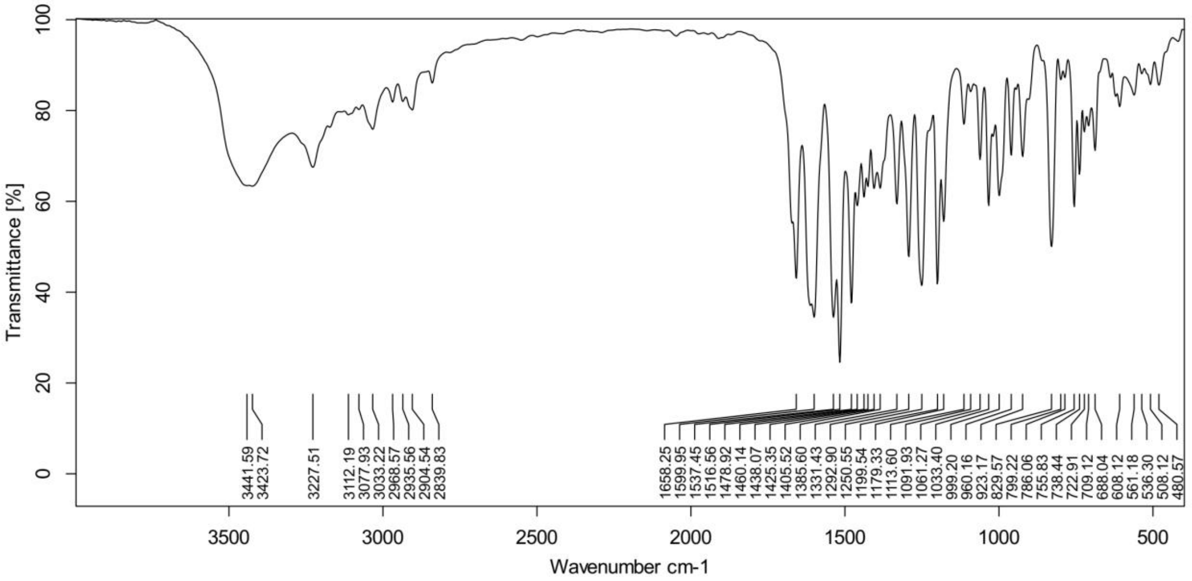

36_TRANS.1 **02:08:48 ã** **14/05/2024**

IR results of compound 11c

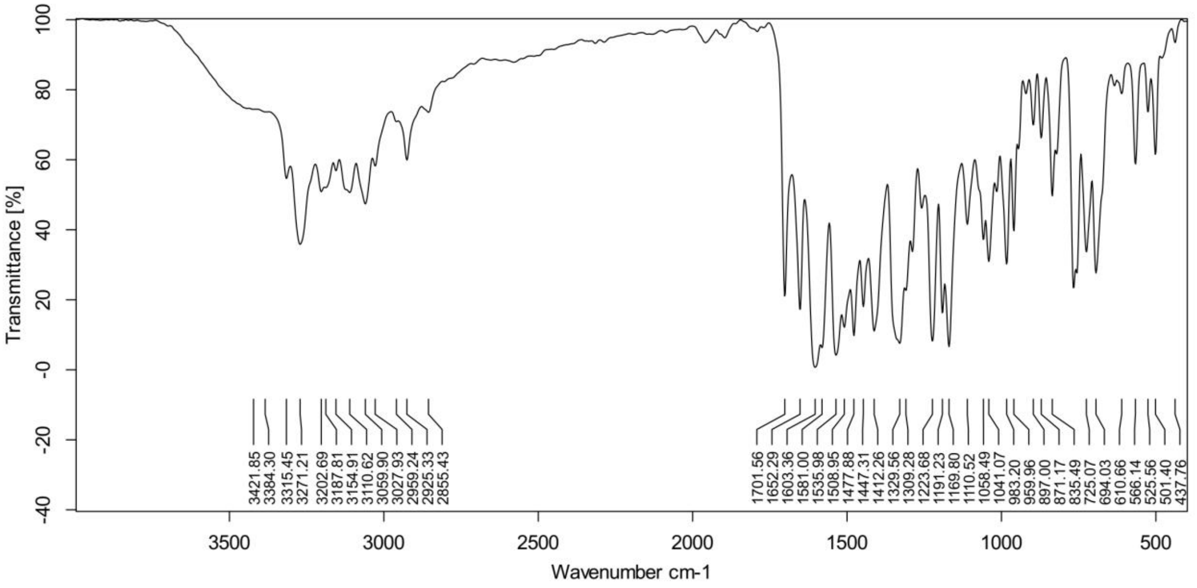

4_TRANS.26 10:42:26 Õ 14/05/2024

IR results of compound 12a

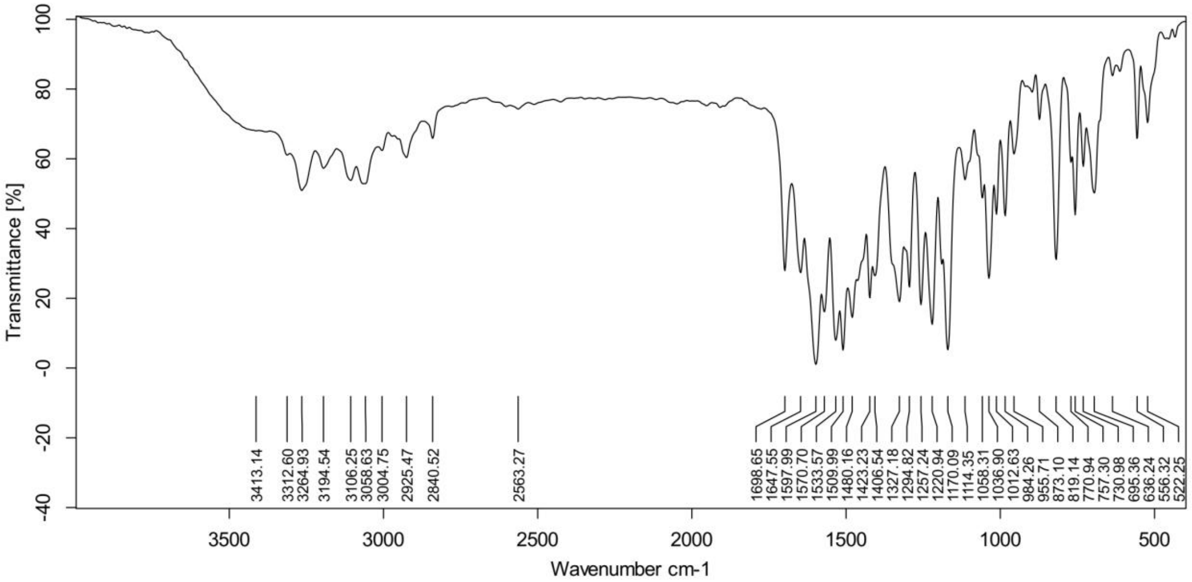

5_TRANS.15 10:48:57 Õ 14/05/2024

IR results of compound 12b

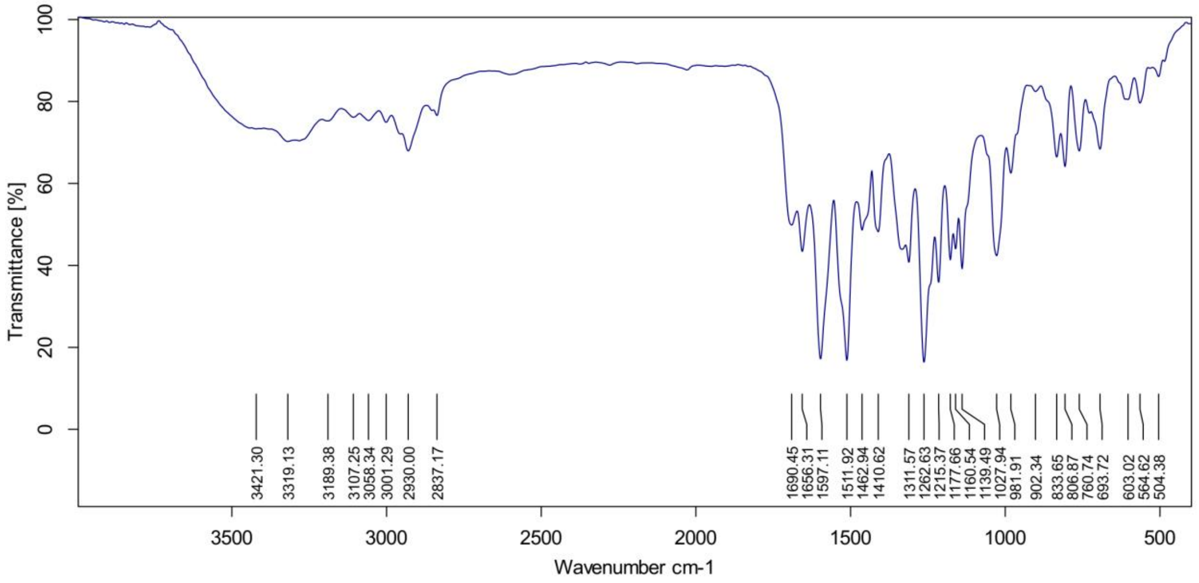

6_TRANS.14 **10:54:32 Õ** **14/05/2024**

IR results of compound 12c

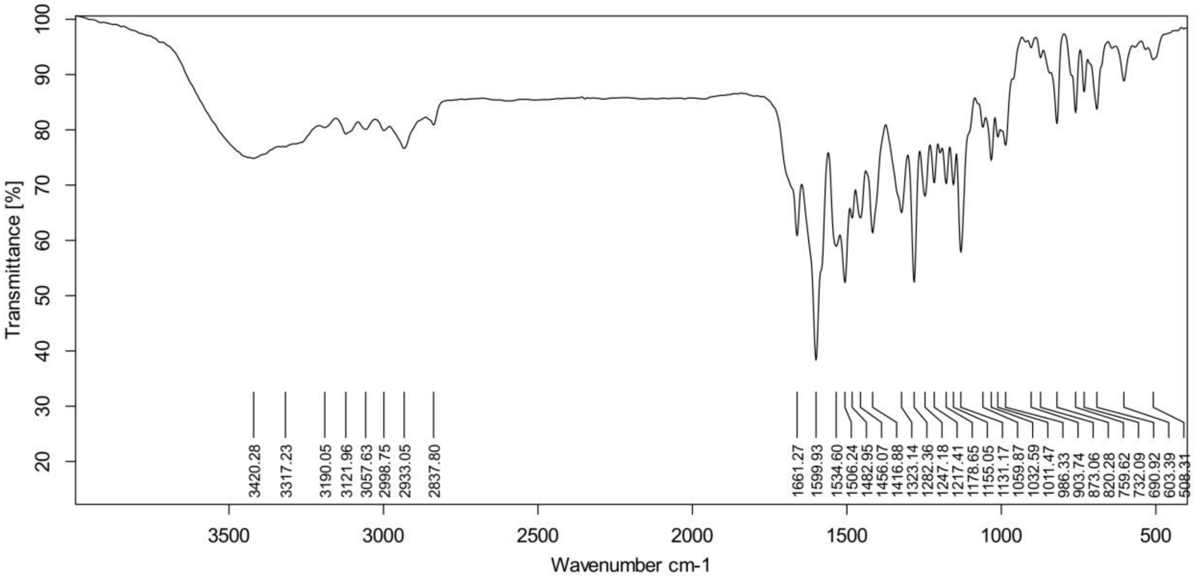

7_TRANS.12 **10:56:13 Õ** **14/05/2024**

IR results of compound 12d

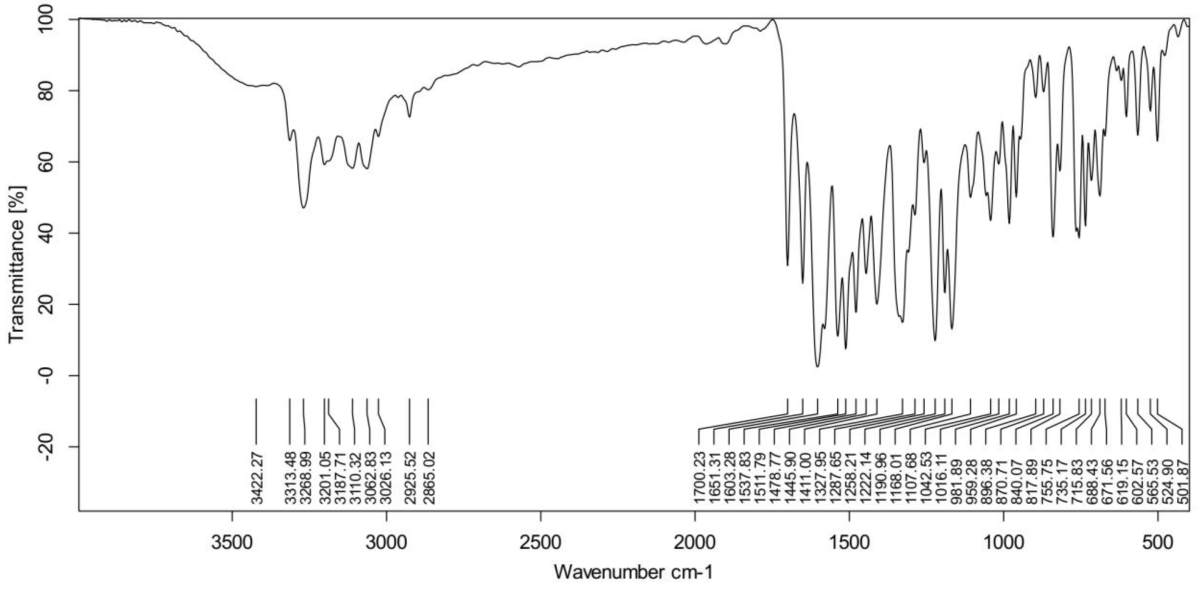

8_TRANS.6 11:02:24 Õ 14/05/2024

IR results of compound 12e

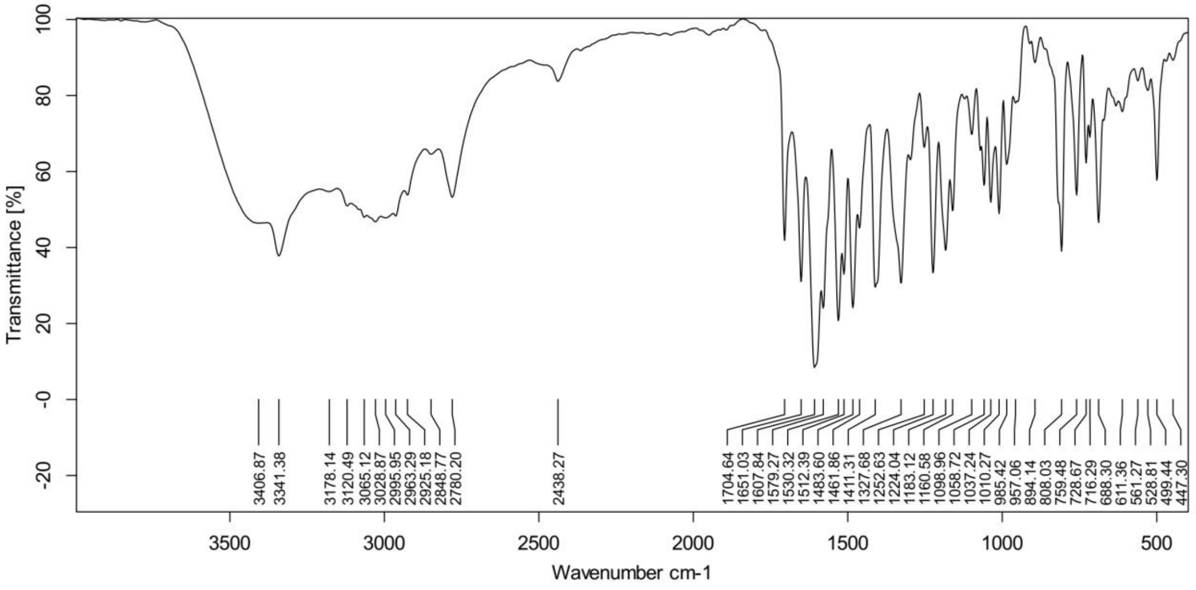

9_TRANS.6 **11:11:37 Õ** **14/05/2024**

IR results of compound 12f

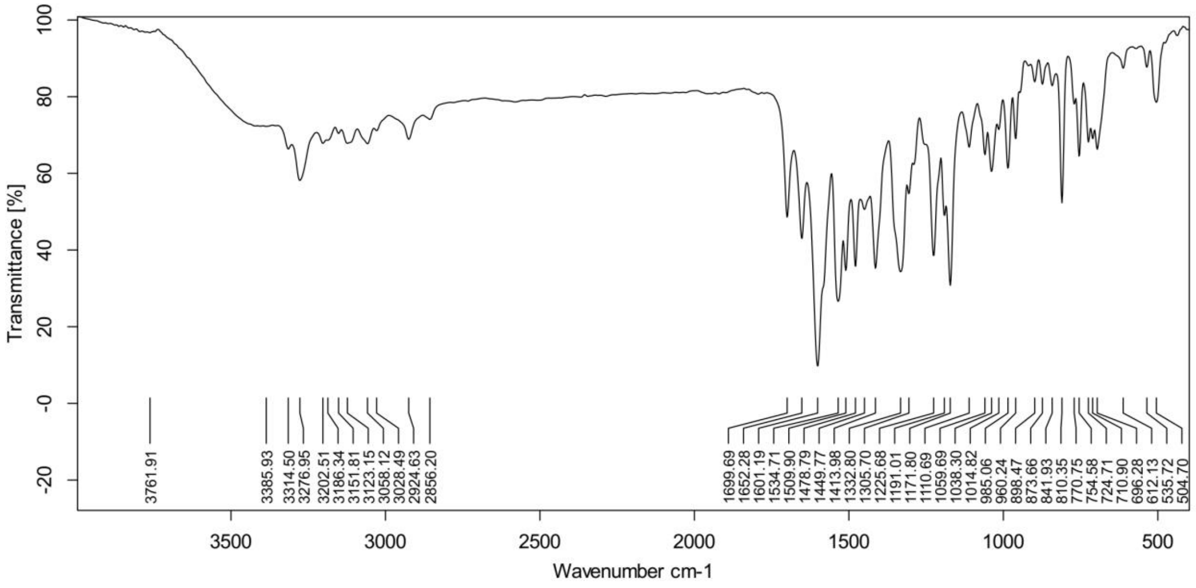

10_TRANS.7 **11:20:55 Õ** **14/05/2024**

IR results of compound 12g

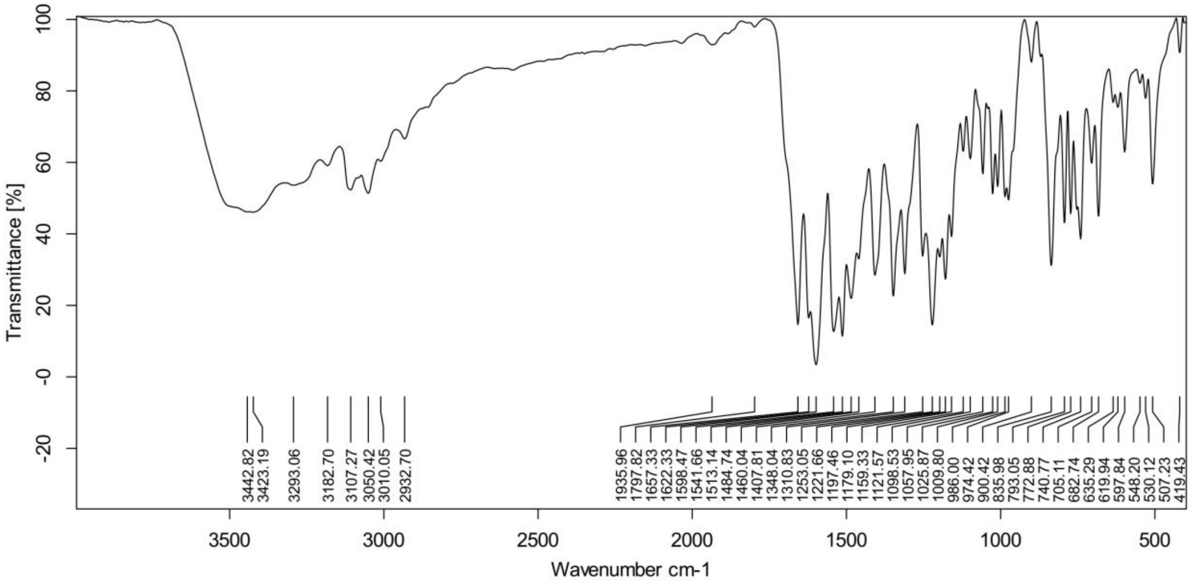

11_TRANS.8 11:29:52 Õ 14/05/2024

IR results of compound 12h

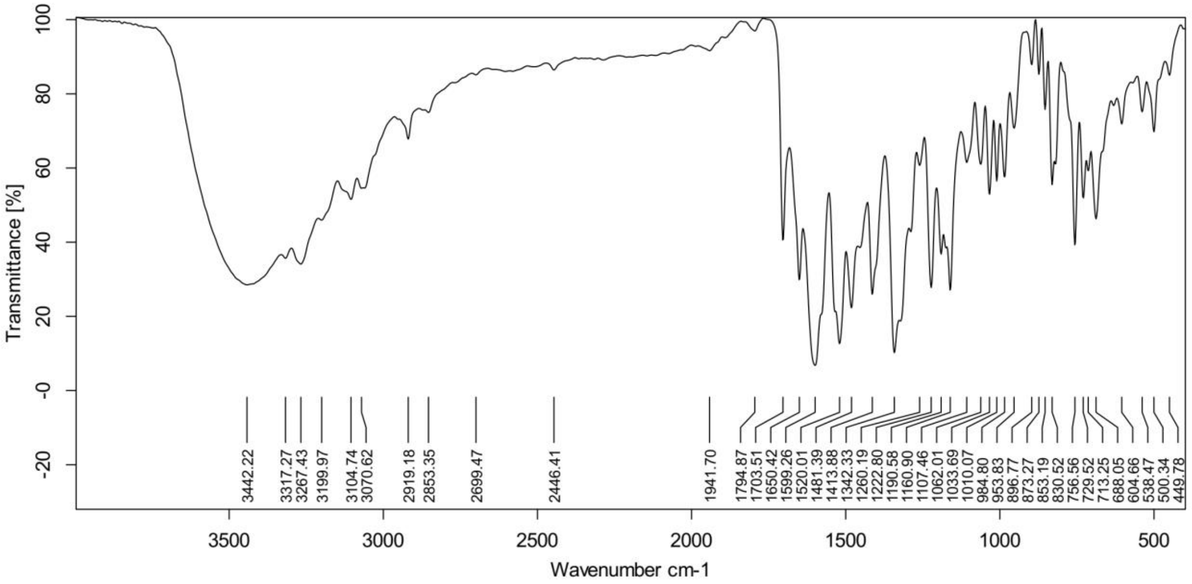

12_TRANS.7 **11:31:37 Õ** **14/05/2024**

IR results of compound 12i

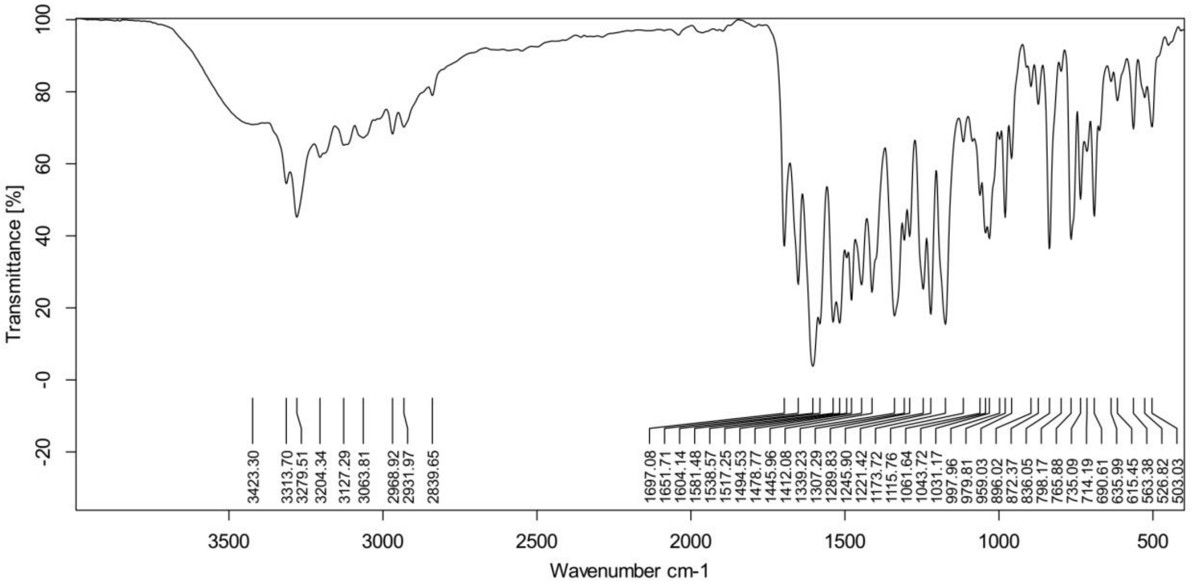

13_TRANS.2 **11:37:29 Õ** **14/05/2024**

IR results of compound 13a

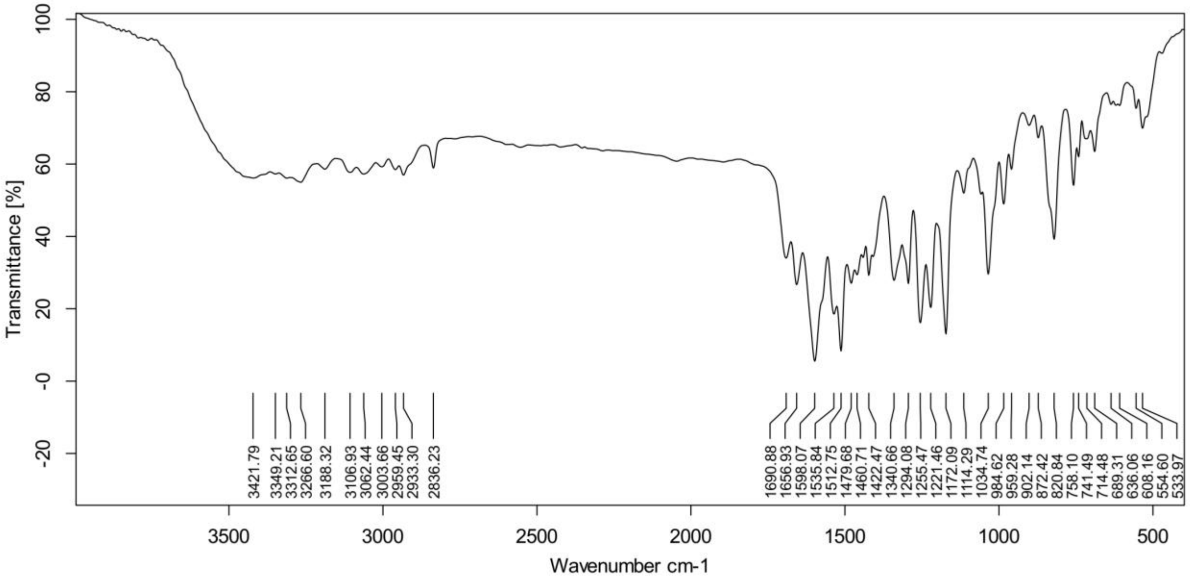

14_TRANS.3 11:58:34 Õ 14/05/2024

IR results of compound 13b

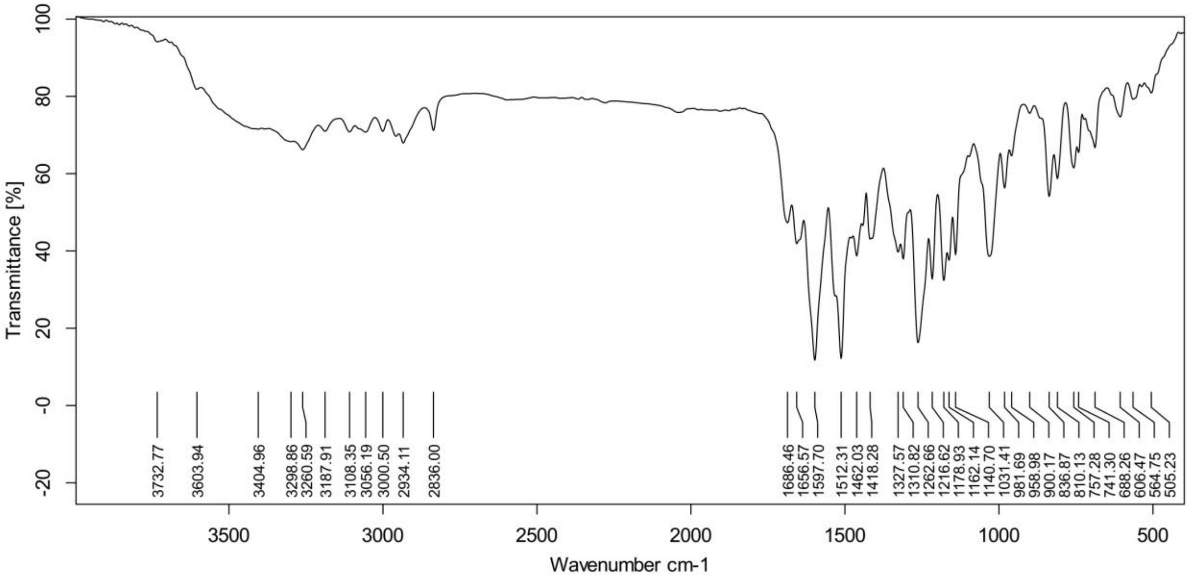

15_TRANS.4 12:10:53 ã 14/05/2024

IR results of compound 13c

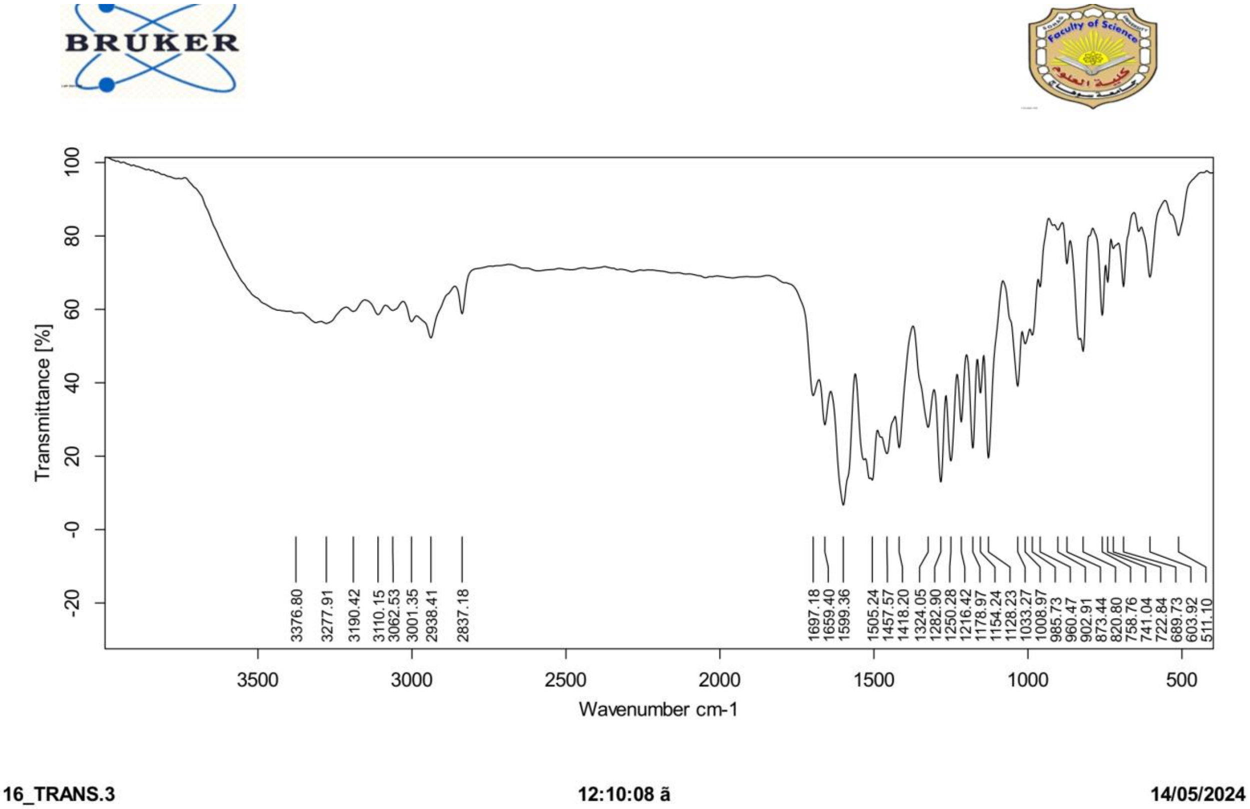

IR results of compound 13d

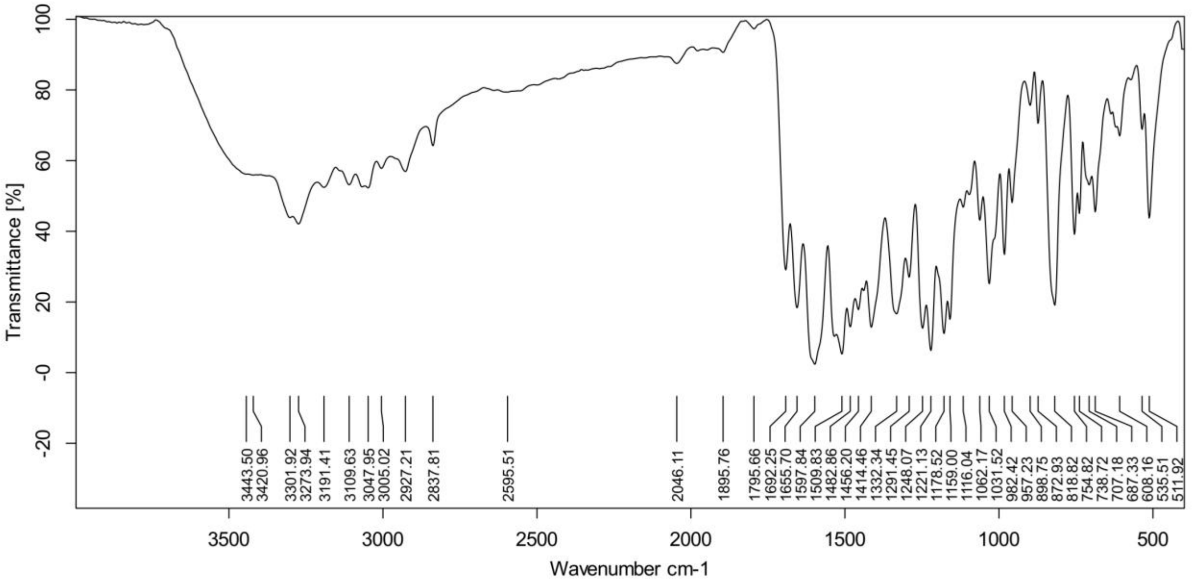

17_TRANS.2 **12:17:08 ã** **14/05/2024**

IR results of compound 13e

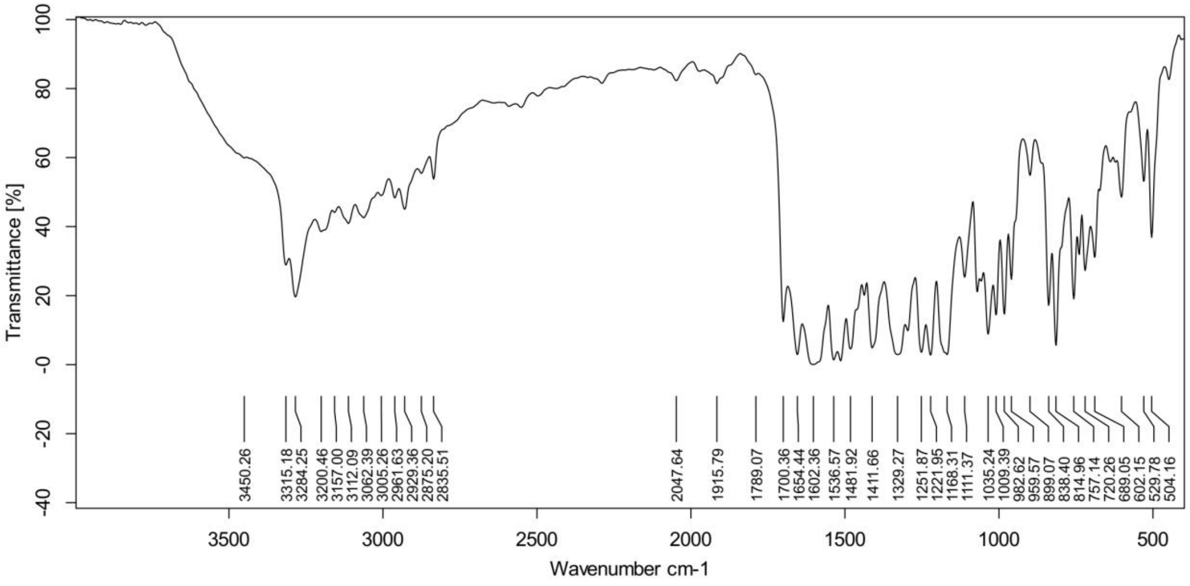

18_TRANS.3 12:22:48 ã 14/05/2024

IR results of compound 13f

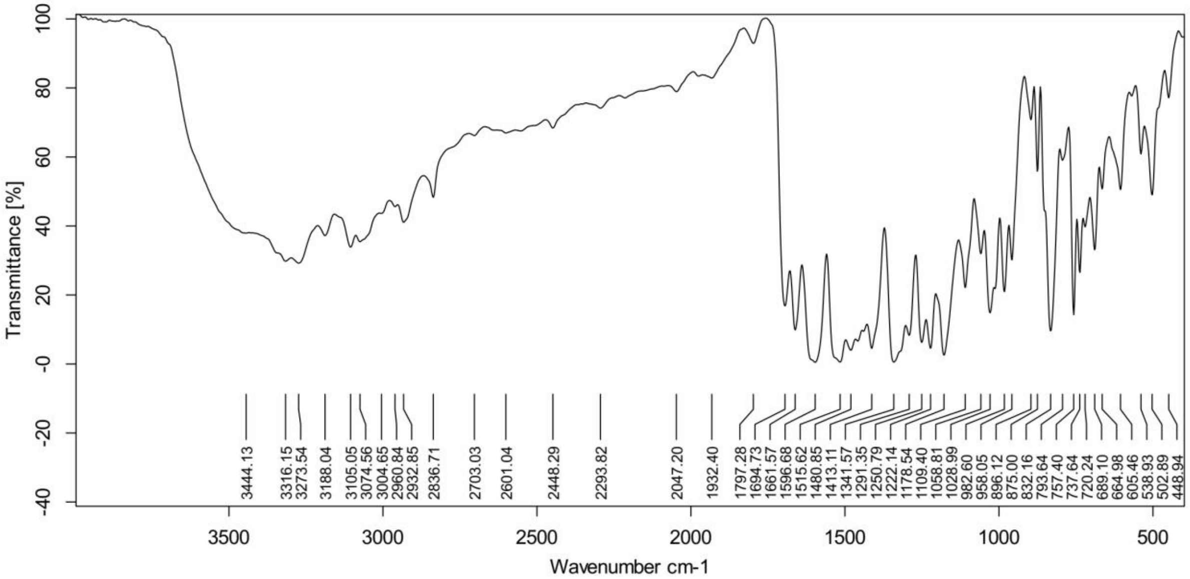

19_TRANS.1 12:28:21 ã 14/05/2024

IR results of compound 13g

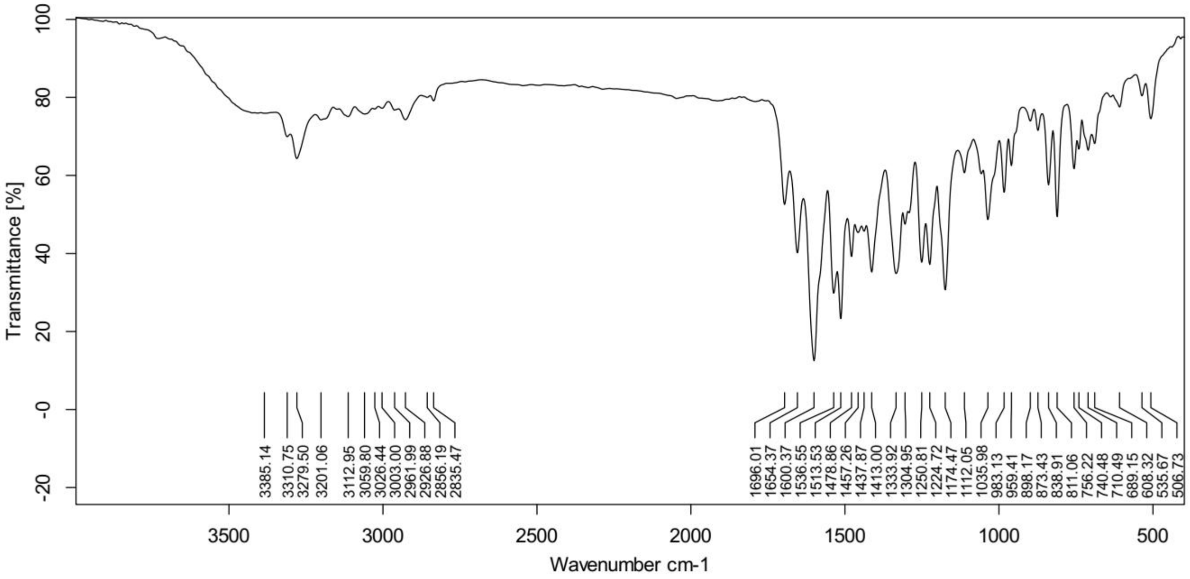

20_TRANS.2 **12:35:43 ã** **14/05/2024**

IR results of compound 13h

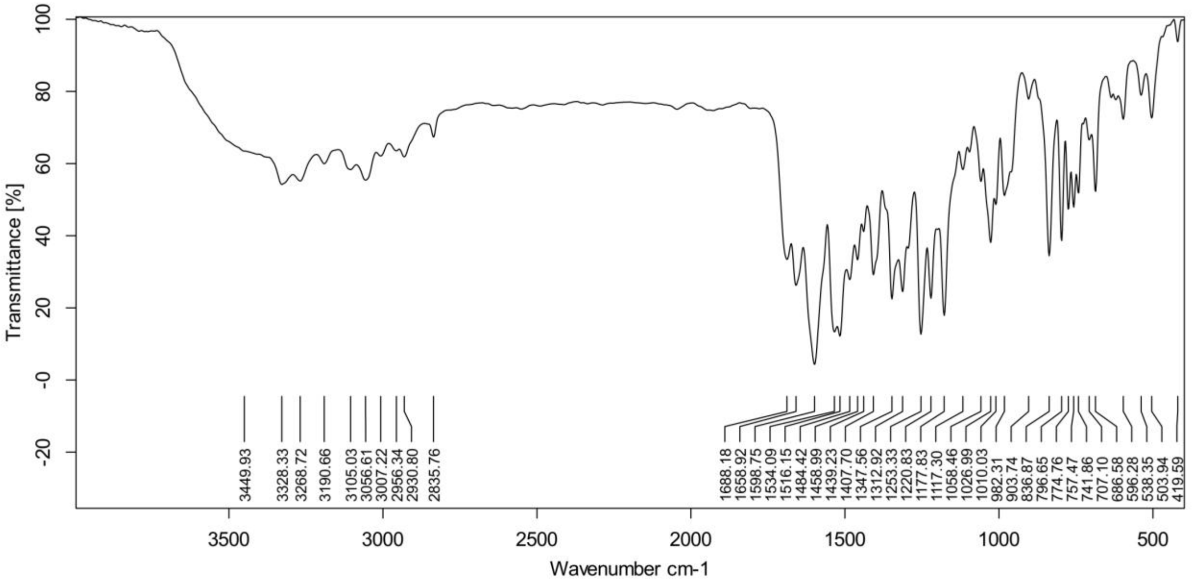

21_TRANS.2 12:43:19 ã 14/05/2024

IR results of compound 13i

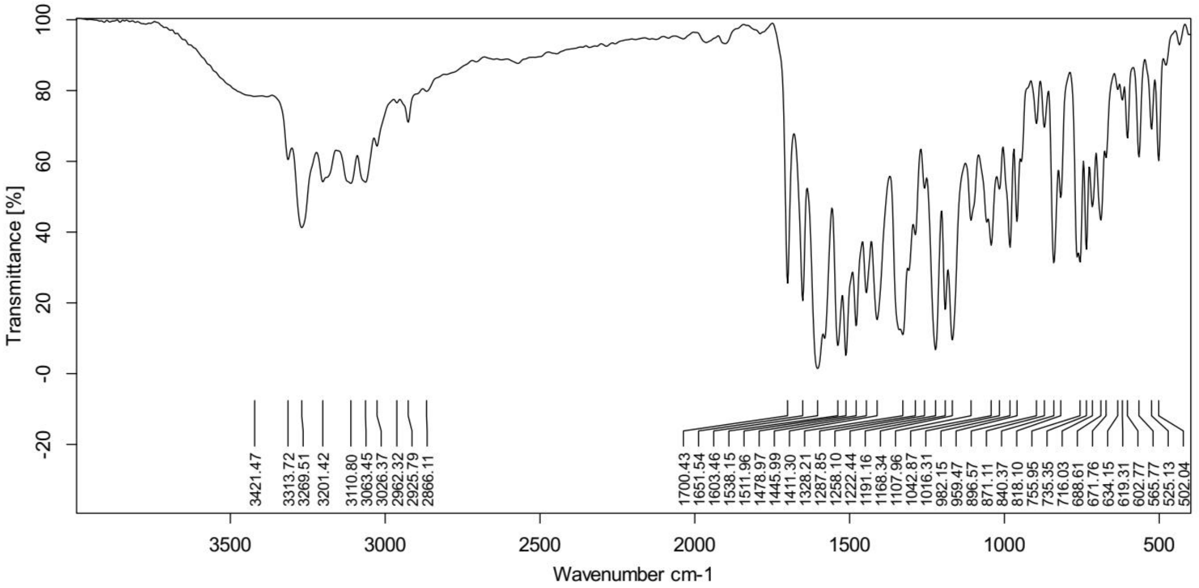

22_TRANS.3 **12:54:31 ã** **14/05/2024**

IR results of compound 14a

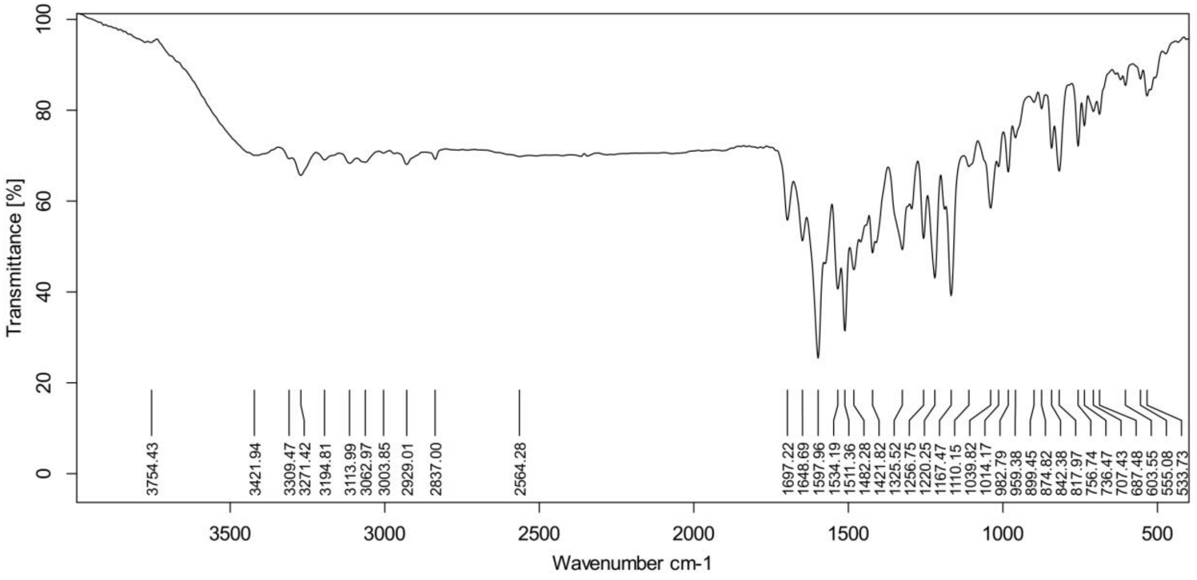

23_TRANS.2 **12:59:37 ã** **14/05/2024**

IR results of compound 14b

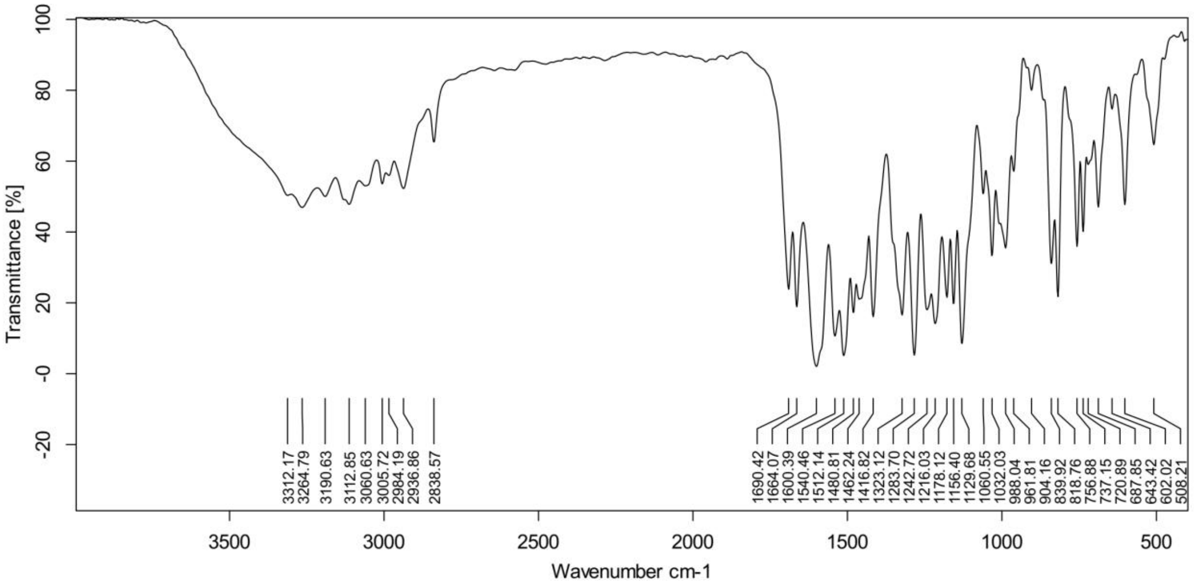

24_TRANS.4 01:06:13 ã 14/05/2024

IR results of compound 14c

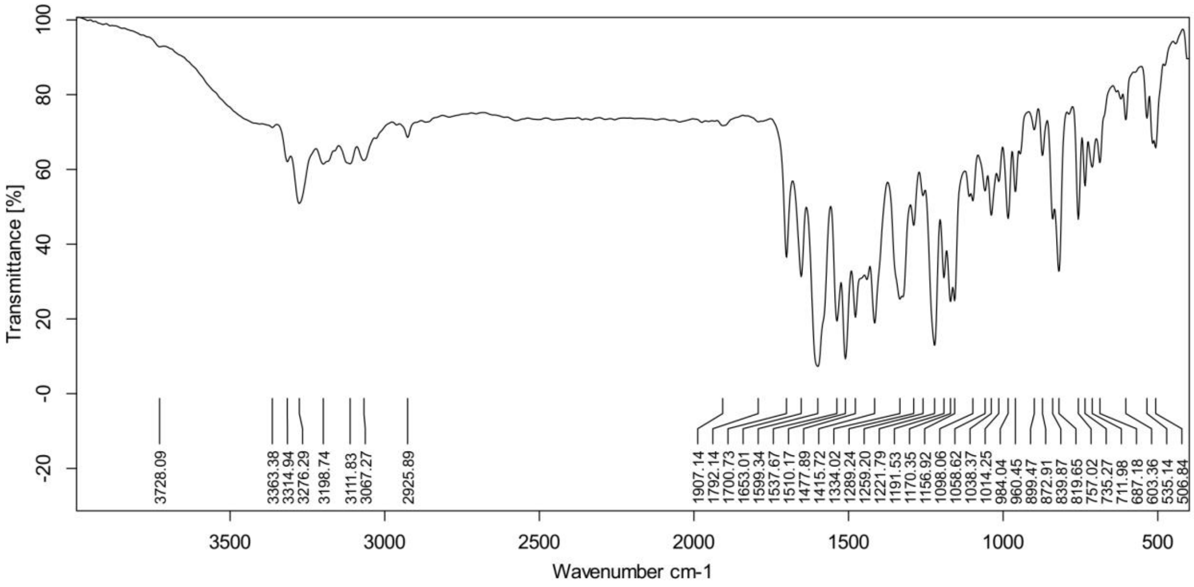

25_TRANS.1 01:12:01 ã 14/05/2024

IR results of compound 14d

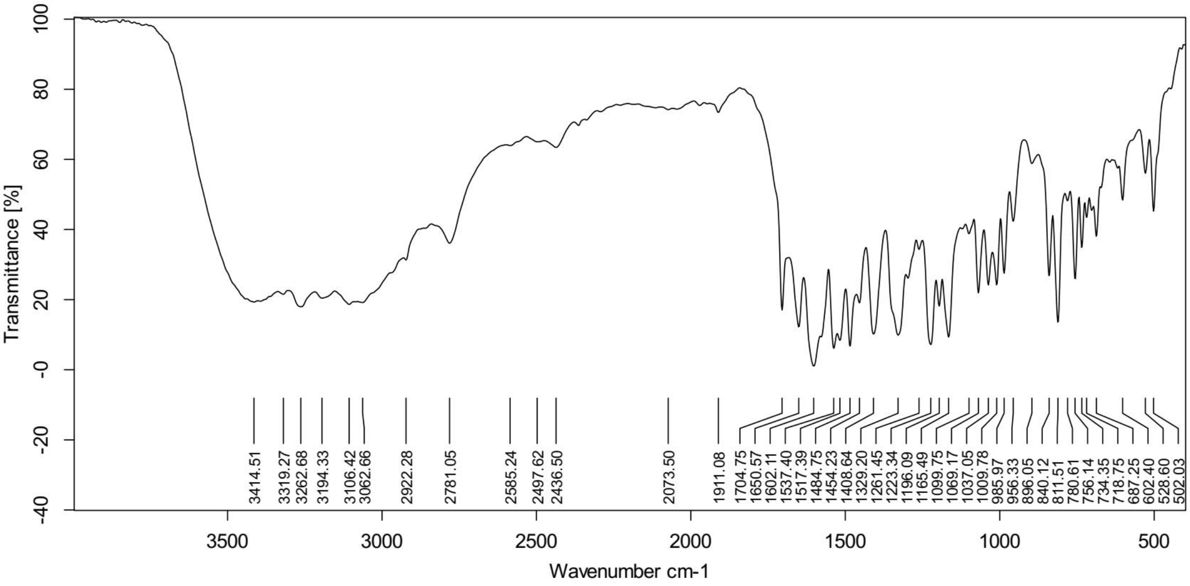

26_TRANS.2 **01:18:55 ã** **14/05/2024**

IR results of compound 14e

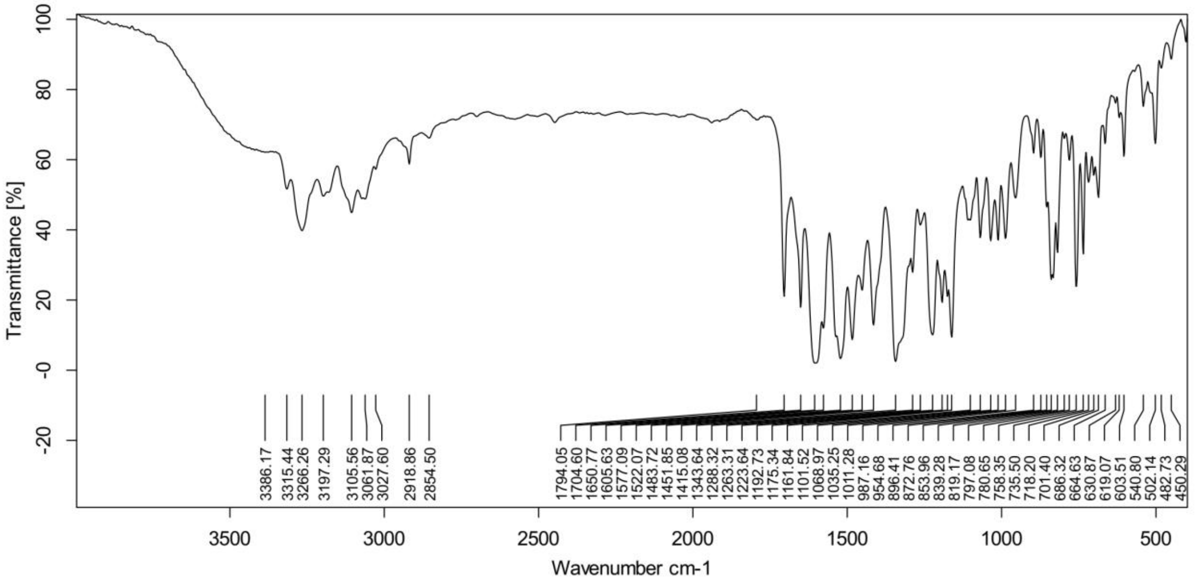

27_TRANS.1 **01:25:22 ã** **14/05/2024**

IR results of compound 14f

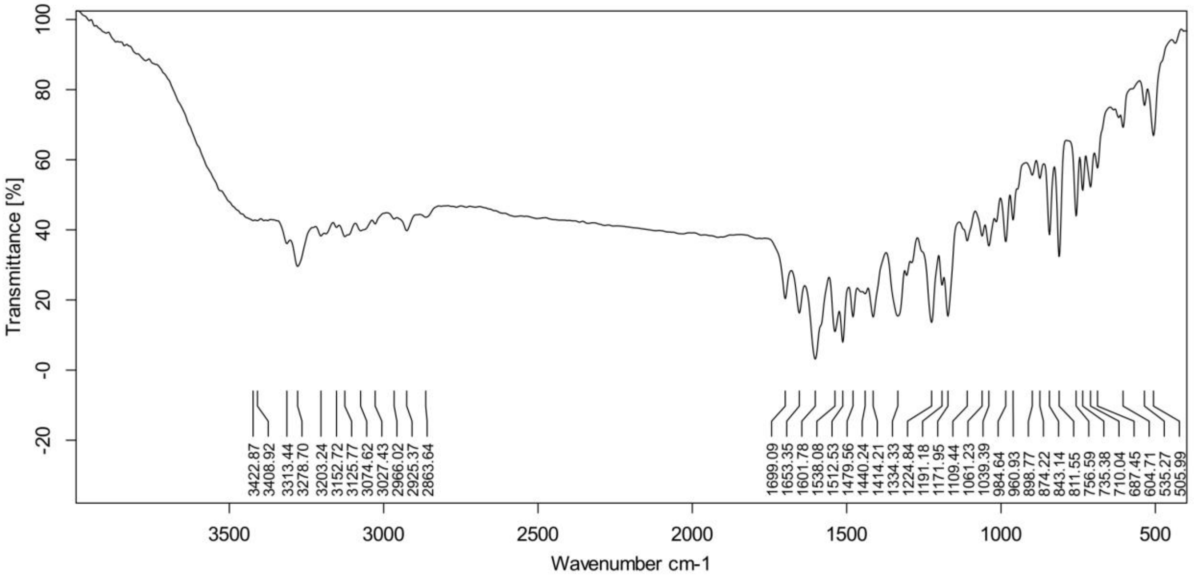

28_TRANS.1 **01:36:31 ã** **14/05/2024**

IR results of compound 14g

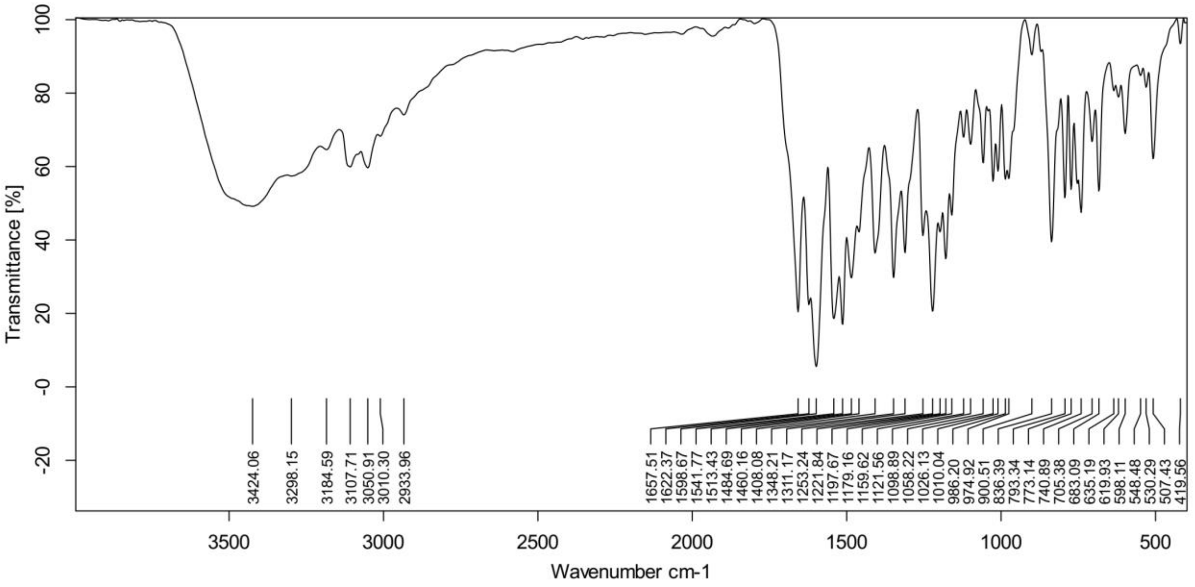

29_TRANS.2 **01:35:54 ã** **14/05/2024**

IR results of compound 13h

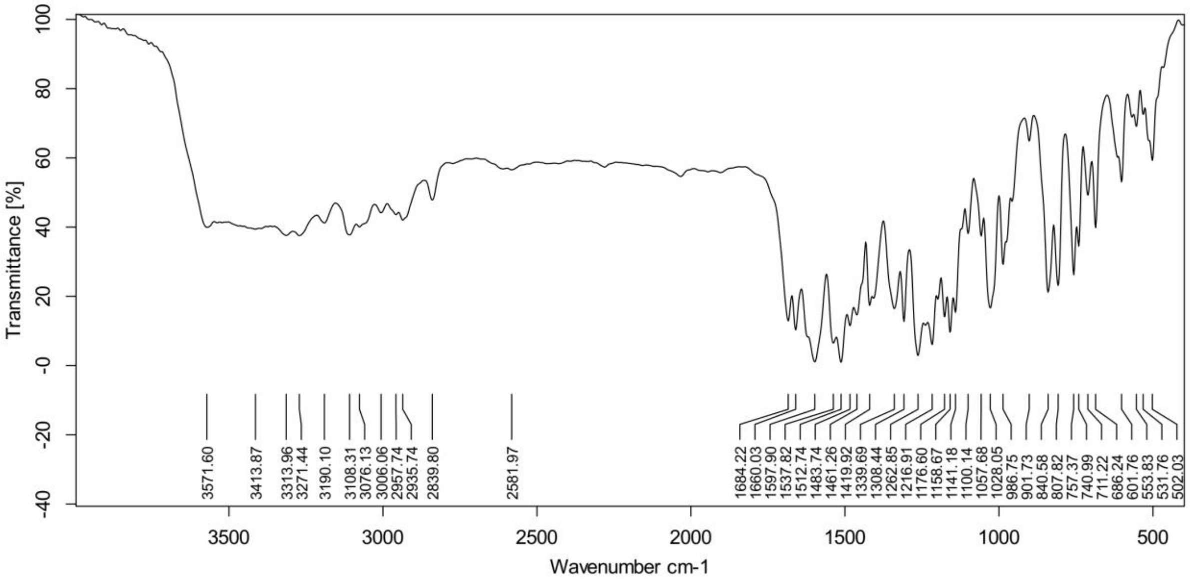

30_TRANS.2 **01:40:18 ã** **14/05/2024**

IR results of compound 13i

^{1}H NMR (400 MHz, DMSO-d_6) and ^{13}C NMR (100 MHz, DMSO-d_6)for the newly synthesized compounds

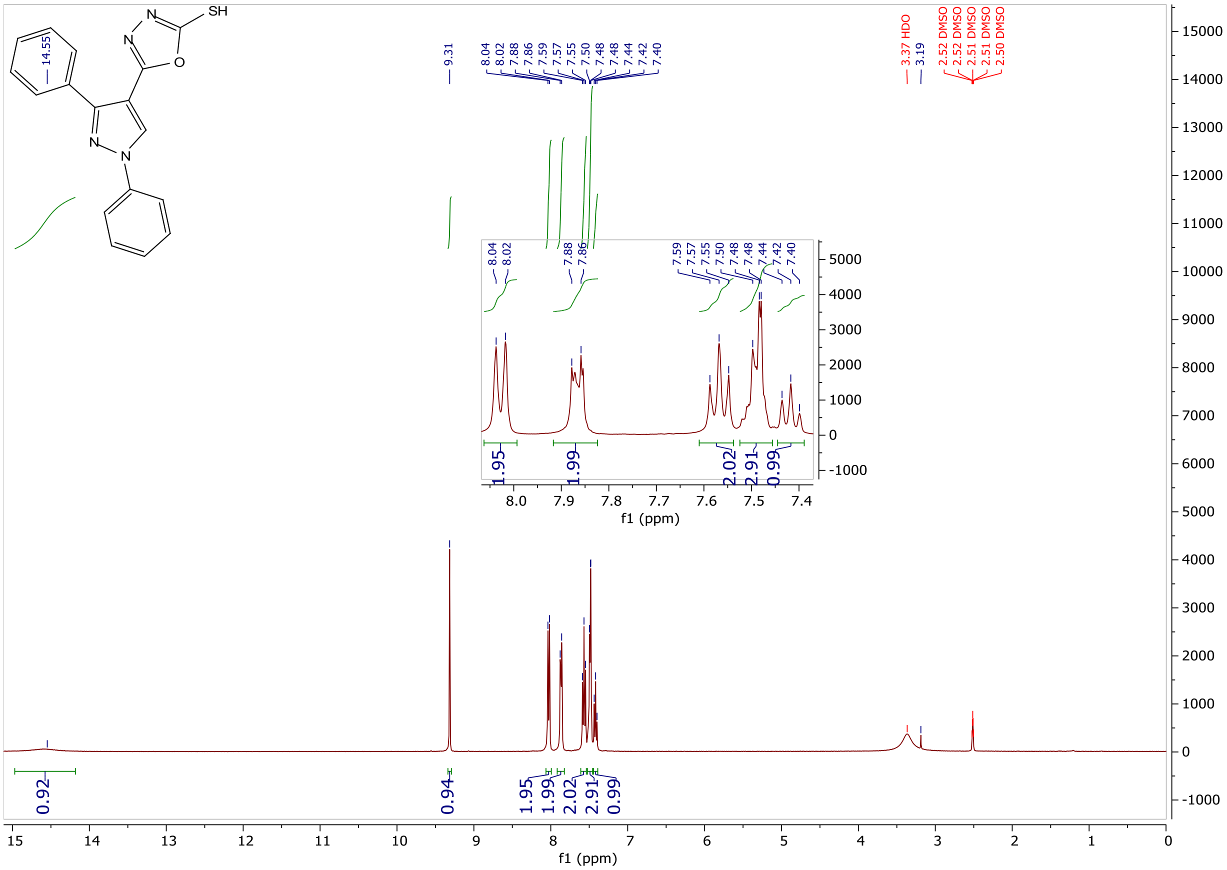

^{1}H NMR spectrum of compound **9a**

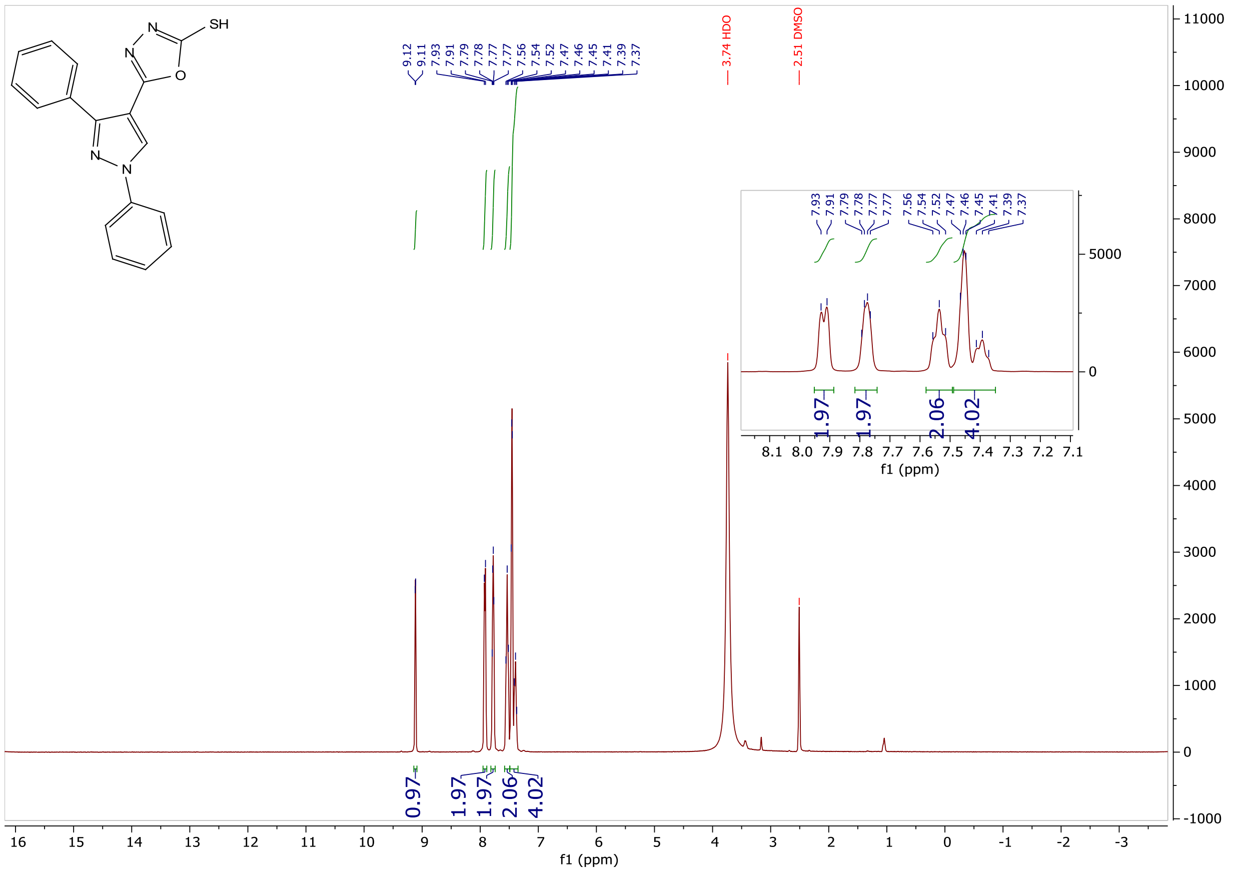

^{1}H NMR spectrum of compound **9a** (D_2O Treated)

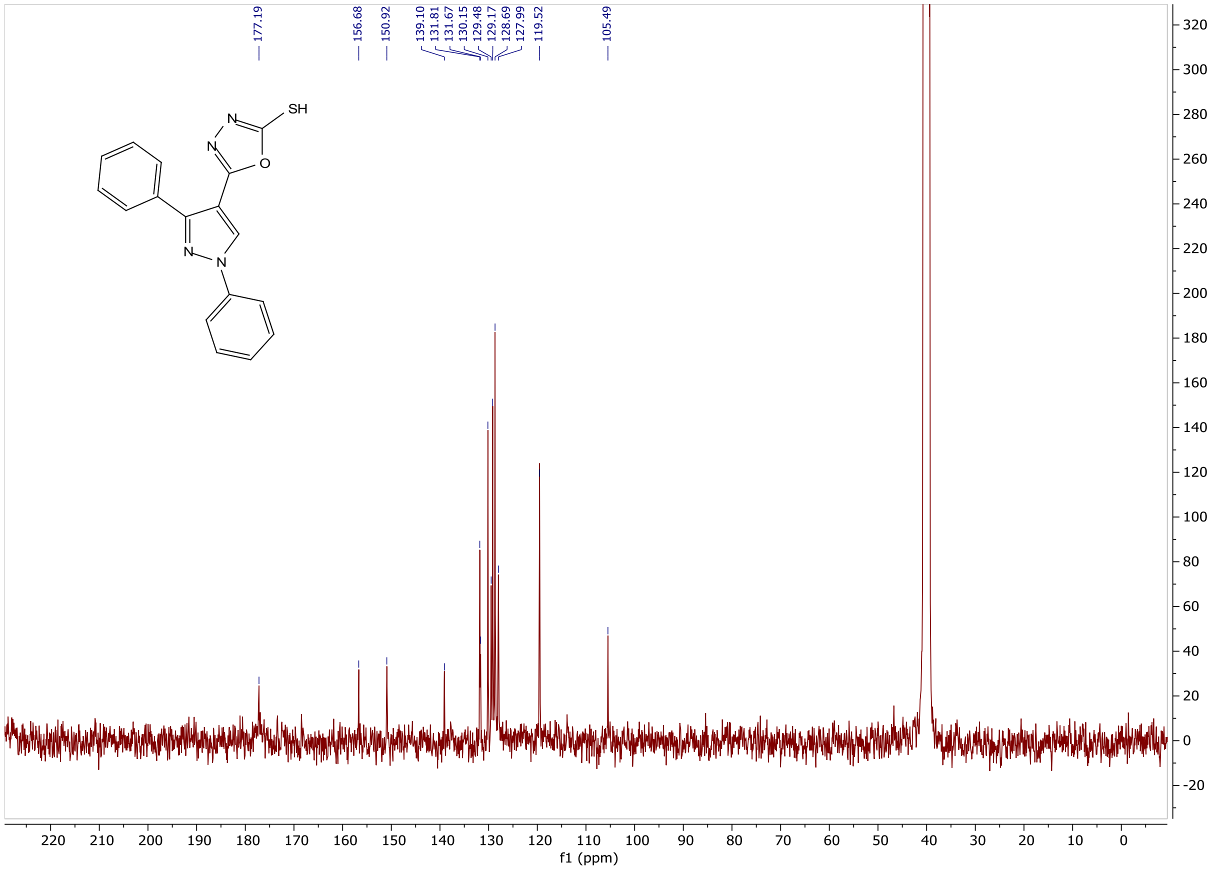

^{13}C NMR spectrum of compound **9a**

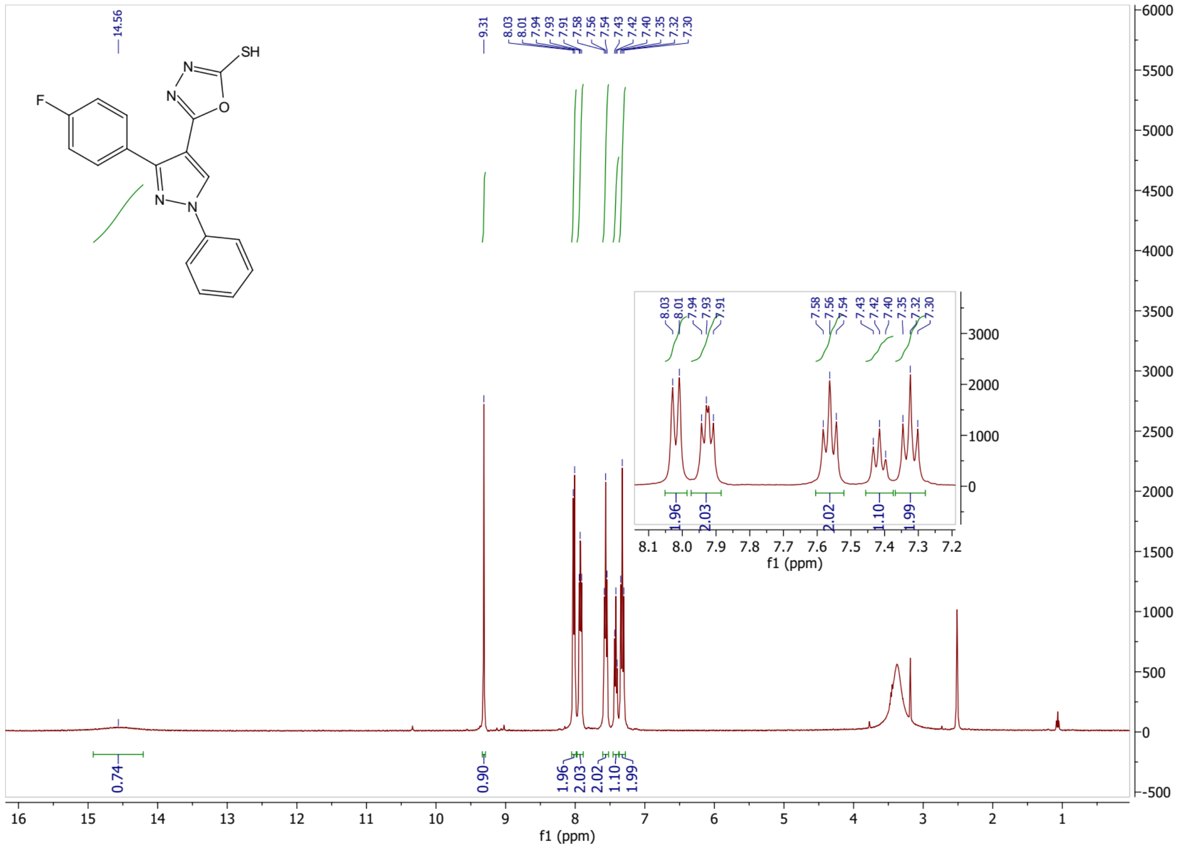

^{1}H NMR spectrum of compound **9b**

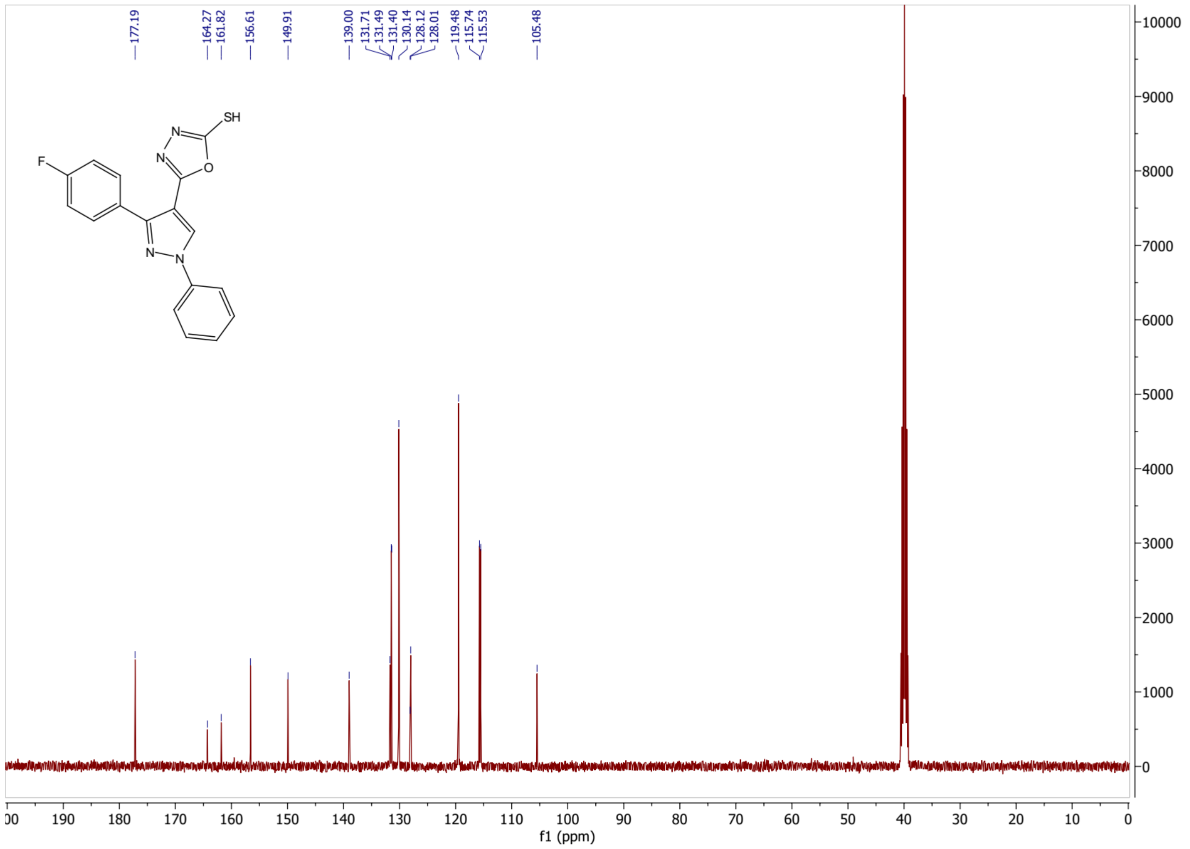

^{13}C NMR spectrum of compound **9b**

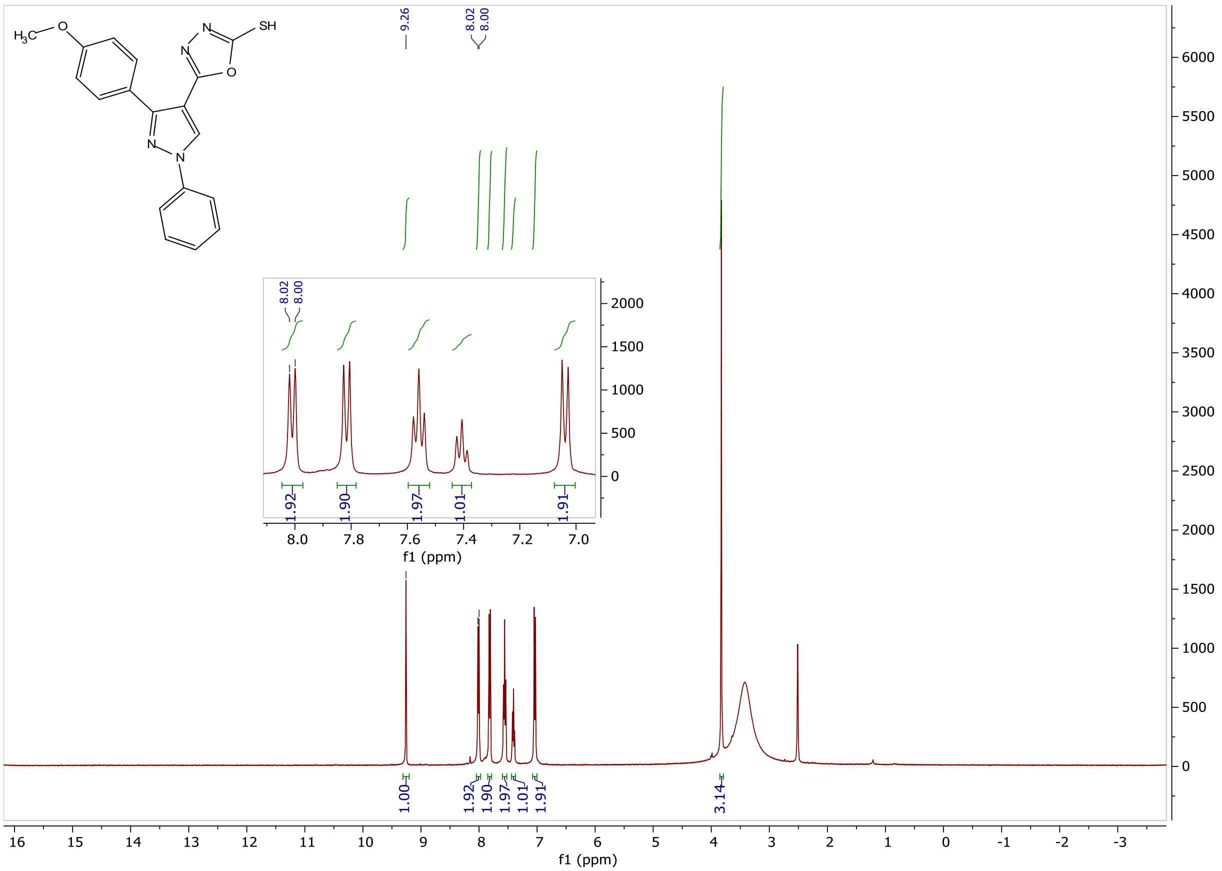

^{1}H NMR spectrum of compound **9c**

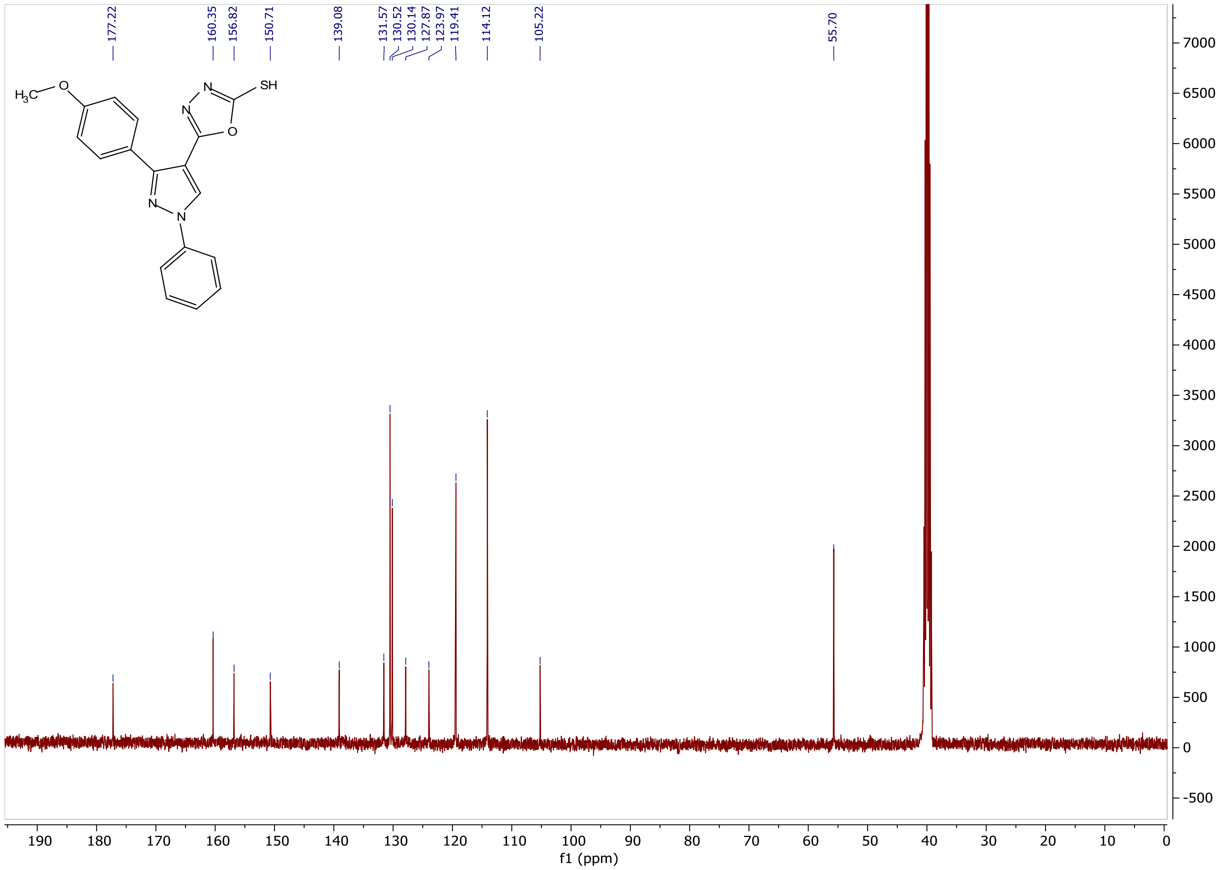

^{13}C NMR spectrum of compound **9c**

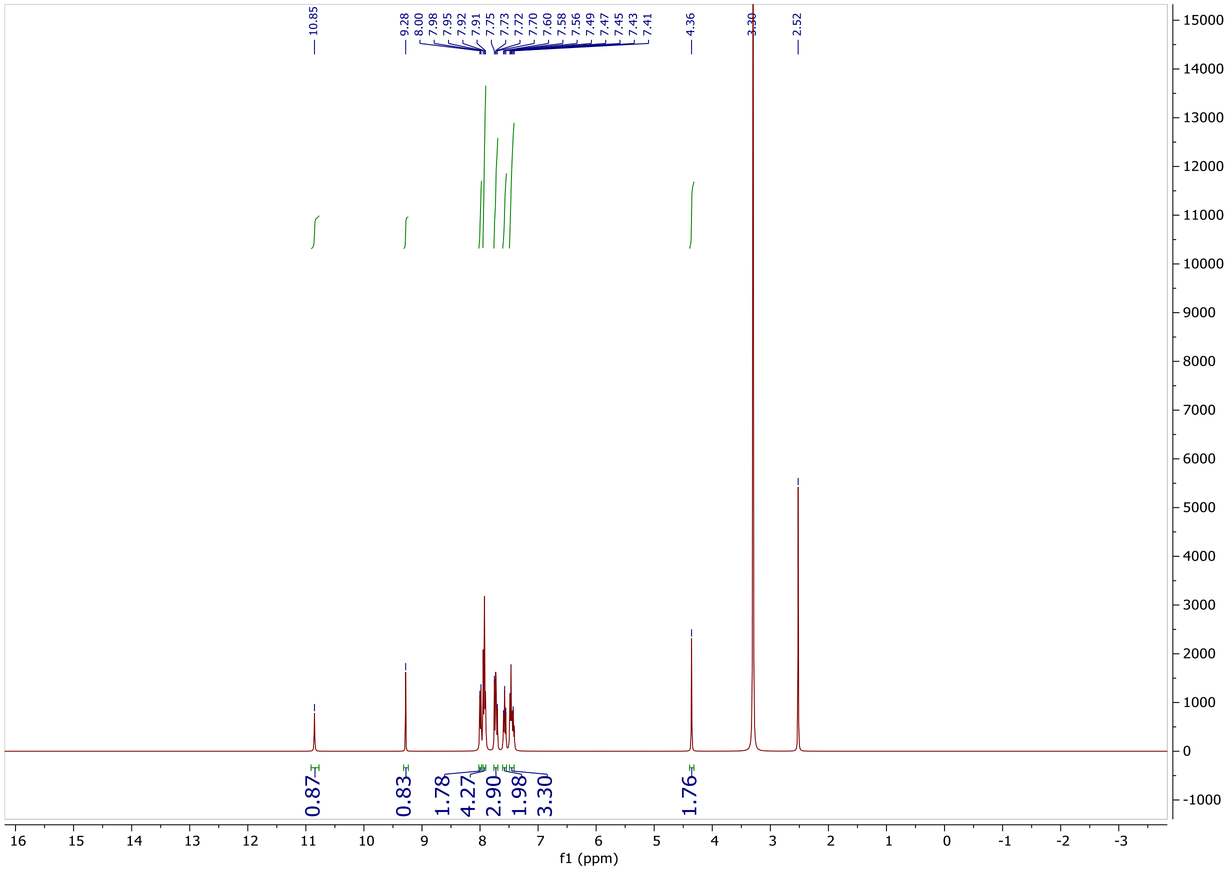

^{1}H NMR spectrum of compound **10a**

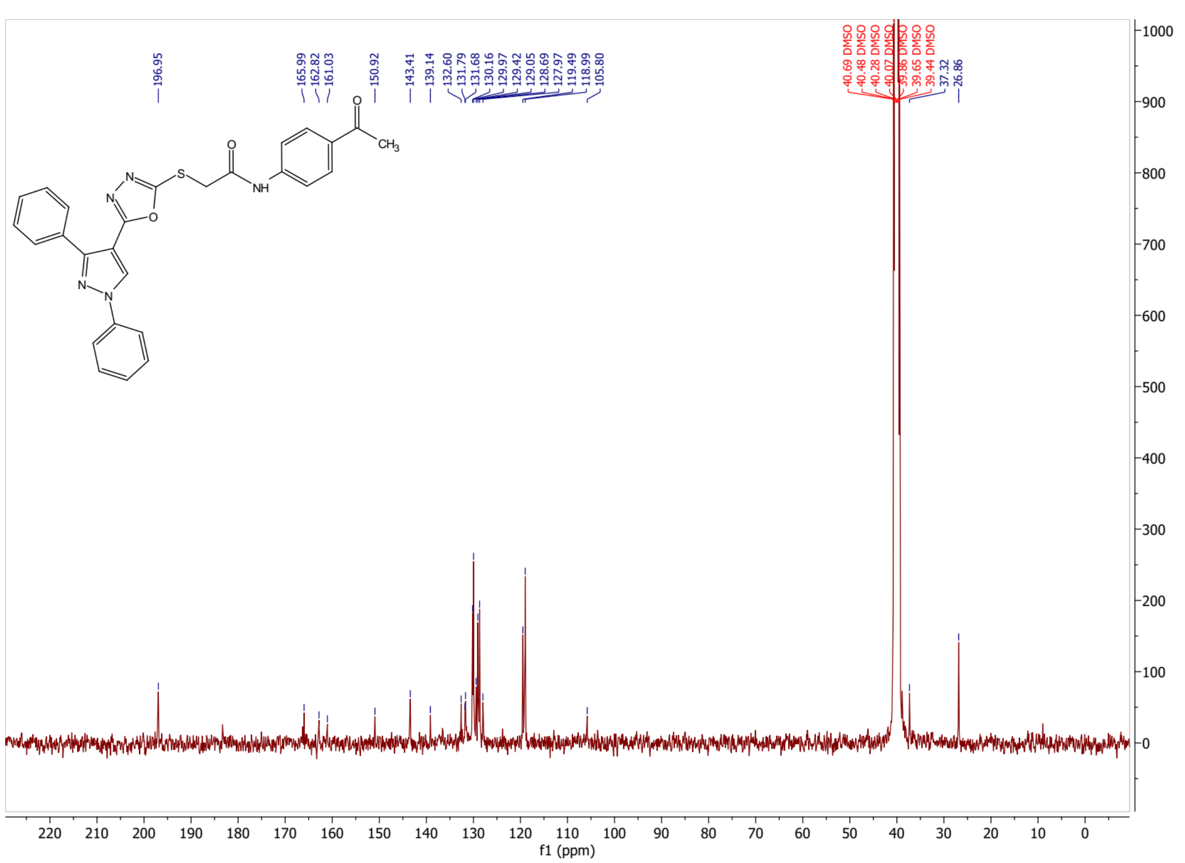

^{13}C NMR spectrum of compound **10a**

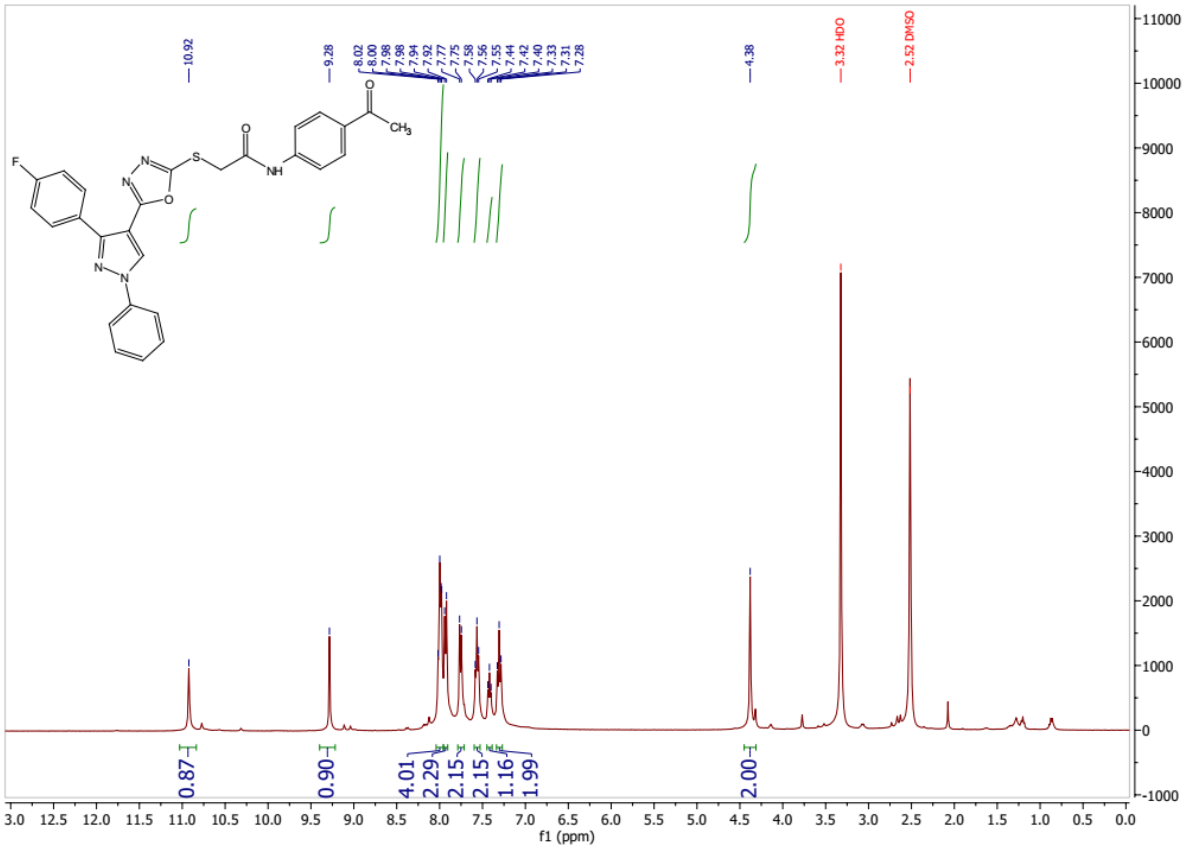

^{1}H NMR spectrum of compound **10b**

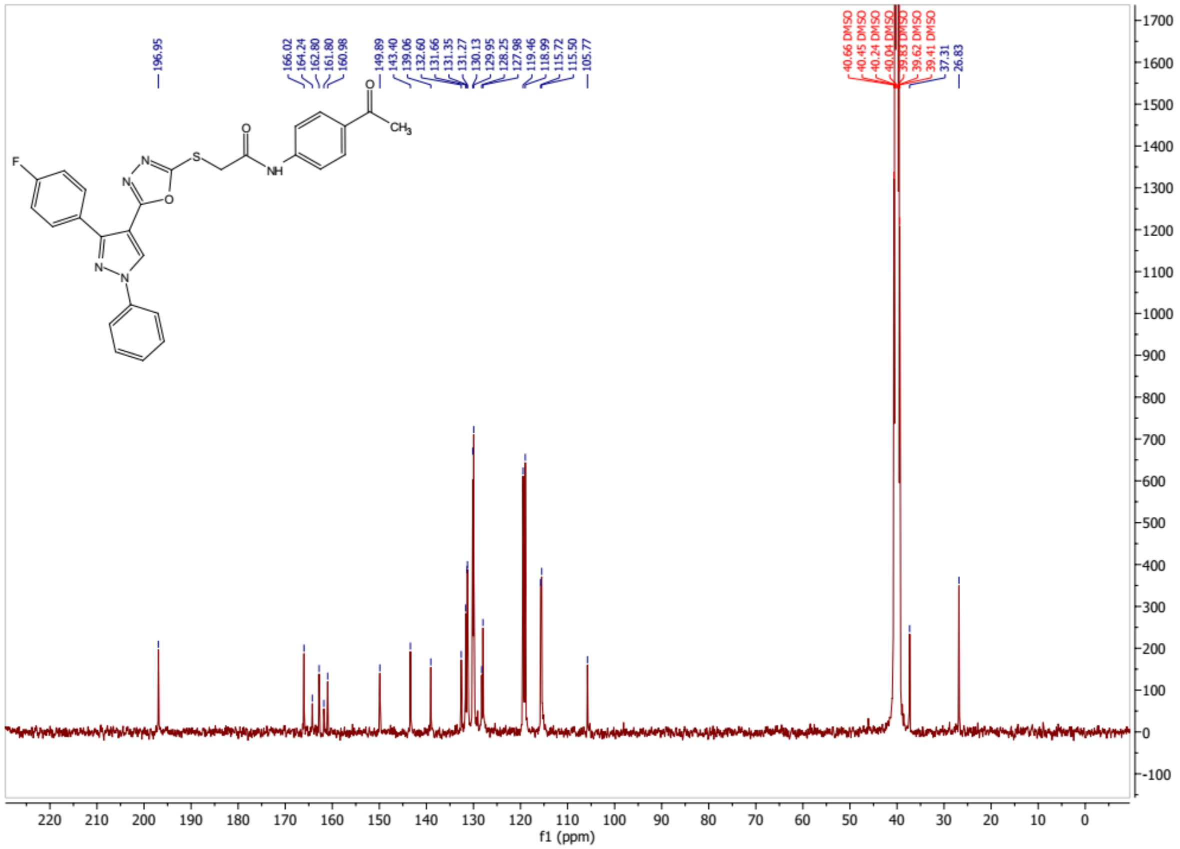

^{13}C NMR spectrum of compound **10b**

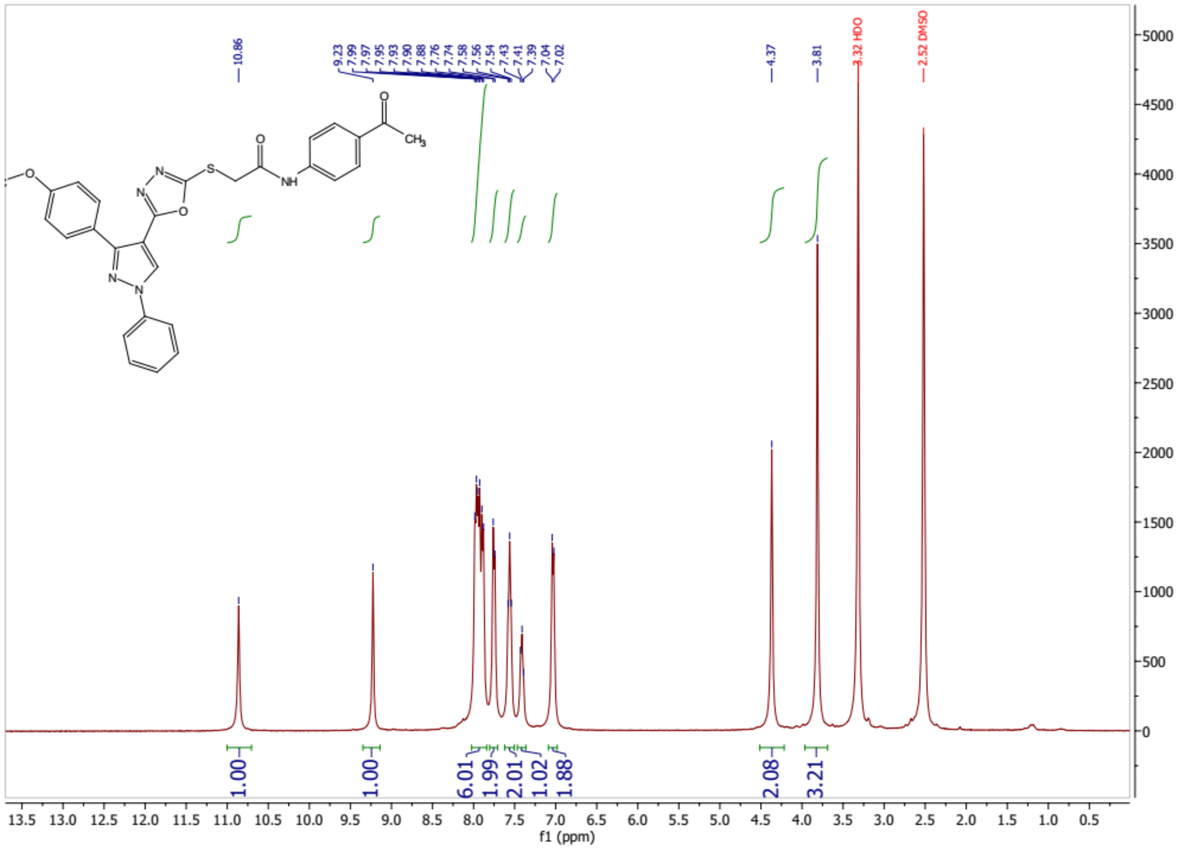

^{1}H NMR spectrum of compound **10c**

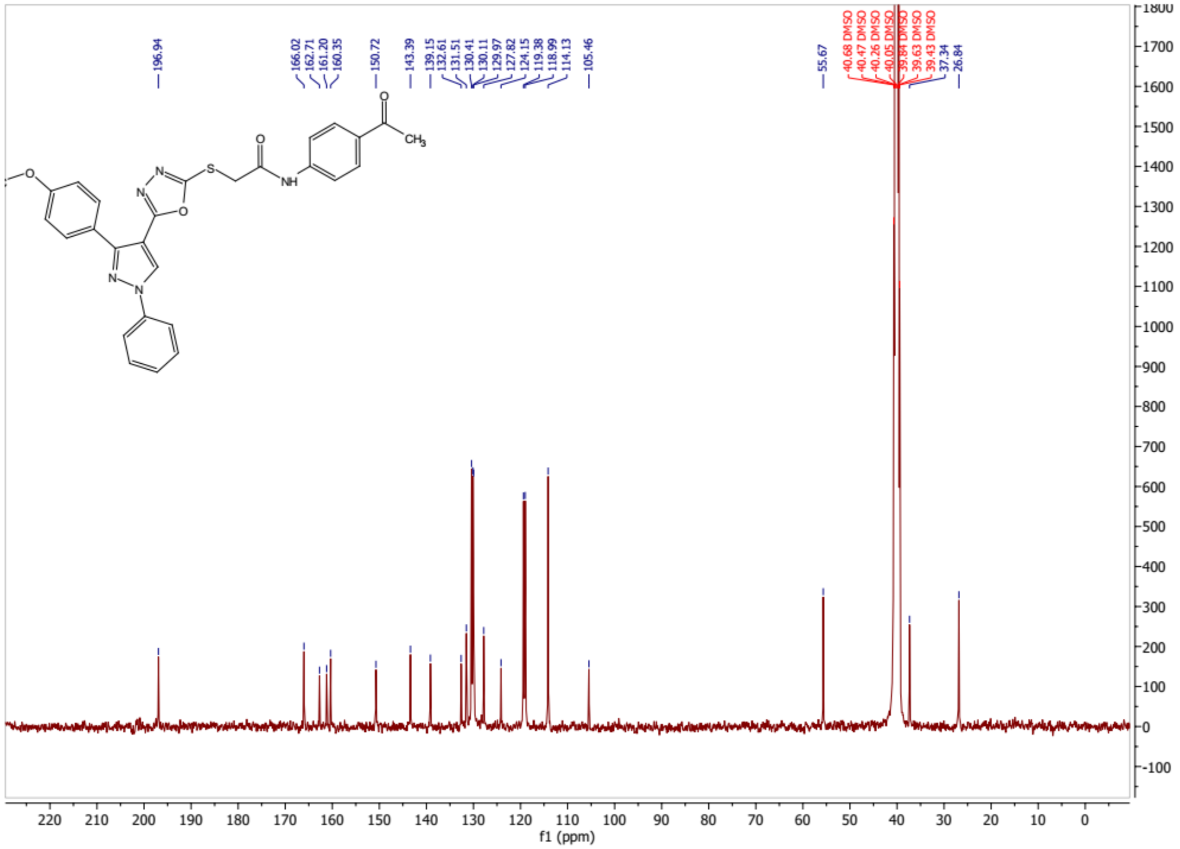

^{13}C NMR spectrum of compound **10c**

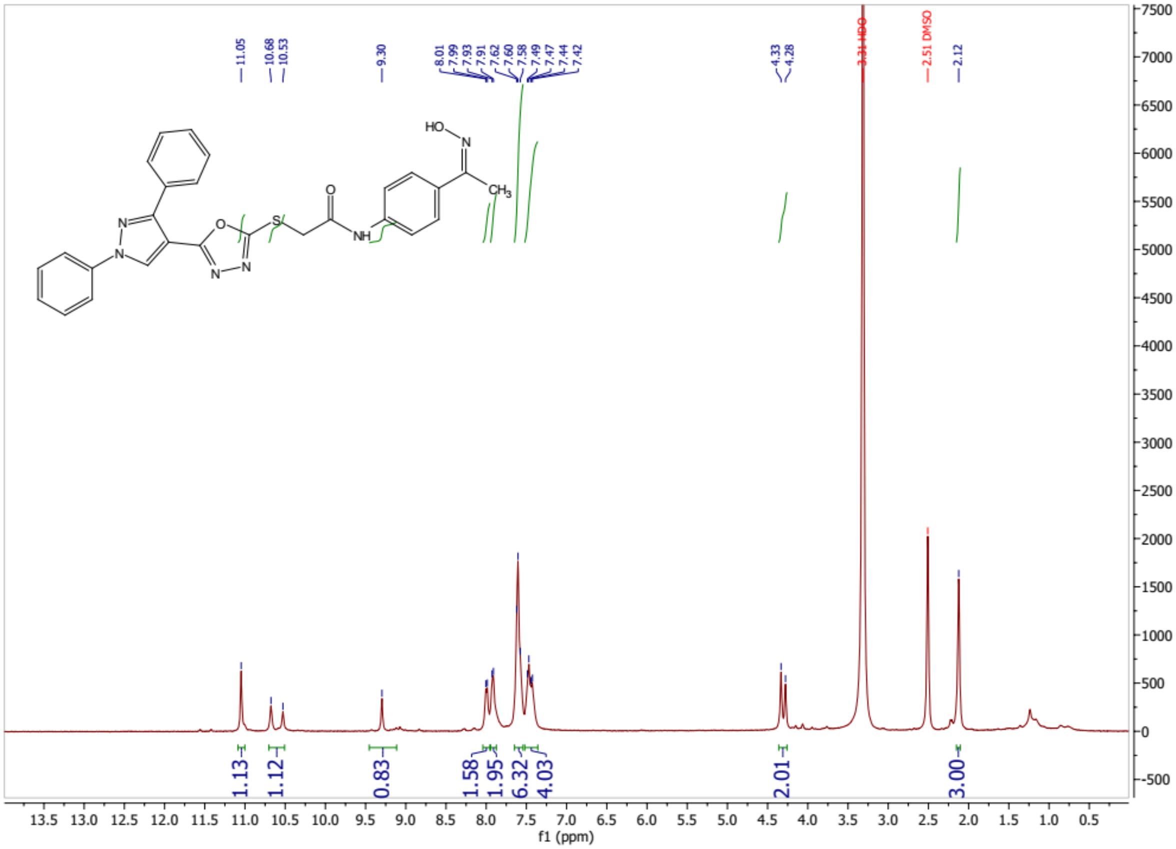

^{1}H NMR spectrum of compound **11a**

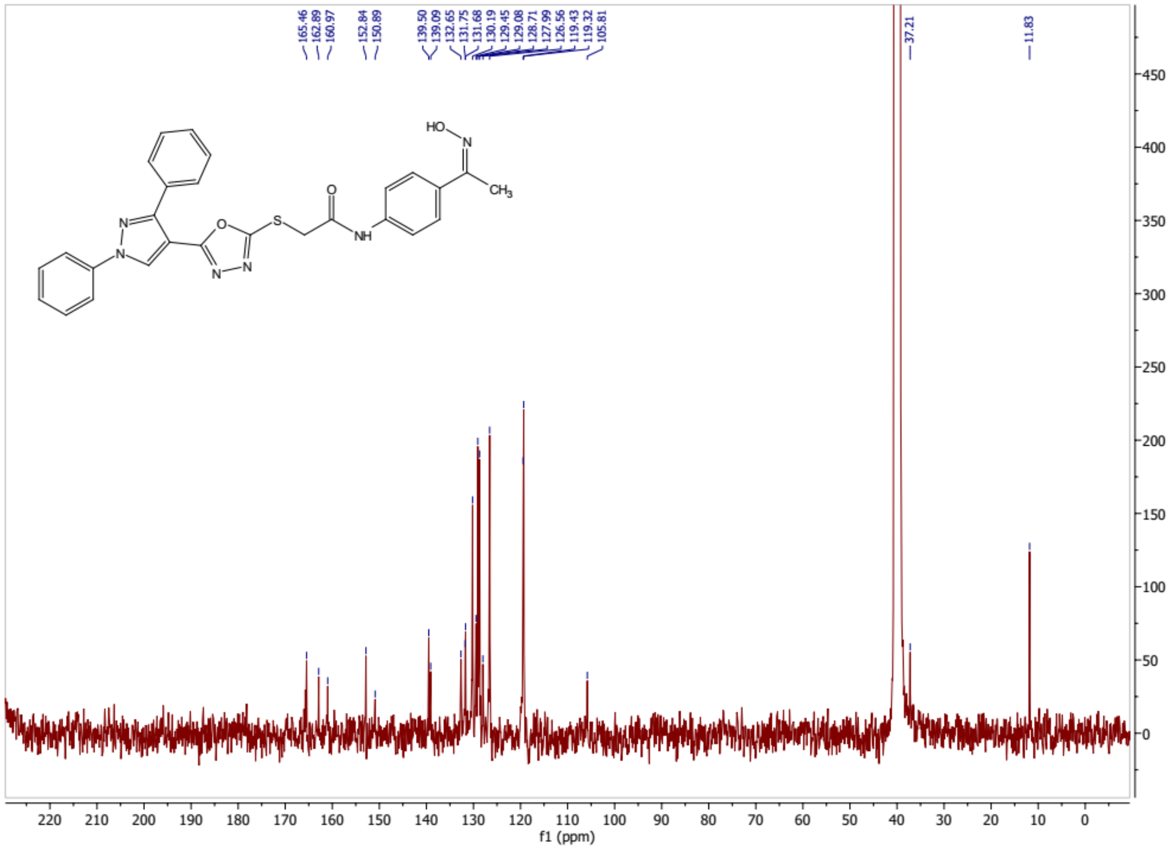

^{13}C NMR spectrum of compound **11a**

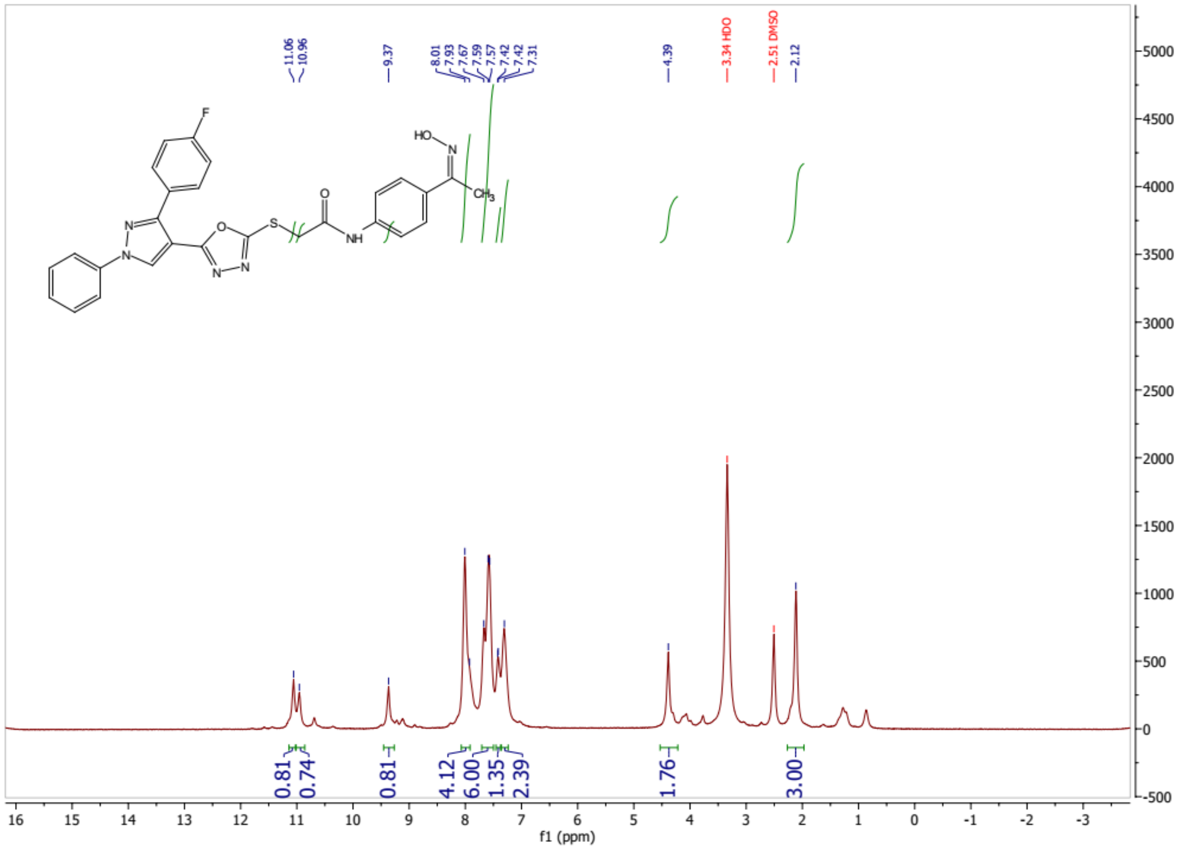

^{1}H NMR spectrum of compound **11b**

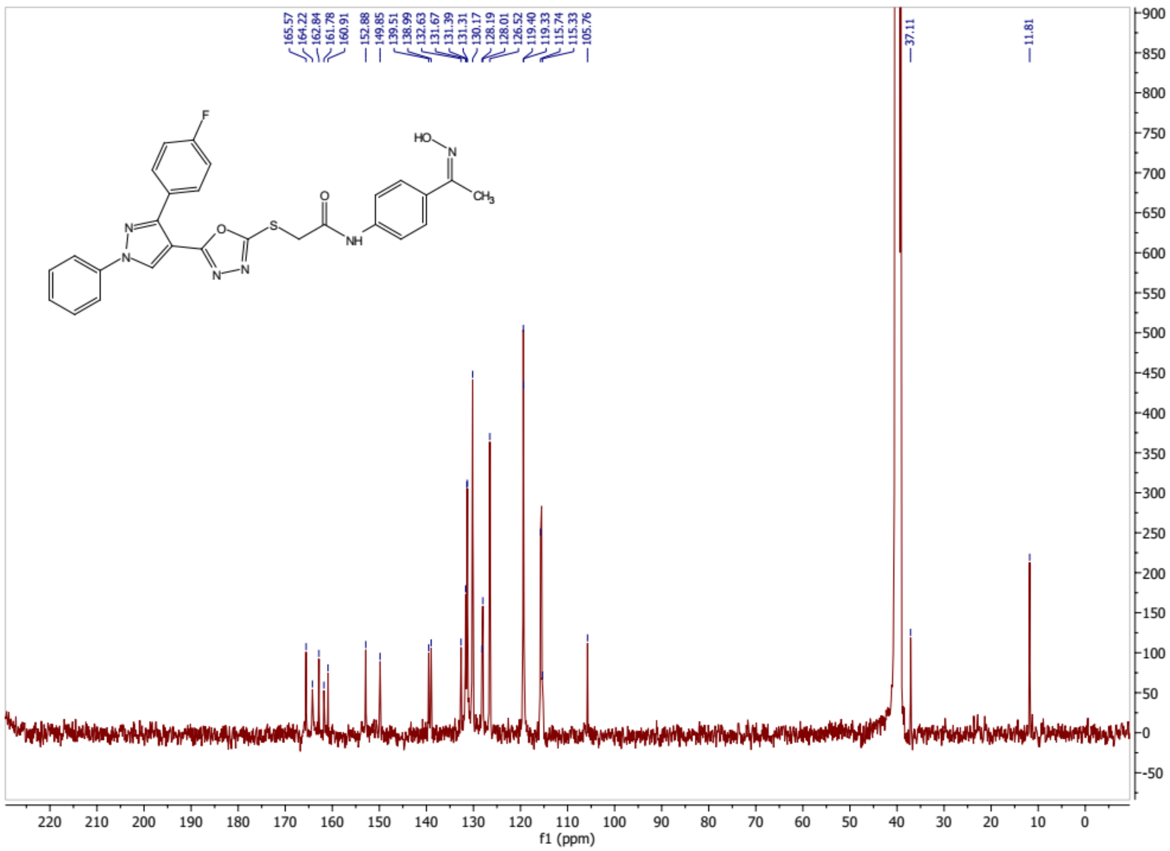

^{13}C NMR spectrum of compound **11b**

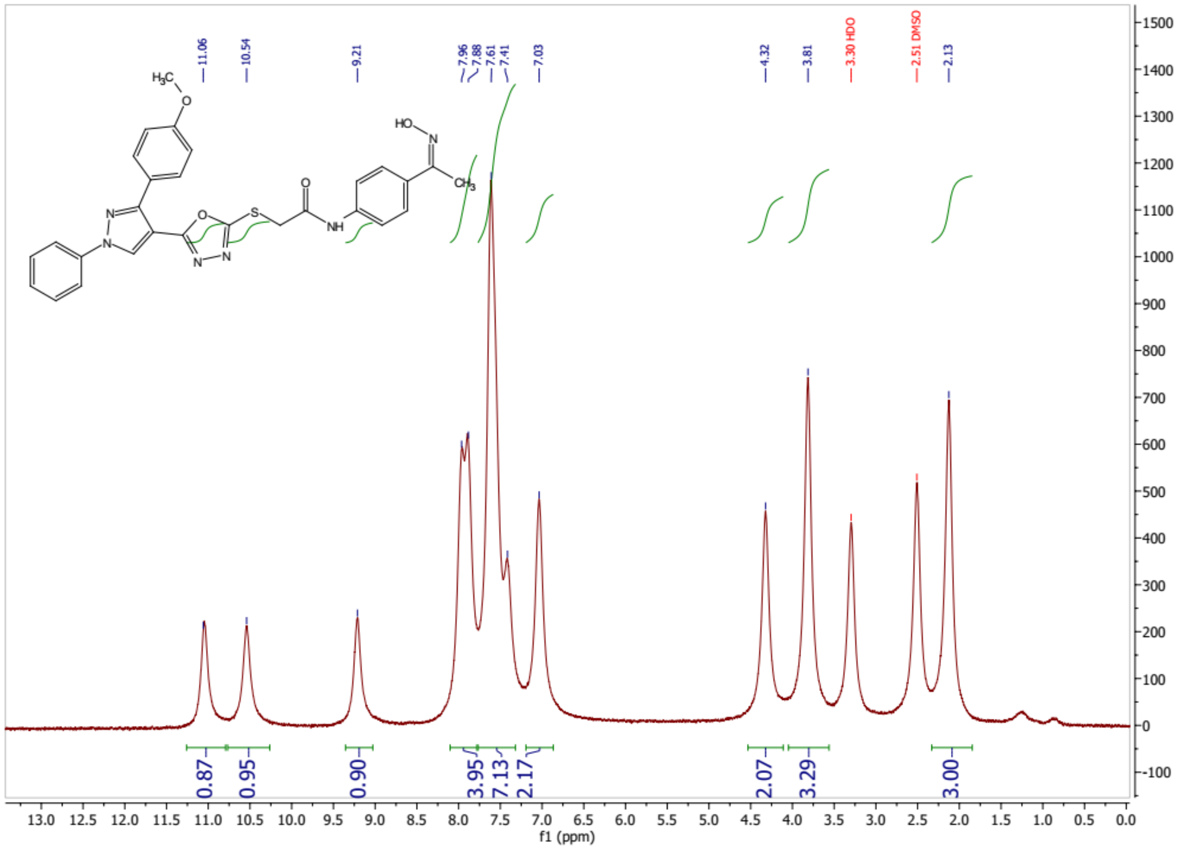

^{13}C NMR spectrum of compound **11c**

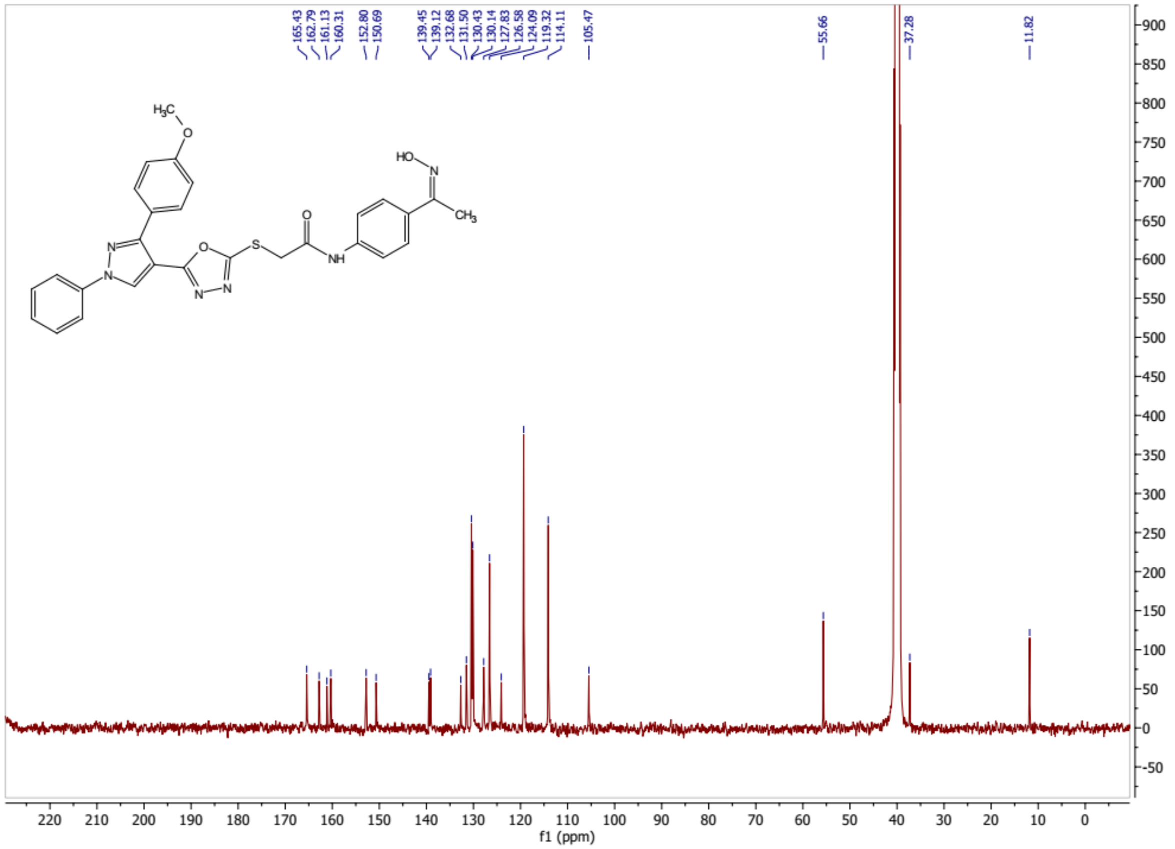

^{13}C NMR spectrum of compound **11c**

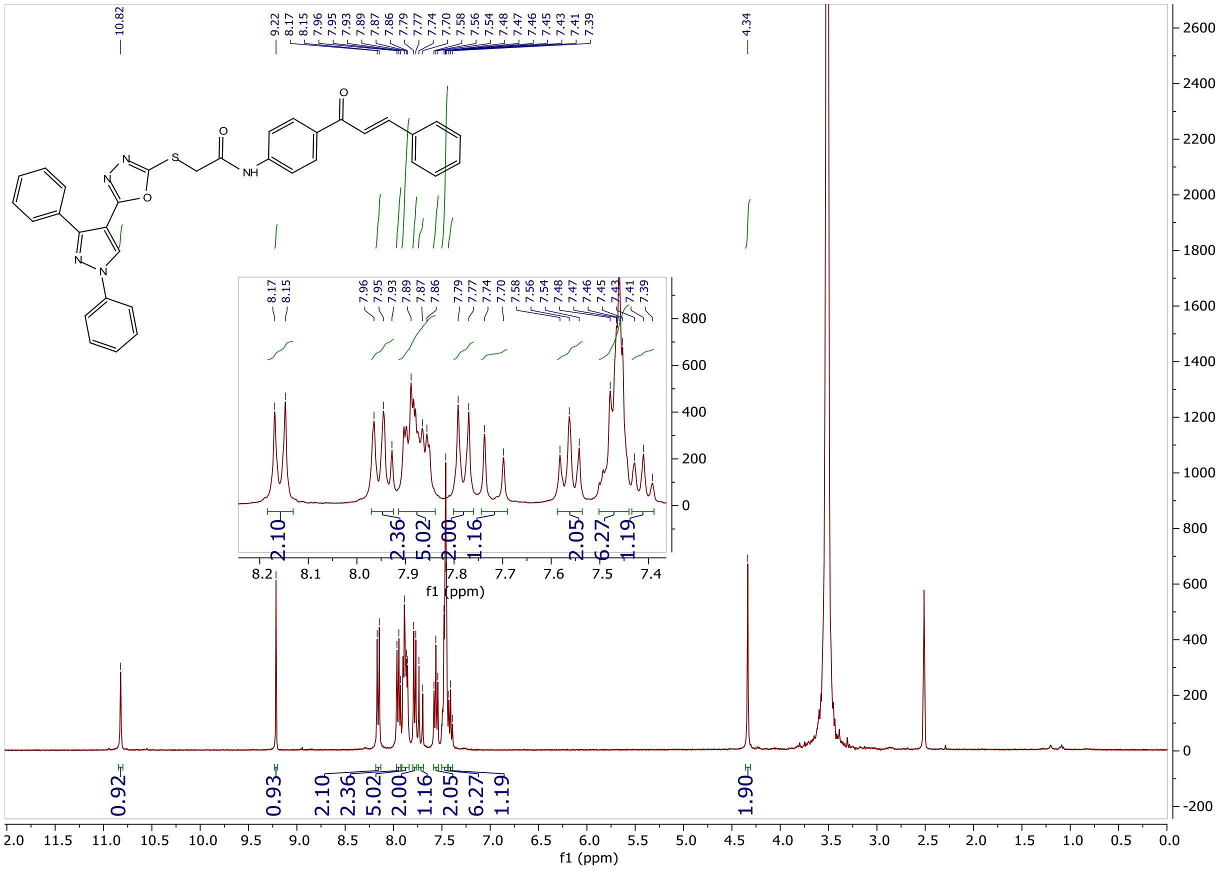

^{1}H NMR spectrum of compound **12a**

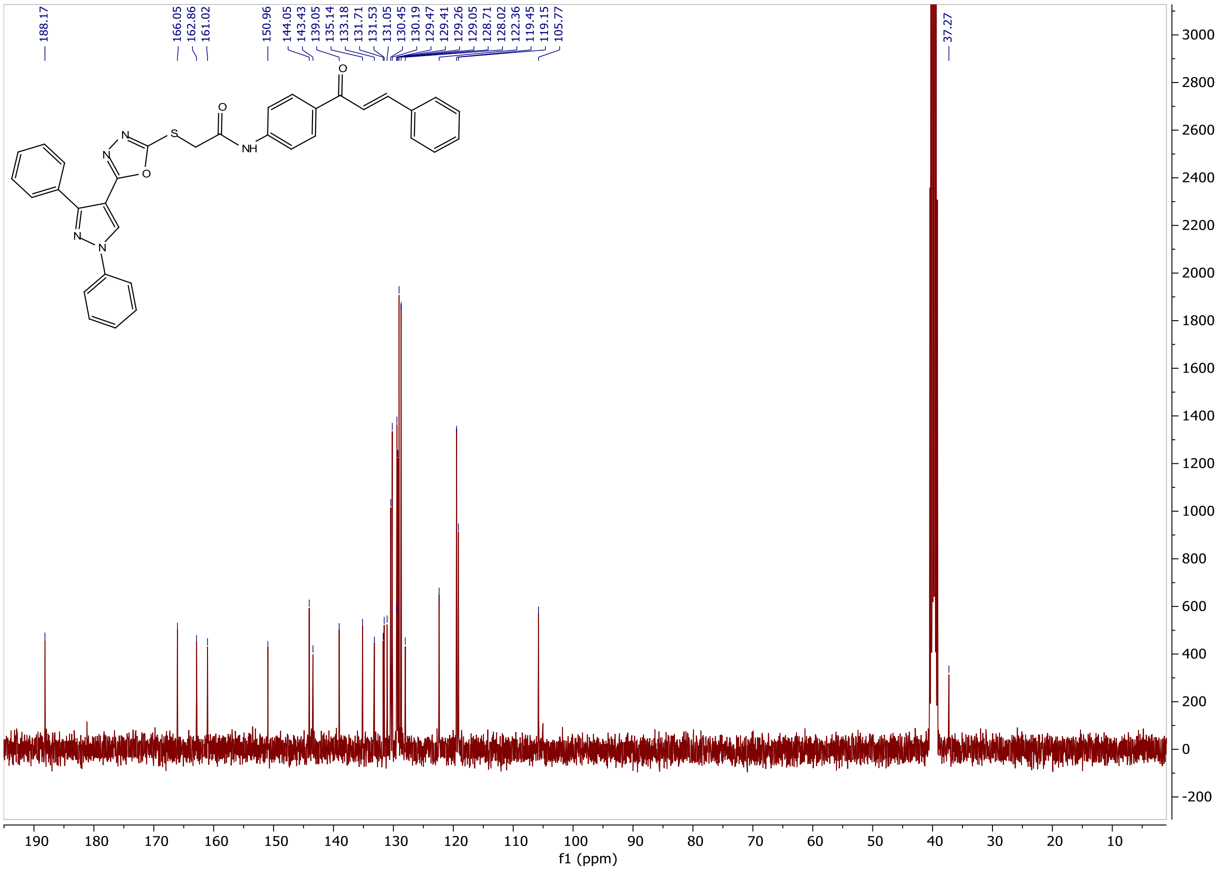

^{13}C NMR spectrum of compound **12a**

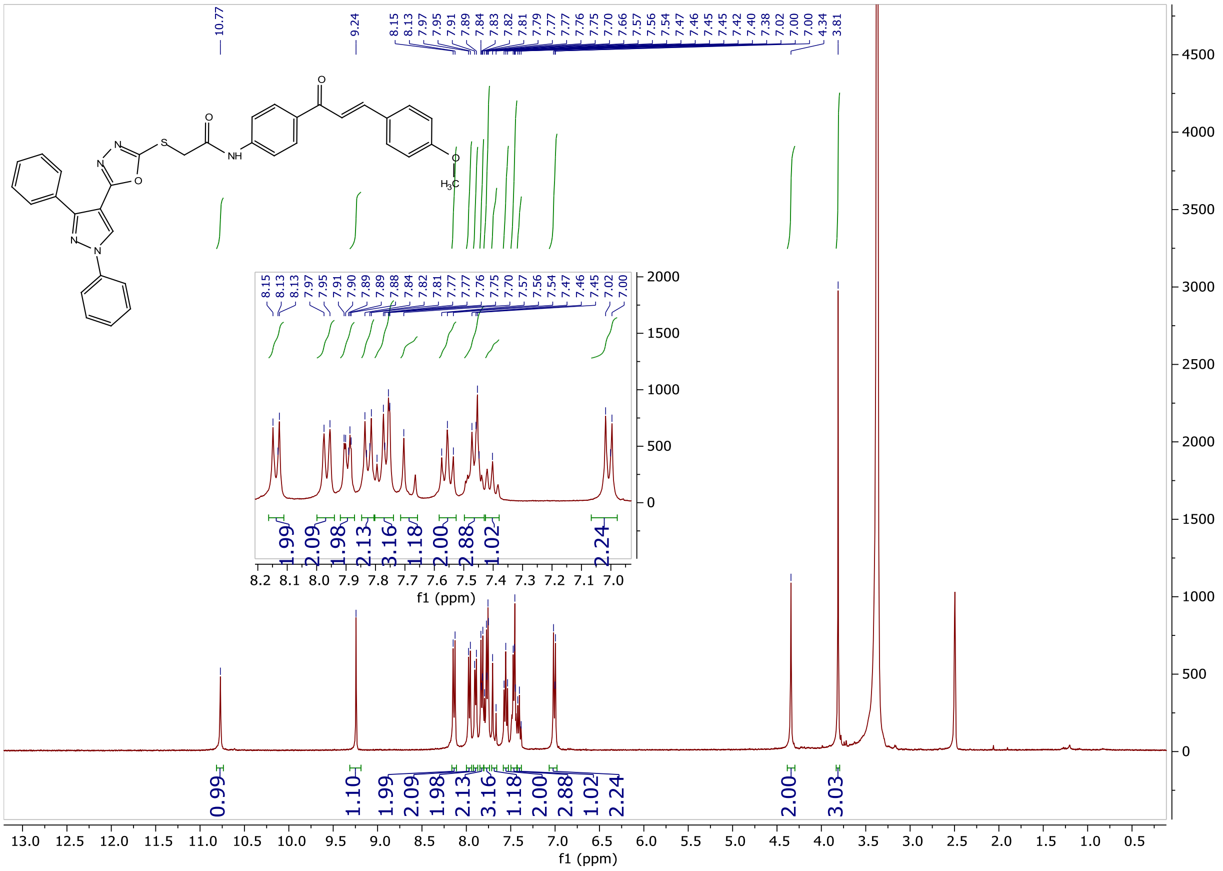

^{1}H NMR spectrum of compound **12b**

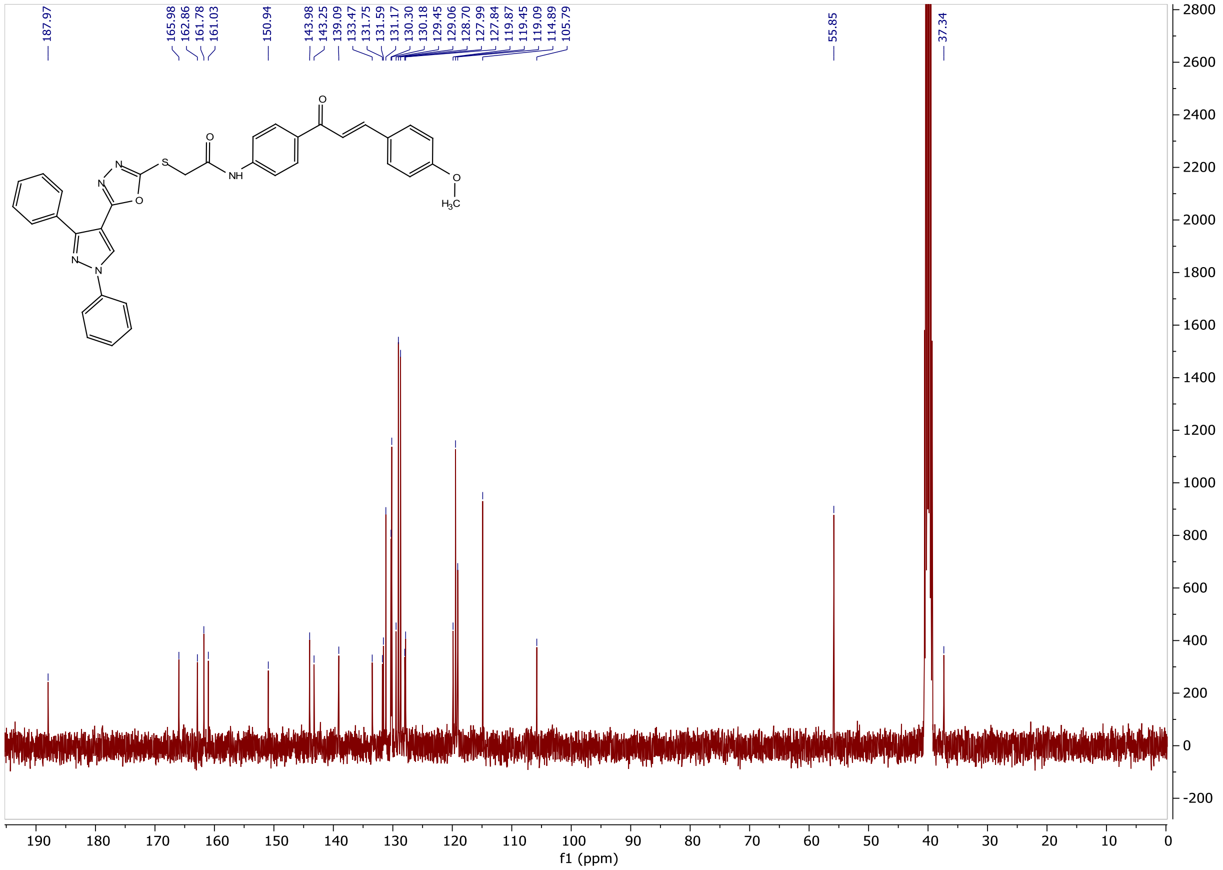

^{13}C NMR spectrum of compound **12b**

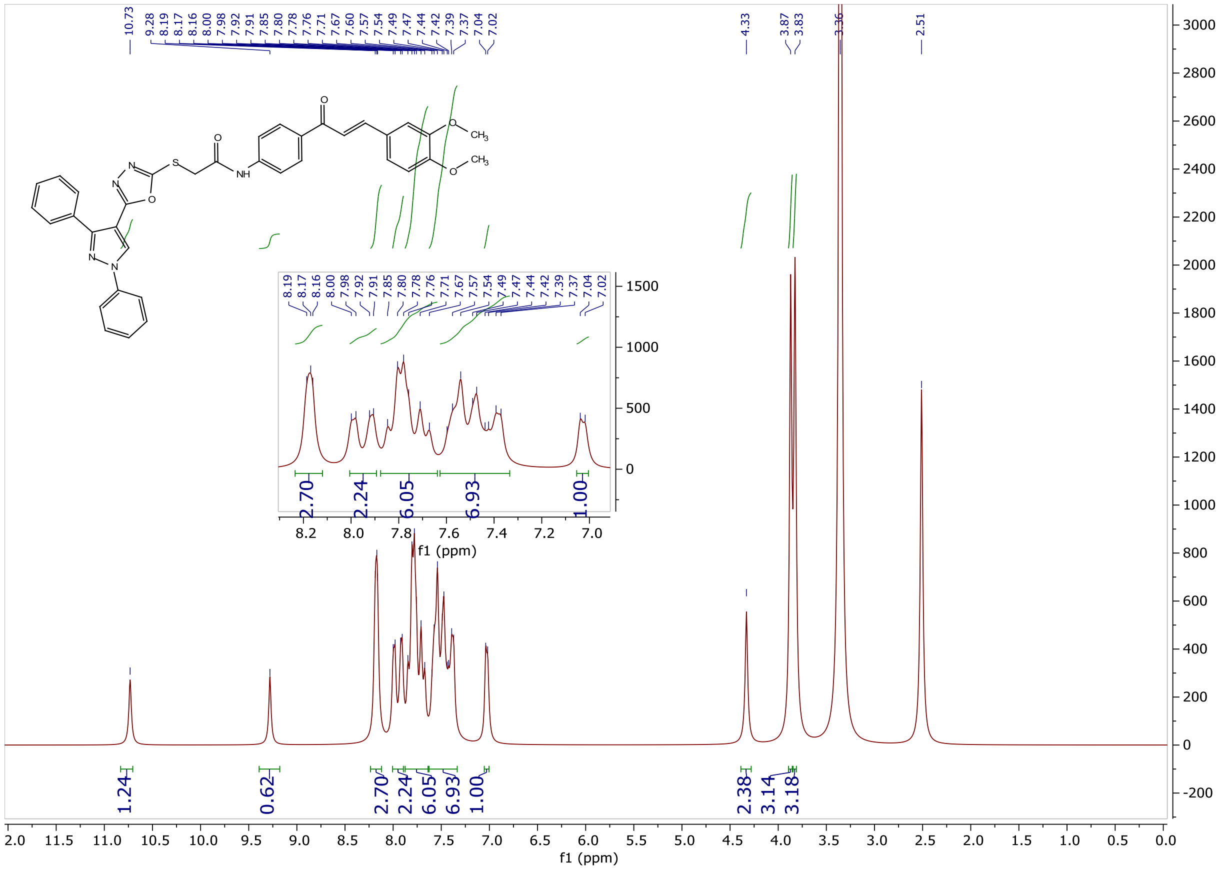

^{1}H NMR spectrum of compound **12c**

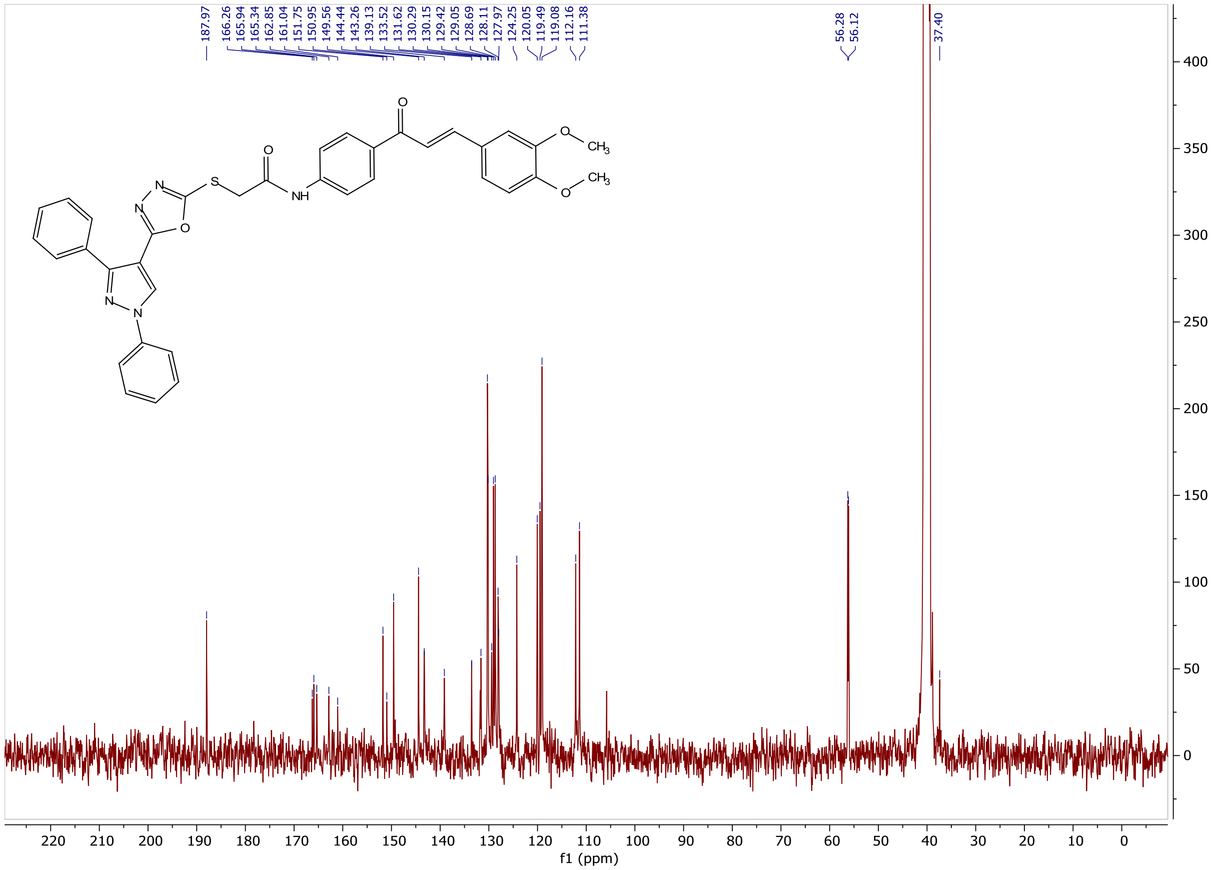

^{13}C NMR spectrum of compound **12c**

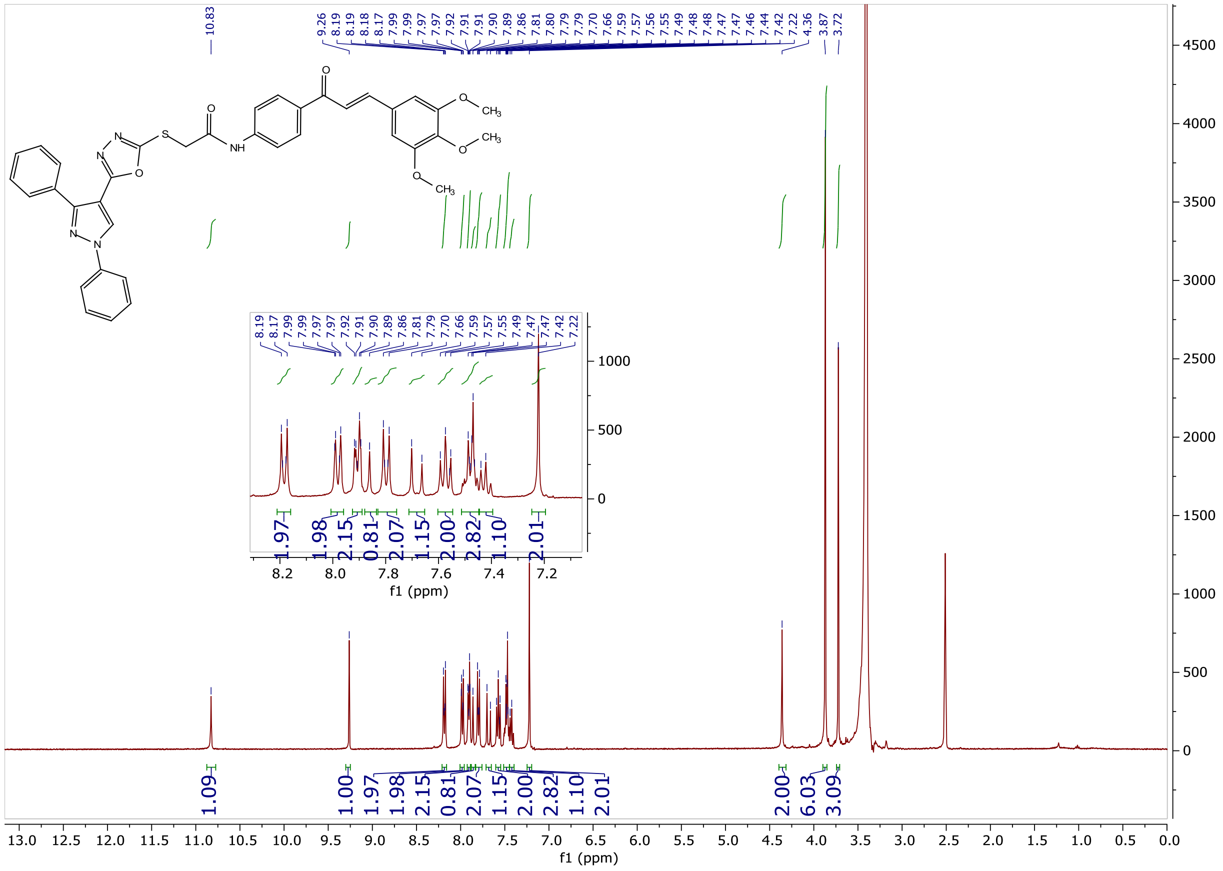

^{1}H NMR spectrum of compound **12d**

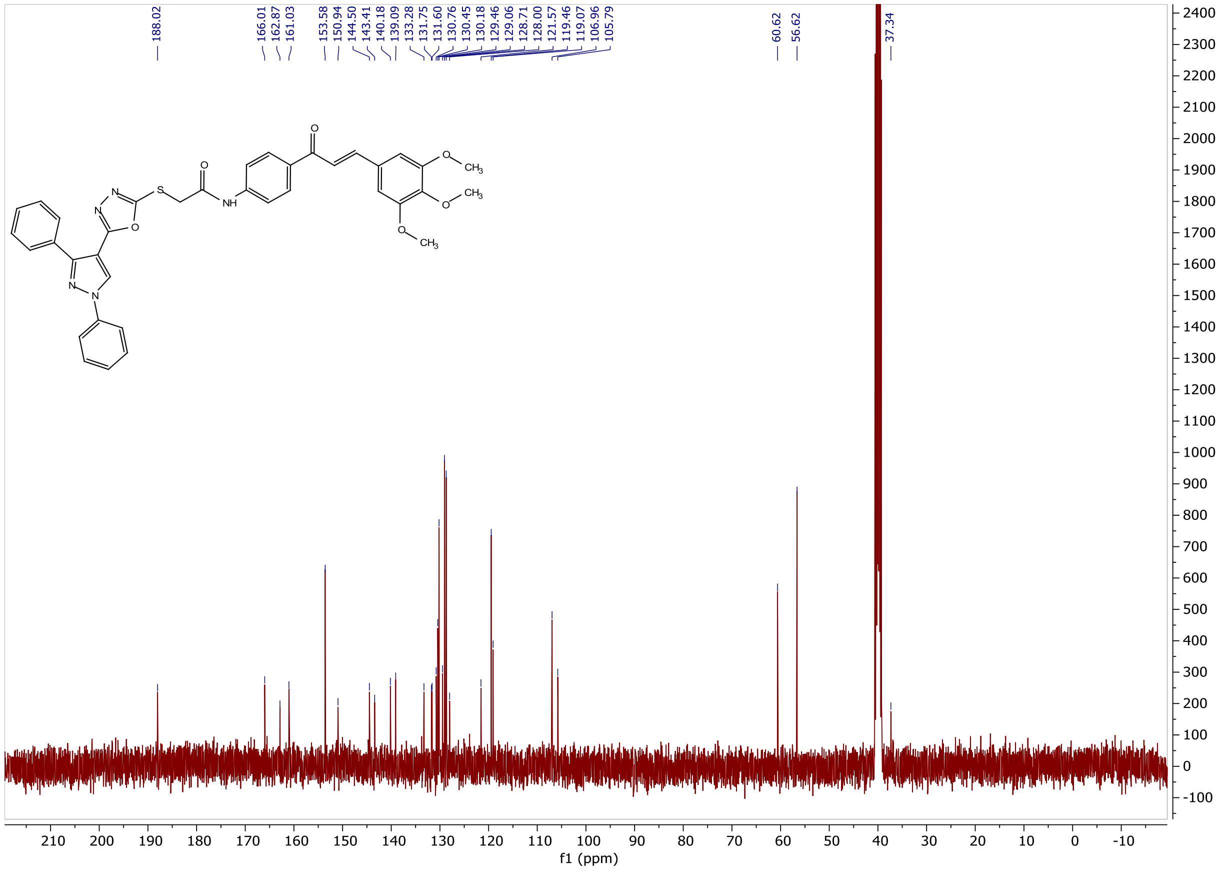

^{13}C NMR spectrum of compound **12d**

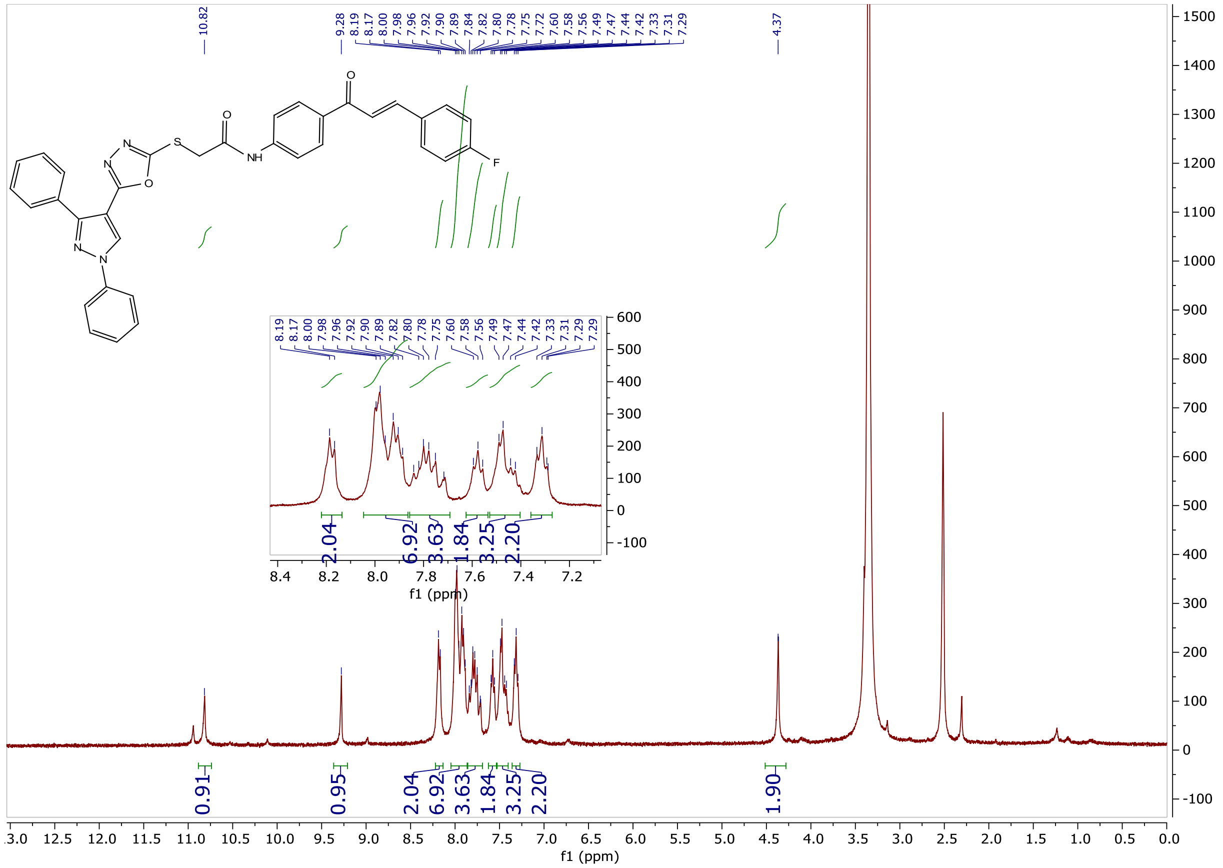

^{1}H NMR spectrum of compound **12e**

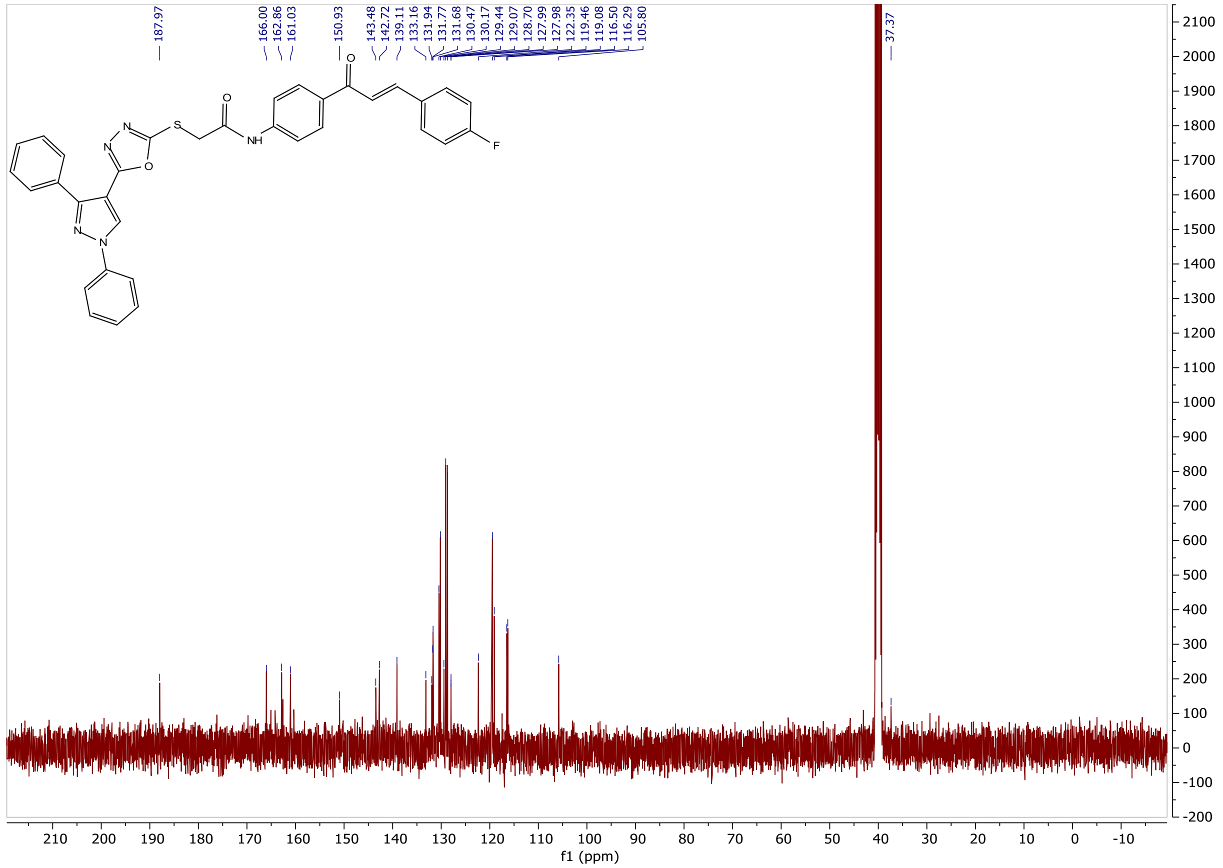

^{13}C NMR spectrum of compound **12e**

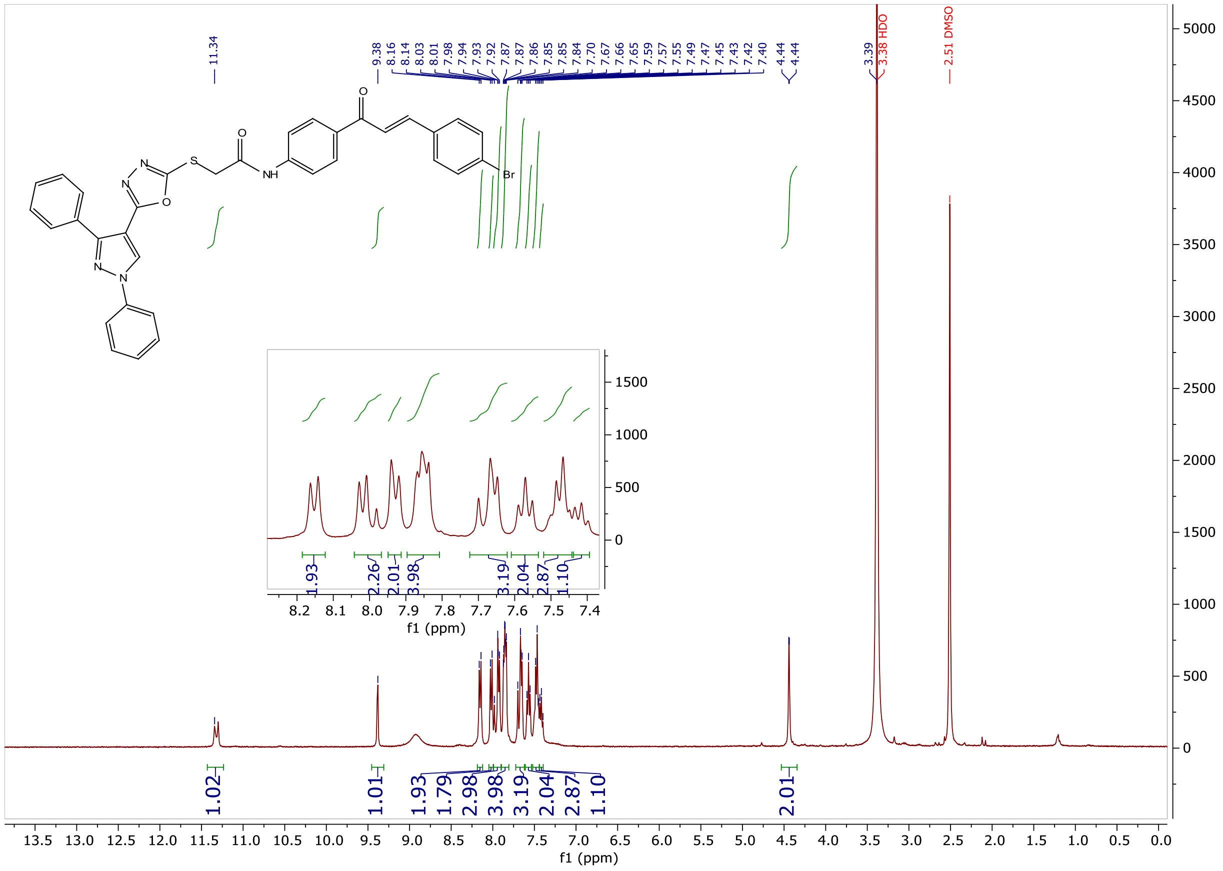

^{1}H NMR spectrum of compound **12f**

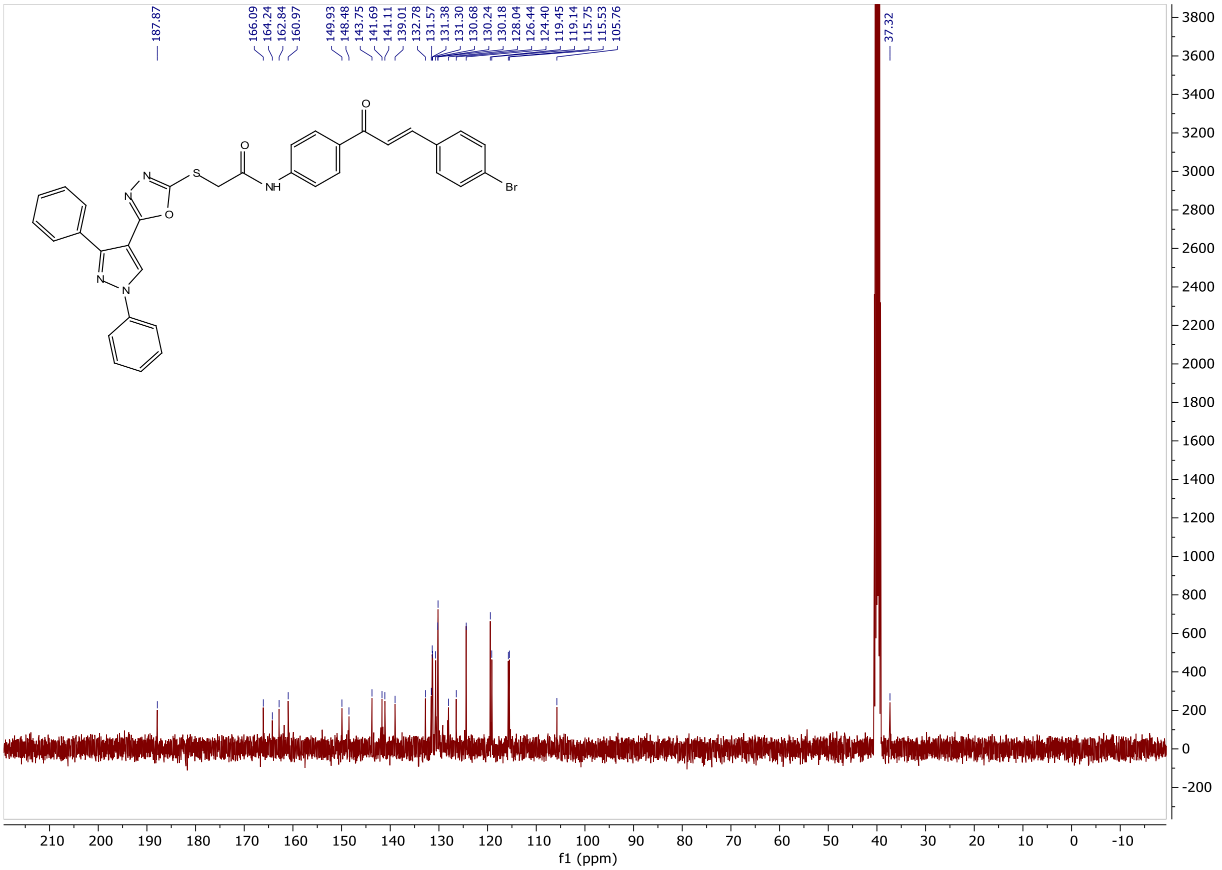

^{13}C NMR spectrum of compound **12f**

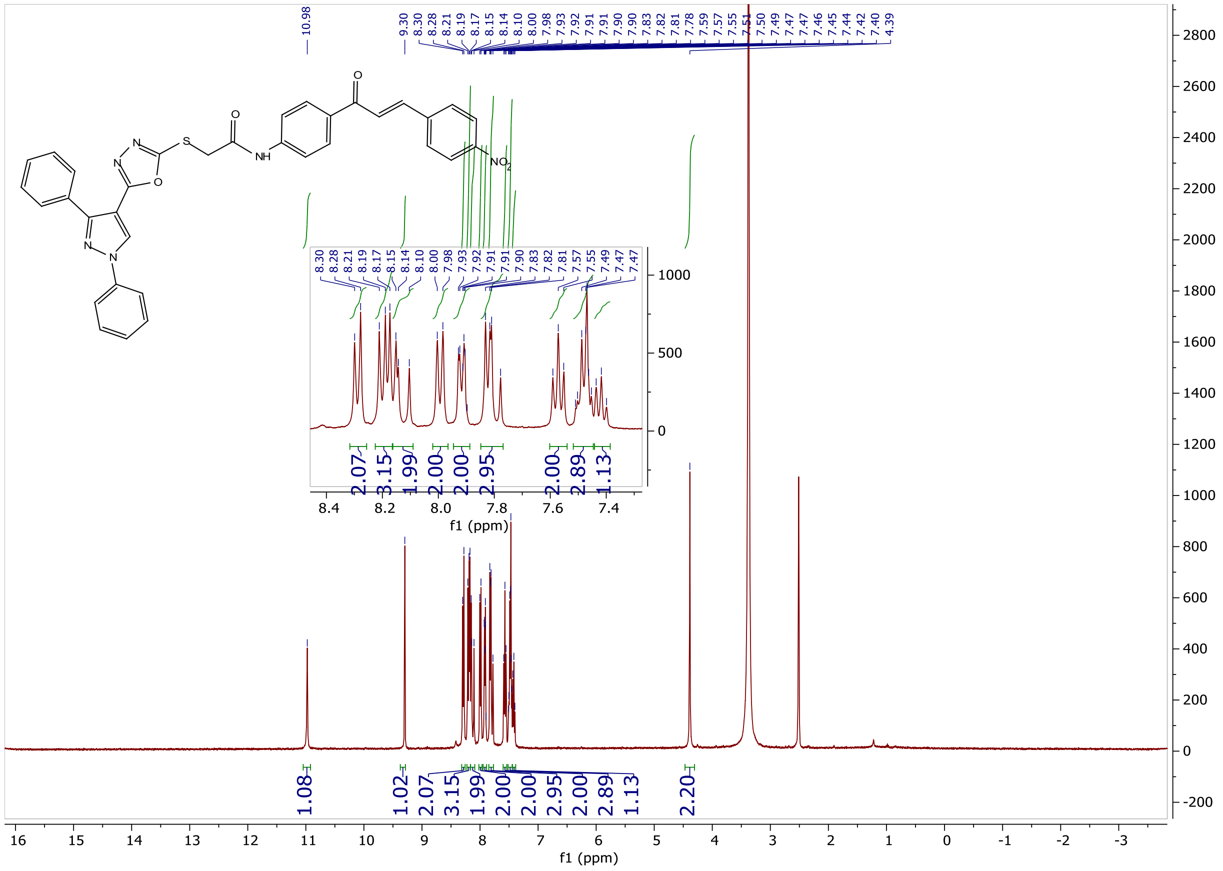

^{1}H NMR spectrum of compound **12g**

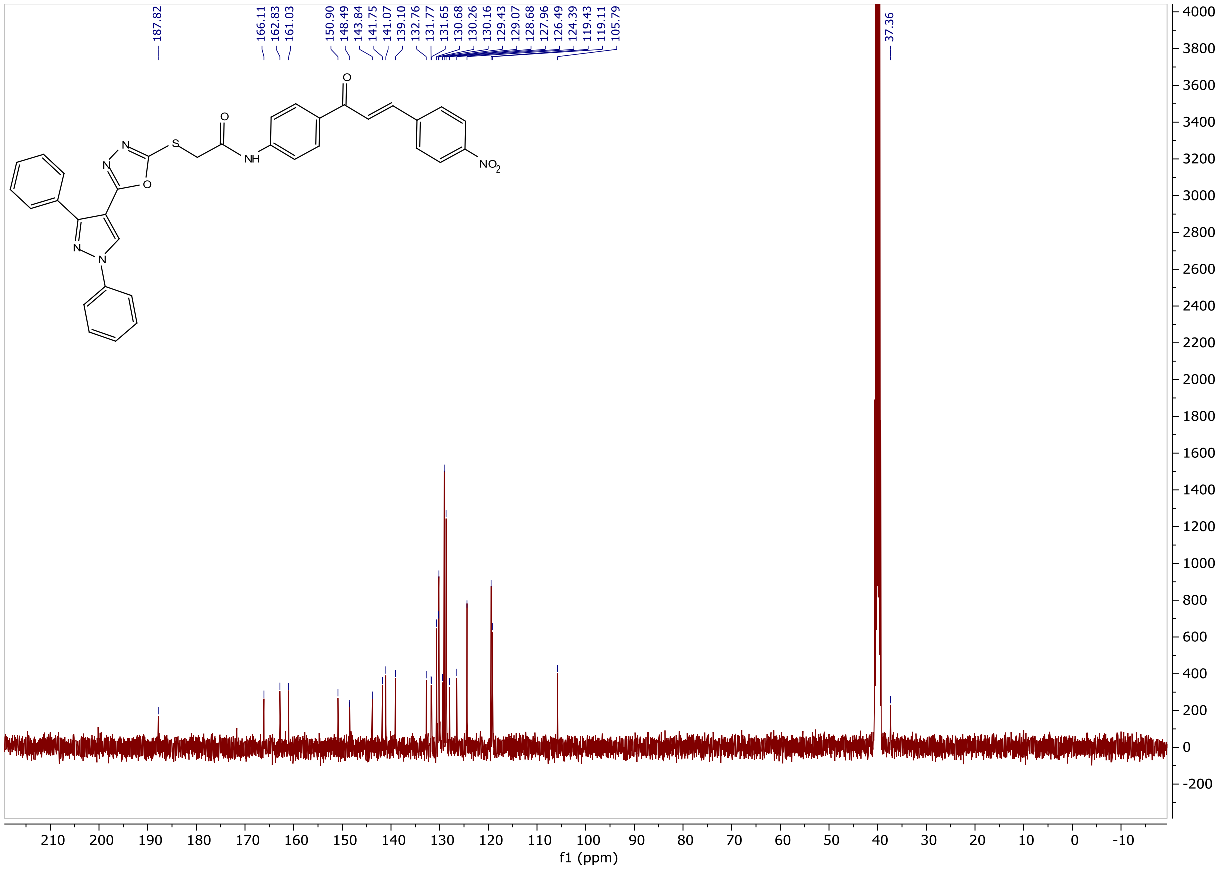

^{13}C NMR spectrum of compound **12g**

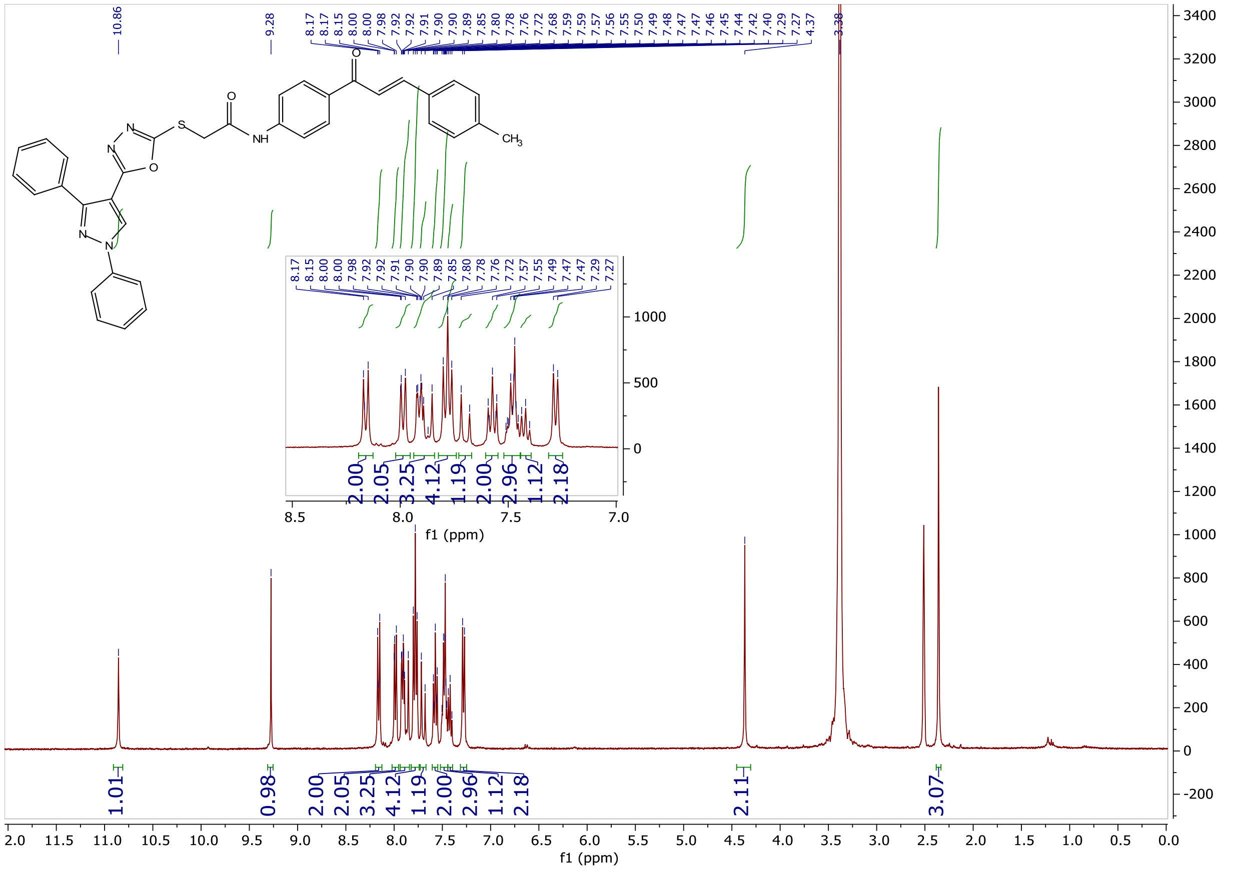

^{1}H NMR spectrum of compound **12h**

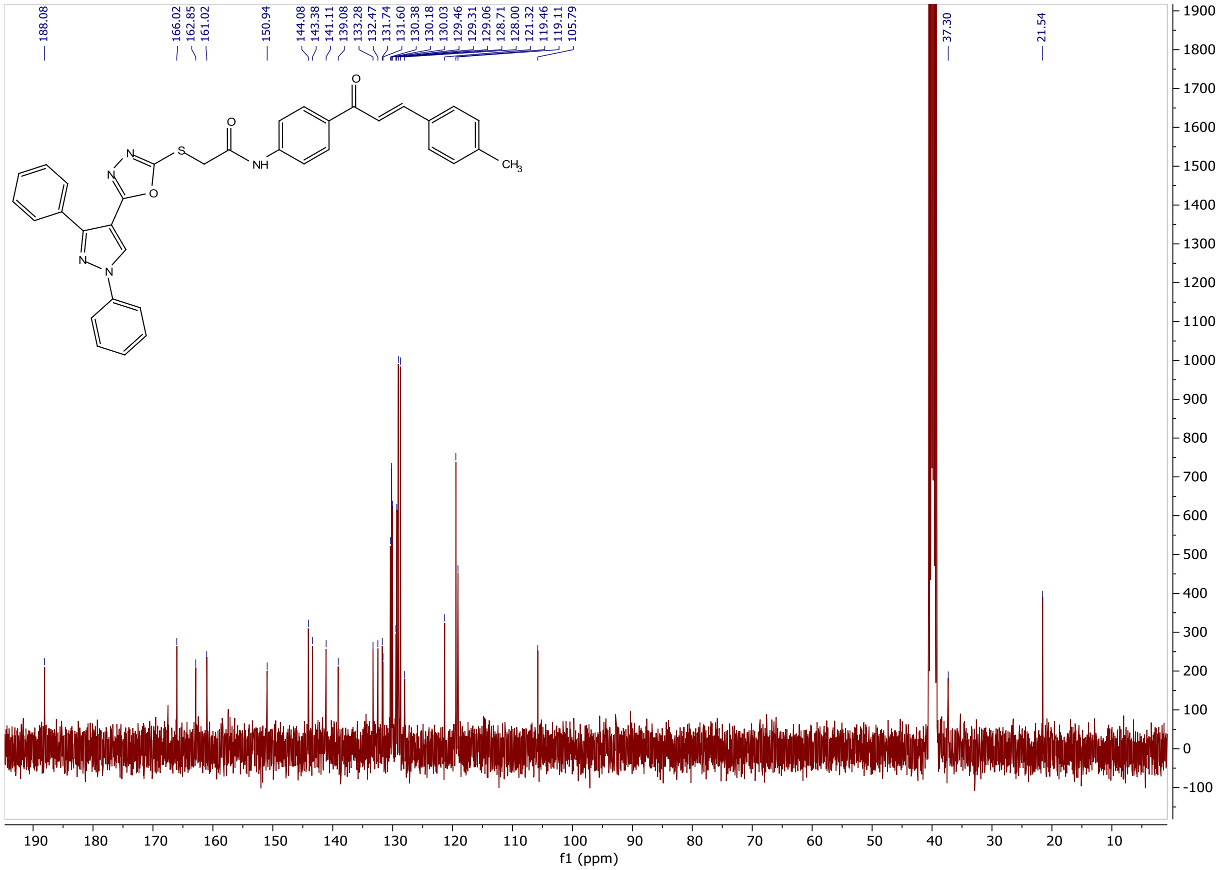

^{13}C NMR spectrum of compound **12h**

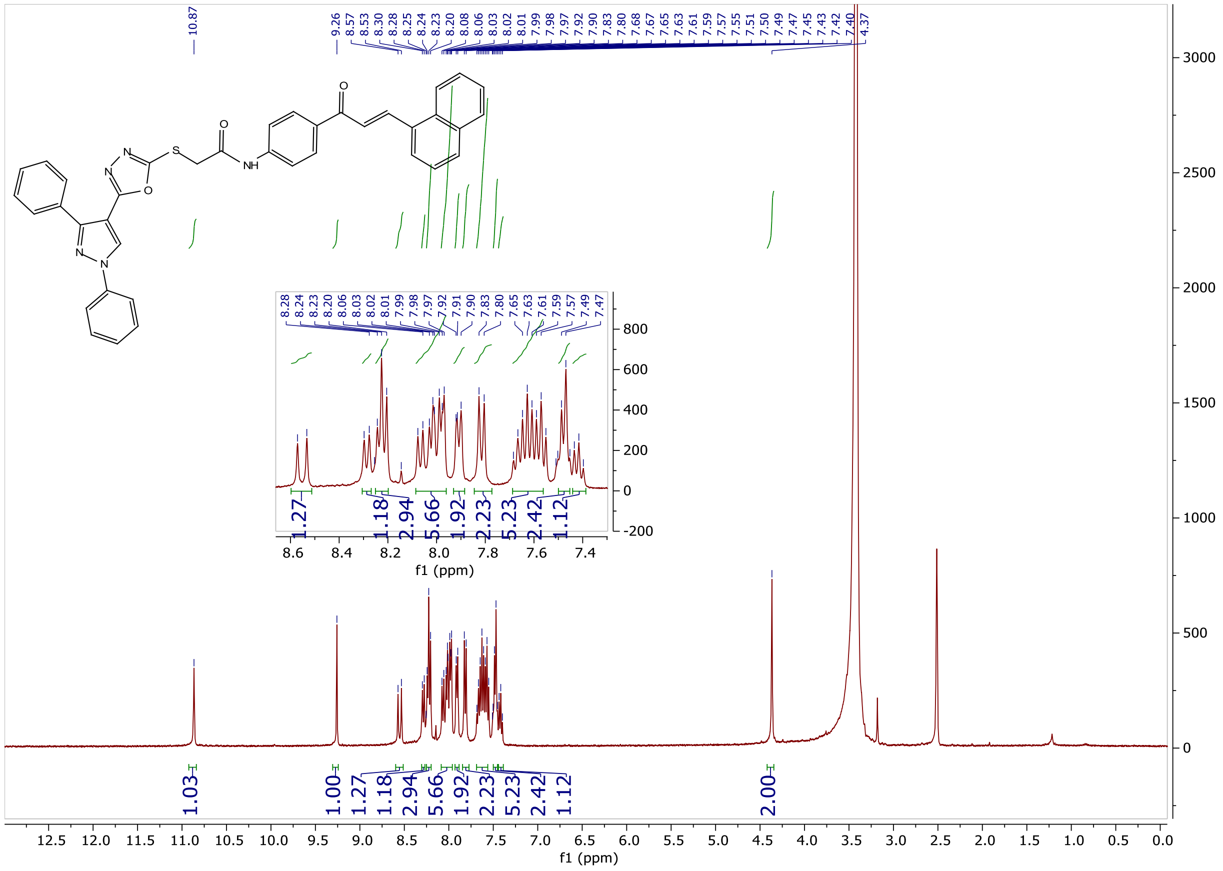

^{1}H NMR spectrum of compound **12i**

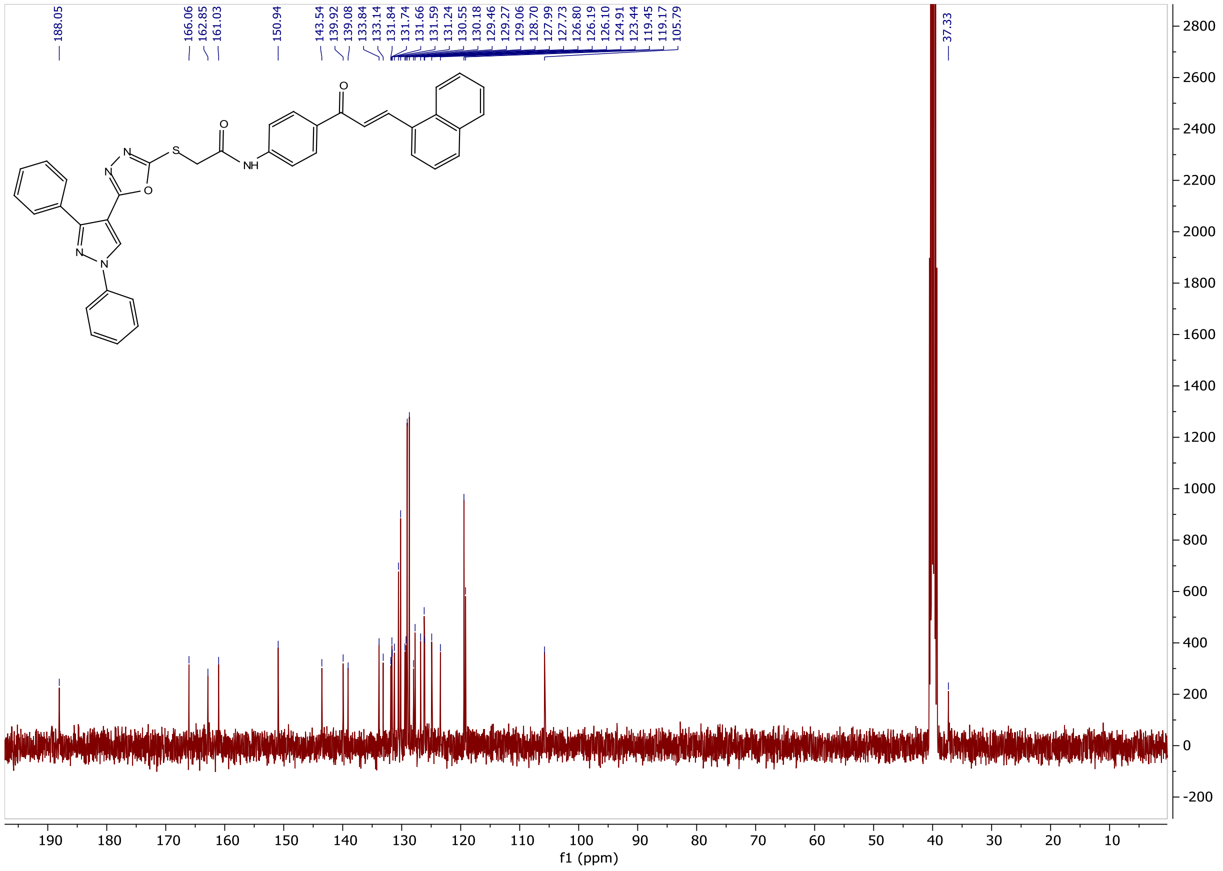

^{13}C NMR spectrum of compound **12i**

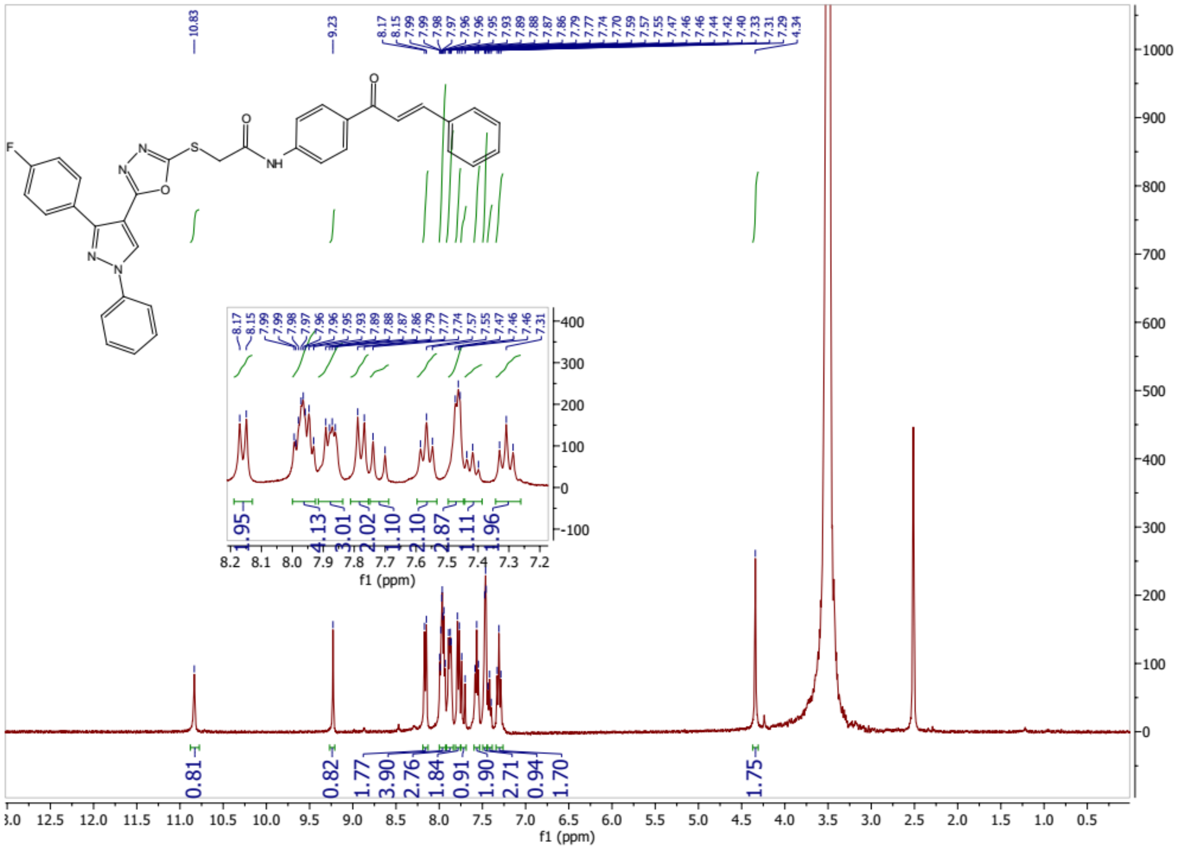

^{1}H NMR spectrum of compound **13a**

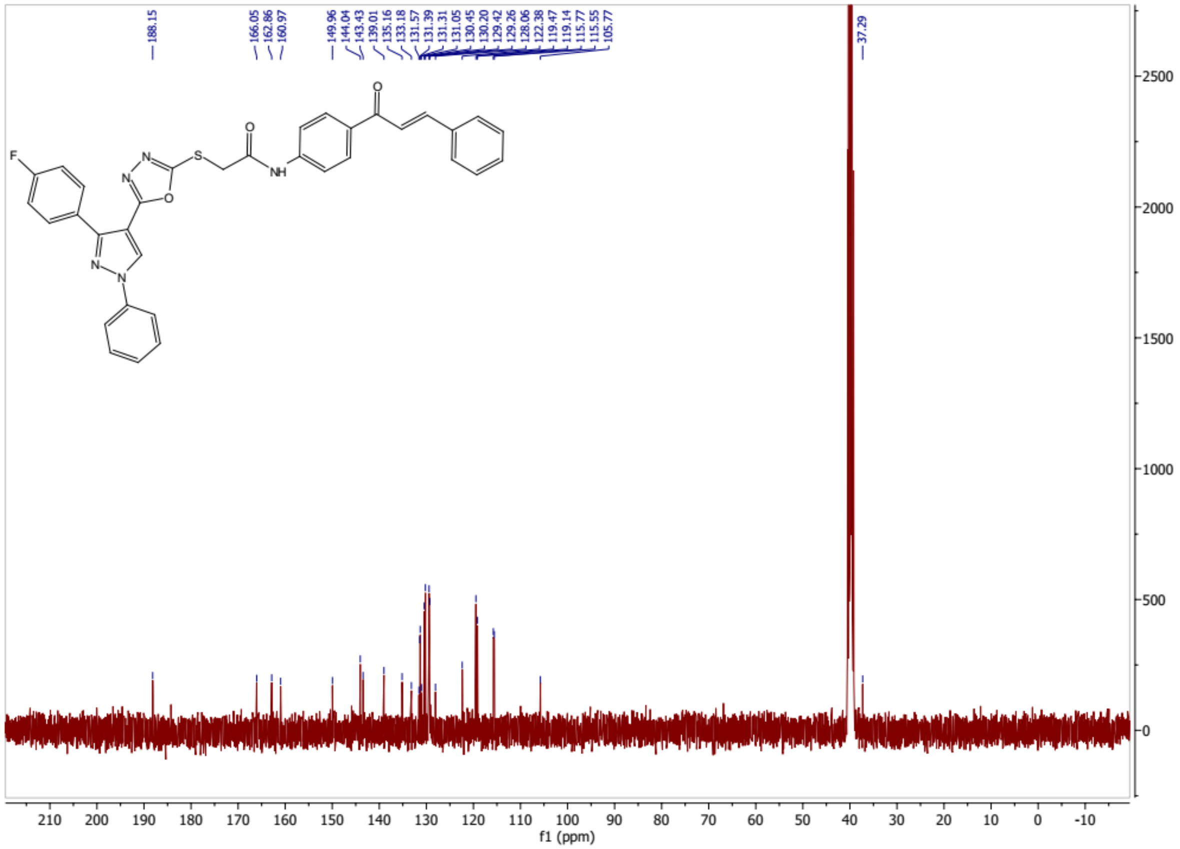

^{13}C NMR spectrum of compound **13a**

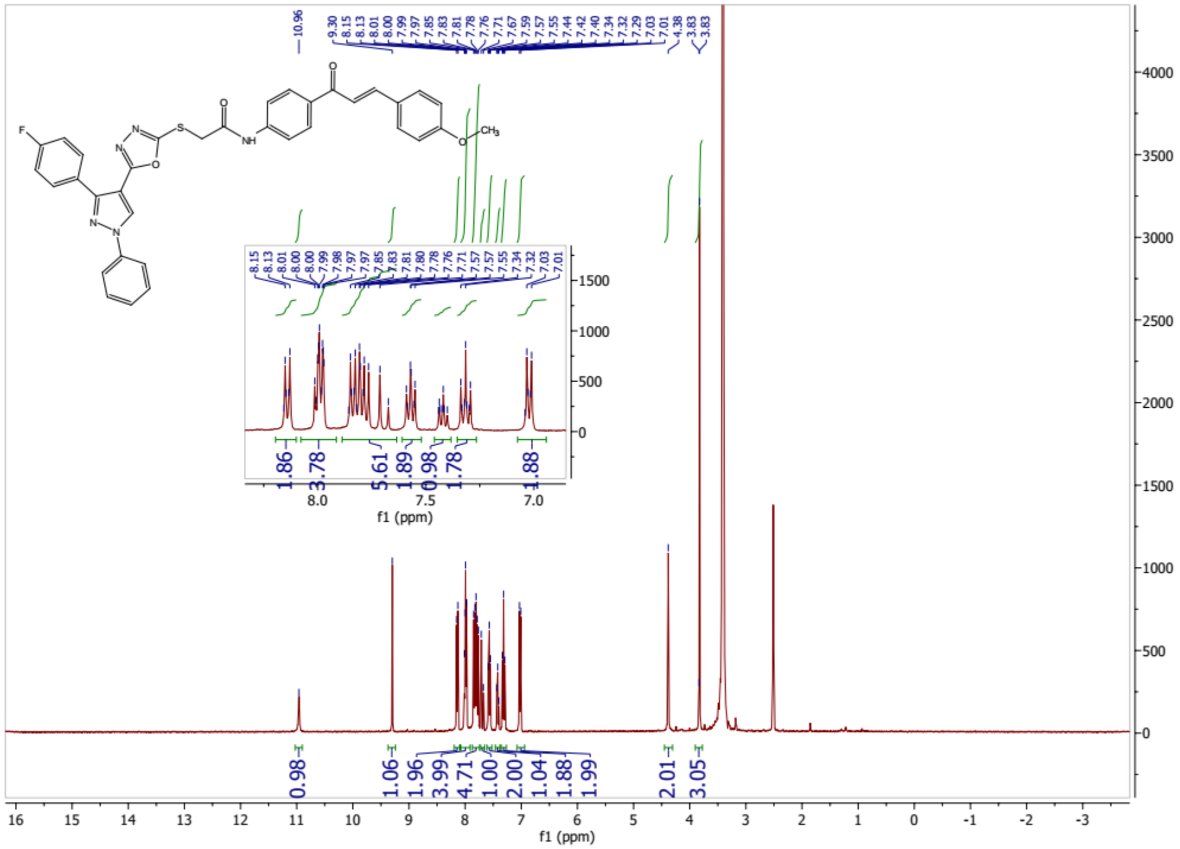

^{1}H NMR spectrum of compound **13b**

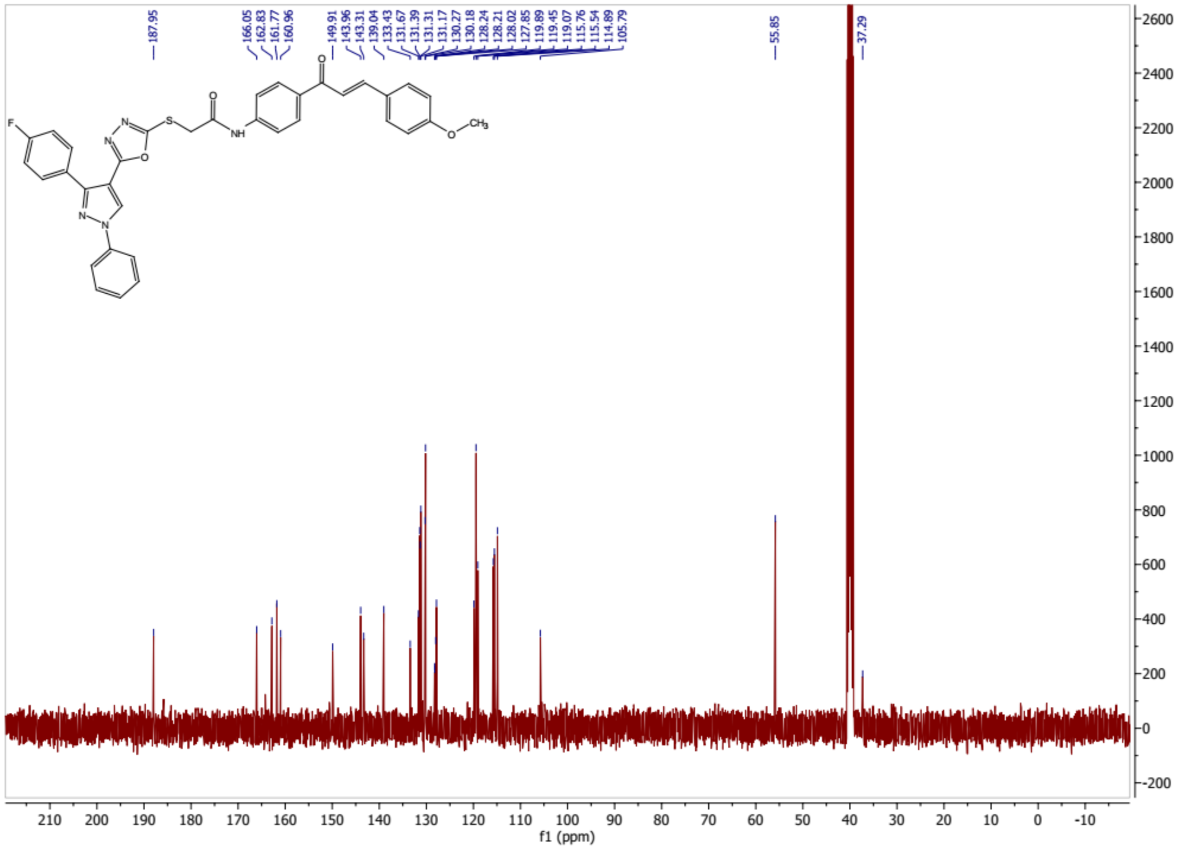

^{13}C NMR spectrum of compound **13b**

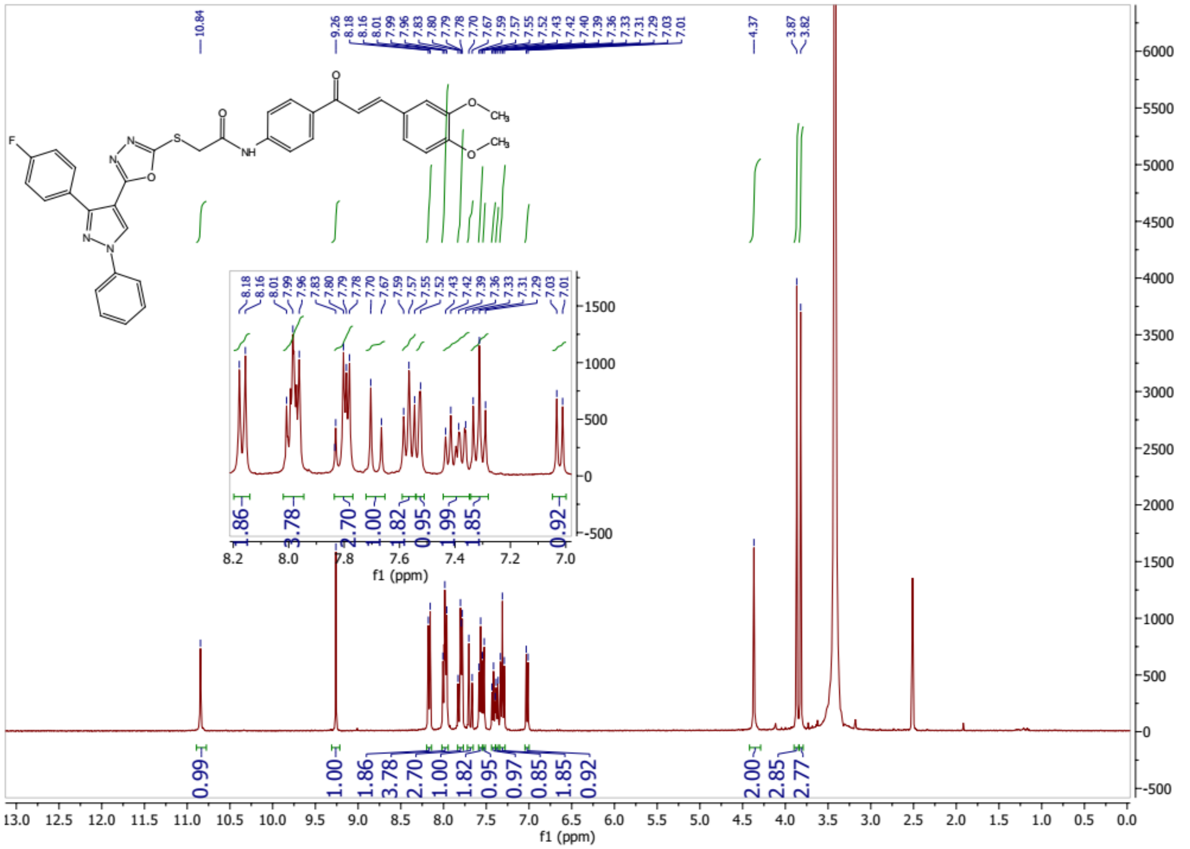

^{1}H NMR spectrum of compound **13c**

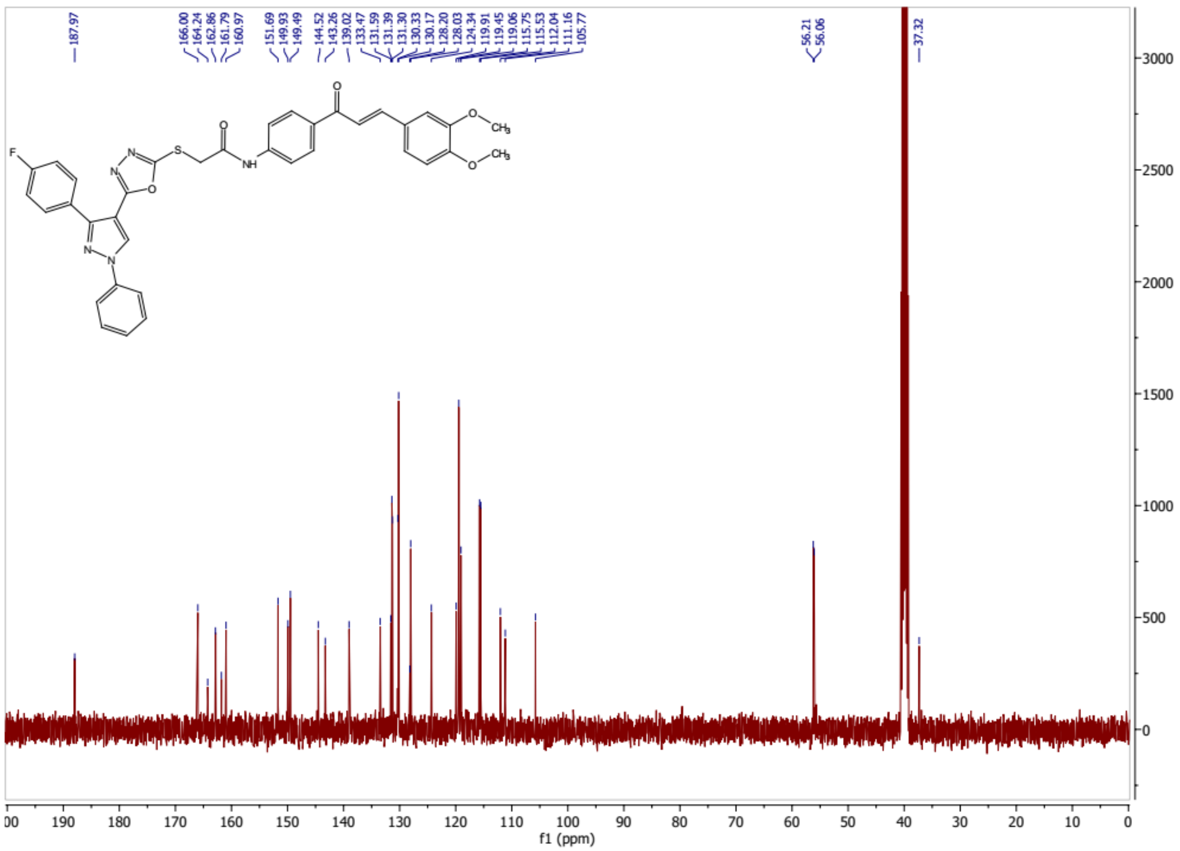

^{13}C NMR spectrum of compound **13c**

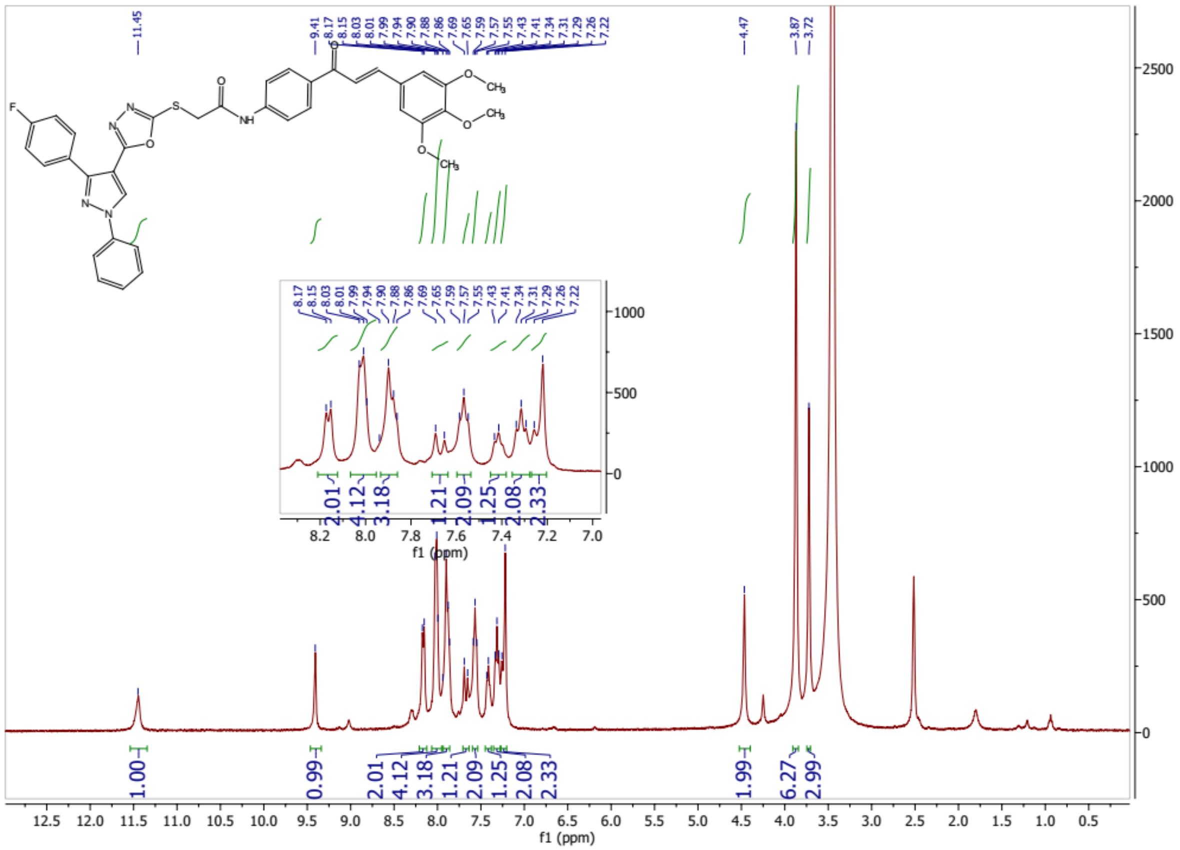

^{1}H NMR spectrum of compound **13d**

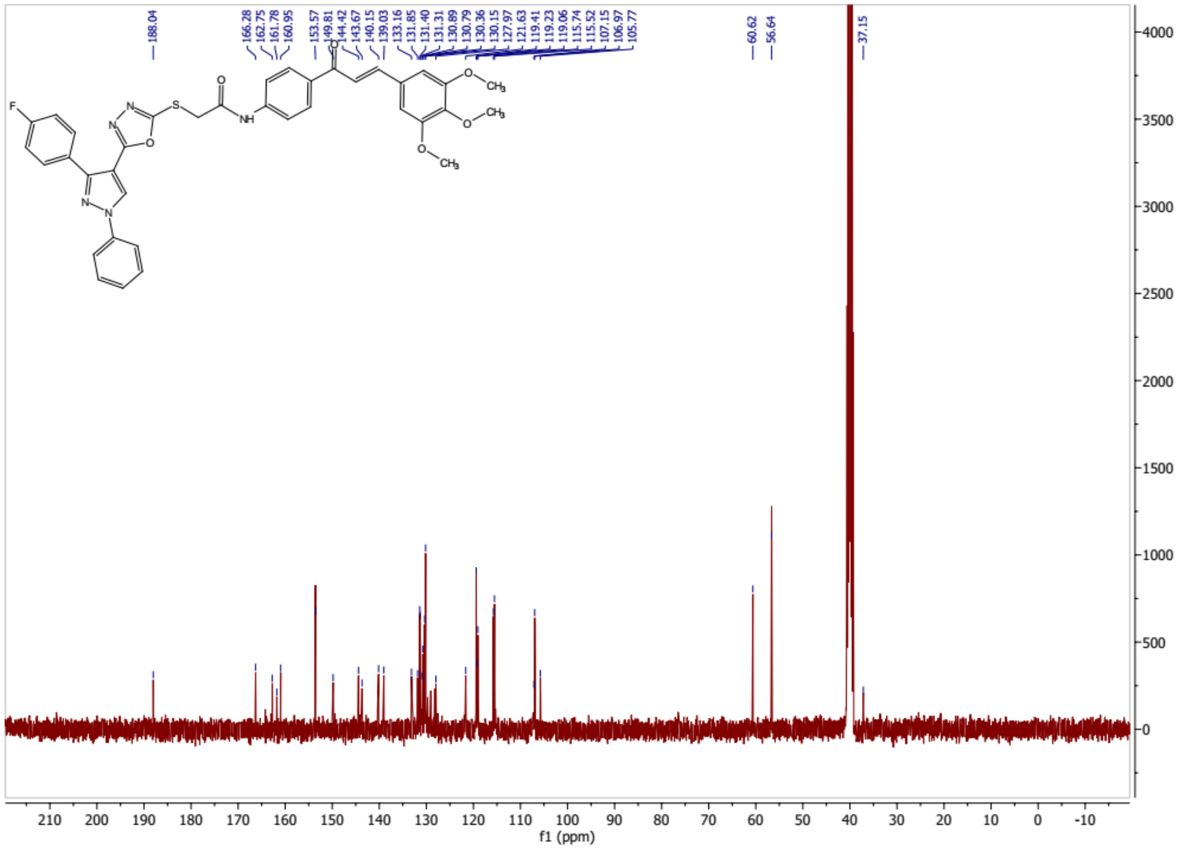

^{13}C NMR spectrum of compound **13d**

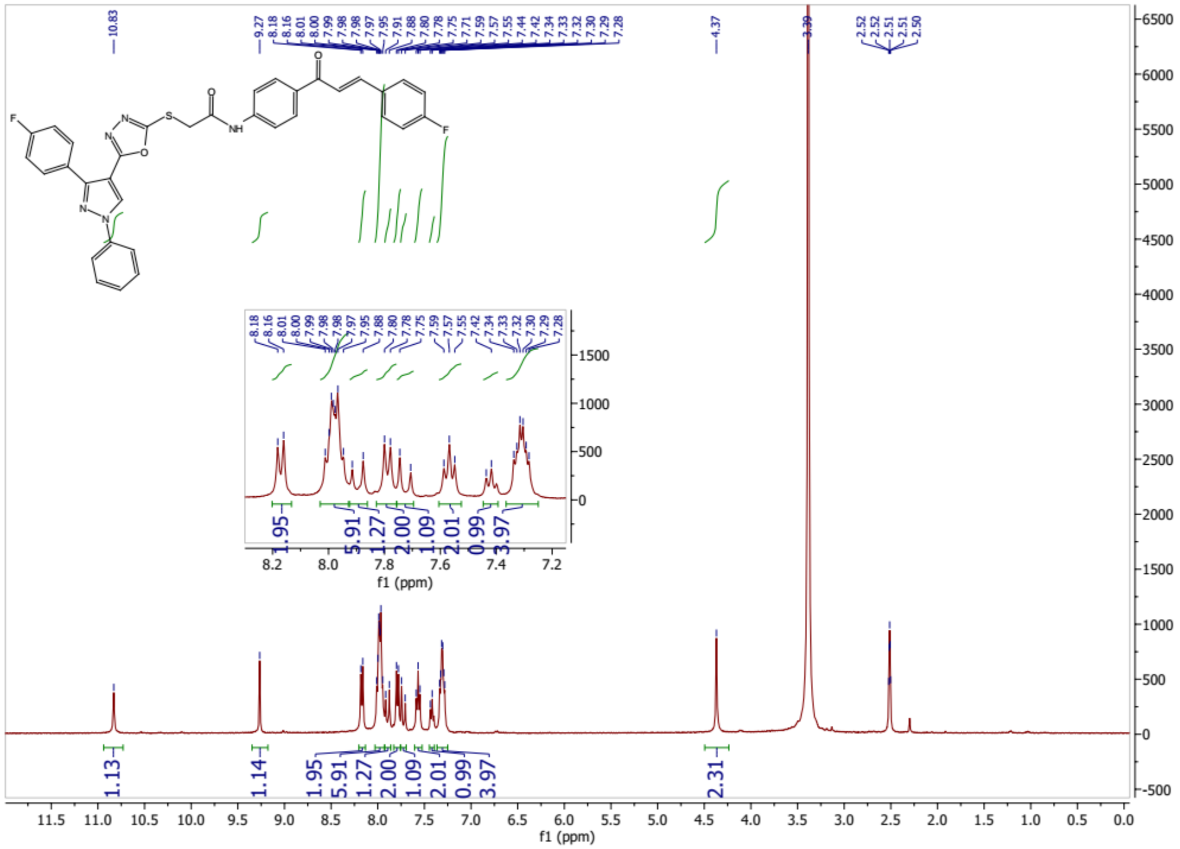

^{1}H NMR spectrum of compound **13e**

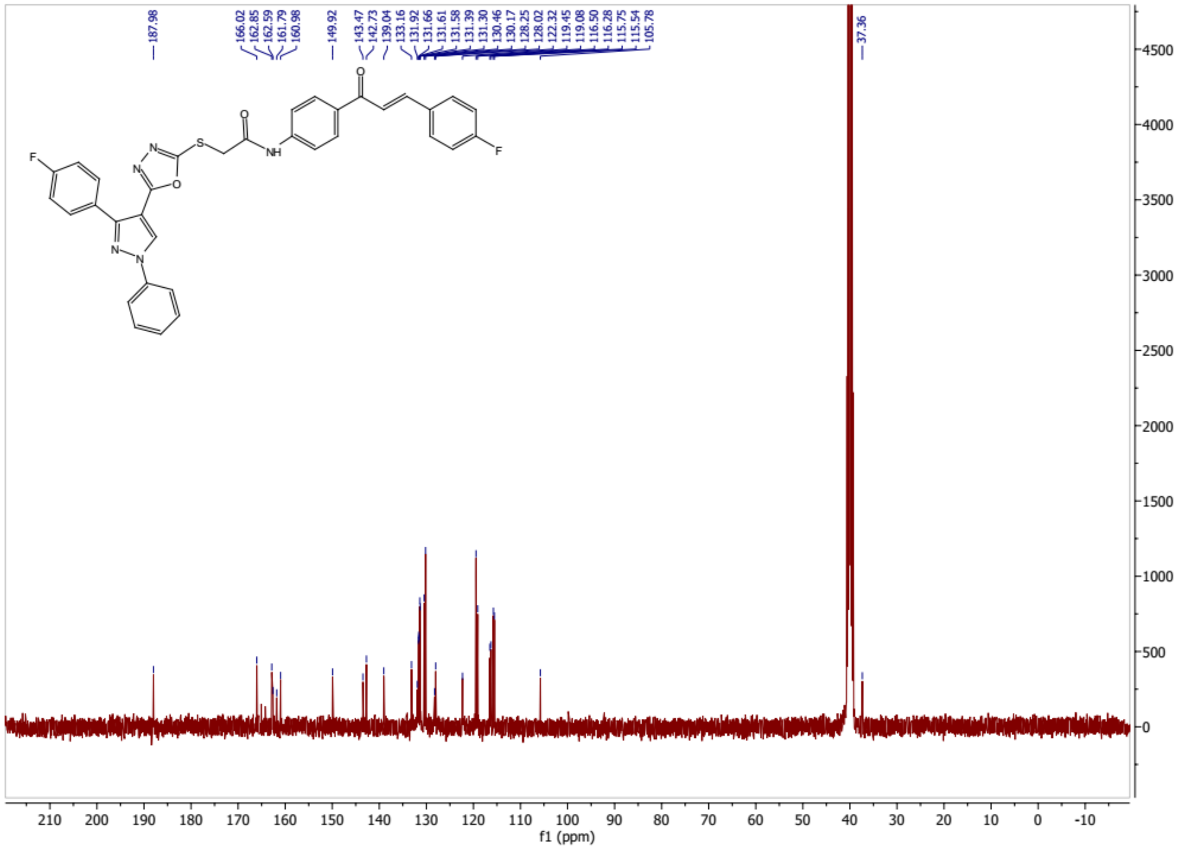

^{13}C NMR spectrum of compound **13e**

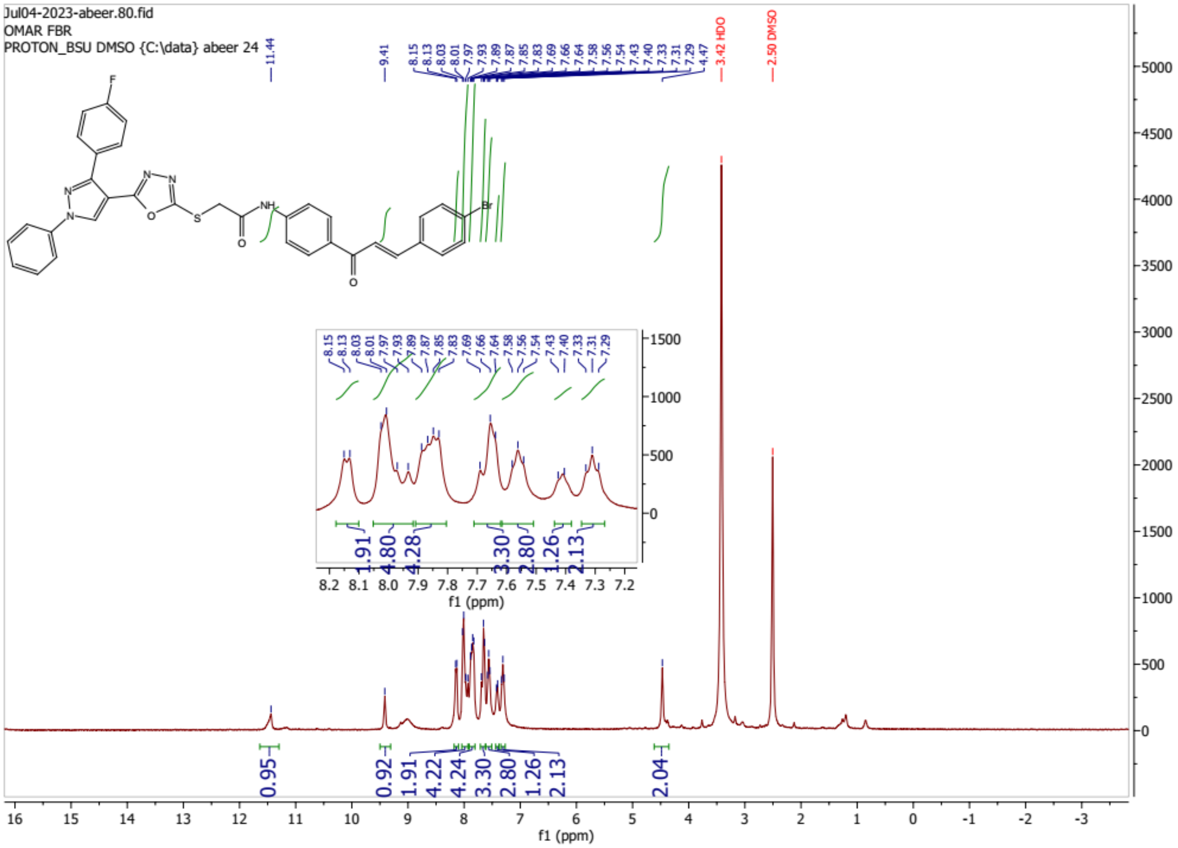

^{1}H NMR spectrum of compound **13f**

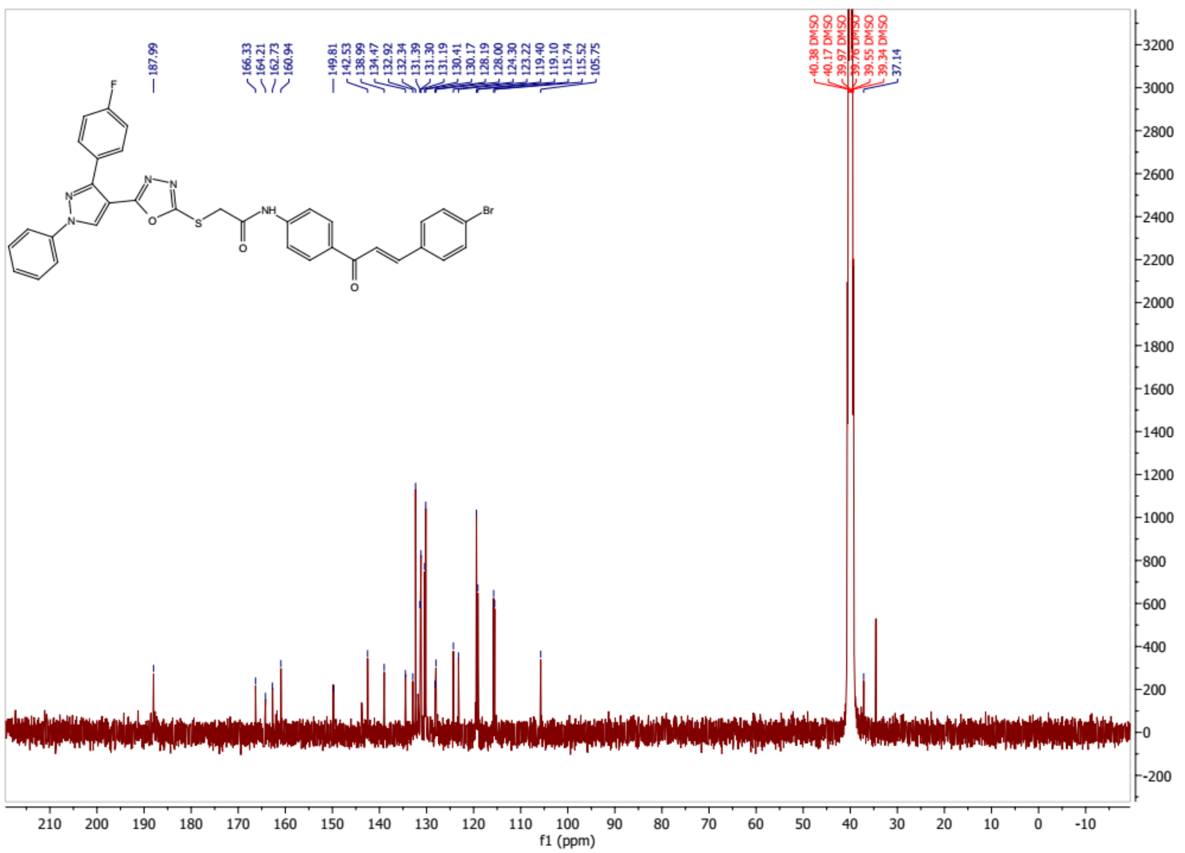

^{13}C NMR spectrum of compound **13f**

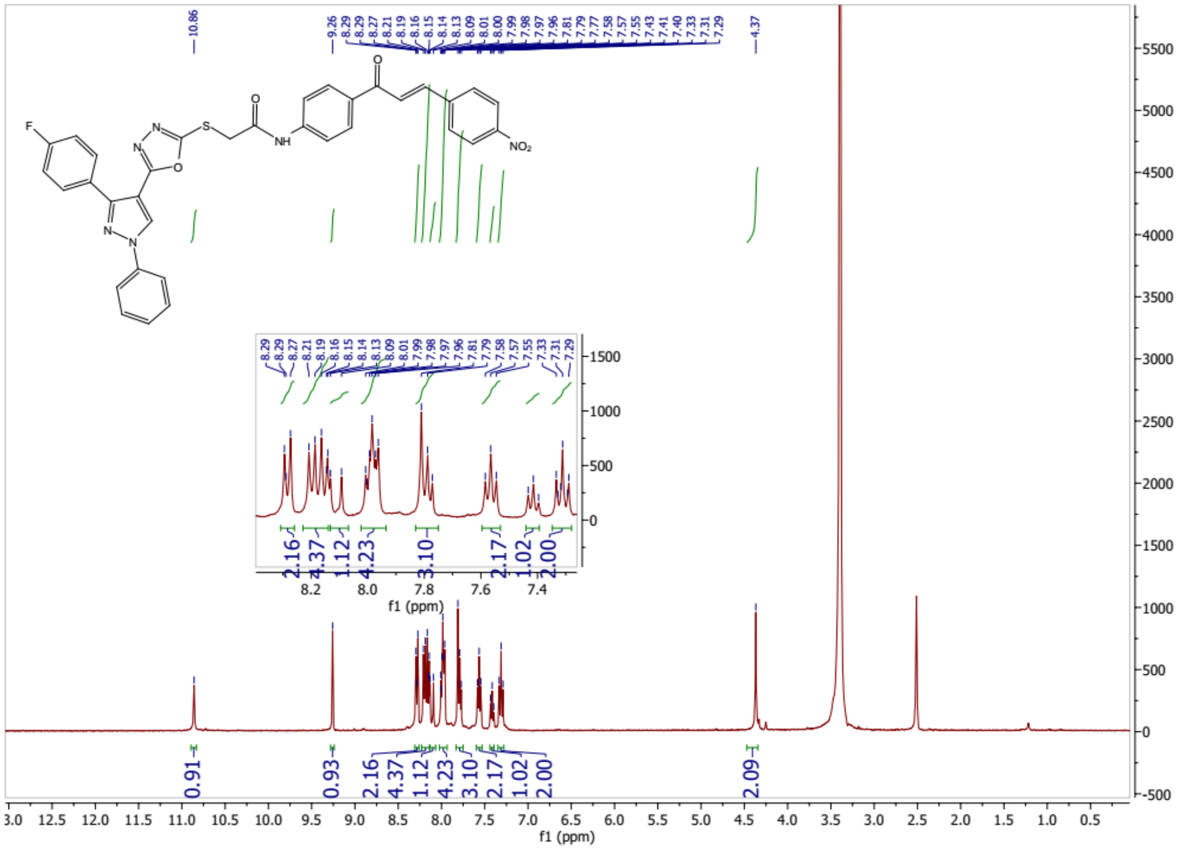

^{1}H NMR spectrum of compound **13g**

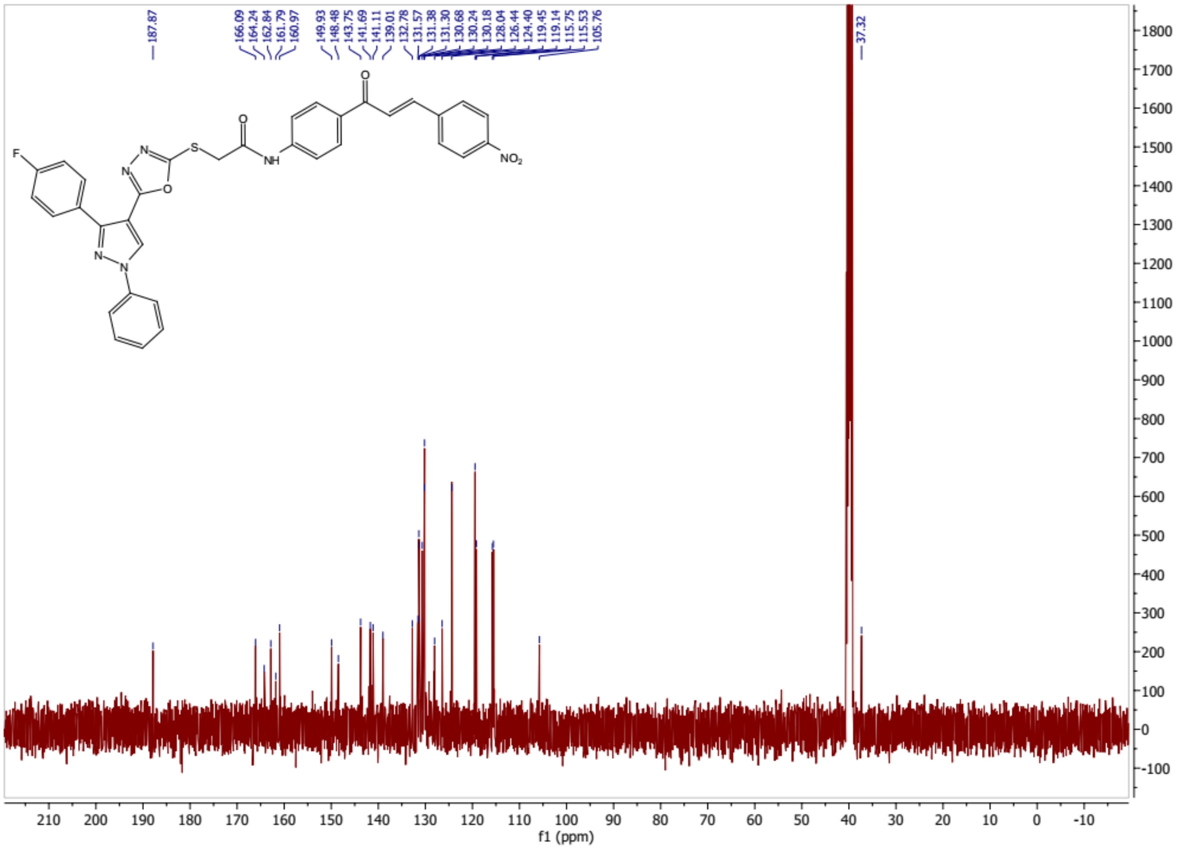

^{13}C NMR spectrum of compound **13g**

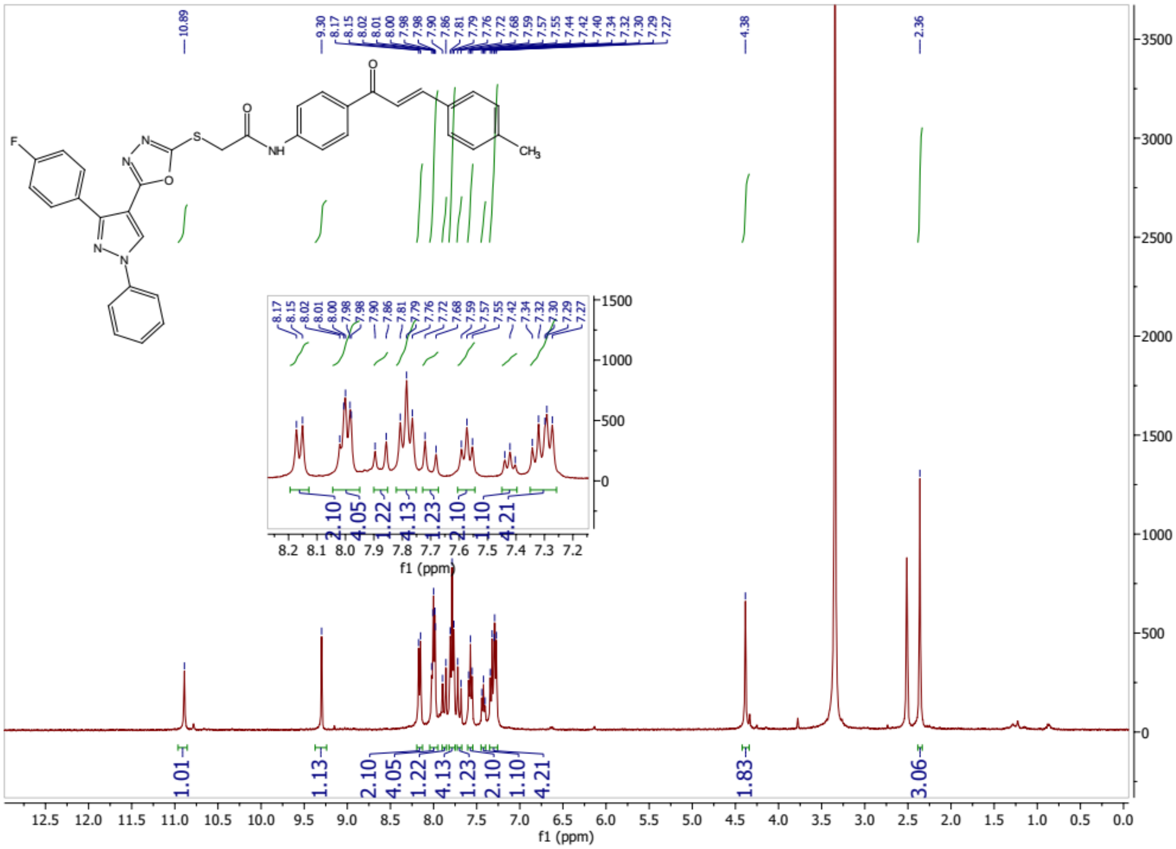

^{1}H NMR spectrum of compound **13h**

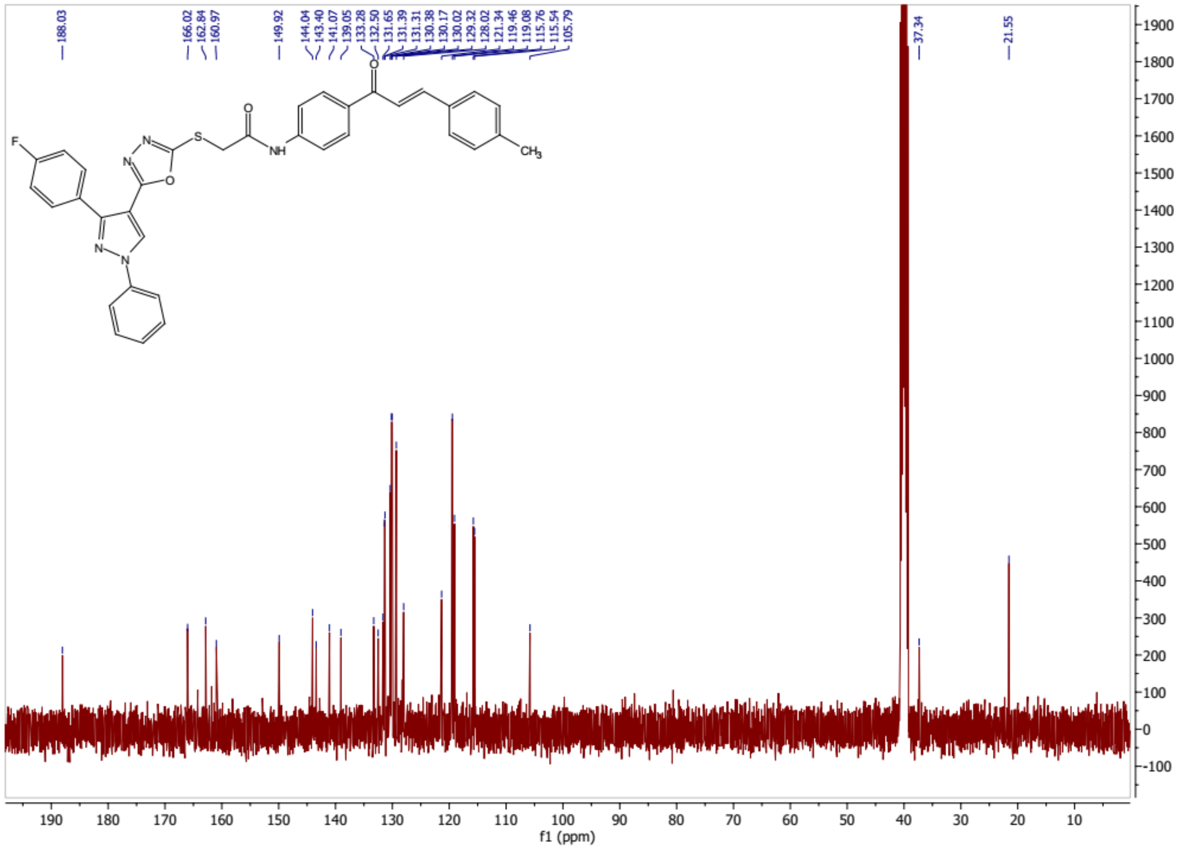

^{13}C NMR spectrum of compound **13h**

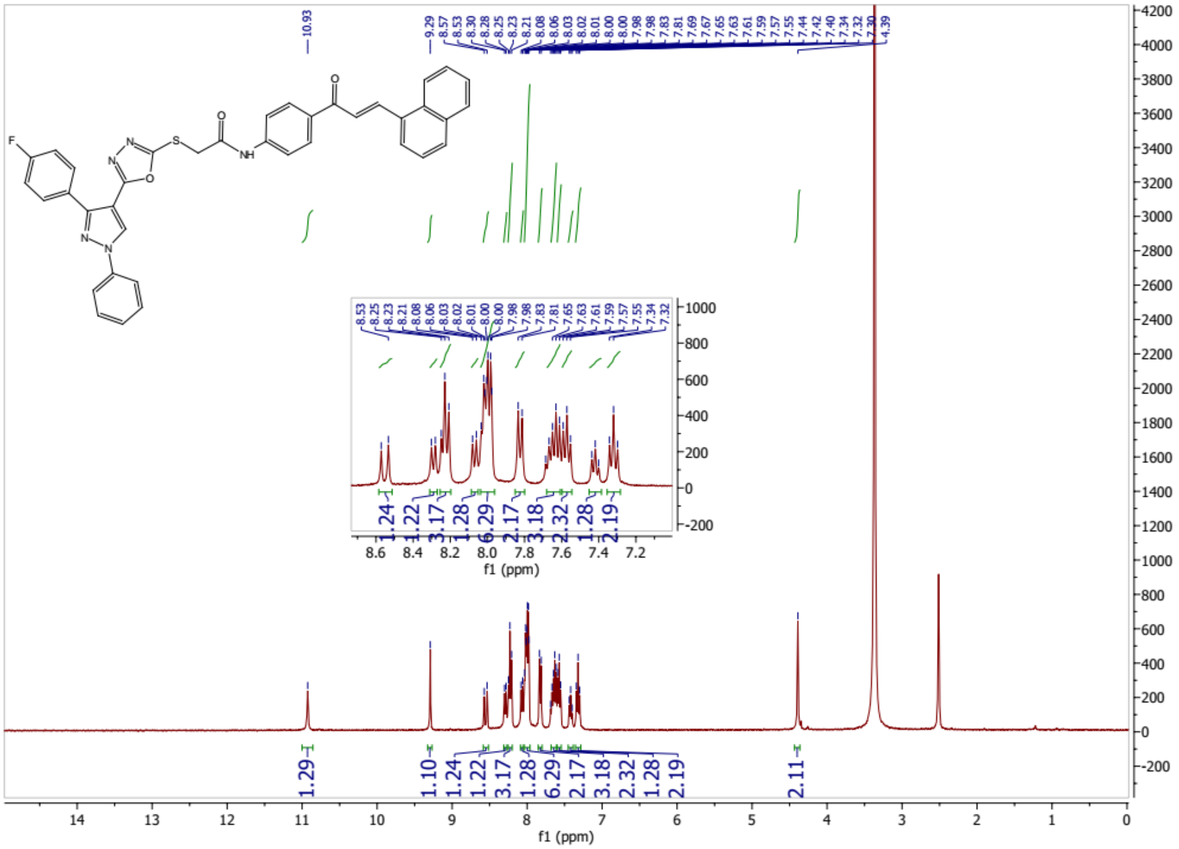

^{1}H NMR spectrum of compound **13i**

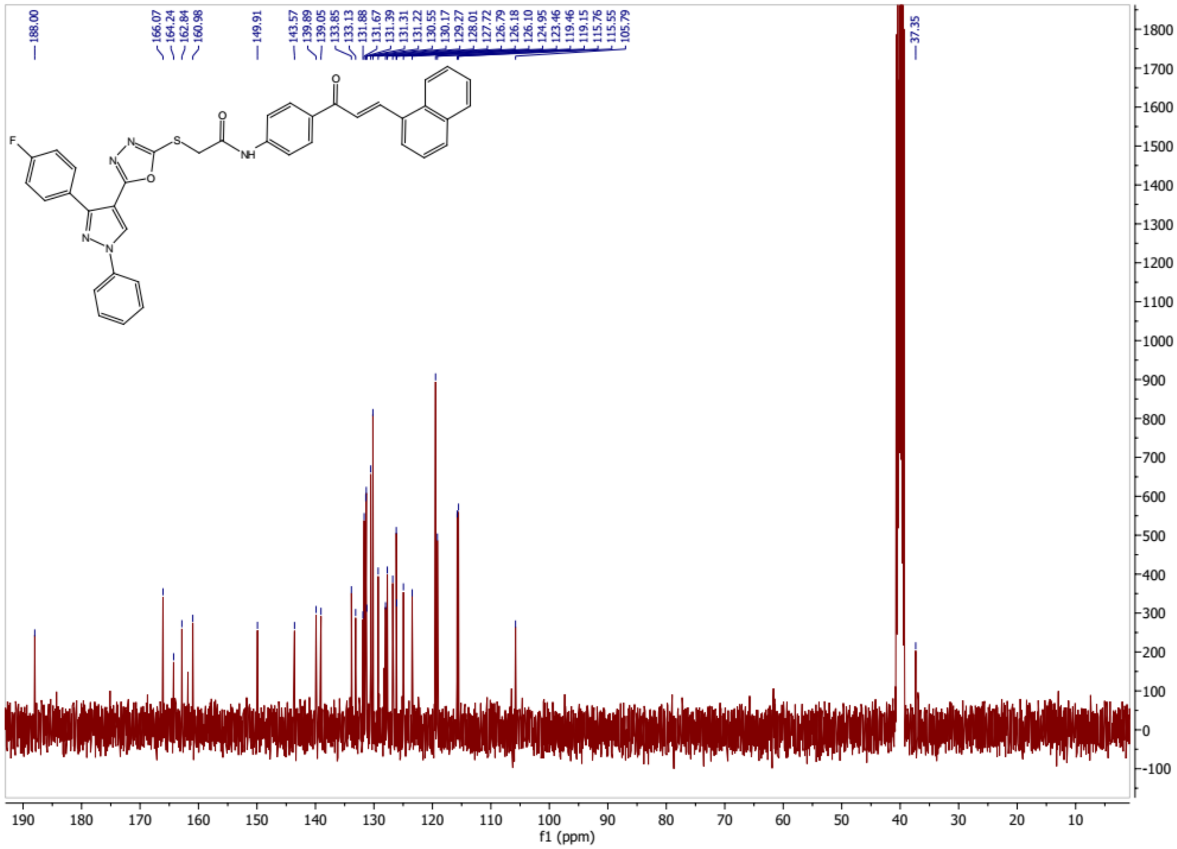

^{13}C NMR spectrum of compound **13i**

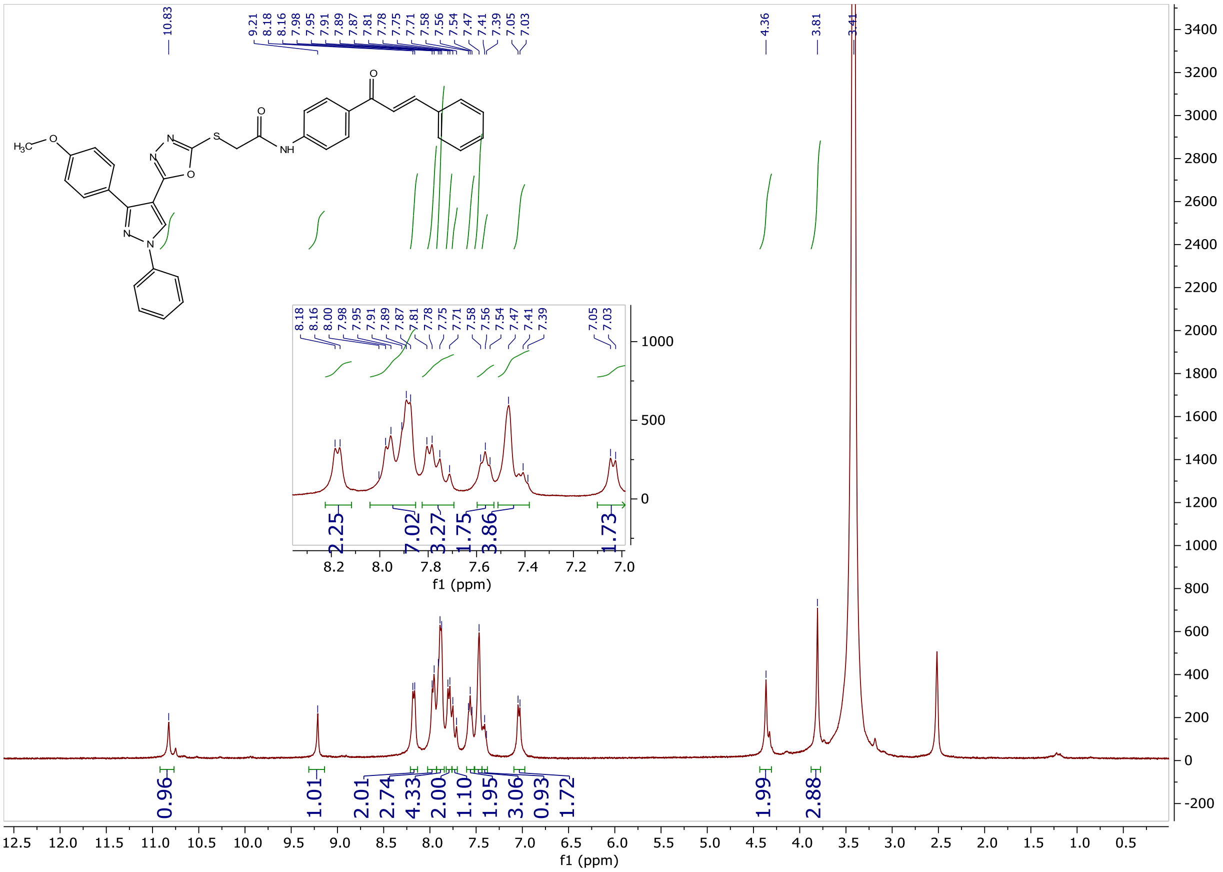

^{1}H NMR spectrum of compound **14a**

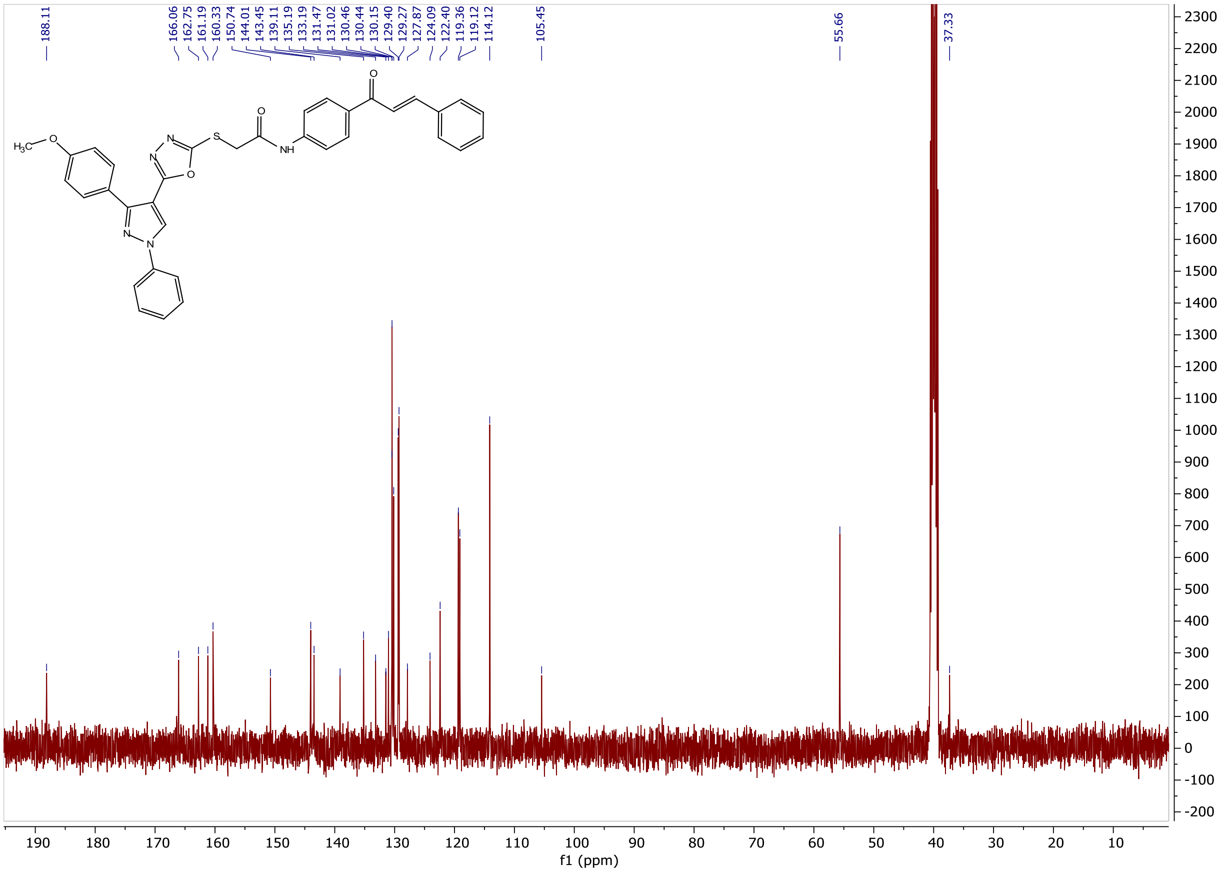

^{13}C NMR spectrum of compound **14a**

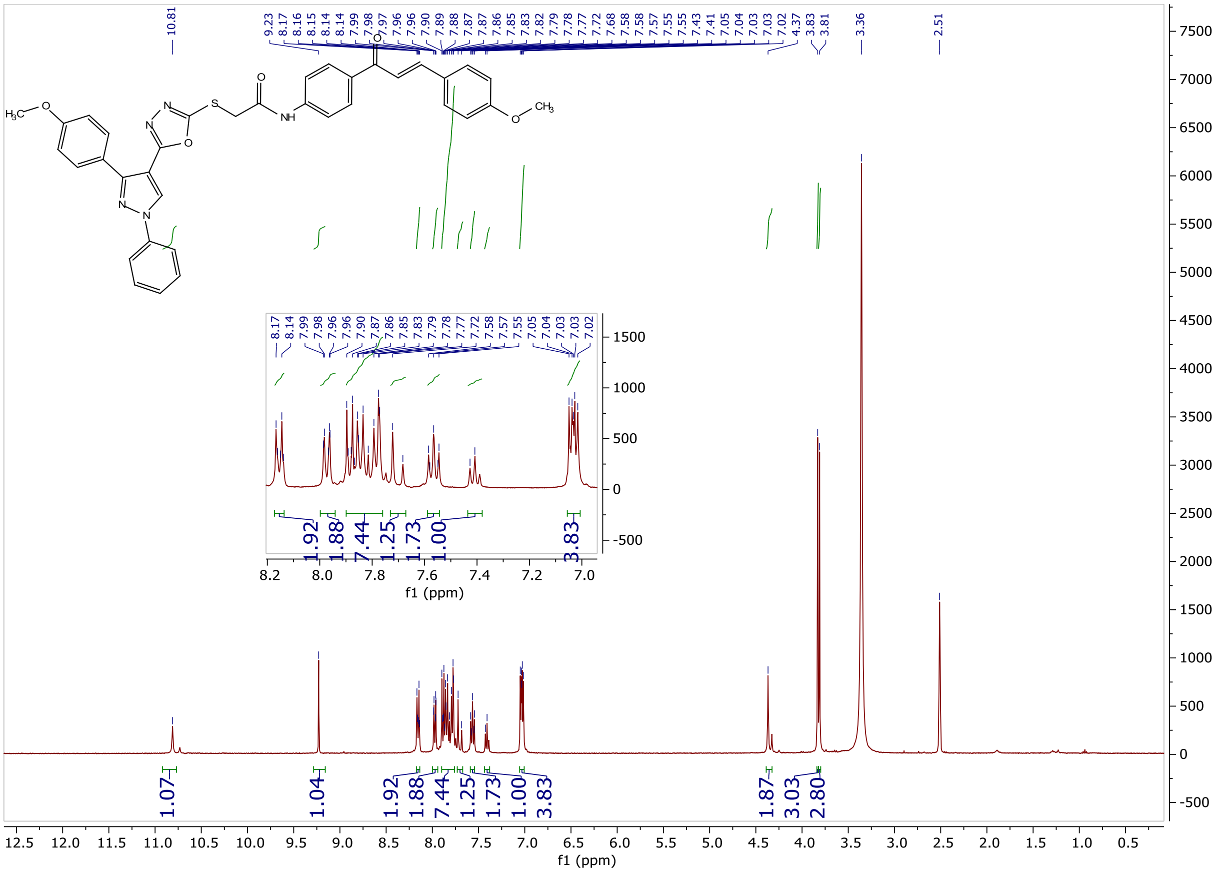

^{1}H NMR spectrum of compound **14b**

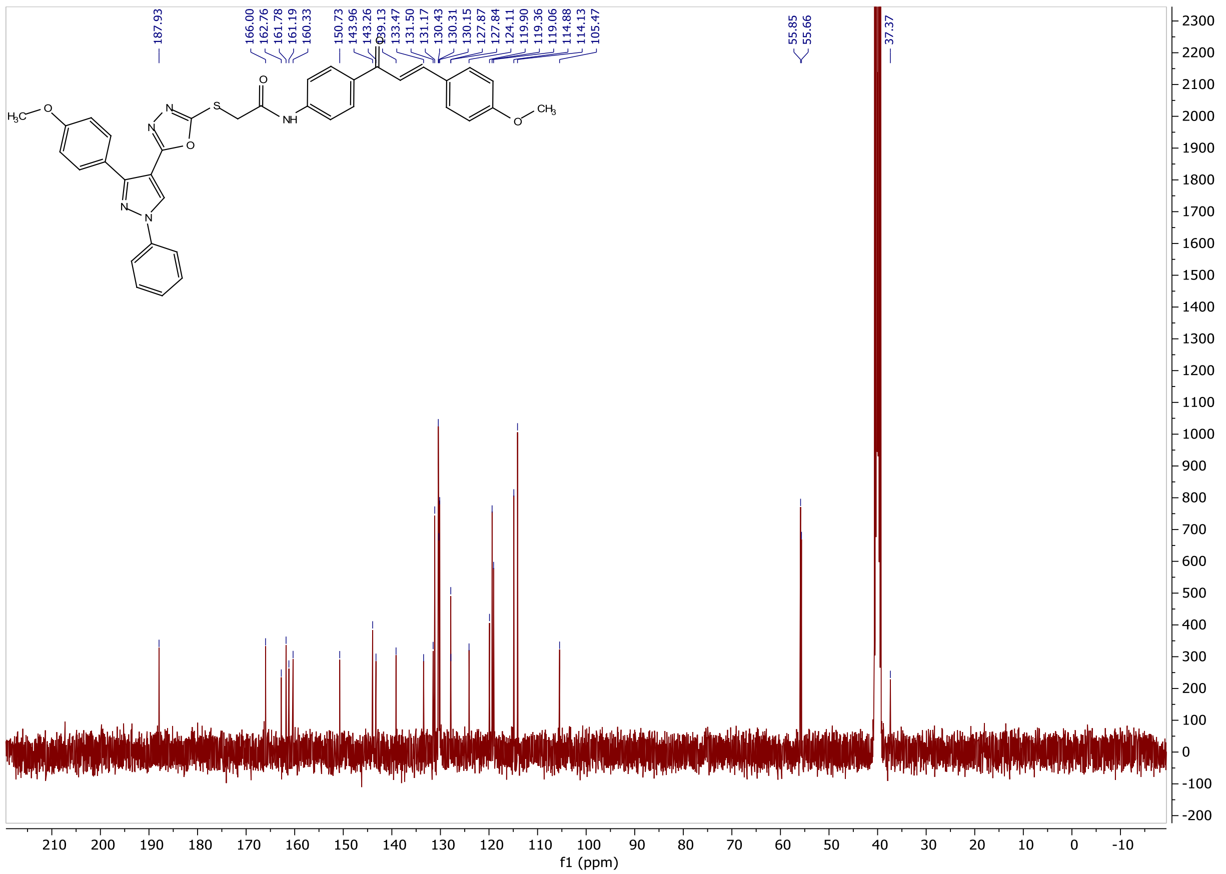

^{13}C NMR spectrum of compound **14b**

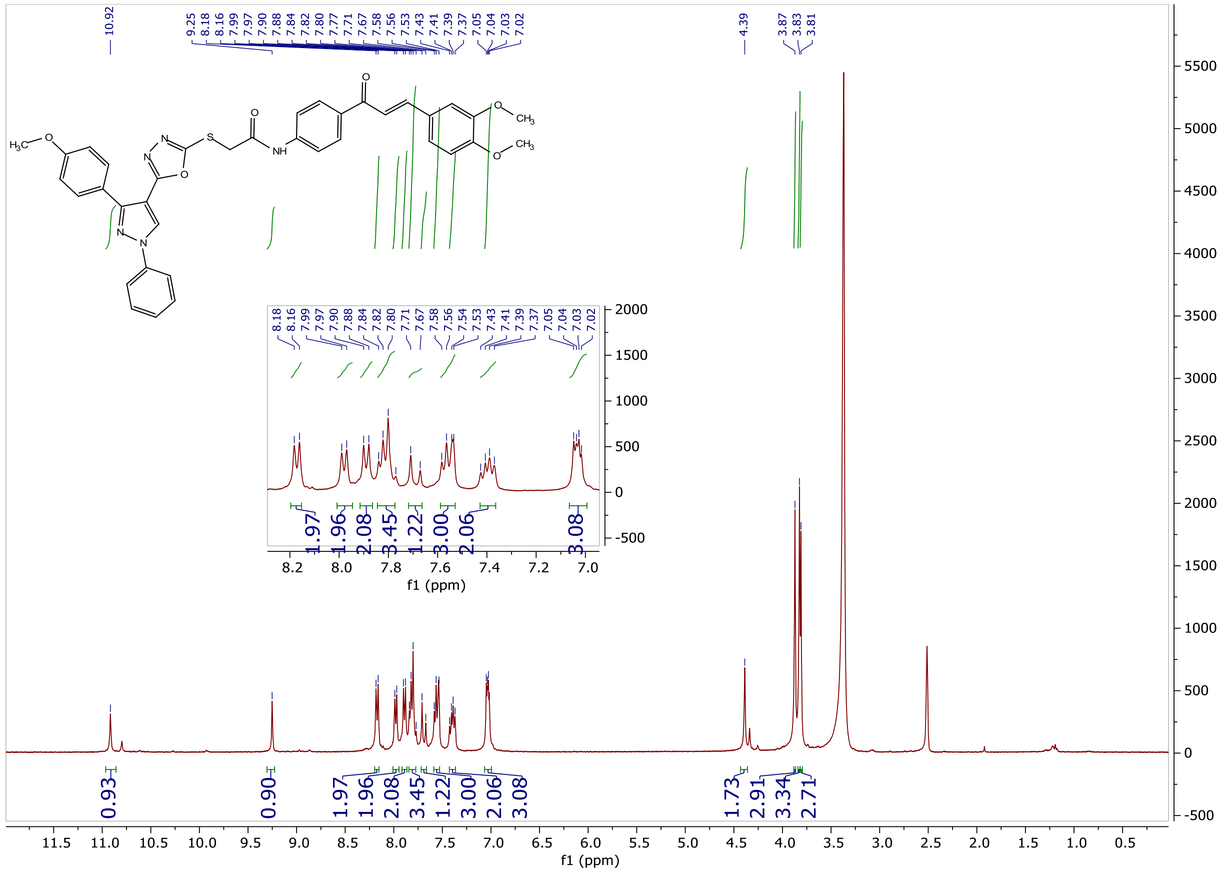

^{1}H NMR spectrum of compound **14c**

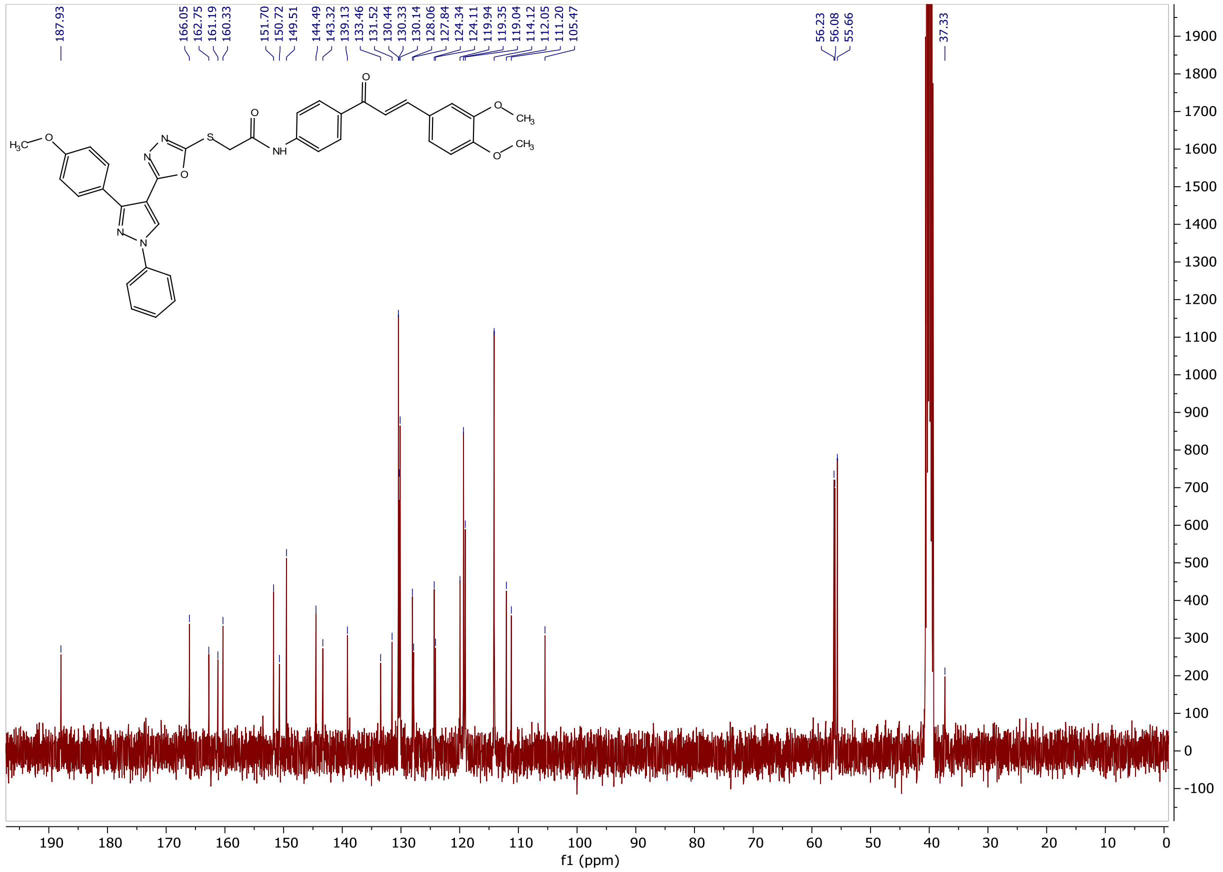

^{13}C NMR spectrum of compound **14c**

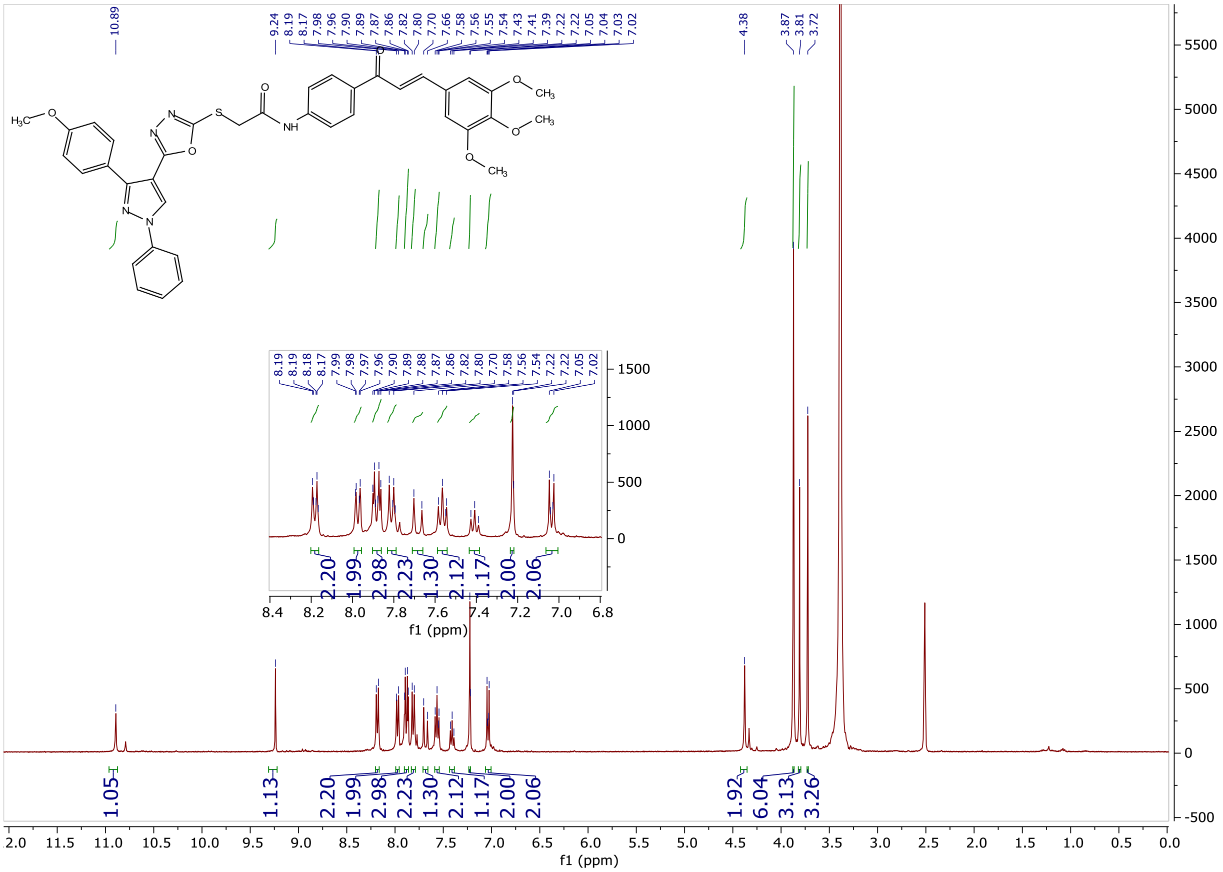

^{1}H NMR spectrum of compound **14d**

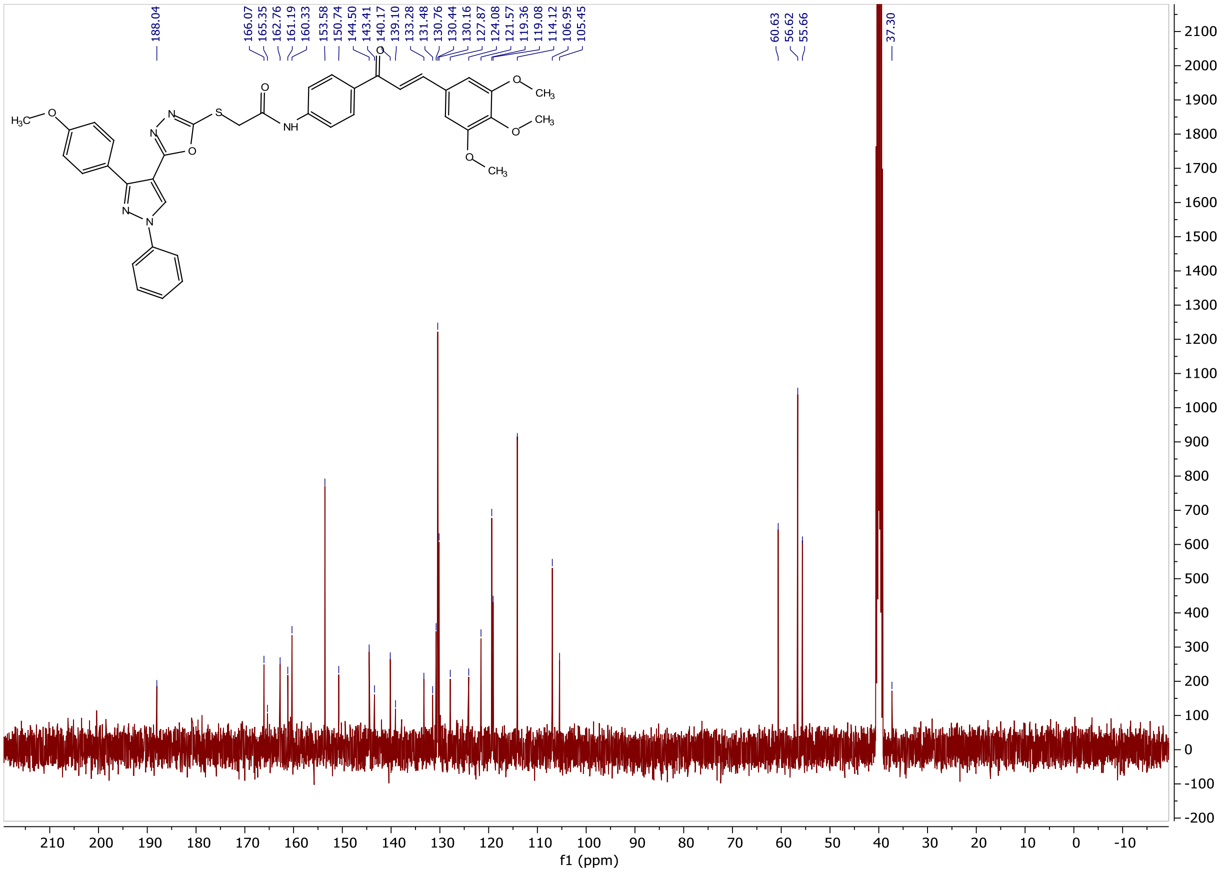

^{13}C NMR spectrum of compound **14d**

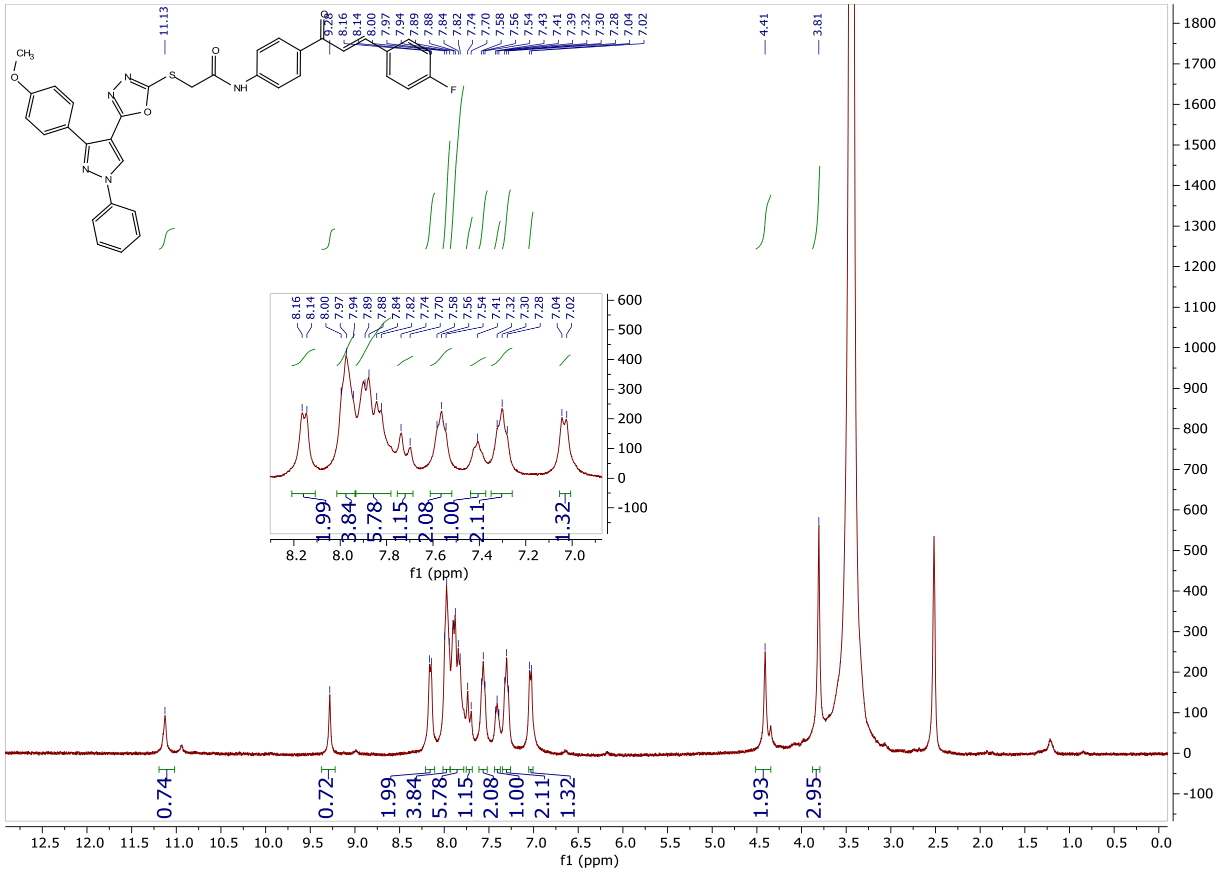

^{1}H NMR spectrum of compound **14e**

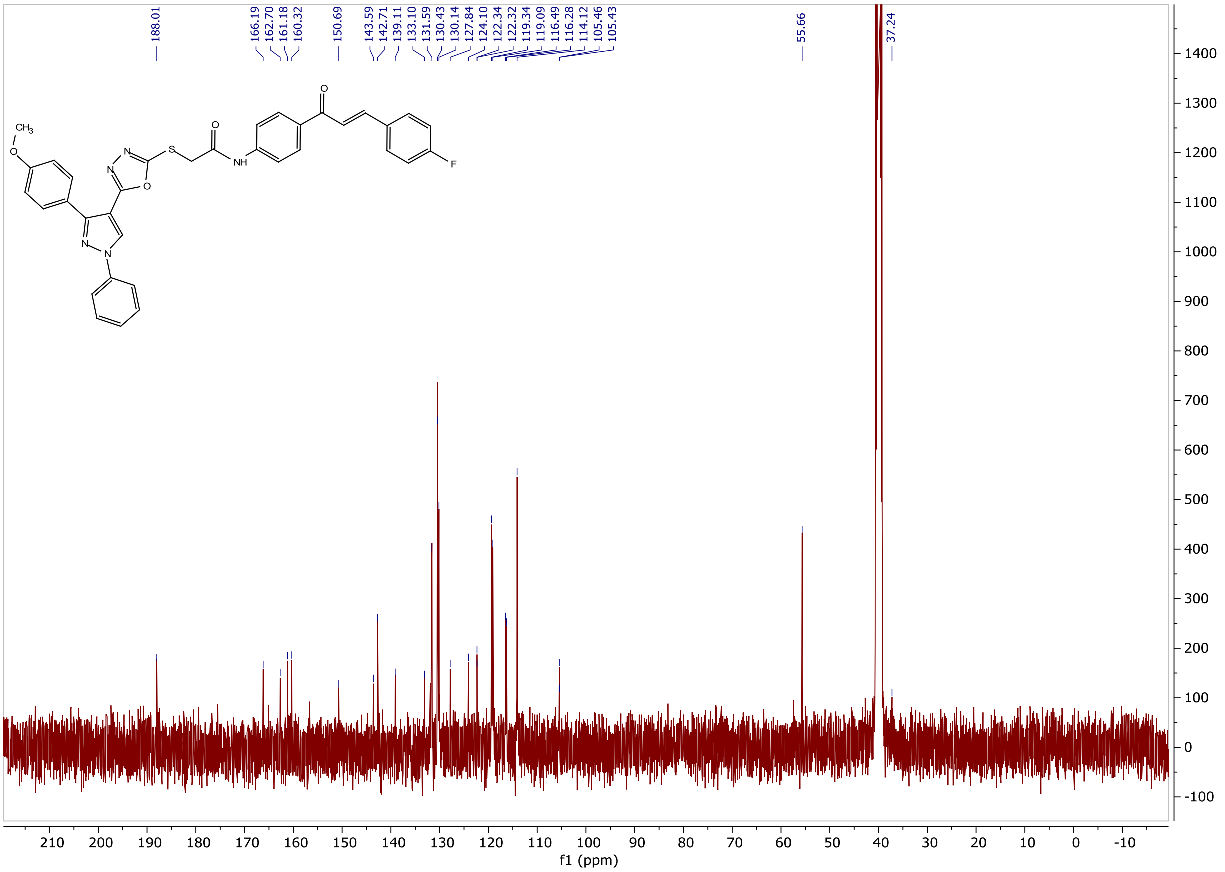

^{13}C NMR spectrum of compound **14e**

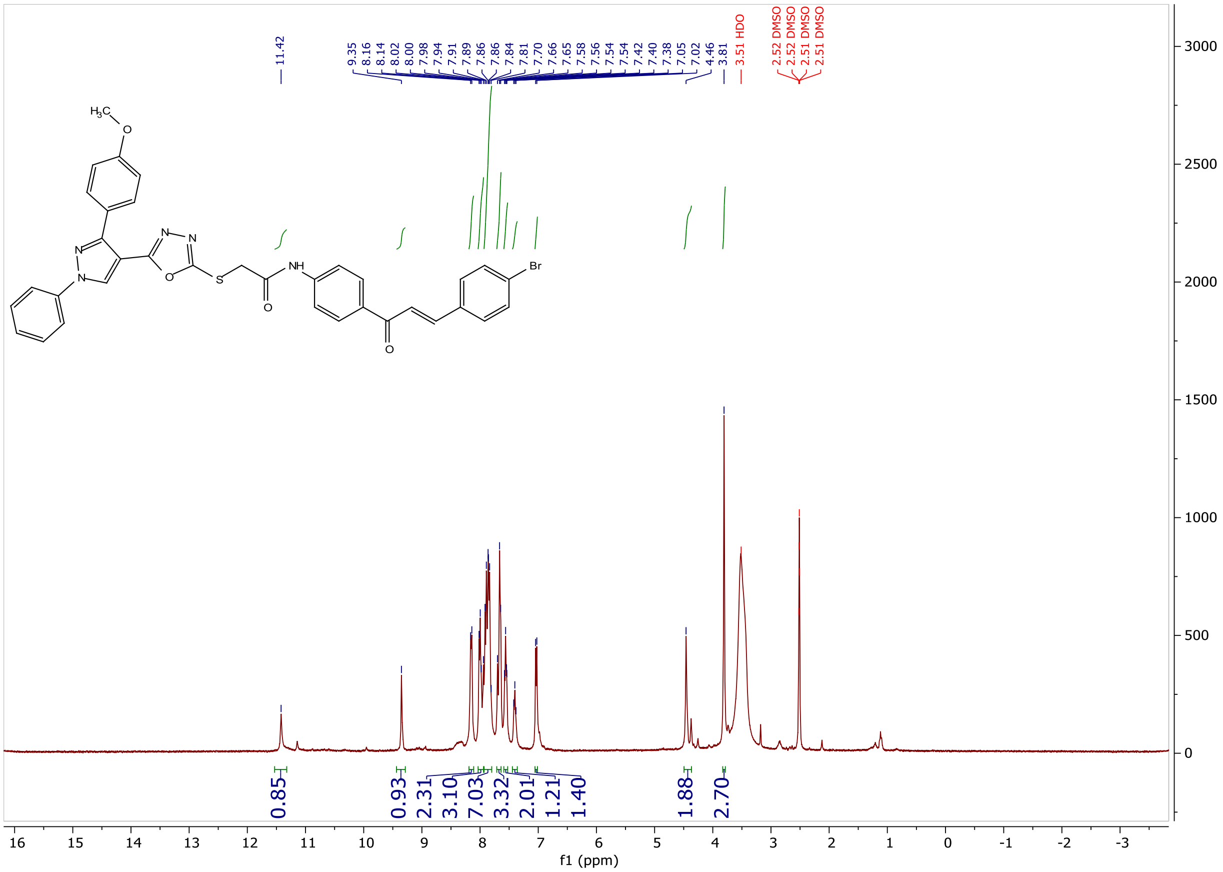

^{1}H NMR spectrum of compound **14f**

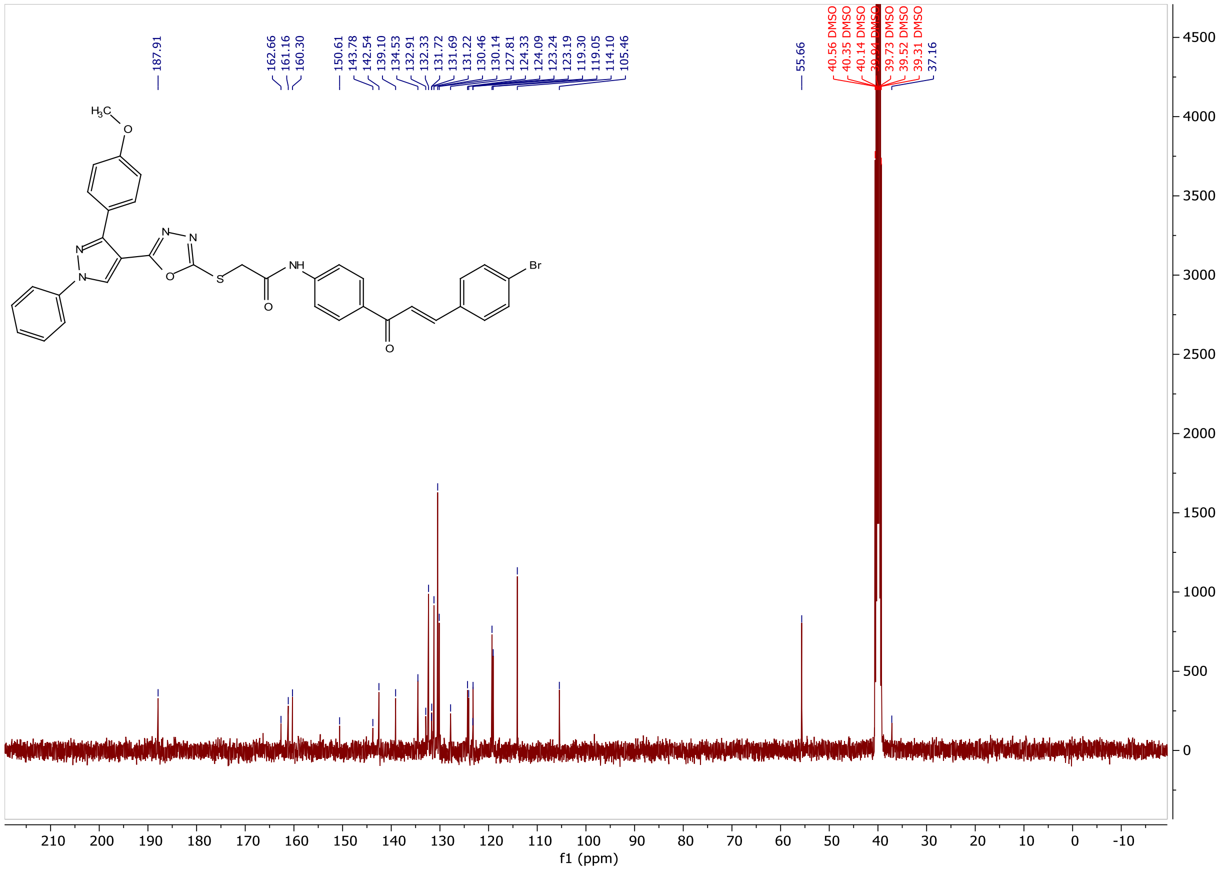

^{13}C NMR spectrum of compound **14f**

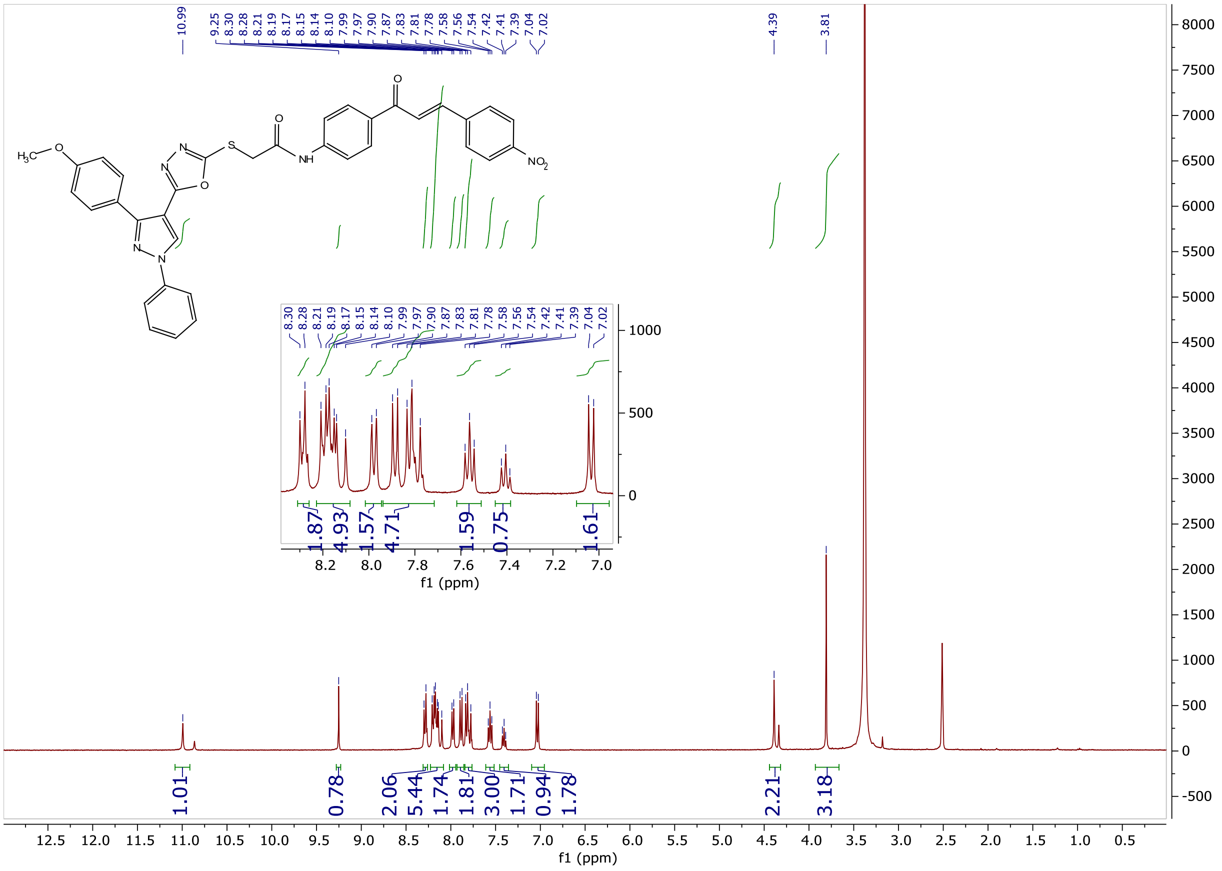

^{1}H NMR spectrum of compound **14g**

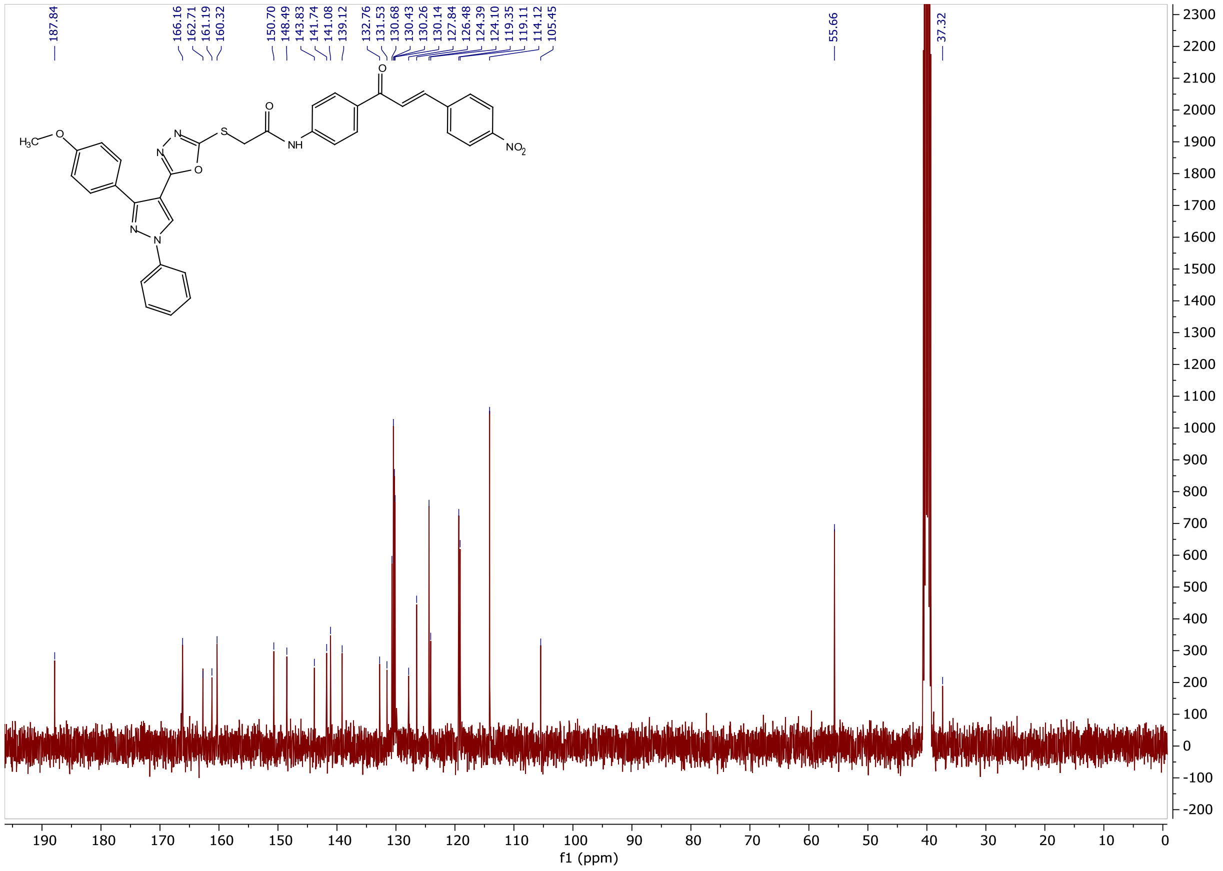

^{13}C NMR spectrum of compound **14g**

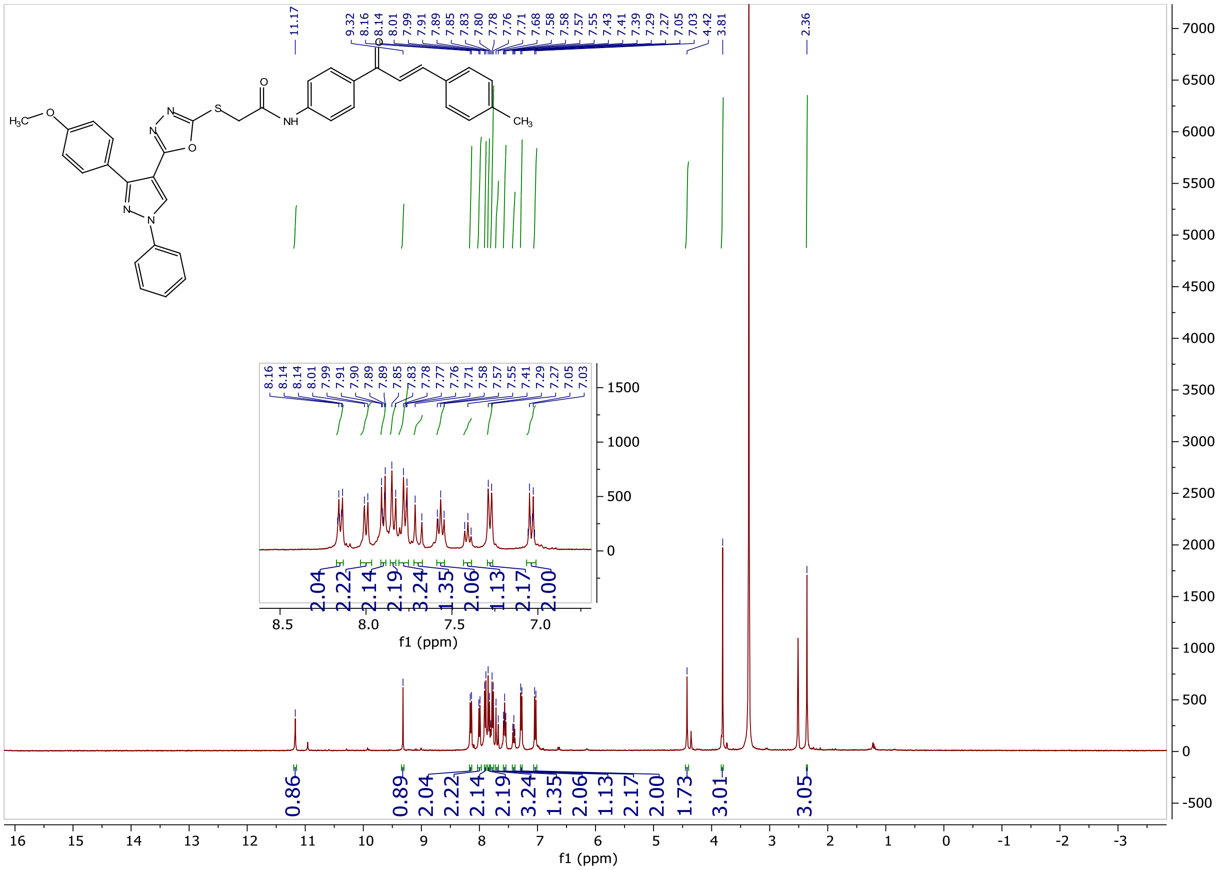

^{1}H NMR spectrum of compound **14h**

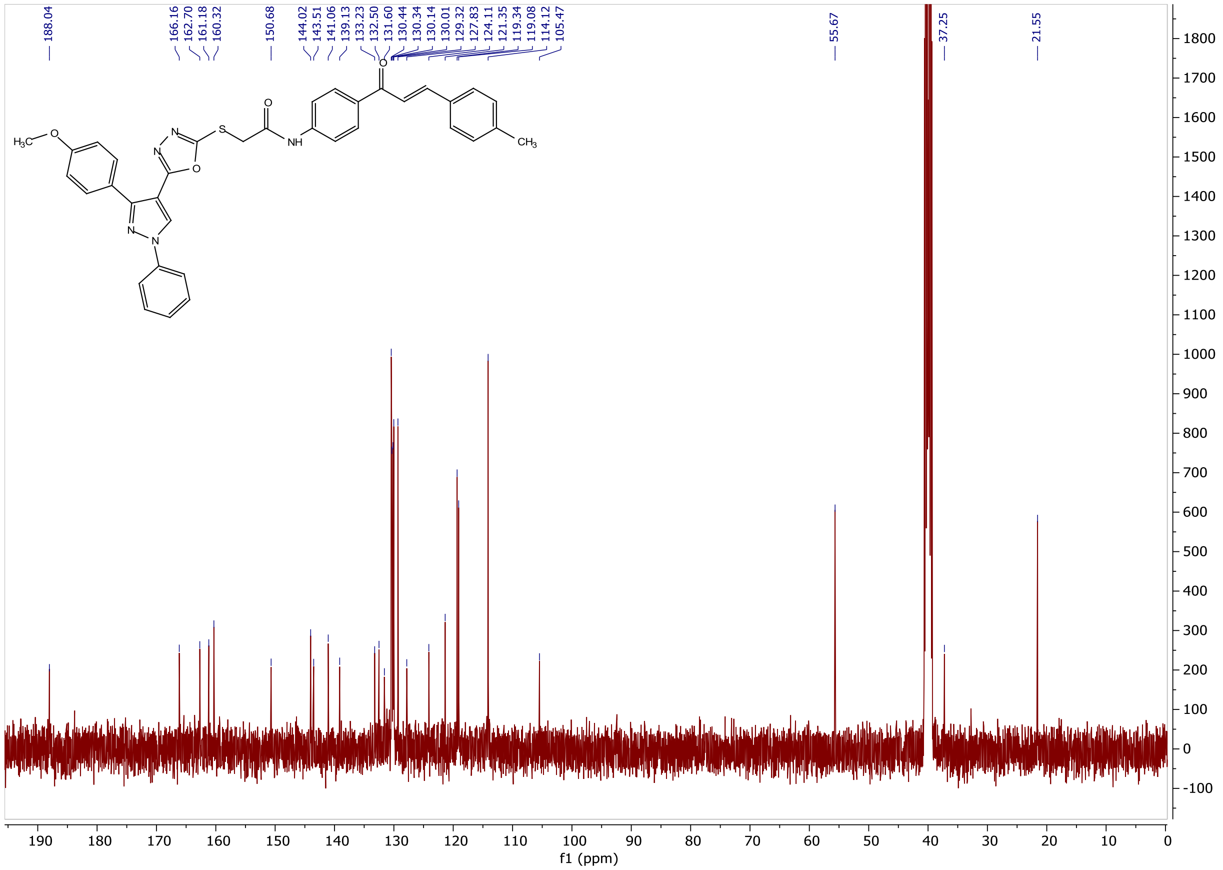

^{13}C NMR spectrum of compound **14h**

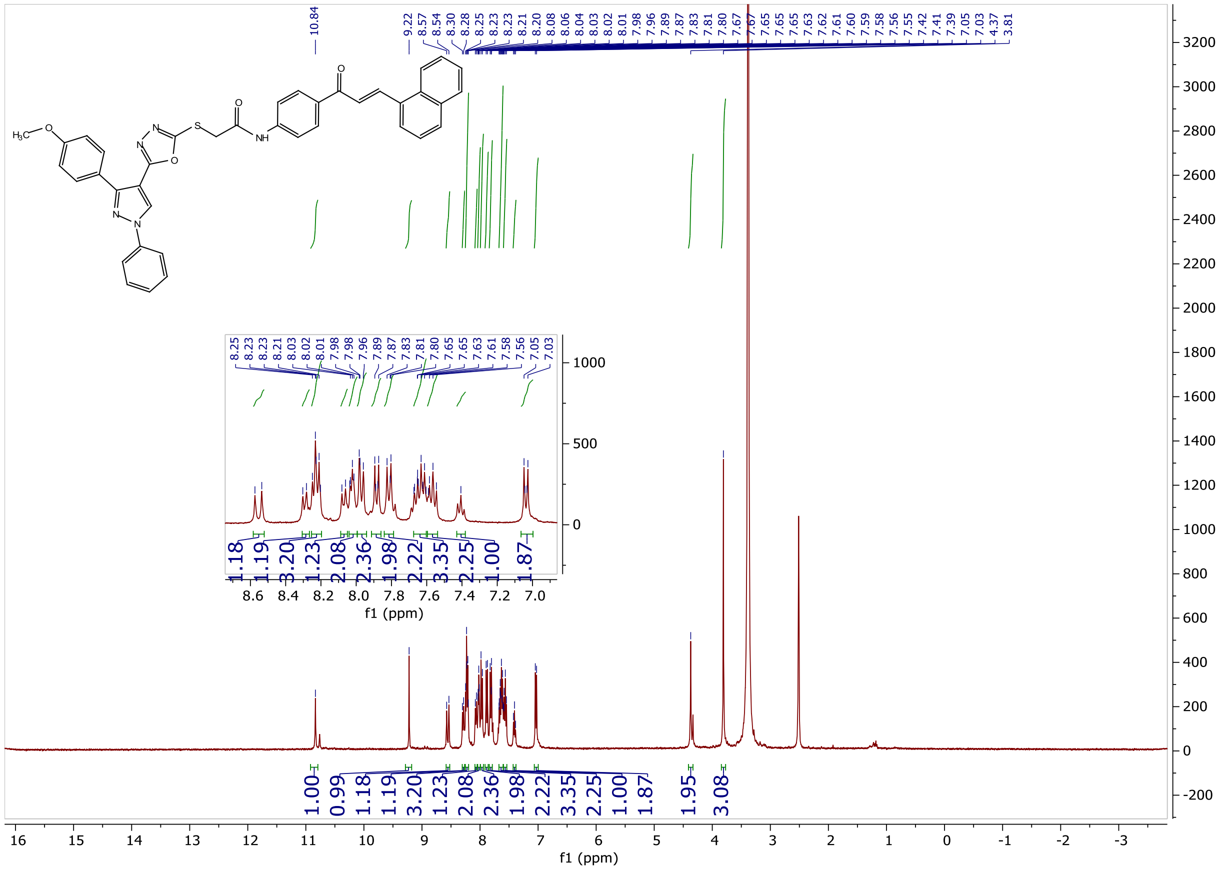

^{1}H NMR spectrum of compound **14i**

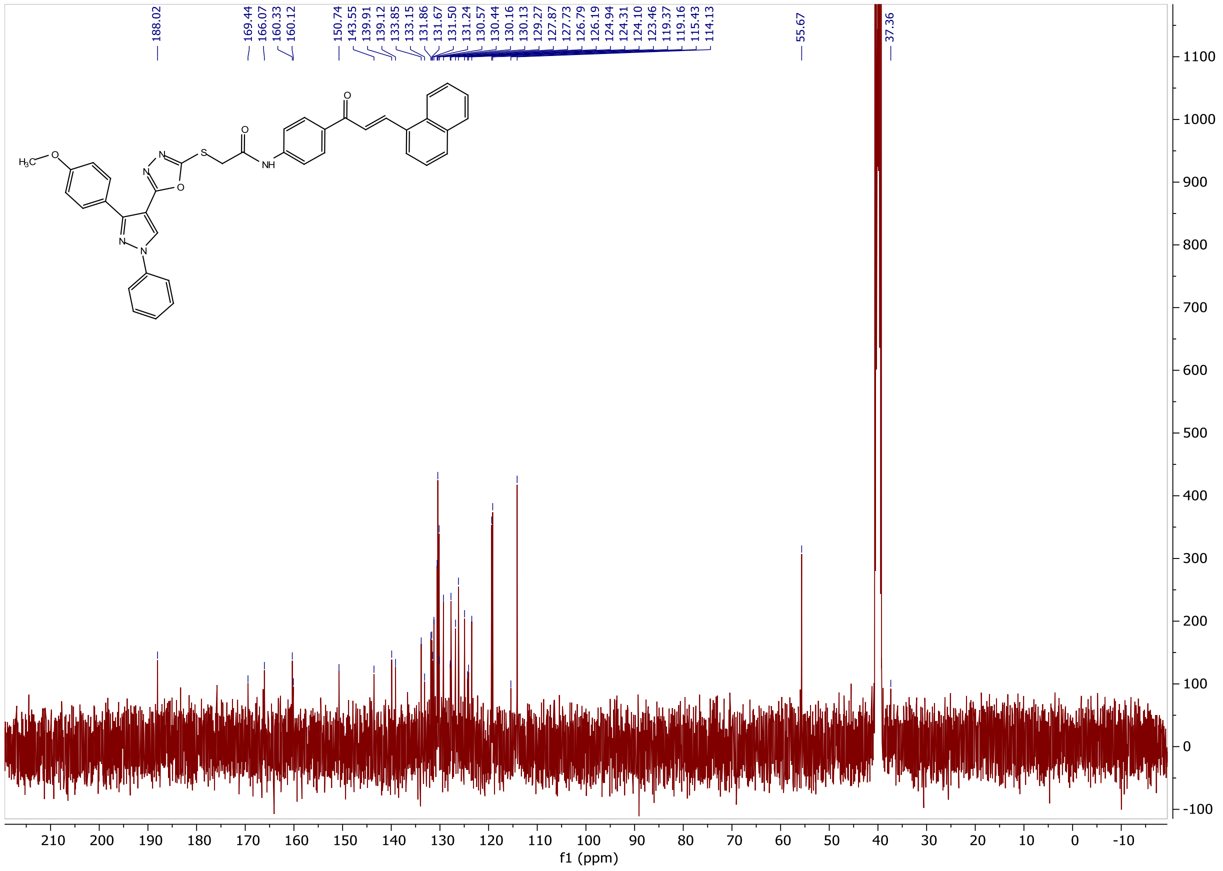

^{13}C NMR spectrum of compound **14i**

HPLC data

22/05/2025 11:48 ص | Chromatogram D:\AHMED\IMPURITY\drug 10a.prm | Page 1 of 2

Sohag University
CENTRAL LAB
DR MOHAMED GAMAL

Chromatogram Info:

File Name	: D:\AHMED\IMPURITY\drug 10a.prm	File Created	: 22/05/2025 11:40:21 ص
Origin	: Acquired, Acquisition started 22/05/2025 11:25:12 ص	Acquired Date	: 22/05/2025 11:40:21 ص
Original Project	: HPLC	By	: Administrator

Printed Version Info:

Printed Version	: - #3; 22/05/2025 11:47:57 ص, IA: 8.0 Rev.3	Printed Date	: 22/05/2025 11:48:14 ص
Report Style	: D:\HPLC\Common\Chromatogram.sty	By	: Administrator
Calibration File	: None		
Project	: HPLC		

Sample Description:

Sample ID	: drug 10a
Sample	:

Sample Parameters:

Amount	: 0.0	Dilution	: 1.0
Inj. Volume [µL]	: 20.0		
ISTD1 Amount	: 0.0	ISTD2 Amount	: 0.0
ISTD3 Amount	: 0.0	ISTD4 Amount	: 0.0
ISTD5 Amount	: 0.0	ISTD6 Amount	: 0.0
ISTD7 Amount	: 0.0	ISTD8 Amount	: 0.0
ISTD9 Amount	: 0.0	ISTD10 Amount	: 0.0

Analysis User Variables:		Method User Variables:	
AnalysisUserVar1	: 0	MethodUserVar1	: 0
AnalysisUserVar2	: 0	MethodUserVar2	: 0
AnalysisUserVar3	: 0	MethodUserVar3	: 0

Acquisition Method	: D:\ahmed\tolt\tolt separation - #201; 22/05/2025 11:09:38 ص		
Description	: aa		
Created	: 17/07/2023 02:47:53 ص	By	: Administrator
Modified	: 22/05/2025 11:09:38 ص	By	: Administrator

Processing Method	: D:\ahmed\tolt\tolt separation - #201; 22/05/2025 11:09:38 ص + manual changes		
Description	: aa		
Created	: 17/07/2023 02:47:53 ص	By	: Administrator
Modified	: 22/05/2025 11:09:38 ص	By	: Administrator

Column	:	Detection	:
Mobile Phase	:	Temperature	:
Flow Rate	:	Pressure	:
Note	:		

Autostop	: None	External Start	: Start Only, Down
Subtraction Chromatogram	: (None)	Matching	: Scale Subtraction Chromatogram

Base	: Not Used	Calibration File	: None	Calculation	: Uncal
Scale Factor	: Not Used	Units After Scaling	: Not Used	Uncal. Response	: 0
Unretained Time	: 0.00 min	Column Length	: 50.00 mm	Column Calc.	: From Width at 50% of Height
Result Table Reports	: All Peaks	Hide ISTD Peak	: Enabled		
Method User Variables:					
MethodUserVar1	: 0	MethodUserVar2	: 0	MethodUserVar3	: 0

HPLC chromatogram info of compound 10a.

HPLC chromatogram info of compound 10a.

22/05/2025 11:48 ص   Chromatogram D:\AHMED\IMPURITY\drug 10a.prm   Page 2 of 2

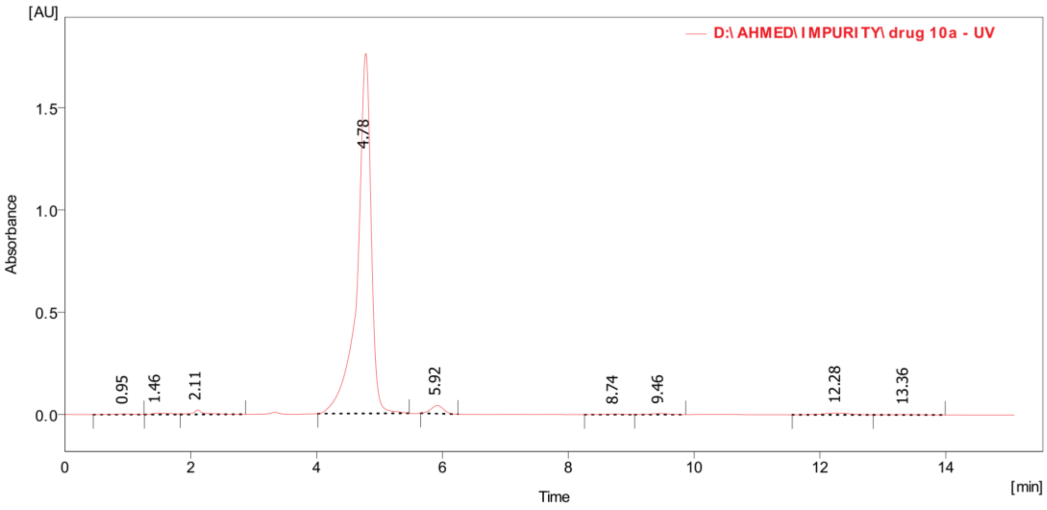

Result Table (Uncal - D:\AHMED\IMPURITY\drug 10a - UV)

	Reten. Time [min]	Area [mAU.s]	Area [%]	Compound Name
1	0.948	70.130	0.2	
2	1.463	137.381	0.5	
3	2.108	268.987	1.0	
4	4.782	26739.696	94.6	
5	5.918	552.330	2.0	
6	8.742	79.680	0.3	
7	9.462	118.724	0.4	
8	12.277	254.366	0.9	
9	13.355	38.686	0.1	
	Total	28259.980	100.0	

HPLC chromatogram of compound 10a.

Sohag University
CENTRAL LAB
DR MOHAMED GAMAL

Chromatogram Info:

File Name	: D:\AHMED\IMPURITY\drug 10 b.prm	File Created	: 04/12/2024 02:36:23 م
Origin	: Acquired, Acquisition started 04/12/2024 02:20:12 م	Acquired Date	: 04/12/2024 02:36:23 م
Original Project	: HPLC	By	: Administrator

Printed Version Info:

Printed Version	: - #2; 04/12/2024 02:51:12 م, IA: 8.0 Rev.3	Printed Date	: 04/12/2024 02:51:19 م
Report Style	: D:\HPLC\Common\Chromatogram.sty	By	: Administrator
Calibration File	: None		
Project	: HPLC		

Sample Description:

Sample ID	: drug 10 b
Sample	:

Sample Parameters:

Amount	: 0.0	Dilution	: 1.0
Inj. Volume [µL]	: 20.0		
ISTD1 Amount	: 0.0	ISTD2 Amount	: 0.0
ISTD3 Amount	: 0.0	ISTD4 Amount	: 0.0
ISTD5 Amount	: 0.0	ISTD6 Amount	: 0.0
ISTD7 Amount	: 0.0	ISTD8 Amount	: 0.0
ISTD9 Amount	: 0.0	ISTD10 Amount	: 0.0

Analysis User Variables:		Method User Variables:	
AnalysisUserVar1	: 0	MethodUserVar1	: 0
AnalysisUserVar2	: 0	MethodUserVar2	: 0
AnalysisUserVar3	: 0	MethodUserVar3	: 0

Acquisition Method	: D:\ahmed\tolt\tolt separation - #188; 04/12/2024 02:01:05 م		
Description	: aa		
Created	: 17/07/2023 02:47:53 ص	By	: Administrator
Modified	: 04/12/2024 02:01:05 م	By	: Administrator

Processing Method	: D:\ahmed\tolt\tolt separation - #188; 04/12/2024 02:01:05 م + manual changes		
Description	: aa		
Created	: 17/07/2023 02:47:53 ص	By	: Administrator
Modified	: 04/12/2024 02:01:05 م	By	: Administrator

Column	:	Detection	:
Mobile Phase	:	Temperature	:
Flow Rate	:	Pressure	:
Note	:		

Autostop	: None	External Start	: Start Only, Down
Subtraction Chromatogram	: (None)	Matching	: Scale Subtraction Chromatogram

Base	: Not Used	Calibration File	: None	Calculation	: Uncal
Scale Factor	: Not Used	Units After Scaling	: Not Used	Uncal. Response	: 0
Unretained Time	: 0.00 min	Column Length	: 50.00 mm	Column Calc.	: From Width at 50% of Height
Result Table Reports	: All Peaks	Hide ISTD Peak	: Enabled		
Method User Variables:					
MethodUserVar1	: 0	MethodUserVar2	: 0	MethodUserVar3	: 0

HPLC chromatogram info of compound 10b.

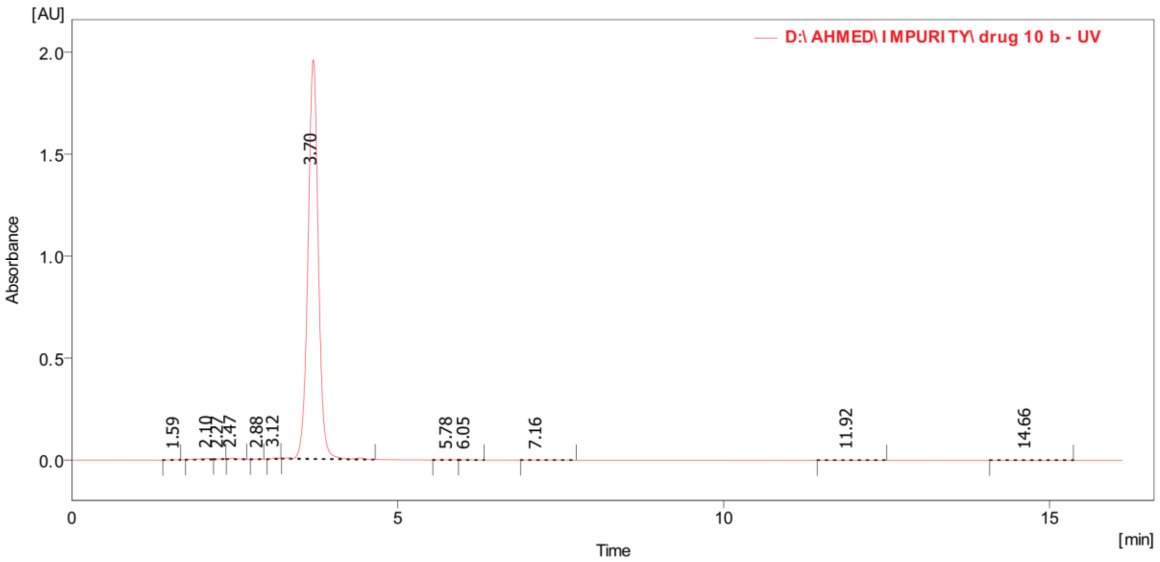

Result Table (Uncal - D:\AHMED\IMPURITY\drug 10 b - UV)

	Reten. Time [min]	Area [mAU.s]	Area [%]	Compound Name
1	1.590	5.101	0.0	
2	2.098	54.444	0.3	
3	2.270	10.819	0.0	
4	2.467	41.317	0.2	
5	2.877	8.302	0.0	
6	3.122	25.791	0.1	
7	3.703	21560.834	99.1	
8	5.782	13.634	0.1	
9	6.048	11.554	0.1	
10	7.155	17.094	0.1	
11	11.922	4.525	0.0	
12	14.657	8.812	0.0	
	Total	21762.226	100.0	

HPLC chromatogram of compound 10b.

Sohag University
CENTRAL LAB
DR MOHAMED GAMAL

Chromatogram Info:

File Name	: D:\ahmed\impurity\drug 10c.prm	File Created	: 22/05/2025 11:56:02 ص
Origin	: Acquired, Acquisition started 22/05/2025 11:40:30 ص	Acquired Date	: 22/05/2025 11:56:02 ص
Original Project	: HPLC	By	: Administrator

Printed Version Info:

Printed Version	: - #2; 22/05/2025 11:58:58 ص, IA: 8.0 Rev.3	Printed Date	: 22/05/2025 11:59:28 ص
Report Style	: D:\HPLC\Common\Chromatogram.sty	By	: Administrator
Calibration File	: None		
Project	: HPLC		

Sample Description:

Sample ID	: drug 10c
Sample	:

Sample Parameters:

Amount	: 0.0	Dilution	: 1.0
Inj. Volume [µL]	: 20.0		
ISTD1 Amount	: 0.0	ISTD2 Amount	: 0.0
ISTD3 Amount	: 0.0	ISTD4 Amount	: 0.0
ISTD5 Amount	: 0.0	ISTD6 Amount	: 0.0
ISTD7 Amount	: 0.0	ISTD8 Amount	: 0.0
ISTD9 Amount	: 0.0	ISTD10 Amount	: 0.0

Analysis User Variables:		Method User Variables:	
AnalysisUserVar1	: 0	MethodUserVar1	: 0
AnalysisUserVar2	: 0	MethodUserVar2	: 0
AnalysisUserVar3	: 0	MethodUserVar3	: 0

Acquisition Method	: D:\ahmed\tolt\tolt separation - #201; 22/05/2025 11:09:38 ص		
Description	: aa		
Created	: 17/07/2023 02:47:53 ص	By	: Administrator
Modified	: 22/05/2025 11:09:38 ص	By	: Administrator

Processing Method	: D:\ahmed\tolt\tolt separation - #201; 22/05/2025 11:09:38 ص + manual changes		
Description	: aa		
Created	: 17/07/2023 02:47:53 ص	By	: Administrator
Modified	: 22/05/2025 11:09:38 ص	By	: Administrator

Column	:	Detection	:
Mobile Phase	:	Temperature	:
Flow Rate	:	Pressure	:
Note	:		

Autostop	: None	External Start	: Start Only, Down
Subtraction Chromatogram	: (None)	Matching	: Scale Subtraction Chromatogram

Base	: Not Used	Calibration File	: None	Calculation	: Uncal
Scale Factor	: Not Used	Units After Scaling	: Not Used	Uncal. Response	: 0
Unretained Time	: 0.00 min	Column Length	: 50.00 mm	Column Calc.	: From Width at 50% of Height
Result Table Reports	: All Peaks	Hide ISTD Peak	: Enabled		
Method User Variables:					
MethodUserVar1	: 0	MethodUserVar2	: 0	MethodUserVar3	: 0

HPLC chromatogram info of compound 10c.

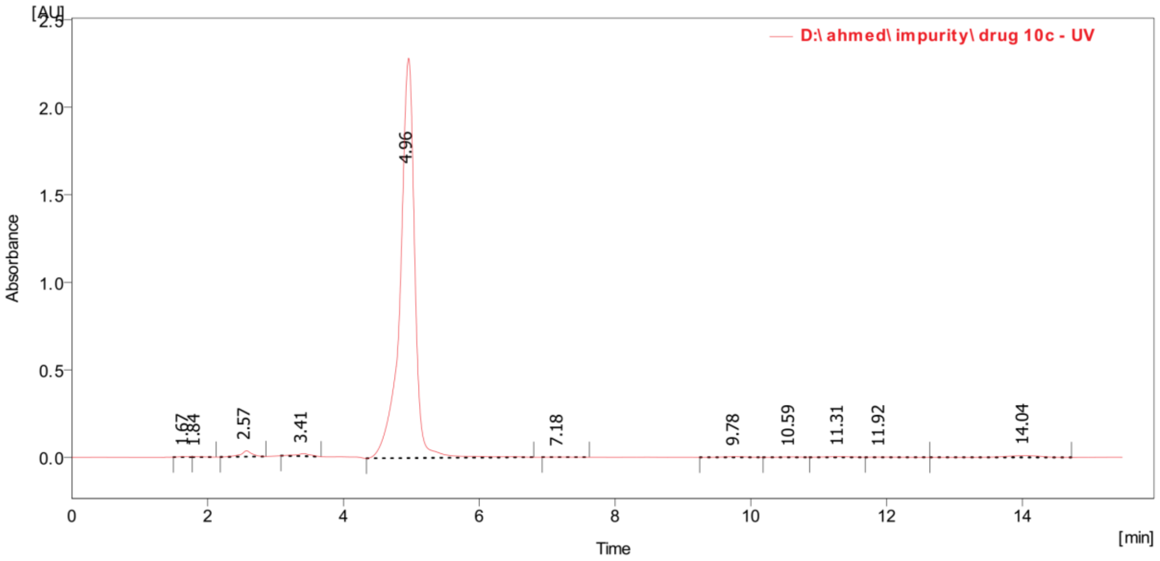

Result Table (Uncal - D:\ahmed\impurity\drug 10c - UV)

	Reten. Time [min]	Area [mAU.s]	Area [%]	Compound Name
1	1.665	29.135	0.1	
2	1.837	24.191	0.1	
3	2.572	391.196	1.1	
4	3.413	223.761	0.6	
5	4.958	34020.741	96.1	
6	7.178	17.780	0.1	
7	9.778	90.830	0.3	
8	10.590	60.300	0.2	
9	11.308	127.613	0.4	
10	11.920	59.287	0.2	
11	14.040	363.048	1.0	
	Total	35407.882	100.0	

HPLC chromatogram of compound 10c.

04/12/2024 03:23 م   Chromatogram D:\ahmed\impurity\drug 11 a.prm   Page 1 of 2

Sohag University
CENTRAL LAB
DR MOHAMED GAMAL

Chromatogram Info:

File Name	: D:\ahmed\impurity\drug 11 a.prm	File Created	: 04/12/2024 03:09:24 م
Origin	: Acquired, Acquisition started 04/12/2024 02:54:13 م	Acquired Date	: 04/12/2024 03:09:24 م
Original Project	: HPLC	By	: Administrator

Printed Version Info:

Printed Version	: - #3; 04/12/2024 03:23:00 م, IA: 8.0 Rev.3	Printed Date	: 04/12/2024 03:23:45 م
Report Style	: D:\HPLC\Common\Chromatogram.sty	By	: Administrator
Calibration File	: None		
Project	: HPLC		

Sample Description:

Sample ID	: drug 11 a
Sample	:

Sample Parameters:

Amount	: 0.0	Dilution	: 1.0
Inj. Volume [µL]	: 20.0		
ISTD1 Amount	: 0.0	ISTD2 Amount	: 0.0
ISTD3 Amount	: 0.0	ISTD4 Amount	: 0.0
ISTD5 Amount	: 0.0	ISTD6 Amount	: 0.0
ISTD7 Amount	: 0.0	ISTD8 Amount	: 0.0
ISTD9 Amount	: 0.0	ISTD10 Amount	: 0.0

Analysis User Variables:		Method User Variables:	
AnalysisUserVar1	: 0	MethodUserVar1	: 0
AnalysisUserVar2	: 0	MethodUserVar2	: 0
AnalysisUserVar3	: 0	MethodUserVar3	: 0

Acquisition Method	: D:\ahmed\tolt\tolt separation - #188; 04/12/2024 02:01:05 م		
Description	: aa		
Created	: 17/07/2023 02:47:53 ص	By	: Administrator
Modified	: 04/12/2024 02:01:05 م	By	: Administrator

Processing Method	: D:\ahmed\tolt\tolt separation - #188; 04/12/2024 02:01:05 م + manual changes		
Description	: aa		
Created	: 17/07/2023 02:47:53 ص	By	: Administrator
Modified	: 04/12/2024 02:01:05 م	By	: Administrator

Column	:	Detection	:
Mobile Phase	:	Temperature	:
Flow Rate	:	Pressure	:
Note	:		

Autostop	: None	External Start	: Start Only, Down
Subtraction Chromatogram	: (None)	Matching	: Scale Subtraction Chromatogram

Base	: Not Used	Calibration File	: None	Calculation	: Uncal
Scale Factor	: Not Used	Units After Scaling	: Not Used	Uncal. Response	: 0
Unretained Time	: 0.00 min	Column Length	: 50.00 mm	Column Calc.	: From Width at 50% of Height
Result Table Reports	: All Peaks	Hide ISTD Peak	: Enabled		
Method User Variables:					
MethodUserVar1	: 0	MethodUserVar2	: 0	MethodUserVar3	: 0

HPLC chromatogram info of compound 11a.

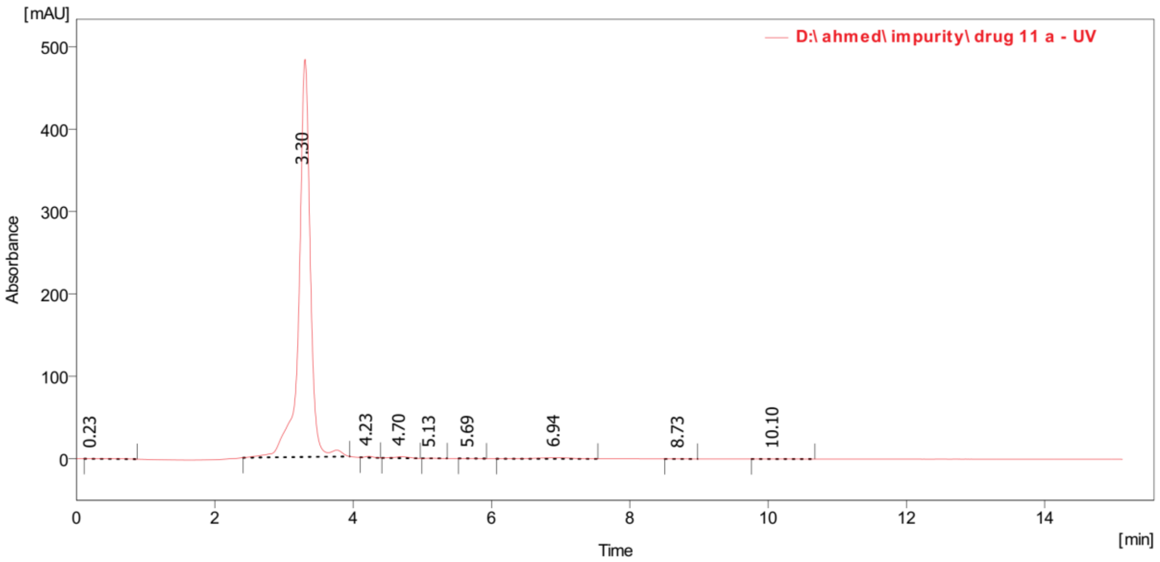

Result Table (Uncal - D:\ahmed\impurity\drug 11 a - UV)

	Reten. Time [min]	Area [mAU.s]	Area [%]	Compound Name
1	0.233	7.001	0.1	
2	3.303	5557.814	98.3	
3	4.227	11.554	0.2	
4	4.702	23.193	0.4	
5	5.135	2.977	0.1	
6	5.693	2.942	0.1	
7	6.937	44.969	0.8	
8	8.732	0.614	0.0	
9	10.100	1.943	0.0	
	Total	5653.007	100.0	

HPLC chromatogram of compound 11a.

Sohag University
CENTRAL LAB
DR MOHAMED GAMAL

Chromatogram Info:

File Name	: D:\AHMED\IMPURITY\drug 11 b.prm	File Created	: 04/12/2024 02:18:35 م
Origin	: Acquired, Acquisition started 04/12/2024 02:03:27 م	Acquired Date	: 04/12/2024 02:18:35 م
Original Project	: HPLC	By	: Administrator

Printed Version Info:

Printed Version	: - #2; 04/12/2024 02:38:58 م, IA: 8.0 Rev.3	Printed Date	: 04/12/2024 02:43:10 م
Report Style	: D:\HPLC\Common\Chromatogram.sty	By	: Administrator
Calibration File	: None		
Project	: HPLC		

Sample Description:

Sample ID	: drug 11 b
Sample	:

Sample Parameters:

Amount	: 0.0	Dilution	: 1.0
Inj. Volume [µL]	: 20.0		
ISTD1 Amount	: 0.0	ISTD2 Amount	: 0.0
ISTD3 Amount	: 0.0	ISTD4 Amount	: 0.0
ISTD5 Amount	: 0.0	ISTD6 Amount	: 0.0
ISTD7 Amount	: 0.0	ISTD8 Amount	: 0.0
ISTD9 Amount	: 0.0	ISTD10 Amount	: 0.0

Analysis User Variables:		Method User Variables:	
AnalysisUserVar1	: 0	MethodUserVar1	: 0
AnalysisUserVar2	: 0	MethodUserVar2	: 0
AnalysisUserVar3	: 0	MethodUserVar3	: 0

Acquisition Method	: D:\ahmed\tolt\tolt separation - #188; 04/12/2024 02:01:05 م		
Description	: aa		
Created	: 17/07/2023 02:47:53 ص	By	: Administrator
Modified	: 04/12/2024 02:01:05 م	By	: Administrator

Processing Method	: D:\ahmed\tolt\tolt separation - #188; 04/12/2024 02:01:05 م + manual changes		
Description	: aa		
Created	: 17/07/2023 02:47:53 ص	By	: Administrator
Modified	: 04/12/2024 02:01:05 م	By	: Administrator

Column	:	Detection	:
Mobile Phase	:	Temperature	:
Flow Rate	:	Pressure	:
Note	:		

Autostop	: None	External Start	: Start Only, Down
Subtraction Chromatogram	: (None)	Matching	: Scale Subtraction Chromatogram

Base	: Not Used	Calibration File	: None	Calculation	: Uncal
Scale Factor	: Not Used	Units After Scaling	: Not Used	Uncal. Response	: 0
Unretained Time	: 0.00 min	Column Length	: 50.00 mm	Column Calc.	: From Width at 50% of Height
Result Table Reports	: All Peaks	Hide ISTD Peak	: Enabled		
Method User Variables:					
MethodUserVar1	: 0	MethodUserVar2	: 0	MethodUserVar3	: 0

HPLC chromatogram info of compound 11b.

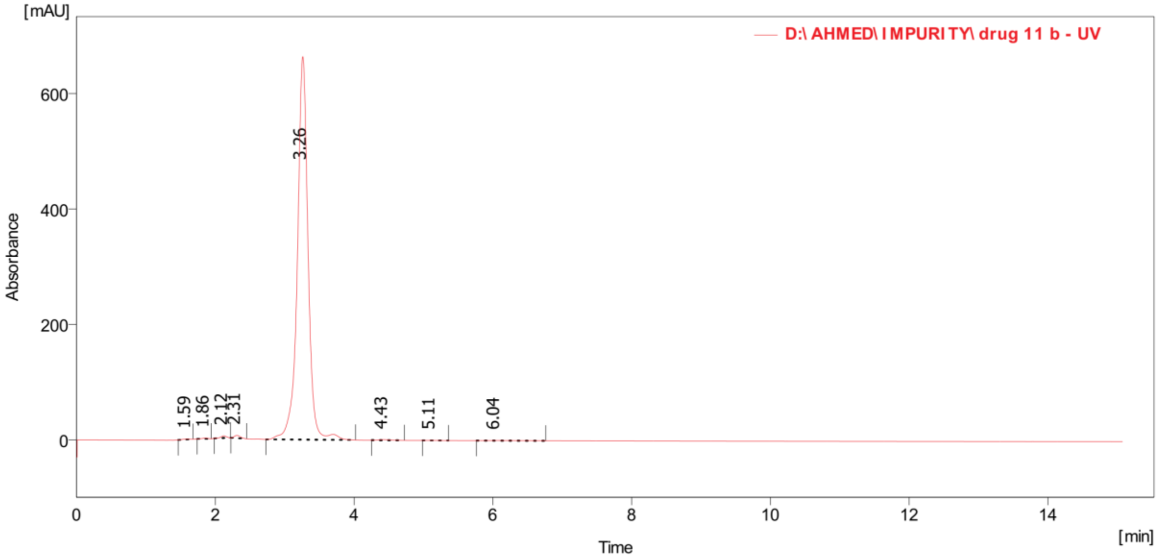

Result Table (Uncal - D: \AHMED\IMPURITY\drug 11 b - UV)

	Reten. Time [min]	Area [mAU.s]	Area [%]	Compound Name
1	1.592	5.433	0.1	
2	1.857	6.274	0.1	
3	2.123	21.236	0.3	
4	2.312	28.430	0.4	
5	3.262	7127.562	98.9	
6	4.430	11.805	0.2	
7	5.113	2.389	0.0	
8	6.045	7.129	0.1	
	Total	7210.259	100.0	

HPLC chromatogram of compound 11b.

Sohag University
CENTRAL LAB
DR MOHAMED GAMAL

Chromatogram Info:

File Name	: D:\ahmed\impurity\drug 11 c.prm	File Created	: 04/12/2024 03:25:33 م
Origin	: Acquired, Acquisition started 04/12/2024 03:10:28 م	Acquired Date	: 04/12/2024 03:25:33 م
Original Project	: HPLC	By	: Administrator

Printed Version Info:

Printed Version	: - #2; 04/12/2024 03:29:39 م, IA: 8.0 Rev.3	Printed Date	: 04/12/2024 03:29:46 م
Report Style	: D:\HPLC\Common\Chromatogram.sty	By	: Administrator
Calibration File	: None		
Project	: HPLC		

Sample Description:

Sample ID	: drug 11 c
Sample	:

Sample Parameters:

Amount	: 0.0	Dilution	: 1.0
Inj. Volume [µL]	: 20.0		
ISTD1 Amount	: 0.0	ISTD2 Amount	: 0.0
ISTD3 Amount	: 0.0	ISTD4 Amount	: 0.0
ISTD5 Amount	: 0.0	ISTD6 Amount	: 0.0
ISTD7 Amount	: 0.0	ISTD8 Amount	: 0.0
ISTD9 Amount	: 0.0	ISTD10 Amount	: 0.0

Analysis User Variables:		Method User Variables:	
AnalysisUserVar1	: 0	MethodUserVar1	: 0
AnalysisUserVar2	: 0	MethodUserVar2	: 0
AnalysisUserVar3	: 0	MethodUserVar3	: 0

Acquisition Method	: D:\ahmed\tolt\tolt separation - #188; 04/12/2024 02:01:05 م		
Description	: aa		
Created	: 17/07/2023 02:47:53 ص	By	: Administrator
Modified	: 04/12/2024 02:01:05 م	By	: Administrator

Processing Method	: D:\ahmed\tolt\tolt separation - #188; 04/12/2024 02:01:05 م + manual changes		
Description	: aa		
Created	: 17/07/2023 02:47:53 ص	By	: Administrator
Modified	: 04/12/2024 02:01:05 م	By	: Administrator

Column	:	Detection	:
Mobile Phase	:	Temperature	:
Flow Rate	:	Pressure	:
Note	:		

Autostop	: None	External Start	: Start Only, Down
Subtraction Chromatogram	: (None)	Matching	: Scale Subtraction Chromatogram

Base	: Not Used	Calibration File	: None	Calculation	: Uncal
Scale Factor	: Not Used	Units After Scaling	: Not Used	Uncal. Response	: 0
Unretained Time	: 0.00 min	Column Length	: 50.00 mm	Column Calc.	: From Width at 50% of Height
Result Table Reports	: All Peaks	Hide ISTD Peak	: Enabled		
Method User Variables:					
MethodUserVar1	: 0	MethodUserVar2	: 0	MethodUserVar3	: 0

HPLC chromatogram info of compound 11c.

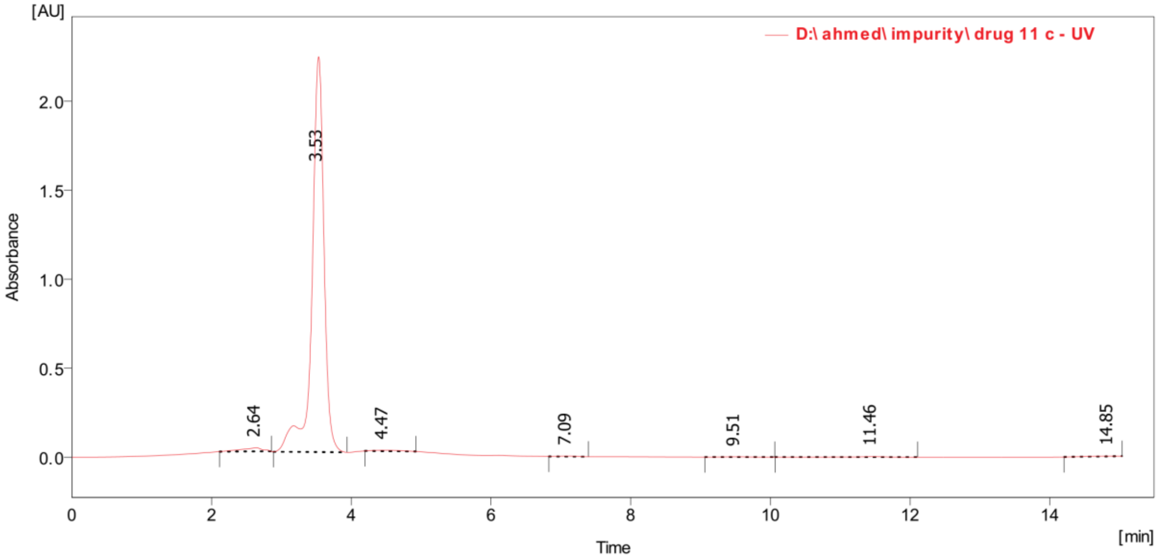

Result Table (Uncal - D:\ahmed\impurity\drug 11 c - UV)

	Reten. Time [min]	Area [mAU.s]	Area [%]	Compound Name
1	2.643	437.635	1.6	
2	3.532	26650.604	97.2	
3	4.472	141.085	0.5	
4	7.095	48.480	0.2	
5	9.505	30.345	0.1	
6	11.463	72.015	0.3	
7	14.850	48.965	0.2	
	Total	27429.129	100.0	

HPLC chromatogram info of compound 11c.

20/05/2025 02:13 م   Chromatogram D:\AHMED\IMPURITY\drug 12a.prm   Page 1 of 2

Sohag University
CENTRAL LAB
DR MOHAMED GAMAL

Chromatogram Info:

File Name	: D:\AHMED\IMPURITY\drug 12a.prm	File Created	: 20/05/2025 11:40:53 ص
Origin	: Acquired, Acquisition started 20/05/2025 12:29:45 م	Acquired Date	: 20/05/2025 12:45:44 م
Original Project	: HPLC	By	: Administrator

Printed Version Info:

Printed Version	: - #2; 20/05/2025 02:13:35 م, IA: 8.0 Rev.3	Printed Date	: 20/05/2025 02:13:49 م
Report Style	: D:\HPLC\Common\Chromatogram.sty	By	: Administrator
Calibration File	: None		
Project	: HPLC		

Sample Description:

Sample ID	: drug 12a
Sample	:

Sample Parameters:

Amount	: 0.0	Dilution	: 1.0
Inj. Volume [µL]	: 20.0		
ISTD1 Amount	: 0.0	ISTD2 Amount	: 0.0
ISTD3 Amount	: 0.0	ISTD4 Amount	: 0.0
ISTD5 Amount	: 0.0	ISTD6 Amount	: 0.0
ISTD7 Amount	: 0.0	ISTD8 Amount	: 0.0
ISTD9 Amount	: 0.0	ISTD10 Amount	: 0.0

Analysis User Variables:		Method User Variables:	
AnalysisUserVar1	: 0	MethodUserVar1	: 0
AnalysisUserVar2	: 0	MethodUserVar2	: 0
AnalysisUserVar3	: 0	MethodUserVar3	: 0

Acquisition Method	: D:\ahmed\tolt\tolt separation - #199; 20/05/2025 12:28:36 م		
Description	: aa		
Created	: 17/07/2023 02:47:53 ص	By	: Administrator
Modified	: 20/05/2025 12:28:36 م	By	: Administrator

Processing Method	: D:\ahmed\tolt\tolt separation - #199; 20/05/2025 12:28:36 م + manual changes		
Description	: aa		
Created	: 17/07/2023 02:47:53 ص	By	: Administrator
Modified	: 20/05/2025 12:28:36 م	By	: Administrator

Column	:	Detection	:
Mobile Phase	:	Temperature	:
Flow Rate	:	Pressure	:
Note	:		

Autostop	: None	External Start	: Start Only, Down
Subtraction Chromatogram	: (None)	Matching	: Scale Subtraction Chromatogram

Base	: Not Used	Calibration File	: None	Calculation	: Uncal
Scale Factor	: Not Used	Units After Scaling	: Not Used	Uncal. Response	: 0
Unretained Time	: 0.00 min	Column Length	: 50.00 mm	Column Calc.	: From Width at 50% of Height
Result Table Reports	: All Peaks	Hide ISTD Peak	: Enabled		
Method User Variables:					
MethodUserVar1	: 0	MethodUserVar2	: 0	MethodUserVar3	: 0

HPLC chromatogram info of compound 12a.

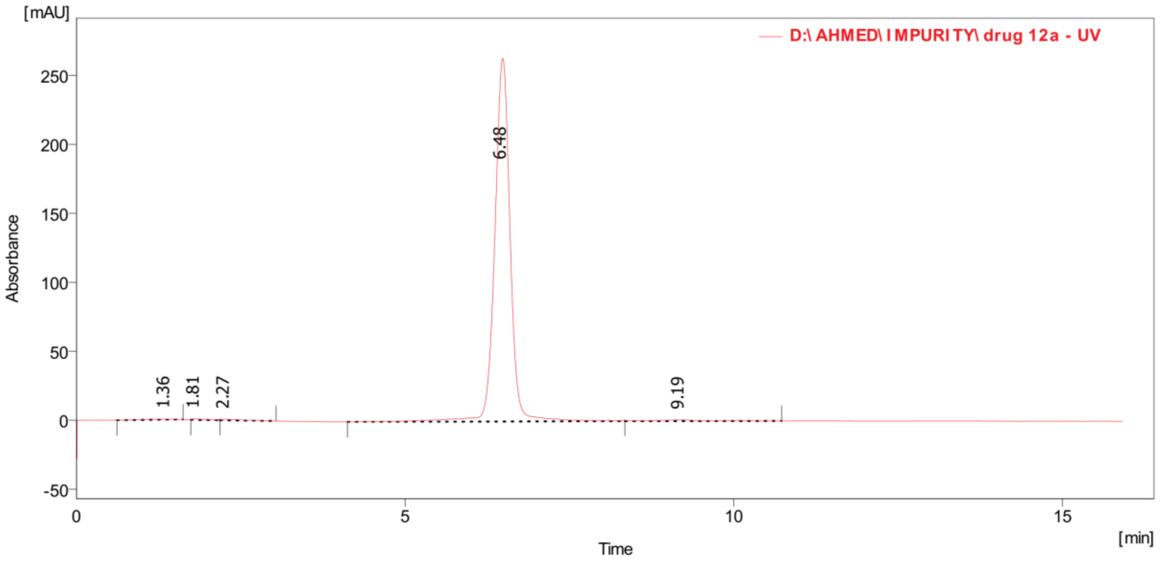

Result Table (Uncal - D:\AHMED\IMPURITY\drug 12a - UV)

	Reten. Time [min]	Area [mAU.s]	Area [%]	Compound Name
1	1.362	12.515	0.3	
2	1.813	10.646	0.2	
3	2.270	17.495	0.4	
4	6.483	4335.925	97.8	
5	9.187	57.224	1.3	
	Total	4433.804	100.0	

HPLC chromatogram of compound 12a.

Sohag University
CENTRAL LAB
DR MOHAMED GAMAL

Chromatogram Info:

File Name	: D:\AHMED\IMPURITY\drug 12b.prm	File Created	: 20/05/2025 11:54:18 ص
Origin	: Acquired, Acquisition started 20/05/2025 12:10:59 م	Acquired Date	: 20/05/2025 12:26:48 م
Original Project	: HPLC	By	: Administrator

Printed Version Info:

Printed Version	: - #2; 20/05/2025 02:04:36 م, IA: 8.0 Rev.3	Printed Date	: 20/05/2025 02:06:26 م
Report Style	: D:\HPLC\Common\Chromatogram.sty	By	: Administrator
Calibration File	: None		
Project	: HPLC		

Sample Description:

Sample ID	: drug 12b
Sample	:

Sample Parameters:

Amount	: 0.0	Dilution	: 1.0
Inj. Volume [μL]	: 20.0		
ISTD1 Amount	: 0.0	ISTD2 Amount	: 0.0
ISTD3 Amount	: 0.0	ISTD4 Amount	: 0.0
ISTD5 Amount	: 0.0	ISTD6 Amount	: 0.0
ISTD7 Amount	: 0.0	ISTD8 Amount	: 0.0
ISTD9 Amount	: 0.0	ISTD10 Amount	: 0.0

Analysis User Variables:		Method User Variables:	
AnalysisUserVar1	: 0	MethodUserVar1	: 0
AnalysisUserVar2	: 0	MethodUserVar2	: 0
AnalysisUserVar3	: 0	MethodUserVar3	: 0

Acquisition Method	: D:\ahmed\tolt\tolt separation - #198; 20/05/2025 12:06:15 م		
Description	: aa		
Created	: 17/07/2023 02:47:53 ص	By	: Administrator
Modified	: 20/05/2025 12:06:15 م	By	: Administrator

Processing Method	: D:\ahmed\tolt\tolt separation - #198; 20/05/2025 12:06:15 م + manual changes		
Description	: aa		
Created	: 17/07/2023 02:47:53 ص	By	: Administrator
Modified	: 20/05/2025 12:06:15 م	By	: Administrator

Column	:	Detection	:
Mobile Phase	:	Temperature	:
Flow Rate	:	Pressure	:
Note	:		

Autostop	: None	External Start	: Start Only, Down
Subtraction Chromatogram	: (None)	Matching	: Scale Subtraction Chromatogram

Base	: Not Used	Calibration File	: None	Calculation	: Uncal
Scale Factor	: Not Used	Units After Scaling	: Not Used	Uncal. Response	: 0
Unretained Time	: 0.00 min	Column Length	: 50.00 mm	Column Calc.	: From Width at 50% of Height
Result Table Reports	: All Peaks	Hide ISTD Peak	: Enabled		
Method User Variables:					
MethodUserVar1	: 0	MethodUserVar2	: 0	MethodUserVar3	: 0

HPLC chromatogram info of compound 12b.

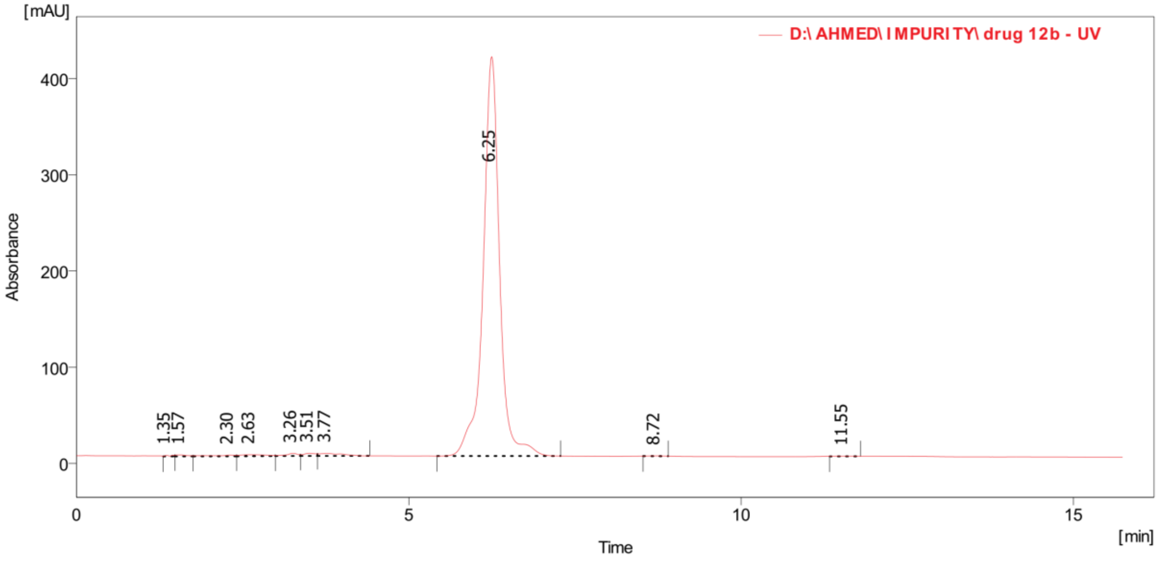

Result Table (Uncal - D:\AHMED\IMPURITY\drug 12b - UV)

	Reten. Time [min]	Area [mAU.s]	Area [%]	Compound Name
1	1.353	7.808	0.1	
2	1.572	16.830	0.2	
3	2.303	27.765	0.4	
4	2.627	39.575	0.5	
5	3.257	34.489	0.5	
6	3.507	35.061	0.5	
7	3.768	65.284	0.9	
8	6.247	7145.063	96.8	
9	8.720	5.176	0.1	
10	11.547	2.464	0.0	
	Total	7379.516	100.0	

HPLC chromatogram of compound 12b.

20/05/2025 02:22 م Chromatogram D:\AHMED\IMPURITY\drug 12c.prm Page 1 of 2

Sohag University
CENTRAL LAB
DR MOHAMED GAMAL

Chromatogram Info:

File Name	: D:\AHMED\IMPURITY\drug 12c.prm	File Created	: 20/05/2025 01:02:16 م
Origin	: Acquired, Acquisition started 20/05/2025 12:46:54 م	Acquired Date	: 20/05/2025 01:02:15 م
Original Project	: HPLC	By	: Administrator

Printed Version Info:

Printed Version	: - #2; 20/05/2025 02:22:27 م, IA: 8.0 Rev.3	Printed Date	: 20/05/2025 02:22:42 م
Report Style	: D:\HPLC\Common\Chromatogram.sty	By	: Administrator
Calibration File	: None		
Project	: HPLC		

Sample Description:

Sample ID	: drug 12c
Sample	:

Sample Parameters:

Amount	: 0.0	Dilution	: 1.0
Inj. Volume [µL]	: 20.0		
ISTD1 Amount	: 0.0	ISTD2 Amount	: 0.0
ISTD3 Amount	: 0.0	ISTD4 Amount	: 0.0
ISTD5 Amount	: 0.0	ISTD6 Amount	: 0.0
ISTD7 Amount	: 0.0	ISTD8 Amount	: 0.0
ISTD9 Amount	: 0.0	ISTD10 Amount	: 0.0

Analysis User Variables:		Method User Variables:	
AnalysisUserVar1	: 0	MethodUserVar1	: 0
AnalysisUserVar2	: 0	MethodUserVar2	: 0
AnalysisUserVar3	: 0	MethodUserVar3	: 0

Acquisition Method	: D:\ahmed\tolt\tolt separation - #199; 20/05/2025 12:28:36 م		
Description	: aa		
Created	: 17/07/2023 02:47:53 ص	By	: Administrator
Modified	: 20/05/2025 12:28:36 م	By	: Administrator

Processing Method	: D:\ahmed\tolt\tolt separation - #199; 20/05/2025 12:28:36 م + manual changes		
Description	: aa		
Created	: 17/07/2023 02:47:53 ص	By	: Administrator
Modified	: 20/05/2025 12:28:36 م	By	: Administrator

Column	:	Detection	:
Mobile Phase	:	Temperature	:
Flow Rate	:	Pressure	:
Note	:		

Autostop	: None	External Start	: Start Only, Down
Subtraction Chromatogram	: (None)	Matching	: Scale Subtraction Chromatogram

Base	: Not Used	Calibration File	: None	Calculation	: Uncal
Scale Factor	: Not Used	Units After Scaling	: Not Used	Uncal. Response	: 0
Unretained Time	: 0.00 min	Column Length	: 50.00 mm	Column Calc.	: From Width at 50% of Height
Result Table Reports	: All Peaks	Hide ISTD Peak	: Enabled		
Method User Variables:					
MethodUserVar1	: 0	MethodUserVar2	: 0	MethodUserVar3	: 0

HPLC chromatogram info of compound 12c.

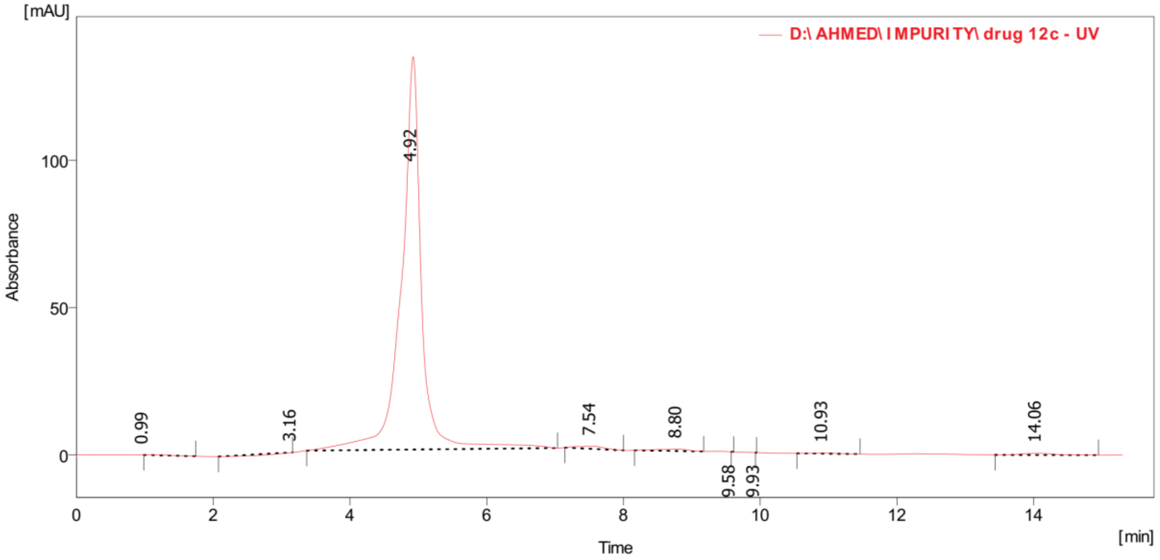

Result Table (Uncal - D:\AHMED\IMPURITY\drug 12c - UV)

	Reten. Time [min]	Area [mAU.s]	Area [%]	Compound Name
1	0.990	2.963	0.1	
2	3.158	15.296	0.5	
3	4.923	2733.891	96.9	
4	7.538	24.043	0.9	
5	8.798	19.588	0.7	
6	9.578	0.006	0.0	
7	9.933	0.001	0.0	
8	10.932	5.150	0.2	
9	14.058	19.777	0.7	
	Total	2820.716	100.0	

HPLC chromatogram of compound 12c.

Sohag University
CENTRAL LAB
DR MOHAMED GAMAL

Chromatogram Info:

File Name	: D:\AHMED\IMPURITY\drug 12d.prm	File Created	: 20/05/2025 01:33:49 م
Origin	: Acquired, Acquisition started 20/05/2025 01:04:01 م	Acquired Date	: 20/05/2025 01:33:49 م
Original Project	: HPLC	By	: Administrator

Printed Version Info:

Printed Version	: - #3; 20/05/2025 02:30:30 م, IA: 8.0 Rev.3	Printed Date	: 20/05/2025 02:30:30 م
Report Style	: D:\HPLC\Common\Chromatogram.sty	By	: Administrator
Calibration File	: None		
Project	: HPLC		

Sample Description:

Sample ID	: drug 12d
Sample	:

Sample Parameters:

Amount	: 0.0	Dilution	: 1.0
Inj. Volume [µL]	: 20.0		
ISTD1 Amount	: 0.0	ISTD2 Amount	: 0.0
ISTD3 Amount	: 0.0	ISTD4 Amount	: 0.0
ISTD5 Amount	: 0.0	ISTD6 Amount	: 0.0
ISTD7 Amount	: 0.0	ISTD8 Amount	: 0.0
ISTD9 Amount	: 0.0	ISTD10 Amount	: 0.0

Analysis User Variables:		Method User Variables:	
AnalysisUserVar1	: 0	MethodUserVar1	: 0
AnalysisUserVar2	: 0	MethodUserVar2	: 0
AnalysisUserVar3	: 0	MethodUserVar3	: 0

Acquisition Method	: D:\ahmed\tolt\tolt separation - #199; 20/05/2025 12:28:36 م		
Description	: aa		
Created	: 17/07/2023 02:47:53 ص	By	: Administrator
Modified	: 20/05/2025 12:28:36 م	By	: Administrator

Processing Method	: D:\ahmed\tolt\tolt separation - #199; 20/05/2025 12:28:36 م + manual changes		
Description	: aa		
Created	: 17/07/2023 02:47:53 ص	By	: Administrator
Modified	: 20/05/2025 12:28:36 م	By	: Administrator

Column	:	Detection	:
Mobile Phase	:	Temperature	:
Flow Rate	:	Pressure	:
Note	:		

Autostop	: None	External Start	: Start Only, Down
Subtraction Chromatogram	: (None)	Matching	: Scale Subtraction Chromatogram

Base	: Not Used	Calibration File	: None	Calculation	: Uncal
Scale Factor	: Not Used	Units After Scaling	: Not Used	Uncal. Response	: 0
Unretained Time	: 0.00 min	Column Length	: 50.00 mm	Column Calc.	: From Width at 50% of Height
Result Table Reports	: All Peaks	Hide ISTD Peak	: Enabled		
Method User Variables:					
MethodUserVar1	: 0	MethodUserVar2	: 0	MethodUserVar3	: 0

HPLC chromatogram info of compound 12d.

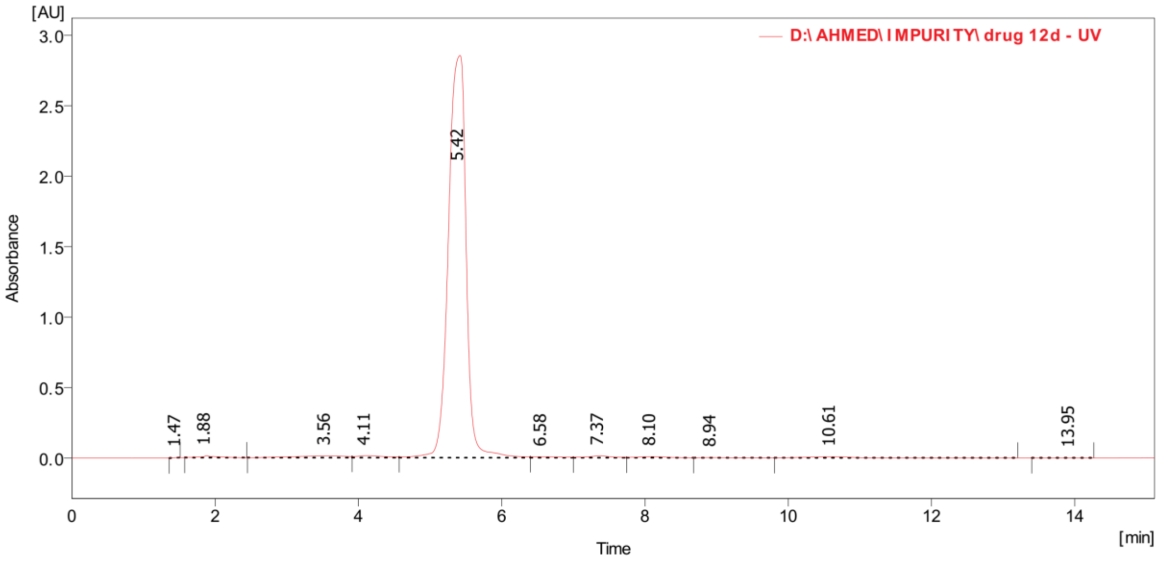

Result Table (Uncal - D:\AHMED\IMPURITY\drug 12d - UV)

	Reten. Time [min]	Area [mAU.s]	Area [%]	Compound Name
1	1.468	10.360	0.0	
2	1.880	180.709	0.4	
3	3.555	608.100	1.2	
4	4.113	379.706	0.7	
5	5.417	49152.371	95.4	
6	6.575	204.325	0.4	
7	7.372	312.528	0.6	
8	8.098	238.679	0.5	
9	8.940	80.135	0.2	
10	10.607	339.968	0.7	
11	13.945	6.593	0.0	
	Total	51513.472	100.0	

HPLC chromatogram of compound 12d.

Sohag University
CENTRAL LAB
DR MOHAMED GAMAL

Chromatogram Info:

File Name	: D:\AHMED\IMPURITY\drug 12e.prm	File Created	: 20/05/2025 02:06:51 م
Origin	: Acquired, Acquisition started 20/05/2025 01:50:51 م	Acquired Date	: 20/05/2025 02:06:50 م
Original Project	: HPLC	By	: Administrator

Printed Version Info:

Printed Version	: - #2; 20/05/2025 02:49:12 م, IA: 8.0 Rev.3	Printed Date	: 20/05/2025 02:49:27 م
Report Style	: D:\HPLC\Common\Chromatogram.sty	By	: Administrator
Calibration File	: None		
Project	: HPLC		

Sample Description:

Sample ID	: drug 12e
Sample	:

Sample Parameters:

Amount	: 0.0	Dilution	: 1.0
Inj. Volume [µL]	: 20.0		
ISTD1 Amount	: 0.0	ISTD2 Amount	: 0.0
ISTD3 Amount	: 0.0	ISTD4 Amount	: 0.0
ISTD5 Amount	: 0.0	ISTD6 Amount	: 0.0
ISTD7 Amount	: 0.0	ISTD8 Amount	: 0.0
ISTD9 Amount	: 0.0	ISTD10 Amount	: 0.0

Analysis User Variables:		Method User Variables:	
AnalysisUserVar1	: 0	MethodUserVar1	: 0
AnalysisUserVar2	: 0	MethodUserVar2	: 0
AnalysisUserVar3	: 0	MethodUserVar3	: 0

Acquisition Method	: D:\ahmed\tolt\tolt separation - #199; 20/05/2025 12:28:36 م		
Description	: aa		
Created	: 17/07/2023 02:47:53 ص	By	: Administrator
Modified	: 20/05/2025 12:28:36 م	By	: Administrator

Processing Method	: D:\ahmed\tolt\tolt separation - #199; 20/05/2025 12:28:36 م + manual changes		
Description	: aa		
Created	: 17/07/2023 02:47:53 ص	By	: Administrator
Modified	: 20/05/2025 12:28:36 م	By	: Administrator

Column	:	Detection	:
Mobile Phase	:	Temperature	:
Flow Rate	:	Pressure	:
Note	:		

Autostop	: None	External Start	: Start Only, Down
Subtraction Chromatogram	: (None)	Matching	: Scale Subtraction Chromatogram

Base	: Not Used	Calibration File	: None	Calculation	: Uncal
Scale Factor	: Not Used	Units After Scaling	: Not Used	Uncal. Response	: 0
Unretained Time	: 0.00 min	Column Length	: 50.00 mm	Column Calc.	: From Width at 50% of Height
Result Table Reports	: All Peaks	Hide ISTD Peak	: Enabled		
Method User Variables:					
MethodUserVar1	: 0	MethodUserVar2	: 0	MethodUserVar3	: 0

HPLC chromatogram info of compound 12e.

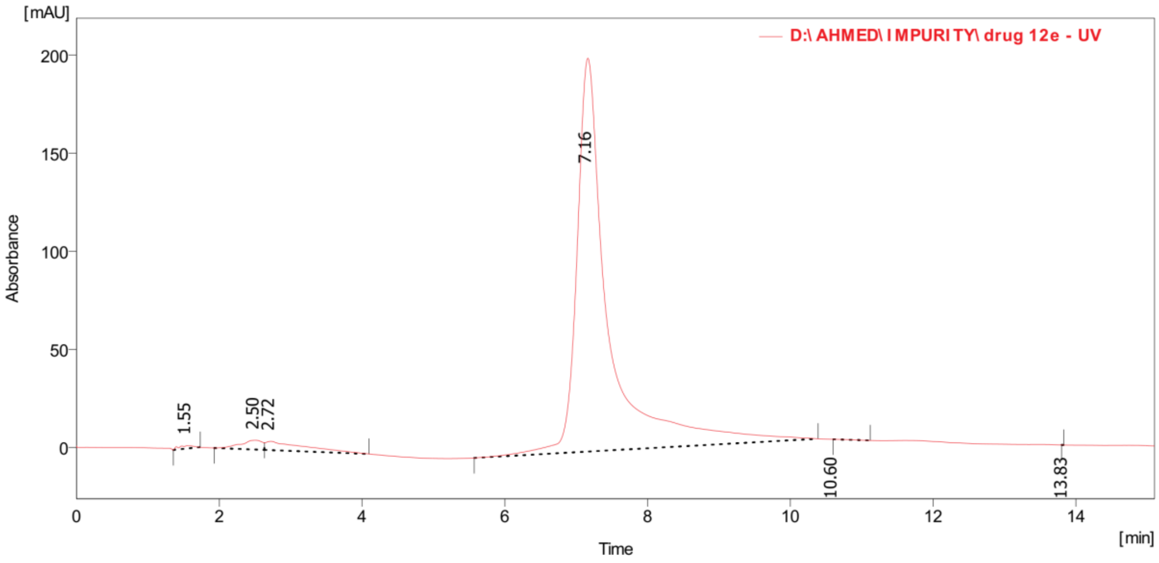

Result Table (Uncal - D:\AHMED\IMPURITY\drug 12e - UV)

	Reten. Time [min]	Area [mAU.s]	Area [%]	Compound Name
1	1.547	21.679	0.3	
2	2.505	101.392	1.6	
3	2.722	181.810	2.8	
4	7.163	6213.972	95.3	
5	10.602	1.340	0.0	
6	13.827	0.002	0.0	
	Total	6520.196	100.0	

HPLC chromatogram of compound 12e.

Sohag University
CENTRAL LAB
DR MOHAMED GAMAL

Chromatogram Info:

File Name	: D:\AHMED\IMPURITY\drug 12f.prm	File Created	: 20/05/2025 02:22:49 م
Origin	: Acquired, Acquisition started 20/05/2025 02:07:35 م	Acquired Date	: 20/05/2025 02:22:49 م
Original Project	: HPLC	By	: Administrator

Printed Version Info:

Printed Version	: - #2; 20/05/2025 02:50:54 م, IA: 8.0 Rev.3	Printed Date	: 20/05/2025 02:51:14 م
Report Style	: D:\HPLC\Common\Chromatogram.sty	By	: Administrator
Calibration File	: None		
Project	: HPLC		

Sample Description:

Sample ID	: drug 12f
Sample	:

Sample Parameters:

Amount	: 0.0	Dilution	: 1.0
Inj. Volume [µL]	: 20.0		
ISTD1 Amount	: 0.0	ISTD2 Amount	: 0.0
ISTD3 Amount	: 0.0	ISTD4 Amount	: 0.0
ISTD5 Amount	: 0.0	ISTD6 Amount	: 0.0
ISTD7 Amount	: 0.0	ISTD8 Amount	: 0.0
ISTD9 Amount	: 0.0	ISTD10 Amount	: 0.0

Analysis User Variables:		Method User Variables:	
AnalysisUserVar1	: 0	MethodUserVar1	: 0
AnalysisUserVar2	: 0	MethodUserVar2	: 0
AnalysisUserVar3	: 0	MethodUserVar3	: 0

Acquisition Method	: D:\ahmed\tolt\tolt separation - #199; 20/05/2025 12:28:36 م		
Description	: aa		
Created	: 17/07/2023 02:47:53 ص	By	: Administrator
Modified	: 20/05/2025 12:28:36 م	By	: Administrator

Processing Method	: D:\ahmed\tolt\tolt separation - #199; 20/05/2025 12:28:36 م + manual changes		
Description	: aa		
Created	: 17/07/2023 02:47:53 ص	By	: Administrator
Modified	: 20/05/2025 12:28:36 م	By	: Administrator

Column	:	Detection	:
Mobile Phase	:	Temperature	:
Flow Rate	:	Pressure	:
Note	:		

Autostop	: None	External Start	: Start Only, Down
Subtraction Chromatogram	: (None)	Matching	: Scale Subtraction Chromatogram

Base	: Not Used	Calibration File	: None	Calculation	: Uncal
Scale Factor	: Not Used	Units After Scaling	: Not Used	Uncal. Response	: 0
Unretained Time	: 0.00 min	Column Length	: 50.00 mm	Column Calc.	: From Width at 50% of Height
Result Table Reports	: All Peaks	Hide ISTD Peak	: Enabled		
Method User Variables:					
MethodUserVar1	: 0	MethodUserVar2	: 0	MethodUserVar3	: 0

HPLC chromatogram info of compound 12f.

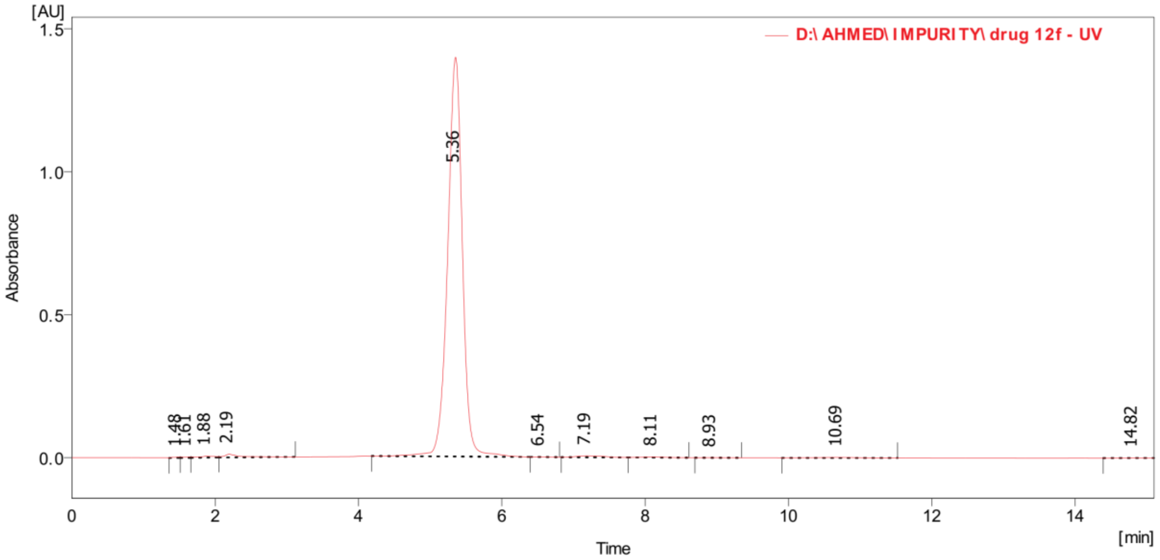

Result Table (Uncal - D:\AHMED\IMPURITY\drug 12f - UV)

	Reten. Time [min]	Area [mAU.s]	Area [%]	Compound Name
1	1.475	18.840	0.1	
2	1.613	21.699	0.1	
3	1.882	81.678	0.4	
4	2.195	179.763	0.9	
5	5.355	19977.900	97.2	
6	6.540	9.592	0.0	
7	7.187	135.834	0.7	
8	8.110	51.459	0.3	
9	8.932	6.941	0.0	
10	10.693	59.565	0.3	
11	14.817	6.491	0.0	
	Total	20549.762	100.0	

HPLC chromatogram of compound 12f.

Sohag University
CENTRAL LAB
DR MOHAMED GAMAL

Chromatogram Info:

File Name	: D:\ahmed\impurity\drug 12g.prm	File Created	: 20/05/2025 02:43:55 م
Origin	: Acquired, Acquisition started 20/05/2025 02:23:40 م	Acquired Date	: 20/05/2025 02:43:55 م
Original Project	: HPLC	By	: Administrator

Printed Version Info:

Printed Version	: - #2; 20/05/2025 02:46:53 م, IA: 8.0 Rev.3	Printed Date	: 20/05/2025 02:47:09 م
Report Style	: D:\HPLC\Common\Chromatogram.sty	By	: Administrator
Calibration File	: None		
Project	: HPLC		

Sample Description:

Sample ID	: drug 12g
Sample	:

Sample Parameters:

Amount	: 0.0	Dilution	: 1.0
Inj. Volume [µL]	: 20.0		
ISTD1 Amount	: 0.0	ISTD2 Amount	: 0.0
ISTD3 Amount	: 0.0	ISTD4 Amount	: 0.0
ISTD5 Amount	: 0.0	ISTD6 Amount	: 0.0
ISTD7 Amount	: 0.0	ISTD8 Amount	: 0.0
ISTD9 Amount	: 0.0	ISTD10 Amount	: 0.0

Analysis User Variables:		Method User Variables:	
AnalysisUserVar1	: 0	MethodUserVar1	: 0
AnalysisUserVar2	: 0	MethodUserVar2	: 0
AnalysisUserVar3	: 0	MethodUserVar3	: 0

Acquisition Method	: D:\ahmed\tolt\tolt separation - #199; 20/05/2025 12:28:36 م		
Description	: aa		
Created	: 17/07/2023 02:47:53 ص	By	: Administrator
Modified	: 20/05/2025 12:28:36 م	By	: Administrator

Processing Method	: D:\ahmed\tolt\tolt separation - #199; 20/05/2025 12:28:36 م + manual changes		
Description	: aa		
Created	: 17/07/2023 02:47:53 ص	By	: Administrator
Modified	: 20/05/2025 12:28:36 م	By	: Administrator

Column	:	Detection	:
Mobile Phase	:	Temperature	:
Flow Rate	:	Pressure	:
Note	:		

Autostop	: None	External Start	: Start Only, Down
Subtraction Chromatogram	: (None)	Matching	: Scale Subtraction Chromatogram

Base	: Not Used	Calibration File	: None	Calculation	: Uncal
Scale Factor	: Not Used	Units After Scaling	: Not Used	Uncal. Response	: 0
Unretained Time	: 0.00 min	Column Length	: 50.00 mm	Column Calc.	: From Width at 50% of Height
Result Table Reports	: All Peaks	Hide ISTD Peak	: Enabled		
Method User Variables:					
MethodUserVar1	: 0	MethodUserVar2	: 0	MethodUserVar3	: 0

HPLC chromatogram info of compound 12g.

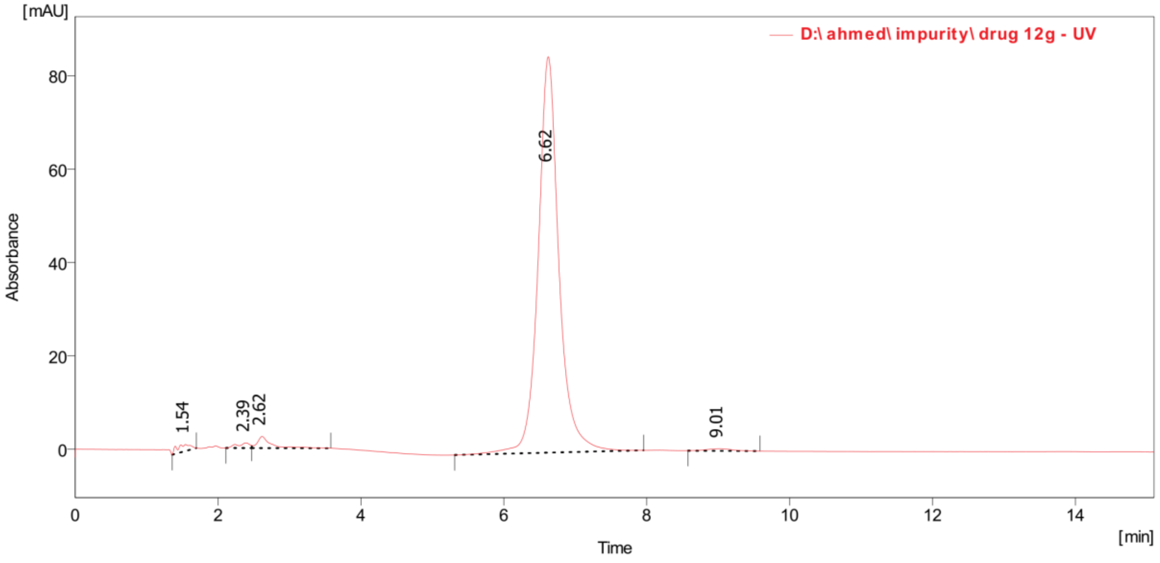

Result Table (Uncal - D: \ahmed\impurity\drug 12g - UV)

	Reten. Time [min]	Area [mAU.s]	Area [%]	Compound Name
1	1.543	19.604	1.1	
2	2.388	13.222	0.7	
3	2.615	33.588	1.9	
4	6.622	1718.074	95.7	
5	9.013	11.449	0.6	
	Total	1795.937	100.0	

HPLC chromatogram of compound 12g.

Sohag University
CENTRAL LAB
DR MOHAMED GAMAL

Chromatogram Info:

File Name	: D:\AHMED\IMPURITY\drug 12h.prm	File Created	: 20/05/2025 01:50:16 م
Origin	: Acquired, Acquisition started 20/05/2025 01:35:04 م	Acquired Date	: 20/05/2025 01:50:16 م
Original Project	: HPLC	By	: Administrator

Printed Version Info:

Printed Version	: - #2; 20/05/2025 02:34:42 م, IA: 8.0 Rev.3	Printed Date	: 20/05/2025 02:36:39 م
Report Style	: D:\HPLC\Common\Chromatogram.sty	By	: Administrator
Calibration File	: None		
Project	: HPLC		

Sample Description:

Sample ID	: drug 12h
Sample	:

Sample Parameters:

Amount	: 0.0	Dilution	: 1.0
Inj. Volume [µL]	: 20.0		
ISTD1 Amount	: 0.0	ISTD2 Amount	: 0.0
ISTD3 Amount	: 0.0	ISTD4 Amount	: 0.0
ISTD5 Amount	: 0.0	ISTD6 Amount	: 0.0
ISTD7 Amount	: 0.0	ISTD8 Amount	: 0.0
ISTD9 Amount	: 0.0	ISTD10 Amount	: 0.0

Analysis User Variables:		Method User Variables:	
AnalysisUserVar1	: 0	MethodUserVar1	: 0
AnalysisUserVar2	: 0	MethodUserVar2	: 0
AnalysisUserVar3	: 0	MethodUserVar3	: 0

Acquisition Method	: D:\ahmed\tolt\tolt separation - #199; 20/05/2025 12:28:36 م		
Description	: aa		
Created	: 17/07/2023 02:47:53 ص	By	: Administrator
Modified	: 20/05/2025 12:28:36 م	By	: Administrator

Processing Method	: D:\ahmed\tolt\tolt separation - #199; 20/05/2025 12:28:36 م + manual changes		
Description	: aa		
Created	: 17/07/2023 02:47:53 ص	By	: Administrator
Modified	: 20/05/2025 12:28:36 م	By	: Administrator

Column	:	Detection	:
Mobile Phase	:	Temperature	:
Flow Rate	:	Pressure	:
Note	:		

Autostop	: None	External Start	: Start Only, Down
Subtraction Chromatogram	: (None)	Matching	: Scale Subtraction Chromatogram

Base	: Not Used	Calibration File	: None	Calculation	: Uncal
Scale Factor	: Not Used	Units After Scaling	: Not Used	Uncal. Response	: 0
Unretained Time	: 0.00 min	Column Length	: 50.00 mm	Column Calc.	: From Width at 50% of Height
Result Table Reports	: All Peaks	Hide ISTD Peak	: Enabled		
Method User Variables:					
MethodUserVar1	: 0	MethodUserVar2	: 0	MethodUserVar3	: 0

\

HPLC chromatogram info of compound 12h.

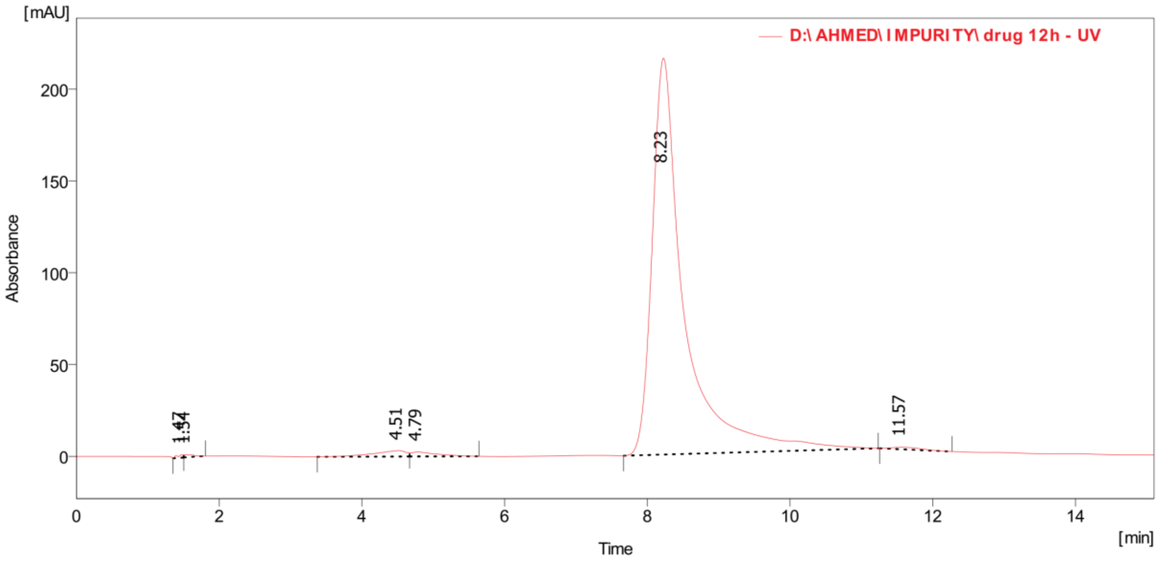

Result Table (Uncal - D:\AHMED\IMPURITY\drug 12h - UV)

	Reten. Time [min]	Area [mAU.s]	Area [%]	Compound Name
1	1.473	9.427	0.1	
2	1.540	13.139	0.2	
3	4.515	102.031	1.5	
4	4.785	63.254	0.9	
5	8.227	6740.071	96.8	
6	11.565	33.247	0.5	
	Total	6961.167	100.0	

HPLC chromatogram of compound 12h.

Sohag University
CENTRAL LAB
DR MOHAMED GAMAL

Chromatogram Info:

File Name	: D:\ahmed\impurity\drug 12i.prm	File Created	: 20/05/2025 03:00:54 م
Origin	: Acquired, Acquisition started 20/05/2025 02:45:47 م	Acquired Date	: 20/05/2025 03:00:53 م
Original Project	: HPLC	By	: Administrator

Printed Version Info:

Printed Version	: - #2; 20/05/2025 03:01:49 م, IA: 8.0 Rev.3	Printed Date	: 20/05/2025 03:02:04 م
Report Style	: D:\HPLC\Common\Chromatogram.sty	By	: Administrator
Calibration File	: None		
Project	: HPLC		

Sample Description:

Sample ID	: drug 12i
Sample	:

Sample Parameters:

Amount	: 0.0	Dilution	: 1.0
Inj. Volume [µL]	: 20.0		
ISTD1 Amount	: 0.0	ISTD2 Amount	: 0.0
ISTD3 Amount	: 0.0	ISTD4 Amount	: 0.0
ISTD5 Amount	: 0.0	ISTD6 Amount	: 0.0
ISTD7 Amount	: 0.0	ISTD8 Amount	: 0.0
ISTD9 Amount	: 0.0	ISTD10 Amount	: 0.0

Analysis User Variables:		Method User Variables:	
AnalysisUserVar1	: 0	MethodUserVar1	: 0
AnalysisUserVar2	: 0	MethodUserVar2	: 0
AnalysisUserVar3	: 0	MethodUserVar3	: 0

Acquisition Method	: D:\ahmed\tolt\tolt separation - #199; 20/05/2025 12:28:36 م		
Description	: aa		
Created	: 17/07/2023 02:47:53 ص	By	: Administrator
Modified	: 20/05/2025 12:28:36 م	By	: Administrator

Processing Method	: D:\ahmed\tolt\tolt separation - #199; 20/05/2025 12:28:36 م + manual changes		
Description	: aa		
Created	: 17/07/2023 02:47:53 ص	By	: Administrator
Modified	: 20/05/2025 12:28:36 م	By	: Administrator

Column	:	Detection	:
Mobile Phase	:	Temperature	:
Flow Rate	:	Pressure	:
Note	:		

Autostop	: None	External Start	: Start Only, Down
Subtraction Chromatogram	: (None)	Matching	: Scale Subtraction Chromatogram

Base	: Not Used	Calibration File	: None	Calculation	: Uncal
Scale Factor	: Not Used	Units After Scaling	: Not Used	Uncal. Response	: 0
Unretained Time	: 0.00 min	Column Length	: 50.00 mm	Column Calc.	: From Width at 50% of Height
Result Table Reports	: All Peaks	Hide ISTD Peak	: Enabled		
Method User Variables:					
MethodUserVar1	: 0	MethodUserVar2	: 0	MethodUserVar3	: 0

HPLC chromatogram info of compound 12i.

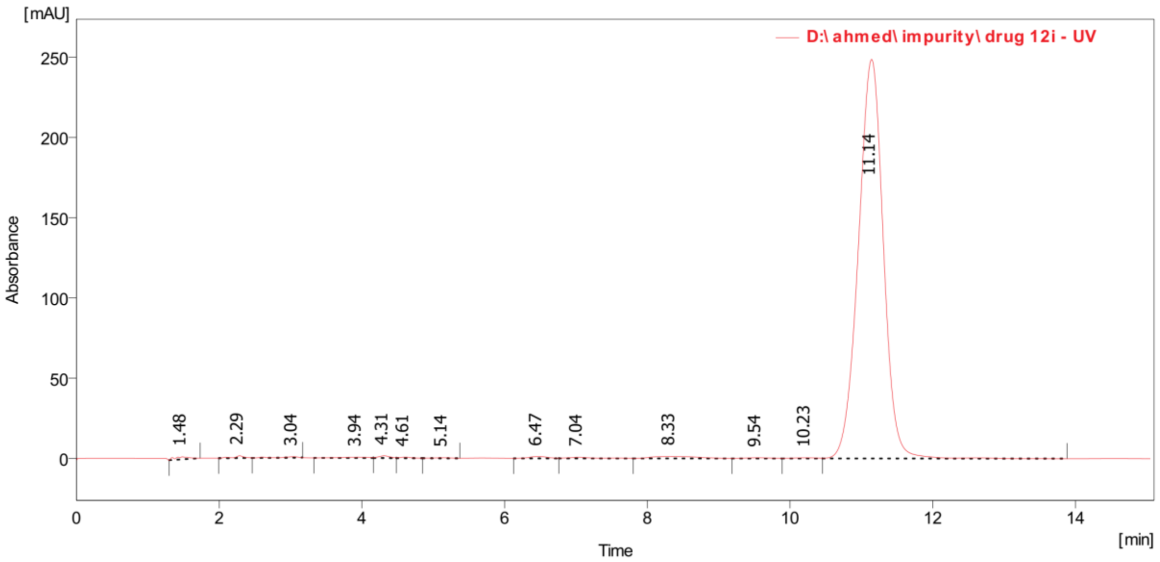

Result Table (Uncal - D:\ahmed\impurity\drug 12i - UV)

	Reten. Time [min]	Area [mAU.s]	Area [%]	Compound Name
1	1.483	22.251	0.4	
2	2.285	14.525	0.2	
3	3.040	10.982	0.2	
4	3.937	14.415	0.2	
5	4.312	15.538	0.2	
6	4.605	6.737	0.1	
7	5.142	4.814	0.1	
8	6.470	20.589	0.3	
9	7.035	17.227	0.3	
10	8.333	56.802	0.9	
11	9.538	11.067	0.2	
12	10.228	10.346	0.2	
13	11.143	6043.403	96.7	
	Total	6248.696	100.0	

HPLC chromatogram of compound 12i.

Sohag University
CENTRAL LAB
DR MOHAMED GAMAL

Chromatogram Info:

File Name	: D:\ahmed\impurity\drug 13a.prm	File Created	: 22/05/2025 12:11:10 م
Origin	: Acquired, Acquisition started 22/05/2025 11:56:04 ص	Acquired Date	: 22/05/2025 12:11:10 م
Original Project	: HPLC	By	: Administrator

Printed Version Info:

Printed Version	: - #2; 22/05/2025 12:15:32 م, IA: 8.0 Rev.3	Printed Date	: 22/05/2025 12:15:43 م
Report Style	: D:\HPLC\Common\Chromatogram.sty	By	: Administrator
Calibration File	: None		
Project	: HPLC		

Sample Description:

Sample ID	: drug 13a
Sample	:

Sample Parameters:

Amount	: 0.0	Dilution	: 1.0
Inj. Volume [µL]	: 20.0		
ISTD1 Amount	: 0.0	ISTD2 Amount	: 0.0
ISTD3 Amount	: 0.0	ISTD4 Amount	: 0.0
ISTD5 Amount	: 0.0	ISTD6 Amount	: 0.0
ISTD7 Amount	: 0.0	ISTD8 Amount	: 0.0
ISTD9 Amount	: 0.0	ISTD10 Amount	: 0.0

Analysis User Variables:		Method User Variables:	
AnalysisUserVar1	: 0	MethodUserVar1	: 0
AnalysisUserVar2	: 0	MethodUserVar2	: 0
AnalysisUserVar3	: 0	MethodUserVar3	: 0

Acquisition Method	: D:\ahmed\tolt\tolt separation - #201; 22/05/2025 11:09:38 ص		
Description	: aa		
Created	: 17/07/2023 02:47:53 ص	By	: Administrator
Modified	: 22/05/2025 11:09:38 ص	By	: Administrator

Processing Method	: D:\ahmed\tolt\tolt separation - #201; 22/05/2025 11:09:38 ص + manual changes		
Description	: aa		
Created	: 17/07/2023 02:47:53 ص	By	: Administrator
Modified	: 22/05/2025 11:09:38 ص	By	: Administrator

Column	:	Detection	:
Mobile Phase	:	Temperature	:
Flow Rate	:	Pressure	:
Note	:		

Autostop	: None	External Start	: Start Only, Down
Subtraction Chromatogram	: (None)	Matching	: Scale Subtraction Chromatogram

Base	: Not Used	Calibration File	: None	Calculation	: Uncal
Scale Factor	: Not Used	Units After Scaling	: Not Used	Uncal. Response	: 0
Unretained Time	: 0.00 min	Column Length	: 50.00 mm	Column Calc.	: From Width at 50% of Height
Result Table Reports	: All Peaks	Hide ISTD Peak	: Enabled		
Method User Variables:					
MethodUserVar1	: 0	MethodUserVar2	: 0	MethodUserVar3	: 0

HPLC chromatogram info of compound 13a.

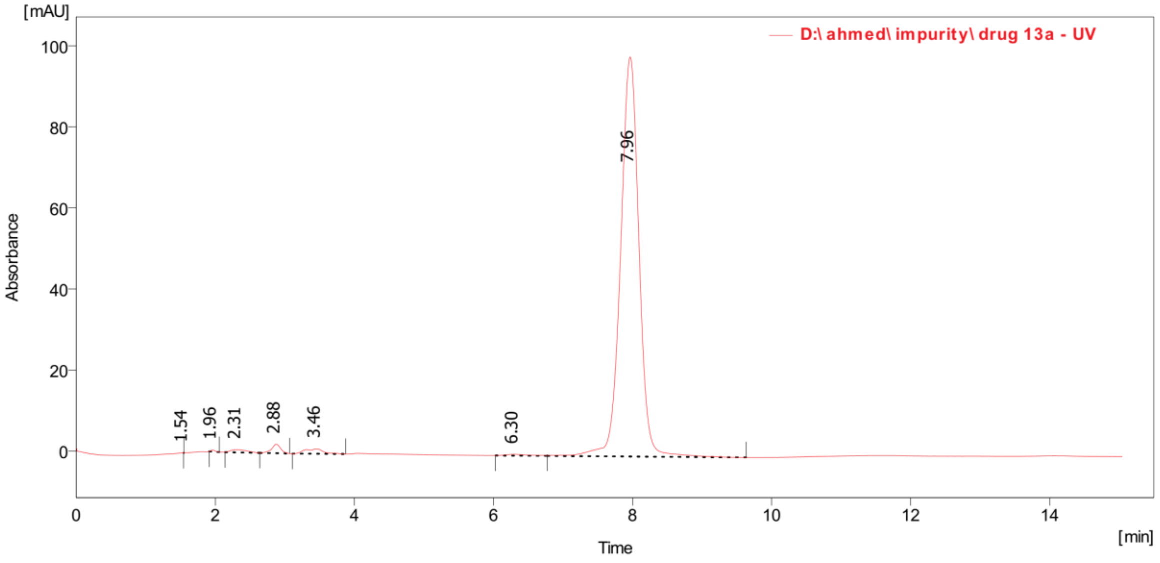

Result Table (Uncal - D: \ahmed\impurity\drug 13a - UV)

	Reten. Time [min]	Area [mAU.s]	Area [%]	Compound Name
1	1.545	0.000	0.0	
2	1.960	1.641	0.1	
3	2.313	11.102	0.6	
4	2.877	20.105	1.1	
5	3.458	22.799	1.2	
6	6.300	8.201	0.4	
7	7.965	1819.245	96.6	
	Total	1883.093	100.0	

HPLC chromatogram of compound 13a.

Sohag University
CENTRAL LAB
DR MOHAMED GAMAL

Chromatogram Info:

File Name	: D:\ahmed\impurity\drug 13b.prm	File Created	: 22/05/2025 12:26:21 م
Origin	: Acquired, Acquisition started 22/05/2025 12:11:13 م	Acquired Date	: 22/05/2025 12:26:21 م
Original Project	: HPLC	By	: Administrator

Printed Version Info:

Printed Version	: - #3; 22/05/2025 12:29:54 م, IA: 8.0 Rev.3	Printed Date	: 22/05/2025 12:30:07 م
Report Style	: D:\HPLC\Common\Chromatogram.sty	By	: Administrator
Calibration File	: None		
Project	: HPLC		

Sample Description:

Sample ID	: drug 13b
Sample	:

Sample Parameters:

Amount	: 0.0	Dilution	: 1.0
Inj. Volume [µL]	: 20.0		
ISTD1 Amount	: 0.0	ISTD2 Amount	: 0.0
ISTD3 Amount	: 0.0	ISTD4 Amount	: 0.0
ISTD5 Amount	: 0.0	ISTD6 Amount	: 0.0
ISTD7 Amount	: 0.0	ISTD8 Amount	: 0.0
ISTD9 Amount	: 0.0	ISTD10 Amount	: 0.0

Analysis User Variables:		Method User Variables:	
AnalysisUserVar1	: 0	MethodUserVar1	: 0
AnalysisUserVar2	: 0	MethodUserVar2	: 0
AnalysisUserVar3	: 0	MethodUserVar3	: 0

Acquisition Method	: D:\ahmed\tolt\tolt separation - #201; 22/05/2025 11:09:38 ص		
Description	: aa		
Created	: 17/07/2023 02:47:53 ص	By	: Administrator
Modified	: 22/05/2025 11:09:38 ص	By	: Administrator

Processing Method	: D:\ahmed\tolt\tolt separation - #201; 22/05/2025 11:09:38 ص + manual changes		
Description	: aa		
Created	: 17/07/2023 02:47:53 ص	By	: Administrator
Modified	: 22/05/2025 11:09:38 ص	By	: Administrator

Column	:	Detection	:
Mobile Phase	:	Temperature	:
Flow Rate	:	Pressure	:
Note	:		

Autostop	: None	External Start	: Start Only, Down
Subtraction Chromatogram	: (None)	Matching	: Scale Subtraction Chromatogram

Base	: Not Used	Calibration File	: None	Calculation	: Uncal
Scale Factor	: Not Used	Units After Scaling	: Not Used	Uncal. Response	: 0
Unretained Time	: 0.00 min	Column Length	: 50.00 mm	Column Calc.	: From Width at 50% of Height
Result Table Reports	: All Peaks	Hide ISTD Peak	: Enabled		
Method User Variables:					
MethodUserVar1	: 0	MethodUserVar2	: 0	MethodUserVar3	: 0

HPLC chromatogram info of compound 13b.

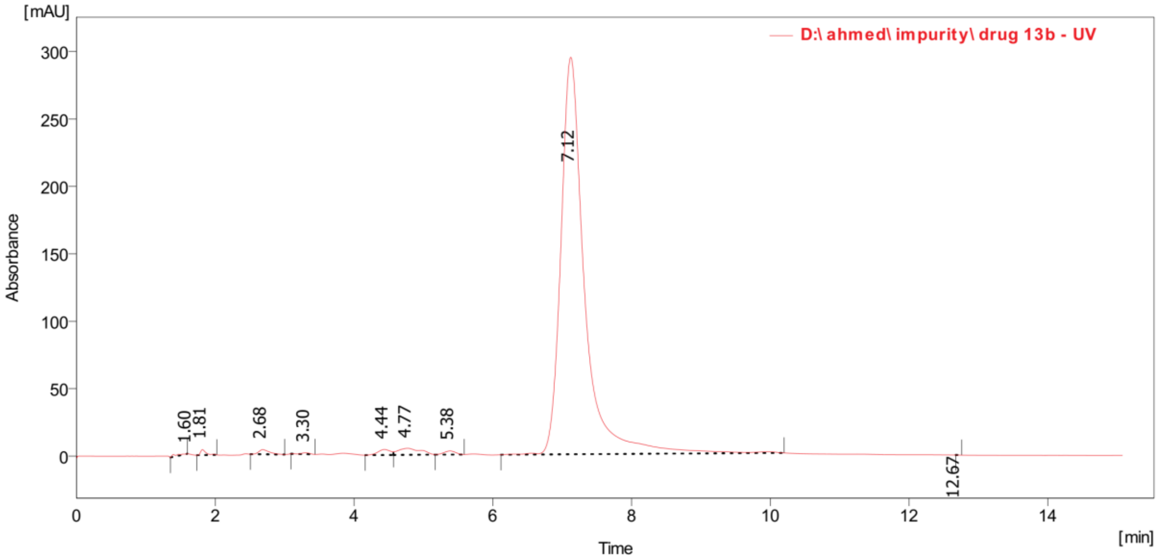

Result Table (Uncal - D: \ahmed\impurity\drug 13b - UV)

	Reten. Time [min]	Area [mAU.s]	Area [%]	Compound Name
1	1.597	9.716	0.1	
2	1.815	21.689	0.3	
3	2.683	36.858	0.5	
4	3.303	8.821	0.1	
5	4.438	51.197	0.7	
6	4.772	110.834	1.5	
7	5.383	31.270	0.4	
8	7.123	6995.840	96.3	
9	12.673	0.028	0.0	
	Total	7266.253	100.0	

HPLC chromatogram of compound 13b.

Sohag University
CENTRAL LAB
DR MOHAMED GAMAL

Chromatogram Info:

File Name	: D:\AHMED\IMPURITY\drug 13c.prm	File Created	: 22/05/2025 12:41:32 م
Origin	: Acquired, Acquisition started 22/05/2025 12:26:23 م	Acquired Date	: 22/05/2025 12:41:32 م
Original Project	: HPLC	By	: Administrator

Printed Version Info:

Printed Version	: - #4; 22/05/2025 12:45:12 م, IA: 8.0 Rev.3	Printed Date	: 22/05/2025 12:45:32 م
Report Style	: D:\HPLC\Common\Chromatogram.sty	By	: Administrator
Calibration File	: None		
Project	: HPLC		

Sample Description:

Sample ID	: drug 13c
Sample	:

Sample Parameters:

Amount	: 0.0	Dilution	: 1.0
Inj. Volume [µL]	: 20.0		
ISTD1 Amount	: 0.0	ISTD2 Amount	: 0.0
ISTD3 Amount	: 0.0	ISTD4 Amount	: 0.0
ISTD5 Amount	: 0.0	ISTD6 Amount	: 0.0
ISTD7 Amount	: 0.0	ISTD8 Amount	: 0.0
ISTD9 Amount	: 0.0	ISTD10 Amount	: 0.0

Analysis User Variables:		Method User Variables:	
AnalysisUserVar1	: 0	MethodUserVar1	: 0
AnalysisUserVar2	: 0	MethodUserVar2	: 0
AnalysisUserVar3	: 0	MethodUserVar3	: 0

Acquisition Method	: D:\ahmed\tolt\tolt separation - #201; 22/05/2025 11:09:38 ص		
Description	: aa		
Created	: 17/07/2023 02:47:53 ص	By	: Administrator
Modified	: 22/05/2025 11:09:38 ص	By	: Administrator

Processing Method	: D:\ahmed\tolt\tolt separation - #201; 22/05/2025 11:09:38 ص + manual changes		
Description	: aa		
Created	: 17/07/2023 02:47:53 ص	By	: Administrator
Modified	: 22/05/2025 11:09:38 ص	By	: Administrator

Column	:	Detection	:
Mobile Phase	:	Temperature	:
Flow Rate	:	Pressure	:
Note	:		

Autostop	: None	External Start	: Start Only, Down
Subtraction Chromatogram	: (None)	Matching	: Scale Subtraction Chromatogram

Base	: Not Used	Calibration File	: None	Calculation	: Uncal
Scale Factor	: Not Used	Units After Scaling	: Not Used	Uncal. Response	: 0
Unretained Time	: 0.00 min	Column Length	: 50.00 mm	Column Calc.	: From Width at 50% of Height
Result Table Reports	: All Peaks	Hide ISTD Peak	: Enabled		
Method User Variables:					
MethodUserVar1	: 0	MethodUserVar2	: 0	MethodUserVar3	: 0

HPLC chromatogram info of compound 13c.

22/05/2025 12:45 م Chromatogram D:\AHMED\IMPURITY\drug 13c.prm Page 2 of 2

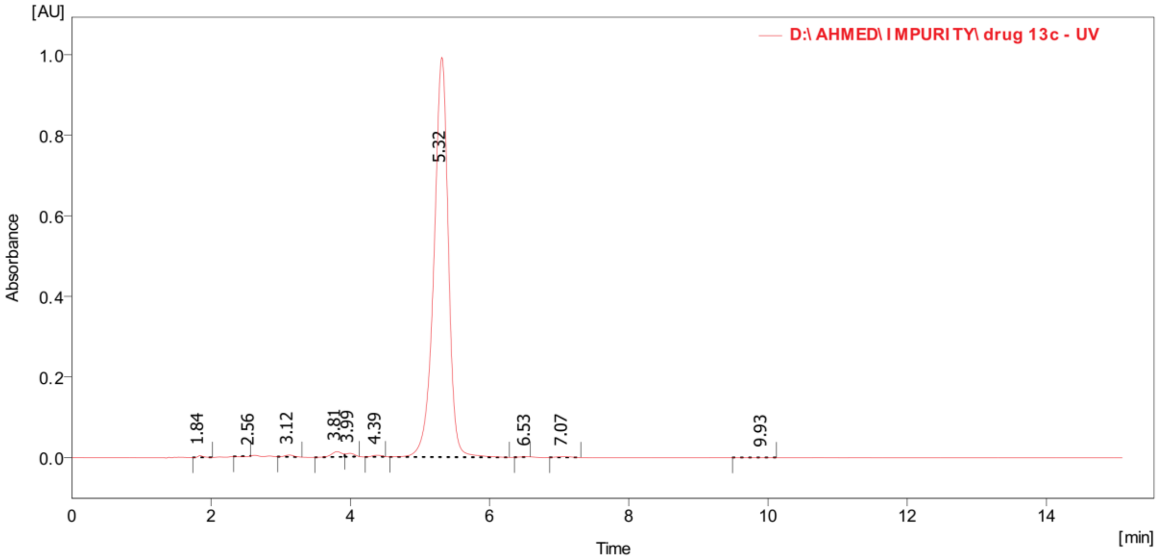

Result Table (Uncal - D:\AHMED\IMPURITY\drug 13c - UV)

	Reten. Time [min]	Area [mAU.s]	Area [%]	Compound Name
1	1.840	21.232	0.1	
2	2.565	15.111	0.1	
3	3.123	39.755	0.3	
4	3.808	134.938	0.9	
5	3.993	70.862	0.5	
6	4.387	34.325	0.2	
7	5.315	14319.356	97.6	
8	6.533	3.298	0.0	
9	7.067	24.302	0.2	
10	9.932	2.047	0.0	
	Total	14665.226	100.0	

HPLC chromatogram of compound 13c.

Sohag University
CENTRAL LAB
DR MOHAMED GAMAL

Chromatogram Info:

File Name	: D:\ahmed\impurity\drug 13d.prm	File Created	: 22/05/2025 12:56:49 م
Origin	: Acquired, Acquisition started 22/05/2025 12:41:47 م	Acquired Date	: 22/05/2025 12:56:49 م
Original Project	: HPLC	By	: Administrator

Printed Version Info:

Printed Version	: - #4; 22/05/2025 12:59:46 م, IA: 8.0 Rev.3	Printed Date	: 22/05/2025 01:00:20 م
Report Style	: D:\HPLC\Common\Chromatogram.sty	By	: Administrator
Calibration File	: None		
Project	: HPLC		

Sample Description:

Sample ID	: drug 13d
Sample	:

Sample Parameters:

Amount	: 0.0	Dilution	: 1.0
Inj. Volume [µL]	: 20.0		
ISTD1 Amount	: 0.0	ISTD2 Amount	: 0.0
ISTD3 Amount	: 0.0	ISTD4 Amount	: 0.0
ISTD5 Amount	: 0.0	ISTD6 Amount	: 0.0
ISTD7 Amount	: 0.0	ISTD8 Amount	: 0.0
ISTD9 Amount	: 0.0	ISTD10 Amount	: 0.0

Analysis User Variables:		Method User Variables:	
AnalysisUserVar1	: 0	MethodUserVar1	: 0
AnalysisUserVar2	: 0	MethodUserVar2	: 0
AnalysisUserVar3	: 0	MethodUserVar3	: 0

Acquisition Method	: D:\ahmed\tolt\tolt separation - #201; 22/05/2025 11:09:38 ص		
Description	: aa		
Created	: 17/07/2023 02:47:53 ص	By	: Administrator
Modified	: 22/05/2025 11:09:38 ص	By	: Administrator

Processing Method	: D:\ahmed\tolt\tolt separation - #201; 22/05/2025 11:09:38 ص + manual changes		
Description	: aa		
Created	: 17/07/2023 02:47:53 ص	By	: Administrator
Modified	: 22/05/2025 11:09:38 ص	By	: Administrator

Column	:	Detection	:
Mobile Phase	:	Temperature	:
Flow Rate	:	Pressure	:
Note	:		

Autostop	: None	External Start	: Start Only, Down
Subtraction Chromatogram	: (None)	Matching	: Scale Subtraction Chromatogram

Base	: Not Used	Calibration File	: None	Calculation	: Uncal
Scale Factor	: Not Used	Units After Scaling	: Not Used	Uncal. Response	: 0
Unretained Time	: 0.00 min	Column Length	: 50.00 mm	Column Calc.	: From Width at 50% of Height
Result Table Reports	: All Peaks	Hide ISTD Peak	: Enabled		
Method User Variables:					
MethodUserVar1	: 0	MethodUserVar2	: 0	MethodUserVar3	: 0

HPLC chromatogram info of compound 13d.

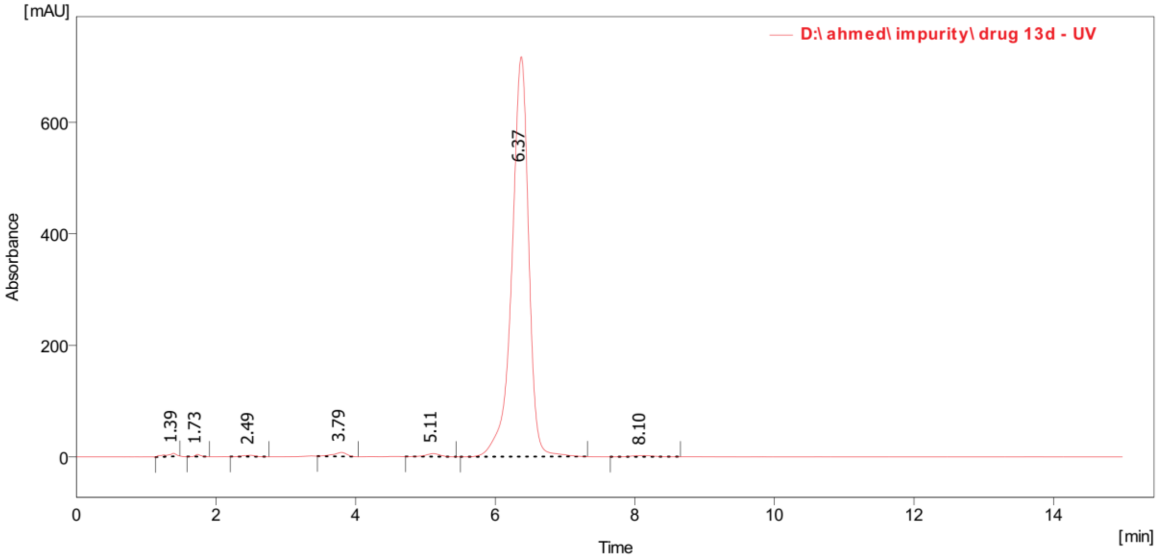

Result Table (Uncal - D: \ahmed\impurity\drug 13d - UV)

	Reten. Time [min]	Area [mAU.s]	Area [%]	Compound Name
1	1.390	58.308	0.5	
2	1.733	20.689	0.2	
3	2.492	30.023	0.2	
4	3.795	95.521	0.8	
5	5.112	75.312	0.6	
6	6.373	12022.840	97.4	
7	8.102	41.481	0.3	
	Total	12344.175	100.0	

HPLC chromatogram of compound 13d.

Sohag University
CENTRAL LAB
DR MOHAMED GAMAL

Chromatogram Info:

File Name	: D:\ahmed\impurity\drug 13e.prm	File Created	: 22/05/2025 01:12:02 م
Origin	: Acquired, Acquisition started 22/05/2025 12:56:53 م	Acquired Date	: 22/05/2025 01:12:02 م
Original Project	: HPLC	By	: Administrator

Printed Version Info:

Printed Version	: - #2; 22/05/2025 01:14:03 م, IA: 8.0 Rev.3	Printed Date	: 22/05/2025 01:14:19 م
Report Style	: D:\HPLC\Common\Chromatogram.sty	By	: Administrator
Calibration File	: None		
Project	: HPLC		

Sample Description:

Sample ID	: drug 13e
Sample	:

Sample Parameters:

Amount	: 0.0	Dilution	: 1.0
Inj. Volume [µL]	: 20.0		
ISTD1 Amount	: 0.0	ISTD2 Amount	: 0.0
ISTD3 Amount	: 0.0	ISTD4 Amount	: 0.0
ISTD5 Amount	: 0.0	ISTD6 Amount	: 0.0
ISTD7 Amount	: 0.0	ISTD8 Amount	: 0.0
ISTD9 Amount	: 0.0	ISTD10 Amount	: 0.0

Analysis User Variables:		Method User Variables:	
AnalysisUserVar1	: 0	MethodUserVar1	: 0
AnalysisUserVar2	: 0	MethodUserVar2	: 0
AnalysisUserVar3	: 0	MethodUserVar3	: 0

Acquisition Method	: D:\ahmed\tolt\tolt separation - #201; 22/05/2025 11:09:38 ص		
Description	: aa		
Created	: 17/07/2023 02:47:53 ص	By	: Administrator
Modified	: 22/05/2025 11:09:38 ص	By	: Administrator

Processing Method	: D:\ahmed\tolt\tolt separation - #201; 22/05/2025 11:09:38 ص + manual changes		
Description	: aa		
Created	: 17/07/2023 02:47:53 ص	By	: Administrator
Modified	: 22/05/2025 11:09:38 ص	By	: Administrator

Column	:	Detection	:
Mobile Phase	:	Temperature	:
Flow Rate	:	Pressure	:
Note	:		

Autostop	: None	External Start	: Start Only, Down
Subtraction Chromatogram	: (None)	Matching	: Scale Subtraction Chromatogram

Base	: Not Used	Calibration File	: None	Calculation	: Uncal
Scale Factor	: Not Used	Units After Scaling	: Not Used	Uncal. Response	: 0
Unretained Time	: 0.00 min	Column Length	: 50.00 mm	Column Calc.	: From Width at 50% of Height
Result Table Reports	: All Peaks	Hide ISTD Peak	: Enabled		
Method User Variables:					
MethodUserVar1	: 0	MethodUserVar2	: 0	MethodUserVar3	: 0

HPLC chromatogram info of compound 13e.

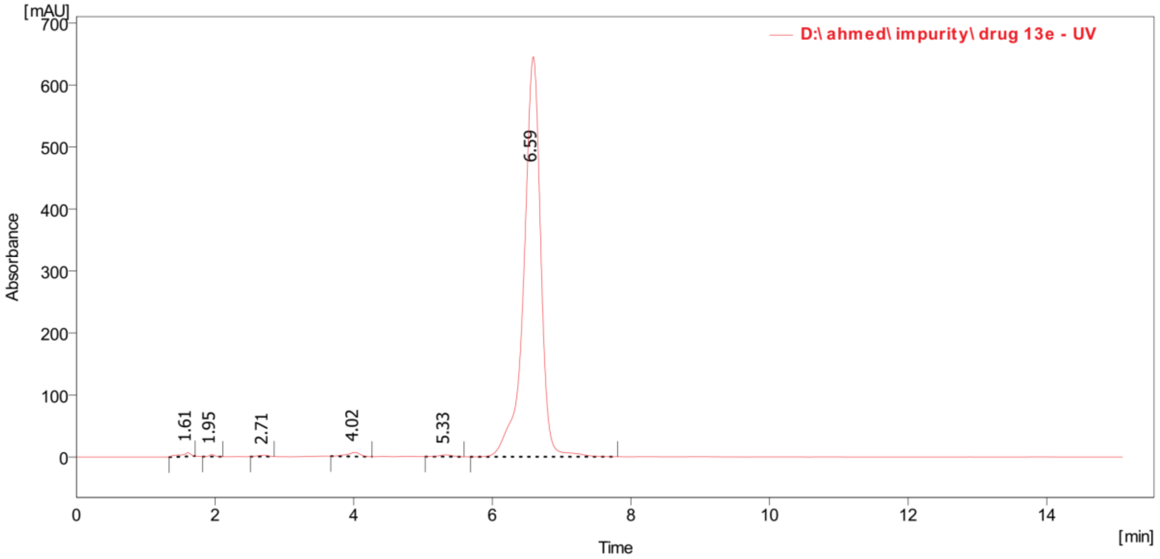

Result Table (Uncal - D:\ahmed\impurity\drug 13e - UV)

	Reten. Time [min]	Area [mAU.s]	Area [%]	Compound Name
1	1.605	64.085	0.6	
2	1.948	17.238	0.2	
3	2.712	22.359	0.2	
4	4.017	87.202	0.8	
5	5.330	36.741	0.3	
6	6.590	10969.564	98.0	
	Total	11197.189	100.0	

HPLC chromatogram of compound 13e.

Sohag University
CENTRAL LAB
DR MOHAMED GAMAL

Chromatogram Info:

File Name	: D:\ahmed\impurity\drug 13f.prm	File Created	: 22/05/2025 01:27:18 م
Origin	: Acquired, Acquisition started 22/05/2025 01:12:05 م	Acquired Date	: 22/05/2025 01:27:18 م
Original Project	: HPLC	By	: Administrator

Printed Version Info:

Printed Version	: - #3; 22/05/2025 01:30:41 م, IA: 8.0 Rev.3	Printed Date	: 22/05/2025 01:31:05 م
Report Style	: D:\HPLC\Common\Chromatogram.sty	By	: Administrator
Calibration File	: None		
Project	: HPLC		

Sample Description:

Sample ID	: drug 13f
Sample	:

Sample Parameters:

Amount	: 0.0	Dilution	: 1.0
Inj. Volume [μL]	: 20.0		
ISTD1 Amount	: 0.0	ISTD2 Amount	: 0.0
ISTD3 Amount	: 0.0	ISTD4 Amount	: 0.0
ISTD5 Amount	: 0.0	ISTD6 Amount	: 0.0
ISTD7 Amount	: 0.0	ISTD8 Amount	: 0.0
ISTD9 Amount	: 0.0	ISTD10 Amount	: 0.0

Analysis User Variables:		Method User Variables:	
AnalysisUserVar1	: 0	MethodUserVar1	: 0
AnalysisUserVar2	: 0	MethodUserVar2	: 0
AnalysisUserVar3	: 0	MethodUserVar3	: 0

Acquisition Method	: D:\ahmed\tolt\tolt separation - #201; 22/05/2025 11:09:38 ص		
Description	: aa		
Created	: 17/07/2023 02:47:53 ص	By	: Administrator
Modified	: 22/05/2025 11:09:38 ص	By	: Administrator

Processing Method	: D:\ahmed\tolt\tolt separation - #201; 22/05/2025 11:09:38 ص + manual changes		
Description	: aa		
Created	: 17/07/2023 02:47:53 ص	By	: Administrator
Modified	: 22/05/2025 11:09:38 ص	By	: Administrator

Column	:	Detection	:
Mobile Phase	:	Temperature	:
Flow Rate	:	Pressure	:
Note	:		

Autostop	: None	External Start	: Start Only, Down
Subtraction Chromatogram	: (None)	Matching	: Scale Subtraction Chromatogram

Base	: Not Used	Calibration File	: None	Calculation	: Uncal
Scale Factor	: Not Used	Units After Scaling	: Not Used	Uncal. Response	: 0
Unretained Time	: 0.00 min	Column Length	: 50.00 mm	Column Calc.	: From Width at 50% of Height
Result Table Reports	: All Peaks	Hide ISTD Peak	: Enabled		
Method User Variables:					
MethodUserVar1	: 0	MethodUserVar2	: 0	MethodUserVar3	: 0

HPLC chromatogram info of compound 13f.

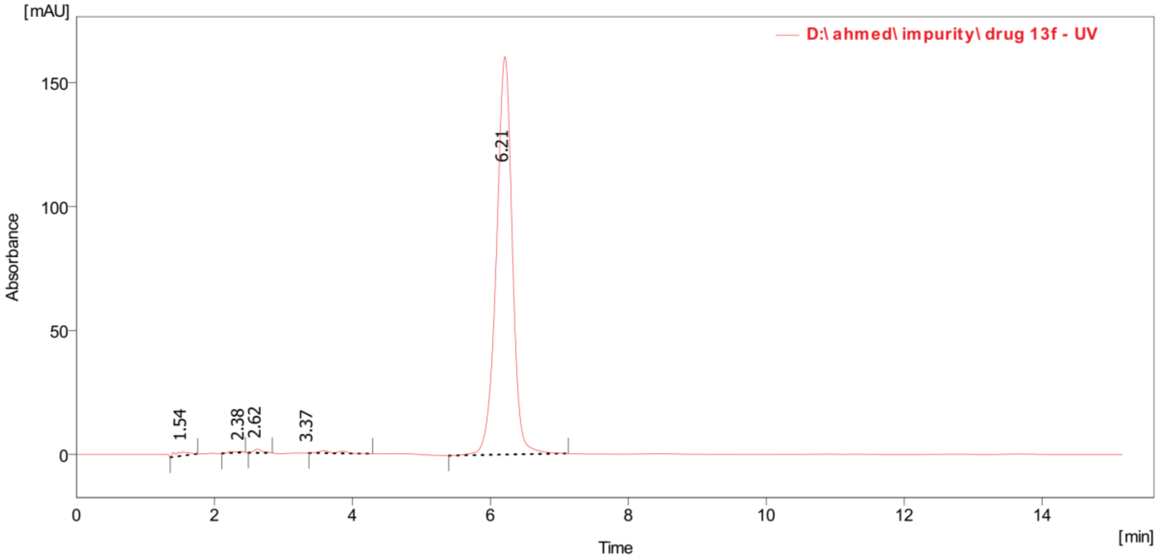

Result Table (Uncal - D:\ahmed\impurity\drug 13f - UV)

	Reten. Time [min]	Area [mAU.s]	Area [%]	Compound Name
1	1.542	22.776	0.9	
2	2.378	6.805	0.3	
3	2.622	12.693	0.5	
4	3.373	19.499	0.7	
5	6.210	2570.964	97.7	
	Total	2632.737	100.0	

HPLC chromatogram of compound 13f.

Sohag University
CENTRAL LAB
DR MOHAMED GAMAL

Chromatogram Info:

File Name	: D:\ahmed\impurity\drug 13g.prm	File Created	: 22/05/2025 01:42:28 م
Origin	: Acquired, Acquisition started 22/05/2025 01:27:24 م	Acquired Date	: 22/05/2025 01:42:27 م
Original Project	: HPLC	By	: Administrator

Printed Version Info:

Printed Version	: - #2; 22/05/2025 01:46:16 م, IA: 8.0 Rev.3	Printed Date	: 22/05/2025 01:46:28 م
Report Style	: D:\HPLC\Common\Chromatogram.sty	By	: Administrator
Calibration File	: None		
Project	: HPLC		

Sample Description:

Sample ID	: drug 13g
Sample	:

Sample Parameters:

Amount	: 0.0	Dilution	: 1.0
Inj. Volume [µL]	: 20.0		
ISTD1 Amount	: 0.0	ISTD2 Amount	: 0.0
ISTD3 Amount	: 0.0	ISTD4 Amount	: 0.0
ISTD5 Amount	: 0.0	ISTD6 Amount	: 0.0
ISTD7 Amount	: 0.0	ISTD8 Amount	: 0.0
ISTD9 Amount	: 0.0	ISTD10 Amount	: 0.0

Analysis User Variables:		Method User Variables:	
AnalysisUserVar1	: 0	MethodUserVar1	: 0
AnalysisUserVar2	: 0	MethodUserVar2	: 0
AnalysisUserVar3	: 0	MethodUserVar3	: 0

Acquisition Method	: D:\ahmed\tolt\tolt separation - #201; 22/05/2025 11:09:38 ص		
Description	: aa		
Created	: 17/07/2023 02:47:53 ص	By	: Administrator
Modified	: 22/05/2025 11:09:38 ص	By	: Administrator

Processing Method	: D:\ahmed\tolt\tolt separation - #201; 22/05/2025 11:09:38 ص + manual changes		
Description	: aa		
Created	: 17/07/2023 02:47:53 ص	By	: Administrator
Modified	: 22/05/2025 11:09:38 ص	By	: Administrator

Column	:	Detection	:
Mobile Phase	:	Temperature	:
Flow Rate	:	Pressure	:
Note	:		

Autostop	: None	External Start	: Start Only, Down
Subtraction Chromatogram	: (None)	Matching	: Scale Subtraction Chromatogram

Base	: Not Used	Calibration File	: None	Calculation	: Uncal
Scale Factor	: Not Used	Units After Scaling	: Not Used	Uncal. Response	: 0
Unretained Time	: 0.00 min	Column Length	: 50.00 mm	Column Calc.	: From Width at 50% of Height
Result Table Reports	: All Peaks	Hide ISTD Peak	: Enabled		
Method User Variables:					
MethodUserVar1	: 0	MethodUserVar2	: 0	MethodUserVar3	: 0

HPLC chromatogram info of compound 13g.

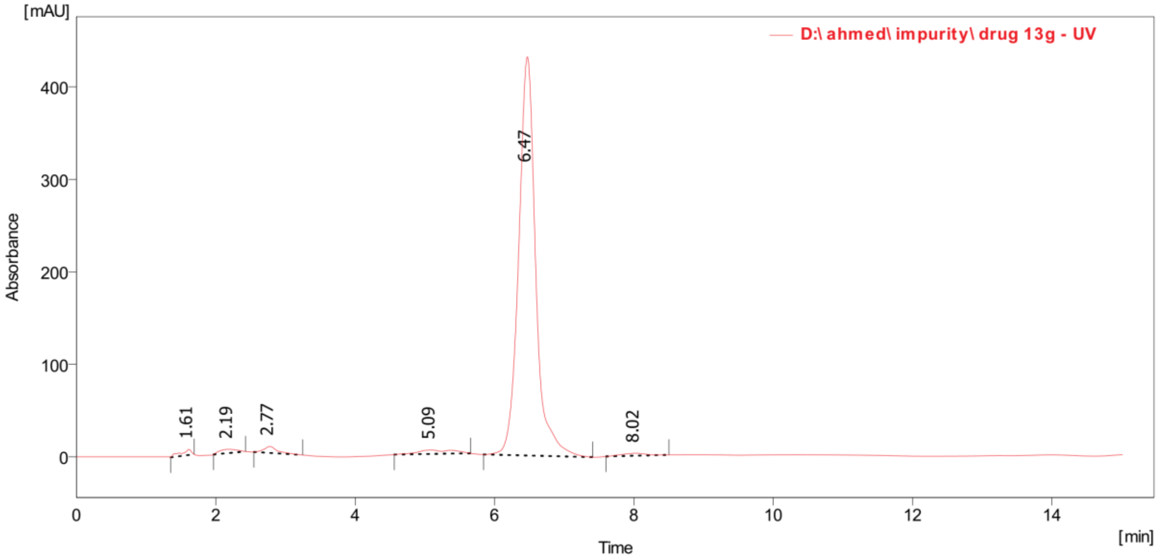

Result Table (Uncal - D: \ahmed\impurity\drug 13g - UV)

	Reten. Time [min]	Area [mAU.s]	Area [%]	Compound Name
1	1.612	64.175	0.8	
2	2.187	71.600	0.9	
3	2.775	97.901	1.3	
4	5.092	148.254	1.9	
5	6.470	7312.318	94.2	
6	8.023	65.159	0.8	
	Total	7759.407	100.0	

HPLC chromatogram of compound 13g.

Sohag University
CENTRAL LAB
DR MOHAMED GAMAL

Chromatogram Info:

File Name	: D:\ahmed\impurity\drug 13h.prm	File Created	: 22/05/2025 01:57:35 م
Origin	: Acquired, Acquisition started 22/05/2025 01:42:31 م	Acquired Date	: 22/05/2025 01:57:35 م
Original Project	: HPLC	By	: Administrator

Printed Version Info:

Printed Version	: - #2; 22/05/2025 02:03:16 م, IA: 8.0 Rev.3	Printed Date	: 22/05/2025 02:03:37 م
Report Style	: D:\HPLC\Common\Chromatogram.sty	By	: Administrator
Calibration File	: None		
Project	: HPLC		

Sample Description:

Sample ID	: drug 13h
Sample	:

Sample Parameters:

Amount	: 0.0	Dilution	: 1.0
Inj. Volume [µL]	: 20.0		
ISTD1 Amount	: 0.0	ISTD2 Amount	: 0.0
ISTD3 Amount	: 0.0	ISTD4 Amount	: 0.0
ISTD5 Amount	: 0.0	ISTD6 Amount	: 0.0
ISTD7 Amount	: 0.0	ISTD8 Amount	: 0.0
ISTD9 Amount	: 0.0	ISTD10 Amount	: 0.0

Analysis User Variables:		Method User Variables:	
AnalysisUserVar1	: 0	MethodUserVar1	: 0
AnalysisUserVar2	: 0	MethodUserVar2	: 0
AnalysisUserVar3	: 0	MethodUserVar3	: 0

Acquisition Method	: D:\ahmed\tolt\tolt separation - #201; 22/05/2025 11:09:38 ص		
Description	: aa		
Created	: 17/07/2023 02:47:53 ص	By	: Administrator
Modified	: 22/05/2025 11:09:38 ص	By	: Administrator

Processing Method	: D:\ahmed\tolt\tolt separation - #201; 22/05/2025 11:09:38 ص + manual changes		
Description	: aa		
Created	: 17/07/2023 02:47:53 ص	By	: Administrator
Modified	: 22/05/2025 11:09:38 ص	By	: Administrator

Column	:	Detection	:
Mobile Phase	:	Temperature	:
Flow Rate	:	Pressure	:
Note	:		

Autostop	: None	External Start	: Start Only, Down
Subtraction Chromatogram	: (None)	Matching	: Scale Subtraction Chromatogram

Base	: Not Used	Calibration File	: None	Calculation	: Uncal
Scale Factor	: Not Used	Units After Scaling	: Not Used	Uncal. Response	: 0
Unretained Time	: 0.00 min	Column Length	: 50.00 mm	Column Calc.	: From Width at 50% of Height
Result Table Reports	: All Peaks	Hide ISTD Peak	: Enabled		
Method User Variables:					
MethodUserVar1	: 0	MethodUserVar2	: 0	MethodUserVar3	: 0

HPLC chromatogram info of compound 13h.

22/05/2025 02:03 م Chromatogram D:\ahmed\impurity\drug 13h.prm Page 2 of 2

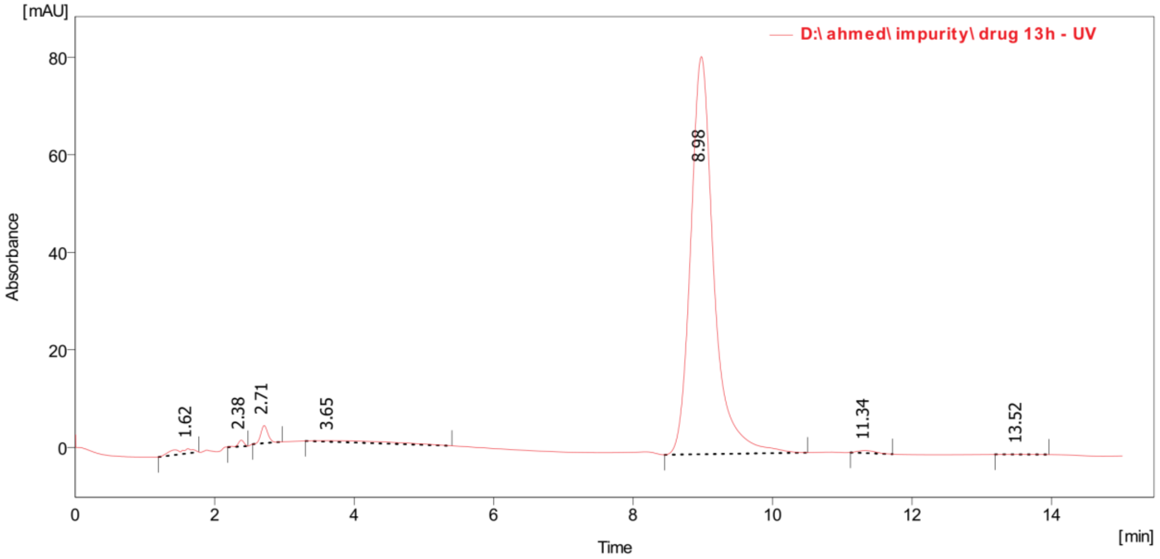

Result Table (Uncal - D: \ahmed\impurity\drug 13h - UV)

	Reten. Time [min]	Area [mAU.s]	Area [%]	Compound Name
1	1.617	21.252	1.1	
2	2.380	7.343	0.4	
3	2.710	25.525	1.3	
4	3.647	28.143	1.4	
5	8.978	1884.976	95.3	
6	11.335	9.100	0.5	
7	13.523	1.093	0.1	
	Total	1977.431	100.0	

HPLC chromatogram of compound 13h.

Sohag University
CENTRAL LAB
DR MOHAMED GAMAL

Chromatogram Info:

File Name	: D:\ahmed\impurity\drug 13i.prm	File Created	: 22/05/2025 02:12:48 م
Origin	: Acquired, Acquisition started 22/05/2025 01:57:39 م	Acquired Date	: 22/05/2025 02:12:48 م
Original Project	: HPLC	By	: Administrator

Printed Version Info:

Printed Version	: - #2; 22/05/2025 02:15:53 م, IA: 8.0 Rev.3	Printed Date	: 22/05/2025 02:16:07 م
Report Style	: D:\HPLC\Common\Chromatogram.sty	By	: Administrator
Calibration File	: None		
Project	: HPLC		

Sample Description:

Sample ID	: drug 13i
Sample	:

Sample Parameters:

Amount	: 0.0	Dilution	: 1.0
Inj. Volume [μL]	: 20.0		
ISTD1 Amount	: 0.0	ISTD2 Amount	: 0.0
ISTD3 Amount	: 0.0	ISTD4 Amount	: 0.0
ISTD5 Amount	: 0.0	ISTD6 Amount	: 0.0
ISTD7 Amount	: 0.0	ISTD8 Amount	: 0.0
ISTD9 Amount	: 0.0	ISTD10 Amount	: 0.0

Analysis User Variables:		Method User Variables:	
AnalysisUserVar1	: 0	MethodUserVar1	: 0
AnalysisUserVar2	: 0	MethodUserVar2	: 0
AnalysisUserVar3	: 0	MethodUserVar3	: 0

Acquisition Method	: D:\ahmed\tolt\tolt separation - #201; 22/05/2025 11:09:38 ص		
Description	: aa		
Created	: 17/07/2023 02:47:53 ص	By	: Administrator
Modified	: 22/05/2025 11:09:38 ص	By	: Administrator

Processing Method	: D:\ahmed\tolt\tolt separation - #201; 22/05/2025 11:09:38 ص + manual changes		
Description	: aa		
Created	: 17/07/2023 02:47:53 ص	By	: Administrator
Modified	: 22/05/2025 11:09:38 ص	By	: Administrator

Column	:	Detection	:
Mobile Phase	:	Temperature	:
Flow Rate	:	Pressure	:
Note	:		

Autostop	: None	External Start	: Start Only, Down
Subtraction Chromatogram	: (None)	Matching	: Scale Subtraction Chromatogram

Base	: Not Used	Calibration File	: None	Calculation	: Uncal
Scale Factor	: Not Used	Units After Scaling	: Not Used	Uncal. Response	: 0
Unretained Time	: 0.00 min	Column Length	: 50.00 mm	Column Calc.	: From Width at 50% of Height
Result Table Reports	: All Peaks	Hide ISTD Peak	: Enabled		
Method User Variables:					
MethodUserVar1	: 0	MethodUserVar2	: 0	MethodUserVar3	: 0

HPLC chromatogram info of compound 13i.

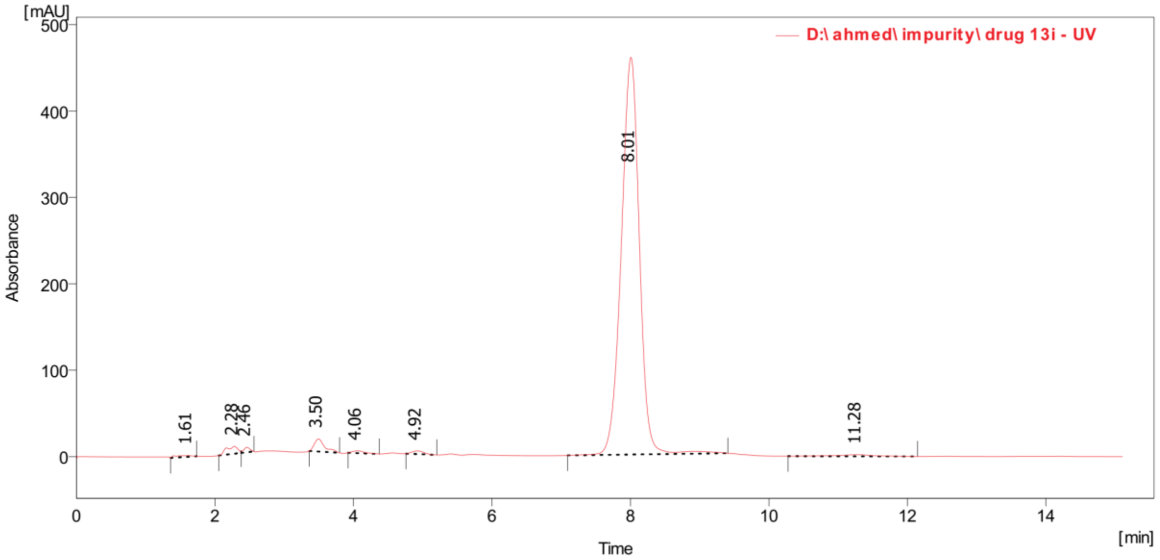

Result Table (Uncal - D:\ahmed\impurity\drug 13i - UV)

	Reten. Time [min]	Area [mAU.s]	Area [%]	Compound Name
1	1.610	28.470	0.3	
2	2.280	101.253	1.1	
3	2.462	35.889	0.4	
4	3.495	152.493	1.7	
5	4.058	32.397	0.4	
6	4.925	44.135	0.5	
7	8.007	8367.562	94.7	
8	11.275	75.035	0.8	
	Total	8837.234	100.0	

HPLC chromatogram of compound 13i.

Sohag University
CENTRAL LAB
DR MOHAMED GAMAL

Chromatogram Info:

File Name	: D:\ahmed\impurity\drug 14a.prm	File Created	: 22/05/2025 02:27:53 م
Origin	: Acquired, Acquisition started 22/05/2025 02:12:51 م	Acquired Date	: 22/05/2025 02:27:53 م
Original Project	: HPLC	By	: Administrator

Printed Version Info:

Printed Version	: - #2; 22/05/2025 02:31:02 م, IA: 8.0 Rev.3	Printed Date	: 22/05/2025 02:31:18 م
Report Style	: D:\HPLC\Common\Chromatogram.sty	By	: Administrator
Calibration File	: None		
Project	: HPLC		

Sample Description:

Sample ID	: drug 14a
Sample	:

Sample Parameters:

Amount	: 0.0	Dilution	: 1.0
Inj. Volume [µL]	: 20.0		
ISTD1 Amount	: 0.0	ISTD2 Amount	: 0.0
ISTD3 Amount	: 0.0	ISTD4 Amount	: 0.0
ISTD5 Amount	: 0.0	ISTD6 Amount	: 0.0
ISTD7 Amount	: 0.0	ISTD8 Amount	: 0.0
ISTD9 Amount	: 0.0	ISTD10 Amount	: 0.0

Analysis User Variables:		Method User Variables:	
AnalysisUserVar1	: 0	MethodUserVar1	: 0
AnalysisUserVar2	: 0	MethodUserVar2	: 0
AnalysisUserVar3	: 0	MethodUserVar3	: 0

Acquisition Method	: D:\ahmed\tolt\tolt separation - #201; 22/05/2025 11:09:38 ص		
Description	: aa		
Created	: 17/07/2023 02:47:53 ص	By	: Administrator
Modified	: 22/05/2025 11:09:38 ص	By	: Administrator

Processing Method	: D:\ahmed\tolt\tolt separation - #201; 22/05/2025 11:09:38 ص + manual changes		
Description	: aa		
Created	: 17/07/2023 02:47:53 ص	By	: Administrator
Modified	: 22/05/2025 11:09:38 ص	By	: Administrator

Column	:	Detection	:
Mobile Phase	:	Temperature	:
Flow Rate	:	Pressure	:
Note	:		

Autostop	: None	External Start	: Start Only, Down
Subtraction Chromatogram	: (None)	Matching	: Scale Subtraction Chromatogram

Base	: Not Used	Calibration File	: None	Calculation	: Uncal
Scale Factor	: Not Used	Units After Scaling	: Not Used	Uncal. Response	: 0
Unretained Time	: 0.00 min	Column Length	: 50.00 mm	Column Calc.	: From Width at 50% of Height
Result Table Reports	: All Peaks	Hide ISTD Peak	: Enabled		
Method User Variables:					
MethodUserVar1	: 0	MethodUserVar2	: 0	MethodUserVar3	: 0

HPLC chromatogram info of compound 14a.

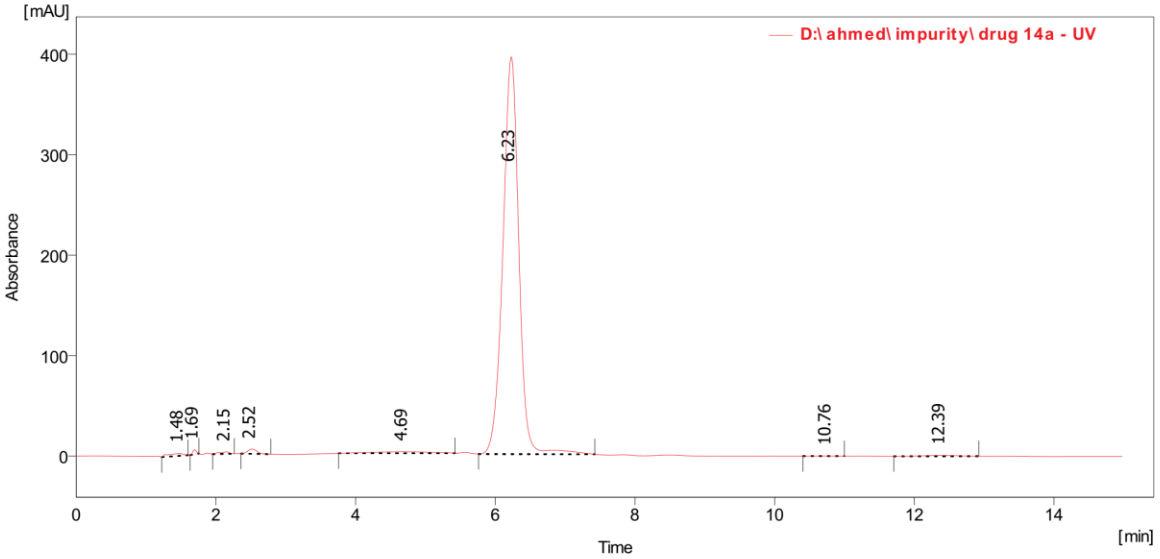

Result Table (Uncal - D: \ahmed\impurity\drug 14a - UV)

	Reten. Time [min]	Area [mAU.s]	Area [%]	Compound Name
1	1.478	38.178	0.6	
2	1.693	17.093	0.3	
3	2.147	20.455	0.3	
4	2.515	42.092	0.6	
5	4.690	93.448	1.4	
6	6.228	6279.056	96.2	
7	10.757	2.572	0.0	
8	12.385	32.770	0.5	
	Total	6525.666	100.0	

HPLC chromatogram info of compound 14a.

Sohag University
CENTRAL LAB
DR MOHAMED GAMAL

Chromatogram Info:

File Name	: D:\ahmed\impurity\drug 14b.prm	File Created	: 25/05/2025 11:11:58 ص
Origin	: Acquired, Acquisition started 25/05/2025 10:56:31 ص	Acquired Date	: 25/05/2025 11:11:58 ص
Original Project	: HPLC	By	: Administrator

Printed Version Info:

Printed Version	: - #2; 25/05/2025 11:14:19 ص, IA: 8.0 Rev.3	Printed Date	: 25/05/2025 11:14:24 ص
Report Style	: D:\HPLC\Common\Chromatogram.sty	By	: Administrator
Calibration File	: None		
Project	: HPLC		

Sample Description:

Sample ID	: drug 14b
Sample	:

Sample Parameters:

Amount	: 0.0	Dilution	: 1.0
Inj. Volume [µL]	: 20.0		
ISTD1 Amount	: 0.0	ISTD2 Amount	: 0.0
ISTD3 Amount	: 0.0	ISTD4 Amount	: 0.0
ISTD5 Amount	: 0.0	ISTD6 Amount	: 0.0
ISTD7 Amount	: 0.0	ISTD8 Amount	: 0.0
ISTD9 Amount	: 0.0	ISTD10 Amount	: 0.0

Analysis User Variables:		Method User Variables:	
AnalysisUserVar1	: 0	MethodUserVar1	: 0
AnalysisUserVar2	: 0	MethodUserVar2	: 0
AnalysisUserVar3	: 0	MethodUserVar3	: 0

Acquisition Method	: D:\ahmed\tolt\tolt separation - #202; 25/05/2025 10:45:28 ص		
Description	: aa		
Created	: 17/07/2023 02:47:53 ص	By	: Administrator
Modified	: 25/05/2025 10:45:28 ص	By	: Administrator

Processing Method	: D:\ahmed\tolt\tolt separation - #202; 25/05/2025 10:45:28 ص + manual changes		
Description	: aa		
Created	: 17/07/2023 02:47:53 ص	By	: Administrator
Modified	: 25/05/2025 10:45:28 ص	By	: Administrator

Column	:	Detection	:
Mobile Phase	:	Temperature	:
Flow Rate	:	Pressure	:
Note	:		

Autostop	: None	External Start	: Start Only, Down
Subtraction Chromatogram	: (None)	Matching	: Scale Subtraction Chromatogram

Base	: Not Used	Calibration File	: None	Calculation	: Uncal
Scale Factor	: Not Used	Units After Scaling	: Not Used	Uncal. Response	: 0
Unretained Time	: 0.00 min	Column Length	: 50.00 mm	Column Calc.	: From Width at 50% of Height
Result Table Reports	: All Peaks	Hide ISTD Peak	: Enabled		
Method User Variables:					
MethodUserVar1	: 0	MethodUserVar2	: 0	MethodUserVar3	: 0

HPLC chromatogram info of compound 14b.

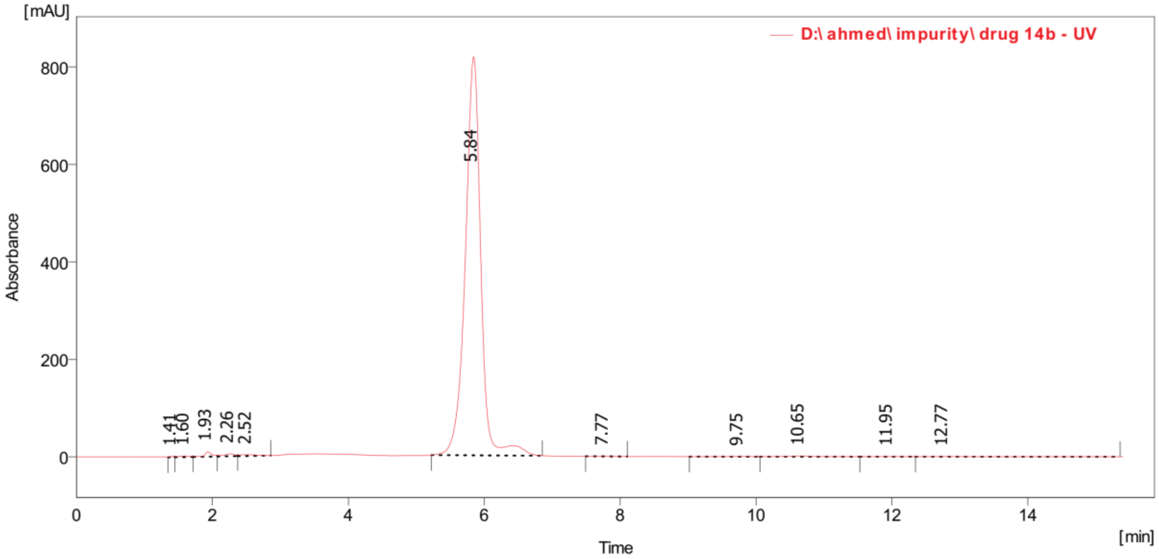

Result Table (Uncal - D: \ahmed\impurity\drug 14b - UV)

	Reten. Time [min]	Area [mAU.s]	Area [%]	Compound Name
1	1.412	8.084	0.1	
2	1.600	27.204	0.2	
3	1.930	70.886	0.5	
4	2.257	58.284	0.4	
5	2.518	47.583	0.4	
6	5.843	12989.241	97.4	
7	7.772	10.771	0.1	
8	9.752	20.916	0.2	
9	10.652	47.798	0.4	
10	11.950	13.057	0.1	
11	12.767	36.000	0.3	
	Total	13329.826	100.0	

HPLC chromatogram of compound 14b.

Sohag University
CENTRAL LAB
DR MOHAMED GAMAL

Chromatogram Info:

File Name	: D:\ahmed\impurity\drug 14c.prm	File Created	: 25/05/2025 11:27:53 ص
Origin	: Acquired, Acquisition started 25/05/2025 11:12:45 ص	Acquired Date	: 25/05/2025 11:27:53 ص
Original Project	: HPLC	By	: Administrator

Printed Version Info:

Printed Version	: - #2; 25/05/2025 11:30:45 ص, IA: 8.0 Rev.3	Printed Date	: 25/05/2025 11:31:01 ص
Report Style	: D:\HPLC\Common\Chromatogram.sty	By	: Administrator
Calibration File	: None		
Project	: HPLC		

Sample Description:

Sample ID	: drug 14c
Sample	:

Sample Parameters:

Amount	: 0.0	Dilution	: 1.0
Inj. Volume [µL]	: 20.0		
ISTD1 Amount	: 0.0	ISTD2 Amount	: 0.0
ISTD3 Amount	: 0.0	ISTD4 Amount	: 0.0
ISTD5 Amount	: 0.0	ISTD6 Amount	: 0.0
ISTD7 Amount	: 0.0	ISTD8 Amount	: 0.0
ISTD9 Amount	: 0.0	ISTD10 Amount	: 0.0

Analysis User Variables:		Method User Variables:	
AnalysisUserVar1	: 0	MethodUserVar1	: 0
AnalysisUserVar2	: 0	MethodUserVar2	: 0
AnalysisUserVar3	: 0	MethodUserVar3	: 0

Acquisition Method	: D:\ahmed\tolt\tolt separation - #202; 25/05/2025 10:45:28 ص		
Description	: aa		
Created	: 17/07/2023 02:47:53 ص	By	: Administrator
Modified	: 25/05/2025 10:45:28 ص	By	: Administrator

Processing Method	: D:\ahmed\tolt\tolt separation - #202; 25/05/2025 10:45:28 ص + manual changes		
Description	: aa		
Created	: 17/07/2023 02:47:53 ص	By	: Administrator
Modified	: 25/05/2025 10:45:28 ص	By	: Administrator

Column	:	Detection	:
Mobile Phase	:	Temperature	:
Flow Rate	:	Pressure	:
Note	:		

Autostop	: None	External Start	: Start Only, Down
Subtraction Chromatogram	: (None)	Matching	: Scale Subtraction Chromatogram

Base	: Not Used	Calibration File	: None	Calculation	: Uncal
Scale Factor	: Not Used	Units After Scaling	: Not Used	Uncal. Response	: 0
Unretained Time	: 0.00 min	Column Length	: 50.00 mm	Column Calc.	: From Width at 50% of Height
Result Table Reports	: All Peaks	Hide ISTD Peak	: Enabled		
Method User Variables:					
MethodUserVar1	: 0	MethodUserVar2	: 0	MethodUserVar3	: 0

HPLC chromatogram info of compound 14c.

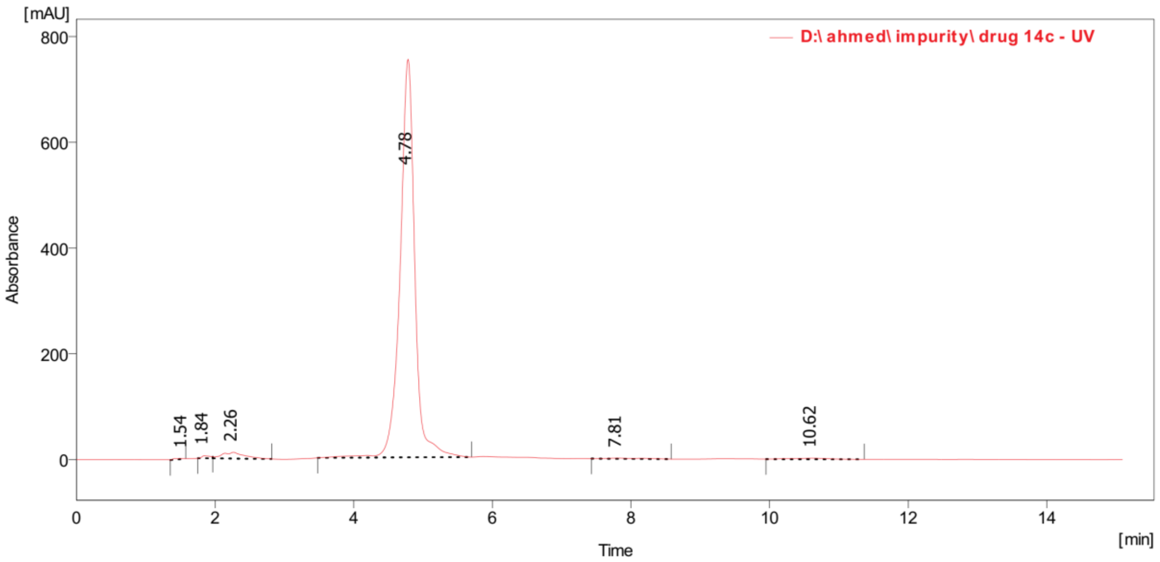

Result Table (Uncal - D: \ahmed\impurity\drug 14c - UV)

	Reten. Time [min]	Area [mAU.s]	Area [%]	Compound Name
1	1.538	12.784	0.1	
2	1.843	40.037	0.3	
3	2.263	265.722	2.3	
4	4.783	11106.056	96.2	
5	7.810	53.841	0.5	
6	10.618	68.102	0.6	
	Total	11546.542	100.0	

HPLC chromatogram of compound 14c.

Sohag University
CENTRAL LAB
DR MOHAMED GAMAL

Chromatogram Info:

File Name	: D:\ahmed\impurity\drug 14d.prm	File Created	: 25/05/2025 11:42:59 ص
Origin	: Acquired, Acquisition started 25/05/2025 11:27:56 ص	Acquired Date	: 25/05/2025 11:42:59 ص
Original Project	: HPLC	By	: Administrator

Printed Version Info:

Printed Version	: - #2; 25/05/2025 11:45:14 ص, IA: 8.0 Rev.3	Printed Date	: 25/05/2025 11:45:27 ص
Report Style	: D:\HPLC\Common\Chromatogram.sty	By	: Administrator
Calibration File	: None		
Project	: HPLC		

Sample Description:

Sample ID	: drug 14d
Sample	:

Sample Parameters:

Amount	: 0.0	Dilution	: 1.0
Inj. Volume [µL]	: 20.0		
ISTD1 Amount	: 0.0	ISTD2 Amount	: 0.0
ISTD3 Amount	: 0.0	ISTD4 Amount	: 0.0
ISTD5 Amount	: 0.0	ISTD6 Amount	: 0.0
ISTD7 Amount	: 0.0	ISTD8 Amount	: 0.0
ISTD9 Amount	: 0.0	ISTD10 Amount	: 0.0

Analysis User Variables:		Method User Variables:	
AnalysisUserVar1	: 0	MethodUserVar1	: 0
AnalysisUserVar2	: 0	MethodUserVar2	: 0
AnalysisUserVar3	: 0	MethodUserVar3	: 0

Acquisition Method	: D:\ahmed\tolt\tolt separation - #202; 25/05/2025 10:45:28 ص		
Description	: aa		
Created	: 17/07/2023 02:47:53 ص	By	: Administrator
Modified	: 25/05/2025 10:45:28 ص	By	: Administrator

Processing Method	: D:\ahmed\tolt\tolt separation - #202; 25/05/2025 10:45:28 ص + manual changes		
Description	: aa		
Created	: 17/07/2023 02:47:53 ص	By	: Administrator
Modified	: 25/05/2025 10:45:28 ص	By	: Administrator

Column	:	Detection	:
Mobile Phase	:	Temperature	:
Flow Rate	:	Pressure	:
Note	:		

Autostop	: None	External Start	: Start Only, Down
Subtraction Chromatogram	: (None)	Matching	: Scale Subtraction Chromatogram

Base	: Not Used	Calibration File	: None	Calculation	: Uncal
Scale Factor	: Not Used	Units After Scaling	: Not Used	Uncal. Response	: 0
Unretained Time	: 0.00 min	Column Length	: 50.00 mm	Column Calc.	: From Width at 50% of Height
Result Table Reports	: All Peaks	Hide ISTD Peak	: Enabled		
Method User Variables:					
MethodUserVar1	: 0	MethodUserVar2	: 0	MethodUserVar3	: 0

HPLC chromatogram info of compound 14d.

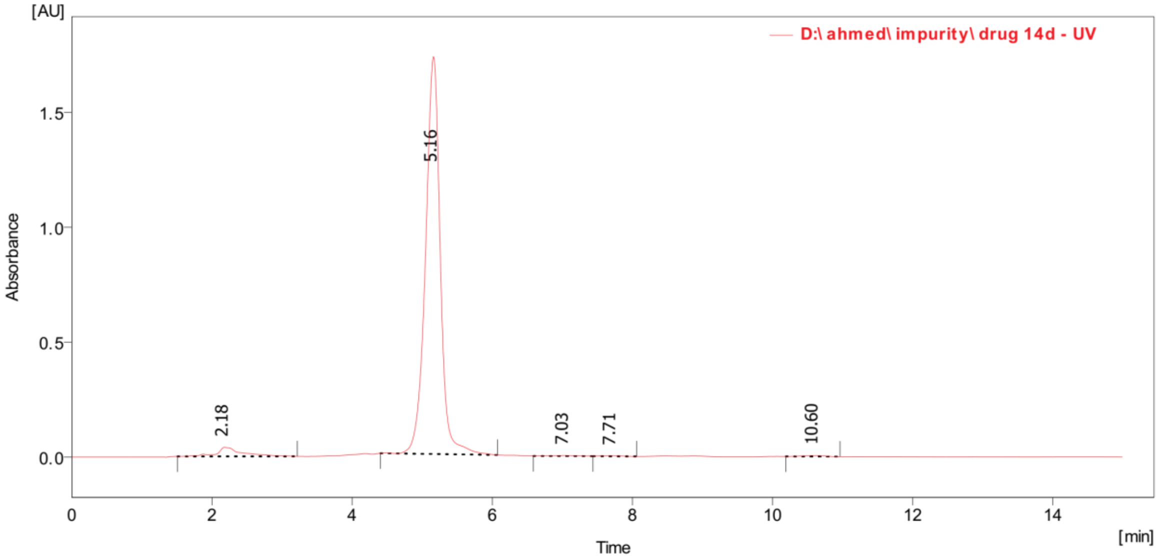

Result Table (Uncal - D:\ahmed\impurity\drug 14d - UV)

	Reten. Time [min]	Area [mAU.s]	Area [%]	Compound Name
1	2.178	1002.250	3.8	
2	5.160	25311.773	95.4	
3	7.032	65.716	0.2	
4	7.708	44.418	0.2	
5	10.597	95.654	0.4	
	Total	26519.810	100.0	

HPLC chromatogram of compound 14d.

Sohag University
CENTRAL LAB
DR MOHAMED GAMAL

Chromatogram Info:

File Name	: D:\ahmed\impurity\drug 14e.prm	File Created	: 22/05/2025 01:47:18 م
Origin	: Acquired, Acquisition started 22/05/2025 01:12:05 م	Acquired Date	: 22/05/2025 01:47:18 م
Original Project	: HPLC	By	: Administrator

Printed Version Info:

Printed Version	: - #3; 22/05/2025 01:30:41 م, IA: 8.0 Rev.3	Printed Date	: 22/05/2025 01:58:05 م
Report Style	: D:\HPLC\Common\Chromatogram.sty	By	: Administrator
Calibration File	: None		
Project	: HPLC		

Sample Description:

Sample ID	: drug 14e
Sample	:

Sample Parameters:

Amount	: 0.0	Dilution	: 1.0
Inj. Volume [µL]	: 20.0		
ISTD1 Amount	: 0.0	ISTD2 Amount	: 0.0
ISTD3 Amount	: 0.0	ISTD4 Amount	: 0.0
ISTD5 Amount	: 0.0	ISTD6 Amount	: 0.0
ISTD7 Amount	: 0.0	ISTD8 Amount	: 0.0
ISTD9 Amount	: 0.0	ISTD10 Amount	: 0.0

Analysis User Variables:		Method User Variables:	
AnalysisUserVar1	: 0	MethodUserVar1	: 0
AnalysisUserVar2	: 0	MethodUserVar2	: 0
AnalysisUserVar3	: 0	MethodUserVar3	: 0

Acquisition Method	: D:\ahmed\tolt\tolt separation - #201; 22/05/2025 11:09:38 ص		
Description	: aa		
Created	: 17/07/2023 02:47:53 ص	By	: Administrator
Modified	: 22/05/2025 11:09:38 ص	By	: Administrator

Processing Method	: D:\ahmed\tolt\tolt separation - #201; 22/05/2025 11:09:38 ص + manual changes		
Description	: aa		
Created	: 17/07/2023 02:47:53 ص	By	: Administrator
Modified	: 22/05/2025 11:09:38 ص	By	: Administrator

Column	:	Detection	:
Mobile Phase	:	Temperature	:
Flow Rate	:	Pressure	:
Note	:		

Autostop	: None	External Start	: Start Only, Down
Subtraction Chromatogram	: (None)	Matching	: Scale Subtraction Chromatogram

Base	: Not Used	Calibration File	: None	Calculation	: Uncal
Scale Factor	: Not Used	Units After Scaling	: Not Used	Uncal. Response	: 0
Unretained Time	: 0.00 min	Column Length	: 50.00 mm	Column Calc.	: From Width at 50% of Height
Result Table Reports	: All Peaks	Hide ISTD Peak	: Enabled		
Method User Variables:					
MethodUserVar1	: 0	MethodUserVar2	: 0	MethodUserVar3	: 0

HPLC chromatogram info of compound 14e.

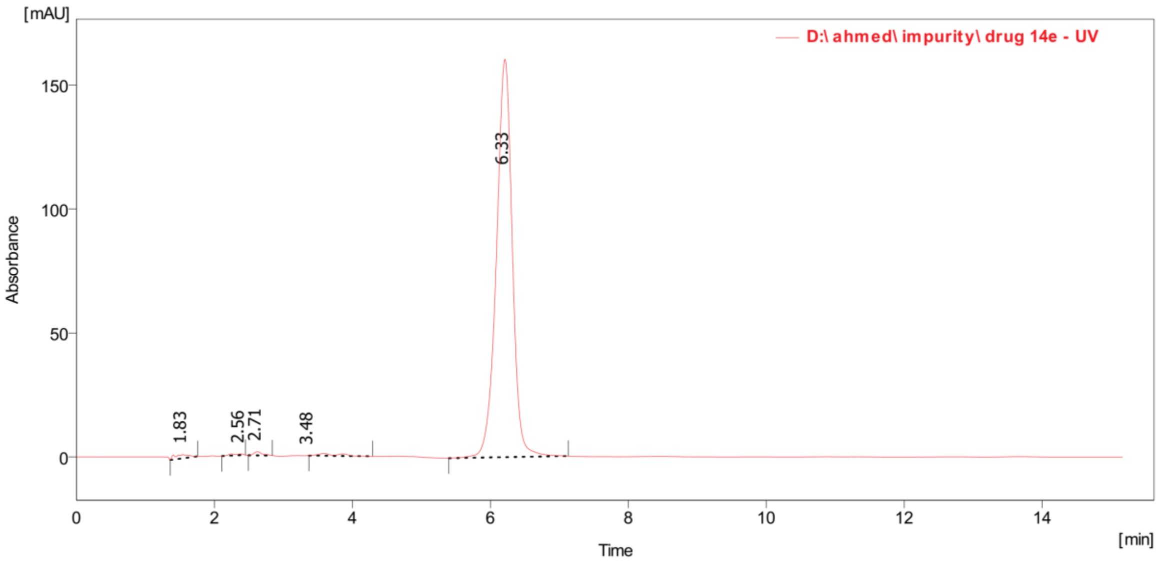

Result Table (Uncal - D:\ahmed\impurity\drug 14e - UV)

	Reten. Time [min]	Area [mAU.s]	Area [%]	Compound Name
1	1.831	22.776	1.2	
2	2.568	6.805	0.5	
3	2.712	12.693	0.4	
4	3.483	19.499	0.8	
5	6.332	2570.964	97.1	
	Total	2632.737	100.0	

HPLC chromatogram of compound 14e.

Sohag University
CENTRAL LAB
DR MOHAMED GAMAL

Chromatogram Info:

File Name	: D:\AHMED\IMPURITY\drug 14f.prm	File Created	: 25/05/2025 11:58:04 ص
Origin	: Acquired, Acquisition started 25/05/2025 11:43:02 ص	Acquired Date	: 25/05/2025 11:58:04 ص
Original Project	: HPLC	By	: Administrator

Printed Version Info:

Printed Version	: - #2; 25/05/2025 12:00:43 م, IA: 8.0 Rev.3	Printed Date	: 25/05/2025 12:02:44 م
Report Style	: D:\HPLC\Common\Chromatogram.sty	By	: Administrator
Calibration File	: None		
Project	: HPLC		

Sample Description:

Sample ID	: drug 14f
Sample	:

Sample Parameters:

Amount	: 0.0	Dilution	: 1.0
Inj. Volume [µL]	: 20.0		
ISTD1 Amount	: 0.0	ISTD2 Amount	: 0.0
ISTD3 Amount	: 0.0	ISTD4 Amount	: 0.0
ISTD5 Amount	: 0.0	ISTD6 Amount	: 0.0
ISTD7 Amount	: 0.0	ISTD8 Amount	: 0.0
ISTD9 Amount	: 0.0	ISTD10 Amount	: 0.0

Analysis User Variables:		Method User Variables:	
AnalysisUserVar1	: 0	MethodUserVar1	: 0
AnalysisUserVar2	: 0	MethodUserVar2	: 0
AnalysisUserVar3	: 0	MethodUserVar3	: 0

Acquisition Method	: D:\ahmed\tolt\tolt separation - #202; 25/05/2025 10:45:28 ص		
Description	: aa		
Created	: 17/07/2023 02:47:53 ص	By	: Administrator
Modified	: 25/05/2025 10:45:28 ص	By	: Administrator

Processing Method	: D:\ahmed\tolt\tolt separation - #202; 25/05/2025 10:45:28 ص + manual changes		
Description	: aa		
Created	: 17/07/2023 02:47:53 ص	By	: Administrator
Modified	: 25/05/2025 10:45:28 ص	By	: Administrator

Column	:	Detection	:
Mobile Phase	:	Temperature	:
Flow Rate	:	Pressure	:
Note	:		

Autostop	: None	External Start	: Start Only, Down
Subtraction Chromatogram	: (None)	Matching	: Scale Subtraction Chromatogram

Base	: Not Used	Calibration File	: None	Calculation	: Uncal
Scale Factor	: Not Used	Units After Scaling	: Not Used	Uncal. Response	: 0
Unretained Time	: 0.00 min	Column Length	: 50.00 mm	Column Calc.	: From Width at 50% of Height
Result Table Reports	: All Peaks	Hide ISTD Peak	: Enabled		
Method User Variables:					
MethodUserVar1	: 0	MethodUserVar2	: 0	MethodUserVar3	: 0

HPLC chromatogram info of compound 14f.

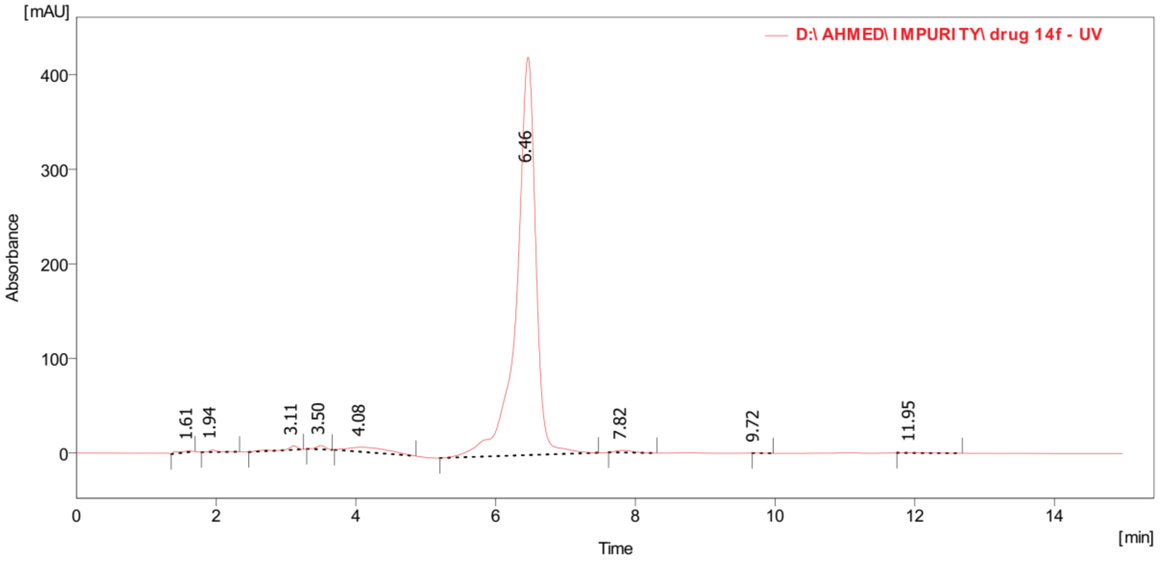

Result Table (Uncal - D:\AHMED\IMPURITY\drug 14f - UV)

	Reten. Time [min]	Area [mAU.s]	Area [%]	Compound Name
1	1.610	25.139	0.3	
2	1.943	19.945	0.2	
3	3.110	50.718	0.6	
4	3.500	36.473	0.4	
5	4.078	212.424	2.5	
6	6.463	8078.759	95.3	
7	7.822	40.362	0.5	
8	9.722	0.847	0.0	
9	11.952	9.466	0.1	
	Total	8474.133	100.0	

HPLC chromatogram of compound 14f.

Sohag University
CENTRAL LAB
DR MOHAMED GAMAL

Chromatogram Info:

File Name	: D:\ahmed\impurity\drug 14g.prm	File Created	: 25/05/2025 12:21:35 م
Origin	: Acquired, Acquisition started 25/05/2025 12:06:30 م	Acquired Date	: 25/05/2025 12:21:35 م
Original Project	: HPLC	By	: Administrator

Printed Version Info:

Printed Version	: - #2; 25/05/2025 12:23:36 م, IA: 8.0 Rev.3	Printed Date	: 25/05/2025 12:23:54 م
Report Style	: D:\HPLC\Common\Chromatogram.sty	By	: Administrator
Calibration File	: None		
Project	: HPLC		

Sample Description:

Sample ID	: drug 14g
Sample	:

Sample Parameters:

Amount	: 0.0	Dilution	: 1.0
Inj. Volume [µL]	: 20.0		
ISTD1 Amount	: 0.0	ISTD2 Amount	: 0.0
ISTD3 Amount	: 0.0	ISTD4 Amount	: 0.0
ISTD5 Amount	: 0.0	ISTD6 Amount	: 0.0
ISTD7 Amount	: 0.0	ISTD8 Amount	: 0.0
ISTD9 Amount	: 0.0	ISTD10 Amount	: 0.0

Analysis User Variables:		Method User Variables:	
AnalysisUserVar1	: 0	MethodUserVar1	: 0
AnalysisUserVar2	: 0	MethodUserVar2	: 0
AnalysisUserVar3	: 0	MethodUserVar3	: 0

Acquisition Method	: D:\ahmed\tolt\tolt separation - #204; 25/05/2025 12:01:33 م		
Description	: aa		
Created	: 17/07/2023 02:47:53 ص	By	: Administrator
Modified	: 25/05/2025 12:01:33 م	By	: Administrator

Processing Method	: D:\ahmed\tolt\tolt separation - #204; 25/05/2025 12:01:33 م + manual changes		
Description	: aa		
Created	: 17/07/2023 02:47:53 ص	By	: Administrator
Modified	: 25/05/2025 12:01:33 م	By	: Administrator

Column	:	Detection	:
Mobile Phase	:	Temperature	:
Flow Rate	:	Pressure	:
Note	:		

Autostop	: None	External Start	: Start Only, Down
Subtraction Chromatogram	: (None)	Matching	: Scale Subtraction Chromatogram

Base	: Not Used	Calibration File	: None	Calculation	: Uncal
Scale Factor	: Not Used	Units After Scaling	: Not Used	Uncal. Response	: 0
Unretained Time	: 0.00 min	Column Length	: 50.00 mm	Column Calc.	: From Width at 50% of Height
Result Table Reports	: All Peaks	Hide ISTD Peak	: Enabled		
Method User Variables:					
MethodUserVar1	: 0	MethodUserVar2	: 0	MethodUserVar3	: 0

HPLC chromatogram info of compound 14g.

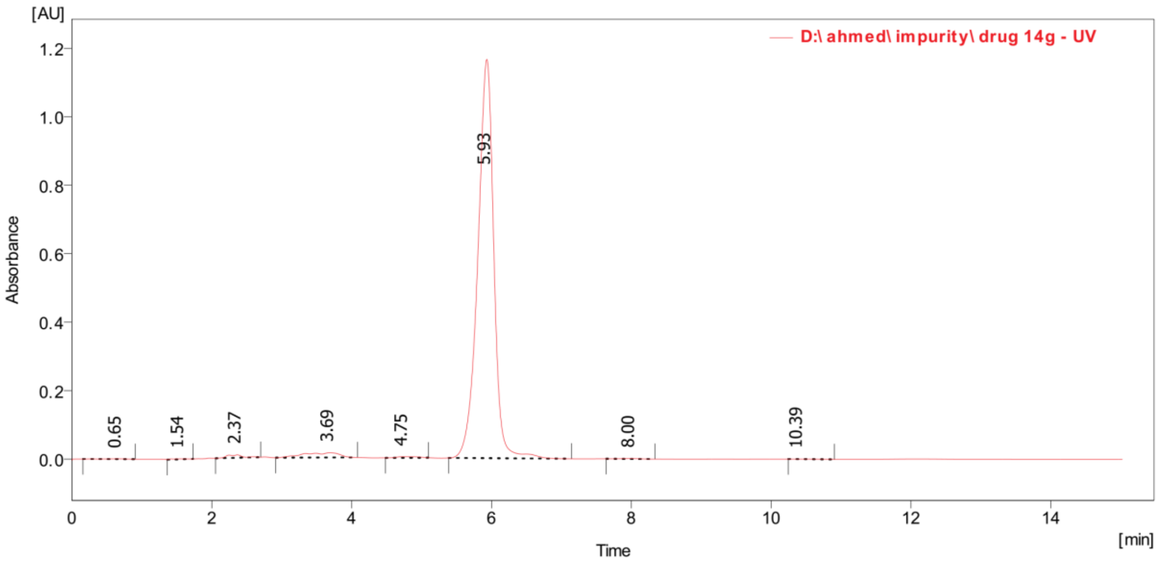

Result Table (Uncal - D:\ahmed\impurity\drug 14g - UV)

	Reten. Time [min]	Area [mAU.s]	Area [%]	Compound Name
1	0.652	22.172	0.1	
2	1.545	22.769	0.1	
3	2.367	128.525	0.7	
4	3.688	510.202	2.6	
5	4.745	67.244	0.3	
6	5.933	18555.176	96.0	
7	7.995	20.591	0.1	
8	10.393	8.138	0.0	
	Total	19334.816	100.0	

HPLC chromatogram of compound 14g.

Sohag University
CENTRAL LAB
DR MOHAMED GAMAL

Chromatogram Info:

File Name	: D:\ahmed\impurity\drug 14h.prm	File Created	: 25/05/2025 12:36:39 م
Origin	: Acquired, Acquisition started 25/05/2025 12:21:36 م	Acquired Date	: 25/05/2025 12:36:39 م
Original Project	: HPLC	By	: Administrator

Printed Version Info:

Printed Version	: - #2; 25/05/2025 12:39:02 م, IA: 8.0 Rev.3	Printed Date	: 25/05/2025 12:39:20 م
Report Style	: D:\HPLC\Common\Chromatogram.sty	By	: Administrator
Calibration File	: None		
Project	: HPLC		

Sample Description:

Sample ID	: drug 1h
Sample	:

Sample Parameters:

Amount	: 0.0	Dilution	: 1.0
Inj. Volume [µL]	: 20.0		
ISTD1 Amount	: 0.0	ISTD2 Amount	: 0.0
ISTD3 Amount	: 0.0	ISTD4 Amount	: 0.0
ISTD5 Amount	: 0.0	ISTD6 Amount	: 0.0
ISTD7 Amount	: 0.0	ISTD8 Amount	: 0.0
ISTD9 Amount	: 0.0	ISTD10 Amount	: 0.0

Analysis User Variables:		Method User Variables:	
AnalysisUserVar1	: 0	MethodUserVar1	: 0
AnalysisUserVar2	: 0	MethodUserVar2	: 0
AnalysisUserVar3	: 0	MethodUserVar3	: 0

Acquisition Method	: D:\ahmed\tolt\tolt separation - #204; 25/05/2025 12:01:33 م		
Description	: aa		
Created	: 17/07/2023 02:47:53 ص	By	: Administrator
Modified	: 25/05/2025 12:01:33 م	By	: Administrator

Processing Method	: D:\ahmed\tolt\tolt separation - #204; 25/05/2025 12:01:33 م + manual changes		
Description	: aa		
Created	: 17/07/2023 02:47:53 ص	By	: Administrator
Modified	: 25/05/2025 12:01:33 م	By	: Administrator

Column	:	Detection	:
Mobile Phase	:	Temperature	:
Flow Rate	:	Pressure	:
Note	:		

Autostop	: None	External Start	: Start Only, Down
Subtraction Chromatogram	: (None)	Matching	: Scale Subtraction Chromatogram

Base	: Not Used	Calibration File	: None	Calculation	: Uncal
Scale Factor	: Not Used	Units After Scaling	: Not Used	Uncal. Response	: 0
Unretained Time	: 0.00 min	Column Length	: 50.00 mm	Column Calc.	: From Width at 50% of Height
Result Table Reports	: All Peaks	Hide ISTD Peak	: Enabled		
Method User Variables:					
MethodUserVar1	: 0	MethodUserVar2	: 0	MethodUserVar3	: 0

HPLC chromatogram info of compound 14h.

25/05/2025 12:39 م Chromatogram D:\ahmed\impurity\drug 14h.prm Page 2 of 2

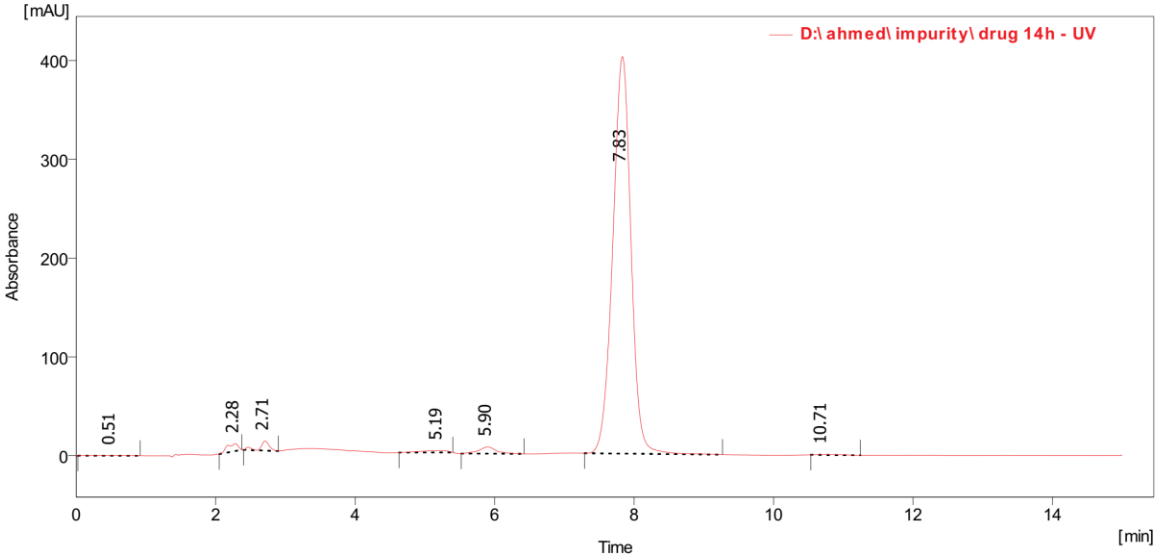

Result Table (Uncal - D:\ahmed\impurity\drug 14h - UV)

	Reten. Time [min]	Area [mAU.s]	Area [%]	Compound Name
1	0.505	6.906	0.1	
2	2.278	85.368	1.1	
3	2.707	86.384	1.1	
4	5.192	51.126	0.7	
5	5.900	122.406	1.6	
6	7.832	7285.586	95.3	
7	10.710	10.441	0.1	
	Total	7648.216	100.0	

HPLC chromatogram of compound 14h.

Sohag University
CENTRAL LAB
DR MOHAMED GAMAL

Chromatogram Info:

File Name	: D:\ahmed\impurity\drug 14i.prm	File Created	: 25/05/2025 12:51:48 م
Origin	: Acquired, Acquisition started 25/05/2025 12:36:44 م	Acquired Date	: 25/05/2025 12:51:47 م
Original Project	: HPLC	By	: Administrator

Printed Version Info:

Printed Version	: - #2; 25/05/2025 12:56:23 م, IA: 8.0 Rev.3	Printed Date	: 25/05/2025 12:56:29 م
Report Style	: D:\HPLC\Common\Chromatogram.sty	By	: Administrator
Calibration File	: None		
Project	: HPLC		

Sample Description:

Sample ID	: drug 14i
Sample	:

Sample Parameters:

Amount	: 0.0	Dilution	: 1.0
Inj. Volume [µL]	: 20.0		
ISTD1 Amount	: 0.0	ISTD2 Amount	: 0.0
ISTD3 Amount	: 0.0	ISTD4 Amount	: 0.0
ISTD5 Amount	: 0.0	ISTD6 Amount	: 0.0
ISTD7 Amount	: 0.0	ISTD8 Amount	: 0.0
ISTD9 Amount	: 0.0	ISTD10 Amount	: 0.0

Analysis User Variables:		Method User Variables:	
AnalysisUserVar1	: 0	MethodUserVar1	: 0
AnalysisUserVar2	: 0	MethodUserVar2	: 0
AnalysisUserVar3	: 0	MethodUserVar3	: 0

Acquisition Method	: D:\ahmed\tolt\tolt separation - #204; 25/05/2025 12:01:33 م		
Description	: aa		
Created	: 17/07/2023 02:47:53 ص	By	: Administrator
Modified	: 25/05/2025 12:01:33 م	By	: Administrator

Processing Method	: D:\ahmed\tolt\tolt separation - #204; 25/05/2025 12:01:33 م + manual changes		
Description	: aa		
Created	: 17/07/2023 02:47:53 ص	By	: Administrator
Modified	: 25/05/2025 12:01:33 م	By	: Administrator

Column	:	Detection	:
Mobile Phase	:	Temperature	:
Flow Rate	:	Pressure	:
Note	:		

Autostop	: None	External Start	: Start Only, Down
Subtraction Chromatogram	: (None)	Matching	: Scale Subtraction Chromatogram

Base	: Not Used	Calibration File	: None	Calculation	: Uncal
Scale Factor	: Not Used	Units After Scaling	: Not Used	Uncal. Response	: 0
Unretained Time	: 0.00 min	Column Length	: 50.00 mm	Column Calc.	: From Width at 50% of Height
Result Table Reports	: All Peaks	Hide ISTD Peak	: Enabled		
Method User Variables:					
MethodUserVar1	: 0	MethodUserVar2	: 0	MethodUserVar3	: 0

HPLC chromatogram info of compound 14i.

25/05/2025 12:56 م Chromatogram D:\ahmed\impurity\drug 14i.prm Page 2 of 2

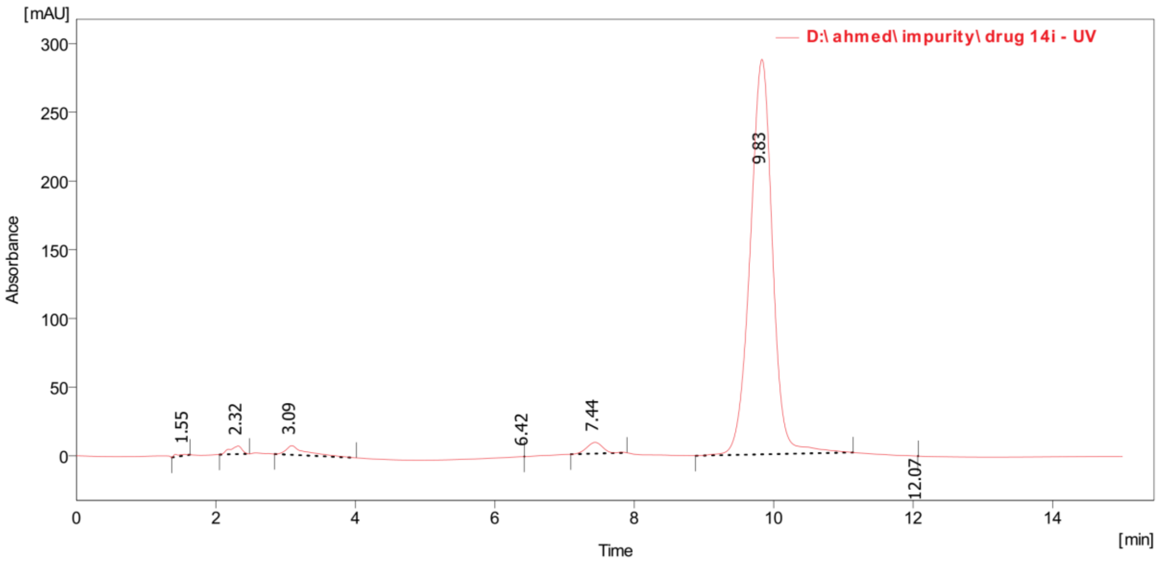

Result Table (Uncal - D:\ahmed\impurity\drug 14i - UV)

	Reten. Time [min]	Area [mAU.s]	Area [%]	Compound Name
1	1.548	13.022	0.2	
2	2.320	72.295	1.1	
3	3.087	135.720	2.0	
4	6.423	0.000	0.0	
5	7.437	132.484	2.0	
6	9.832	6408.398	94.8	
7	12.068	0.001	0.0	
	Total	6761.921	100.0	

HPLC chromatogram of compound 14i.

Elemental analysis results

The compounds codes have been changed into the following codes
codes **6a-b** changed in the manuscript into **9a-b**
codes **9a-i** changed in the manuscript into **12a-i**
codes **9j-r** changed in the manuscript into **14a-i**

جامعة الأزهر
Al-Azhar University
المركز الإقليمي للفطريات وتطبيقاتها
The Regional Center for Mycology and Biotechnology

Requester Data:
Name: **Dr. Omar Mamdouh Kamal Alshazly**
Authority: Faculty of Pharmacy, Sohag University
Sample Data:
Ten samples had been submitted for elemental analysis.
Analysis Report:

Sample Code	C%	H%	N%
6a	63.91	3.89	17.65
6b	61.54	4.15	16.12
9a	69.85	4.41	12.23
9b	68.61	4.68	11.68
9c	67.40	4.62	11.07
9d	65.82	4.68	10.61
9e	68.05	4.19	11.87
9f	61.85	3.73	10.84
9g	65.12	3.94	13.60
9h	70.19	4.67	11.98

INVESTIGATOR
Dr. M. Mourad

DIRECTOR
M. Mansour
17.7.2024

شارع المخيم الدائم - مدينة نصر - القاهرة
تليفون: ٢٢٦٢٠٣٧٣ (٠٢٠٢) فاكس: ٢٢٦٢٠٣٧٣ (٠٢٠٢)
البريد الإلكتروني : rcmb@azhar.edu.eg
الموقع الإلكتروني:
http:// www.azhar.edu.eg.htm
http://www.azhar.edu.eg/pages/fungi_center.htm
Facebook: RCMB AZHAR
صندوق بريد ١١٧٥١ مدينة نصر القاهرة

جامعة الأزهر
Al-Azhar University
المركز الإقليمى للفطريات وتطبيقاتها
The Regional Center for Mycology and Biotechnology

Requester Data:

Name: **Dr. Omar Mamdouh Kamal Alshazly**
Authority: Faculty of Pharmacy, Sohag University

Sample Data:

Ten samples had been submitted for elemental analysis.

Analysis Report:

Sample Code	C%	H%	N%
9i	74.72	4.37	11.26
9j	68.71	4.59	11.63
9k	67.43	4.67	11.04
9L	66.12	4.75	10.61
9m	65.09	4.68	10.13
9n	66.38	4.32	11.26
9o	60.89	3.96	10.37
9p	64.01	4.15	13.03
9q	68.69	4.78	11.35
9r	70.84	4.69	10.67

INVESTIGATOR **DIRECTOR**

Dr. m. mourad. M. Mansour
17. 7. 2024

شارع المخيم الدائم - مدينة نصر - القاهرة
البريد الإلكتروني : rcmb@azhar.edu.eg
الموقع الإلكتروني:
صندوق بريد ١١٧٥١ مدينة نصر القاهرة
تليفون : ٢٢٦٢٠٣٧٣ (٠٢٠٢) فاكس : ٢٢٦٢٠٣٧٣ (٠٢٠٢)
http:// www.azhar.edu.eg.htm
http://www.azhar.edu.eg/pages/fungi_center.htm
Facebook: RCMB AZHAR

Requester Data:

Name: Dr. Omar Mamdouh Kamal Alshazly

Authority: Faculty of Pharmacy, Sohag University

Sample Data:

One sample had been submitted for elemental analysis.

Analysis Report:

Sample Code	C%	H%	N%
12i	72.25	4.37	11.26

Head of the Lab

Dr. m. mourad

Prof. Dr. Mohamed Mourad

11/5/2025

DIRECTOR

M. Mansour

12/5/2025

Prof. Dr. Mohamed Mansour

Docking methodology

AutoDock Vina v.1.2.0 was used for carrying out the molecular docking [1, 2]. The experimental procedure followed for the in silico molecular docking analysis of the synthesized compound was as reported by Blessy and Sharmila [3] with slight modifications. The interactions of the synthesized compounds and reference drugs with the proteins VEGFR-2 (PDB ID: 2OH4) and EGFR (PDB ID: 1M17) were studied using AutoDock Vina v.1.2.0 [55]. The structure of docked compound was built using Chem. 3D ultra 12.0 software [Chemical Structure Drawing Standard; Cambridge Soft corporation, USA (2010)] assigned with proper 2D orientation. The energy of each molecule was minimized using ChemBio3D and were then used as input for AutoDock Vina, in order to carry out the docking simulation. The crystal structures of VEGFR-2 (PDB ID: 2OH4) and EGFR (PDB ID: 1M17) enzymes were downloaded from protein data bank. The protein preparation was done using the reported standard protocol; the target protein file was prepared by leaving the associated residue with protein using Auto preparation of target protein file AutoDock 4.2(MGLTools 1.5.6). The graphical user interface program was used to set the grid box for docking simulations. The grid was set so that it surrounds the region of interest in the macromolecule.43The docking algorithm provided with AutoDock Vina v.1.2.0 was used to search for the best docked conformation between ligand and protein. During the docking process, a maximum of nine conformers were considered for each ligand. The conformations with the most favorable (least) free binding energy were selected for analyzing the interactions between the target receptor and ligands by Discovery Studio Visualizer and PyMOL. The ligands are represented indifferent color; H-bonds and the interacting residues are represented in ball and stick model representation [1].

References

[1] D. Zeleke, R. Eswaramoorthy, Z. Belay, Y. Melaku, Synthesis and antibacterial, antioxidant, and molecular docking analysis of some novel quinoline derivatives, Journal of Chemistry 2020(1) (2020) 1324096.
[2] O. Trott, A.J. Olson, AutoDock Vina: improving the speed and accuracy of docking with a new scoring function, efficient optimization, and multithreading, Journal of computational chemistry 31(2) (2010) 455-461.
[3] J.J. Blessy, D.J.S. Sharmila, Molecular simulation of N-acetylneuraminic acid analogs and molecular dynamics studies of cholera toxin-Neu5Gc complex, Journal of Biomolecular Structure and Dynamics 33(5) (2015) 1126-1139.
